# Scaffold Remodelling of Diazaspirotricycles Enables Synthesis of Diverse sp^3^‐Rich Compounds With Distinct Phenotypic Effects

**DOI:** 10.1002/chem.202203992

**Published:** 2023-03-22

**Authors:** Ephraim A. Okolo, Axel Pahl, Sonja Sievers, Christopher M. Pask, Adam Nelson, Stephen P. Marsden

**Affiliations:** ^1^ School of Chemistry University of Leeds Leeds LS2 9JT UK; ^2^ Max-Planck Institute of Molecular Physiology Department of Chemical Biology Otto-Hahn-Strasse 11 Dortmund 44227 Germany; ^3^ Astbury Centre for Structural Molecular Biology University of Leeds Leeds LS2 9JT UK

**Keywords:** dearomatisation, library synthesis, molecular diversity, phenotypic screening

## Abstract

A ‘top down’ scaffold remodelling approach to library synthesis was applied to spirotricyclic ureas prepared by a complexity‐generating oxidative dearomatisation. Eighteen structurally‐distinct, sp^3^‐rich scaffolds were accessed from the parent tricycle through ring addition, cleavage and expansion strategies. Biological screening of a small compound library based on these scaffolds using the cell‐painting assay demonstrated distinctive phenotypic responses engendered by different library members, illustrating the functional as well as structural diversity of the compounds.

## Graphical abstract

**Structural and functional diversity from simple precursors**: an efficient entry into a diverse sp^3^‐rich compound library was achieved by scaffold remodelling (ring cleavage, expansion and addition) of readily‐prepared spirotricyclic ureas. The resulting library shows significant scaffold diversity despite the common precursors, while individual compounds demonstrate distinctive phenotypic effects in the cell‐painting assay, suggesting the library is also functionally diverse.

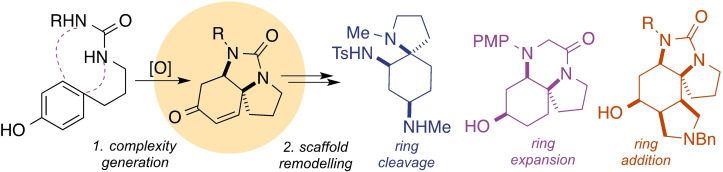

## Introduction

The availability of high‐quality, structurally‐diverse small molecule libraries is crucial to the discovery and development of new drugs and chemical probes. Much recent attention has been focused upon the developments of libraries of screening compounds^[1]^ and fragments^[2]^ with high degrees of sp^3^ character, to complement the more sp^2^‐rich nature of many commercial screening libraries.[^3^, ^4^] This latter facet is undoubtedly driven at least in part by the widespread use of synthetic methods (e. g. amide formation, Suzuki coupling)[^3^, ^5^] which can rapidly generate large libraries from widely‐available starting materials, but which often lead to ‘hit’ molecules which have poor properties for subsequent development into lead compounds. In some cases this is identified as a potential contributory factor to the high attrition rates of new small molecule drugs.^[4]^

A variety of complementary approaches to the synthesis of diverse sp^3^‐rich compound libraries has been taken. “Bottom‐up” diversity‐oriented synthetic approaches^[6]^ employ structurally and/or stereochemically‐diverse building blocks to generate sp^3^‐rich libraries.^[7]^ Alternatively, inspiration has been sought from natural products, which are inherently biologically‐relevant as well as structurally distinct from synthetic compounds.^[8]^ Generation of compound libraries based on sub‐structures of biologically‐validated natural products (‘pseudo‐natural products’) has proven highly productive in the discovery of new bioactive compound classes;^[9]^ alternatively, remodelling of natural product scaffolds themselves can generate distinct but related scaffold architectures with distinct activity.^[10]^

We have focused attention on a complementary approach, wherein complexity‐generating cycloaddition reactions have been used to rapidly generate highly‐functionalised polycylic scaffolds which can be further diversified by subsequent addition/modification, ring addition, ring expansion or ring cleavage.[^11^, ^12^] This approach facilitates the rapid assembly of diverse scaffold libraries that are entirely synthetic but distantly related to natural products:[^11a^, ^11b^] the biological relevance of these compounds has been demonstrated in, for example, the creation of fragment libraries providing crystallographic hits against distinct protein classes^[11a]^ and the identification of molecules active against the Hedgehog signalling pathway and as anti‐parasitic agents.^[11c]^

**Figure 1:**
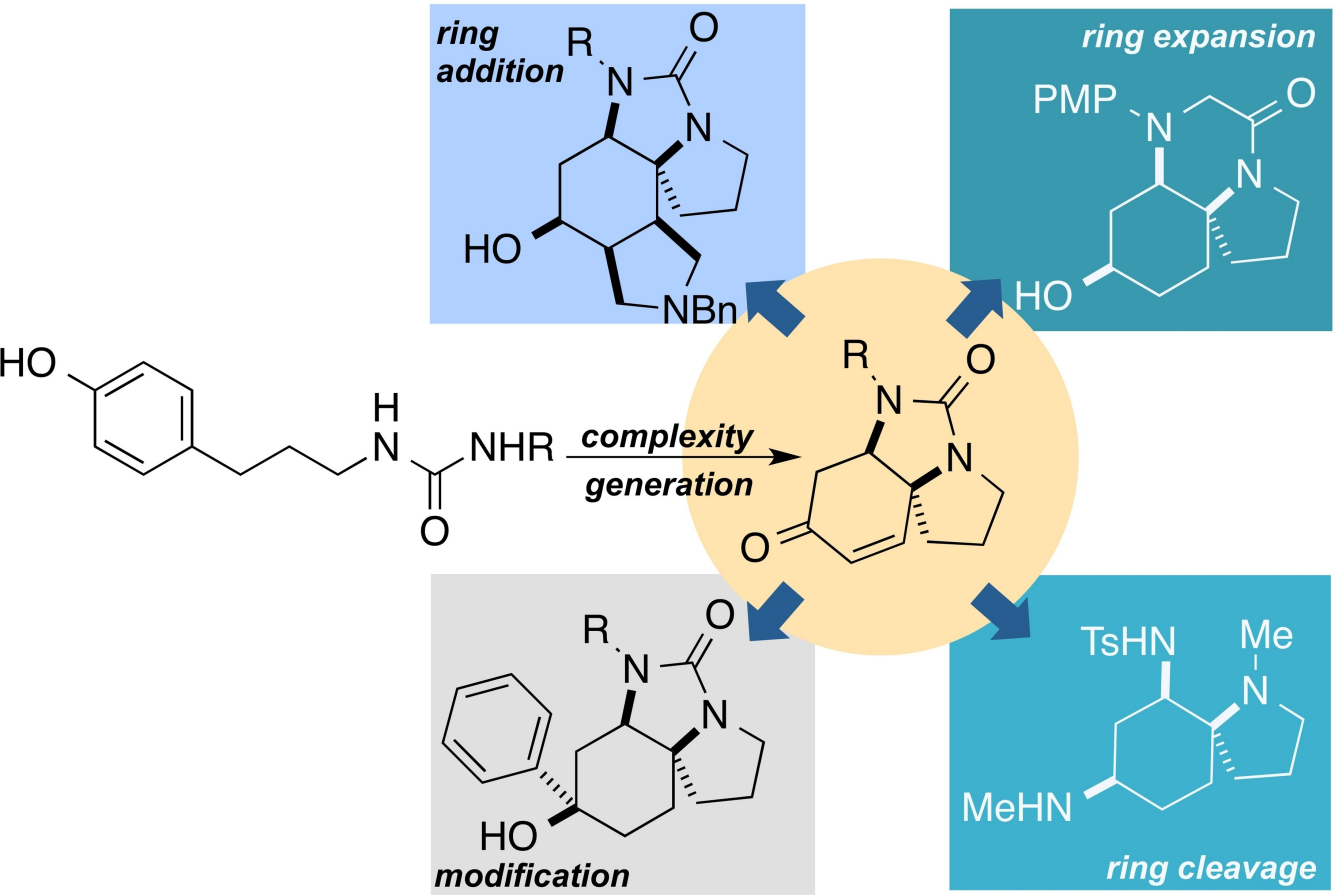
Schematic representation of ‘top down’ library synthesis based on scaffold remodelling of diazaspirotricycles.

At the heart of this approach is the need for highly efficient complexity‐generating reactions. Dearomatisation reactions can be efficient ways to rapidly increase molecular complexity, creating sp^3^‐rich 3D skeleta from simple sp^2^‐rich building blocks.^[13]^ We have recently developed an oxidative dearomatisation reaction that generates spirotricyclic diamines in a single step from readily‐available (hydroxyphenyl)propyl ureas.^[14]^ The resulting products contain rich functionality which we believed would be ideal for exploitation in a top‐down approach to the generation of diverse scaffolds (Figure 1). Herein we describe the successful realisation of this approach, and the generation of a small screening library based upon the scaffolds generated. The biological relevance of these scaffolds is demonstrated through phenotypic screening in a cell painting assay: distinct phenotypic effects are observed for compounds based on scaffolds which are structurally distinct but synthetically closely‐related.

## Results and Discussion

### Synthesis and manipulation of spirotricyclic ureas

**Scheme 1:**
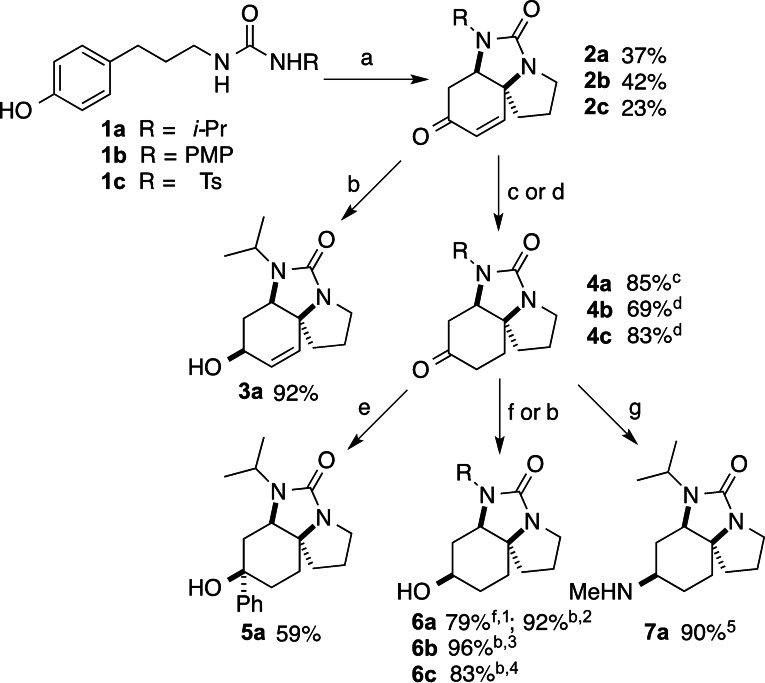
Synthesis and structural manipulation of the core spirotricyclic ureas **2**. Compound suffixes: R=*i*‐Pr (**a**), PMP (**b**), Ts (**c**). (a) 1.1 equiv. PIFA, CH_2_Cl_2_/HFIP; (b) NaBH_4_, CeCl_3_, MeOH, −78 °C; (c) 2 % [(Ph_3_P)_3_RhCl], Et_3_SiH, THF, then aq. HCl; (d) 20 % w/w Pd(OH)_2_/C, H_2_ (1 atm.), EtOAc; (e) PhLi, Et_2_O; (f) LiAlH_4_, THF, r.t.; (g) MeNH_2_, Ti(OiPr)_4_, EtOH, r.t. then NaBH_4_, −78 °C to r.t.; ^1^ 93 : 7 dr; ^2^ 100:0 dr; ^3^ 85 : 15 dr; ^4^ 78 : 22 dr; ^5^ 88 : 12 dr.

We designed spirotricyclic ureas **2 a**–**c** bearing structurally and electronically‐diverse *N*‐urea substituents as our basic building blocks for scaffold elaboration (note: throughout, the suffixes **a**, **b** and **c** refer to structures bearing 2‐propyl, 4‐methoxyphenyl and *p‐*toluenesulfonyl substituents respectively). The requisite ureas were prepared using our previously‐developed oxidative dearomatisation^[14]^ using the corresponding hydroxyphenyl ureas **1 a**–**c** (see Supporting Information), Scheme 1. Although the oxidative dearomatisation reactions were moderate yielding, the short nature of the synthetic route allowed ready supply of material for further study.

Before moving on to scaffold diversification, we demonstrated functionalisation of the enone functionality in the spirotricyclic ureas. Chemo‐ and stereoselective reduction to allylic alcohol was achieved under Luche conditions,^[15]^ giving a single diastereomer **3 a**, whose stereochemistry was determined crystallographically. Enones **2 a**–**c** could be reduced to the saturated derivatives **4 a**–**c** in excellent yields either by rhodium‐catalysed conjugate reduction with triethylsilane or by hydrogenation over Pd(OH)_2_/C. Nucleophilic addition to the resulting ketones was also highly stereoselective: addition of a Grignard reagent to **4 a** gave a single diastereomeric alcohol **5 a**, while reduction could be most effectively achieved using sodium borohydride with added cerium(III) chloride,^[16]^ again giving a single diastereomeric product **6 a** (use of lithium aluminium hydride was both lower yielding and less selective). Reductions of **4 b** and **4 c** were similarly high‐yielding but slightly less selective. Finally, stereoselective conversion to the secondary amine **7 a** was best achieved by pre‐formation of the corresponding imine and reduction with sodium borohydride, giving an 88 : 12 mixture of disastereomers. The stereochemistry of alcohol **6 a** and amine **7 a** was unambiguously determined by X‐ray crystallography of suitable derivatives (see Supporting Information). Analysis of crystal structures of the saturated ketones **4 b** and **4 c** (Supporting Information) shows that they adopt boat‐like conformations about the cyclohexanone ring: the stereochemical outcome of the addition reactions is consistent with attack on the less‐hindered exocyclic face of the carbonyl or derived imine.

### Scaffold diversification by ring addition

**Scheme 2:**
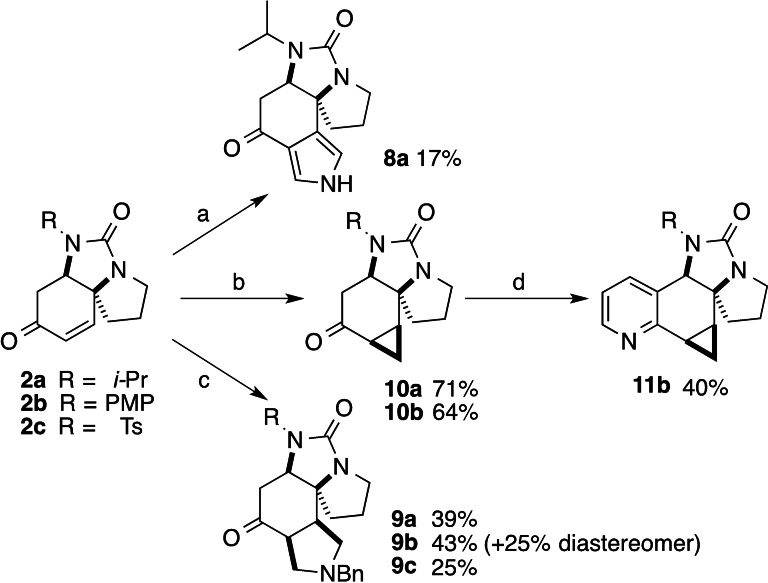
Scaffold diversification by ring addition reactions. Compound suffixes: R=*i*‐Pr (**a**), PMP (**b**), Ts (**c**). (a) KOtBu, TosMIC, THF; (b) (Me_3_S=O)Cl, NaH, THF; (c) (MeOCH_2_)(TMSCH_2_)NBn, LiF, MeCN; (d) 12 % NaAuCl_4_, propargylamine, EtOH, reflux.

We began investigations into the diversification of ureas **2** to novel scaffolds through ring addition reactions to the enone (Scheme 2). Reaction with TosMIC and potassium *tert*‐butoxide^[17]^ gave a low yield of pyrrole **8 a**.

Construction of a saturated pyrrolidine ring could be achieved by [3+2] cycloaddition of an in situ generated azomethine ylide.^[18]^ Adducts **9 a** and **9 c** were isolated as single diastereoisomers, while the PMP‐derivatives **9 b** were formed as separable diastereomers (ca. 2 : 1 ratio). Although minor diastereomers of **9 a**/**c** were neither isolated nor unambiguously observed in the complex crude ^1^H NMR spectra, we cannot rule out their presence in the crude reaction mixtures. The stereochemistry of the major isomers was determined by NMR analysis (NOE and analysis of coupling constants) of the derived alcohols (Supporting Information). Cyclopropanation of the enone was performed under Corey‐Chaykovsky conditions,^[19]^ giving adducts **10 a**/**b** in good yield as single diastereoisomers following chromatography (small amounts of an apparent minor diastereomer were visible in the ^1^H NMR of the crude). The relative configuration of **10 a** was unambiguously assigned by X‐ray crystallography, supporting the preferential attack on the enone *syn*‐to the diamine function, an outcome also consistent with that assigned in the azomethine cycloadditions above.

We also sought to exploit the ketone functionality to perform annulations. While reaction of the saturated ketone **4 b** with propargylamine under gold catalysis^[20]^ gave an inseparable mixture of regioisomeric pyridines, the corresponding annulation using the cyclopropyl ketone **10 b** was successful, giving a moderate yield of pyridine **11 b** as a single isomer.

### Scaffold diversification by ring cleavage

**Scheme 3:**
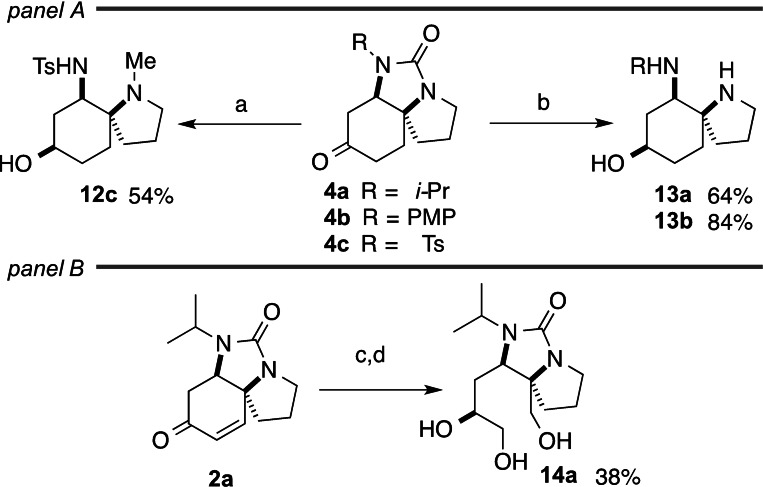
Scaffold diversification by ring‐cleavage reactions. Compound suffixes: R=*i*‐Pr (**a**), PMP (**b**), Ts (**c**). a) LiAlH_4_, THF, reflux; b) LiAlH_4_, THF, reflux; then NH_2_OH.HCl, cat. HCl; c) NaBH_4_, CeCl_3_, MeOH; then TBSOTf, 2,6‐lutidine, CH_2_Cl_2_; d) O_3_, CH_2_Cl_2_, −78 °C; then MeOH, NaBH_4_, −78 °C; then TBAF, THF.

Two targets were identified for scaffold diversification of the parent spirotricycles **2** through ring cleavage: the urea and the alkene. Cleavage of the ureas could be conveniently achieved by lithium aluminium hydride treatment of saturated ketones **4** (Scheme 3, panel A). The *N*‐tosyl urea **4 c** underwent direct reductive cleavage (and accompanying ketone reduction) to reveal the *N*‐methyl spirobicyclic diamine **12 c**. The *N‐iso*‐propyl and *N*‐(4‐methoxyphenyl) ureas **4 a** and **4 b**, by contrast, gave spirotricyclic imidazolidines as the initial products: cleavage of the aminal by treatment with hydroxylamine hydrochloride gave the spirobicyclic diamines **13 a**/**b**. The structures of all three ring‐cleaved products were determined crystallographically. The divergent behaviour of the ureas is presumably the result of expulsion of a sulfonamidyl anion from the initial hydride addition product from **4 c**, whereas the less nucleofugal aliphatic and arylamino groups may remain in the ring‐closed form in the adducts of hydride addition to **4 a**/**b**.

The alkene functionality in the spirotricyclic scaffolds **2** was cleaved by ozonolysis of the TBS ether of the allylic alcohol formed by Luche reduction of **2 a**. Reductive work‐up with sodium borohydride and deprotection of the silyl ether with TBAF gave the fused azabicyclic triol **14 a** (Scheme 3, panel B).

### Scaffold diversification by ring expansion

**Scheme 4:**
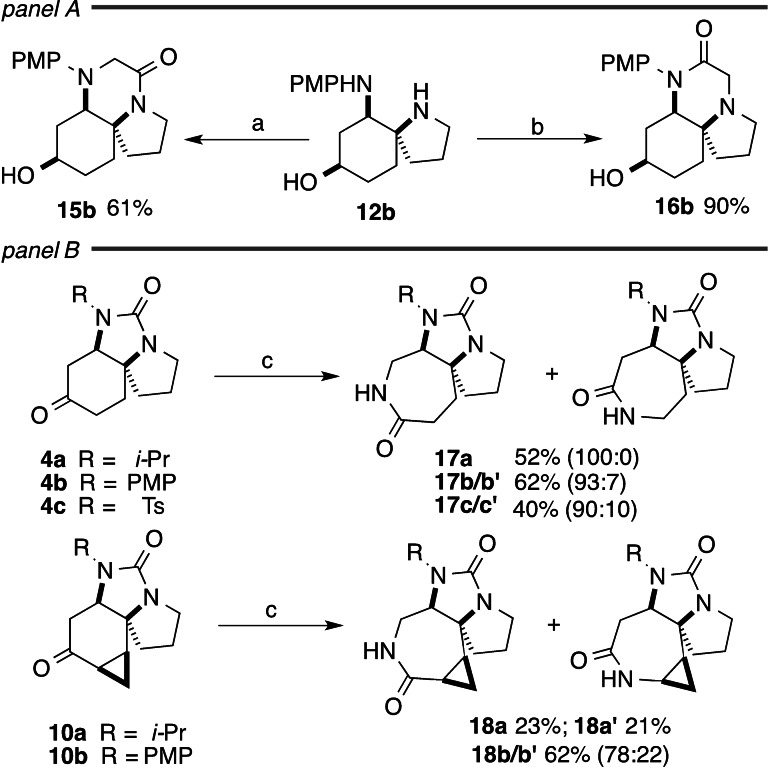
Scaffold diversification by ring expansion. Compound suffixes: R=*i*‐Pr (**a**), PMP (**b**), Ts (**c**). a) ClCH_2_COCl, DIPEA, THF; b) BrCH_2_CO_2_Me, KHCO_3_, THF; then NaOMe, MeOH; c) NH_2_OH.HCl, K_2_CO_3_, EtOH/H_2_O, reflux; then TsCl, DMAP, Et_3_N, CH_2_Cl_2_; then cat. H_2_SO_4_, MeOH, reflux; ^1^ 93 : 7 regio; ^2^ 90 : 10 regio; ^3^ 78 : 22 regio.

Finally, we examined ring expansion as an entry to alternative scaffolds and again both the urea and the cyclohexanone rings embedded in spirotricycles **2** were targeted. Formal insertion of a methylene unit to the urea was achieved by reaction of the diamine **12 b** with complementary α‐haloacetic acid derivatives (Scheme 4, panel A). Thus, reaction with chloroacetyl chloride proceeds directly to piperazinone **15 b**, presumably via initial acylation of the pyrrolidine followed by internal substitution of the aliphatic carbon by the less nucleophilic aryl amine. The regiocomplementary outcome could be achieved by initial alkylation of the pyrrolidine with methyl bromoacetate, followed by base‐mediated amidation to give **16 b**. Ring expansion of the saturated ketones **4 a**–**c** was achieved by Beckmann rearrangement, giving the tricyclic lactams **17 a**–**c** (Scheme 4, panel B). The reactions were unexpectedly regioselective: lactam **17 a** was formed as a 93 : 7 mixture of regioisomers as judged from the crude ^1^H NMR, but isolated as a single compound, whose structure was determined crystallographically. Lactams **17 b**/**c** gave slightly less regioselective rearrangements (3 : 1 and 2 : 1 in the ^1^H NMR of the crude) but were isolated as predominantly the isomer shown. In an attempt to access the regiocomplementary lactams, we examined the Beckmann rearrangement of the cyclopropyl ketones **4 a**/**b**, reasoning that substitution in the form of the cyclopropyl group might bias the oxime geometry to place the leaving group *anti*‐ to the more‐substituted carbon. In the event, however, migration of the other carbon was still favoured, giving **18 a**/**18 b** as the predominant isomers, albeit with significant formation of the isomeric products **18 a′** and **18 b′**. It is possible that the impact of additional substitution on migratory aptitude in the case of the cyclopropanes is somewhat negated by the change in hybridisation (more sp^2^‐like) at the migrating carbon.

### Library synthesis and biological evaluation

**Figure 2:**
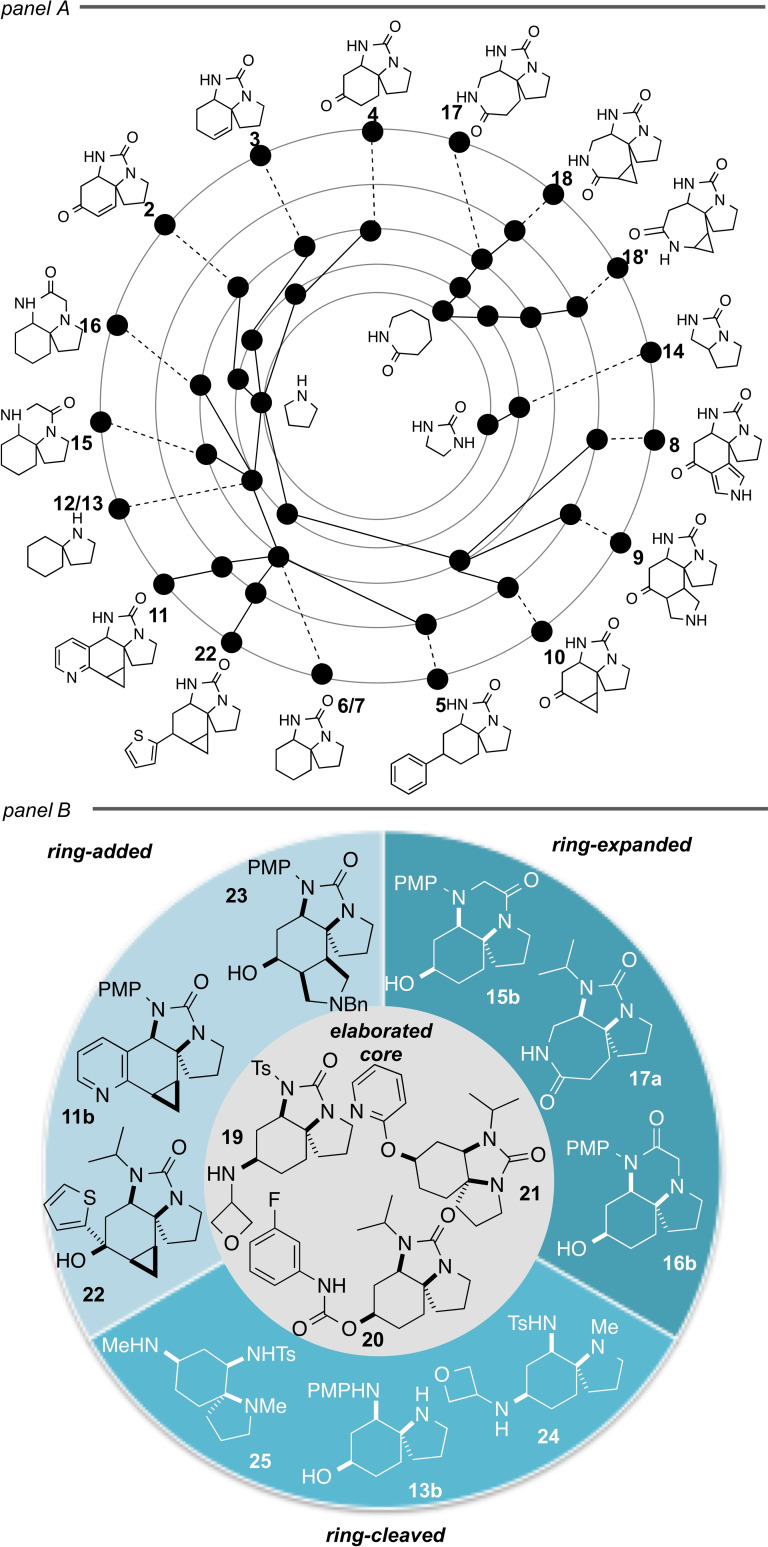
Panel A: Hierarchical scaffold tree showing the diversity of, and relationship between, the scaffolds prepared. The represented frameworks (outer ring) and the iteratively simplified frameworks (inner rings) are represented by black circles. Panel B: Selection of the screening compounds prepared from the diverse scaffolds.

Our ‘top down’ approach has enabled the rapid conversion of the complex spirotricyclic intermediates **2** into a wide range of distinct molecular scaffolds.^[21]^ The hierarchical relationship between the 18 represented frameworks, in which only doubly‐bonded alpha atoms are retained, may be determined by systematic simplification using established iterative prioritisation rules (Figure 2, Panel A and Supporting Information).^[22]^ This analysis reveals significant diversity at the higher levels, despite the majority of the frameworks ultimately resolving to the same parent monocycle (pyrrolidine). The ability to cleave and expand rings within the complex intermediates **2**, and to append additional rings, was critical to realising this scaffold diversity.

We next sought to demonstrate the utility of these scaffolds in the synthesis of a small screening library. A library of 51 compounds was prepared; a selection of these compounds is shown in Figure 2, Panel B (see Supporting Information for the full library and synthetic details). The scaffolds **4 a**–**c** could be directly elaborated by reductive amination alone (e. g. to **19**) or with an additional acylation; and the corresponding alcohols **6 a**–**c** could also be converted to carbamates (e. g. to **20**), alkylated or (hetero)arylated (e. g. to **21**). Similar functionalisations, as well as addition of carbon nucleophiles (e. g. to give **22**), could also be carried out on the ring‐added, ring‐expanded or ring‐cleaved products (giving for example **23**–**25**). The library was further analysed for three‐dimensionality (PMI plot), lead‐likeness, and the fraction of sp^3^‐hybridised carbons (Fsp^3^) (Supporting Information), with >94 % of compounds having Fsp^3^>0.5.

**Figure 3:**
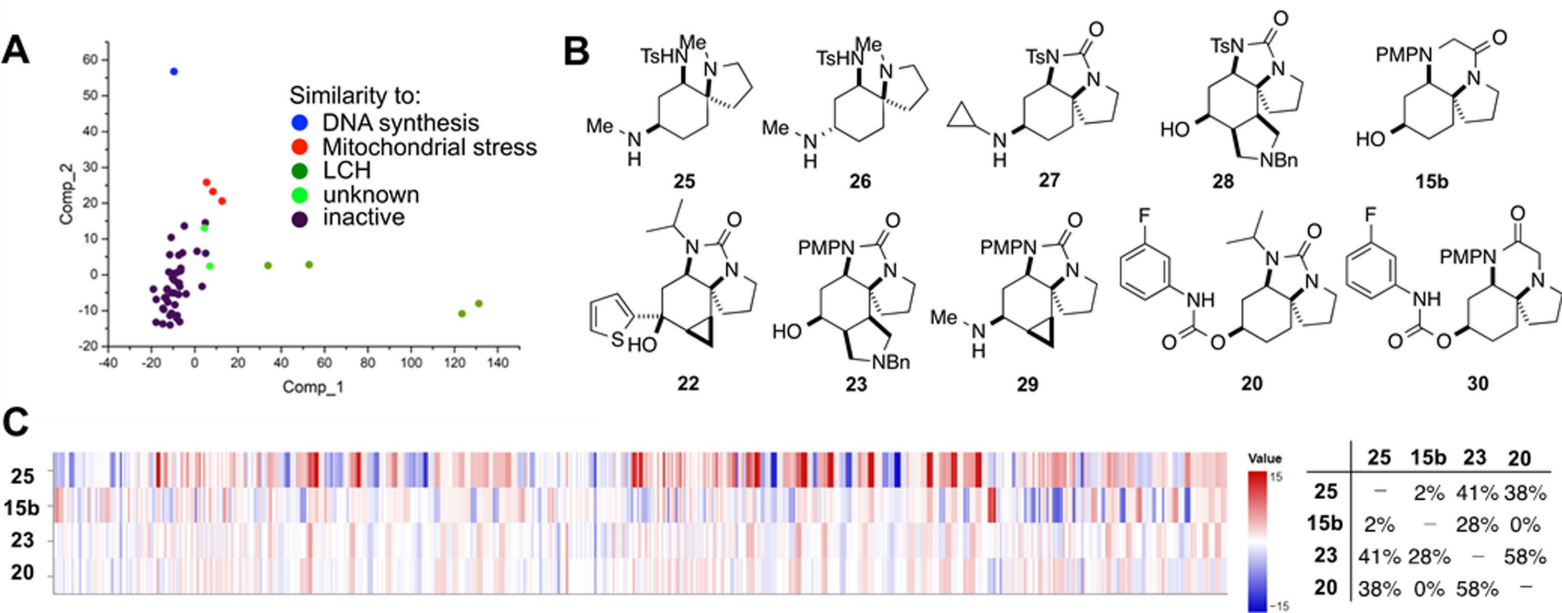
Evaluation of the library of 52 compounds (30 μM) in the cell painting assay. Panel A: Dimension reduction analysis (PCA) of data for the screened compounds (30 μM), explained variance: PC1 (59.5 %), PC2 (11.2 %), PC3 (7.7 %). The most similar functional cluster is shown by colour: lysosomotropism/cholesterol homeostasis (dark green), mitochondrial stress (red) and DNA synthesis (blue). Compounds that were not identified as hits (purple; induction <5%), and compounds that with <70 % similarity to any functional cluster (light green) are also shown. Panel B: Structures of hit compounds. Panel C: Fingerprints of selected bioactive compounds visualised as heatmap profiles (left), and pairwise similarities between fingerprints (biosimilarities) (right). The set of 579 parameters is divided into parameters related to the cell (1–229), cytoplasm (230–461) and nuclei (462–579). Values were normalised to the DMSO control. blue: decreased parameter. red: increased parameter.

To enable an assessment of biological relevance, the library was screened in the cell painting assay which interrogates many biological pathways simultaneously.^[23]^ Here, six different dyes were used to stain different cellular compartments; high‐content imaging and automated image analysis then enabled extraction and quantification of 579 parameters corresponding to specific morphological features. The biological profile of compounds may be described in terms of fingerprints which capture the changes in these parameters relative to DMSO control. Morphological fingerprints were determined for the 52 screening compounds at 10, 30 and 50 μM, and were compared with those of around 4000 reference compounds (mainly determined at 10 μM). Bioactivity was assessed in terms of an induction value: that is, the percentage of significantly changed parameters (median absolute deviation of the parameter value >±3) relative to DMSO control. An established[^23b^, ^24^] dimension reduction analysis of data for the screened compounds at 30 μM was performed, and is presented in Panel A (Figure 3). At this concentration, we identified ten compounds that were considered to be active (induction values >5%; Panels A, B, Figure 3). Comparison of fingerprints revealed clusters^[23c]^ of compounds with distinct morphological effects: these effects were similar to those of known modulators of lysosomotropism/cholesterol homeostasis (**25**–**28**),^[25]^ DNA synthesis (**15 b**)^[26]^ and mitochondrial stress (**22**, **23**, **29**). In addition, two compounds (**20** and **30**) were not similar to previously identified functional clusters (similarity <70 %). The fingerprints for the compounds with the highest induction value from each of these clusters (**25**, **15 b**, **23** and **20**) are displayed as heatmap profiles in Figure 3 (Panel C, left). Crucially, pairwise comparison, based on Pearson correlation, revealed that the morphological fingerprints for these compounds have low biosimilarity (Panel C, right) (fingerprints are typically considered similar if biosimilarity >75 %^[27]^). These results suggest that our top‐down synthetic approach can deliver sets of screening compounds are functionally (as well as structurally) diverse.^[28]^

## Conclusion

A ‘top down’ approach to the synthesis of sp^3^‐rich libraries has been demonstrated based on scaffold remodelling of diazaspirotricyclic compounds available in a single step from simple sp^2^‐rich precursors via a complexity‐generating dearomatising cyclisation cascade. Compounds based on eighteen structurally distinct skeleta were created through ring‐addition, expansion and cleavage pathways. Screening of a small 51‐membered compound library in the cell painting assay revealed compounds active for the induction of different distinct morphological changes, suggesting a diversity of modes of action. Our approach is therefore capable of efficiently generating compounds which are functionally as well as skeletally diverse.

## Conflict of interest

The authors declare no conflict of interest.

1

## Supporting information

As a service to our authors and readers, this journal provides supporting information supplied by the authors. Such materials are peer reviewed and may be re‐organized for online delivery, but are not copy‐edited or typeset. Technical support issues arising from supporting information (other than missing files) should be addressed to the authors.

Supporting Information

## Data Availability

The data that support the findings of this study are available in the supplementary material of this article.
