# Supplementary material for: Scaffold Remodelling of Diazaspirotricycles Enables Synthesis of Diverse sp3‐Rich Compounds With Distinct Phenotypic Effects
Source: Chemistry. 2023 Mar 22;29(26):e202203992. doi: 10.1002/chem.202203992 (PMC10946999; doi:10.1002/chem.202203992)
Supplement: Supplementary file 1 — Supporting Information [file CHEM-29-0-s001.pdf]

Chemistry–A European Journal

Supporting Information

Scaffold Remodelling of Diazaspirotricycles Enables Synthesis of Diverse sp^3-Rich Compounds With Distinct Phenotypic Effects

Ephraim A. Okolo, Axel Pahl, Sonja Sievers, Christopher M. Pask, Adam Nelson,* and Stephen P. Marsden*

Supporting Information

**Page**	**Contents**
1	Table of contents
2	General experimental
	Experimental procedures and compound data:
3-33	Starting materials and products for Schemes 1-4
34-62	Structures and synthesis of screening compounds
63-64	Molecular property analysis of screening compounds
65-151	^{1}H and ^{13}C NMR spectra
152-154	Cell painting assay
155-165	X-ray structures
166	References

General Experimental

All non-aqueous reactions were performed under an atmosphere of nitrogen unless otherwise stated. Water-sensitive reactions were performed in oven-dried glassware, cooled under nitrogen before use. Solvents were removed *in vacuo* using a Büchi rotary evaporator and a Vacuubrand PC2001 Vario diaphragm pump. A Genevac EZ-2 Elite centrifugal evaporator was used for the removal of MeOH–H_2O after Mass-Directed purification. Tetrahydrofuran (THF), CH_2Cl_2, toluene and CH_3CN were dried and purified by means of a Pure Solv MD solvent purification system (Innovative Technology Inc.). Anhydrous *N,N*-dimethylacetamide (DMA), *N,N*-dimethylformamide (DMF) and 1,4-dioxane was obtained in SureSeal bottles from Sigma-Aldrich. All other solvents used were of chromatography or analytical grade. Petrol refers to petroleum spirit (b.p. 40-60 °C). Commercially available starting materials were obtained from Sigma-Aldrich, Fluka, Acros or Alfa-Aesar and were used without purification unless stated.

Thin layer chromatography (TLC) was carried out on aluminium backed silica (Merck silica gel 60 F_{254}) plates supplied by Merck. Visualisation of the plates was achieved using an ultraviolet lamp (λ_{max} = 254 nm), $KMnO_4$, anisaldehyde or ninhydrin. LCMS analysis was generally carried out on an Agilent 1200 series LC system comprising a Bruker HCT Ultra ion trap mass spectrometer. The solvent system used was CH_3CN/H_2O + 0.1% formic acid with a Phenomenex Luna C18 50 × 2 mm 5 micron column. Flash chromatography was carried out using silica gel 60 (60-63 µm particles) supplied by Merck or using Biotage silica or ISOLUTE C_{18} pre-packed cartridges on a Flashmaster II or CombiFlash Companion. Strong cation exchange solid phase extraction (SCX-SPE) was carried out using pre-packed Discovery DSC-SCX cartridges supplied by Supleco. Mass-directed HPLC purification was carried out using an Agilent 1260 Infinity HPLC system comprising an Agilent 6120 Quadrupole LC/MS and Agilent G1968D active splitter.

High resolution mass spectra (HRMS) were recorded on a Bruker Daltonics micrOTOF or Bruker MaXis Impact spectrometer with electrospray ionisation (ESI) source. Where EI ionisation was required, a Waters/Micromass GCT Premier spectrometer was used.

Proton (^1H) and carbon (^{13}C) NMR spectral data were collected on a Bruker Advance 400, 500 or 600, Bruker DPX500 or DPX300 spectrometers. Carbon spectra were recorded using proton decoupling. Chemical shifts (δ) are quoted in parts per million (ppm) and referenced to the residual solvent peak. Coupling constants (*J*) are quoted in Hertz (Hz) and splitting patterns reported in an abbreviated manner: app. (apparent), s (singlet), d (doublet), t (triplet), q (quartet), m (multiplet). Assignments were made with the aid of COSY, DEPT-135, HMQC, HMBC and NOESY experiments.

Synthesis of Cyclisation Precursors 1a-c

1-(Benzyloxy)-4-iodobenzene S1

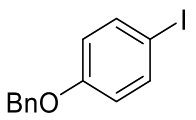

To a mixture of 4-iodophenol (9.68 g, 44.0 mmol, 1.0 eq.) and potassium carbonate (7.91 g, 57.2 mmol, 1.3 eq.) in HPLC grade acetonitrile (20 mL), benzyl bromide (5.75 mL, 48.4 mmol, 1.1 eq.) was added and the mixture was stirred at room temperature for 20 h. The reaction mixture was filtered through a sintered funnel washing with 300 mL DCM and concentrated *in vacuo*. Recrystallisation from hexane afforded a brown solid (12.8 g, 41.3 mmol, 94%); $\mathbf{R_f}$ = 0.54 (5% EtOAc in hexane). The NMR data is in agreement with the literature.[1] ^{1}**H NMR** (400 MHz, $CDCl_3$): δ ppm 7.59 (2H, d, *J* = 8.8, Ar*H*), 7.46 – 7.37 (5H, m, Ar*H*), 6.79 (2H, d, *J* = 8.8, Ar*H*), 5.07 (2H, s, ArC*H₂*). ^{13}**C NMR** (100 MHz, $CDCl_3$): δ 158.7 (*Ar*), 138.3 (*Ar*H), 136.6 (*Ar*), 128.7 (*Ar*H), 128.2 (*Ar*H), 127.5 (*Ar*H), 117.4 (*Ar*H) 83.1 (*Ar*I), 70.1 (Ar*C*H_2). **IR** v_{max} (neat)/cm^{-1}: 3062, 3030, 2928, 2874 (C-H), 1579, 1481, 1378 (C=C), 1235 (C-O).

3-[(4-Benzyloxy)phenyl]propanal S2

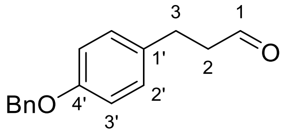

To a mixture of sodium bicarbonate (10.8 g, 129 mmol, 2.0 eq.), tetrabutylammonium chloride (17.9 g, 64.6 mmol, 1.0 eq.), palladium acetate (146 mg, 0.646 mmol, 1 mol%) and 1-(benzyloxy)-4-iodobenzene **S1** (20.0 g, 64.6 mmol, 1.0 eq.) in anhydrous DMF (65.5 mL) was added allyl alcohol (6.59 mL, 96.8 mmol, 1.5 eq.) and the mixture was heated at 50 °C for 20 h. The reaction mixture was filtered through a plug of Celite eluting with EtOAc (400 mL) and evaporated *in vacuo*. The filtrate was extracted with EtOAc (5 × 100 mL) in water (50 mL), washed with brine (100 mL), dried over Na_2SO_4 and evaporated *in vacuo*. Purification by flash chromatography on silica gel eluting with 10 – 20% EtOAc in hexane followed by recrystallisation from Et_2O afforded a brown solid (12.8 g, 53.1 mmol, 82% yield); $\mathbf{R_f}$ = 0.55 (20% EtOAc in hexane). The NMR data is in agreement with the literature.[2] ^{1}**H NMR** (400 MHz, $CDCl_3$): δ ppm 9.84 (1H, t, *J* = 1.3, 1-*H*), 7.51 – 7.35 (5H, m, Ar*H*), 7.17 (2H, d, *J* = 8.4, Ar*H*), 6.97 (2H, d, *J* = 8.8, Ar*H*), 5.09 (2H, s, ArC*H*$_2$O), 2.95 (2H, t, *J* = 7.5, 3-$H_{A,B}$),

2.78 (2H, td, *J* = 7.6, 1.2, 2-$H_{A,B}$). **^{13}C NMR** (100 .MHz, $CDCl_3$): δ 201.8 (1-*C*), 157.4 (*Ar*), 137.2 (*Ar*), 132.7 (*Ar*), 129.3 (*Ar*H), 128.6 (*Ar*H), 128.0 (*Ar*H), 127.5 (*Ar*H), 115.1 (*Ar*H), 70.1 (Ar*C*H_2O), 45.5 (3-*C*), 27.4 (2-*C*). **IR** ν_{max} (neat)/cm^{-1}: 3067, 3033, 2925, 2859, 2733 (C-H), 1716 (C=O), 1608, 1579, 1510 (C=C), 1234 (C-O).

3-(4-(Benzyloxy)phenyl)propan-1-amine S3

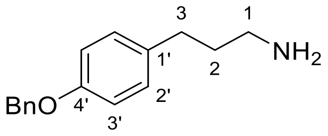

A mixture of 3-[(4-benzyloxy)phenyl]propanal **S2** (300 mg, 1.25 mmol, 1.0 eq.) and hydroxylamine hydrochloride (104 mg, 1.50 mmol, 1.2 eq.) in 3 mL of anhydrous EtOH was allowed to stir for 1 h at room temperature. 12 M HCl (0.42 mL, 5.0 mmol, 4.0 eq.) was then added to the reaction mixture followed by zinc dust (205 mg, 3.13 mmol, 2.5 eq.) and the mixture was left to stir for 40 min, after which 0.36 mL of 30% aqueous ammonia and 0.77 mL of 6 M NaOH were added. The reaction mixture was extracted with DCM (5 × 20 mL), dried over Na_2SO_4 and concentrated *in vacuo*. Purification by silica gel chromatography eluting with 3 – 7% MeOH in DCM followed by 3 – 10% of saturated NH_3/MeOH in DCM, afforded a white solid (132 mg, 0.55 mmol, 44% yield); **R_f** = 0.39 (6% NH_3/MeOH in DCM). **^{1}H NMR** (300 MHz, MeOD): δ ppm 7.34 – 7.14 (5H, m, Ar*H*), 7.00 (2H, d, *J* = 8.6, Ar*H*), 6.79 (2H, d, *J* = 8.7, Ar*H*), 4.93 (2H, s, ArOC*H₂*), 2.56 (2H, t, *J* = 7.4, 1-$H_{A,B}$), 2.49 (2H, t, *J* = 7.6, 3-$H_{A,B}$), 1.66 (2H, quint, *J* = 7.5, 2-$H_{A,B}$). **^{13}C NMR** (75 MHz, MeOD): δ ppm 158.4 (*Ar*), 138.9 (*Ar*), 135.3 (*Ar*), 130.2 (*Ar*H), 129.4 (*Ar*H), 128.7 (*Ar*H), 128.4 (*Ar*H), 115.9 (*Ar*H), 71.0 (ArO*C*H_2), 41.7 (1-*C*), 34.9 (3-*C*), 33.2 (2-*C*). **IR** ν_{max} (neat)/cm^{-1}: 3366 (N-H), 3061, 3032, 2927, 2857 (C-H), 1235 (C-O). **HRMS** (ESI): $C_{16}H_{20}NO$ [M + H^+]: calculated 242.1539, found 242.1538.

1-(3-(4-(Benzyloxy)phenyl)propyl)-3-isopropylurea S4

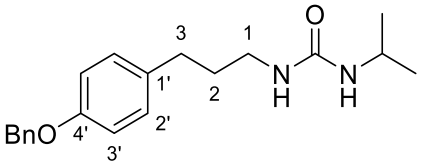

Isopropyl isocyanate (0.63 mL, 6.45 mmol, 1.1 eq.) was added to a solution of 3-(4-(benzyloxy)phenyl)propan-1-amine **S3** (1.41 g, 5.86 mmol, 1.0 eq.) in anhydrous DCM (33 mL) and the mixture was refluxed for 5 h. The reaction mixture was then evaporated *in vacuo.* Flash chromatography with 50 – 100% EtOAc in hexane afforded the compound as a white solid (1.71 g, 5.25 mmol, 90% yield); $\mathbf{R_f}$ = 0.75 (100% EtOAc). **^{1}H NMR** (300 MHz, MeOD): δ ppm 7.36 – 7.14 (5H, m, Ar*H*), 7.00 (2H, d, *J* = 8.4, Ar*H*), 6.80 (2H, d, *J* = 8.7, Ar*H*), 4.93 (2H, s, ArOCH_2), 3.70 (1H, hept, *J* = 6.5, isopropyl C*H*), 3.01 (2H, t, *J* = 7.0, 1-$H_{A,B}$), 2.47 (2H, t, *J* = 7.5, 3-$H_{A,B}$), 1.64 (2H, quint, *J* = 7.3, 2-$H_{A,B}$), 1.02 (6H, d, *J* = 6.5, isopropyl CH_3). **^{13}C NMR** (75 MHz, MeOD): δ ppm 160.5 (urea *C*=O), 158.4 (*Ar*), 138.9 (*Ar*), 135.4 (*Ar*), 130.3 (*Ar*H), 129.4 (*Ar*H), 128.7 (*Ar*H), 128.5 (*Ar*H), 115.8 (*Ar*H), 71.0 (ArOCH_2), 42.8 (isopropyl-*C*H), 40.4 (1-*C*), 33.3 (3-*C*), 33.2 (2-*C*), 23.5 (isopropyl CH_3). **IR** ν_{max} (neat)/cm^{-1}: 3328 (N-H), 2964, 2935, 2864 (C-H), 1726 (C=O), 1236 (C-O). **HRMS** (ESI): $C_{20}H_{27}N_2O_2$ [M + H$^+$]: calculated 327.2067, found 327.2065.

1-[3-(4-Hydroxyphenyl)propyl]-3-isopropylurea 1a

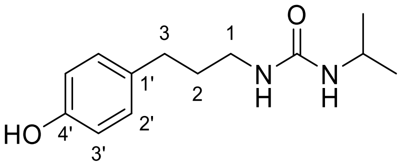

Debenzylation:

To a mixture of Pd(OH)$_2$/C (10.1 mg, 10% w/w) and 1-(3-(4-(benzyloxy)phenyl)propyl)-3-isopropylurea **S4** (101 mg, 0.31 mmol, 1.0 eq.) under an atmosphere of nitrogen, 5 mL of MeOH was added gently. The reaction mixture was degassed and hydrogen gas was bubbled through it with the aid of a balloon, and this procedure was repeated twice. The mixture was then allowed to stir under a balloon of hydrogen for 23 h at room temperature. The reaction mixture was filtered through a plug of Celite washing with 100 mL MeOH. The filtrate was evaporated *in vacuo*. Flash chromatography eluting with 80 - 100% EtOAc in hexane afforded the product as a colourless oil (70 mg, 0.30 mmol, 92%).

Urea formation:

Isopropyl isocyanate (1.64 mL, 16.7 mmol, 1.01 eq.) was added to a solution of the 4-(3-aminopropyl)phenol (2.50 g, 16.6 mmol, 1.0 eq.) in anhydrous THF and the mixture was refluxed for 1 h. The reaction mixture was evaporated *in vacuo.* Flash chromatography with 50 – 100% EtOAc in hexane afforded the product as a colourless oil (3.24 g, 13.6 mmol, 83% yield); **R_f** = 0.55 (100% EtOAc). **^{1}H NMR** (500 MHz, MeOD): δ ppm 7.02 (2H, d, *J* = 8.4, Ar*H*), 6.74 (2H, d, *J* = 8.4, Ar*H*), 3.82 (1H, hept, *J* = 6.5, isopropyl C*H*), 3.13 (2H, t, *J* = 7.0, 1-$H_{A,B}$), 2.53 (2H, t, *J* = 7.5, 3-$H_{A,B}$), 1.76 (2H, quint, *J* = 7.5, 2-$H_{A,B}$), 1.14 (6H, d, *J* =6.6, isopropyl CH_3). **^{13}C NMR** (125 MHz, MeOD): δ ppm 160.7 (urea *C*=O), 156.5 (*Ar*), 134.0 (*Ar*), 130.4 (*Ar*H), 116.3 (*Ar*H), 43.0 (isopropyl *C*H), 40.6 (1-*C*), 33.6 (3-*C*), 33.3 (2-*C*), 23.7 (isopropyl *C*H$_3$). **IR** ν_{max} (neat)/cm^{-1}: 3334 (O-H, N-H), 3014, 2969, 2931, 2872 (C-H), 1558, 1514, 1455 (C=C), 1240 (C-O).**HRMS** (ESI): $C_{13}H_{21}N_2O_2$ [M + H$^+$]: calculated 237.1598, found 237.1592

***N*-Benzyl-3-[4-(benzyloxy)phenyl]propan-1-amine S5**

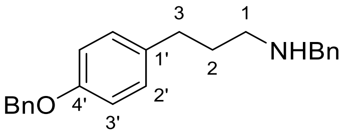

To a solution of 3-[(4-benzyloxy)phenyl]propanal **S2** (1.00 g, 4.16 mmol, 1.0 eq.) in anhydrous MeOH (8.40 ml), BnNH_2 (0.60 ml, 5.49 mmol, 1.3 eq.) was added and the mixture was allowed to stir for 1.5 h at room temperature. Sodium borohydride (79.4 mg, 2.10 mmol, 0.50 eq.) was then added and stirring continued at room temperature for 2.5 h. The reaction mixture was extracted with DCM (5 × 20 mL), dried over Na_2SO_4 and evaporated *in vacuo*. Flash chromatography on silica gel eluting with 5 – 10% MeOH in DCM afforded a thick brown oil (1.14 g, 3.44 mmol, 83%); **R_f** = 0.39 (100% EtOAc). **^{1}H NMR** (400 MHz, $CDCl_3$): δ ppm 7.37 – 7.25 (5H, m, Ar*H*), 7.25 – 7.14 (5H, m, Ar*H*), 7.01 (2H, d, *J* = 8.4, Ar*H*), 6.81 (2H, d, *J* = 8.4, Ar*H*), 4.96 (2H, s, OCH_2Ar), 3.71 (2H, s, NHCH_2Ar), 2.59 (2H, t, *J* = 7.2 Hz, 1-$H_{A,B}$), 2.53 (2H, t, *J* = 7.2, 3-$H_{A,B}$), 2.01 (1H, s, N*H*), 1.75 (2H, quint, *J* = 7.2, 2-$H_{A,B}$). **^{13}C NMR** (100 MHz, $CDCl_3$): δ ppm 157.1 (*Ar*), 139.9 (*Ar*), 137.3 (*Ar*), 134.5 (*Ar*), 129.3 (*Ar*H), 128.6 (*Ar*H), 128.5 (*Ar*H), 128.3 (*Ar*H), 127.9 (*Ar*H), 127.5 (*Ar*H), 127.1 (*Ar*H), 114.8 (*Ar*H), 70.1 (O*C*H$_2$Ar), 53.9 (NH*C*H$_2$Ar), 48.7 (1-*C*H$_2$), 32.7 (3-*C*H$_2$), 31.7 (2-*C*H$_2$). **IR** ν_{max} (neat)/cm^{-1}: 3407 (N-H), 3061, 3030, 2928, 2859, 2804 (C-H), 1609, 1509, 1452 (C=C), 1236 (C-O). **HRMS** (ESI): $C_{23}H_{26}NO$ [M + H$^+$]: calculated 332.2009, found 332.2006.

4-(3-Aminopropyl)phenol S6

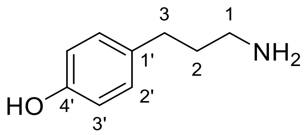

To $Pd(OH)_2/C$ (3.03 g, 20% w/w) under an inert atmosphere in a round bottomed flask, 10 mL of MeOH was gently added. A solution of *N*-benzyl-3-[4-(benzyloxy)phenyl]propan-1-amine **S5** (15.1 g, 45.7mmol, 1.0 eq.) in MeOH (40 mL) was then added followed by 76.6 mL of AcOH. The reaction mixture was diluted (by adding 260 mL of MeOH) and transferred to the steel vessel of a Parr hydrogenator. The mixture was degassed and nitrogen gas was bubbled into it. This procedure was repeated twice. Similarly, the reaction mixture was degassed and hydrogen gas was bubbled into it. This procedure was also repeated twice. From the control unit, the mixture was made to stir at 1000 rpm. The pressure and temperature were set at 15 bar and 40 °C respectively. After 24 h, the reaction mixture was filtered through a plug of celite with MeOH (400 mL) and evaporated *in vacuo*. Flash chromatography on silica gel eluting with 10% MeOH in DCM followed by 10% saturated NH_3/MeOH in DCM afforded a sticky brown oil (6.90 g, 45.6 mmol, quant.); $\mathbf{R_f}$ = 0.19 (10% saturated NH_3/MeOH in DCM). The NMR data aligns with the literature.[3] ^{1}**HNMR** (500 MHz, MeOD): δ ppm 6.90 (2H, d, *J* = 8.5, Ar*H*), 6.58 (2H, d, 8.5, Ar*H*), 2.53 (2H, t, *J* = 7.5, 1-$H_{A,B}$), 2.44 (2H, t, *J* = 7.5, 3-$H_{A,B}$), 1.63 (2H, quint, *J* = 7.5, 2-$H_{A,B}$). ^{13}**CNMR** (125 MHz, MeOD): δ ppm 155.3 (*Ar*), 132.5 (*Ar*), 128.9 (*Ar*H), 114.8 (*Ar*H), 40.6 (1-*C*), 34.3 (2-*C*), 31.9 (3-*C*). **IR** ν_{max} (neat)/cm^{-1}: 3348 (N-H), 3009, 2928, 2854, 2674, 2586 (C-H), 1592, 1513, 1452 (C=C), 1244 (C-O).

1-(3-(4-Hydroxyphenyl)propyl)-3-(4-methoxyphenyl)urea 1b

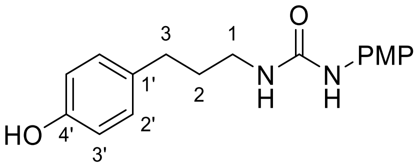

4-Methoxyphenyl isocyanate (0.30 mL, 2.30 mmol, 1.01 eq.) was added to a solution of 4-(3-aminopropyl)phenol **S6** (345 mg, 2.28 mmol, 1.0 eq.) in anhydrous THF and the mixture was refluxed for 1 h. The reaction mixture was evaporated *in vacuo.* Flash chromatography with 50 – 100% EtOAc in hexane afforded the product as a brown oil (497 mg, 1.66 mmol, 73%

yield); R_f = 0.41 (70% EtOAc in hexane). **^{1}H NMR** (500 MHz, MeOD) δ ppm 7.12 (2H, d, *J* = 9.0, Ar*H*), 6.91 (2H, d, *J* = 8.5, Ar*H*), 6.73 (2H, d, *J* = 9.0, Ar*H*), 6.59 (2H, d, *J* = 8.5, Ar*H*), 3.63 (3H, s, ArOC*H₃*), 3.07 (2H, t, *J* = 7.0, 1-$H_{A,B}$), 2.45 (2H, t, *J* = 7.5, 3-$H_{A,B}$), 1.67 (2H, quint, *J* = 7.5, 2-$H_{A,B}$). **^{13}C NMR** (125 MHz, MeOD): δ ppm 158.9 (Urea *C*=O), 157.1 (*Ar*), 156.5 (*Ar*), 133.9 (*Ar*), 133.7 (*Ar*), 130.3 (*Ar*H), 122.8 (*Ar*H), 116.1 (*Ar*H), 115.1 (*Ar*H), 55.9 (ArOCH_3), 40.4 (1-*C*), 33.4 (3-*C*), 33.3 (2-*C*). **IR** v_{max} (neat)/cm^{-1}: 3308 (O-H, N-H), 3053, 2935, 2837 (C-H), 1647 (C=O), 1554, 1509, 1441 (C=C), 1228 (C-O). **HRMS** (ESI): $C_{17}H_{21}N_2O_3$ [M + H$^+$]: calculated 301.1547, found 301.1543.

***N*-((3-(4-Hydroxyphenyl)propyl)carbamoyl)-4-methylbenzenesulfonamide 1c**

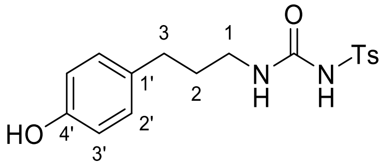

4-Toluenesulfonyl isocyanate (0.35 mL, 2.28 mmol, 1.01 eq.) was added to a solution of 4-(3-aminopropyl)phenol **S6** (341 mg, 2.26 mmol, 1.0 eq.) in anhydrous THF and the mixture was refluxed for 1 h. The reaction mixture was evaporated *in vacuo.* Flash chromatography with 30 – 90% EtOAc in hexane afforded the product as a brown oil (595 mg, 1.72 mmol, 76% yield); R_f = 0.48 (70% EtOAc in hexane). **^{1}H NMR** (300 MHz, MeOD): δ ppm 7.74 (2H, d, *J* = 8.4, Ar*H*), 7.25 (2H, d, *J* = 8.1, Ar*H*), 6.78 (2H, d, *J* = 8.7, Ar*H*), 6.56 (2H, d, *J* = 8.4, Ar*H*), 2.97 (2H, t, *J* = 6.9, 1-$H_{A,B}$), 2.33 - 2.24 (5H, m, 3-$H_{A,B}$; ArCH_3), 1.54 (2H, quint, *J* = 6.9, 2-$H_{A,B}$). **^{13}C NMR** (75 MHz, MeOD): δ ppm 156.4 (Urea *C*=O), 153.9 (*Ar*), 145.7 (*Ar*), 138.7 (*Ar*), 133.5 (*Ar*), 130.6 (*Ar*H), 130.2 (*Ar*H), 128.5 (*Ar*H), 116.1 (*Ar*H), 40.3 (1-*C*), 32.9 (3-*C*), 32.7 (2-*C*), 21.5 (ArCH_3). **IR** v_{max} (neat)/cm^{-1}: 3352 (O-H, N-H), 2929, 2860 (C-H), 1668 (C=O), 1539,1514, 1444 (C=C), 1157 (C-O). **HRMS** (ESI): $C_{17}H_{21}N_2O_4S$ [M + H$^+$]: calculated 349.1217, found 349.1217

Dearomatising Cyclisations:

(6a*R, 10a*S**)-6-Isopropyl-2,3,6a,7-tetrahydro-1*H,*5*H*-benzo[*d*]pyrrolo[1,2-*c*]imidazole-5,8(6*H*)-dione 2a**

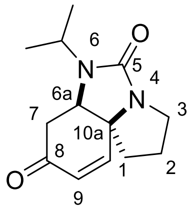

A solution of PIFA (1.21g, 2.81 mmol, 1.1 eq.) dissolved in DCM/HFIP (7ml, 50:50) was added to a solution of 1-[3-(4-hydroxyphenyl)propyl]-3-isopropylurea **1a** (603 mg, 2.55 mmol, 1.0 eq.) in 7 mL of the same solvent system at 0 °C and the mixture was stirred for 2 h after which it was allowed to warm up to room temperature for 20 min. The reaction mixture was washed with 20 mL of 10% Na_2CO_3 solution and the aqueous layer was extracted with DCM (4 × 25 mL) and evaporated *in vacuo*. Flash chromatography on silica gel eluting with 1% MeOH in DCM gave the product as a colourless oil (222 mg, 0.95 mmol, 37%); $\mathbf{R_f}$ **=** 0.36 (100% EtOAc). **^{1}H NMR** (300 MHz, MeOD): δ ppm 6.58 (1H, d, *J* = 10.2, 9-*H*), 5.96 (1H, d, *J* =10.2, 10-*H*), 4.04 (1H, dd, *J* = 6.9, 5.3, 6a-*H*), 3.82 (1H, hept, *J* = 6.9, isopropyl C*H*), 3.65 (1H, ddd, *J* = 15.6, 7.8, 3.9, 3-H_A), 3.08 – 2.96 (1H, m, 3-H_B), 2.81 (1H, dd, *J* = 16.1, 5.2, 7-H_A), 2.61 (1H, dd, *J* = 16.1, 6.9, 7-H_B), 2.05 - 1.90 (1H, m, 2-H_A), 1.90 – 1.82 (1H, m, 1-H_A), 1.82 – 1.72 (2H, m, 1-H_B, 2-H_B), 1.14 (3H, d, *J* = 6.9, isopropyl CH_{3A}), 1.11 (3H, d, *J* = 6.9, isopropyl CH_{3B}). **^{13}C NMR** (75 MHz, MeOD): δ ppm 198.2 (8-*C*), 164.2 (5-*C*), 147.8 (9-*C*), 128.2 (10-*C*), 64.7 (10a-*C*), 59.1 (6a-*C*), 46.7 (3-*C*), 46.0 (isopropyl *C*H), 41.9 (7-*C*), 35.8 (1-*C*), 25.5 (2-*C*), 21.4 (isopropyl CH_{3B}), 19.5 (isopropyl CH_{3A}). **IR** ν_{max} (neat)/cm^{-1}: 2966, 2938, 2876 (C-H), 1681 (C=O), 1456 (C=C). **HRMS** (ESI): $C_{13}H_{19}N_2O_2$ [M + H$^+$]: calculated 235.1441, found 235.1437.

(6a*R,10a*S**)-6-(4-Methoxyphenyl)-2,3,6a,7-tetrahydro-1*H*,5*H*-benzo[*d*]pyrrolo[1,2-*c*]imidazole-5,8(6*H*)-dione 2b**

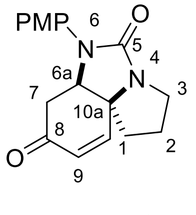

A solution of PIFA (361 mg, 0.84 mmol, 1.01 eq.) dissolved in DCM/HFIP (0.62 mL, 50:50) was added to a solution of 1-(3-(4-hydroxyphenyl)propyl)-3-(4-methoxyphenyl)urea **1b** (250 mg, 0.83 mmol, 1.0 eq.) in 2.7 mL of the same solvent system at 0 °C and the mixture was stirred for 2 h after which it was allowed to warm up to room temperature in 20 min, and then evaporated *in vacuo*. Flash

chromatography on silica gel eluting with 1% MeOH in DCM gave a white solid as product (104 mg, 0.35 mmol, 42%); **R_f =** 0.35 (100% EtOAc). **^{1}H NMR** (300 MHz, $CDCl_3$): δ ppm 7.13 (2H, d, *J* = 9.0, Ar*H*), 6.91 (2H, d, *J* = 9.0, Ar*H*), 6.56 (1H, d, *J* = 10.2, 9-*H*), 6.13 (1H, d, *J* = 10.3, 10-*H*), 4.36 (1H, t, *J* = 5.1, 6a-*H*), 3.99 – 3.88 (1H, m, 3-H_A), 3.80 (3H, s, ArOCH_3), 3.31 - 3.19 (1H, m, 3-H_B), 2.74 (1H, dd, *J* = 16.7, 5.1, 7-H_A), 2.66 (1H, dd, *J* = 16.7, 5.3, 7-H_B), 2.30 – 1.98 (4H, m, 1-$H_{A,B}$; 2-$H_{A,B}$). **^{13}C NMR** (75 MHz, $CDCl_3$): δ ppm 195.2 (8-*C*), 160.7 (5-*C*), 157.7 (*Ar*), 146.2 (9-*C*), 129.8 (*Ar*), 127.7 (10-*C*), 125.8 (*Ar*H), 114.7 (*Ar*H), 62.8 (10a-*C*), 61.7 (6a-*C*), 55.5 (ArO*C*H$_3$), 46.0 (3-*C*), 38.2 (7-*C*) 35.6 (1-*C*), 25.7 (2-*C*). **IR** ν_{max} (neat)/cm^{-1}: 2957, 2900, 2834 (C-H), 1686 (C=O), 1582, 1510, 1443, 1390 (C=C). **HRMS** (ESI): $C_{17}H_{19}N_2O_3$ [M + H$^+$]: calculated 299.1390, found 299.1390.

(6a*R,10a*S**)-6-(4-Toluenesulfonyl)-2,3,6a,7-tetrahydro-1*H*,5*H*-benzo[*d*]pyrrolo[1,2-*c*]imidazole-5,8(6*H*)-dione 2c**

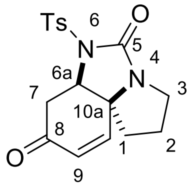

A solution of PIFA (645 mg, 1.50 mmol, 1.01 eq.) dissolved in DCM/HFIP (1.1 mL, 50:50) was added to a solution of *N*-((3-(4-hydroxyphenyl)propyl)carbamoyl)-4-methylbenzenesulfonamide **1c** (518 mg, 1.49 mmol, 1.0 eq.) in 4.9 mL of the same solvent system at 0 °C and the mixture was stirred for 2 h after which it was allowed to warm up to room temperature in 20 min, and then evaporated *in vacuo*. Flash chromatography on silica gel eluting with 40% EtOAc in hexane gave a white solid as the product (118 mg, 0.34 mmol, 23%) **R_f =** 0.57 (100% EtOAc). **^{1}H NMR** (300 MHz, $CDCl_3$): δ ppm 7.85 (2H, d, *J* = 8.3, Ar*H*), 7.26 (2H, d, *J* = 8.2, Ar*H*), 6.40 (1H, d, *J* = 10.3, 9-*H*), 6.05 (1H, d, *J* = 10.3, 10-*H*), 4.54 (1H, dd, *J* = 8.8, 5.8, 6a-*H*), 3.85 – 3.70 (1H, m, 3-H_A), 3.12 (1H, dd, *J* = 15.9, 5.7, 7-H_A), 3.05 (1H, ddd, *J* = 12.9, 5.7, 1.2, 3-H_B), 2.86 (1H, dd, *J* = 15.9, 8.8, 7-H_B), 2.36 (3H, s, ArCH_3), 2.04 – 1.81 (3H, m, 1-H_A, 2-$H_{A,B}$), 1.63 (1H, dt, *J* = 12.8, 9.5, 1-H_B). **^{13}C NMR** (75 MHz, $CDCl_3$): δ ppm 194.5 (8-*C*), 156.3 (5-*C*), 145.3 (*Ar*), 142.8 (9-*C*), 135.5 (*Ar*), 129.7 (*Ar*H), 129.0 (10-*C*), 128.3 (*Ar*H), 63.2 (10a-*C*), 58.3 (6a-*C*), 44.7 (3-*C*), 41.4 (7-*C*) 35.1 (1-*C*), 23.6 (2-*C*), 21.7 (Ar*C*H$_3$). **IR** ν_{max} (neat)/cm^{-1}: 2958, 2930 (C-H), 1736, 1685 (C=O), 1597, 1494, 1458, 1369 (C=C). **HRMS** (ESI): $C_{17}H_{19}N_2O_4S$ [M + H$^+$]: calculated 347.1060, found 347.1060.

(6a*R,8*S**,10*S**)-8-Hydroxy-6-isopropyl-2,3,6,6a,7,8-hexahydro-1*H*,5*H*-benzo[*d*]pyrrolo[1,2-*c*]imidazol-5-one 3a**

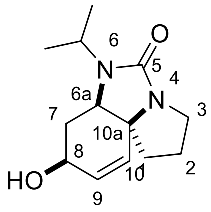

(Assignment of stereochemistry: X-ray crystal structure - CCDC deposition number: 2174963[4])

A mixture of enone **2a** (186 mg, 0.79 mmol, 1.0 eq.) and $CeCl_3.7H_2O$ (354 mg, 0.95 mmol, 1.2 eq.) in 4 mL of HPLC grade methanol was allowed to stir for 30 min at -78 °C after which $NaBH_4$ (35.9 mg, 0.95 mmol, 1.2 eq.) was added and the mixture was further stirred at the same temperature for 40 min. The reaction mixture was then allowed to warm up to room temperature for 1 h. It was extracted with EtOAc (5 × 20mL), dried over $MgSO_4$ and evaporated *in vacuo*. Silica gel chromatography eluting with 70 – 90% EtOAc in hexane afforded the compound as a white solid (173 mg, 0.73 mmol, 92% yield); $\mathbf{R_f}$ = 0.42 (100% EtOAc). ^{1}**H NMR** (300 MHz, MeOD): δ ppm 5.87 (1H, dt, *J* = 10.2, 1.3, 10-*H*), 5.67 (1H, dd, *J* = 10.2, 2.2, 9-*H*), 4.21 (1H, ddd, *J* = 10.8, 4.5, 2.4, 8-*H*), 3.96 (1H, hept, *J* = 6.9, isopropyl C*H*), 3.80 (1H, dd, *J* = 12.0, 5.1, 6a-*H*), 3.71 (1H, ddd, *J* = 12.3, 5.7, 3.0, 3-H_A), 3.04 (1H, ddd, *J* = 12.2, 9.2, 5.7, 3-H_B), 2.44 (1H, dtd, *J* = 11.2, 4.8, 1.4, 7-H_A), 2.05 – 1.79 (2H, m, 2-$H_{A,B}$), 1.69 (1H, ddd, *J* = 12.3, 7.8, 2.6, 1-H_A), 1.61 – 1.38 (2H, m, 1-H_B, 7-H_B), 1.28 (3H, d, *J* = 6.9, isopropyl CH_{3A}), 1.26 (3H, d, *J* = 6.6, isopropyl CH_{3B}). ^{13}**C NMR** (75 MHz, MeOD): δ ppm 164.5 (5-*C*), 134.7 (10-*C*), 127.0 (9-*C*), 65.8 (10a-*C*), 65.5 (8-*C*), 56.0 (6-*C*), 46.1 (isopropyl *C*H), 45.3 (3-*C*), 39.8 (7-*C*), 36.3 (1-*C*), 23.6 (2-*C*), 22.4 (isopropyl CH_{3A}), 19.7 (isopropyl CH_{3B}). **IR** ν_{max} (neat)/cm^{-1}: 3369 (O-H), 2971, 2937 (C-H), 1666 (C=O), 1416 (C=C), 1223 (C-O). **HRMS** (ESI): $C_{13}H_{21}N_2O_2$ [M + H$^+$]: calculated 237.1598, found 237.1598.

(6a*R,10a*S**)-6-Isopropylhexahydro-1*H*,5*H*-benzo[*d*]pyrrolo[1,2-*c*]imidazole-5,8(6*H*)-dione 4a**

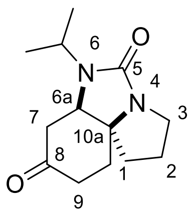

To a solution of the enone **2a** (197 mg, 0.84 mmol, 1.0 eq.) and tris(triphenyl)rhodium(I) chloride (15.6 mg, 16.9 µmol, 2.0 mol%) in 5 mL THF, 6 mL of TES was added. The mixture was stirred at room temperature for 24 h. 0.05 mL of 1M HCl was then added and the mixture was stirred at room temperature for 1 h and evaporated *in vacuo*. Flash chromatography eluting with 50 – 100% EtOAc in hexane afforded the product **4a** (167 mg, 0.71 mmol, 85%); $\mathbf{R_f}$ = 0.43 (100% EtOAc). ^{1}**H NMR** (300 MHz, MeOD) δ ppm 4.03 (1H, dd, *J* = 5.1, 3.2, 6a-*H*), 3.71 (1H, hept, *J* = 6.9, isopropyl C*H*), 3.53 (1H, ddd, *J* = 12.0, 6.0, 3.3, 3-H_A), 2.92 (1H, ddd, *J* = 12.0, 5.7, 3.6, 3-H_B), 2.83 (1H, dd, *J* = 15.5, 5.3, 7-H_A), 2.50 (1H, dd, *J* = 15.5, 3.1, 7-H_B), 2.31 – 2.22 (2H, m, 1-H_A, 9-H_A), 2.03 (1H, ddd, *J* = 14.5, 10.1, 6.5, 1-

H_B), 1.95 – 1.78 (4H, m, 9-H_B, 10-H_A, 2-$H_{A,B}$), 1.65 – 1.52 (1H, m, 10-H_B), 1.14 (3H, d, *J* = 6.9, isopropyl CH_{3A}), 1.12 (3H, d, *J* = 6.9, isopropyl CH_{3B}). **^{13}C NMR** (75 MHz, MeOD) δ ppm 213.1 (8-*C*), 164.9 (5-*C*), 66.6 (10a-*C*), 60.0 (6a-*C*), 46.3 (isopropyl *C*H), 45.3 (3-*C*), 43.3 (7-*C*), 38.0 (10-*C*), 36.1 (1-*C*), 29.5 (9-*C*), 24.7 (2-*C*), 21.4 (isopropyl CH_{3B}), 19.5 (isopropyl CH_{3A}). **IR** ν_{max} (neat)/cm^{-1}: 2967 (C-H), 1682 (C=O). **HRMS** (ESI): $C_{13}H_{21}N_2O_2$ [M + H$^+$]: calculated 237.1598, found 237.1595.

(6a*R,10a*S**)-6-(4-Methoxyphenyl)hexahydro-1*H*,5*H*-benzo[*d*]pyrrolo[1,2-*c*]imidazole-5,8(6*H*)-dione 4b**

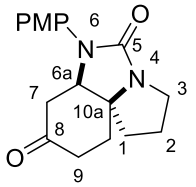

To a mixture of $Pd(OH)_2$/C (20.8 mg, 20% w/w) and enone **2b** (104 mg, 0.35 mmol, 1.0 eq.) under an atmosphere of nitrogen, 10 mL of HPLC grade EtOAc was added gently. The reaction mixture was degassed and hydrogen gas was bubbled through it with the aid of a balloon, and this procedure was repeated twice. The mixture was then allowed to stir under a balloon of hydrogen for 23 h at room temperature. The reaction mixture was filtered through a plug of Celite washing with 100 mL EtOAc. The filtrate was evaporated *in vacuo*. Flash chromatography eluting with 2 - 4% MeOH in DCM afforded the product **4b** as a brown solid (72 mg, 0.24 mmol, 69%); **R_f** = 0.41 (100% EtOAc). **^{1}H NMR** (500 MHz, $CDCl_3$): δ ppm 7.04 (2H, d, *J* = 9.0, Ar*H*), 6.81 (2H, d, *J* = 9.0, Ar*H*), 4.35 (1H, t, *J* = 4.0, 6a-*H*), 3.76 (1H, ddd, *J* = 11.5, 5.5, 3.5, 3-H_A), 3.71 (3H, s, ArOCH_3), 3.07 (1H, ddd, *J* = 11.5, 6.0, 3.5, 3-H_B), 2.60 – 2.54 (2H, m, 7-$H_{A,B}$), 2.54 -2.49 (1H, m, 1-H_A), 2.31 (1H, dt, *J* = 19.0, 3.9, 1-H_B), 2.03 – 1.89 (5H, m, 2-$H_{A,B}$; 9-$H_{A,B}$; 10-H_A), 1.80 (1H, ddd, *J* = 16.0, 9.5, 3.5, 10-H_B). **^{13}C NMR** (125 MHz, $CDCl_3$): δ ppm 208.2 (8-*C*), 160.0 (5-*C*), 156.3 (*Ar*), 128.9 (*Ar*), 124.3 (*Ar*H), 113.6 (*Ar*H), 63.2 (10a-*C*), 60.0 (6a-*C*), 54.5 (ArO*C*H$_3$), 43.5 (3-*C*), 39.4 (7-*C*) 37.4 (10-*C*), 34.2 (1-*C*), 28.7 (9-*C*), 23.4 (2-*C*). **IR** ν_{max} (neat)/cm^{-1}: 2998, 2953, 2891 (C-H), 1710 (C=O), 1617, 1586, 1516, 1406 (C=C). **HRMS** (ESI): $C_{17}H_{21}N_2O_3$ [M + H$^+$]: calculated 301.1547, found 301.1544.

(6a*R,10a*S**)-6-(4-Toluenesulfonyl)hexahydro-1*H*,5*H*-benzo[*d*]pyrrolo[1,2-*c*]imidazole-5,8(6*H*)-dione 4c**

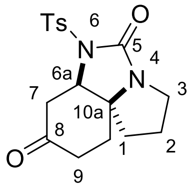

To a mixture of $Pd(OH)_2/C$ (27.0 mg, 20% w/w) and enone **2c** (133 mg, 0.38 mmol, 1.0 eq.) under an atmosphere of nitrogen, 10 mL of HPLC grade EtOAc was added gently. The reaction mixture was degassed and hydrogen gas was bubbled through it with the aid of a balloon, and this procedure was repeated twice. The mixture was then allowed to stir under a balloon of hydrogen for 27 h at room temperature. The reaction mixture was filtered through a plug of Celite washing with 100 mL EtOAc. The filtrate was evaporated *in vacuo*. Flash chromatography eluting with 50 - 90% EtOAc in hexane afforded the product **4c** as a white solid (111 mg, 0.32 mmol, 83%); **R_f =** 0.61 (100% EtOAc). **^{1}H NMR** (400 MHz, $CDCl_3$): δ ppm 7.83 (2H, d, *J* = 8.4, Ar*H*), 7.32 (2H, d, *J* = 8.8, Ar*H*), 4.56 (1H, dd, *J* = 5.2, 3.8, 6a-*H*), 3.66 (1H, ddd, *J* = 12.0, 5.6, 3.2, 3-H_A), 3.13 (1H, dd, *J* = 16.3, 3.7, 7-H_A), 2.94 (1H, ddd, *J* = 12.1, 9.2, 4.9, 3-H_B), 2.84 (1H, dd, *J* = 16.3, 5.3, 7-H_B), 2.35 (3H, s, ArCH_3), 2.34 - 2.18 (2H, m, 1-$H_{A,B}$), 2.03 – 1.79 (5H, m, 2-$H_{A,B}$; 9-$H_{A,B}$; 10-H_A), 1.59-1.50 (1H, m, 10-H_B). **^{13}C NMR** (100 MHz, $CDCl_3$): δ ppm 208.1 (8-*C*), 156.6 (5-*C*), 145.0 (*Ar*), 135.4 (*Ar*), 129.6 (*Ar*H), 128.4 (*Ar*H), 65.1 (10a-*C*), 59.4 (6a-*C*), 43.6 (3-*C*), 42.9 (7-*C*), 37.1 (10-*C*), 34.8 (1-*C*), 28.4 (9-*C*), 23.5 (2-*C*), 21.7 (ArCH$_3$). **IR** ν_{max} (neat)/cm^{-1}: 2959 (C-H), 1718 (C=O), 1596, 1494, 1455, 1352 (C=C). **HRMS** (ESI): $C_{17}H_{21}N_2O_4S$ [M + H$^+$]: calculated 349.1217, found 349.1217.

(6a*R,8*R**,10a*S**)-8-Hydoxy-6-isopropyl-8-phenyloctahydro-1*H*,5*H*-benzo[*d*]pyrrolo[1,2-*c*]imidazol-5-one 5a**

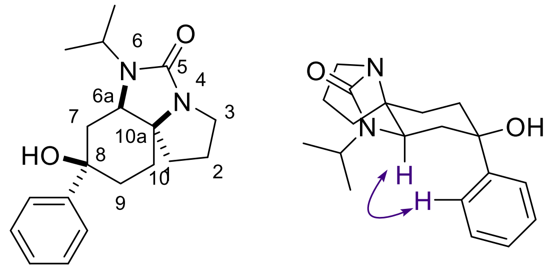

(Assignment of stereochemistry: NOESY)

To a solution of the ketone **4a** (19.0 mg, 0.08 mmol, 1.0 eq.) in 4 mL THF at -78 °C, 1.9 M PhLi in diethyl ether (0.17 mL, 0.32 mmol, 4.0 eq.) was added and stirred at the same temperature for 4.5 h after which it was left to warm up to room temperature overnight. 0.10 mL of saturated aqueous ammonia and 0.9 mL of saturated aqueous ammonium chloride were added and allowed to stir for 20 min. The reaction mixture was diluted with 100 mL EtOAc, dried over Na_2SO_4 and evaporated *in vacuo*. Flash chromatography with 1 - 3% MeOH in DCM afforded the product **5a** as a single diastereomer (15.0 mg, 0.05 mmol, 59% yield). **R_f** = 0.36 (5% MeOH in DCM). **^{1}H NMR** (500 MHz, $CDCl_3$): δ ppm 7.43 (2H, dt, *J* = 8.0, 2.0, Ar*H*), 7.33 (2H, tt, *J* = 7.5, 2.0, Ar*H*), 7.24 (1H, tt, *J* = 7.5, 2.0, Ar*H*), 3.96 (1H, hept, *J* = 6.9, isopropyl C*H*), 3.75 (1H, ddd, *J* = 12.2, 9.1, 5.7, 3-H_A), 3.55 (1H, dd, *J* = 8.9, 5.6, 6a-*H*), 2.92 (1H, ddd, *J* = 12.2, 9.2, 5.6, 3-H_B), 2.55 (1H, ddd, *J* = 14.0, 5.5, 1.3, 7-H_A), 2.26 (1H,

s, 8-CO*H*), 2.11 – 2.03 (1H, m, 9-H_A), 1.99 – 1.91 (2H, m, 7-H_B, 9-H_B), 1.81 (1H, ddd, *J* = 14.5, 6.5, 3.5, 10-H_A), 1.79 – 1.67 (2H, m, 2-$H_{A,B}$), 1.46 (1H, ddd, *J* = 12.0, 8.0, 3.0, 1-H_A), 1.35 (1H, dt, *J* = 12.0, 10.0, 1-H_B), 1.28 (1H, ddd, *J* = 14.7, 9.2, 3.8, 10-H_B), 1.22 (3H, d, *J* = 6.8, isopropyl CH_{3A}), 1.18 (3H, d, *J* = 6.9, isopropyl CH_{3B}). **^{13}C NMR** (125 MHz, $CDCl_3$): δ ppm 163.4 (5-*C*), 145.7 (*Ar*), 128.8 (*Ar*H), 127.6 (*Ar*H), 125.2 (*Ar*H), 72.2 (8-*C*), 65.4 (10a-*C*), 55.7 (6a-*C*), 44.7 (isopropyl *C*H), 44.0 (3-*C*), 42.7 (7-*C*), 35.2 (1-*C*), 35.1 (9-*C*), 28.7 (10-*C*), 23.2 (2-*C*), 22.0 (isopropyl *C*H_{3A}), 19.9 (isopropyl *C*H_{3B}). **IR** ν_{max} (neat)/cm^{-1}: 3388 (O-H), 2964 (C-H), 1673 (C=O), 1447 (C=C), 1052 (C-O). **HRMS** (ESI): $C_{19}H_{27}N_2O_2$ [M + H$^+$]: calculated 315.2067, found 315.2061.

(6a*R,8*R**,10a*S**)-8-Hydroxy-6-isopropyloctahydro-1*H*,5*H*-benzo[*d*]pyrrolo[1,2-*c*]imidazol-5-one 6a**

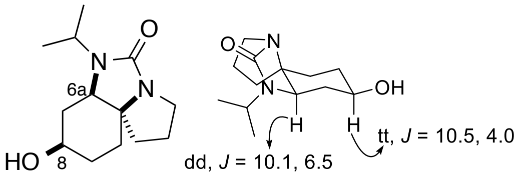

*(Assignment of stereochemistry: J value analysis; X-ray crystal structure of derivative **20**)*

Method 1

To a solution of the ketone **4a** (81.0 mg, 0.34 mmol, 1.0 eq.) in anhydrous THF (4 mL), 0.13 mL of $LiAlH_4$ (4 M in THF) was added at room temperature and the mixture was allowed to stir for 30 min. The reaction mixture was quenched with 0.1 mL 10% KOH and 0.1 mL H_2O, diluted with 100 mL EtOAc, dried over Na_2SO_4 and evaporated *in vacuo*. Flash chromatography with 1 – 4% MeOH in DCM afforded the product as a diastereomeric mixture in the ratio of 93:7 (65.0 mg, 0.27 mmol, 79% yield). **R_f** = 0.34 (5% MeOH in DCM). **^{1}H NMR** (Major diastereomer, 500 MHz, $CDCl_3$): δ ppm 3.99 (1H, hept, *J* = 7.0, isopropyl C*H*), 3.77 (1H, ddd, *J* = 12.4, 9.2, 5.2, 3-H_A), 3.56 (1H, tt, *J* = 10.5, 4.0, 8-*H*), 3.44 (1H, dd, *J* = 10.1, 6.5, 6a-*H*), 2.85 (1H, ddd, *J* =12.0, 6.0, 3.0, 3-H_B), 2.33 (1H, dddd, *J* = 12.7, 6.5, 4.3, 2.2, 7-H_A), 2.10 (1H, s, 8-CHO*H*), 1.85 (1H, dt, *J* = 14.5, 4.0, 10-H_A), 1.80 – 1.62 (3H, m, 2-$H_{A,B}$; 9-H_A), 1.50 – 1.34 (4H, m, 1-$H_{A,B}$; 7-H_B; 9-H_B), 1.31 – 1.23 (1H, m, 10-H_B), 1.15 (3H, d, *J* = 7.0, isopropyl CH_{3A}), 1.11 (3H, d, *J* = 6.5, isopropyl CH_{3B}). Signals for minor isomer visible at: 3.72 (0.08H, ddd, *J* = 12.0, 6.0, 3.0, 3-H_A), 3.63 (0.07H, dd, *J* = 8.0, 6.0, 7-H_A). **^{13}C NMR** (Major diastereomer, 125 MHz, $CDCl_3$): δ ppm 162.6 (5-*C*), 66.2 (8-*C*), 64.5 (10a-*C*), 53.5 (6a-*C*), 43.2 (isopropyl *C*H), 43.0 (3-*C*), 40.8 (7-*C*), 33.6 (1-*C*), 29.6 (10-*C*), 28.1 (9-*C*), 21.9 (2-*C*), 21.4 (isopropyl *C*H_{3A}), 18.7 (isopropyl *C*H_{3B}). Signals for minor isomer visible at: 64.7, 63.3, 52.3, 36.4, 34.3, 27.2, 24.6, 22.1, 21.2, 18.6. **IR** ν_{max} (neat)/cm^{-1}: 3399 (O-H), 2937 (C-H), 1666 (C=O), 1056 (C-O). **HRMS** (ESI): $C_{13}H_{23}N_2O_2$ [M + H$^+$]: calculated 239.1754, found 239.1750.

Method 2

A mixture of the ketone **4a** (24.0 mg, 0.10 mmol, 1.0 eq.) and $CeCl_3.7H_2O$ (45.5 mg, 0.12 mmol, 1.2 eq.) in 3 mL of HPLC grade MeOH and it was allowed to stir at –78 °C for 30 min. $NaBH_4$ (4.62 mg, 0.12 mmol, 1.2 eq.) was then added and the mixture was left to warm up to room temperature for another 30 min. The reaction mixture was evaporated *in vacuo*. It was then taken up in 1 mL H_2O, diluted with 50 mL EtOAc, dried over Na_2SO_4 and evaporated *in vacuo*. Flash chromatography with 1 – 4% MeOH in DCM afforded the product **6a** as a single diastereomer (22.0 mg, 0.09 mmol, 92% yield) that is identical to the major diastereomer obtained from **Method 1**.

(6a*R,8*R**,10a*S**)-8-Hydroxy-6-(4-methoxyphenyl)octahydro-1*H*,5*H*-benzo[*d*]pyrrolo[1,2-*c*]imidazol-5-one 6b**

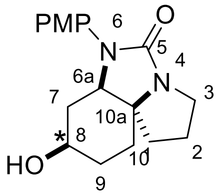

Major diastereomer, *dr* 85:15

(Assignment of stereochemistry: X-ray structure - CCDC deposition number: 2174964)

A mixture of the ketone **4b** (30.0 mg, 0.10 mmol, 1.0 eq.) and $CeCl_3.7H_2O$ (44.7 mg, 0.12 mmol, 1.2 eq.) in 3 mL of HPLC grade MeOH was allowed to stir at –78 °C for 30 min. $NaBH_4$ (4.54 mg, 0.12 mmol, 1.2 eq.) was then added and the mixture was left to warm up to room temperature for another 30 min. The reaction mixture was evaporated *in vacuo*. It was then taken up in 1 mL H_2O, diluted with 50 mL EtOAc, dried over Na_2SO_4 and evaporated *in vacuo*. Flash chromatography with 1 – 3% MeOH in DCM afforded the product **6b** as an 85:15 mixture of diastereomers (29.0 mg, 0.10 mmol, 96% yield). $\mathbf{R_f}$ = 0.37 (4% MeOH in DCM). **^{1}H NMR** (Major diastereomer, 500 MHz, $CDCl_3$): δ ppm 7.27 (2H, d, *J* = 9.0, Ar*H*), 6.80 (2H, d, *J* = 9.5, Ar*H*), 3.96 (1H, dd, *J* = 9.5, 6.2, 6a-*H*), 3.85 (1H, ddd, *J* = 12.3, 9.0, 5.5, 3-H_A), 3.72 (3H, s, ArOCH_3), 3.65 (1H, qd, *J* = 9.0, 4.3, 8-*H*), 2.99 (1H, ddd, *J* =12.0, 6.0, 3.0, 3-H_B), 2.24 (1H, dddd, *J* = 12.7, 6.2, 4.5, 1.9, 7-H_A), 1.91 (1H, dt, *J* = 14.5, 4.0, 10-H_A), 1.88 – 1.62 (6H, m, 1-$H_{A,B}$; 2-$H_{A,B}$; 8-CHO*H*; 9-H_A), 1.49 (1H, tdd, *J* = 12.9, 9.4, 3.6, 9-H_B), 1.41 – 1.31 (2H, m, 7-H_B, 10-H_B). Signals for minor isomer visible at: 4.15 (0.17H, dd, *J* = 6.5, 5.5, 6a-*H*), 4.03 (0.18H, quint, *J* = 5.5, 8-*H*). **^{13}C NMR** (Major diastereomer, 125 MHz, $CDCl_3$): δ ppm 162.0 (5-*C*), 156.4 (*Ar*), 131.4 (*Ar*), 123.4 (*Ar*H), 114.4 (*Ar*H), 69.9 (8-*C*), 64.4 (10a-*C*), 58.4 (6a-*C*), 55.5 (ArOCH_3), 44.3 (3-*C*), 38.0 (7-*C*), 35.4 (1-*C*), 30.5 (9-*C*), 29.0 (10-*C*), 23.2 (2-*C*). Signals for minor isomer visible at: 64.6, 64.0, 57.7, 36.3, 33.9, 28.4, 26.6, 23.7. **IR** ν_{max} (neat)/cm^{-1}: 3398 (O-H), 2934 (C-H), 1674 (C=O), 1582, 1510.

(6a*R,8*R**,10a*S**)-8-Hydroxy-6-(4-toluenesulfonyl)octahydro-1*H*,5*H*-benzo[*d*]pyrrolo[1,2-*c*]imidazol-5-one 6c**

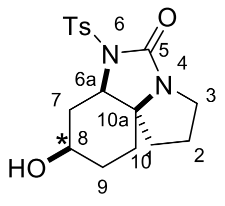

Major diastereomer, *dr* 78:22

*(Assignment of stereochemistry: by analogy with **6a/b**)*

A mixture of the ketone **4c** (18.0 mg, 0.05 mmol, 1.0 eq.) and $CeCl_3.7H_2O$ (23.2 mg, 0.06 mmol, 1.2 eq.) in 3 mL of HPLC grade MeOH was allowed to stir at –78 °C for 30 min. $NaBH_4$ (2.40 mg, 0.06 mmol, 1.2 eq.) was then added and the mixture was left to warm up to room temperature for another 30 min. The reaction mixture was evaporated *in vacuo*. It was then taken up in 1 mL H_2O, diluted with 50 mL EtOAc, dried over Na_2SO_4 and evaporated *in vacuo*. Flash chromatography with 1 – 2% MeOH in DCM afforded the product **6c** as a 78:22 mixture of diastereomers (15.0 mg, 0.04 mmol, 83% yield). $\mathbf{R_f}$ = 0.44 (4% MeOH in DCM). **^{1}H NMR** (Major diastereomer, 500 MHz, $CDCl_3$): δ ppm 7.87 (2H, d, *J* = 8.5, Ar*H*), 7.24 (2H, d, *J* = 8.5, Ar*H*), 4.17 (1H, dd, *J* = 10.0, 6.4, 6a-*H*), 3.74 – 3.61 (2H, m, 8-*H*, 3-H_A), 2.89 (1H, ddd, *J* =12.1, 9.4, 5.7, 3-H_B), 2.67 (1H, dddd, *J* = 12.8, 6.4, 4.5, 1.9, 7-H_A), 2.35 (3H, s, ArCH_3), 1.86 – 1.70 (4H, m, 2-$H_{A,B}$; 9-H_A, 10-H_A), 1.60 – 1.24 (6H, m, 1-$H_{A,B}$; 7-H_B; 8-CHO*H*; 9-H_B; 10-H_B). Signals for minor isomer visible at: 4.29 (0.29H, dd, *J* = 8.0, 5.5, 6a-*H*), 4.09 (0.29H, quint, *J* = 5.0, 8-*H*). **^{13}C NMR** (Major diastereomer, 125 MHz, $CDCl_3$): δ ppm 158.4 (5-*C*), 144.8 (*Ar*), 136.2 (*Ar*), 129.6 (*Ar*H), 128.2 (*Ar*H), 66.4 (8-*C*), 65.1 (10a-*C*), 57.7 (6a-*C*), 44.0 (3-*C*), 39.4 (7-*C*), 34.4 (1-*C*), 30.0 (9-*C*), 28.2 (10-*C*), 22.9 (2-*C*), 21.7 (ArCH_3). Signals for minor isomer visible at: 158.1, 144.7, 136.1, 129.5, 128.1, 65.4, 63.9, 57.3, 43.8, 36.7, 35.2, 27.6, 25.3, 23.0. **IR** ν_{max} (neat)/cm^{-1}: 3388 (O-H), 2922, 2852 (C-H), 1727 (C=O), 1658, 1597 (C=C), 1161 (C-O). **HRMS** (ESI): $C_{17}H_{23}N_2O_4S$ [M + H$^+$]: calculated 351.1373, found 351.1369.

(6a*R,8*R**,10a*S**)-6-Isopropyl-8-(methylamino)octahydro-1*H*,5*H*-benzo[*d*]pyrrolo[1,2-*c*]imidazol-5-one 7a**

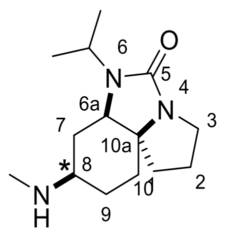

Major diastereomer, *dr* 88:12

*(Assignment of stereochemistry: NOE, X-ray of derived sulfonamide **32**)*

To a solution of the ketone **4a** (22.0 mg, 0.09 mmol, 1.0 eq.) in 6 mL THF, 33 wt% methylamine in EtOH (0.10 mL, 0.93 mmol, 10.0 eq.) and titanium isopropoxide (0.06 mL, 0.19 mmol, 2.0 eq.) were

added and the mixture was left to stir at room temperature overnight. $NaBH_4$ (5.30 mg, 0.14 mmol, 1.5 eq.) was added to the reaction mixture at -78 °C and it was stirred at the same temperature for 30 min. It was then allowed to warm up to room temperature for another 30 min. The reaction mixture was evaporated *in* vacuo. It was then taken up in 1 mL H_2O, diluted with 50 mL EtOAc, dried over $MgSO_4$ and evaporated *in vacuo*. Flash chromatography with 5% MeOH in DCM, followed by 99:9:1 of DCM/MeOH/aq. NH_3 respectively, afforded the product as a diastereomeric mixture in the ratio of 88:12 (21.0 mg, 0.08 mmol, 90% yield). **^{1}H NMR** (Major diastereomer, 500 MHz, $CDCl_3$): δ ppm 3.98 (1H, hept, *J* = 7.0, isopropyl C*H*), 3.77 (1H, ddd, *J* = 12.4, 9.2, 5.3, 3-H_A), 3.41 (1H, dd, *J* = 10.4, 6.5, 6a-*H*), 2.84 (1H, ddd, *J* = 12.4, 9.3, 5.9, 3-H_B), 2.39 – 2.31 (4H, m, 8-*H*, NHCH_3), 2.26 (1H, dddd, *J* =12.5, 6.1, 3.7, 2.2, 9-H_A), 1.88 – 1.82 (1H, m, 7-H_A), 1.80 – 1.65 (4H, m, 2-$H_{A,B}$; 10-H_A; N*H*), 1.45 – 1.34 (2H, m, 1-$H_{A,B}$), 1.29 – 1.15 (3H, m, 7-H_B, 9-H_B, 10-H_B), 1.14 (3H, d, *J* = 7.0, isopropyl CH_{3A}), 1.11 (3H, d, *J* = 7.0, isopropyl CH_{3B}). Signals for minor isomer visible at: 3.69 (0.14H, ddd, *J* = 12.1, 8.8, 6.0, 3-H_A), 3.61 (0.13H, t, 5.5, 6a-*H*). **^{13}C NMR** (Major diastereomer, 125 MHz, $CDCl_3$): δ ppm 162.6 (5-*C*), 64.9 (10a-*C*), 54.0 (6a-*C*), 53.7 (8-*C*), 43.2 (isopropyl *C*H), 43.0 (3-*C*), 38.4 (7-*C*), 33.8 (1-*C*), 32.3 (NH*C*H$_3$), 28.8 (10-*C*), 26.4 (9-*C*), 21.9 (2-*C*), 21.5 (isopropyl *C*H$_{3A}$), 18.7 (isopropyl *C*H$_{3B}$). Signals for minor isomer visible at: 162.4, 64.8, 53.8, 43.3, 34.8, 32.8, 25.3, 24.4, 22.2, 21.2, 18.5. **IR** v_{max} (neat)/cm^{-1}: 3306 (N-H), 2966, 2936, 2791 (C-H), 1686 (C=O). **HRMS** (ESI): $C_{14}H_{26}N_3O$ [M + H$^+$]: calculated 252.2070, found 252.2079.

(6a*R,11b*S**)-6-Isopropyl-2,3,6a,7-tetrahydro-1*H*-pyrrolo[1',2':3,4]imidazole[4,5-*e*]isoindole-5,8(6*H*, 10*H*)-dione 8a**

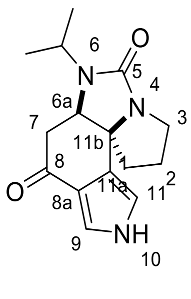

(Confirmation of structure: X-ray crystal structure – CCDC deposition number 2175091)

To a mixture of potassium *tert*-butoxide (382 mg, 3.40 mmol, 5.0 eq.) and TosMIC (132.8 mg, 0.68 mmol, 1.0 eq.), a solution of the enone **2a** (160 mg, 0.68 mmol, 1.0 eq.) in 3.5 mL anhydrous THF was added and the mixture was stirred for 17 h at room temperature. The reaction mixture was extracted with DCM (5 × 12 mL), dried over Na_2SO_4 and evaporated *in vacuo*. Flash chromatography on silica gel eluting with 50 – 100% EtOAc in hexane afforded a brown oil **8a** (32.0 mg, 0.12 mmol, 17%). **R_f** = 0.50 (100% EtOAc). **^{1}H NMR** (300 MHz, MeOD): δ ppm 7.25 (1H, d, *J* = 1.8, Ar*H*), 6.80 (1H, d, *J* = 1.8, Ar*H*), 4.15 (1H, dd, *J* = 9.5, 5.7, 6a-H), 3.92 (1H, hept, *J* = 6.9, isopropyl C*H*), 3.79 (1H, ddd, *J* = 12.0,

7.2, 3.4, 3-H_A), 3.02 (1H, ddd, *J* = 12.4, 8.8, 7.0, 3-H_B), 2.87 (1H, dd, *J* = 16.1, 5.7, 7-H_A), 2.61 (1H, dd, *J* = 16.2, 9.6, 7-H_B), 2.12 – 1.78 (4H, m, 1-$H_{A,B}$; 2-$H_{A,B}$), 1.18 (6H, d, *J* = 6.9, isopropyl C$H_{3A,B}$). ^{13}**C NMR** (75 MHz, MeOD): δ ppm 194.0 (8-*C*), 165.0 (5-*C*), 127.8 (*Ar*), 120.44 (*Ar*H), 120.36 (*Ar*), 117.6 (*Ar*H), 64.9 (11b-*C*), 59.2 (6a-*C*), 46.2 (isopropyl *C*H), 45.6 (3-*C*), 45.2 (7-*C*), 37.4 (1-*C*), 24.5 (2-*C*), 22.1 (isopropyl-CH_{3A}), 19.8 (isopropyl-CH_{3B}). **IR** v_{max} (neat)/cm^{-1}: 3271 (N-H), 2968, 2931 (C-H), 1691, 1665 (C=O), 1521, 1474, 1456 (C=C). X-ray crystal structure: See *Scheme 24* in thesis

(6a*R,8a*S**,11a*R**,11b*S**)-10-Benzyl-6-isopropyloctahydro-1*H*-pyrrolo[1',2':3,4]imidazo[4,5-*e*]isoindole-5,8(6*H*,8a*H*)-dione 9a**

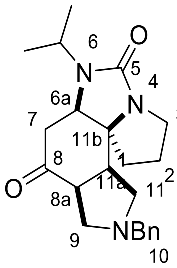

To a mixture of lithium fluoride (23.4 mg, 0.90 mmol, 1.2 eq.) and the enone **4a** (175 mg, 0.75 mmol, 1.0 eq.) in dry MeCN (1.60 mL), *N*-(methoxymethyl)-*N*-(trimethylsilylmethyl)benzylamine (0.22 mL, 0.83 mmol, 1.1 eq.) was added and the mixture was left to stir overnight. The reaction mixture was extracted with DCM (5 × 10 mL) in 10 mL of water, dried over sodium sulphate and evaporated *in vacuo*. Flash chromatography on silica gel eluting with 50 – 100% EtOAc in hexane afforded the product **9a** (106 mg, 0.29 mmol, 39%). $\mathbf{R_f}$ **=** 0.58 (10% MeOH in DCM). ^{1}**H NMR** (500 MHz, $CDCl_3$): δ ppm 7.27 - 7.11 (5H, m, Ar*H*), 3.87 (1H, hept, 7.0, isopropyl C*H*), 3.83 (1H, dd, *J* = 6.0, 2.5, 6a-H), 3.74 – 3.68 (1H, m, 3-H_A), 3.64 (1H, d, *J* = 12.5, ArCH_{2A}), 3.59 (1H, d, *J* = 13.0, ArCH_{2B}), 3.15 (1H, dd, *J* = 10.0, 7.2, 9-H_A), 2.98 – 2.92 (1H, m, 8a-*H*), 2.91 – 2.85 (2H, m, 3-H_B, 11-H_A), 2.63 (2H, dd, *J* = 5.5, 2.5, 7-$H_{A,B}$), 2.54 (1H, t, *J* = 10.2, 9-H_B), 2.41 (1H, ddd, *J* = 13.0, 8.0, 3.0, 11a-*H*), 2.30 (1H, dd, *J* = 10.3, 8.0, 11-H_B), 1.98 – 1.89 (1H, m, 1-H_A), 1.78 – 1.69 (3H, m, 1-H_B; 2-$H_{A,B}$), 1.14 (3H, d, *J* = 7.0, isopropyl CH_{3A}), 1.09 (3H, d, *J* = 7.0, isopropyl CH_{3B}). ^{13}**C NMR** (125 MHz, $CDCl_3$): δ ppm 206.7 (8-*C*), 162.5 (5-*C*), 137.7 (*Ar*), 127.6 (*Ar*H), 127.4 (*Ar*H), 126.1 (*Ar*H), 64.0 (11b-*C*), 60.8 (6a-*C*), 59.7 (Ar*C*H_2), 52.5 (11-*C*), 49.1 (9-*C*), 48.2 (8a-*C*), 48.0 (11a-*C*), 46.6 (3-*C*), 43.6 (isopropyl *C*H), 39.9 (7-*C*), 37.5 (1-*C*), 24.6 (2-*C*), 20.2 (isopropyl CH_{3B}), 18.1 (isopropyl CH_{3A}). **IR** v_{max} (neat)/cm^{-1}: 2967, 2929, 2799 (C-H), 1685 (C=O), 1453, 1413, 1379 (C=C). **HRMS** (ESI): $C_{22}H_{30}N_3O_2$ [M + H$^+$]: calculated 368.2333, found 368.2330.

(6a*R,8a*S**,11a*R**,11b*S**)-10-Benzyl-6-(4-methoxyphenyl)octahydro-1*H*-pyrrolo[1',2':3,4]imidazo[4,5-*e*]isoindole-5,8(6*H*,8a*H*)-dione 9b** and **(6a*R**,8a*R**,11a*S**,11b*S**)-10-**

benzyl-6-(4-methoxyphenyl)octahydro-1*H*-pyrrolo[1',2':3,4]imidazo[4,5-*e*]isoindole-5,8(6*H*,8a*H*)-dione 9b'

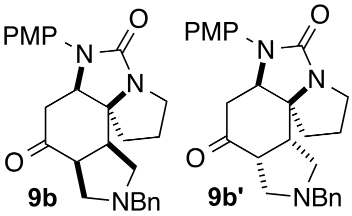

To a mixture of lithium fluoride (22.1 mg, 0.85 mmol, 1.2 eq.) and the enone **2b** (211 mg, 0.71 mmol, 1.0 eq.) in dry MeCN (5.00 mL), *N*-(methoxymethyl)-*N*-(trimethylsilylmethyl)benzylamine (0.20 mL, 0.78 mmol, 1.1 eq.) was added and the mixture was left to stir overnight. The reaction mixture was evaporated *in vacuo* and taken up in 1 mL H_2O. 100 mL EtOAc was then added and the mixture was dried over sodium sulfate, and concentrated *in vacuo*. Flash chromatography on silica gel eluting with 2% MeOH in DCM afforded the two diastereomeric pyrrolidines **9b** [**major product**, 131 mg, 0.30 mmol, 43%, $\mathbf{R_f}$ = 0.38 (4% MeOH in DCM)] and **9b'** [**minor product**, 72 mg, 0.17 mmol, 25%, $\mathbf{R_f}$ = 0.35 (4% MeOH in DCM)]. **^{1}H NMR** (**Major diastereomer 9b**, 500 MHz, $CDCl_3$): δ ppm 7.26 - 7.16 (5H, m, Ar*H*), 7.14 (2H, d, *J* = 9.0, Ar*H*), 6.82 (2H, d, *J* = 9.0, Ar*H*), 4.50 (1H, br.d, *J* = 6.0, 6a-H), 3.81 (1H, ddd, *J* = 12.3, 9.2, 5.3, 3-H_A), 3.72 (3H, s, ArOCH_3), 3.68 (1H, d, *J* = 13.0, ArCH_{2A}), 3.48 (1H, d, *J* = 13.0, ArCH_{2B}), 3.11 (1H, dd, *J* = 14.5, 6.5, 7-H_A), 3.00 (1H, ddd, *J* = 12.2, 8.7, 5.2, 3-H_B), 2.90 (1H, dd, *J* = 10.0, 1.5, 9-H_A), 2.84 – 2.81 (1H, m, 11-H_A), 2.81 – 2.77 (2H, m, 8a-*H*, 11a-*H*), 2.75 (1H, dd, 9.5, 6.0, 9-H_B), 2.65 (1H, t, 9.0, 11-H_B), 2.45 (1H, br.d, 14.5, 7-H_B), 1.97 – 1.62 (4H, m, 1-$H_{A,B}$; 2-$H_{A,B}$). **^{13}C NMR** (**Major diastereomer 9b**, 125 MHz, $CDCl_3$): δ ppm 211.1 (8-*C*), 160.9 (5-*C*), 157.3 (*Ar*), 138.6 (*Ar*), 130.0 (*Ar*), 128.6 (*Ar*H), 128.6 (*Ar*H), 127.4 (*Ar*H), 125.4 (*Ar*H), 114.7 (*Ar*H), 66.2 (11b-*C*), 60.6 (6a-*C*), 59.8 (Ar*C*H_2), 59.1 (9-*C*), 55.7 (ArO*C*H_3), 54.1 (11-*C*), 49.0 (11a-*C*), 44.4 (3-*C*), 43.4 (8a-*C*), 41.3 (7-*C*), 35.1 (1-*C*), 23.6 (2-*C*). **IR** ν_{max} (neat)/cm^{-1}: 2961, 2834, 2800 (C-H), 1697 (C=O), 1610, 1584, 1511 (C=C), 1245 (C-O). **HRMS** (ESI): $C_{26}H_{30}N_3O_3$ [M + H$^+$]: calculated 432.2282, found 432.2289. **^{1}H NMR** (**Minor diastereomer 9b'**, 500 MHz, $CDCl_3$): δ ppm 7.29 - 7.21 (5H, m, Ar*H*), 6.96 (2H, d, *J* = 9.0, Ar*H*), 6.81 (2H, d, *J* = 9.0, Ar*H*), 4.29 (1H, dd, *J* = 3.9, 2.2, 6a-*H*), 3.79 (1H, ddd, *J* = 11.5, 6.0, 2.0, 3-H_A), 3.71 (3H, s, ArOCH_3), 3.68 (1H, d, *J* = 13.0, ArCH_{2A}), 3.62 (1H, d, *J* = 13.0, ArCH_{2B}), 3.18 (1H, dd, *J* = 9.8, 7.2, 9-H_A), 3.10 (1H, ddd, *J* = 12.9, 9.9, 7.1, 8a-*H*), 3.03 (1H, dt, *J* = 11.7, 7.0, 3-H_B), 2.93 (1H, dd, *J* = 8.0, 5.5, 11-H_A), 2.64 – 2.49 (3H, m, 9-H_B, 7-$H_{A,B}$), 2.49 – 2.43 (1H, m, 11a-*H*), 2.38 (1H, dd, 10.3, 7.9, 11-H_B), 2.10 – 1.84 (4H, m, 1-$H_{A,B}$; 2-$H_{A,B}$). **^{13}C NMR** (**Minor diastereomer 9b'**, 125 MHz, $CDCl_3$): δ ppm 206.0 (8-*C*), 161.0 (5-*C*), 156.6 (*Ar*), 137.8 (*Ar*), 128.5 (*Ar*), 127.6 (*Ar*H), 127.4 (*Ar*H), 126.1 (*Ar*H), 124.7 (*Ar*H), 113.6 (*Ar*H), 64.2 (11b-*C*), 63.1 (6a-*C*), 59.7 (Ar*C*H_2), 54.5 (ArO*C*H_3), 52.4 (11-*C*), 49.0 (9-*C*), 48.1 (8a-*C*), 48.1 (11a-*C*), 46.4 (3-*C*), 37.8 (7-*C*), 37.7 (1-*C*), 24.8 (2-*C*). **IR** ν_{max} (neat)/cm^{-1}: 2960, 2928, 2836 (C-H),

1697 (C=O), 1610, 1583, 1511 (C=C), 1246 (C-O). **HRMS** (ESI): $C_{26}H_{30}N_3O_3$ [M + H$^+$]: calculated 432.2282, found 432.2292.

(6a*R,8a*S**,11a*R**,11b*S**)-10-Benzyl-6-tosyloctahydro-1*H*-pyrrolo[1',2':3,4]imidazo[4,5-*e*]isoindole-5,8(6*H*,8a*H*)-dione 9c**

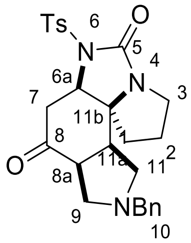

To a mixture of lithium fluoride (5.71 mg, 0.22 mmol, 1.2 eq.) and the enone **2c** (64.7 mg, 0.19 mmol, 1.0 eq.) in dry MeCN (5.00 mL), *N*-(methoxymethyl)-*N*-(trimethylsilylmethyl)benzylamine (0.05 mL, 0.78 mmol, 1.1 eq.) was added and the mixture was left to stir overnight. The reaction mixture was evaporated *in vacuo* and taken up in 1 mL H_2O. 100 mL EtOAc was then added and the mixture was dried over sodium sulfate and concentrated *in vacuo*. Flash chromatography on silica gel eluting with 0.5 - 2% MeOH in DCM afforded the pyrrolidine **9c** (22 mg, 0.05 mmol, 25%). $\mathbf{R_f}$ = 0.36 (3% MeOH in DCM). **^{1}H NMR** (500 MHz, $CDCl_3$): δ ppm 7.78 (2H, d, *J* = 8.4, Ar*H*), 7.25 - 7.14 (7H, m, Ar*H*), 4.43 (1H, dd, *J* = 4.2, 2.2, 6a-H), 3.64 (1H, ddd, *J* = 11.5, 8.0, 5.6, 3-H_A), 3.59 (1H, d, *J* = 13.0, ArCH_{2A}), 3.54 (1H, d, *J* = 13.0, ArCH_{2B}), 3.50 (1H, dd, *J* = 19.0, 2.0, 7-H_A), 3.14 (1H, dd, *J* = 10.1, 6.8, 9-H_A), 2.96 (1H, ddd, *J* = 11.5, 6.0, 4.0, 3-H_B), 2.86 (1H, dd, *J* = 8.0, 5.6, 11-H_A), 2.77 – 2.73 (1H, m, 8a-*H*), 2.73 – 2.69 (1H, m, 7-H_B), 2.45 (1H, t, 10.2, 9-H_B), 2.43 – 2.37 (1H, m, 11a-*H*), 2.35 (3H, s, ArCH_3), 2.13 (1H, dd, *J* = 10.3, 8.1, 11-H_B), 2.05 – 1.71 (4H, m, 1-$H_{A,B}$; 2-$H_{A,B}$). **^{13}C NMR** (125 MHz, $CDCl_3$): δ ppm 205.3 (8-*C*), 156.5 (5-*C*), 144.2 (*Ar*), 137.5 (*Ar*), 134.1 (*Ar*), 128.6 (*Ar*H), 127.5 (*Ar*H), 127.4 (*Ar*H), 127.4 (*Ar*H) 126.2 (*Ar*H), 65.2 (11b-*C*), 62.5 (6a-*C*), 59.5 (ArCH_2), 52.8 (11-*C*), 48.7 (9-*C*), 48.1 (8a-*C*), 47.9 (11a-*C*), 46.1 (3-*C*), 40.2 (7-*C*), 37.3 (1-*C*), 24.1 (2-*C*), 20.7 (ArCH_3). **IR** ν_{max} (neat)/cm^{-1}: 3029, 2922, 2801 (C-H), 1720 (C=O), 1596, 1513, 1458 (C=C). **HRMS** (ESI): $C_{26}H_{30}N_3O_4S$ [M + H$^+$]: calculated 480.1952, found 480.1964.

(6a*R,8a*R**,9a*S**,9b*S**)-6-Isopropylhexahydro-1*H*-cyclopropa[5,6]benzo[1,2-*d*]pyrrolo[1,2-c]imidazole-5,8(6*H*, 8a*H*)-dione 10a**

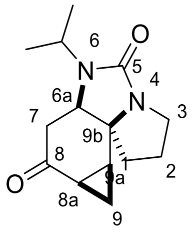

(Assignment of stereochemistry: X-ray crystal structure - CCDC deposition number: 2175090)

To a mixture of trimethylsulfoxonium chloride (75.9 mg, 0.59 mmol, 1.1 eq.) and 60% sodium hydride in mineral oil (23.6 mg, 0.59 mmol, 1.1 eq.) at 0 °C, 2 mL of anhydrous THF was added and the mixture was left to stir for 15 min. The ice bath was removed and the chalky mixture was left to stir at room temperature for 30 min. 2 mL of anhydrous THF was then used to transfer the enone **2a** (126 mg, 0.54 mmol, 1.0 eq.) to the chalky mixture and was further left to stir for 2.5 h. The reaction mixture was extracted with DCM (5 × 15 mL) in 5 mL water, dried over Na_2SO_4 and concentrated *in vacuo*. Flash chromatography on silica gel eluting with 1% MeOH in DCM afforded the compound as a white solid **10a** (95 mg, 0.38 mmol, 71%). **R_f** = 0.36 (100% EtOAc). **^{1}H NMR** (300 MHz, MeOD): δ ppm 3.87 (1H, dd, *J* = 11.4, 6.3, 6a-H), 3.81 (1H, hept, *J* = 6.9, isopropyl C*H*), 3.68 (1H, ddd, 12.3, 8.8, 5.5, 3-H_A), 3.02 (1H, ddd,12.2, 8.8, 5.6, 3-H_B), 2.52 – 2.34 (2H, m, 7-$H_{A,B}$), 2.00 – 1.81 (4H, m, 9a-*H*, 1-H_A, 2-$H_{A,B}$), 1.70 – 1.61 (1H, m, 8a-*H*), 1.53 – 1.37 (2H, m, 1-H_B, 9-H_A), 1.27 (1H, td, *J* = 9.0, 5.4, 9-H_B), 1.11 (3H, d, 6.9, isopropyl CH_{3A}), 1.10 (3H, d, 6.6, isopropyl CH_{3B}). **^{13}C NMR** (75 MHz, MeOD): δ ppm 208.9 (8-*C*), 164.7 (5-*C*), 66.1 (9b-*C*), 60.6 (6a-*C*), 46.1 (isopropyl *C*H), 45.0 (3-C), 41.4 (7-C), 37.3 (1-C), 28.41 (8a-*C*), 28.37 (9a-*C*), 23.5 (2-C), 22.1 (isopropyl *C*H_{3B}), 19.7 (isopropyl *C*H_{3A}), 16.5 (9-*C*). **IR** ν_{max} (neat)/cm^{-1}: 2972, 2938, 2892 (C-H), 1682 (C=O). **HRMS** (ESI): $C_{14}H_{21}N_2O_2$ [M + H$^+$]: calculated 249.1598, found 249.1598.

(6a*R,8a*R**,9a*S**,9b*S**)-6-(4-Methoxyphenyl)hexahydro-1*H*-cyclopropa[5,6]benzo[1,2-*d*]pyrrolo[1,2-c]imidazole-5,8(6*H*, 8a*H*)-dione 10b**

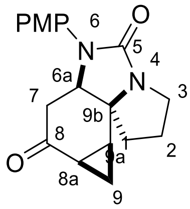

*(Assignment of stereochemistry: by analogy with **10a**)*

To a mixture of trimethylsulfoxonium chloride (240 mg, 1.87 mmol, 1.1 eq.) and 60% sodium hydride in mineral oil (74.9 mg, 1.87 mmol, 1.1 eq.) at 0 °C, 12.7 mL of anhydrous THF was added and the mixture was left to stir for 15 min. The ice bath was removed and the chalky mixture was left to stir at room temperature for 30 min. 3 mL of anhydrous DCM was then used to transfer the enone **2b** (508 mg, 0.54 mmol, 1.70 eq.) to the chalky mixture and was further left to stir overnight. The reaction mixture was extracted with DCM (5 × 20 mL) in 5 mL water, dried over sodium sulphate and concentrated *in vacuo*. Flash chromatography on silica gel eluting with 1% MeOH in DCM afforded the product **10b** (343 mg, 1.10 mmol, 64%). **R_f** = 0.56 (100% EtOAc). **^{1}H NMR** (500 MHz, $CDCl_3$): δ ppm 7.18 (2H, d, 9.5, Ar*H*), 6.80 (2H, d, 9.0, Ar*H*), 4.24 (1H, dd, *J* = 11.6, 5.9, 6a-H), 3.90 (1H, ddd, 12.0, 6.5, 3.5, 3-H_A), 3.71 (3H, s, ArOCH_3), 3.16 (1H, ddd,12.3, 9.3, 4.9, 3-H_B), 2.42 (1H, ddd, 14.1, 5.9,

1.3, 7-H_A), 2.28 (1H, dd, 14.0, 11.8, 7-H_B), 2.05 – 1.95 (3H, 1-$H_{A,B}$; 2-H_A), 1.96 – 1.91 (1H, m, 9a-*H*), 1.76 – 1.67 (1H, m, 2-H_B), 1.62 – 1.56 (1H, m, 8a-*H*), 1.45 (1H, q, *J* = 5.5, 9-H_A), 1.30 (1H, td, *J* = 8.9, 6.0, 9-H_B). **^{13}C NMR** (125 MHz, $CDCl_3$): δ ppm 205.1 (8-*C*), 159.7 (5-*C*), 155.8 (*Ar*), 129.2 (*Ar*), 122.4 (*Ar*H), 113.5 (*Ar*H), 62.6 (9b-*C*), 61.0 (6a-*C*), 54.5 (ArOCH_3), 43.2 (3-C), 36.7 (7-C), 36.0 (1-C), 26.9 (8a-*C*), 26.6 (9a-*C*), 22.0 (2-C), 14.8 (9-*C*). **IR** ν_{max} (neat)/cm^{-1}: 2960, 2837 (C-H), 1692 (C=O), 1611, 1512, 1460 (C=C), 1246 (C-O). **HRMS** (ESI): $C_{18}H_{21}N_2O_3$ [M + H$^+$]: calculated 313.1547, found 313.1545.

(4b*R, 10a*S**, 10b*S**, 11a*R**)-5-(4-Methoxyphenyl)-4b,9,10,10b,11,11a-hexahydro-8*H*-cyclopropa[*h*]pyrrolo[1',2':1,5]imidazo [4,5-*f*]quinolin-6(5*H*)-one 11b**

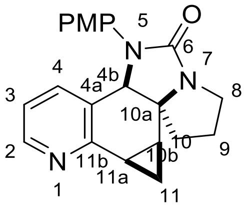

Anhydrous THF (1 mL) was added to a dry mixture of trimethylsulphoxonium chloride (11.6 mg, 0.09 mmol, 1.1 eq.) and 60% NaH in mineral oil (3.6 mg, 0.09 mmol, 1.1 eq.) and the mixture was stirred at 0 °C for 15 min after which it was allowed to warm up to room temperature for 30 min. 4 mL of anhydrous THF was then used to transfer the ketone **10b** (24 mg, 0.08 mmol, 1.0 eq.) to the reaction mixture and it was stirred for 19 h at room temperature. The reaction mixture was then extracted with DCM (5 × 20 mL), dried over $MgSO_4$ and evaporated *in vacuo*. A mixture of the crude, $NaAuCl_4.2H_2O$ (3.8 mg, 12 mol%) and propargylamine (10.2 µL, 0.16 mmol, 2.0) in HPLC grade EtOH was refluxed for 16 h. The reaction mixture was extracted with DCM (5 × 20 mL), dried over Na_2SO_4, and evaporated *in vacuo*. Flash chromatography eluting with 1 – 6 % MeOH in DCM afforded the product as a colourless oil (7 mg, 0.02 mmol, 25% yield over two steps). **^{1}H NMR** (600 MHz, $CDCl_3$) δ ppm 8.47 (1H, d, *J* = 4.8, Ar*H*), 7.29 (1H, t, *J* = 6.6, Ar*H*), 7.22 – 7.20 (1H, m, Ar*H*), 6.85 -6.80 (4H, m, Ar*H*), 4.73 (1H, s, 4b-*H*), 3.77 – 3.72 (4H, m, 8-H_A, *Ar*OCH_3*)*, 3.46 – 3.39 (2H, m, 8-H_B, 11a-*H*), 2.34 – 2.23 (1H, m, 10-H_A), 2.22 - 2.09 (2H, m, 9-$H_{A,B}$), 2.06 (1H, q, *J* = 7.2, 10-H_B), 1.83 (1H, dt, *J* = 12.7, 7.9, 10b-*H*), 1.71 (1H, td, *J* = 8.4, 5.4, 11-H_A), 1.65 (1H, dd, *J* = 7.8, 5.4, 11-H_B). **^{13}C NMR** (150 MHz, $CDCl_3$) δ ppm 159.7 (6-*C*), 158.4 (*Ar*), 155.8 (*Ar*), 145.6 (*Ar*H), 139.2 (*Ar*H), 129.0 (*Ar*), 128.5 (*Ar*H), 127.4 (*Ar*), 120.9 (*Ar*H), 114.1 (*Ar*H), 67.0 (4b-*C*), 60.6 (10a-*C*), 54.5 (ArOCH_3), 43.8 (8-*C*), 37.7 (10-*C*), 26.7 (9-*C*), 26.1 (11a-*C*), 18.7 (10b-*C*), 16.4 (11-*C*). **IR** ν_{max} (neat)/cm^{-1}: 2959, 2931 (C-H), 1700 (C=O), 1513, 1457, 1381 (C=C), 1248 (C-O). **HRMS** (ESI): $C_{21}H_{22}N_3O_2$ [M + H$^+$]: calculated 348.1707, found 348.1706.

***N*-(5*S**,6*R**,8*R**)-8-Hydroxy-1-methyl-1-azaspiro[4.5]decan-6-yl)-4-methylbenzenesulfonamide 12c**

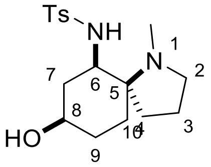

(Assignment of stereochemistry: X-ray crystal structure - CCDC deposition number: 2175095)

To a solution of the ketone **101c** (54 mg, 0.16 mmol, 1.0 eq.) in 4 mL of anhydrous THF, 1 M $LiAlH_4$ in THF (1.55 mL, 1.55 mmol, 10.0 eq.) was added and the mixture was refluxed for 6.5 h. The reaction mixture was quenched with 0.1 mL 10% KOH and and 0.1 mL H_2O, diluted with 50 mL EtOAc, dried over Na_2SO_4, and evaporated *in vacuo.* Flash chromatography eluting with 5% MeOH in DCM followed by 5% of a saturated solution of NH_3/MeOH in DCM afforded the product **122** as a pale yellow solid (28 mg, 0.08 mmol, 54% yield); $\mathbf{R_f}$ **=** 0.42 (6% of saturated NH_3/MeOH in DCM). **^{1}H NMR** (300 MHz, $CDCl_3$): δ ppm 7.69 (2H, d, *J* = 8.1, Ar*H*), 7.21 (2H, d, *J* = 8.2, Ar*H*), 3.78 (1H, tt, *J* = 6.0, 3.6, 8-*H*), 2.91 (1H, dd, *J* = 6.6, 4.4, 6-*H*), 2.64 - 2.52 (1H, m, 2-H_A), 2.46 – 2.37 (1H, m, 2-H_B), 2.35 (3H, s, ArCH_3), 2.31 (3H, s, 1-CH_3), 1.99 (1H, ddd, *J* = 14.1, 9.3, 4.2, 9-H_A), 1.83 (1H, dt, *J* = 13.4, 6.6, 7-H_A), 1.74 – 1.62 (2H, m, 7-H_B, 10-H_A), 1.60 – 1.47 (3H, m, 9-H_B, 4-H_A, 3-H_A), 1.47 – 1.30 (2H, m, 3-H_B, 4-H_B), 1.16 (1H, ddd, 13.8, 7.2, 4.5, 10-H_B). **^{13}C NMR** (75 MHz, $CDCl_3$): δ ppm 143.1 (*Ar*), 137.5 (*Ar*), 129.5 (*Ar*H), 127.2 (*Ar*H), 67.0 (8-*C*), 63.5 (5-*C*), 56.1 (6-*C*), 55.5 (2-*C*), 38.5 (1-*C*), 36.6 (4-*C*), 35.7 (7-*C*), 31.3 (9-*C*), 25.7 (10-*C*), 22.6 (3-*C*), 21.6 (Ar*C*H_3). **IR** ν_{max} (neat)/cm^{-1}: 3245 (O-H, N-H), 2929, 2871, 2787 (C-H), 1599, 1447, 1382 (C=C). **HRMS** (ESI): $C_{17}H_{27}N_2O_3S$ [M + H^+]: calculated 339.1737, found 339.1735.

(5*S,6*R**,8*R**)-6-(Isopropylamino)-1-methyl-1-azaspiro[4.5]decan-8-ol 13a**

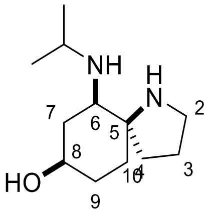

*(Assignment of stereochemistry: by analogy with **13b**)*

To a solution of the ketone **4a** (59 mg, 0.25 mmol, 1.0 eq.) in 6.5 mL of anhydrous THF, 4 M $LiAlH_4$ in ether (0.63 mL, 2.52 mmol, 10.0 eq.) was added and refluxed for 12 h. The reaction mixture was quenched with 0.1 mL 10% KOH and and 0.1 mL H_2O, diluted with 50 mL EtOAc, dried over Na_2SO_4, filtered and evaporated *in vacuo*. To a mixture of the crude and hydroxylamine hydrochloride (88 mg, 1.26 mmol, 5.0 eq.) was added 7 mL of 0.01% HCl and heated at 60 °C for 1 h. 29.3 mL of 0.5 M HCl was then added to the reaction mixture and washed with $CHCl_3$ (2 × 25 mL). The aqueous layer was carefully basified with copious amounts of solid Na_2CO_3, extracted with $CHCl_3$ (7 × 25 mL), dried over Na_2SO_4 and evaporated *in vacuo*. Flash chromatography eluting with 3% of a saturated solution of NH_3/MeOH in DCM afforded a brown solid **13a** (34 mg, 0.16 mmol, 64% yield); $\mathbf{R_f}$ = 0.33 (6%

NH_3/MeOH in DCM). **^{1}H NMR** (400 MHz, $CDCl_3$): δ ppm 3.87 (1H, quint, *J* = 2.4, 8-*H*), 2.91 (1H, ddd, *J* = 11.8, 7.6, 4.2, 2-H_A), 2.83 (1H, hept, *J* = 6.4, isopropyl C*H*), 2.61 (1H, dt, *J* = 11.6, 4.0, 2-H_B), 2.51 (1H, t, *J* = 3.2, 6-*H*), 2.04 (1H, td, *J* = 13.4, 4.2, 10-H_A), 1.99 - 1.93 (1H, m, 7-H_A), 1.84 – 1.45 (6H, m, 9-$H_{A,B}$; 3-$H_{A,B}$; 4-$H_{A,B}$), 1.31 (1H, dt, *J* = 14.4, 2.8, 7-H_B), 1.12 – 1.05 (1H, m, 10-H_B), 1.03 (3H, d, *J* = 6.4, isopropyl CH_{3A}), 0.95 (3H, d, *J* = 6.4, isopropyl CH_{3B}). **^{13}C NMR** (100 MHz, $CDCl_3$): δ ppm 66.9 (8-*C*), 64.9 (5-*C*), 56.0 (6-*C*), 45.5 (2-*C*), 45.4 (isopropyl *C*H), 35.2 (4-*C*), 31.8 (9-*C*), 29.9 (7-*C*), 28.1 (10-*C*), 27.2 (3-*C*), 24.5 (isopropyl *C*H_{3A}), 21.2 (isopropyl *C*H_{3B}). **IR** ν_{max} (neat)/cm^{-1}: 3256 (O-H, N-H), 2958, 2928, 2865 (C-H), 1135 (C-O). **HRMS** (ESI): $C_{12}H_{25}N_2O$ [M + H$^+$]: calculated 213.1961, found 213.1960.

5*S,6*R**,8*R**)-6-(4-Methoxyphenyl)amino-1-azaspiro[4.5]decan-8-ol 13b**

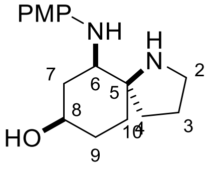

(Assignment of stereochemistry: X-ray crystal structure - CCDC deposition number: 2175097)

To a solution of the ketone **4b** (798 mg, 2.66 mmol, 1.0 eq.) in 33 mL of anhydrous THF, 2 M $LiAlH_4$ in THF (7.98 mL, 16.0 mmol, 6.0 eq.) was added and refluxed for 11.5 h. The reaction mixture was quenched with 2 mL 10% KOH and and 2 mL H_2O, diluted with 300 mL EtOAc, dried over Na_2SO_4, filtered and evaporated *in vacuo*. To a mixture of the crude and hydroxylamine hydrochloride (924 mg, 13.3 mmol, 5.0 eq.), 74 mL of 0.01% HCl was added and heated at 60 °C overnight. The reaction mixture was washed with $CHCl_3$ (2 × 50 mL). The aqueous layer was carefully basified with copious amounts of solid Na_2CO_3, extracted with $CHCl_3$ (7 × 50 mL), dried over Na_2SO_4 and evaporated *in vacuo*. Flash chromatography eluting with 8 – 10% MeOH in DCM followed by 6 - 10% of a saturated solution of NH_3/MeOH in DCM afforded a pale yellow oil **13b** (620 mg, 2.24 mmol, 84% yield); **R_f** = 0.37 (6% NH_3/MeOH in DCM). **^{1}H NMR** (400 MHz, $CDCl_3$): δ ppm 6.70 (2H, d, 8.8, Ar*H*), 6.58 (2H, d, 8.8, Ar*H*), 3.75 – 3.68 (1H, m, 8-*H*), 3.67 (3H, s, ArOCH_3), 3.00 (1H, dd, 8.3, 3.5, 6-*H*), 2.93 (1H, dt, *J* = 10.4, 6.7, 2-H_A), 2.81 (1H, dt, *J* = 10.4, 6.5, 2-H_B), 1.95 – 1.79 (3H, m, 7-H_A, 9-$H_{A,B}$), 1.76 – 1.36 (6H, m, 3-$H_{A,B}$; 4-$H_{A,B}$; 7-H_B; 10-H_A), 1.27 (1H, ddd, *J* = 13.6, 9.6, 4.0, 10-H_B). **^{13}C NMR** (100 MHz, $CDCl_3$): δ ppm 151.5 (*Ar*), 141.0 (*Ar*), 115.1 (*Ar*H), 113.9 (*Ar*H), 67.0 (8-*C*), 62.4 (5-*C*), 55.8 (6-*C*), 54.8 (ArO*C*H_3), 45.2 (2-*C*), 34.5 (9-*C*), 34.5 (4-*C*), 31.2 (10-*C*), 30.6 (7-*C*), 25.1 (3-*C*). **IR** ν_{max} (neat)/cm^{-1}: 3346 (O-H, N-H), 2931, 2862, 2832 (C-H), 1509, 1464, 1441 (C=C), 1232 (C-O). **HRMS** (ESI): $C_{16}H_{25}N_2O_2$ [M + H$^+$]: calculated 277.1911, found 277.1909.

(6a*R,8*S**,10a*S**)-8-((*tert*-Butyldimethylsilyl)oxy)-6-isopropyl-2,3,6,6a,7,8-hexahydro-1*H*,5*H*-benzo[*d*]pyrrolo[1,2-*c*]imidazol-5-one S7**

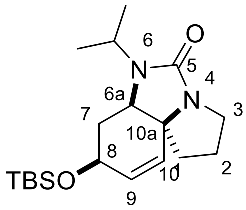

A mixture of enone **2a** (57.0 mg, 0.24 mmol, 1.0 eq.) and $CeCl_3.7H_2O$ (108 mg, 0.29 mmol, 1.2 eq.) in 2 mL of HPLC grade methanol was allowed to stir for 30 min at -78 °C after which $NaBH_4$ (11.0 mg, 0.29 mmol, 1.2 eq.) was added and the mixture was further stirred at the same temperature for 40 min. The reaction mixture was then allowed to warm up to room temperature for 1 h. It was extracted with DCM (5 × 20mL), dried over $MgSO_4$ and evaporated *in vacuo*. To a solution of the crude in 2 mL of anhydrous DCM; 2,6-lutidine (0.06 mL, 0.48 mmol, 2.0 eq.) and TBSOTf (0.08 mL, 0.36 mmol, 1.5 eq.) were added. The mixture was allowed to stir at room temperature for 15 h. It was extracted with DCM (5 × 20 mL), dried over $MgSO_4$ and evaporated *in vacuo*. Flash chromatography with 70 – 90% EtOAc in hexane afforded the product **S7** as a white solid (82 mg, 0.23 mmol, 96% yield over two steps); $\mathbf{R_f}$ = 0.82 (100% EtOAc). ^{1}**H NMR** (300 MHz, MeOD): δ ppm 5.67 (1H, dt, *J* = 10.2, 1.2, 10-*H*), 5.51 (1H, dd, *J* = 10.2, 2.1, 9-*H*), 4.28 – 4.19 (1H, m, 8-*H*), 3.83 (1H, hept, *J* = 6.9, isopropyl C*H*), 3.68 (1H, dd, *J* = 12.1, 4.9, 6a-*H*), 3.58 (1H, ddd, *J* = 12.2, 9.0, 5.8, 3-H_A), 2.91 (1H, ddd, *J* = 12.2, 9.2, 5.7, 3-H_B), 2.21 (1H, dtd, *J* = 11.3, 4.8, 1.4, 7-H_A), 1.89 – 1.67 (2H, m, 2-$H_{A,B}$), 1.55 (1H, ddd, *J* = 10.6, 7.9, 2.6, 1-H_A), 1.47 – 1.29 (2H, m, 1-H_B, 7-H_B), 1.14 (3H, d, *J* = 6.9, isopropyl CH_{3A}), 1.12 (3H, d, *J* = 6.6, isopropyl CH_{3B}), 0.79 (9H, s, tert-butyl CH_3), 0.00 (3H, s, SiCH_{3A}), -0.01 (3H, s, SiCH_{3B}). ^{13}**C NMR** (300MHz, MeOD): δ ppm 164.5 (5-*C*), 135.3 (10-*C*), 126.7 (9-*C*), 67.0 (8-*C*), 65.7 (10a-*C*), 55.8 (6a-*C*), 46.1 (isopropyl *C*H), 45.3 (3-*C*), 40.6 (7-*C*), 36.2 (1-*C*), 26.2 (tert-butyl CH_3), 23.6 (2-*C*), 22.4 (isopropyl CH_{3A}), 19.7 (isopropyl CH_{3B}), 18.9 (tert-butyl *C*), -4.5 (SiCH_{3B}), -4.7 (SiCH_{3A}). **IR** ν_{max} (neat)/cm^{-1}: 2956, 2930 (C-H), 1678 (C=O), 1508 (C=C), 1062 (C-O). **HRMS** (ESI): $C_{19}H_{34}N_2NaO_2Si$ [M + Na^+]: calculated 373.2282, found 373.2281.

(1*R, 7a*R**)-1-(2,3-Dihydoxypropyl)-7a-(hydroxymethyl)-2-isopropylhexahydro-3*H*-pyrrolo[1,2-*c*]imidazole-3-one 14a**

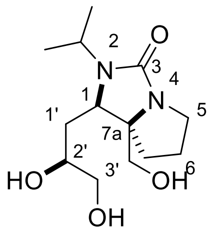

Through a solution of the alkene **S7** (40 mg, 0.11 mmol, 1.0 eq.) in 1 mL DCM, ozonized oxygen gas was passed at -78 °C until the solution turned blue in colour. Oxygen gas was then passed through the solution to get rid of the residual ozone (indicated by the disappearance of the blue colour). 8 mL

of methanol was added followed by a careful addition of $NaBH_4$ (excess) at -78 °C, and left to stand for 30 min at the same temperature. The reaction mixture was then allowed to warm up to room temperature overnight and evaporated *in vacuo*. It was extracted with EtOAc (5×20 mL) in 5 mL water, dried over Na_2SO_4 and evaporated *in vacuo*. To a solution of the crude in 4 mL THF, 0.10 mL of 1 M TBAF (in THF) was added and the mixture was allowed to stir overnight. The reaction mixture was evaporated *in vacuo* and taken up in 2 mL H_2O. It was then diluted with 100 mL EtOAc, dried over Na_2SO_4 and evaporated *in vacuo*. Flash chromatography with 4 – 10% MeOH in DCM afforded the product **14a** with 4% TBAF contamination (12.0 mg, 0.04 mmol, 39%); $\mathbf{R_f}$ = 0.23 (10% MeOH in DCM). **^{1}H NMR** (500 MHz, MeOD): δ ppm 3.84 (1H, qd, *J* = 6.6, 3.0, 2'-*H*), 3.80 – 3.68 (3H, m, 1-*H,* 5-H_A, isopropyl C*H*), 3.63 – 3.54 (3H, m, 3'-H_A, 7a-C$H_{2A,B}$), 3.42 (1H, dd, *J* = 11.0, 7.0, 3'-H_B), 2.97 – 2.92 (4H, m, 5-H_B, 2'-CHO*H*, 3'- CH$_2$O*H*, 7a- CH$_2$O*H*), 2.06 (1H, ddd, *J* = 14.5, 10.0, 2.5, 1'-H_A), 1.99 – 1.92 (1H, m, 7-H_A), 1.84 – 1.70 (3H, m, 1'-H_B, 6-$H_{A,B}$), 1.47 (1H, dt, *J* = 12.7, 8.8, 7-H_B), 1.24 (3H, d, *J* = 6.9, isopropyl CH_{3A}), 1.19 (3H, d, *J* = 6.8, isopropyl CH_{3B}). **^{13}C NMR** (125 MHz, MeOD): δ ppm 161.6 (3-*C*), 68.8 (7a-*C*), 68.3 (2'-*C*), 65.9 (3'-*C*), 60.8 (7a-*C*H$_2$OH), 58.2 (1-*C*), 45.1 (5-*C*), 44.5 (isopropyl *C*H), 32.7 (7-*C*), 31.7 (1'-*C*), 23.1 (6-*C*), 19.9 (isopropyl *C*H$_{3B}$), 18.6 (isopropyl *C*H$_{3A}$). **IR** ν_{max} (neat)/cm^{-1}: 3385 (O-H), 2962, 2927, 2876 (C-H), 1670 (C=O), 1053 (C-O). **HRMS** (ESI): $C_{13}H_{25}N_2O_4$ [M + H$^+$]: calculated 273.1809, found 273.1801.

(7a*R, 9*R**, 11a*S**)-9-Hydroxy-7-(4-methoxyphenyl)octahydro-1*H*-pyrrolo[1,2-*d*]quinoxalin-5(6*H*)-one 15b**

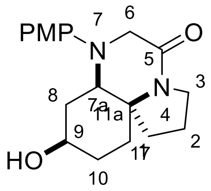

To a solution of the diamine **12b** (100 mg, 0.36 mmol, 1.0 eq.) in 14 mL THF was added chloroacetyl chloride (0.03 mL, 0.40 mmol, 1.1 eq.) and DIPEA (0.15 mL, 0.87 mmol, 2.4 eq.) at -78 °C. The reaction mixture was left to warm up to room temperature overnight. 2 M KOH (2 mL) was then added and also left to stir at room temperature overnight. The reaction mixture was extracted with EtOAc (5 × 20 mL), dried over Na_2SO_4 and evaporated *in vacuo*. Flash chromatography eluting with 1 – 4% MeOH in DCM afforded the product **15b** as a colourless oil (70 mg, 0.22 mmol, 61% yield); $\mathbf{R_f}$ **=** 0.38 (5% MeOH in DCM). **^{1}H NMR** (400 MHz, CDCl$_3$): δ ppm 7.01 (2H, d, *J* = 8.8, Ar*H*), 6.83 (2H, d, *J* = 8.8, Ar*H*), 4.34 (1H, s, 9-CHO*H*), 3.96 (1H, d, *J* = 17.6, 6-H_A), 3.79 – 3.66 (5H, m, 9-*H*, 3-H_A, ArOCH_3), 3.58 – 3.47 (1H, m, 3-H_B), 3.32 (1H, d, *J* = 17.6, 6-H_B), 3.24 (1H, t, *J* = 3.2, 7a-*H*), 2.27 – 2.06 (3H, m, 1-H_A, 8-H_A, 11-H_A), 2.02 – 1.92 (2H, m, 2-$H_{A,B}$), 1.89 – 1.80 (1H, m, 10-H_A), 1.59 – 1.46 (3H, m, 1-H_B, 10-H_B, 11-H_B), 1.41 (1H, dt, *J* = 15.0, 3.0, 8-H_B). **^{13}C NMR** (100 MHz, CDCl$_3$): δ ppm 164.0 (5-*C*), 156.1 (*Ar*),

142.0 (*Ar*), 124.2 (*Ar*H), 114.1 (*Ar*H), 64.6 (9-*C*), 62.7 (11a-*C*), 60.0 (7a-*C*), 58.4 (6-*C*), 54.4 (ArO*C*H$_3$), 43.2 (3-*C*), 33.6 (11-*C*), 29.9 (10-*C*), 29.5 (8-*C*), 24.3 (1-*C*), 19.8 (2-*C*). **IR** ν_{max} (neat)/cm^{-1}: 3434 (O-H), 2935, (C-H), 1642 (C=O), 1509, 1463, 1427 (C=C), 1242 (C-O). **HRMS** (ESI): $C_{18}H_{25}N_2O_3$ [M + H$^+$]: calculated 317.1860, found 317.1857.

Methyl 2-((5*S, 6*R**, 8*R**)-8-Hydroxy-6-((4-methoxyphenyl)amino)-1-azaspiro[4.5]decan-1-yl)acetate S8**

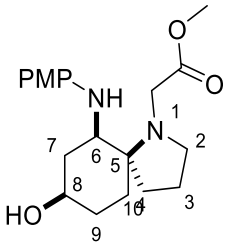

A mixture of the diamine **12b** (33 mg, 0.12 mmol, 1.0 eq), methyl bromoacetate (13.6 µL, 0.14 mmol, 1.2 eq.) and KHCO$_3$ (16 mg, 0.16 mmol, 1.3 eq.) in 2 mL of anhydrous THF was refluxed for 6 h and evaporated *in vacuo*. Flash chromatography eluting with 1 – 2% MeOH in DCM afforded the product **S8** as a brown oil (25 mg, 0.07 mmol, 60% yield); **R$_f$ =** 0.54 (6% MeOH in DCM). **^{1}H NMR** (400 MHz, CDCl$_3$) δ ppm 6.77 (2H, d, *J* = 9.2, Ar*H*), 6.72 (2H, d, *J* = 8.8, Ar*H*), 3.92 (1H, quint, *J* = 3.2, 8-*H*), 3.86 (1H, d, *J* = 16.8, pyrrolidine NC*H$_{2A}$*), 3.68 (3H, s, COOC*H$_3$*), 3.61 (3H, s, ArOC*H$_3$*), 3.21 (1H, d, *J* = 16.9, pyrrolidine NC*H$_{2B}$*), 3.11 (1H, ddd, *J* = 10.6, 7.7, 5.4, 2-*H$_A$*), 2.99 (1H, t, *J* = 3.3, 6-*H*), 2.64 (1H, dt, *J* = 10.5, 7.4, 2-*H$_B$*), 2.19 -2.06 (2H, m, 7-*H$_{A,B}$*), 1.91 – 1.81 (2H, m, 9-*H$_{A,B}$*), 1.79 – 1.50 (6H, m, 3-*H$_{A,B}$*; NH, 10-*H$_A$*, 4-*H$_{A,B}$*), 1.26 (1H, dt, 12.8, 3.7, 10-*H$_B$*). **^{13}C NMR** (100 MHz, CDCl$_3$) δ ppm 173.4 (ester *C*=O), 153.5 (*Ar*), 142.3 (*Ar*), 118.3 (*Ar*H), 114.6 (*Ar*H), 66.8 (8-*C*), 66.3 (5-*C*), 58.4 (6-*C*), 55.8 (COO*C*H$_3$), 53.9 (2-*C*), 52.8 (pyrrolidine N*C*H$_2$), 51.8 (ArO*C*H$_3$) 34.9 (4-*C*), 32.3 (7-*C*), 31.5 (9-*C*), 24.1 (10-*C*), 22.9 (3-*C*). **IR** ν_{max} (neat)/cm^{-1}: 3368 (O-H, N-H), 2933 (C-H), 1734 (C=O), 1509, 1439, 1365 (C=C), 1232 (C-O). **HRMS** (ESI): $C_{19}H_{29}N_2O_4$ [M + H$^+$]: calculated 349.2122, found 349.2120.

(7a*R,9*R**,11a*S**)-9-Hydroxy-7-(4-methoxyphenyl)octahydro-1H-pyrrolo[1,2-*d*]quinoxalin-6(5*H*)-one 16b**

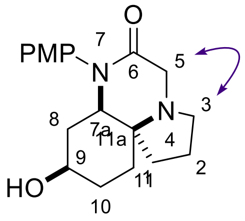

(Assignment of regiochemistry: NOE H3/H5)

A solution of the diamine **S8** (18 mg, 0.05 mmol, 1.0 eq.) and NaOMe (7.1 mg, 0.13 mmol, 1.1 eq.) in 2 mL of anhydrous MeOH was refluxed for 6 h. The reaction mixture was evaporated *in vacuo*. Flash chromatography eluting with 2 – 10% MeOH in DCM afforded the product **16b** as a brown oil (14 mg, 0.04 mmol, 88%); **R_f =** 0.26 (6% MeOH in DCM) When the reaction was telescoped from the diamine **12b** without further purification of the intermediate **S8,** the piperazinone **16b** was isolated in 90% yield. **^{1}H NMR** (400 MHz, $CDCl_3$): δ ppm 7.11 (2H, d, *J* = 9.2, Ar*H*), 6.85 (2H, d, *J* = 8.8, Ar*H*), 3.81 (1H, d, *J* = 17.6, 5-H_A), 3.73 (3H, s, ArOCH_3), 3.63 – 3.55 (2H, m, 9-*H*, 7a-*H*), 3.31 (1H, d, *J* = 17.6, 5-H_B), 3.05 (1H, ddd, *J* = 10.4, 6.4, 3.6, 3-H_A), 2.86 (1H, dt, 10.5, 6.8, 3-H_B), 2.05 (1H, dt, *J* = 13.4, 4.6, 8-H_A), 2.02 – 1.96 (1H, m, 10-H_A), 1.95 -1.89 (1H, m, 8-H_B), 1.85 – 1.82 (1H, m, 10-H_B), 1.82 – 1.77 (2H, m, 2-$H_{A,B}$), 1.77 – 1.60 (4H, m, 1-H_A; 11-$H_{A,B}$; 9-CHO*H*), 1.19 – 1.11 (1H, m, 1-H_B). **^{13}C NMR** (100 MHz, $CDCl_3$): δ ppm 169.7 (6-*C*), 158.3 (*Ar*), 133.9 (*Ar*), 127.9 (*Ar*H), 114.5 (*Ar*H), 67.6 (9-*C*), 62.2 (11a-*C*), 61.9 (7a-*C*), 55.6 (3-*C*), 55.5 (ArO*C*H_3), 54.5 (5-*C*), 36.9 (8-*C*), 36.5 (10-*C*), 30.7 (11-*C*), 30.2 (1-*C*), 23.0 (2-*C*). **IR** ν_{max} (neat)/cm^{-1}: 3378 (O-H), 2940, 2870 (C-H), 1642 (C=O), 1509, 1431, 1365 (C=C), 1242 (C-O). **HRMS** (ESI): $C_{18}H_{25}N_2O_3$ [M + H$^+$]: calculated 317.1860, found 317.1855.

(5a*R,11a*S**)-6-Isopropyloctahydro-9*H*-pyrrolo[1',2':1,5]imidazo[4,5-*c*]azepine-3,7-dione 17a**

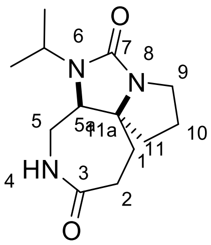

(Assignment of regiochemistry: COSY and X-ray crystal structure - CCDC deposition number: 2175092)

A solution of the ketone **4a** (51 mg, 0.22 mmol, 1.0 eq.), hydroxylamine hydrochloride (31 mg, 0.44 mmol, 2.0 eq.) and potassium carbonate (91.2 mg, 0.66 mmol, 3.0 eq.) in 3 mL EtOH/H_2O (2:1 respectively) was refluxed for 2 h. The reaction mixture was extracted with DCM (5 × 20 mL), dried over $MgSO_4$ and evaporated *in vacuo*. To a solution of the crude, TsCl (63.0 mg, 0.33 mmol, 1.5 eq.) and DMAP (2.69 mg, 0.02 mmol, 10 mol%) in 14 mL of anhydrous DCM; Et_3N (52 µL, 0.37 mmol, 1.7 eq.) was added and the mixture was allowed to stir at room temperature for 1.5 h. The reaction mixture was evaporated *in vacuo*. 1 mL of 98% sulphuric acid was then added to a solution of the crude in anhydrous MeOH and refluxed for 2 h. The reaction mixture was neutralized with copious amounts of solid Na_2CO_3 in 5 mL H_2O, extracted with DCM (5×40 mL), dried over $MgSO_4$ and evaporated *in vacuo*. Flash chromatography with 3 – 4% MeOH in DCM afforded the product **17a** as a pale yellow oil (28 mg, 0.11 mmol, 52%). **R_f** = 0.20 (5% MeOH in DCM). **^{1}H NMR** (500 MHz, $CDCl_3$): δ ppm 6.37 (1H, s, 4-*H*), 3.94 (1H, hept, *J* = 7.0, isopropyl C*H*), 3.79 (1H, ddd, *J* = 12.5, 4.5, 3.0, 9-H_A), 3.53 (1H, t, *J* = 5.5, 5a-*H*), 3.37 (2H, t, *J* = 6.0, 5-$H_{A,B}$), 2.84 (1H, ddd, *J* = 12.0, 5.5, 3.0, 9-H_B), 2.65 (1H,

ddd, *J* = 17.3, 9.4, 4.4, 2-H_A), 2.39 (1H, ddd, *J* = 17.3, 7.5, 4.1, 2-H_B), 1.90 – 1.69 (5H, m, 1-$H_{A,B}$; 10-$H_{A,B}$; 11-H_A), 1.42 – 1.31 (1H, m, 11-H_B), 1.18 (3H, d, *J* = 6.9, isopropyl CH_{3A}), 1.16 (3H, d, *J* = 6.9, isopropyl CH_{3B}). **^{13}C NMR** (125 MHz, $CDCl_3$): δ ppm 174.7 (3-*C*), 162.4 (7-*C*), 67.5 (11a-*C*), 58.8 (5a-*C*), 43.8 (isopropyl *C*H), 43.2 (9-*C*), 41.7 (5-*C*), 34.1 (11-*C*), 30.1 (2-*C*), 25.6 (1-*C*), 21.8 (10-*C*), 21.1 (isopropyl *C*H_{3A}), 18.6 (isopropyl *C*H_{3B}). **IR** ν_{max} (neat)/cm^{-1}: 3398 (N-H), 2976, 2945 (C-H), 1671, 1656 (C=O). **HRMS** (ESI): $C_{13}H_{22}N_3O_2$ [M + H$^+$]: calculated 252.1707, found 252.1707.

(5a*R,11a*S**)-6-(4-Methoxyphenyl)octahydro-9*H*-pyrrolo[1',2':1,5]imidazo[4,5-*c*]azepine-3,7-dione 17b** and **(5a*R**,11a*S**)-6-(4-methoxyphenyl)hexahydro-9*H*-pyrrolo[1',2':1,5]imidazo[4,5-*d*]azepine-4,7(1*H*,5*H*)-dione 17b'**

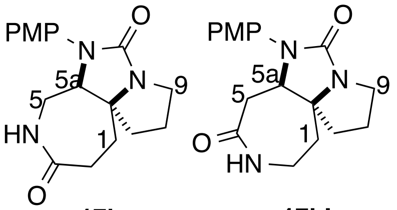

17b **17b'**

(Assignment of regiochemistry: COSY)

A solution of the ketone **4b** (127 mg, 0.42 mmol, 1.0 eq.), hydroxylamine hydrochloride (58.6 mg, 0.84 mmol, 2.0 eq.) and potassium carbonate (175 mg, 1.27 mmol, 3.0 eq.) in 8 mL EtOH/H_2O (2:1 respectively) was refluxed for 2 h. The reaction mixture was evaporated *in vacuo* and taken up in 2 mL H_2O. It was then diluted with 100 mL EtOAc, dried over Na_2SO_4 and evaporated *in vacuo*. To a solution of the crude, TsCl (121 mg, 0.63 mmol, 1.5 eq.), and DMAP (5.16 mg, 0.04 mmol, 10 mol%) in 14 mL of anhydrous DCM; Et_3N (0.10 mL, 0.72 mmol, 1.7 eq.) was added and the mixture was allowed to stir at room temperature for 1.5 h. The reaction mixture was evaporated *in vacuo*. 2 mL of 98% sulphuric acid was then added to a solution of the crude in 7 mL of anhydrous MeOH and refluxed for 2 h. The reaction mixture was neutralized with copious amounts of solid Na_2CO_3 in 10 mL H_2O, extracted with EtOAc (5 × 50 mL), dried over Na_2SO_4 and evaporated *in vacuo*. Flash chromatography with 1 – 6% MeOH in DCM afforded the product as an isomeric mixture in the ratio of 93:7 **17b/b'** (83 mg, 0.26 mmol, 62%). **R_f** = 0.36 (6% MeOH in DCM). **^{1}H NMR** (Major isomer, 400 MHz, $CDCl_3$): δ ppm 7.20 (2H, d, *J* = 9.2, Ar*H*), 6.83 (2H, d, *J* = 8.8, Ar*H*), 6.26 (1H, s, 4-*H*), 4.08 (1H, t, *J* = 5.1, 5a-*H*), 3.84 (1H, ddd, *J* = 14.0, 9.6, 4.9, 9-H_A), 3.72 (3H, s, ArOCH_3), 3.30 (2H, t, *J* = 4.9, 5-$H_{A,B}$), 2.98 (1H, ddd, *J* = 14.4, 9.2, 5.6, 9-H_B), 2.69 (1H, ddd, *J* = 17.6, 8.8, 5.6, 2-H_A), 2.45 (1H, dt, *J* = 17.6, 5.6, 2-H_B), 1.98 – 1.77 (5H, m, 1-$H_{A,B}$; 10-$H_{A,B}$; 11-H_A), 1.71 – 1.61 (1H, m, 11-H_B). Signals for minor isomer visible at: 4.15 (0.07H, dd, *J* = 10.6, 2.9, 5a-*H*), 2.80 (0.08H, dd, *J* = 14.8, 10.6, 5-*H*). **^{13}C NMR** (Major isomer, 100 MHz, $CDCl_3$): δ ppm 175.3 (3-*C*), 161.5 (7-*C*), 157.1 (*Ar*), 130.3 (*Ar*), 124.4 (*Ar*H), 114.6 (*Ar*H), 67.4 (11a-*C*), 63.0 (5a-*C*), 55.6 (ArO*C*H_3), 44.4 (9-*C*), 40.4 (5-*C*), 36.0 (11-*C*), 31.3 (2-*C*),

26.9 (1-*C*), 23.1 (10-*C*). Signals for minor isomer visible at: ppm 124.8, 59.3, 50.8, 38.2, 35.4, 32.2, 23.5. **IR** ν_{max} (neat)/cm^{-1}: 3291 (N-H), 3052, 2954, 2837 (C-H), 1693, 1659 (C=O), 1513, 1465, 1403 (C=C), 1246 (C-O). **HRMS** (ESI): $C_{17}H_{22}N_3O_3$ [M + H$^+$]: calculated 316.1656, found 316.1657.

(5a*R,11a*S**)-6-(4-Toluenesulfonyl)octahydro-9*H*-pyrrolo[1',2':1,5]imidazo[4,5-*c*]azepine-3,7-dione 17c** and **(5a*R**,11a*S**)-6-(4-toluenesulfonyl)hexahydro-9*H*-pyrrolo[1',2':1,5]imidazo[4,5-*d*]azepine-4,7(1*H*,5*H*)-dione 17c'**

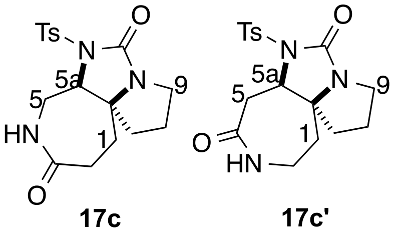

17c **17c'**

(Assignment of regiochemistry: COSY)

A solution of the ketone **4c** (110 mg, 0.32 mmol, 1.0 eq.), hydroxylamine hydrochloride (45.0 mg, 0.64 mmol, 2.0 eq.) and potassium carbonate (134 mg, 0.96 mmol, 3.0 eq.) in 6 mL EtOH/H_2O (2:1 respectively) was refluxed for 2 h. The reaction mixture was evaporated *in vacuo* and taken up in 2 mL H_2O. It was then diluted with 100 mL EtOAc, dried over Na_2SO_4 and evaporated *in vacuo*. To a solution of the crude, TsCl (93.0 mg, 0.48 mmol, 1.5 eq.), and DMAP (3.95 mg, 0.03 mmol, 10 mol%) in 12 mL of anhydrous DCM; Et_3N (0.08 mL, 0.55 mmol, 1.7 eq.) was added and the mixture was allowed to stir at room temperature for 1.5 h. The reaction mixture was evaporated *in vacuo*. 1.5 mL of 98% sulphuric acid was then added to a solution of the crude in 5 mL of anhydrous MeOH and refluxed for 2 h. The reaction mixture was neutralized with copious amounts of solid Na_2CO_3 in 10 mL H_2O, extracted with EtOAc (5 × 50 mL), dried over Na_2SO_4 and evaporated *in vacuo*. Flash chromatography with 1 – 4% MeOH in DCM afforded the product as an isomeric mixture in the ratio of 90:10 **17c/c'** (46 mg, 0.13 mmol, 40%). $\mathbf{R_f}$ = 0.41 (5% MeOH in DCM). **^{1}H NMR** (Major isomer, 400 MHz, $CDCl_3$): δ ppm 7.85 (2H, d, *J* = 8.3, Ar*H*), 7.27 (2H, d, *J* = 8.1, Ar*H*), 6.04 (1H, t, *J* = 7.2, 4-*H*), 4.16 (1H, dd, *J* = 6.8, 2.1, 5a-*H*), 3.75 (1H, ddd, 15.5, 6.2, 2.2, 9-H_A), 3.72 – 3.59 (2H, m, 5-$H_{A,B}$), 2.92 – 2.82 (1H, m, 9-H_B), 2.56 (1H, apdt, *J* = 18.0, 7.2, 2-H_A), 2.46 (1H, dt, *J* = 17.6, 5.5, 2-H_B), 2.37 (3H, s, ArCH_3), 1.86 – 1.69 (5H, m, 1-$H_{A,B}$; 10-$H_{A,B}$; 11-H_A), 1.25 – 1.09 (1H, m, 11-H_B). Signals for minor isomer visible at: 7.91 (0.23H, d, *J* = 8.3, Ar*H*), 6.33 (0.11H, s, 3-*H*), 4.43 (0.11H, dd, *J* = 7.6, 5.6, 5a-*H*). **^{13}C NMR** (Major isomer, 100 MHz, $CDCl_3$): δ ppm 173.3 (3-*C*), 156.6 (7-*C*), 144.2 (*Ar*), 134.4 (*Ar*), 128.8 (*Ar*H), 127.0 (*Ar*H), 67.1 (11a-*C*), 61.0 (5a-*C*), 43.0 (9-*C*), 41.4 (5-*C*), 34.7 (11-*C*), 29.8 (2-*C*), 24.9 (1-*C*), 21.5 (10-*C*), 20.7 (Ar*C*H_3). Signals for minor isomer visible at: 170.0, 128.6, 127.5, 57.4, 49.8, 37.6, 36.9, 34.7, 33.9, 21.9. **IR** ν_{max} (neat)/cm^{-1}: 3228 (N-H), 3062, 2960 (C-H), 1726, 1657 (C=O), 1596, 1464, 1400 (C=C) **HRMS** (ESI): $C_{17}H_{22}N_3O_4S$ [M + H$^+$]: calculated 364.1326, found 364.1323.

(2a*R,5a*R**,6a*S**,6b*S**)-2-Isopropyloctahydro-1*H*,7*H*-cyclopropa[*c*]pyrrolo[1',2':3,4]imidazo[4,5-*e*]azepine-1,5(5a*H*)-dione 18a** and **(2a*R**,5a*R**,6a*S**,6b*S**)-2-isopropyloctahydro-1*H*,7*H*-cyclopropa[*b*]pyrrolo[1',2':3,4]imidazo[4,5-*d*]azepine-1,4(2*H*)-dione 18a'**

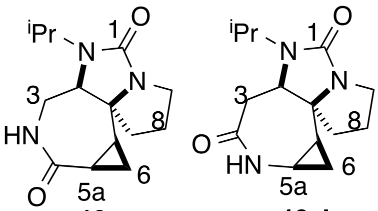

18a **18a'**

*(Assignment of regiochemistry: COSY for **18a** and X-ray crystal structures - CCDC deposition number: 2175093 for **18a** and 2224747 for **18a'**)*

A solution of the cyclopropane scaffold **4a** (50.0 mg, 0.20 mmol, 1.0 eq.), hydroxylamine hydrochloride (28.0 mg, 0.40 mmol, 2.0 eq.) and potassium carbonate (83.5 mg, 0.60 mmol, 3.0 eq.) in 3 mL EtOH/H_2O (2:1 respectively) was refluxed for 2 h. The reaction mixture was evaporated *in vacuo* and taken up in 2 mL H_2O. It was then diluted with 100 mL EtOAc, dried over Na_2SO_4 and evaporated *in vacuo*. To a solution of the crude, TsCl (57.6 mg, 0.30 mmol, 1.5 eq.) and DMAP (2.46 mg, 0.02 mmol, 10 mol%) in 14 mL of anhydrous DCM; Et_3N (47.0 µL, 0.34 mmol, 1.7 eq.) was added and the mixture was allowed to stir at room temperature for 1.5 h. The reaction mixture was evaporated *in vacuo*. 0.90 mL of 98% sulphuric acid was then added to a solution of the crude in 3 mL of anhydrous MeOH and refluxed for 2 h. The reaction mixture was neutralized with copious amounts of solid Na_2CO_3 in 5 mL H_2O, extracted with EtOAc (5×20 mL), dried over Na_2SO_4 and evaporated *in vacuo*. Flash chromatography with 1 – 6% MeOH in DCM afforded the isomeric lactams **18a** [major isomer, 12 mg, 0.05 mmol, 23%, $\mathbf{R_f}$ = 0.31 (6% MeOH in DCM)] and **18a'** [minor isomer, 11 mg, 0.04 mmol, 21%, $\mathbf{R_f}$ = 0.37 (6% MeOH in DCM)]. **^{1}H NMR** (**Major isomer 18a**, 500 MHz, $CDCl_3$): δ ppm 5.71 (1H, s, 4-*H*), 4.02 (1H, hept, *J* = 7.0, isopropyl C*H*), 3.93 (1H, dd, *J* = 15.2, 6.7, 3-H_A), 3.60 (1H, dt, *J* = 11.7, 7.8, 9-H_A), 3.54 (1H, br. s, 2a-*H*), 3.11 (1H, ddd, *J* = 15.4, 6.2, 2.5, 3-H_B), 2.97 – 2.87 (1H, m, 9-H_B), 1.87 – 1.79 (3H, m, 7-H_A, 8-$H_{A,B}$), 1.71 – 1.65 (1H, m, 5a-*H*), 1.52 – 1.43 (1H, m, 7-H_B), 1.18 (3H, d, *J* = 6.9, isopropyl CH_{3A}), 1.14 (3H, d, *J* = 6.9, isopropyl CH_{3B}), 1.02 – 0.96 (1H, m, 6-H_A). 0.90 (1H, td, *J* = 8.5, 5.6, 6a-*H*), 0.73 (1H, q, *J* = 5.5, 6-H_B). **^{13}C NMR** (**Major isomer 18a**, 125 MHz, $CDCl_3$): δ ppm 170.5 (5-*C*), 163.0 (1-*C*), 66.5 (6b-*C*), 57.3 (2a-*C*), 46.4 (9-*C*), 43.5 (isopropyl *C*H), 42.7 (3-*C*), 37.5 (7-*C*), 22.2 (8-*C*), 21.3 (isopropyl *C*H_{3A}), 18.5 (isopropyl *C*H_{3B}), 15.7 (6a-*C*), 15.0 (5a-*C*), 7.1 (6-*C*). **IR** ν_{max} (neat)/cm^{-1}: 3320 (N-H), 2968, 2904, 2878 (C-H), 1681 (C=O). **HRMS** (ESI): $C_{14}H_{22}N_3O_2$ [M + H$^+$]: calculated 264.1707, found 264.1701. **^{1}H NMR** (**Minor isomer 18a'**, 500 MHz, $CDCl_3$): δ ppm 5.52 (1H, s, 5-*H*), 3.83 (1H, t, *J* = 3.5, 2a-*H*), 3.78 (1H, hept, *J* = 7.0, isopropyl C*H*), 3.68 – 3.61 (1H, m, 9-H_A), 3.12 (1H, dd, *J* = 13.5, 3.0, 3-H_A), 2.97 – 2.89 (1H, m, 9-H_B), 2.65 (1H, td, *J* = 7.2, 4.2, 5a-*H*), 2.47

(1H, ddd, *J* = 13.5, 3.9, 1.7, 3-H_B), 1.93 – 1.77 (3H, m, 7-H_A, 8-$H_{A,B}$), 1.48 (1H, td, *J* = 11.5, 9.2, 7-H_B), 1.25 (3H, d, *J* = 6.9, isopropyl CH_{3A}), 1.23 (3H, d, *J* = 6.9, isopropyl CH_{3B}), 1.00 (1H, ddd, *J* = 9.0, 7.3, 5.0, 6-H_A). 0.87 – 0.79 (1H, m, 6a-*H*), 0.73 (1H, dt, *J* = 6.5, 4.5, 6-H_B). ^{13}**C NMR** (**Minor isomer 18a'**, 125 MHz, $CDCl_3$): δ ppm 171.2 (4-*C*), 162.1 (1-*C*), 66.3 (6b-*C*), 56.6 (2a-*C*), 45.3 (9-*C*), 44.5 (isopropyl *C*H), 39.0 (7-*C*), 36.8 (3-*C*), 25.4 (5a-*C*), 22.6 (8-*C*), 20.4 (isopropyl CH_{3A}), 19.1 (6a-*C*), 18.4 (isopropyl CH_{3B}), 12.1 (6-*C*). **IR** ν_{max} (neat)/cm^{-1}: 3266 (N-H), 2968, 2930 (C-H), 1684 (C=O). **HRMS** (ESI): $C_{14}H_{22}N_3O_2$ [M + H^+]: calculated 264.1707, found 264.1704.

(2a*R,5a*R**,6a*S**,6b*S**)-2-(4-Methoxyphenyl)octahydro-1*H*,*7H*-cyclopropa[*c*]pyrrolo[1',2':3,4]imidazo[4,5-*e*]azepine-1,5(5a*H*)-dione 18b**

(2a*R,5a*R**,6a*S**,6b*S**)-2-Isopropyloctahydro-1*H*,*7H*-cyclopropa[*b*]pyrrolo[1',2':3,4]imidazo[4,5-*d*]azepine-1,4(2*H*)-dione 18b'**

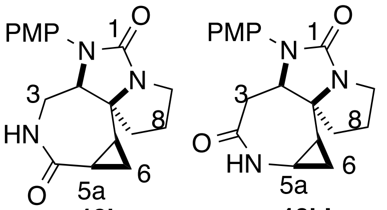

18b **18b'**

*(Assignment of regiochemistry: COSY and by analogy with **18a/a'**)*

A solution of the cyclopropane scaffold **4b** (101 mg, 0.32 mmol, 1.0 eq.), hydroxylamine hydrochloride (45.2 mg, 0.65 mmol, 2.0 eq.) and potassium carbonate (134 mg, 0.97 mmol, 3.0 eq.) in 6 mL EtOH/H_2O (2:1 respectively) was refluxed for 2 h. The reaction mixture was evaporated *in vacuo* and taken up in 2 mL H_2O. It was then diluted with 100 mL EtOAc, dried over Na_2SO_4 and evaporated *in vacuo*. To a solution of the crude, TsCl (93.4 mg, 0.49 mmol, 1.5 eq.), and DMAP (3.95 mg, 0.03 mmol, 10 mol%) in 14 mL of anhydrous DCM; Et_3N (0.08 mL, 0.55 mmol, 1.7 eq.) was added and allowed to stir at room temperature for 1.5 h. The reaction mixture was evaporated *in vacuo*. 1.5 mL of 98% sulphuric acid was then added to a solution of the crude in 5 mL of anhydrous MeOH and refluxed for 2 h. The reaction mixture was neutralized with copious amounts of solid Na_2CO_3 in 10 mL H_2O, extracted with EtOAc (5 × 50 mL), dried over Na_2SO_4 and evaporated *in vacuo*. Flash chromatography with 1 – 6% MeOH in DCM afforded the product as an isomeric mixture of **18b**/**b'** in the ratio of 78:22 (83 mg, 0.26 mmol, 62%). $\mathbf{R_f}$ = 0.36 (6% MeOH in DCM). ^{1}**H NMR** (**Major isomer 18b**, 500 MHz, $CDCl_3$): δ ppm 7.22 (2H, d, *J* = 9.0, Ar*H*), 6.83 (2H, d, *J* = 9.0, Ar*H*), 5.62 (1H, s, 4-*H*), 4.34 (1H, t, *J* = 3.4, 2a-*H*), 3.82 – 3.73 (1H, m, 9-H_A), 3.72 (3H, s, ArOCH_3), 3.08 (1H, dt, 12.5, 3.5, 9-H_B), 3.03 (1H, dd, *J* = 13.5, 3.0, 3-H_A), 2.68 (1H, td, *J* = 7.0, 4.0, 5a-*H*), 2.47 (1H, ddd, *J* = 13.8, 3.4, 1.7, 3-H_B), 2.05 (1H, ddd, *J* = 11.5, 5.0, 1.5, 7-H_A), 1.97 – 1.92 (2H, m, 8-$H_{A,B}$), 1.81 (1H, dt, *J* = 11.0, 9.0, 7-H_B), 1.07 (1H, ddd, 9.5, 4.5, 2.0, 6-H_A), 0.97 – 0.91 (1H, m, 6a-*H*), 0.84 – 0.79 (1H, m, 6-H_B). Signal for

minor isomer **18b'** visible at: 5.50 (0.28H, t, *J* = 5.5, 5-*H*). ^{13}**C NMR** (**Major isomer 18b**, 125 MHz, $CDCl_3$): δ ppm 170.9 (5-*C*), 160.7 (1-*C*), 156.4 (*Ar*), 129.3 (*Ar*), 125.4 (*Ar*H), 113.4 (*Ar*H), 65.6 (6b-*C*), 59.7 (2a-*C*), 54.4 (ArO*C*H_3), 45.6 (9-*C*), 39.2 (7-*C*), 35.1 (3-*C*), 25.2 (5a-*C*), 22.8 (8-*C*), 19.4 (6a-*C*), 12.1 (6-*C*). Signals for minor isomer **18b'** visible at: 155.9, 122.9, 113.7, 65.4, 61.0, 54.5, 46.4, 39.9, 38.3, 22.4, 16.2, 15.3, 7.1. **IR** ν_{max} (neat)/cm^{-1}: 3300 (N-H), 2933 (C-H), 1691 (C=O), 1585, 1513, 1458 (C=C), 1246 (C-O). **HRMS** (ESI): $C_{18}H_{22}N_3O_3$ [M + H$^+$]: calculated 328.1656, found 328.1653.

Structures and Synthesis of Screening Compounds

(a) by functionalisation of the tricyclic cores (20 compounds)

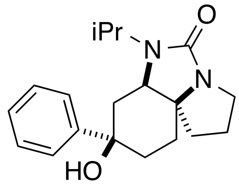

5a

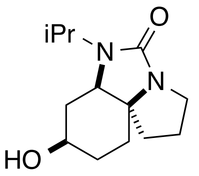

6a

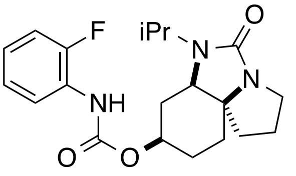

31

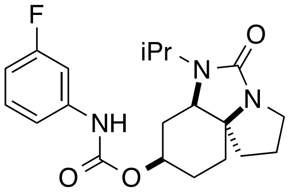

20

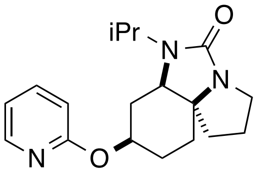

21

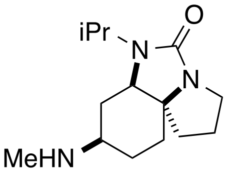

7a

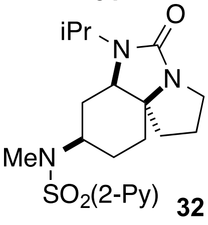

32

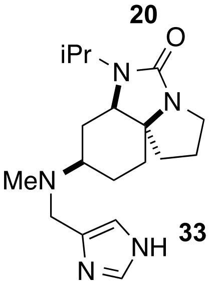

33

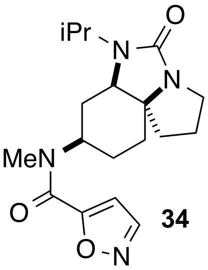

34

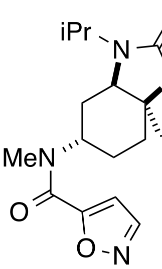

35

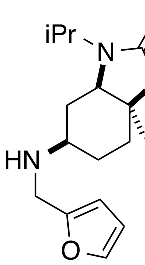

36

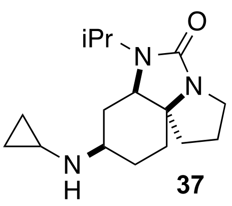

37

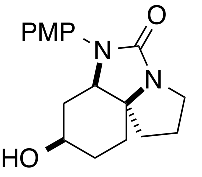

6b

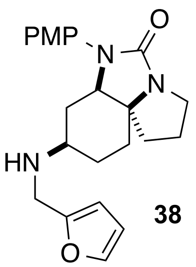

38

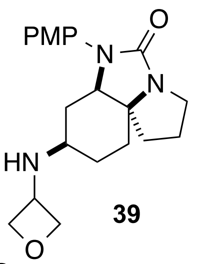

39

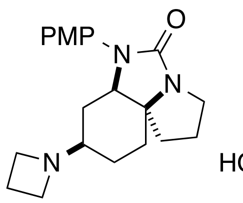

40

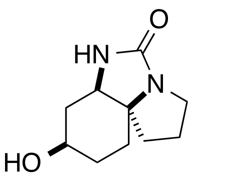

41

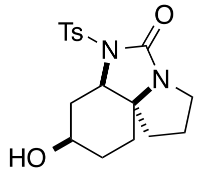

6c

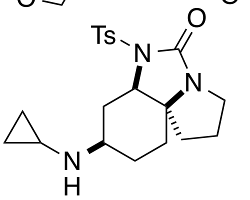

27

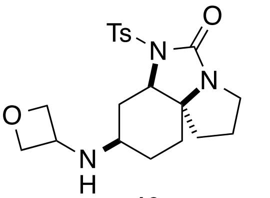

19

(6a*R,8*R**,10a*S**)-8-(Oxetan-3-ylamino)-6-(4-toluenesulfonyl)octahydro-1*H*,5*H*-benzo[*d*]pyrrolo[1,2-*c*]imidazol-5-one 19**

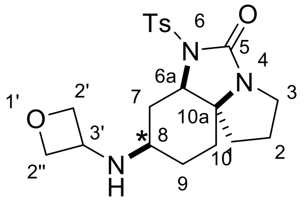

Major diastereomer, *dr* 85:15

*(Assignment of stereochemistry: by analogy with **7a**)*

To a solution of the ketone **4c** (55.0 mg, 0.16 mmol, 1.0 eq.) in 6 mL THF, 3-aminooxetane (22.0 µL, 0.32 mmol, 2.0 eq.) and titanium isopropoxide (0.10 mL, 0.32 mmol, 2.0 eq.) were added and the mixture was left to stir at room temperature overnight. $NaBH_4$ (9.10 mg, 0.24 mmol, 1.5 eq.) was added to the reaction mixture at -78 °C and it was stirred at the same temperature for 2 h. It was then allowed to warm up to room temperature for 1 h. The reaction mixture was evaporated *in vacuo*. It was then taken up in 2 mL H_2O, diluted with 100 mL EtOAc, dried over Na_2SO_4 and evaporated *in vacuo*. Flash chromatography with 1 - 2% MeOH in DCM afforded the product as an 85:15 mixture of diastereomers (26.0 mg, 0.06 mmol, 41% yield). $\mathbf{R_f}$ = 0.38. **^{1}H NMR** (Major diastereomer, 500 MHz, $CDCl_3$): δ ppm 7.86 (2H, d, *J* = 8.5, Ar*H*), 7.25 (2H, d, *J* = 8.0, Ar*H*), 4.77 (1H, t, *J* = 7.0, 2'-H_A), 4.74 (1H, t, *J* = 6.5, 2''-H_A), 4.32 (1H, t, *J* = 6.5, 2'-H_B), 4.30 (1H, t, *J* = 6.5, 2''-H_B), 4.15 (1H, dd, *J* = 10.3, 6.2, 6a-*H*), 3.97 (1H, quint, *J* = 6.7, 3'-*H*), 3.70 (1H, ddd, *J* = 12.4, 9.1, 5.6, 3-H_A), 2.86 (1H, ddd, *J* = 12.0, 6.5, 3.0, 3-H_B), 2.54 – 2.45 (2H, m, 7-H_A, 8-*H*), 2.36 (3H, s, ArCH_3), 1.81 (1H, dt, *J* =15.0, 4.0, 10-H_A), 1.78 – 1.70 (2H, m, 2-$H_{A,B}$), 1.59 – 1.08 (7H, m, 1-$H_{A,B}$; 7-H_B; 8-CHN*H*; 9-$H_{A,B}$; 10-H_B). Signals for minor isomer visible at: 4.21 (0.17H, t, *J* = 6.0, 6a-*H*), 3.88 (0.18H, quint, *J* = 6.5, 3'-*H*), 3.61 (0.20H, dt, *J* = 12.0, 7.6, 3-*H*). **^{13}C NMR** (Major diastereomer, 125 MHz, $CDCl_3$): δ ppm 158.5 (5-*C*), 144.8 (*Ar*), 136.3 (*Ar*), 129.6 (*Ar*H), 128.1 (*Ar*H), 80.6 (2'-*C*), 80.5 (2''-*C*), 65.4 (10a-*C*), 57.6 (6a-*C*), 51.6 (8-*C*), 51.5 (3'-*C*), 44.0 (3-*C*), 38.3 (7-*C*), 34.4 (1-*C*), 29.0 (10-*C*), 28.2 (9-*C*), 22.8 (2-*C*), 21.7 (Ar*C*H_3). Signals for minor isomer visible at: 157.6, 144.7, 136.2, 128.0, 80.3, 80.0, 65.5, 58.5, 51.4, 47.0, 43.7, 35.5, 33.8, 26.0, 23.2. **IR** ν_{max} (neat)/cm^{-1}: 3303 (N-H), 2946, 2867 (C-H), 1727 (C=O), 1596, 1494, 1461 (C=C), 1168 (C-O). **HRMS** (ESI): $C_{20}H_{28}N_3O_4S$ [M + H$^+$]: calculated 405.1795, found 405.1802.

(6a*R,8*R**,10a*S**)-6-Isopropyl-5-oxooctahydro-1*H*,5*H*-benzo[*d*]pyrrolo[1,2-*c*]imidazol-8-yl (3-fluorophenyl)carbamate 20**

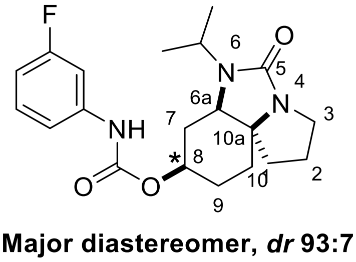

Major diastereomer, *dr* 93:7

A mixture of the ketone **4a** (27.0 mg, 0.11 mmol, 1.0 eq.) and $CeCl_3.7H_2O$ (51.0 mg, 0.14 mmol, 1.2 eq.) in 3 mL of HPLC grade MeOH was allowed to stir at – 78 °C for 30 min. $NaBH_4$ (5.18 mg, 0.14 mmol, 1.2 eq.) was then added and the mixture was left to warm up to room temperature for another 30 min. The reaction mixture was evaporated *in vacuo*. It was then taken up in 1 mL H_2O, diluted with 50 mL EtOAc, dried over Na_2SO_4 and evaporated *in vacuo*. To a solution of the crude

product in 5 mL DCM, 3-fluorophenylisocyanate (13 µL, 0.11 mmol, 1.0 eq.) and TEA (0.05 mL, 0.34 mmol, 3.0 eq.) were added and the mixture was stirred at room temperature overnight. The reaction mixture was evaporated *in vacuo*. Flash chromatography with 1 – 4% MeOH in DCM afforded the carbamate as a diastereomeric mixture in the ratio of 93:7 (36 mg, 0.10 mmol, 84% yield). **^{1}H NMR** (Major diastereomer, 500 MHz, $CDCl_3$): δ ppm 7.30 (1H, d, *J* = 10.6, Ar*H*), 7.17 (1H, td, *J* = 8.2, 6.6, Ar*H*), 7.00 (1H, d, *J* = 8.0, Ar*H*), 6.68 (1H, td, *J* = 8.3, 2.3, Ar*H*), 4.69 (1H, tt, *J* = 10.8, 4.0, 8-*H*), 3.99 (1H, hept, *J* = 6.9, isopropyl C*H*), 3.79 (1H, ddd, *J* = 12.4, 9.3, 5.2, 3-H_A), 3.52 (1H, dd, *J* = 9.9, 6.5, 6a-*H*), 2.86 (1H, ddd, *J* =12.5, 7.0, 4.0, 3-H_B), 2.43 – 2.36 (1H, m, 7-H_A), 1.88 (1H, dt, *J* = 15.0, 4.0, 10-H_A), 1.85 – 1.80 (1H, m, 9-H_A), 1.77 (1H, ddd, *J* = 12.5, 6.0, 3.0, 1-H_A), 1.75 – 1.66 (1H, m, 2-H_A), 1.62 – 1.40 (4H, m, 1-H_B, 2-H_B, 7-H_B, 9-H_B), 1.37 (1H, ddd, *J* = 14.6, 10.8, 4.7, 10-H_B), 1.14 (3H, d, *J* = 6.9, isopropyl CH_{3A}), 1.12 (3H, d, *J* = 6.9, isopropyl CH_{3B}). Signals for minor isomer visible at: 5.06 (0.07H, quint, *J* = 4.5, 8-*H*), 3.70 (0.07H, dd, *J* = 8.2, 5.7, 6a-*H*). **^{13}C NMR** (Major diastereomer, 125 MHz, $CDCl_3$): δ ppm 162.6 (5-*C*), 162.2 (d, *J* = 243, *Ar*F), 151.8 (carbamate *C*=O), 138.7 (d, *J* =13.2, *Ar*), 129.1 (d, *J* = 9.50, *Ar*H), 112.8 (*Ar*H), 108.9 (d, *J* = 21.3, *Ar*H), 104.9 (d, *J* = 26.8, *Ar*H), 69.3 (8-*C*), 64.4 (10a-*C*), 53.2 (6a-*C*), 43.3 (isopropyl *C*H), 43.0 (3-*C*), 36.9 (7-*C*), 33.6 (1-*C*), 27.6 (10-*C*), 26.0 (9-*C*), 21.9 (2-*C*), 21.4 (isopropyl CH_{3A}), 18.7 (isopropyl CH_{3B}). Signals for minor isomer visible at: 52.1, 34.4, 25.1, 24.1, 22.2, 21.2. **IR** ν_{max} (neat)/cm^{-1}: 3254 (N-H); 3076, 2968 (C-H); 1723, 1671 (C=O); 1606, 1546, 1495 (C=C); 1221 (C-O). **HRMS** (ESI): $C_{20}H_{27}FN_3O_3$ [M + H$^+$]: calculated 376.2031, found 376.2028.

(6a*R,8*R**,10a*S**)-6-Isopropyl-8-(pyridine-2-yloxy)octahydro-1*H*,5*H*-benzo[*d*]pyrrolo[1,2-*c*]imidazol-5-one 21**

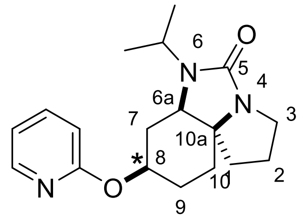

Major diastereomer, *dr* 93:7

To a mixture of the alcohol **6a** (28.0 mg, 0.12 mmol, 1.0 eq., obtained from LAH reduction of **4a**) and NaH (7.20 mg, 0.18 mmol, 1.5 eq.) in 4 mL THF, 2-fluoropyridine (0.02 mL, 0.18 mmol, 1.5 eq.) was added and the mixture was heated at 65 °C for 24 h. The reaction mixture was evaporated *in vacuo* and taken up in 1 mL H_2O. It was then diluted with 50 mL DCM, dried over $MgSO_4$, and evaporated *in vacuo*. Flash chromatography with 1 – 5% MeOH in DCM afforded the product as a diastereomeric mixture in the ratio of 93:7 (12 mg, 0.04 mmol, 32% yield). **R_f** = 0.41 (5% MeOH in DCM). **^{1}H NMR** (Major diastereomer, 500 MHz, $CDCl_3$): δ ppm 8.05 (1H, ddd, *J* = 5.1, 1.9, 0.6, Ar*H*), 7.49 (1H, ddd, *J* = 8.4, 7.1, 2.0, Ar*H*), 6.78 (1H, ddd, 7.0, 5.1, 0.9, Ar*H*), 6.62 (1H, dt, *J* = 8.5, 0.8, Ar*H*), 5.00 (1H, ddt, *J* =

11.4, 9.6, 4.2, 8-*H*), 3.99 (1H, hept, *J* = 7.0, isopropyl C*H*), 3.81 (1H, ddd, *J* = 12.5, 7.0, 3.0, 3-H_A), 3.55 (1H, dd, *J* = 10.2, 6.4, 6a-*H*), 2.89 (1H, ddd, *J* =12.3, 9.3, 5.8, 3-H_B), 2.48 (1H, dddd, *J* = 12.6, 6.4, 4.5, 1.8, 7-H_A), 1.93 – 1.85 (2H, m, 9-H_A, 10-H_A), 1.82 – 1.58 (4H, m, 2-$H_{A,B}$; 7-H_B; 10-H_B), 1.51 – 1.38 (3H, m, 1-$H_{A,B}$; 9-H_B), 1.13 (3H, d, *J* = 7.0, isopropyl CH_{3A}), 1.12 (3H, d, *J* = 6.5, isopropyl CH_{3B}). Signals for minor isomer visible at: 3.70 (0.07H, dd, *J* = 12.0, 4.5, 6a-*H*), 2.99 (0.08H, ddd, *J* = 12.3, 8.8, 6.1, 3-*H*). **^{13}C NMR** (Major diastereomer, 125 MHz, $CDCl_3$): δ ppm 162.5 (5-*C*), 162.0 (*Ar*), 145.7 (*Ar*H), 137.7 (*Ar*H), 115.6 (*Ar*H) 110.8 (*Ar*H), 68.7 (8-*C*), 64.6 (10a-*C*), 53.5 (6a-*C*), 43.2 (isopropyl *C*H), 43.0 (3-*C*), 36.8 (7-*C*), 33.8 (1-*C*), 27.9 (9-*C*), 26.0 (10-*C*), 21.9 (2-*C*), 21.4 (isopropyl CH_{3A}), 18.7 (isopropyl CH_{3B}). Signals for minor isomer visible at: 129.2, 126.4, 116.0, 110.4, 66.9, 63.9, 63.4, 53.1, 43.4, 21.4, 18.5. **IR** ν_{max} (neat)/cm^{-1}: 2964 (C-H), 1692 (C=O), 1594, 1569, 1470 (C=C), 1286 (C-O). **HRMS** (ESI): $C_{18}H_{26}N_3O_2$ [M + H$^+$]: calculated 316.2020, found 316.2020.

(6a*R,8*S**,8a*R**,9a*S**, 9b*S**)-8-Hydroxy-6-isopropyl-8-(thiophen-2-yl)octahydro-1*H*-cyclopropa[5,6]benzo[1,2-*d*]pyrrolo[1,2-*d*]imidazol-5(6*H*)-one 22**

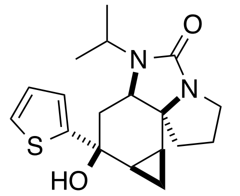

*(Stereochemical assignment: by analogy with **5a**)*

To a mixture of the cyclopropane scaffold **10a** (20.0 mg, 0.08 mmol, 1.0 eq.) and $CeCl_3$ (39.7 mg, 0.16 mmol, 2.0 eq.) in 4 mL of anhydrous THF at -78 °C, 1M 2-thienylmagnesium bromide in THF (0.16 mL, 0.16 mmol, 2.0 eq.) was added and the mixture was stirred at the same temperature for 1 h. The reaction mixture was then allowed to warm up to room temperature overnight and it was quenched with 1 mL H_2O. It was evaporated *in vacuo*, taken up in 1 mL H_2O, diluted with 100 mL EtOAc, dried over Na_2SO_4 and evaporated *in vacuo*. Flash chromatography eluting with 0.5 – 3% MeOH in DCM afforded the product as a 92:8 mixture of diastereomers (13.0 mg, 0.04 mmol, 49% yield). **^{1}H NMR** (500 MHz, $CDCl_3$): 7.25 (1H, dd, *J* = 5.1, 1.1, Ar*H*), 7.09 (1H, dd, *J* = 3.5, 1.2, Ar*H*), 6.95 (1H, dd, *J* = 5.1, 3.6, Ar*H*), 3.82 (1H, hept, *J* = 7.0, isopropyl C*H*), 3.73 (1H, dt, *J* = 12.1, 7.7, 3-H_A), 3.03 – 2.95 (2H, m, 3-H_B, 6a-*H*), 2.22 (1H, s, 8-CHO*H*), 2.12 (1H, ddd, *J* = 13.0, 5.2, 1.4, 7-H_A), 1.89 – 1.81 (2H, m, 2-$H_{A,B}$), 1.80 – 1.71 (2H, m, 1-H_A, 8a-*H*), 1.63 (1H, t, *J* = 13.0, 7-H_B), 1.28 (1H, q, *J* = 10.5, 1-H_B), 1.22 – 1.19 (1H, m, 9a-*H*), 1.08 (3H, d, *J* = 6.8, isopropyl CH_{3A}), 0.94 (3H, d, *J* = 6.9, isopropyl CH_{3B}), 0.89 (1H, q, *J* = 5.7, 9-H_A), 0.74 (1H, td, *J* = 8.8, 6.0, 9-H_B). Signal for minor isomer visible at: 4.07 (0.09H, br.d, *J* = 3.2, 6a-*H*). **^{13}C NMR** (125 MHz, $CDCl_3$): δ ppm 161.7 (5-*C*), 150.3 (*Ar*), 125.7 (*Ar*H), 124.1 (*Ar*H), 122.7 (*Ar*H), 70.7 (8-*C*), 63.0 (9b-*C*), 53.9 (6a-*C*), 43.2 (isopropyl *C*H), 42.6 (3-*C*), 39.4 (7-*C*), 35.8 (1-*C*), 23.4 (8a-*C*), 21.8 (2-*C*), 21.3 (isopropyl CH_{3A}), 19.7 (9a-*C*), 18.3 (isopropyl CH_{3B}), 5.9 (9-*C*). Signals for minor isomer

visible at: 126.5, 123.3, 122.4, 55.3, 43.5, 36.8, 23.1, 21.1, 18.6, 18.5, 16.6. **IR** ν_{max} (neat)/cm^{-1}: 3355 (O-H), 2967, 2934 (C-H), 1673 (C=O), 1458, 1420, 1365 (C=C), 1232 (C-O). **HRMS** (ESI): $C_{18}H_{25}N_2O_2S$ [M + H$^+$]: calculated 333.1631, found 333.1624.

(6a*R,8*R**,10a*S**)-8-(Cyclopropylamino)-6-tosyloctahydro-1*H*,5*H*-benzo[*d*]pyrrolo[1,2-*c*]imidazol-5-one 27**

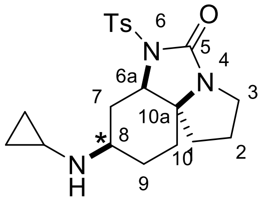

Major diastereomer, *dr* 87:13

*(Assignment of stereochemistry: by analogy with **7a**)*

To a solution of the ketone **4c** (24.0 mg, 0.07 mmol, 1.0 eq.) in 4 mL THF, cyclopropylamine (24.0 µL, 0.34 mmol, 5.0 eq.) and titanium isopropoxide (0.04 mL, 0.14 mmol, 2.0 eq.) were added and the mixture was left to stir at room temperature overnight. $NaBH_4$ (3.90 mg, 0.10 mmol, 1.5 eq.) was then added to the reaction mixture at -78 °C and it was stirred at the same temperature for 30 min. It was then allowed to warm up to room temperature for another 30 min. The reaction mixture was evaporated *in* vacuo. It was then taken up in 1 mL H_2O, diluted with 50 mL DCM, dried over Na_2SO_4 and evaporated *in vacuo*. Flash chromatography with 1 – 4% MeOH in DCM afforded the product as a diastereomeric mixture in the ratio of 87:13 (12.0 mg, 0.03 mmol, 45% yield). $\mathbf{R_f}$ = 0.41 (6% MeOH in DCM). **^{1}H NMR** (Major diastereomer, 500 MHz, $CDCl_3$): δ ppm 7.86 (2H, d, *J* = 8.5, Ar*H*), 7.24 (2H, d, *J* = 9.0, Ar*H*), 4.19 (1H, dd, *J* = 10.3, 6.4, 6a-*H*), 3.70 (1H, ddd, *J* = 12.5, 8.8, 6.0, 3-H_A), 2.91 – 2.73 (3H, m, 3-H_B, 7-H_A, 8-*H*), 2.35 (3H, s, ArCH_3), 2.15 (1H, quint, *J* = 5.0, cyclopropyl C*H*), 1.87 – 1.69 (5H, m, 2-$H_{A,B}$; 9-H_A; 10-$H_{A,B}$), 1.52 (1H, ddd, *J* = 12.0, 7.2, 2.6, 1-H_A), 1.38 – 1.29 (3H, m, 1-H_B, 7-H_B, 9-H_B), 0.65 – 0.40 (4H, m, cyclopropyl $CH_{2A,B,C,D}$). Signals for minor isomer visible at: 7.91 (0.32H, d, *J* = 8.5, Ar*H*), 4.36 (0.15H, t, 5.0, 6a-*H*). **^{13}C NMR** (Major diastereomer, 125 MHz, $CDCl_3$): δ ppm 157.4 (5-*C*), 143.8 (*Ar*), 135.2 (*Ar*), 128.5 (*Ar*H), 127.2 (*Ar*H), 64.4 (10a-*C*), 56.6 (6a-*C*), 52.5 (8-*C*), 42.9 (3-*C*), 35.7 (7-*C*), 33.4 (1-*C*), 27.9 (9-*C*), 26.8 (cyclopropyl *C*H), 24.9 (10-*C*), 21.7 (2-*C*), 20.6 (Ar*C*H_3), 5.0 (cyclopropyl $CH_{2A,B}$), 4.6 (cyclopropyl $CH_{2C,D}$). Signals for minor isomer visible at: 64.0, 57.3, 52.4, 42.6, 34.6, 29.9, 28.6, 27.7. **IR** ν_{max} (neat)/cm^{-1}: 3274 (N-H), 2939, 2868 (C-H), 1726 (C=O), 1597, 1494, 1446 (C=C). **HRMS** (ESI): $C_{20}H_{28}N_3O_3S$ [M + H$^+$]: calculated 390.1846, found 390.1852.

(6a*R,8*S**,8a*R**,9a*S**, 9b*S**)-6-(4-Methoxyphenyl)-8-(methylamino)octahydro-1*H*-cyclopropa[5,6]benzo[1,2-*d*]pyrrolo[1,2-*c*]imidazol-5(6*H*)-one 29**

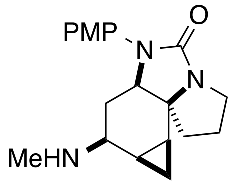

*(Assignment of stereochemistry: analysis of J values on derivative **45**)*

To a solution of the cyclopropane scaffold **10b** (9.00 mg, 0.03 mmol, 1.0 eq.) in 4 mL THF, 2M methylamine in MeOH (0.15 mL, 0.29 mmol, 10.0 eq.) and titanium isopropoxide (0.02 mL, 0.06 mmol, 2.0 eq.) were added and the mixture was left to stir at room temperature overnight. $NaBH_4$ (1.60 mg, 0.04 mmol, 1.5 eq.) was added to the reaction mixture at -78 °C and it was stirred at the same temperature for 30 min. It was then allowed to warm up to room temperature for another 30 min. The reaction mixture was evaporated *in* vacuo. It was then taken up in 1 mL H_2O, diluted with 50 mL EtOAc, dried over Na_2SO_4 and evaporated *in vacuo*. Flash chromatography with 2 - 3% of saturated NH_3/MeOH in DCM afforded the product **29** as a 93:7 mixture of diastereomers (5.3 mg, 0.020 mmol, 56% yield). **^{1}H NMR** (**Major diastereomer**, 500 MHz, $CDCl_3$): δ ppm 7.21 (2H, d, *J* = 9.0, Ar*H*), 6.79 (2H, d, *J* = 9.0, Ar*H*), 3.85 – 3.79 (1H, m, 3-H_A), 3.77 (1H, dd, *J* = 12.5, 5.5, 6a-*H*), 3.71 (3H, s, ArOCH_3), 3.12 -3.04 (2H, m, 3-H_B, 8-*H*), 2.40 (3H, s, NHCH_3), 1.97 -1.90 (3H, m, 2-$H_{A,B}$; 7-H_A), 1.89 – 1.84 (1H, m, 1-H_A), 1.63 (1H, q, *J* = 11.0, 1-H_B), 1.52 - 1.44 (1H, m, 8a-*H*), 0.99 – 0.88 (2H, m, 7-H_B, 9a-*H*), 0.71 (1H, q, *J* = 5.5, 9-H_A), 0.54 (1H, td, *J* = 8.7, 5.8, 9-H_B). Signal for minor isomer visible at: 3.38 (0.07H, dt, *J* = 12.7, 3.7, 3-*H*). **^{13}C NMR** (125 MHz, $CDCl_3$): δ ppm 160.2 (5-*C*), 155.4 (*Ar*), 130.1 (*Ar*), 122.6 (*Ar*H), 113.3 (*Ar*H), 63.0 (9b-*C*), 57.4 (6a-*C*), 54.5 (ArOCH$_3$), 51.6 (8-*C*), 42.8 (3-*C*), 36.4 (1-*C*), 31.7 (NH*C*H$_3$), 26.8 (7-*C*), 21.8 (2-*C*), 16.8 (9a-*C*), 16.2 (8a-*C*), 3.5 (9-*C*). Signals for minor isomer visible at: 63.5, 62.4, 58.0, 57.6, 51.0, 46.6, 24.6, 15.7, 14.4. **IR** ν_{max} (neat)/cm^{-1}: 3288 (N-H), 2953, 2790 (C-H), 1693 (C=O), 1512, 1460, 1427 (C=C), 1246 (C-O). **HRMS** (ESI): $C_{19}H_{26}N_3O_2$ [M + H$^+$]: calculated 328.2020, found 328.2016.

(6a*R,8*R**,10a*S**)-6-Isopropyl-5-oxooctahydro-1*H*,5*H*-benzo[*d*]pyrrolo[1,2-*c*]imidazol-8-yl (2-fluorophenyl)carbamate 31**

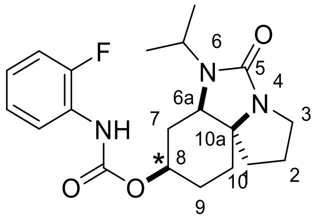

Major diastereomer, *dr* 88:12

To a solution of the alcohol **6a** (14.0 mg, 0.06 mmol, 1.0 eq., obtained from LAH reduction of **4a**) in 5 mL DCM, 2-fluorophenylisocyanate (0.01 mL, 0.07 mmol, 1.5 eq.) and TEA (0.03 mL, 0.18 mmol, 3.0

eq.) were added and the mixture was stirred at room temperature overnight. The reaction mixture was evaporated *in vacuo*. Flash chromatography with 10 – 90% EtOAc in hexane afforded the product as a diastereomeric mixture in the ratio of 88:12 (17 mg, 0.05 mmol, 77% yield). **R_f** = 0.47 (80% EtOAc in hexane). **^{1}H NMR** (Major diastereomer, 500 MHz, $CDCl_3$): δ ppm 8.00 (1H, br.s, Ar*H*), 7.06 (1H, t, *J* = 7.8, Ar*H*), 7.03 – 6.98 (1H, m, Ar*H*), 6.96 – 6.91 (1H, m, Ar*H*), 6.76 (1H, s, ArN*H*), 4.70 (1H, tt, *J* = 11.0, 4.0, 8-*H*), 4.00 (1H, hept, *J* = 7.0, isopropyl C*H*), 3.81 (1H, ddd, *J* = 12.4, 9.2, 5.2, 3-H_A), 3.51 (1H, dd, *J* = 10.0, 6.5, 6a-*H*), 2.88 (1H, ddd, *J* =12.3, 9.4, 5.8, 3-H_B), 2.42 (1H, dddd, *J* = 10.5, 6.0, 4.5, 2.0, 7-H_A), 1.90 (1H, dt, *J* = 15.0, 4.5, 10-H_A), 1.86 – 1.71 (3H, m, 2-$H_{A,B}$; 9-H_A), 1.66 – 1.61 (1H, m, 9-H_B), 1.56 – 1.32 (4H, m, 1-$H_{A,B}$; 7-H_B, 10-H_B), 1.15 (3H, d, *J* = 7.0, isopropyl CH_{3A}), 1.12 (3H, d, *J* = 7.0, isopropyl CH_{3B}). Signals for minor isomer visible at: 5.07 (0.14H, quint, *J* = 5.0, 8-*H*), 3.70 (0.14H, dd, *J* = 8.5, 6.0, 6a-*H*), 2.19 (0.13H, dt, *J* = 10.7, 5.2, 10-*H*). **^{13}C NMR** (Major diastereomer, 125 MHz, $CDCl_3$): δ ppm 162.4 (5-*C*), 151.6 (carbamate *C*=O), 151.1 (d, *J* = 241, *Ar*F), 125.3 (d, *J* =10.0, *Ar*), 123.6 (d, *J* = 3.63, *Ar*H), 122.5 (d, *J* = 7.25, *Ar*H), 119.1 (*Ar*H), 113.9 (d, *J* = 19.0, *Ar*H), 69.8 (8-*C*), 64.4 (10a-*C*), 53.2 (6a-*C*), 43.3 (isopropyl *C*H), 43.0 (3-*C*), 36.9 (7-*C*), 33.6 (1-*C*), 27.7 (10-*C*), 26.0 (9-*C*), 21.9 (2-*C*), 21.4 (isopropyl CH_{3A}), 18.7 (isopropyl CH_{3B}). Signals for minor isomer visible at: 68.4, 52.0, 34.3, 33.7, 25.0, 24.0, 22.1, 21.2, 18.6. **IR** ν_{max} (neat)/cm^{-1}: 3246 (N-H); 2967 (C-H); 1724, 1682 (C=O); 1620, 1597, 1538 (C=C); 1258 (C-O). **HRMS** (ESI): $C_{20}H_{27}FN_3O_3$ [M + H$^+$]: calculated 376.2031, found 376.2027.

(6a*R,8*R**,10a*S**)-6-Isopropyl-5-oxooctahydro-1*H*,5*H*-benzo[*d*]pyrrolo[1,2-*c*]imidazol-8-yl)-*N*-methylpyridine-3-sulfonamide 32**

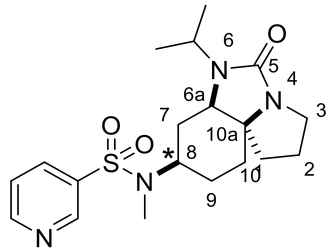

(Assignment of stereochemistry: X-ray crystal structure - CCDC deposition number: 2175096)

To a solution of the ketone **4a** (27.0 mg, 0.11 mmol, 1.0 eq.) in 5 mL THF, 2M methylamine in MeOH (0.55 mL, 1.10 mmol, 10.0 eq.) and titanium isopropoxide (0.07 mL, 0.22 mmol, 2.0 eq.) were added and the mixture was left to stir at room temperature overnight. $NaBH_4$ (6.40 mg, 0.17 mmol, 1.5 eq.) was added to the reaction mixture at -78 °C and it was stirred at the same temperature for 30 min. It was then allowed to warm up to room temperature for another 30 min. The reaction mixture was evaporated *in* vacuo. It was then taken up in 1 mL H_2O, diluted with 100 mL EtOAc, dried over Na_2SO_4 and evaporated *in vacuo*. To a solution of the crude and DMAP (1.00 mg, 2.75 µmol, 2.5 mol%) in 6 mL DCM, pyridine-3-sulfonyl chloride (14.0 µL, 0.12 mmol, 1.1 eq.) and TEA (23.0 µL, 0.17 mmol, 1.5 eq.) were added. The reaction mixture was allowed to stir at room temperature for 12 h after which

1 mL of saturated aqueous Na_2CO_3 was added. It was then diluted with 100 mL EtOAc, dried over Na_2SO_4 and evaporated *in vacuo*. Flash chromatography with 1 - 3% MeOH in DCM afforded the product **32** as a single diastereomer (30.0 mg, 0.08 mmol, 67% yield). $\mathbf{R_f}$ = 0.30 (5% MeOH in DCM). **^{1}H NMR** (500 MHz, $CDCl_3$): δ ppm 9.03 (1H, dd, *J* = 2.3, 0.6, Ar*H*), 8.80 (1H, dd, *J* = 4.8, 1.6, Ar*H*), 8.09 (1H, ddd, *J* = 8.0, 2.3, 1.7, Ar*H*), 7.47 (1H, ddd, *J* = 8.0, 4.8, 0.7, Ar*H*), 4.03 (1H, hept, *J* = 6.9, isopropyl C*H*), 3.89 (1H, tt, *J* = 12.0, 3.5, 8-*H*), 3.83 (1H, ddd, *J* = 12.5, 9.4, 5.0, 3-H_A), 3.55 (1H, dd, *J* = 9.9, 6.7, 6a-*H*), 2.84 (1H, ddd, *J* = 12.4, 9.5, 5.9, 3-H_B), 2.74 (3H, s, NCH_3), 2.00 (1H, dddd, *J* =12.5, 6.3, 3.5, 2.4, 7-H_A), 1.89 (1H, dt, *J* = 14.7, 3.8, 10-H_A), 1.85 – 1.66 (2H, m, 2-$H_{A,B}$), 1.60 – 1.49 (2H, m, 7-H_B, 9-H_A), 1.48 – 1.42 (2H, m, 1-$H_{A,B}$), 1.37 (1H, ddd, *J* = 14.7, 12.6, 3.9, 10-H_B), 1.29 - 1.21 (1H, m, 9-H_B), 1.13 (3H, d, *J* = 6.9, isopropyl CH_{3A}), 1.10 (3H, d, *J* = 6.8, isopropyl CH_{3B}). **^{13}C NMR** (125 MHz, $CDCl_3$): δ ppm 163.4 (5-*C*), 153.2 (*Ar*H), 147.8 (*Ar*H), 136.5 (*Ar*), 134.5 (*Ar*H), 123.8 (*Ar*H), 65.2 (10a-*C*), 54.4 (6a-*C*), 53.2 (8-*C*), 44.2 (isopropyl *C*H), 44.0 (3-*C*), 37.0 (7-*C*), 34.4 (1-*C*), 30.1 (10-*C*), 28.6 (N*C*H_3), 24.8 (9-*C*), 22.8 (2-*C*), 22.4 (isopropyl CH_{3B}), 19.8 (isopropyl CH_{3A}). **IR** ν_{max} (neat)/cm^{-1}: 3054, 2966 (C-H), 1686 (C=O), 1572, 1463, 1438 (C=C). **HRMS** (ESI): $C_{19}H_{29}N_4O_3S$ [M + H$^+$]: calculated 393.1955, found 393.1952.

(6a*R,8*R**,10a*S**)-8-(((1-*H*-Imidazol-4-yl)methyl)amino)-6-isopropyloctahydro-1*H*,5*H*-benzo[*d*]pyrrolo[1,2-*c*]imidazol-5-one 33**

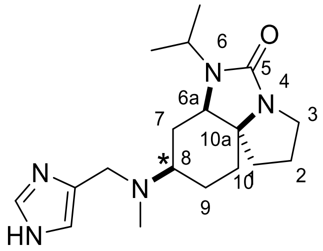

To a solution of the ketone **4a** (28.0 mg, 0.12 mmol, 1.0 eq.) in 5 mL THF, 2M methylamine in MeOH (0.60 mL, 1.20 mmol, 10.0 eq.) and titanium isopropoxide (0.07 mL, 0.24 mmol, 2.0 eq.) were added and the mixture was left to stir at room temperature overnight. $NaBH_4$ (6.80 mg, 0.18 mmol, 1.5 eq.) was added to the reaction mixture at -78 °C and it was stirred at the same temperature for 30 min. It was then allowed to warm up to room temperature for another 30 min. The reaction mixture was evaporated *in* vacuo. It was then taken up in 1 mL H_2O, diluted with 100 mL EtOAc, dried over Na_2SO_4 and evaporated *in vacuo*. To a mixture of the crude, 4-imidazolecarboxaldehyde (17.3 mg, 0.18 mmol, 1.5 eq.) and sodium triacetoxyborohydride (38.1 mg, 0.18 mmol, 1.5 eq.) in 5 mL DCM; 14.0 µL of acetic acid was added. The reaction mixture was allowed to stir at room temperature for 3 days after which 2 mL of saturated aqueous Na_2CO_3 was added. It was diluted with 100 mL DCM, dried over Na_2SO_4 and evaporated *in vacuo*. Flash chromatography with 5% MeOH in DCM followed by 2 - 4% of saturated NH_3/MeOH in DCM afforded the product **33** as a single diastereomer but an 80:20

mixture of tautomers (8.00 mg, 0.03 mmol, 15% yield). **R_f** = 0.57 (8% of saturated NH_3/MeOH in DCM). **^{1}H NMR** (400 MHz, $CDCl_3$): δ ppm 7.53 (0.75H, s, Ar*H*), 7.51 (0.20H, s, Ar*H*), 6.85 (0.72H, s, Ar*H*), 6.83 (0.20H, s, Ar*H*), 3.99 (0.78H, hept, *J* = 6.8, isopropyl C*H*), 3.90 (0.20H, hept, *J* = 6.8, isopropyl C*H*), 3.77 (0.78H, ddd, *J* = 12.4, 7.2, 3.2, 3-H_A), 3.69 (0.15H, ddd, *J* = 12.4, 6.0, 3.6, 3-H_A), 3.65 (0.28H, m, 6a-*H*), 3.55 (1.44H, s, ArC$H_{2A,B}$), 3.52 (0.41H, s, ArC$H_{2A,B}$), 3.41 (0.86H, dd, *J* = 10.0, 6.4, 6a-*H*), 2.86 (1.21H, ddd, *J* = 12.4, 6.4, 3.2, 3-H_B), 2.48 (0.77H, t, *J* =11.2, 8-*H*), 2.20 – 2.04 (3.87H, s, NCH_3, 7-H_A), 1.88 (0.96H, dt, *J* = 14.4, 4,1, 10-H_A), 1.82 – 1.30 (8.09H, m, 1-$H_{A,B}$; 2-$H_{A,B}$; 7-H_B; 9-$H_{A,B}$; ArN*H*), 1.29 – 1.18 (1.23H, m, 10-H_B), 1.15 (3.21H, d, *J* = 6.8, isopropyl CH_{3A}), 1.12 (2.73H, d, *J* = 6.8, isopropyl CH_{3B}). **^{13}C NMR** (Major tautomer, 100 MHz, $CDCl_3$): δ ppm 163.6 (5-*C*), 153.6 (*Ar*), 152.7 (*Ar*), 134.9 (*Ar*H), 66.0 (10a-*C*), 57.7 (8-*C*), 55.5 (6a-*C*), 49.9 (Ar*C*H_2, **missing – found by HSQC**), 44.2 (isopropyl *C*H), 44.0 (3-*C*), 36.9 (NCH_3), 35.0 (7-*C*), 34.7 (9-*C*), 30.5 (10-*C*), 23.0 (1-*C*), 22.6 (isopropyl *C*H_{3A}), 22.4 (2-*C*), 19.8 (isopropyl *C*H_{3B}). Signals for minor tautomer visible at: 56.6, 44.4, 36.6, 27.2, 23.3, 22.1, 21.6, 19.5. **IR** ν_{max} (neat)/cm^{-1}: 3049, 2967, 2937, 2794 (C-H), 1679 (C=O), 1459, 1412 (C=C). **HRMS** (ESI): $C_{18}H_{30}N_5O$ [M + H^+]: calculated 332.2445, found 332.2437.

(6a*R,8*R**,10a*S**)-6-Isopropyl-5-oxooctahydro-1*H*,5*H*-benzo[*d*]pyrrolo[1,2-*c*]imidazol-8-yl)-*N*-methylisoxazole-5-carboxamide 34** and **(6a*R*,8*S*,10a*S*)-6-isopropyl-5-oxooctahydro-1*H*,5*H*-benzo[*d*]pyrrolo[1,2-*c*]imidazol-8-yl)-*N*-methylisoxazole-5-carboxamide 35**

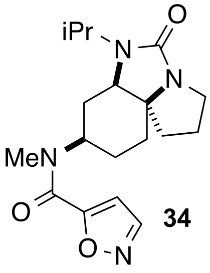

34

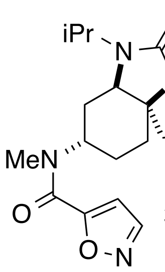

35

To a solution of the ketone **4a** (30.0 mg, 0.13 mmol, 1.0 eq.) in 5 mL THF, 33 wt% methylamine in EtOH (0.16 mL, 1.30 mmol, 10.0 eq.) and titanium isopropoxide (0.08 mL, 0.26 mmol, 2.0 eq.) were added and the mixture was left to stir at room temperature overnight. $NaBH_4$ (7.40 mg, 0.20 mmol, 1.5 eq.) was added to the reaction mixture at -78 °C and it was further stirred at the same temperature for 30 min. It was then allowed to warm up to room temperature for another 30 min. The reaction mixture was evaporated *in vacuo*. It was then taken up in 1 mL H_2O, diluted with 100 mL EtOAc, dried over Na_2SO_4 and evaporated *in vacuo*. To a solution of the crude and DMAP (1.00 mg, 8.19 µmol, 6.3 mol%) in 6 mL DCM, isoxazole-5-carbonyl chloride (14.0 µL, 0.14 mmol, 1.1 eq.) and TEA (27.0 µL, 0.20 mmol, 1.5 eq.) were added. The reaction mixture was allowed to stir at room temperature overnight after which 2 mL of saturated aqueous Na_2CO_3 was added. It was diluted with 100 mL DCM, dried over Na_2SO_4 and evaporated *in vacuo*. Flash chromatography with 1 - 2% MeOH

in DCM afforded the products **34** [Major diastereomer, 22.0 mg, 0.06 mmol, 50% yield, 63:37 mixture of rotamers, **R_f** = 0.37 (5% MeOH in DCM)] and **35** [Minor diastereomer, 4.00 mg, 0.01 mmol, 9% yield, 50:50 mixture of rotamers, **R_f** = 0.41 (5% MeOH in DCM)]. **^{1}H NMR** (**Major diastereomer 34**, 400 MHz, $CDCl_3$): δ ppm 8.28 (0.3H, d, *J* = 1.7, Ar*H*), 8.25 (0.6H, d, *J* = 1.6, Ar*H*), 6.74 (0.3H, d, *J* = 1.6, Ar*H*), 6.71 (0.6H, d, *J* = 1.7, Ar*H*), 4.48 (0.6H, tt, 12.4, 3.2, 8-*H*), 4.02 (1.0H, m, isopropyl C*H*), 3.82 (1.4H, m, 3-H_A), 3.60 (0.6H, dd, *J* = 9.7, 6.8, 6a-*H*), 3.51 (0.3H, dd, *J* = 9.7, 6.7, 6a-*H*), 2.98 (1.9H, s, NCH_3), 2.91 (1.0H, s, NCH_3), 2.85 (1.1H, ddd, *J* = 15.0, 7.5, 4.0, 3-H_B), 2.26 – 2.18 (0.4H, m, 7-H_A), 2.18 – 2.10 (0.6H, m, 7-H_A), 1.95 (1.0H, tt, *J* = 14.5, 3.6, 2-H_A), 1.87 – 1.28 (8.0H, m, 1-$H_{A,B}$; 2-H_B; 7-H_B; 9-$H_{A,B}$; 10-$H_{A,B}$), 1.15 (2.9H, d, *J* = 6.8, isopropyl CH_{3A}), 1.12 (3.0H, d, *J* = 6.9, isopropyl CH_{3A}). **^{13}C NMR** (**Major diastereomer 34**, 100 MHz, $CDCl_3$): δ ppm 164.1 (amide *C*=O), 163.6 (5-*C*), 158.3/158.1 (*Ar*), 150.3/150.1 (*Ar*H), 107.7/107.4 (*Ar*H), 65.5/65.3 (10a-*C*), 54.4/54.0 (6a-*C*), 50.2 (8-*C*), 44.3 (isopropyl *C*H), 44.1 (3-*C*), 37.6/35.9 (7-*C*), 34.5/34.4 (1-*C*), 30.6/28.2 (NCH_3), 30.1 (10-*C*), 26.1 (9-*C*), 24.6/22.9 (2-*C*), 22.6/22.5 (isopropyl CH_{3A}), 19.9 (isopropyl CH_{3B}). **IR** ν_{max} (neat)/cm^{-1}: 3104, 2965, 2935 (C-H), 1692, 1643 (C=O), 1576, 1512 (C=C), 1283 (C-O). **HRMS** (ESI): $C_{18}H_{27}N_4O_3$ [M + H$^+$]: calculated 347.2078, found 347.2072. **^{1}H NMR** (**Minor diastereomer 35**, 500 MHz, $CDCl_3$): δ ppm 8.23 (1.0H, s, Ar*H*), 6.71 (0.5H, s, Ar*H*), 6.54 (0.5H, s, Ar*H*), 4.46 (0.5H, s, 8-*H*), 4.23 (0.5H, s, 8-*H*), 3.94 (0.5H, hept, *J* = 6.6, isopropyl C*H*), 3.80 (0.5H, hept, *J* = 6.7, isopropyl C*H*), 3.74 (0.5H, s, 6a-*H*), 3.66 (1.4H, s, 6a-*H*, 3-H_A), 3.07 (1.5H, s, NCH_3), 2.93 (1.4H, s, NCH_3), 2.88 (0.9H, m, 3-H_B), 2.20 – 1.91 (2.2H, m, 2-H_A, 7-H_A), 1.85 – 1.55 (7.1H, m, 1-H_A; 2-H_B; 7-H_B; 9-$H_{A,B}$; 10-$H_{A,B}$), 1.46 – 1.30 (1.2H, m, 1-H_B), 1.21 (1.5H, d, *J* = 7.0, isopropyl CH_{3A}), 1.18 (1.5H, d, *J* = 7.0, isopropyl CH_{3B}), 1.07 (1.5H, d, *J* = 7.0, isopropyl CH_{3B}), 1.01 (1.5H, d, *J* = 7.0, isopropyl CH_{3A}). **^{13}C NMR** (**Minor diastereomer 35**, 125 MHz, $CDCl_3$): δ ppm 164.5 (amide *C*=O), 163.0/162.9 (5-*C*), 158.9/158.0 (*Ar*), 150.1/149.9 (*Ar*H), 107.5/105.6 (*Ar*H), 64.8/64.6 (10a-*C*), 57.5/57.1 (6a-*C*), 48.8/48.3 (8-*C*), 44.5 (isopropyl *C*H), 44.1 (3-*C*), 37.4/37.2 (1-*C*), 33.4/28.3 (NCH_3), 30.3/28.5 (7-*C*), 27.7/27.5 (10-*C*), 23.6/23.5 (9-*C*), 22.9 (2-*C*), 21.9/21.4 (isopropyl CH_{3A}), 19.3 (isopropyl CH_{3B}). **IR** ν_{max} (neat)/cm^{-1}: 3100, 2927 (C-H), 1686, 1642 (C=O), 1576, 1460 (C=C), 1245 (C-O). **HRMS** (ESI): $C_{18}H_{27}N_4O_3$ [M + H$^+$]: calculated 347.2078, found 347.2072.

(6a*R,8*R**,10a*S**)-8-[(Furan-2-ylmethyl)amino]-6-isopropyloctahydro-1*H*,5*H*-benzo[*d*]pyrrolo[1,2-*c*]imidazol-5-one 36**

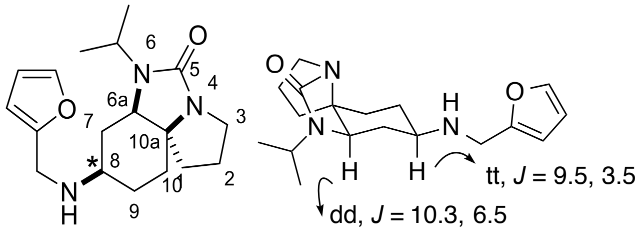

(Assignment of stereochemistry: J value analysis)

To a solution of the ketone **4a** (39.0 mg, 0.17 mmol, 1.0 eq.) in 4 mL THF, furfurylamine (17.0 µL, 0.20 mmol, 1.2 eq.) and titanium isopropoxide (0.10 mL, 0.33 mmol, 2.0 eq.) were added and the mixture was left to stir at room temperature overnight. $NaBH_4$ (9.4 mg, 0.25 mmol, 1.5 eq.) was added to the reaction mixture at -78 °C and it was stirred at the same temperature for 30 min. It was then allowed to warm up to room temperature for another 30 min. The reaction mixture was evaporated *in vacuo* and dissolved in 1 mL DMSO. Reverse phase chromatography by the use of a biotage machine (MeCN/1% HCOOH in H_2O) afforded the product **36** as a single diastereomer (28 mg, 0.09 mmol, 53% yield). **R_f** = 0.36 (6% MeOH in DCM). **^{1}H NMR** (500 MHz, $CDCl_3$): δ ppm 7.30 (1H, dd, *J* = 1.8, 0.7, Ar*H*), 6.26 (1H, dd, *J* = 3.1, 1.9, Ar*H*), 6.16 (1H, d, *J* = 3.0, Ar*H*), 3.98 (1H, hept, *J* = 7.0, isopropyl C*H*), 3.88 – 3.69 (3H, m, 3-H_A, Ar$CH_{2A,B}$), 3.39 (1H, dd, *J* = 10.3, 6.5, 6a-*H*), 2.85 (1H, ddd, *J* = 12.3, 9.4, 5.8, 3-H_B), 2.49 (1H, tt *J* = 9.5, 3.5, 8-*H*), 2.28 – 2.21 (1H, m, 7-H_A), 1.88 – 1.83 (1H, m, 9-H_A), 1.75 – 1.67 (3H, m, 2-$H_{A,B}$; 10-H_A,), 1.42 – 1.36 (2H, m, 1-$H_{A,B}$), 1.31 – 1.18 (4H, m, 7-H_B, 9-H_B, 10-H_B, ArCH_2N*H*), 1.13 (3H, d, *J* = 7.0, isopropyl CH_{3A}), 1.10 (3H, d, *J* = 7.0, isopropyl CH_{3B}). **^{13}C NMR** (125 MHz, $CDCl_3$): δ ppm 162.5 (5-*C*), 152.1 (*Ar*), 141.0 (*Ar*H), 109.3 (*Ar*H), 106.4 (*Ar*H), 64.8 (10a-*C*), 53.6 (6a-*C*), 51.4 (8-*C*), 43.2 (isopropyl *C*H), 43.0 (3-*C*), 42.0 (ArCH_2), 38.4 (7-*C*), 33.7 (1-*C*), 28.7 (9-*C*), 26.5 (10-*C*), 21.9 (2-*C*), 21.5 (isopropyl CH_{3A}), 18.7 (isopropyl CH_{3B}). **IR** ν_{max} (neat)/cm^{-1}: 3304 (N-H); 2933 (C-H); 1687 (C=O); 1512, 1460 (C=C); 1246 (C-O). **HRMS** (ESI): $C_{18}H_{28}N_3O_2$ [M + H$^+$]: calculated 318.2176, found 318.2177.

(6a*R,8*R**,10a*S**)-8-(Cyclopropylamino)-6-isopropyloctahydro-1*H*,5*H*-benzo[*d*]pyrrolo[1,2-*c*]imidazol-5-one 37**

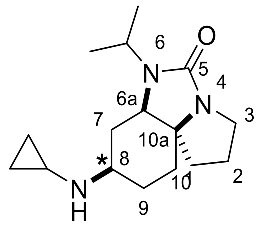

Major diastereomer, *dr* 79:21

*(Assignment of stereochemistry: by analogy with **7a**)*

To a solution of the ketone **4a** (18.0 mg, 0.08 mmol, 1.0 eq.) in 4 mL THF, cyclopropylamine (0.03 mL, 0.38 mmol, 5.0 eq.) and titanium isopropoxide (0.05 mL, 0.15 mmol, 2.0 eq.) were added and the mixture was left to stir at room temperature overnight. $NaBH_4$ (4.30 mg, 0.11 mmol, 1.5 eq.) was added to the reaction mixture at -78 °C and it was stirred at the same temperature for 30 min. It was then allowed to warm up to room temperature for another 30 min. The reaction mixture was evaporated *in* vacuo. It was then taken up in 1 mL H_2O, diluted with 50 mL DCM, dried over Na_2SO_4 and evaporated *in vacuo*. Flash chromatography with 1 – 3.5% MeOH in DCM afforded the product **37** as a diastereomeric mixture in the ratio of 79:21 (16.0 mg, 0.06 mmol, 76% yield). **R_f** = 0.47 (8%

MeOH in DCM). **^{1}H NMR** (Major diastereomer, 500 MHz, $CDCl_3$): δ ppm 3.99 (1H, hept, *J* = 7.0, isopropyl C*H*), 3.78 (1H, ddd, *J* = 12.4, 9.1, 5.2, 3-H_A), 3.43 (1H, dd, *J* = 10.2, 6.6, 6a-*H*), 2.85 (1H, ddd, *J* = 12.4, 9.3, 5.9, 3-H_B), 2.58 (1H, tt, *J* =11.0, 3.5, 8-*H*), 2.35 – 2.30 (1H, m, 7-H_A), 2.12 (1H, ddd, *J* = 10.2, 6.8, 3.7, cyclopropyl C*H*), 1.89 – 1.84 (1H, m, 10-H_A), 1.81 – 1.70 (3H, m, 2-$H_{A,B}$; 9-H_A), 1.43 – 1.38 (2H, m, 1-$H_{A,B}$), 1.26 – 1.17 (3H, m, 7-H_B, 9-H_B, 10-H_B), 1.14 (3H, d, *J* = 7.0, isopropyl CH_{3A}), 1.12 (3H, d, *J* = 7.0, isopropyl CH_{3B}), 0.44 – 0.27 (4H, m, cyclopropyl $CH_{2A,B}$; cyclopropyl $CH_{2C,D}$). Signals for minor isomer visible at: 3.70 (0.33H, ddd, *J* = 11.9, 8.8, 5.9, 3-H_A), 3.60 (0.27H, t, 5.2, 6a-*H*), 2.65 (0.27H, ddd, *J* = 14.0, 7.5, 4.0, 8-*H*). **^{13}C NMR** (Major diastereomer, 125 MHz, $CDCl_3$): δ ppm 162.6 (5-*C*), 64.9 (10a-*C*), 53.7 (6a-*C*), 52.8 (8-*C*), 43.2 (isopropyl *C*H), 43.0 (3-*C*), 39.0 (7-*C*), 33.8 (1-*C*), 28.9 (10-*C*), 27.1 (9-*C*), 26.7 (cyclopropyl *C*H), 21.9 (2-*C*), 21.5 (isopropyl CH_{3A}), 18.7 (isopropyl CH_{3B}), 5.5 (cyclopropyl $CH_{2A,B}$), 5.2 (cyclopropyl $CH_{2C,D}$). Signals for minor isomer visible at: 64.7, 57.8, 54.1, 48.9, 43.3, 43.0, 36.7, 35.0, 27.9, 25.4, 22.2, 21.2, 18.5, 4.6. **IR** ν_{max} (neat)/cm^{-1}: 3288 (O-H), 2969, 2934 (C-H), 1687 (C=O). **HRMS** (ESI): $C_{16}H_{28}N_3O$ [M + H$^+$]: calculated 278.2227, found 278.2224.

(6a*R,8*R**,10a*S**)-8-[(Furan-2-ylmethyl)amino]-6-(4-methoxyphenyl)octahydro-1*H*,5*H*-benzo[*d*]pyrrolo[1,2-*c*]imidazol-5-one 38**

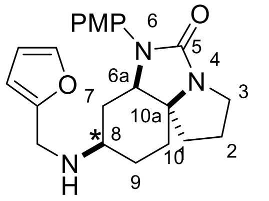

*(Assignment of stereochemistry: analogy with **7a**)*

To a solution of the ketone **4b** (40.0 mg, 0.13 mmol, 1.0 eq.) in 6 mL THF, 3-aminooxetane (14.1 µL, 0.16 mmol, 1.2 eq.) and titanium isopropoxide (0.08 mL, 0.27 mmol, 2.0 eq.) were added and the mixture was left to stir at room temperature overnight. $NaBH_4$ (6.05 mg, 0.16 mmol, 1.2 eq.) was added to the reaction mixture at -78 °C and it was stirred at the same temperature for 1 h. It was then allowed to warm up to room temperature overnight. The reaction mixture was evaporated *in vacuo*. It was then taken up in 2 mL H_2O, diluted with 100 mL DCM, dried over Na_2SO_4 and evaporated *in vacuo*. Flash chromatography with 1 - 3% MeOH in DCM afforded the product **38** as a single diastereomer (45.7 mg, 0.90 mmol, 90% yield). **R_f** = 0.42 (5% MeOH in DCM). **^{1}H NMR** (500 MHz, $CDCl_3$): δ ppm 7.27 (2H, d, *J* = 9.0, Ar*H*), 7.25 (1H, dd, *J* = 2.0, 0.5, Ar*H*), 6.80 (2H, d, *J* = 9.0, Ar*H*), 6.21 (1H, dd, *J* = 3.0, 2.0, Ar*H*), 6.04 (1H, dd, *J* = 3.0, 0.5, Ar*H*), 3.93 (1H, dd, *J* = 10.1, 6.2, 6a-*H*), 3.84 (1H, ddd, *J* = 12.3, 9.0, 5.5, 3-H_A), 3.72 (3H, s, ArOCH_3), 3.67 (2H, d, *J* = 1.5, ArC$H_{2A,B}$), 2.98 (1H, ddd, *J* = 12.3, 9.2, 5.8, 3-H_B), 2.51 (1H, qd, *J* = 8.6, 4.0, 8-*H*), 2.17 (1H, dddd, *J* = 12.5, 8.0, 6.0, 2.0, 7-H_A), 1.93 – 1.55 (6H, m, 1-$H_{A,B}$; 2-$H_{A,B}$; 9-H_A; 10-H_A), 1.37 – 1.25 (3H, m, 8-CHN*H*, 9-H_B; 10-H_B), 1.24 – 1.18 (1H, m,

7-H_B). **^{13}C NMR** (125 MHz, $CDCl_3$): δ ppm 162.0 (5-*C*), 156.3 (*Ar*) 153.8 (*Ar*), 141.8 (*Ar*H), 131.6 (*Ar*), 123.4 (*Ar*H), 114.3 (*Ar*H), 110.1 (*Ar*H), 106.7 (*Ar*H), 64.9 (10a-*C*), 58.4 (6a-*C*), 55.5 (ArO*C*H$_3$), 51.9 (8-*C*), 44.3 (3-*C*), 43.3 (Ar*C*H$_2$) 36.0 (7-*C*), 35.4 (1-*C*), 29.7 (10-*C*), 28.0 (9-*C*), 23.2 (2-*C*). **IR** ν_{max} (neat)/cm^{-1}: 3315 (N-H), 2925, 2854 (C-H), 1692 (C=O), 1511, 1462, 1394 (C=C), 1245 (C-O). **HRMS** (ESI): $C_{22}H_{28}N_3O_3$ [M + H$^+$]: calculated 382.2125, found 382.2125.

(6a*R,8*R**,10a*S**)-6-(4-Methoxyphenyl)-8-(oxetan-3-ylamino)octahydro-1*H*,5*H*-benzo[*d*]pyrrolo[1,2-*c*]imidazol-5-one 39**

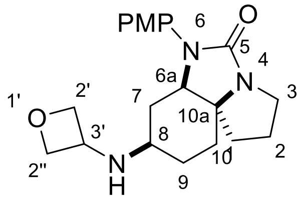

*(Assignment of stereochemistry: by analogy with **7a**)*

To a solution of the ketone **4b** (19.0 mg, 0.06 mmol, 1.0 eq.) in 6 mL THF, 3-aminooxetane (5.30 µL, 0.08 mmol, 1.2 eq.) and titanium isopropoxide (0.04 mL, 0.13 mmol, 2.0 eq.) were added and the mixture was left to stir at room temperature overnight. $NaBH_4$ (2.88 mg, 0.08 mmol, 1.2 eq.) was added to the reaction mixture at -78 °C and it was stirred at the same temperature for 1 h. It was then allowed to warm up to room temperature for 4 h. The reaction mixture was evaporated *in vacuo*. It was then taken up in 1 mL H_2O, diluted with 50 mL EtOAc, dried over Na_2SO_4 and evaporated *in vacuo*. Flash chromatography with 2 - 4% MeOH in DCM afforded the product **39** as a single diastereomer (13.0 mg, 0.04 mmol, 58% yield). **R_f** = 0.37. **^{1}H NMR** (400 MHz, $CDCl_3$): δ ppm 7.27 (2H, d, *J* = 8.8, Ar*H*), 6.81 (2H, d, *J* = 9.2, Ar*H*), 4.71 (1H, t, *J* = 6.4, 2'-H_A), 4.66 (1H, t, *J* = 6.8, 2''-H_A), 4.29 (1H, t, *J* = 6.4, 2'-H_B), 4.23 (1H, t, *J* = 6.4, 2''-H_B), 3.98 – 3.89 (2H, m, 6a-*H*, 3'-*H*), 3.85 (1H, ddd, *J* = 12.4, 9.0, 5.7, 3-H_A), 3.73 (3H, s, ArOC*H$_3$*), 2.98 (1H, ddd, *J* = 12.3, 9.1, 5.9, 3-H_B), 2.47 (1H, tt, *J* = 14.6, 4.0, 8-*H*), 2.09 (1H, dddd, *J* =10.1, 5.9, 4.0, 1.9, 7-H_A), 1.95 – 1.73 (3H, m, 2-$H_{A,B}$; 10-H_A), 1.70 – 1.54 (3H, m, 1-$H_{A,B}$; 9-H_A), 1.37 – 1.21 (2H, m, 9-H_B, 10-H_B), 1.21 – 1.13 (1H, m, 7-H_B). **^{13}C NMR** (100 MHz, $CDCl_3$): δ ppm 162.0 (5-*C*), 156.4 (*Ar*), 131.4 (*Ar*), 123.3 (*Ar*H), 114.4 (*Ar*H), 80.8 (2'-*C*), 80.3 (2''-*C*), 64.7 (10a-*C*), 58.2 (6a-*C*), 55.5 (ArO*C*H$_3$), 51.8 (8-*C*), 51.4 (3'-*C*), 44.3 (3-*C*), 36.6 (7-*C*), 35.4 (1-*C*), 29.8 (10-*C*), 28.8 (9-*C*), 23.2 (2-*C*). **IR** ν_{max} (neat)/cm^{-1}: 3296 (N-H), 2933, 2864 (C-H), 1691 (C=O), 1511, 1462, 1395 (C=C), 1246 (C-O). **HRMS** (ESI): $C_{20}H_{28}N_3O_3$ [M + H$^+$]: calculated 358.2125, found 358.2128.

(6a*R,8*R**,10a*S**)-8-(Azetidin-1-yl)-6-(4-methoxyphenyl)octahydro-1*H*,5*H*-benzo[*d*]pyrrolo[1,2-*c*]imidazol-5-one 40**

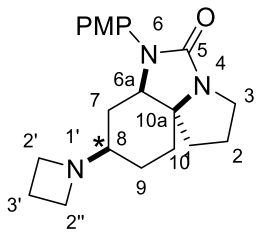

Major diastereomer, *dr* 95:5

(Assignment of stereochemistry: X-ray crystal structure - CCDC deposition number: 2175098)

To a solution of the ketone **4b** (20.0 mg, 0.07 mmol, 1.0 eq.) in 6 mL THF, azetidine (14.1 µL, 0.21 mmol, 3.0 eq.) and titanium isopropoxide (0.04 mL, 0.14 mmol, 2.0 eq.) were added and the mixture was left to stir at room temperature overnight. Sodium triacetoxyborohydride (29.7 mg, 0.14 mmol, 2.0 eq.) was added to the reaction mixture at -78 °C and it was allowed to warm up to room temperature overnight. 1 mL of saturated aqueous Na_2CO_3 was added. It was then diluted with 100 mL EtOAc, dried over Na_2SO_4 and evaporated *in vacuo*. Flash chromatography with 3 - 10% MeOH in DCM afforded the product **40** as a 95:5 mixture of diastereomers (10.0 mg, 0.03 mmol, 44% yield). R_f = 0.14 (10% MeOH in DCM). ^{1}H NMR (Major diastereomer, 500 MHz, $CDCl_3$): δ ppm 7.27 (2H, d, *J* = 9.0, Ar*H*), 6.81 (2H, d, *J* = 9.0, Ar*H*), 3.88 (1H, dd, *J* = 10.7, 6.2, 6a-*H*), 3.83 (1H, ddd, *J* = 12.0, 7.0, 3.5, 3-H_A), 3.72 (3H, s, ArOCH_3), 3.07 (4H, sext, *J* = 7.0, 2'-$H_{A,B}$; 2''-$H_{A,B}$), 2.97 (1H, ddd, *J* = 12.3, 9.3, 5.7, 3-H_B), 2.06 – 1.98 (2H, m, 7-H_A, 8-*H*), 1.94 (2H, t, *J* = 7.0, 3'-$H_{A,B}$), 1.92 – 1.74 (2H, m, 2-$H_{A,B}$), 1.66 – 1.49 (3H, m, 9-H_A, 10-$H_{A,B}$), 1.30 (1H, aptd, *J* = 11.5, 3.5, 1-H_A), 1.23 – 1.12 (2H, m, 1-H_B, 9-H_B), 1.05 (1H, q, *J* = 11.0, 7-H_B). Signal for minor isomer visible at: 4.15 (0.05H, t, *J* = 5.0, 6a-*H*). ^{13}C NMR (Major diastereomer, 125 MHz, $CDCl_3$): δ ppm 162.0 (5-*C*), 156.4 (*Ar*) 131.5 (*Ar*), 123.6 (*Ar*H), 114.3 (*Ar*H), 64.8 (10a-*C*), 62.5 (8-*C*), 58.0 (6a-*C*), 55.5 (ArO*C*H$_3$), 53.2 (2'/2''-*C*), 44.2 (3-*C*), 35.3 (10-*C*), 32.5 (7-*C*), 29.2 (1-*C*), 24.3 (9-*C*), 23.0 (2-*C*), 17.0 (3'-*C*). Signals for minor isomer visible at: δ ppm 62.3, 58.3, 32.9, 26.9, 23.7. IR ν_{max} (neat)/cm^{-1}: 2932, 2833 (C-H), 1692 (C=O), 1511, 1462, 1441 (C=C), 1244 (C-O). HRMS (ESI): $C_{20}H_{28}N_3O_2$ [M + H$^+$]: calculated 342.2176, found 342.2173.

(6a*R,10a*S**)-Hexahydro-1*H*,5*H*-benzo[*d*]pyrrolo[1,2-*c*]imidazole-5,8(6*H*)-dione S9**

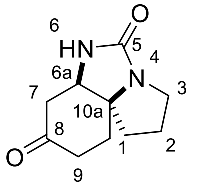

(Assignment of stereochemistry: X-ray crystal structure - CCDC deposition number: 2174966)

To a mixure of the ketone **4b** (13 mg, 0.04 mmol, 1.0 eq.) and CAN (94 mg, 0.17, 4.0 eq.) in 0.43 mL of HPLC grade MeCN, 0.24 mL of H_2O was added and the mixture was left to stir for 5 min. The reaction mixture was extracted with EtOAc (5 × 20 mL), dried over $MgSO_4$, and evaporated *in vacuo*.

Flash chromatography with 2 – 4% MeOH in DCM afforded the product **S9** as a pale yellow solid (8 mg, 0.04 mmol, 95% yield). Data matches that in the literature.[4] **^{1}H NMR** (400 MHz, $CDCl_3$): δ ppm 4.79 (1H, s, 6-*H*), 3.99 (1H, m, 6a-*H*), 3.65 (1H, ddd, *J* = 12.0, 5.6, 4.0, 3-H_A), 2.98 (1H, ddd, *J* = 12.0, 5.2, 2.0, 3-H_B), 2.64 (1H, dd, *J* = 16.3, 4.5, 7-H_A), 2.52 (1H, ddd, *J* = 18.8, 10.4, 1.6, 1-H_A), 2.45 (1H, dd, *J* = 16.4, 3.6, 7-H_B), 2.29 (1H, dt, *J* = 19.0, 3.9, 1-H_B), 1.99 – 1.83 (5H, m, 2-$H_{A,B}$; 10-H_A, 9-$H_{A,B}$), 1.81 – 1.71 (1H, m, 10-H_B). **^{13}C NMR** (100 MHz, $CDCl_3$): δ ppm 208.6 (8-*C*), 162.8 (5-*C*), 65.9 (10a-*C*), 55.1 (6a-*C*), 43.1 (3-*C*), 42.5 (7-*C*), 37.2 (10-*C*), 34.1 (1-*C*), 29.1 (9-*C*), 23.9 (2-*C*). **IR** ν_{max} (neat)/cm^{-1}: 3312 (N-H), 2969, 2938 (C-H), 1734, 1703 (C=O). **HRMS** (ESI): $C_{10}H_{14}N_2NaO_2$ [M + Na^+]: calculated 217.0947, found 217.0946.

(6a*R,8*R**,10a*S**)-8-Hydroxyoctahydro-1*H*,5*H*-benzo[*d*]pyrrolo[1,2-*c*]imidazole-5-one 41**

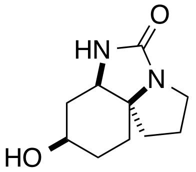

To a mixture of the ketone **S9** (14.0 mg, 0.07 mmol, 1.0 eq.) and $CeCl_3.7H_2O$ (32.2 mg, 0.09 mmol, 1.2 eq.) in 3 mL of HPLC grade MeOH at –78 °C, $NaBH_4$ (3.30 mg, 0.09 mmol, 1.2 eq.) was added and the reaction mixture was stirred at the same temperature for 30 min. It was then allowed to warm up to room temperature for another 30 min. The reaction mixture was evaporated *in vacuo*. It was then taken up in 1 mL H_2O, diluted with 50 mL EtOAc, dried over Na_2SO_4 and evaporated *in vacuo*. Flash chromatography with 3 – 8% MeOH in DCM afforded the product as an 85:15 mixture of diastereomers (12.0 mg, 0.06 mmol, 85% yield). **R_f** = 0.34 (8% MeOH in DCM). **^{1}H NMR** (**Major diastereomer**, 500 MHz, MeOD): δ ppm 4.81 (1H, s, N*H*), 3.73 – 3.65 (2H, m, 3-H_A, 8-*H*), 3.49 (1H, dd, *J* = 8.5, 6.5, 6a-*H*), 2.94 (1H, ddd, *J* = 12.0, 8.8, 6.2, 3-H_B), 2.17 (1H, dt, *J* = 12.5, 6.5, 7-H_A), 1.90 – 1.80 (4H, m, 1-H_A; 2-$H_{A,B}$; 9-H_A), 1.66 – 1.61 (1H, m, 10-H_A), 1.56 – 1.45 (3H, m, 1-H_B, 7-H_B, 10-H_B), 1.31 (1H, qd, *J* = 11.5, 3.5, 9-H_B), 1.18 (1H, s, 8-CHO*H*). Signal for minor isomer visible at: 4.12 (0.18H, quint, *J* = 5.0, 8-*H*). **^{13}C NMR** (**Major diastereomer**, 125 MHz, MeOD): δ ppm 164.5 (5-*C*), 66.0 (10a-*C*), 65.4 (8-*C*), 52.5 (6a-*C*), 42.9 (3-*C*), 39.0 (7-*C*), 34.0 (10-*C*), 29.3 (1-*C*), 28.2 (9-*C*), 23.0 (2-*C*). Signals for minor isomer visible at: 63.1, 52.3, 36.3, 34.7, 27.6, 26.0, 23.3. **IR** ν_{max} (neat)/cm^{-1}: 3298 (N-H, O-H); 2931, 2855 (C-H); 1689 (C=O); 1049 (C-O). **HRMS** (ESI): $C_{10}H_{17}N_2O_2$ [M + H^+]: calculated 197.1285, found 197.1278.

(b) by functionalisation of ring addition products (16 compounds)

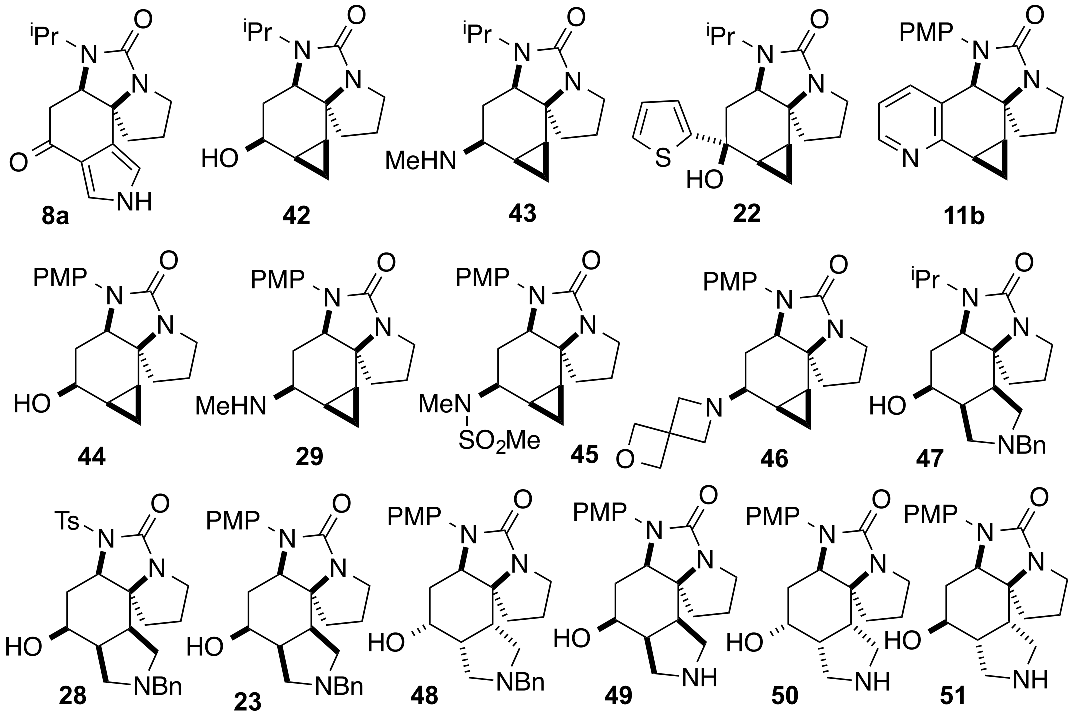

(6a*R,8*S**,8a*S**,11a*R**,11b*S**)-10-Benzyl-8-hydroxy-6-(4-methoxyphenyl)decahydro-1*H*-pyrrolo[1',2':3,4]imidazo[4,5-*e*]isoindol-5(6*H*)-one 23**

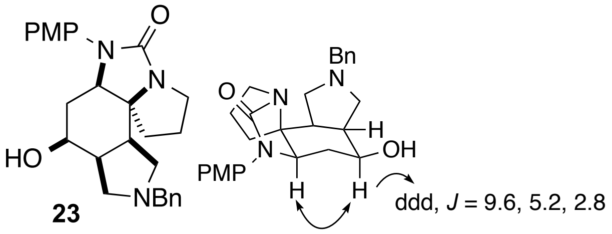

*(Stereochemical assignment of **23** through combination of NOESY and J constant analysis;*

To a solution of the pyrrolidine **9b** (26.0 mg, 0.06 mmol, 1.0 eq.) in 4 mL THF at -78 °C, LS-selectride (66.0 µL, 0.07 mmol, 1.1 eq.) was added and the mixture was allowed to stir for 1 h. The reaction mixture was quenched with 0.01 mL of 10% KOH and 0.01 mL H_2O. It was then diluted with 100 mL EtOAc, dried over Na_2SO_4 and evaporated *in vacuo*. Flash chromatography with 1 – 7% MeOH in DCM afforded the product **23** [7.6 mg, 0.02 mmol, 29% yield, $\mathbf{R_f}$ = 0.39 (7% MeOH in DCM)] ^{1}**H NMR** (500 MHz, $CDCl_3$): δ ppm 7.36 (2H, d, *J* = 9.0, Ar*H*), 7.34 – 7.27 (5H, m, Ar*H*), 6.88 (2H, d, *J* = 9.0, Ar*H*), 4.30 (1H, t, *J* = 5.0, 6a-*H*), 4.24 (1H, ddd, *J* = 9.6, 5.2, 2.8, 8-*H*), 3.89 (1H, ddd, *J* = 12.4, 9.4, 5.7, 3-H_A), 3.79 (3H, s, ArOCH_3), 3.73 (1H, d, *J* = 13.0, ArCH_{2A}), 3.66 (1H, d, *J* = 12.5, ArCH_{2B}), 3.09 (1H, ddd, *J* = 12.5, 8.0, 3.0, 3-H_B), 3.00 – 2.81 (2H, m, 9-$H_{A,B}$), 2.79 (1H, t, *J* = 9.0, 11-H_A), 2.66 (1H, q, *J* = 9.0, 11a-*H*), 2.63 – 2.57 (1H, m, 8a-*H*), 2.49 – 2.39 (1H, m, 11-H_B), 2.19 (1H, ddd, *J* = 14.6, 9.8, 5.1, 7-H_A), 1.97 – 1.74 (4H, m, 1-H_A; 2-$H_{A,B}$; 8-CHO*H*), 1.64 – 1.62 (1H, m, 7-H_B), 1.57 – 1.53 (1H, m, 1-H_B). ^{13}**C NMR** (125 MHz, $CDCl_3$): δ ppm 168.8 (5-*C*), 160.4 (*Ar*), 155.3 (*Ar*), 130.0 (*Ar*), 127.7 (*Ar*H), 127.5 (*Ar*H), 126.3 (*Ar*H),

122.2 (*Ar*H), 113.4 (*Ar*H), 64.6 (11b-*C*), 64.4 (8-*C*), 59.2 (Ar*C*H_2), 55.4 (11-*C*), 55.2 (9-*C*), 55.1 (6a-*C*), 54.5 (ArO*C*H_3), 43.2 (3-*C*), 39.0 (11a-*C*), 38.3 (8a-*C*), 32.9 (1-*C*), 31.1 (7-*C*), 22.2 (2-*C*). **IR** ν_{max} (neat)/cm^{-1}: 3399 (O-H), 2927, 2834, 2800 (C-H), 1688 (C=O), 1611, 1584, 1511 (C=C), 1245 (C-O). **HRMS** (ESI): $C_{26}H_{32}N_3O_3$ [M + H^+]: calculated 434.2438, found 434.2438.

(6a*R,8*S**,8a*S**,11a*R**,11b*S**)-10-Benzyl-8-hydroxy-6-isopropyldecahydro-1*H*-pyrrolo[1',2':3,4]imidazo[4,5-*e*]isoindol-5(6*H*)-one 28**

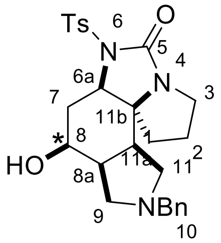

*(Assignment of stereochemistry: by analogy with **23**)*

To a solution of the pyrrolidine **9c** (16.0 mg, 0.03 mmol, 1.0 eq.) in 4 mL THF at -78 °C, LS-selectride (37.0 µL, 0.04 mmol, 1.1 eq.) was added and the mixture was allowed to stir for 1 h. The reaction mixture was quenched with 0.01 mL of 10% KOH and 0.01 mL H_2O. It was then diluted with 100 mL EtOAc, dried over Na_2SO_4 and evaporated *in vacuo*. Flash chromatography with 3 – 7% MeOH in DCM afforded the product as a single diastereomer (6.0 mg, 0.01 mmol, 37% yield). $\mathbf{R_f}$ = 0.37 (8% MeOH in DCM). **^{1}H NMR** (500 MHz, $CDCl_3$): δ ppm 7.89 (2H, d, *J* = 8.3, Ar*H*), 7.29 – 7.20 (7H, m, Ar*H*), 4.09 (1H, dd, *J* = 10.5, 5.4, 6a-*H*), 3.77 (1H, dt, *J* = 9.3, 5.6, 8-*H*), 3.70 (1H, d, *J* = 13.5, ArCH_{2A}), 3.65 (1H, ddd, *J* = 12.5, 6.0, 2.5, 3-H_A), 3.62 (1H, d, *J* = 13.0, ArCH_{2B}), 2.96 – 2.89 (1H, m, 11-H_A), 2.88 – 2.78 (3H, m, 3-H_B, 9-$H_{A,B}$), 2.44 – 2.38 (1H, m, 7-H_A), 2.36 (3H, s, ArCH_3), 2.32 (1H, dd, *J* = 10.6, 8.5, 11-H_B), 2.18 (1H, ddd, *J* = 14.0, 9.5, 5.5, 7-H_B), 2.16 – 2.11 (1H, m, 8a-*H*), 1.95 – 1.88 (2H, m, 11a-*H*, 8-CHO*H*), 1.78 (1H, ddd, *J* = 12.2, 7.5, 4.6, 1-H_A), 1.72 – 1.64 (2H, m, 2-$H_{A,B}$), 1.55 (1H, ddd, *J* = 12.0, 4.5, 2.0, 1-H_B). **^{13}C NMR** (125 MHz, $CDCl_3$): δ ppm 157.5 (5-*C*), 143.9 (*Ar*), 138.0 (*Ar*, **missing – observed through the HMBC of ArC$H_{2A,B}$**), 134.2 (*Ar*), 128.5 (*Ar*H), 127.8 (*Ar*H), 127.5 (*Ar*H), 127.4 (*Ar*H), 126.4 (*Ar*H), 69.5 (8-*C*), 65.8 (11b-*C*), 61.0 (6a-*C*), 59.8 (Ar*C*H_2), 55.0 (9-*C*), 53.0 (11-*C*), 46.9 (11a-*C*), 46.7 (3-*C*), 43.4 (8a-*C*), 36.3 (7-*C*), 35.5 (1-*C*), 23.5 (2-*C*), 20.7 (Ar*C*H_3). **IR** ν_{max} (neat)/cm^{-1}: 3399 (O-H), 3030, 2923 (C-H), 1729 (C=O), 1596, 1494, 1453 (C=C), 1169 (C-O). **HRMS** (ESI): $C_{26}H_{32}N_3O_4S$ [M + H^+]: calculated 482.2108, found 482.2108.

(6a*R,8*S**,8a*R**,9a*S**, 9b*S**)-8-Hydroxy-6-isopropyloctahydro-1*H*-cyclopropa[5,6]benzo[1,2-*d*]pyrrolo[1,2-*c*]imidazol-5(6*H*)-one 42**

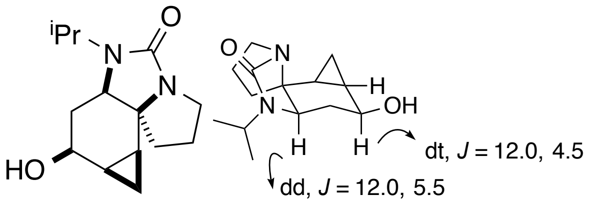

(Assignment of stereochemistry: analysis of J values)

A mixture of the cyclopropane scaffold **10a** (30.0 mg, 0.12 mmol, 1.0 eq.) and $CeCl_3.7H_2O$ (54.0 mg, 0.14 mmol, 1.2 eq.) in 3 mL of HPLC grade MeOH was allowed to stir at –78 °C for 30 min. $NaBH_4$ (5.48 mg, 0.14 mmol, 1.2 eq.) was then added and the mixture was left to warm up to room temperature for another 30 min. The reaction mixture was evaporated *in vacuo*. It was then taken up in 1 mL H_2O, diluted with 50 mL EtOAc, dried over Na_2SO_4 and evaporated *in vacuo*. Flash chromatography with 1 – 3% MeOH in DCM afforded the product **42** as a 90:10 mixture of diastereomers (27.0 mg, 0.11 mmol, 90% yield). $\mathbf{R_f}$ = 0.29 (4% MeOH in DCM). **^{1}H NMR** (Major diastereomer, 500 MHz, $CDCl_3$): 4.19 (1H, dt, *J* = 12.0, 4.5, 8-*H*), 3.92 (1H, hept, *J* = 7.0, isopropyl C*H*), 3.73 (1H, ddd, *J* = 12.0, 6.0, 3.5, 3-H_A), 3.27 (1H, dd, *J* = 12.0, 5.5, 6a-*H*), 2.95 (1H, ddd, *J* =12.1, 8.9, 6.1, 3-H_B), 1.99 (1H, dtd, *J* = 12.5, 5.0, 1.0, 7-H_A), 1.88 – 1.81 (2H, m, 2-$H_{A,B}$), 1.74 – 1.70 (2H, m, 1-$H_{A,}$ 8-CHO*H*), 1.45 (1H, tt, *J* = 8.5, 5.0, 8a-*H*), 1.37 (1H, q, *J* = 11.0, 1-H_B), 1.21 – 1.13 (1H, m, 7-H_B), 1.12 (3H, d, *J* = 6.5, isopropyl CH_{3A}), 1.10 (3H, d, *J* = 7.0, isopropyl CH_{3B}), 0.92 (1H, td, 8.9, 5.3, 9a-*H*), 0.75 (1H, q, *J* = 5.6, 9-H_A), 0.52 (1H, td, *J* = 8.6, 5.7, 9-H_B). Signal for minor isomer visible at: 4.33 (0.11H, dt, *J* = 4.0, 2.0, 8-*H*). **^{13}C NMR** (Major diastereomer, 125 MHz, $CDCl_3$): δ ppm 162.9 (5-*C*), 65.6 (8-*C*), 64.3 (9b-*C*), 55.5 (6a-*C*), 44.2 (isopropyl *C*H), 43.7 (3-*C*), 36.8 (1-*C*), 34.0 (7-*C*), 22.8 (2-*C*), 22.4 (isopropyl CH_{3A}), 19.7 (isopropyl CH_{3B}), 19.5 (8a-*C*), 18.9 (9a-*C*), 4.4 (9-*C*). Signals for minor isomer visible at: 65.4, 64.5, 50.0, 43.6. **IR** ν_{max} (neat)/cm^{-1}: 3378 (O-H), 2967, 2880 (C-H), 1667 (C=O), 1221 (C-O). **HRMS** (ESI): $C_{14}H_{23}N_2O_2$ [M + H$^+$]: calculated 251.1754, found 251.1754.

(6a*R,8*S**,8a*R**,9a*S**, 9b*S**)-6-Isopropyl-8-(methylamino)octahydro-1*H*-cyclopropa[5,6]benzo[1,2-*d*]pyrrolo[1,2-*c*]imidazol-5(6*H*)-one 43**

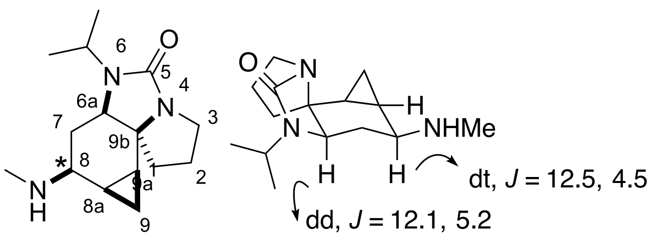

(Assignment of stereochemistry: analysis of J values)

To a solution of the cyclopropane scaffold **10a** (30.0 mg, 0.12 mmol, 1.0 eq.) in 6 mL THF, 2M methylamine in MeOH (0.60 mL, 1.21 mmol, 10.0 eq.) and titanium isopropoxide (0.06 mL, 0.24 mmol, 2.0 eq.) were added and the mixture was left to stir at room temperature overnight. $NaBH_4$

(6.90 mg, 0.18 mmol, 1.5 eq.) was added to the reaction mixture at -78 °C and it was stirred at the same temperature for 30 min. It was then allowed to warm up to room temperature for another 30 min. The reaction mixture was evaporated *in* vacuo. It was then taken up in 1 mL H_2O, diluted with 50 mL EtOAc, dried over Na_2SO_4 and evaporated *in vacuo*. To a solution of the crude, isonicotinoyl chloride (23.7 mg, 0.13 mmol, 1.1 eq.) and DMAP (14.8 mg, 0.12 mmol, 1.0 eq.) in 1 mL DMF; TEA (0.10 mL, 0.73 mmol,6.0 eq.) was added. The reaction mixture was stirred at room temperature for 16 h and then heated at 100 °C for 6 h. It was then evaporated *in vacuo*. Flash chromatography with 1 - 3% of saturated NH_3/MeOH in DCM afforded the intermediate amine **43** as a single diastereomer (8.0 mg, 0.030 mmol, 25% yield). $\mathbf{R_f}$ = 0.37 (6% of saturated NH_3/MeOH in DCM). **^{1}H NMR** (500 MHz, $CDCl_3$): δ ppm 3.92 (1H, hept, *J* = 7.0, isopropyl C*H*), 3.77 (1H, ddd, *J* = 11.5, 6.5, 4.0, 3-H_A), 3.24 (1H, dd, *J* = 12.1, 5.2, 6a-*H*), 3.00 (1H, dt, *J* = 12.5, 4.5, 8-*H*), 2.94 (1H, ddd, *J* = 11.0, 6.5, 3.0, 3-H_B), 2.47 (3H, s, NHCH_3), 1.92 (1H, dt, *J* =12.5, 5.0, 7-H_A), 1.87 – 1.80 (2H, m, 2-$H_{A,B}$), 1.72 (1H, ddd, *J* = 12.0, 6.5, 3.5, 1-H_A), 1.65 (1H, s, N*H*), 1.47 – 1.41 (1H, m, 8a-*H*), 1.38 (1H, q, *J* = 11.5, 1-H_B), 1.12 (3H, d, *J* = 7.0, isopropyl CH_{3A}), 1.10 (3H, d, *J* = 7.0, isopropyl CH_{3B}), 0.97 (1H, q, *J* = 12.5, 7-H_B), 0.85 (1H, td, *J* = 8.9, 5.3, 9a-*H*), 0.60 (1H, q, *J* = 5.6, 9-H_A), 0.45 (1H, td, *J* = 8.7, 5.6, 9-H_B). **^{13}C NMR** (125 MHz, $CDCl_3$): δ ppm 161.9 (5-*C*), 63.8 (9b-*C*), 54.5 (6a-*C*), 51.6 (8-*C*), 43.2 (isopropyl *C*H), 42.6 (3-*C*), 35.9 (1-*C*), 32.3 (NH*C*H_3), 30.6 (7-*C*), 21.7 (2-*C*), 21.4 (isopropyl *C*H_{3A}), 18.5 (isopropyl *C*H_{3A}), 16.7 (9a-*C*), 16.1 (8a-*C*), 3.1 (9-*C*). **IR** v_{max} (neat)/cm^{-1}: 3302 (N-H), 2964, 2933, 2790 (C-H), 1688 (C=O). **HRMS** (ESI): $C_{15}H_{26}N_3O$ [M + H$^+$]: calculated 264.2070, found 264.2063.

(6a*R**,8*S**,8a*R**,9a*S**, 9b*S**)-8-Hydroxy-6-(4-methoxyphenyl)octahydro-1*H*-cyclopropa[5,6]benzo[1,2-*d*]pyrrolo[1,2-*c*]imidazol-5(6*H*)-one 44

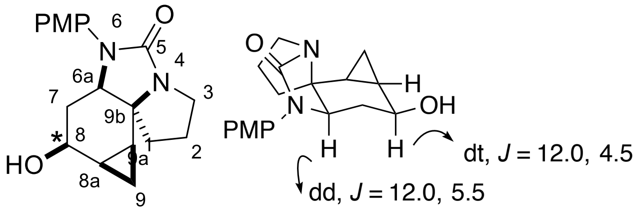

(Assignment of stereochemistry: analysis of J values)

A mixture of the cyclopropane scaffold **10b** (15.0 mg, 0.05 mmol, 1.0 eq.) and $CeCl_3.7H_2O$ (21.5 mg, 0.06 mmol, 1.2 eq.) in 3 mL of HPLC grade MeOH was allowed to stir at –78 °C for 30 min. $NaBH_4$ (2.20 mg, 0.06 mmol, 1.2 eq.) was then added and the mixture was left to warm up to room temperature for another 30 min. The reaction mixture was evaporated *in vacuo*. It was then taken up in 1 mL H_2O, diluted with 50 mL EtOAc, dried over Na_2SO_4 and evaporated *in vacuo*. Flash chromatography with 1 – 4% MeOH in DCM afforded the product

44 as a single diastereomer (14.0 mg, 0.04 mmol, 93% yield). **R$_f$** = 0.44 (100% EtOAc). **^{1}H NMR** (500 MHz, $CDCl_3$): 7.21 (2H, d, *J* = 9.0, Ar*H*), 6.79 (2H, d, *J* = 9.0, Ar*H*), 4.19 (1H, dt, *J* = 12.0, 4.5, 8-*H*), 3.83 (1H, ddd, *J* = 14.5, 8.0, 2.5, 3-H_A), 3.78 (1H, dd, *J* = 12.5, 5.5, 6a-*H*), 3.71 (3H, s, ArOCH_3), 3.08 (1H, ddd, *J* =14.5, 8.1, 4.3, 3-H_B), 1.99 – 1.96 (1H, m, 7-H_A), 1.95 – 1.90 (2H, m, 2-$H_{A,B}$), 1.90 – 1.84 (1H, m, 1-H_A), 1.63 (1H, q, *J* = 11.0, 1-H_B), 1.53 – 1.47 (2H, m, 8a-*H*, 8-CHO*H*), 1.04 (1H, q, *J* = 12.5, 7-H_B), 1.01 – 0.96 (1H, m, 9a-*H*), 0.80 (1H, q, *J* = 5.6, 9-H_A), 0.58 (1H, td, *J* = 8.7, 5.8, 9-H_B). **^{13}C NMR** (125 MHz, $CDCl_3$): δ ppm 160.2 (5-*C*), 155.5 (*Ar*), 130.0 (*Ar*), 122.7 (*Ar*H), 113.3 (*Ar*H), 64.4 (8-*C*), 62.6 (9b-*C*), 57.6 (6a-*C*), 54.5 (ArO*C*H_3), 42.9 (3-*C*), 36.3 (1-*C*), 30.0 (7-*C*), 21.8 (2-*C*), 19.1 (8a-*C*), 18.`1 (9a-*C*), 3.5 (9-*C*). **IR** ν_{max} (neat)/cm^{-1}: 3401 (O-H), 3010, 2954, 2836 (C-H), 1677 (C=O), 1611, 1511, 1462 (C=C), 1245 (C-O). **HRMS** (ESI): $C_{18}H_{23}N_2O_3$ [M + H^+]: calculated 315.1703, found 315.1699.

***N*-[(6a*R**,8*S**,8a*R**,9a*S**,9b*S**)-6-(4-Methoxyphenyl)-5-oxodecahydro-1*H*-cyclopropa[5,6]benzo[1,2-*c*]imidazol-8-yl]methanesulfonamide 45**

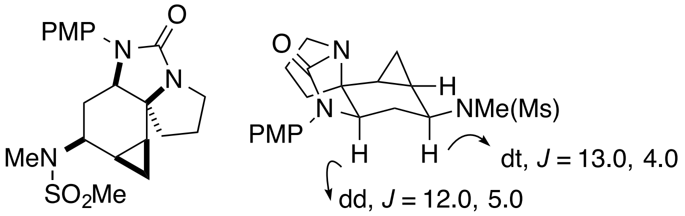

(Assignment of stereochemistry: analysis of J values)

To a solution of the cyclopropane scaffold **10b** (23.0 mg, 0.07 mmol, 1.0 eq.) in 4 mL THF, 2M methylamine in MeOH (0.37 mL, 0.74 mmol, 10.0 eq.) and titanium isopropoxide (0.04 mL, 0.15 mmol, 2.0 eq.) were added and the mixture was left to stir at room temperature overnight. $NaBH_4$ (4.18 mg, 0.11 mmol, 1.5 eq.) was added to the reaction mixture at -78 °C and it was stirred at the same temperature for 30 min. It was then allowed to warm up to room temperature for another 30 min. The reaction mixture was evaporated *in* vacuo. It was then taken up in 1 mL H_2O, diluted with 50 mL EtOAc, dried over Na_2SO_4 and evaporated *in vacuo*. To a solution of the crude and DMAP (1.00 mg, 0.12 mmol, 0.11 eq.) in 5 mL DCM; methanesulfonyl chloride (0.01 mL, 0.13 mmol, 1.7 eq.) and TEA (0.02 mL, 0.14 mmol, 1.9 eq.) were added. The reaction mixture was then stirred at room temperature overnight. It was evaporated *in vacuo*. Flash chromatography with 1 - 2% MeOH in DCM afforded the product as an 89:11 mixture of diastereomers (21.0 mg, 0.05 mmol, 73% yield). **R$_f$** = 0.56 (4% MeOH in DCM). **^{1}H NMR** (**Major diastereomer**, 500 MHz, $CDCl_3$): δ ppm 7.20 (2H, d, *J* = 9.0, Ar*H*), 6.80 (2H, d, *J* = 9.0, Ar*H*), 4.45 (1H, dt, *J* = 13.0, 4.0, 8-*H*), 3.93 (1H, dd, *J* = 12.0, 5.0, 6a-*H*), 3.81 (1H, ddd, *J* = 12.5, 6.5, 3.5, 3-H_A), 3.72 (3H, s, ArOCH_3), 3.06 (1H, ddd, *J* = 12.0, 5.5, 3.5, 3-H_B), 2.77 (3H, s,

SCH_3), 2.74 (3H, s, NCH_3), 1.97 -1.88 (3H, m, 1-H_A, 2-$H_{A,B}$), 1.72 (1H, dt, *J* = 13.0, 4.5, 7-H_A), 1.69 - 1.60 (1H, m, 1-H_B), 1.34 (1H, q, *J* = 12.5, 7-H_B), 1.28 – 1.20 (1H, m, 8a-*H*), 0.94 – 0.86 (2H, m, 9-H_A, 9a-*H*), 0.80 -0.73 (1H, m, 9-H_B). Signal for minor isomer visible at: 4.36 (0.12H, br.d, *J* = 11.2, 6a-*H*). **^{13}C NMR** (**Major diastereomer**,125 MHz, $CDCl_3$): δ ppm 159.9 (5-*C*), 155.6 (*Ar*), 129.7 (*Ar*), 122.6 (*Ar*H), 113.4 (*Ar*H), 62.4 (9b-*C*), 57.8 (6a-*C*), 54.5 ($ArOCH_3$), 49.8 (8-*C*), 42.9 (3-*C*), 37.8 (SCH_3), 36.5 (1-*C*), 27.9 (NCH_3), 25.4 (7-*C*), 21.8 (2-*C*), 15.2 (9a-*C*), 15.0 (8a-*C*), 5.4 (9-*C*). Signals for minor isomer visible at: 159.6, 155.3, 122.0, 113.3, 73.7, 70.0, 62.3, 55.9, 53.4, 49.3, 48.7, 27.0, 17.7, 15.8, 6.4. **IR** ν_{max} (neat)/cm^{-1}: 2956 (C-H), 1695 (C=O), 1512, 1464, 1444 (C=C), 1246 (C-O). **HRMS** (ESI): $C_{20}H_{28}N_3O_4S$ [M + H$^+$]: calculated 406.1795, found 406.1794.

(6a*R,8*S**,8a*R**,9a*S**,9b*S**)-6-(4-Methoxyphenyl)-8-(2-oxa-6-azaspiro[3.3]heptan-6-yl)octahydro-1*H*-cyclopropa[5,6]benzo[1,2-*d*]imidazol-5(6*H*)-one 46**

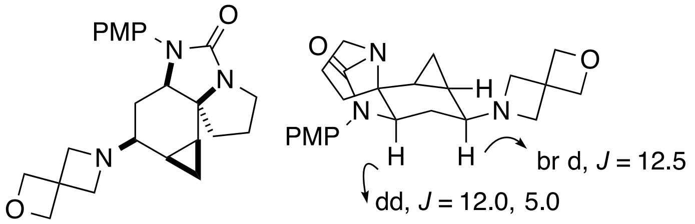

(Assignment of stereochemistry: analysis of J values)

To a solution of the cyclopropane scaffold **10b** (17.0 mg, 0.05 mmol, 1.0 eq.) in 4 mL THF, 2-oxa-6-aza-spiro[3.3]heptane (0.01 mL, 0.11 mmol, 2.0 eq.) and titanium isopropoxide (0.03 mL, 0.11 mmol, 2.0 eq.) were added and the mixture was left to stir at room temperature overnight. $NaBH_4$ (3.10 mg, 0.08 mmol, 1.5 eq.) was added to the reaction mixture at -78 °C and it was stirred at the same temperature for 30 min. It was then allowed to warm up to room temperature for another 30 min. The reaction mixture was evaporated *in* vacuo. It was then taken up in 1 mL H_2O, diluted with 50 mL EtOAc, dried over Na_2SO_4 and evaporated *in vacuo*. Flash chromatography with 1 - 6% MeOH in DCM afforded the product as an 88:12 mixture of diastereomers (19.0 mg, 0.05 mmol, 88% yield). **R_f** = 0.40 (4% MeOH in DCM). **^{1}H NMR** (**Major diastereomer**, 500 MHz, $CDCl_3$): δ ppm 7.19 (2H, d, *J* = 9.0, Ar*H*), 6.80 (2H, d, *J* = 9.5, Ar*H*), 4.65 (4H, s, 1'-$H_{A,B}$; 3'-$H_{A,B}$), 3.79 (1H, ddd, *J* = 12.0, 7.5, 4.5, 3-H_A), 3.72 (3H, s, $ArOCH_3$), 3.70 (1H, dd, *J* = 12.0, 5.0, 6a-*H*), 3.32 (4H, s, 5'-$H_{A,B}$; 7'-$H_{A,B}$), 3.06 (1H, dt, *J* = 12.2, 7.4, 3-H_B), 2.54 (1H, br.d, *J* = 12.5, 8-*H*), 1.96 -1.82 (3H, m, 1-H_A, 2-$H_{A,B}$), 1.66 – 1.56 (2H, m, 1-H_B, 7-H_A), 1.23 – 1.17 (1H, m, 8a-*H*), 0.87 (1H, td, *J* = 8.9, 5.3, 9a-*H*), 0.80 (1H, q, *J* = 12.5, 7-H_B), 0.72 (1H, q, *J* = 5.5, 9-H_A), 0.55 – 0.48 (1H, m, 9-H_B). Signal for minor isomer visible at: 4.15 (0.13H, dd, *J* = 11.2, 6.0, 6a-*H*). **^{13}C NMR** (125 MHz, $CDCl_3$): δ ppm 160.1 (5-*C*), 155.5 (*Ar*), 130.1 (*Ar*), 122.7 (*Ar*H), 113.4 (*Ar*H), 80.2 (1' or 3'-*C*), 63.0 (9b-*C*), 60.7 (5' or 7'-*C*), 59.2 (8-*C*), 57.1 (6a-*C*), 54.5 ($ArOCH_3$), 42.8 (3-*C*), 37.7 (4'-*C*), 36.5 (1-*C*), 24.2 (7-*C*), 21.8 (2-*C*), 16.0 (9a-*C*), 14.0 (8a-*C*), 3.8 (9-*C*). Signals for minor isomer visible at:

121.8, 113.2, 68.8, 65.3, 62.6, 53.6, 38.6, 36.2, 31.1, 28.6, 21.0, 6.4. **IR** ν_{max} (neat)/cm^{-1}: 2936, 2863, 2833 (C-H), 1692 (C=O), 1583, 1511, 1460 (C=C), 1245 (C-O). **HRMS** (ESI): $C_{23}H_{30}N_3O_3$ [M + H$^+$]: calculated 396.2282, found 396.2279.

(6a*R,8a*S**,11a*R**,11b*S**)-10-Benzyl-8-hydroxy-6-isopropyldecahydro-1*H*-pyrrolo[1',2':3,4]imidazo[4,5-*e*]isoindol-5(6*H*)-one 47**

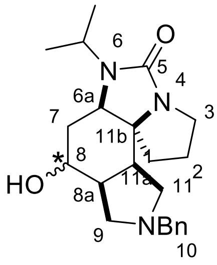

***dr* 3:2**

*(Assignment of stereochemistry: by analogy with **23**)*

A mixture of the pyrrolidine **9a** (17.0 mg, 0.04 mmol, 1.0 eq.) and $CeCl_3.7H_2O$ (20.7 mg, 0.06 mmol, 1.2 eq.) in 3 mL of HPLC grade MeOH was allowed to stir at rt for 30 min. The temperature was lowered to –78 °C and $NaBH_4$ (2.10 mg, 0.06 mmol, 1.2 eq.) was then added and the mixture was left to warm up to room temperature for another 30 min. The reaction mixture was evaporated *in vacuo*. It was then taken up in 1 mL H_2O, diluted with 50 mL EtOAc, dried over Na_2SO_4 and evaporated *in vacuo*. Flash chromatography with 5 – 10% MeOH in DCM afforded the product as a 3:2 mixture of diastereomers (10.0 mg, 0.03 mmol, 58% yield). **R_f** = 0.26 (10% MeOH in DCM). **^{1}H NMR** (Major diastereomer, 500 MHz, $CDCl_3$): δ ppm 3.99 (1H, hept, *J* = 7.0, 17-*H*). **^{1}H NMR** (Minor diastereomer, 500 MHz, $CDCl_3$): δ ppm 3.89 (0.7H, hept, *J* = 7.0, 17'-*H*). **^{13}C NMR** (125 MHz, $CDCl_3$): δ ppm 163.2, 161.3, 128.3, 127.92, 127.87, 127.8, 127.5, 127.4, 127.3, 126.3, 65.6, 65.3, 63.1, 62.1, 59.5, 59.3, 59.1, 57.2, 56.7, 53.9, 53.3, 50.8, 47.3, 44.3, 43.8, 43.4, 43.3, 41.8, 40.2, 39.4, 37.5, 36.1, 35.7, 33.7, 23.9, 23.7, 21.0, 20.4, 18.2, 17.9. **IR** ν_{max} (neat)/cm^{-1}: 3364 (O-H), 2965, 2923 (C-H), 1671 (C=O), 1453, 1418, 1380 (C=C), 1072 (C-O). **HRMS** (ESI): $C_{22}H_{32}N_3O_2$ [M + H$^+$]: calculated 370.2489, found 370.2485.

(6a*R,8*R**,8a*R**,11a*S**,11b*S**)-10-Benzyl-8-hydroxy-6-(4-methoxyphenyl)decahydro-1*H*-pyrrolo[1',2':3,4]imidazo[4,5-*e*]isoindol-5(6*H*)-one 48**

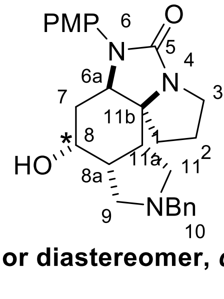

Major diastereomer, *dr* 3:1

*(Stereochemical assignment: from compound **50**)*

To a solution of the pyrrolidine **9b'** (40.0 mg, 0.09 mmol, 1.0 eq.) in 4 mL THF at -78 °C, LS-selectride (0.10 mL, 0.10 mmol, 1.1 eq.) was added and the mixture was allowed to stir for 1 h. The reaction mixture was quenched with 0.01 mL of 10% KOH and 0.01 mL H_2O. It was then diluted with 100 mL EtOAc, dried over Na_2SO_4 and evaporated *in vacuo*. Flash chromatography with 3 – 10% MeOH in DCM afforded the product as a 3:1 mixture of diastereomers (16 mg, 0.04 mmol, 40% yield). $\mathbf{R_f}$ = 0.28 (8% MeOH in DCM). **^{1}H NMR** (Major diastereomer, 500 MHz, $CDCl_3$): δ ppm 7.34 – 7.20 (5H, m, Ar*H*), 7.13 (2H, d, *J* = 9.0, Ar*H*), 6.83 (2H, d, *J* = 9.0, Ar*H*), 4.08 (1H, t, *J* = 4.3, 6a-*H*), 3.71 (3H, s, ArOC*H*$_3$), 3.65 (1H, d, *J* = 12.9, ArC*H*$_{2A}$). **No further characterisation possible because of extensive signal overlap**. Signal for minor isomer visible at: 4.13 (0.35H, t, *J* = 8.5, 6a-*H*). **^{13}C NMR** (Major diastereomer, 125 MHz, $CDCl_3$): δ ppm 161.2, 159.3, 155.9, 155.6, 129.6, 127.8, 127.4, 123.3, 113.5, 69.8, 64.6, 62.0, 60.0, 55.9, 54.5, 52.7, 46.9, 46.8, 43.5, 36.8, 32.8, 24.4. Signals for minor isomer visible at: 155.6, 129.8, 128.0, 127.6, 126.2, 123.2, 113.3, 64.9, 63.2, 61.4, 59.4, 56.8, 54.5, 53.9, 44.6, 43.7, 40.3, 37.9, 31.1, 24.1. **IR** ν_{max} (neat)/cm^{-1}: 3368 (O-H), 2924 (C-H), 1692 (C=O), 1513, 1454, 1427 (C=C), 1246 (C-O). **HRMS** (ESI): $C_{26}H_{32}N_3O_3$ [M + H$^+$]: calculated 434.2438, found 434.2435.

(6a*R**,8*S**,8a*S**,11a*R**,11b*S**)-8-Hydroxy-6-(4-methoxyphenyl)decahydro-1*H*-pyrrolo[1',2':3,4]imidazo[4,5-*e*]isoindol-5(6*H*)-one 49

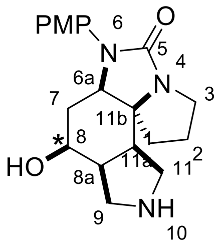

To a solution of the pyrrolidine **9b** (52.0 mg, 0.12 mmol, 1.0 eq.) in 4 mL THF at -78 °C, LS-selectride (0.13 mL, 0.13 mmol, 1.1 eq.) was added and the mixture was allowed to stir for 1 h. The reaction mixture was quenched with 0.01 mL of 10% KOH and 0.01 mL H_2O. It was then diluted with 100 mL EtOAc, dried over Na_2SO_4 and evaporated *in vacuo*. To a mixture of the crude and $Pd(OH)_2$/C (in excess to form a slurry) under nitrogen, 10 mL of HPLC grade MeOH was added gently. The reaction mixture was degassed and hydrogen gas was bubbled through it with the aid of a balloon, and this procedure was repeated twice. The mixture was then allowed to stir under a balloon of hydrogen overnight at room temperature. The reaction mixture was then filtered through a plug of Celite washing with 100 mL MeOH and evaporated *in vacuo*. Flash chromatography eluting with 5 - 15% of saturated NH_3/MeOH in

DCM afforded the product **49** as a single diastereomer (11 mg, 0.03 mmol, 27%). $\mathbf{R_f}$ = 0.44 (15% of saturated NH_3/MeOH in DCM). **^{1}H NMR** (500 MHz, $CDCl_3$): δ ppm 7.31 (2H, d, *J* = 9.0, Ar*H*), 6.82 (2H, d, *J* = 9.5, Ar*H*), 4.26 (1H, t, *J* = 5.3, 6a-*H*), 4.19 (1H, dt, *J* = 8.5, 4.5, 8-*H*), 3.86 (1H, ddd, *J* = 12.5, 9.5, 5.8, 3-H_A), 3.73 (3H, s, ArOCH_3), 3.27 – 3.13 (3H, m, 9-$H_{A,B}$; 11-H_A), 3.06 (1H, ddd, *J* = 12.0, 4.5, 3.0, 3-H_B), 2.75 (1H, t, *J* = 9.8, 11-H_B), 2.53 – 2.43 (3H, m, 8a-*H*, 11a-*H*, 8-CHO*H*), 2.05 (1H, ddd, *J* = 14.0, 8.5, 5.5, 7-H_A), 1.97 – 1.73 (4H, m, 1-H_A; 2-$H_{A,B}$; 10-N*H*), 1.61 – 1.53 (2H, m, 1-H_B, 7-H_B). **^{13}C NMR** (125 MHz, $CDCl_3$): δ ppm 160.5 (5-*C*), 155.4 (*Ar*), 130.0 (*Ar*), 122.3 (*Ar*H), 113.4 (*Ar*H), 64.6 (8-*C*), 64.4 (11b-*C*), 54.8 (6a-*C*), 54.5 (ArO*C*H_3), 48.7 (9-*C*), 48.2 (11-*C*), 43.3 (3-*C*), 40.4 (8a-*C*), 38.8 (11a-*C*), 32.9 (1-*C*), 31.4 (7-*C*), 22.2 (2-*C*). **IR** ν_{max} (neat)/cm^{-1}: 3294 (O-H, N-H), 3051, 2927 (C-H), 1687 (C=O), 1611, 1512, 1461 (C=C), 1246 (C-O). **HRMS** (ESI): $C_{19}H_{26}N_3O_3$ [M + H$^+$]: calculated 344.1969, found 344.1964.

(6a*R,8*R**,8a*R**,11a*S**,11b*S**)-8-Hydroxy-6-(4-methoxyphenyl)decahydro-1*H*-pyrrolo[1',2':3,4]imidazo[4,5-*e*]isoindol-5(6*H*)-one 50** and **(6a*R**,8*S**,8a*R**,11a*S**,11b*S**)-8-hydroxy-6-(4-methoxyphenyl)decahydro-1*H*-pyrrolo[1',2':3,4]imidazo[4,5-*e*]isoindol-5(6*H*)-one 51**

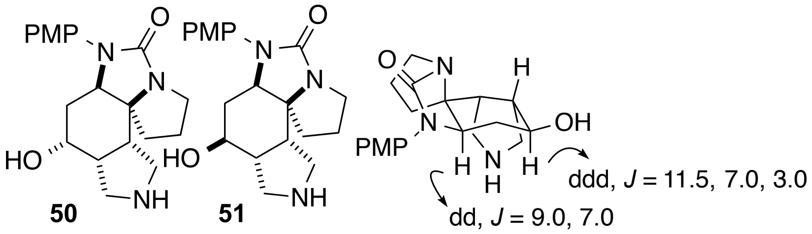

*(Stereochemical assignment of **51** by analysis of J constants; compound **50** by deduction)*

To a solution of the pyrrolidine **9b'** (39.0 mg, 0.09 mmol, 1.0 eq.) in 4 mL THF at -78 °C, LS-selectride (0.10 mL, 0.10 mmol, 1.1 eq.) was added and allowed to stir for 1 h. The reaction mixture was quenched with 0.01 mL of 10% KOH and 0.01 mL H_2O. It was then diluted with 100 mL EtOAc, dried over Na_2SO_4 and evaporated *in vacuo*. To a mixture of the crude and $Pd(OH)_2$/C (in excess to form a slurry) under nitrogen, 10 mL of HPLC grade MeOH was added gently. The reaction mixture was degassed and hydrogen gas was bubbled through it with the aid of a balloon, and this procedure was repeated twice. The mixture was then allowed to stir under a balloon of hydrogen overnight at room temperature. The reaction mixture was then filtered through a plug of Celite washing with 100 mL MeOH and evaporated *in vacuo*. Flash chromatography eluting with 10 - 15% of saturated NH_3/MeOH in DCM afforded the products **50** [9.00 mg, 0.03 mmol, 30% yield, $\mathbf{R_f}$ = 0.24 (20% of saturated NH_3/MeOH in DCM)] and **51** [3.0 mg, 0.01 µmol, 7% yield, $\mathbf{R_f}$ = 0.51 (20% of saturated NH_3/MeOH in DCM)]. **^{1}H NMR** (**Major diastereomer 50**, 500 MHz, $CDCl_3$): δ ppm 7.17 (2H, d, *J* = 9.0, Ar*H*), 6.83 (2H, d, *J* = 9.5, Ar*H*), 4.07 (1H, t, *J* = 4.7, 6a-*H*), 3.81 (1H, ddd, *J* = 10.5, 7.1, 3.4, 3-H_A), 3.72

(3H, s, ArOCH_3), 3.70 – 3.66 (1H, m, 8-*H*), 3.44 (1H, t, *J* = 10.0, 9-H_A), 3.17 – 3.13 (1H, m, 11-H_A), 3.04 – 2.92 (2H, m, 3-H_B, 8-CHO*H*), 2.81 (1H, t, *J* = 10.2, 11-H_B), 2.70 (1H, t, *J* = 10.5, 9-H_B), 2.21 – 2.13 (1H, m, 8a-*H*), 2.10 (1H, ddd, *J* = 15.2, 6.2, 4.5, 7-H_A), 1.89 – 1.76 (6H, m, 1-$H_{A,B}$; 2-$H_{A,B}$; 7-H_B; 11a-*H*). **^{13}C NMR** (**Major diastereomer 50**, 125 MHz, $CDCl_3$): δ ppm 161.6 (5-*C*), 156.0 (*Ar*), 129.6 (*Ar*), 123.6 (*Ar*H), 113.5 (*Ar*H), 68.9 (8-*C*), 64.8 (11b-*C*), 62.0 (6a-*C*), 54.5 (ArO*C*H$_3$), 49.2 (9-*C*), 47.7 (11a-*C*), 47.5 (3-*C*), 45.2 (11-*C*), 45.1 (8a-*C*), 36.8 (1-*C*), 33.7 (7-*C*), 24.2 (2-*C*). **IR** ν_{max} (neat)/cm^{-1}: 3294 (O-H, N-H), 3051, 2927 (C-H), 1687 (C=O), 1611, 1512, 1461 (C=C), 1246 (C-O). **HRMS** (ESI): $C_{19}H_{26}N_3O_3$ [M + H$^+$]: calculated 344.1969, found 344.1964. **^{1}H NMR** (**Minor diastereomer 51**, 500 MHz, $CDCl_3$): δ ppm 7.08 (2H, d, *J* = 8.5, Ar*H*), 6.81 (2H, d, *J* = 9.0, Ar*H*), 4.12 (1H, dd, *J* = 9.0, 7.0, 6a-*H*), 3.95 (1H, ddd, *J* = 11.5, 7.0, 3.0, 8-*H*), 3.71 (3H, s, ArOCH_3), 3.55 (1H, dt, *J* = 11.3, 8.0, 3-H_A), 3.29 (1H, dd, *J* = 11.6, 5.9, 9-H_A), 3.25 – 3.18 (2H, m, 9-H_B, 11-H_A), 3.13 (1H, ddd, *J* = 11.5, 8.0, 5.0, 3-H_B), 3.01 (1H, t, *J* = 11.0, 11-H_B), 2.62 – 2.40 (4H, m, 8a-*H*, 11a-*H*, 10-N*H*, 8-CHO*H*), 2.04 – 1.91 (4H, m, 1-H_A; 2-$H_{A,B}$; 7-H_A), 1.84 – 1.75 (1H, m, 7-H_B), 1.69 -1.61 (1H, m, 1-H_B). **^{13}C NMR** (**Minor diastereomer 51**, 125 MHz, $CDCl_3$): δ ppm 159.6 (5-*C*), 155.9 (*Ar*), 129.5 (*Ar*), 123.7 (*Ar*H), 113.3 (*Ar*H), 64.7 (8-*C*), 63.1 (11b-*C*), 61.5 (6a-*C*), 54.5 (ArO*C*H$_3$), 49.6 (11-*C*), 47.0 (9-*C*), 45.9 (11a-*C*), 44.0 (3-*C*), 40.2 (1-*C*), 38.5 (8a-*C*), 30.6 (7-*C*), 24.3 (2-*C*). **IR** ν_{max} (neat)/cm^{-1}: 3353 (O-H, N-H), 2928 (C-H), 1681 (C=O), 1610, 1512, 1428 (C=C), 1246 (C-O). **HRMS** (ESI): $C_{19}H_{26}N_3O_3$ [M + H$^+$]: calculated 344.1969, found 344.1960.

(c) by functionalisation of ring-cleavage products (5 compounds)

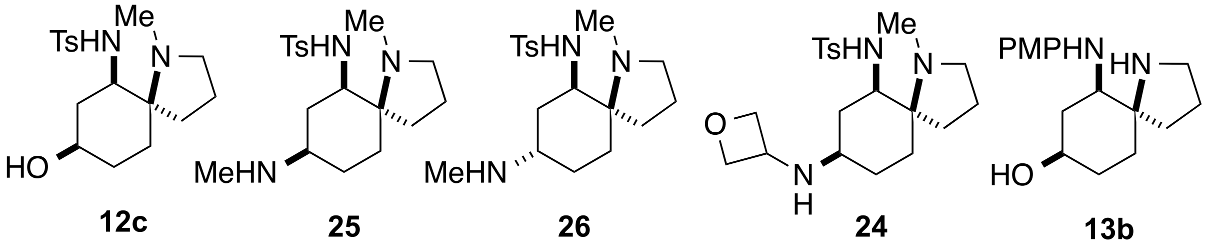

12c **25** **26** **24** **13b**

4-Methyl-*N*-[(5*S,6*R**)-1-methyl-8-oxo-1-azaspiro[4.5]decan-6-yl)benzenesulfonamide S7**

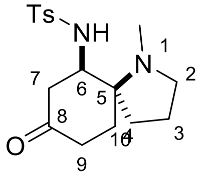

To a solution of the alcohol **12c** (200 mg, 0.59 mmol, 1.0 eq.) in 10.5 mL of acetone; 3.3 M Jones reagent [0.31 mL, 1.18 mmol, 2.0 eq.; freshly prepared by slowly adding 0.26 mL of 98% sulfuric acid to a stirring solution of chromium(VI) oxide (300 mg, 3.00 mmol) in 0.60 mL of water at 0 °C and then leaving the mixture to warm up to room temperature for 30 min] was added and the mixture was stirred at room temperature for 1 h 20 min. 5 mL EtOH was added to the reaction mixture and it was

basified with excess solid Na_2CO_3 to pH 8 as monitored by a strip of pH paper. It was then extracted with DCM (5 × 30 mL), dried over Na_2SO_4 and evaporated *in vacuo*. Flash chromatography with 1 – 3% MeOH in DCM afforded the product **S7** (60.0 mg, 0.18 mmol, 30% yield). **R_f** = 0.44 (10% MeOH in DCM). **^{1}H NMR** (500 MHz, $CDCl_3$): δ ppm 7.67 (2H, d, *J* = 8.3, Ar*H*), 7.23 (2H, d, *J* = 8.0, Ar*H*), 3.23 (1H, dd, *J* = 9.4, 4.9, 6-*H*), 2.82 (1H, ddd, *J* = 9.0, 7.0, 3.2, 2-H_A), 2.52 (1H, dd, *J* = 16.2, 9.4, 7-H_A), 2.49 – 2.43 (2H, m, 2-H_B, 7-H_B), 2.42 – 2.37 (1H, m, 9-H_A), 2.36 (3H, s, ArCH_3), 2.32 (3H, s, 1-CH_3), 2.18 (1H, ddd, *J* = 16.0, 10.5, 5.5, 9-H_B), 2.07 (1H, ddd, *J* = 15.5, 10.5, 5.0, 10-H_A), 1.69 – 1.61 (1H, m, 3-H_A), 1.59 – 1.52 (4H, m, 3-H_B; 4-$H_{A,B}$; 10-H_B). **^{13}C NMR** (125 MHz, $CDCl_3$): δ ppm 207.8 (8-*C*), 142.4 (*Ar*), 136.3 (*Ar*), 128.6 (*Ar*H), 126.1 (*Ar*H), 62.4 (5-*C*), 54.4 (2-*C*), 54.2 (6-*C*), 43.0 (7-*C*), 36.9 (1-*C*), 36.6 (9-*C*), 35.7 (4-*C*), 25.2 (10-*C*), 21.0 (3-*C*), 20.5 (Ar*C*H$_3$). **IR** ν_{max} (neat)/cm^{-1}: 3261 (N-H), 2927, 2796 (C-H), 1672 (C=O), 1598, 1494, 1449 (C=C). **HRMS** (ESI): $C_{17}H_{25}N_2O_3S$ [M + H$^+$]: calculated 337.1580, found 337.1576.

4-Methyl-*N*-[(5*S,6*R**,8*R**)-1-methyl-8-(oxoetan-3-ylamino)-1-azaspiro[4.5]decan-6-yl)benzenesulfonamide 24**

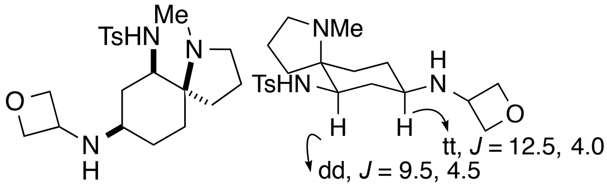

(Assignment of stereochemistry: analysis of J values)

To a solution of the ketone **S7** (15.0 mg, 0.04 mmol, 1.0 eq.) in 3 mL MeOH, 3-aminooxetane (0.01 mL, 0.14 mmol, 3.2 eq.) and titanium isopropoxide (0.03 mL, 0.09 mmol, 2.0 eq.) were added and the mixture was left to stir at room temperature overnight. $NaBH_4$ (2.60 mg, 0.07 mmol, 1.5 eq.) was then added to the reaction mixture at -78 °C and it was stirred at the same temperature for 30 min. It was then allowed to warm up to room temperature for another 30 min. The reaction mixture was evaporated *in* vacuo. It was then taken up in 1 mL H_2O, diluted with 100 mL EtOAc, dried over Na_2SO_4 and evaporated *in vacuo*. Flash chromatography with 1 – 4% MeOH in DCM afforded the product as a 78:22 mixture of diastereomers (3.60 mg, 0.01 mmol, 21% yield). **R_f** = 0.42 (5% MeOH in DCM). **^{1}H NMR** (Major diastereomer, 500 MHz, $CDCl_3$): δ ppm 7.69 (2H, d, *J* = 8.3, Ar*H*), 7.21 (2H, d, *J* = 8.1, Ar*H*), 4.71 (1H, t, *J* = 6.8, 2'-H_A), 4.67 (1H, t, *J* = 6.8, 2''-H_A), 4.31 – 4.26 (2H, m, 2'-H_B, 2''-H_B), 3.88 (1H, quint, *J* = 6.6, 3'-*H*), 3.06 (1H, dd, *J* = 9.5, 4.5, 6-*H*), 2.83 – 2.76 (1H, m, 2-H_A), 2.51 (1H, tt, *J* = 12.5, 4.0, 8-*H*), 2.44 (1H, dt, *J* = 16.0, 8.0, 2-H_B), 2.35 (3H, s, ArCH_3), 2.34 (3H, s, 1-CH_3), 2.15 (1H, s, 8-TsN*H*), 1.95 (1H, ddd, *J* = 14.6, 5.7, 4.3, 4-H_A), 1.69 – 1.66 (1H, m, 7-H_A), 1.54 – 1.49 (4H, m, 3-H_A, 9-H_A, 10-$H_{A,B}$), 1.40 – 1.38 (2H, m, 3-H_B, 7-H_B), 1.34 – 1.30 (1H, m, 9-H_B), 1.16 – 1.08 (1H, m, 4-H_B). Signal for minor isomer visible at: 3.81 (0.29H, quint, *J* = 6.5, 3'-H). **^{13}C NMR** (Major diastereomer, 125 MHz,

$CDCl_3$): δ ppm 141.8 (*Ar*), 137.9 (*Ar*), 128.4 (*Ar*H), 125.8 (*Ar*H), 79.3 (2'-*C*), 79.1 (2''-*C*), 60.7 (5-*C*), 56.8 (6-*C*), 55.7 (2-*C*), 51.5 (8-*C*), 50.2 (3'-*C*), 39.0 (9-*C*), 37.8 (1-*C*), 29.9 (7-*C*), 29.1 (10-*C*), 27.5 (4-*C*), 22.0 (3-*C*), 20.5 (Ar*C*H_3). Signals for minor isomer visible at: 142.3, 140.2, 128.5, 126.4, 66.1, 31.1. **IR** ν_{max} (neat)/cm^{-1}: 3294 (N-H), 2951, 2868, 2794 (C-H), 1598, 1513, 1454 (C=C), 1160 (C-O). **HRMS** (ESI): $C_{20}H_{32}N_3O_3S$ [M + H^+]: calculated 394.2159, found 394.2139.

4-Methyl-*N*-[(5*S,6*R**,8*R**)-1-methyl-8-(methylamino)-1-azaspiro[4.5]decan-6-yl)benzenesulfonamide 25** and **4-methyl-*N*-[(5*S**,6*R**,8*S**)-1-methyl-8-(methylamino)-1-azaspiro[4.5]decan-6-yl)benzenesulfonamide 26**

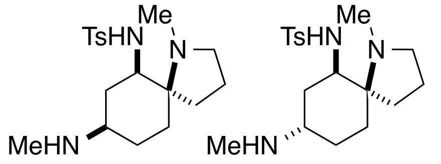

25 **26**

*(Assignment of stereochemistry: by analogy with **24**)*

To a mixture of the ketone **S7** (20.0 mg, 0.06 mmol, 1.0 eq.) and STAB (19.1 mg, 0.09 mmol, 1.5 eq.) in 5 mL THF, 2M $MeNH_2$ in MeOH (0.30 mL, 0.60 mmol, 10.0 eq.) and AcOH (0.01 mL, 0.17 mmol, 2.9 eq.) were added and the mixture was stirred at room temperature overnight. 2 mL of a saturated aqueous solution of Na_2CO_3 was then added to the reaction mixture and it was evaporated *in vacuo*. It was then taken up in 1 mL of H_2O, diluted with 100 mL DCM, dried over Na_2SO_4, and evaporated *in vacuo*. To a solution of the crude in 5 mL THF, cyclopropyl isocyanate (0.01 mL, 0.12 mmol, 2.0 eq.) was added and the mixture was refluxed for 30 h. The reaction mixture was evaporated *in vacuo*. Flash chromatography eluting with 2% MeOH in DCM followed by 2 – 10% MeOH in DCM afforded the intermediate amines **25** [7 mg, 0.02 mmol, 33% yield, $\mathbf{R_f}$ = 0.50 (7% of saturated NH_3/MeOH in DCM)] and **26** [4 mg, 0.01 mmol, 19% yield, $\mathbf{R_f}$ = 0.41 (7% of saturated NH_3/MeOH in DCM)] as products. **^{1}H NMR** (**Compound 25**, 500 MHz, $CDCl_3$): δ ppm 7.71 (2H, d, *J* = 8.0, Ar*H*), 7.20 (2H, d, *J* = 8.0, Ar*H*), 3.14 (1H, dd, *J* = 7.6, 4.2, 6-*H*), 2.84 – 2.76 (1H, m, 2-H_A), 2.52 – 2.40 (2H, m, 2-H_B, 8-*H*), 2.35 (3H, s, ArCH_3), 2.33 (3H, s, 1-CH_3), 2.26 (3H, s, NHCH_3), 1.96 (1H, ddd, *J* = 13.0, 8.5, 4.0, 4-H_A), 1.68 – 1.35 (10H, m, TsN*H*, N*H*CH_3, 3-$H_{A,B}$; 7-$H_{A,B}$; 9-$H_{A,B}$; 10-$H_{A,B}$), 1.11 (1H, ddd, *J* = 13.5, 8.0, 4.5, 4-H_B). **^{13}C NMR** (**Compound 25**, 125 MHz, $CDCl_3$): δ ppm 142.6 (*Ar*), 139.0 (*Ar*), 129.3 (*Ar*H), 127.0 (*Ar*H), 62.2 (5-*C*), 58.4 (6-*C*), 56.3 (2-*C*), 55.5 (8-*C*), 38.8 (9-*C*, **missing but observed by HMQC**) 38.1 (1-*C*), 34.1 (7-*C*, **missing but observed by HMQC**), 33.9 (NH*C*H_3), 29.2 (10-*C*), 25.9 (4-*C*, **missing but observed by HMQC**), 22.6 (3-*C*), 21.5 (Ar*C*H_3). **IR** ν_{max} (neat)/cm^{-1}: 3324 (N-H), 2941, 2866, 2791 (C-H), 1598, 1541, 1450 (C=C). **HRMS** (ESI): $C_{18}H_{30}N_3O_2S$ [M + H^+]: calculated 352.2053, found 352.2054. **^{1}H NMR** (**Compound 26**, 500 MHz, $CDCl_3$): δ ppm 7.69 (2H, d, *J* = 8.5, Ar*H*), 7.23 (2H, d, *J* = 8.0, Ar*H*), 2.69 (1H,

t, J = 3.0, 6-H), 2.59 (1H, tt, J = 11.0, 4.0, 8-H), 2.35 (3H, s, ArCH_3), 2.33 – 2.25 (3H, m, 2-$H_{A,B}$; 7-H_A), 2.23 (3H, s, NHCH_3), 2.17 (3H, s, 1-CH_3), 1.84 – 1.78 (1H, m, 9-H_A), 1.75 – 1.64 (3H, m, 3-H_A, 4-H_A, 10-H_A), 1.42 – 1.28 (4H, m, TsNH, 3-H_B, 4-H_B, 10-H_B), 1.16 (1H, ddd, J = 14.0, 11.0, 3.1, 7-H_B), 1.00 (1H, qd, J = 13.0, 3.5, 9-H_B). **^{13}C NMR** (**Compound 26**, 125 MHz, $CDCl_3$): δ ppm 143.1 (*Ar*), 136.6 (*Ar*), 129.5 (*Ar*H), 127.4 (*Ar*H), 67.1 (5-*C*), 54.0 (6-*C*), 53.8 (2-*C*), 52.2 (8-*C*), 38.5 (1-*C*), 33.6 (NHCH_3), 32.42 (7-*C*), 32.38 (10-*C*), 30.0 (9-*C*), 28.4 (4-*C*), 22.3 (3-*C*), 21.5 (ArCH_3). **IR** ν_{max} (neat)/cm^{-1}: 3158 (N-H), 2939, 2862, 2792 (C-H), 1598, 1512, 1447 (C=C). **HRMS** (ESI): $C_{18}H_{30}N_3O_2S$ [M + H$^+$]: calculated 352.2053, found 352.2062.

(d) by functionalisation of ring expansion products (10 compounds)

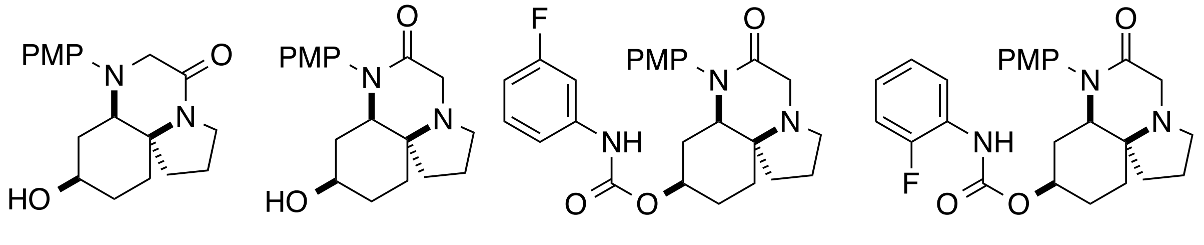

15b **16b** **30** **52**

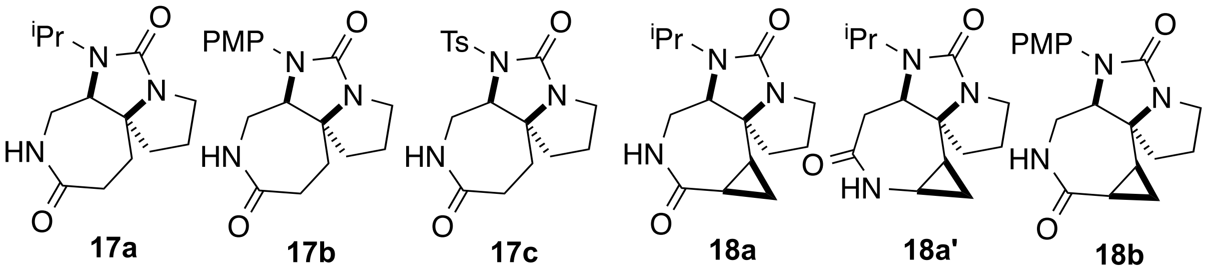

17a **17b** **17c** **18a** **18a'** **18b**

(7a*R,9*R**,11a*S**)-7-(4-Methoxyphenyl)-6-oxodecahydro-1H-pyrrolo[1,2-*d*]quinoxalin-9-yl (2-fluorophenyl)carbamate 30**

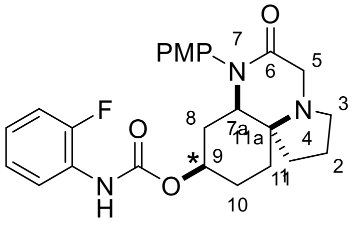

To a solution of the alcohol **16b** (27.0 mg, 0.09 mmol, 1.0 eq.) in 4 mL DCM, 2-fluorophenylisocyanate (0.01 mL, 0.09 mmol, 1.1 eq.) and TEA (0.04 mL, 0.26 mmol, 3.0 eq.) were added and the mixture was stirred at room temperature overnight. The reaction mixture was evaporated *in vacuo*. Flash chromatography with 1 – 8% MeOH in DCM afforded the product **30** (33.0 mg, 0.07 mmol, 86% yield) **R$_f$** = 0.29 (4% MeOH in DCM). **^{1}H NMR** (500 MHz, $CDCl_3$): δ ppm 7.93 (1H, t, J = 8.3, ArH), 7.09 (2H, d, J = 9.0, ArH), 7.07 – 6.99 (2H, m, ArH), 6.96 – 6.91 (1H, m, ArH), 6.80 (2H,

d, J = 9.0, ArH), 6.78 (1H, s, NH), 4.70 (1H, tt, J = 8.2, 4.2, 9-H), 3.85 (1H, d, J = 18.0, 5-H_A), 3.72 (1H, dd, J = 8.5, 5.6, 7a-H), 3.70 (3H, s, ArOCH_3), 3.38 (1H, d, J = 18.0, 5-H_B), 3.15 – 3.05 (1H, m, 3-H_A), 2.97 – 2.85 (1H, m, 3-H_B), 2.20 – 2.00 (2H, m, 1-H_A, 8-H_A), 2.00 – 1.67 (7H, m,2-$H_{A,B}$; 8-H_B; 10-$H_{A,B}$; 11-$H_{A,B}$), 1.30 – 1.20 (1H, m, 1-H_B). **^{13}C NMR** (125 MHz, $CDCl_3$): δ ppm 168.3 (6-*C*), 157.3 (carbamate *C*=O), 151.5 (*Ar*), 151.2 (d, J = 242, *Ar*F), 132.3 (*Ar*), 126.6 (*Ar*H), 125.2 (d, J =9.75, *Ar*), 123.6 (d, J = 3.63, *Ar*H), 122.5 (d, J = 6.38, *Ar*H), 119.2 (*Ar*H), 113.9 (d, J = 18.8, *Ar*H), 113.5 (*Ar*H), 69.6 (9-*C*), 61.0 (11a-*C*), 60.0 (7a-*C*), 54.4 (ArO*C*H_3), 54.3 (3-*C*), 53.0 (5-*C*), 35.2 (10-*C*), 32.5 (8-*C*), 28.2 (1-*C*), 26.4 (11-*C*), 21.6 (2-*C*). **IR** ν_{max} (neat)/cm^{-1}: 3253 (N-H); 2955, 2836 (C-H); 1723, 1656 (C=O); 1619, 1536, 1510, 1456 (C=C); 1229 (C-O). **HRMS** (ESI): $C_{25}H_{29}FN_3O_4$ [M + H$^+$]: calculated 454.2137, found 454.2145.

(7a*R,9*R**,11a*S**)-7-(4-Methoxyphenyl)-6-oxodecahydro-1H-pyrrolo[1,2-*d*]quinoxalin-9-yl (3-fluorophenyl)carbamate 52**

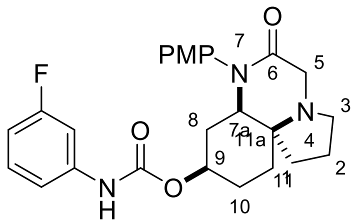

To a solution of the alcohol **16b** (22.0 mg, 0.07 mmol, 1.0 eq.) in 5 mL DCM, 3-fluorophenylisocyanate (0.01 mL, 0.09 mmol, 1.3 eq.) and TEA (0.03 mL, 0.21 mmol, 3.0 eq.) were added and the mixture was stirred at room temperature overnight. The reaction mixture was evaporated *in vacuo*. Flash chromatography with 1 – 4% MeOH in DCM afforded the product **52** (23.0 mg, 0.05 mmol, 73% yield). **R_f** = 0.37 (4% MeOH in DCM). **^{1}H NMR** (500 MHz, $CDCl_3$): δ ppm 7.33 (1H, br.s, ArH), 7.15 (1H, dt, J = 11.0, 2.3, ArH), 7.10 (2H, d, J = 9.5, ArH), 6.86 (1H, dd, J = 8.0, 1.5, ArH), 6.76 (2H, d, J = 9.0, ArH), 6.65 (1H, td, J = 8.3, 2.4, ArH), 4.73 (1H, tt, J = 7.0, 4.0, 9-H), 3.86 (1H, d, J = 18.0, 5-H_A), 3.75 (1H, dd, J = 8.0, 3.8, 7a-H), 3.66 (3H, s, ArOCH_3), 3.43 (1H, d, J = 17.5, 5-H_B), 3.14 – 3.04 (1H, m, 3-H_A), 3.00 – 2.90 (1H, m, 3-H_B), 2.14 – 2.08 (1H, m, 11-H_A), 2.07 – 1.84 (8H, m, 1-$H_{A,B}$; 2-$H_{A,B}$; 8-$H_{A,B}$; 10-$H_{A,B}$), 1.28 (1H, ddd, J = 14.0, 8.5, 3.5, 11-H_B). **^{13}C NMR** (125 MHz, $CDCl_3$): δ ppm 167.8 (6-*C*), 162.1 (d, J = 243, *Ar*F), 157.4 (*Ar*), 151.6 (carbamate *C*=O), 138.7 (d, J = 11.0, *Ar*), 113.9 (*Ar*), 129.0 (d, J = 1.88, *Ar*H), 126.8 (*Ar*H), 113.4 (*Ar*H), 112.9 (*Ar*H), 108.8 (d, J = 21.3, *Ar*H), 105.0 (d, J = 26.4, *Ar*H), 68.7 (9-*C*), 61.0 (11a-*C*), 59.5 (7a-*C*), 54.3 (ArO*C*H_3), 53.4 (3-*C*), 52.3 (5-*C*), 34.9 (10-*C*), 31.9 (8-*C*), 26.5 (1-*C*, 11-*C*, **confirmed from HMQC**), 21.3 (2-*C*). **IR** ν_{max} (neat)/cm^{-1}: 3268 (N-H); 2952 (C-H); 1723, 1642 (C=O); 1605, 1544, 1510 (C=C); 1223 (C-O). **HRMS** (ESI): $C_{25}H_{29}FN_3O_4$ [M + H$^+$]: calculated 454.2137, found 454.2141.

Molecular property analysis of screening compounds

The molecular properties of the screening library were analysed using our previously described Lead-Likeness And Molecular Analysis (LLAMA) toolkit.[5] Where a compound was isolated as an inseparable mixture of diastereoisomers, the structure of the major diastereomer was used as the input. The 'lead-likeness' parameter[5] plot shows that 66% of compounds are within 'lead-like' space.

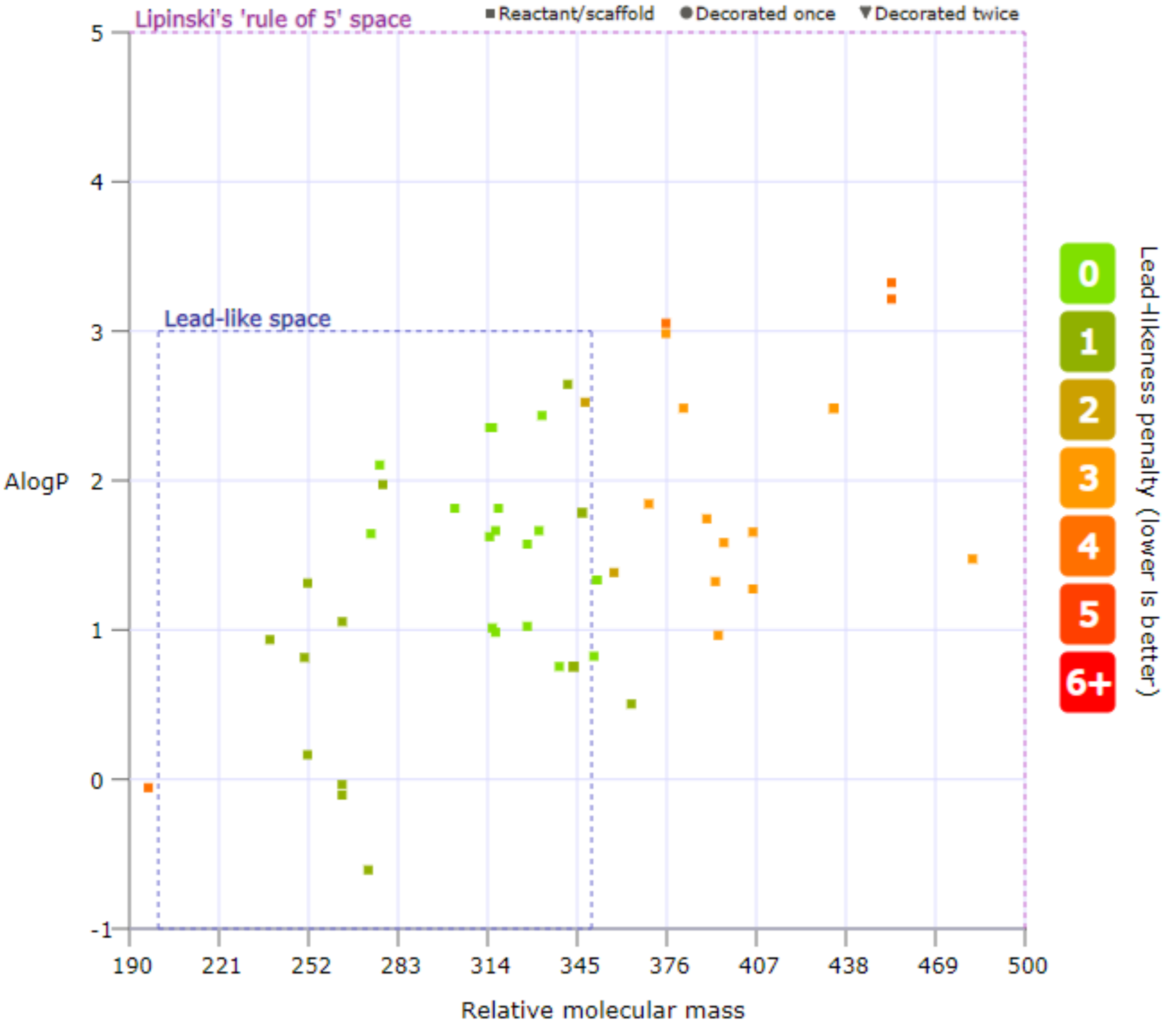

Of those that fall outside the 'lead-like' space, all do so on the basis of the molecular weight exceeding the 'lead-like' limits, while three of the 51 compounds ALSO exceed the lipophicity (AlogP) limit of 3. The library is therefore not excessively lipophilic.

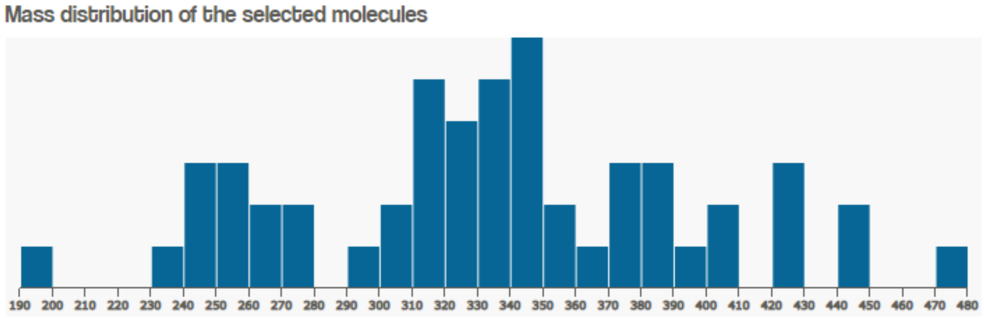

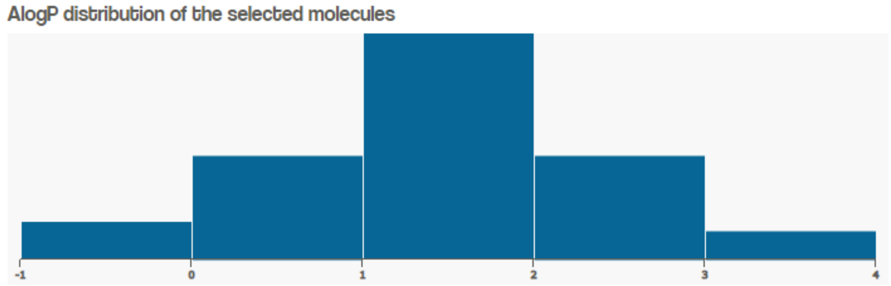

Analysis of Fsp[3] showed the distribution below, with a mean Fsp[3] of 0.68.

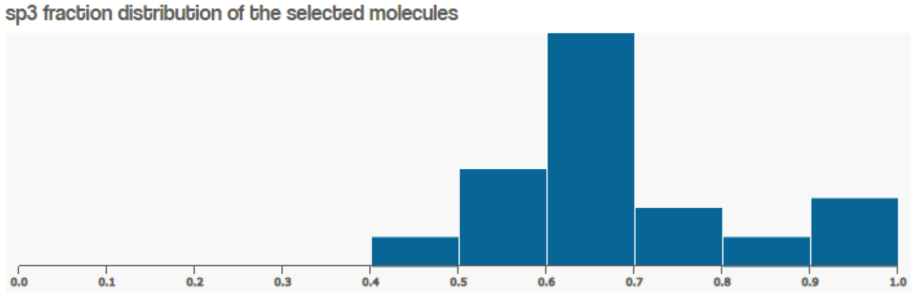

Shape analysis for the library is shown below, using a PMI plot.

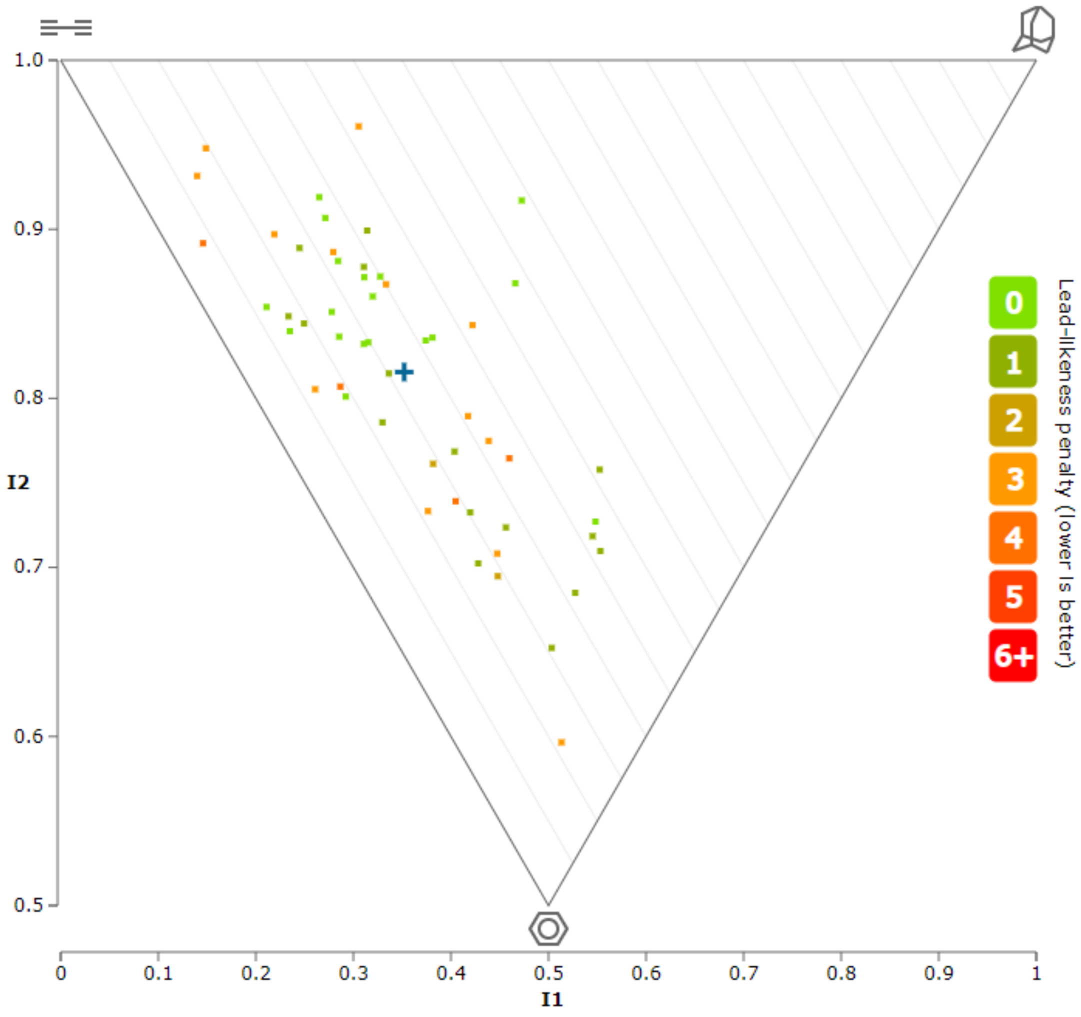

^{1}H and ^{13}C Spectra

1-(Benzyloxy)-4-iodobenzene S1

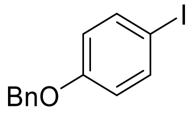

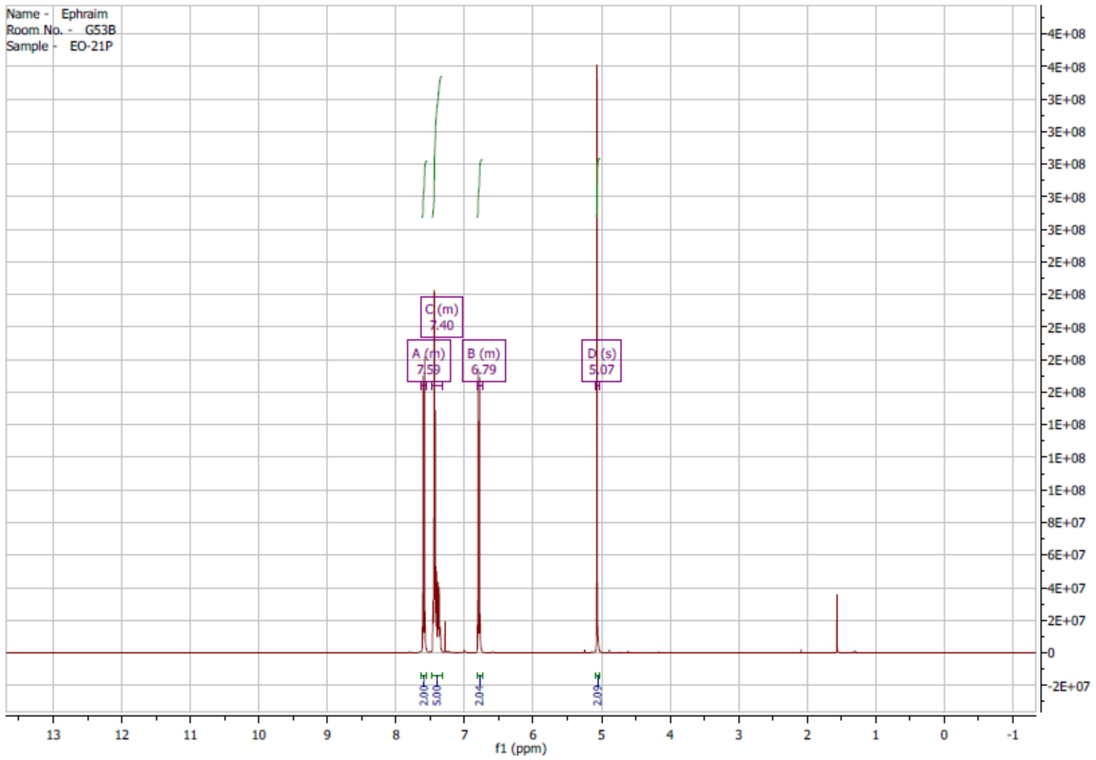

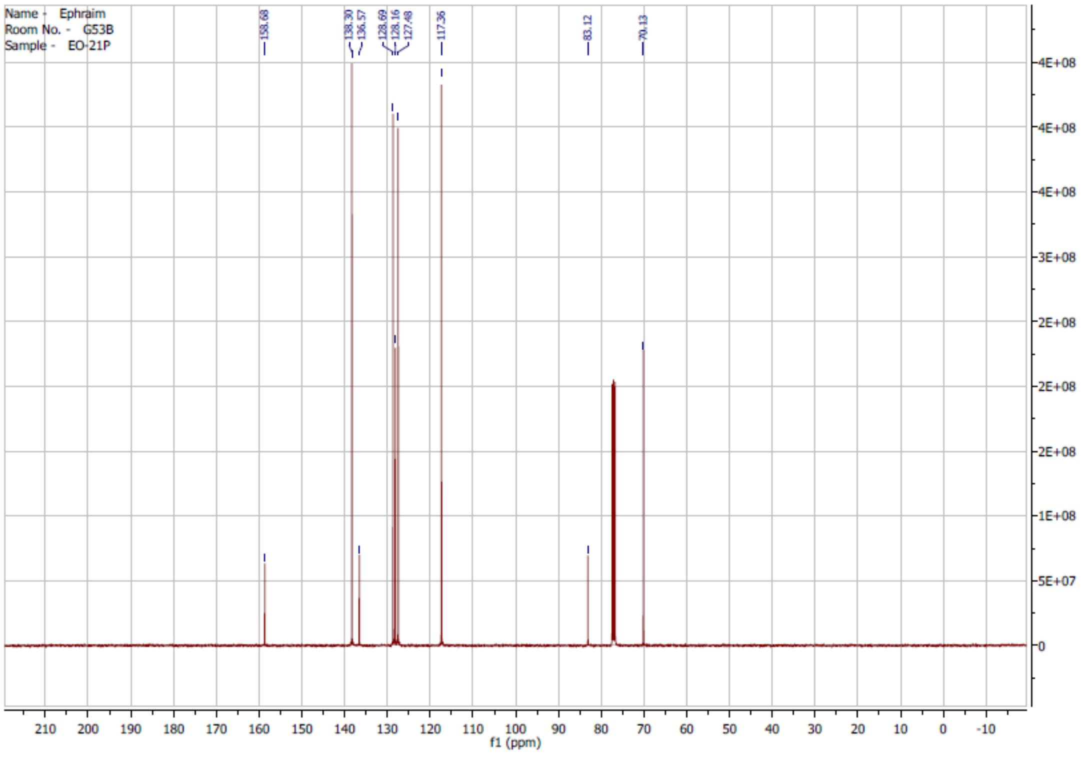

3-[(4-Benzyloxy)phenyl]propanal S2

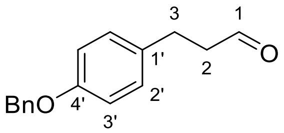

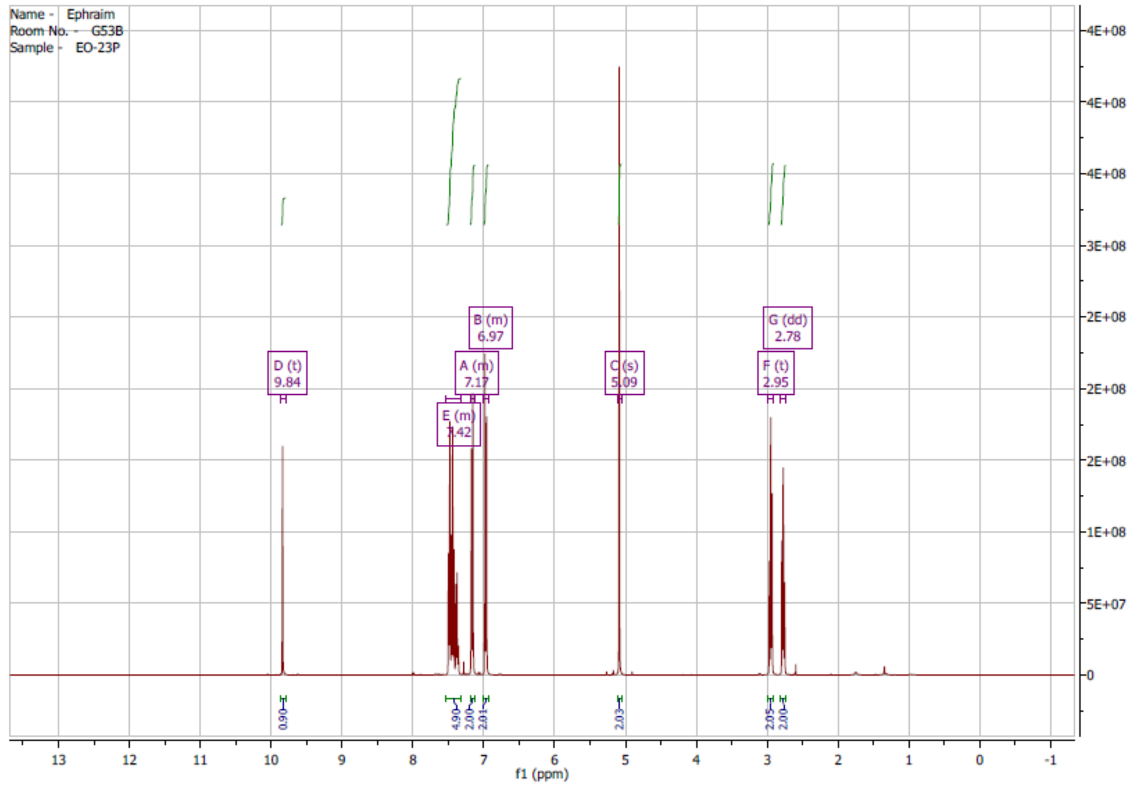

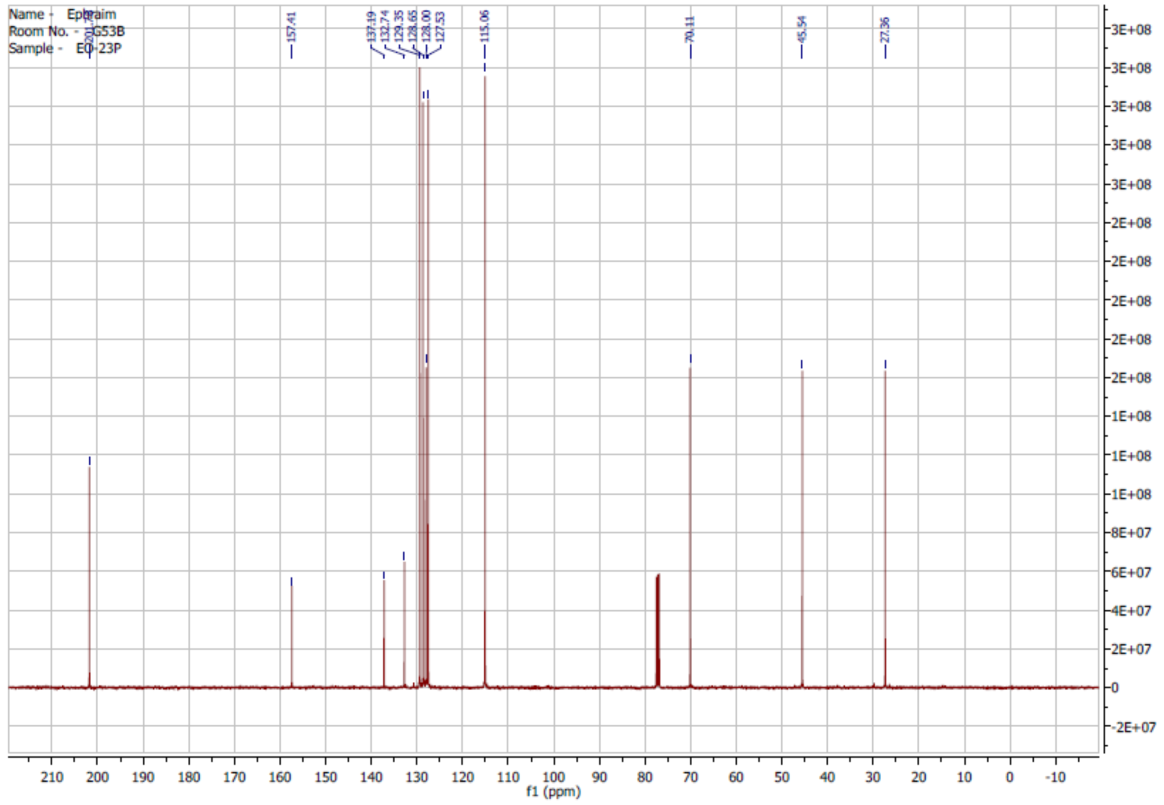

3-(4-(Benzyloxy)phenyl)propan-1-amine S3

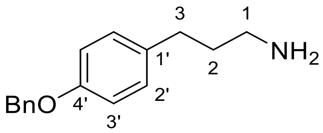

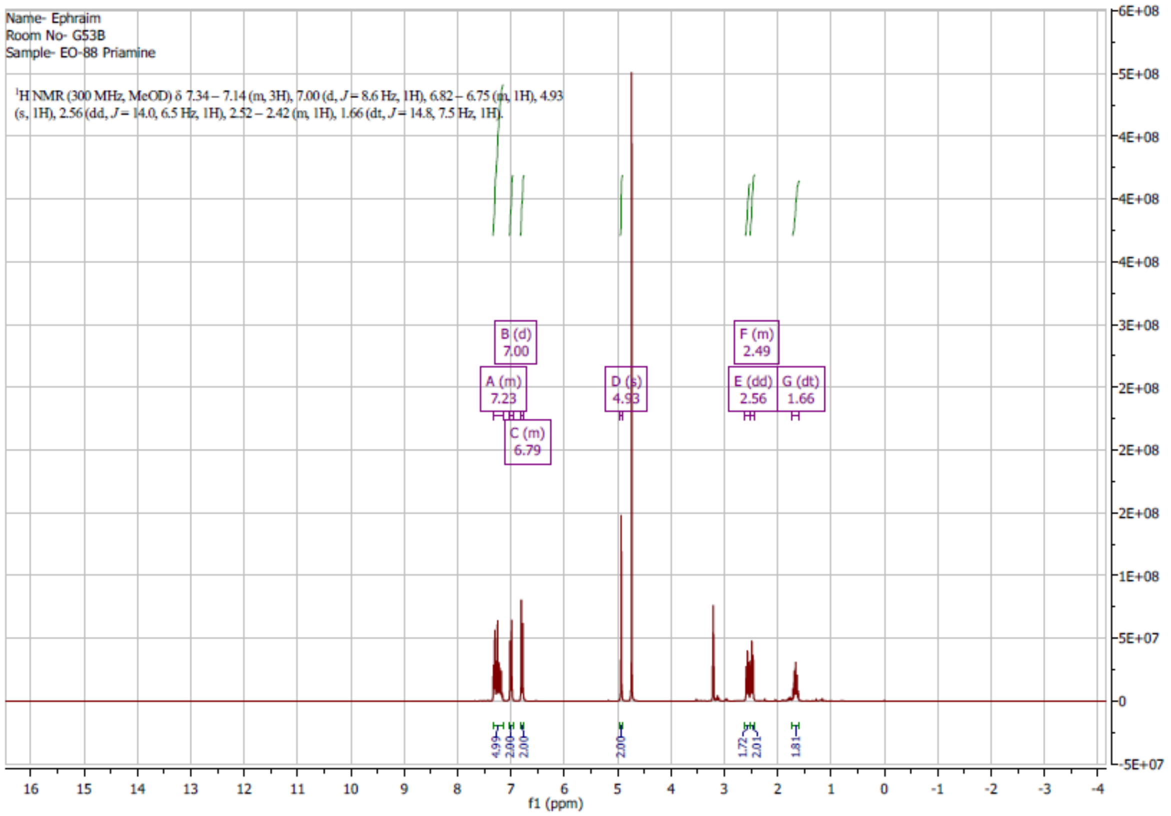

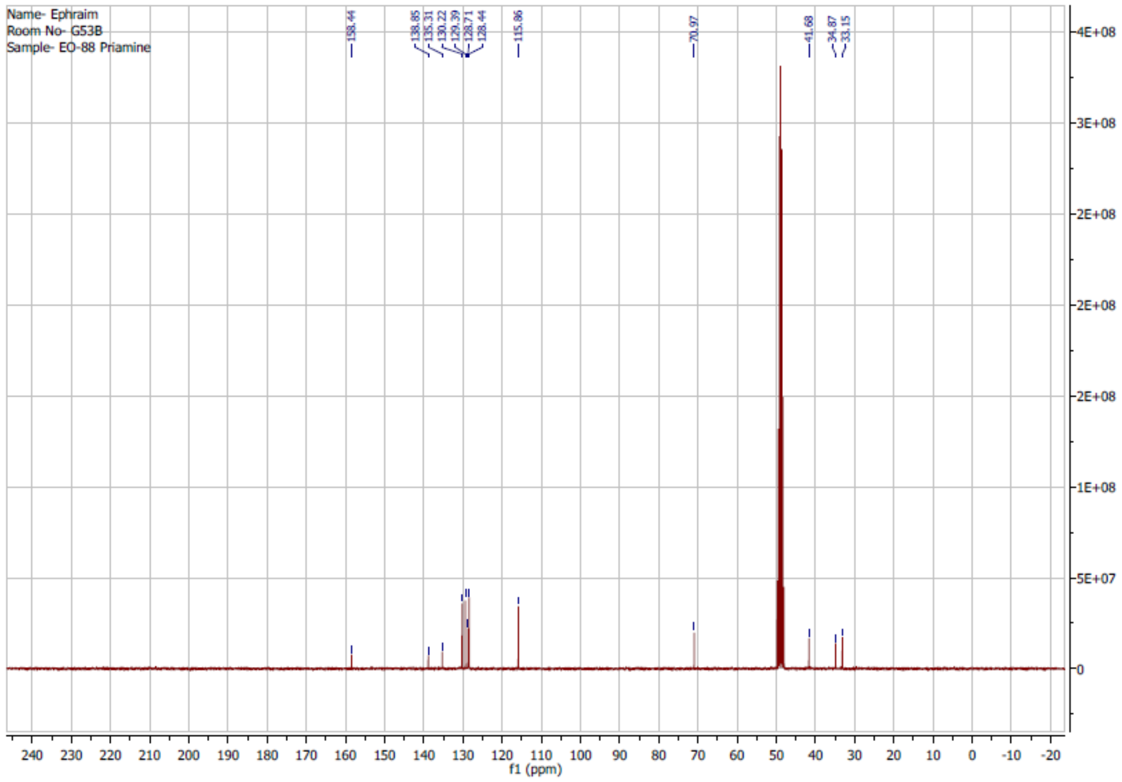

1-(3-(4-(Benzyloxy)phenyl)propyl)-3-isopropylurea S4

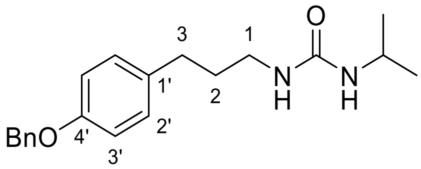

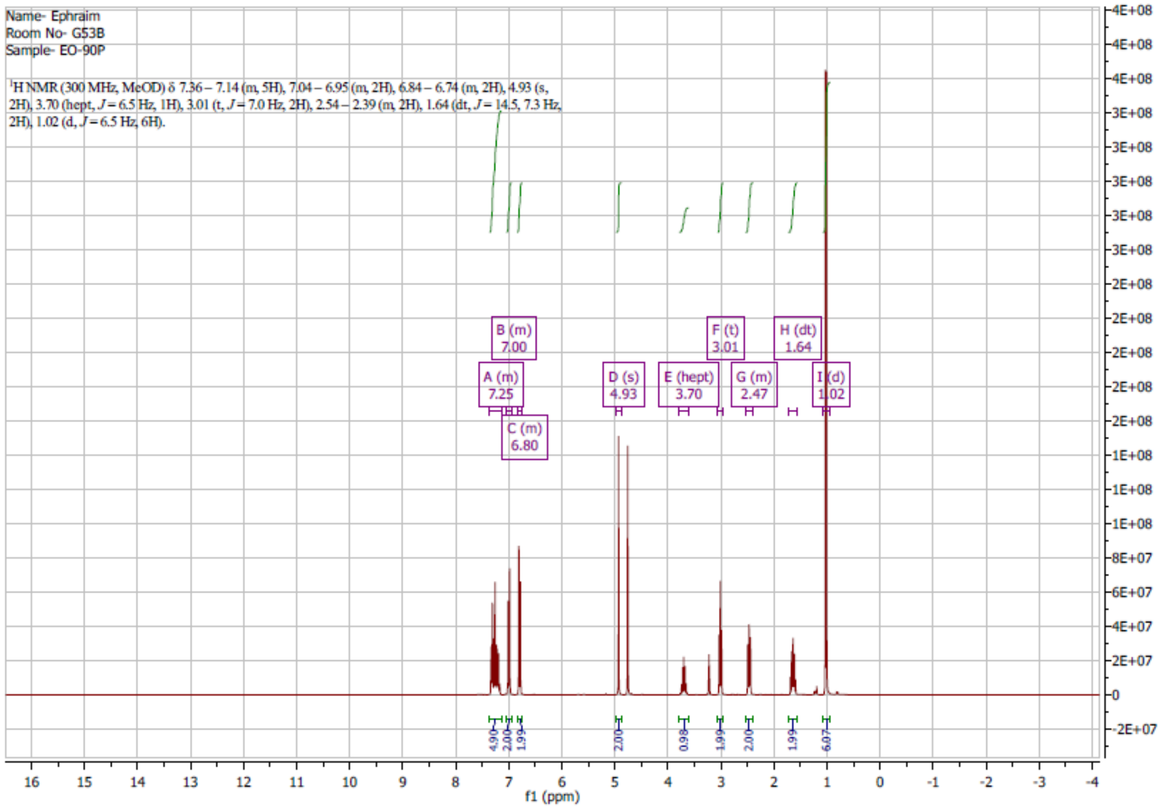

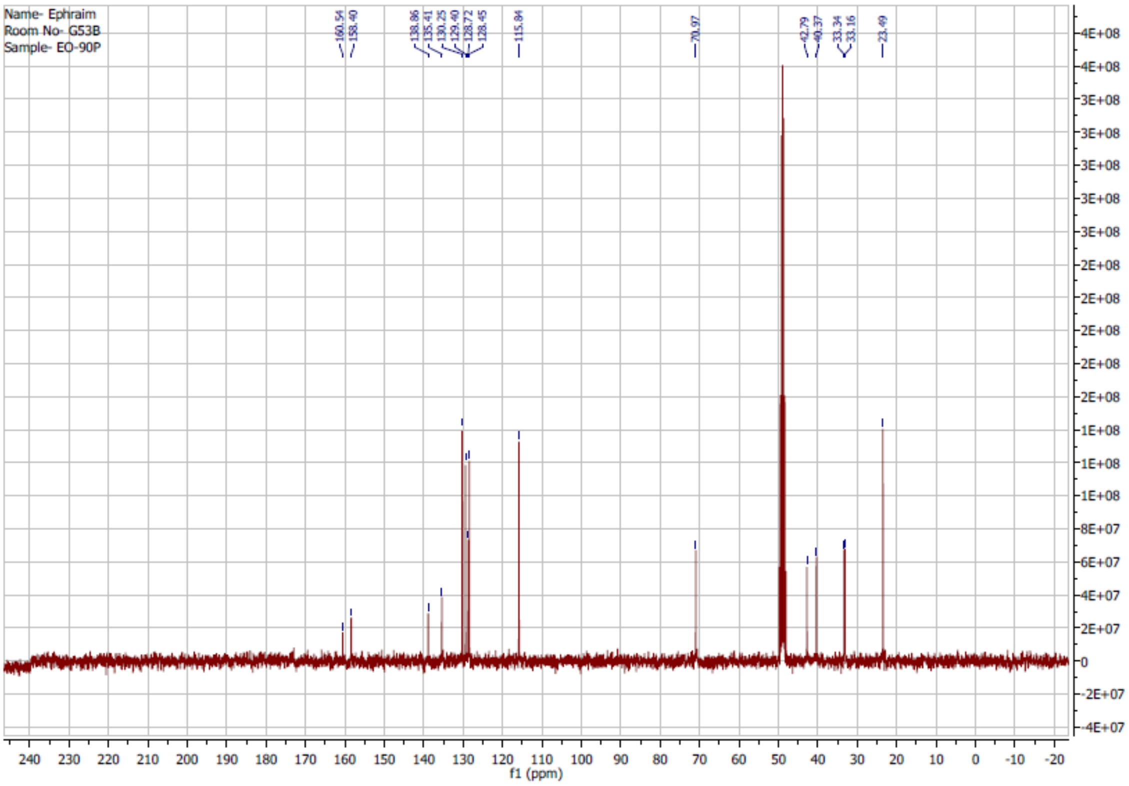

1-[3-(4-Hydroxyphenyl)propyl]-3-isopropylurea 1a

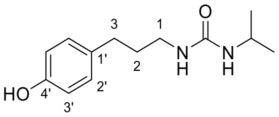

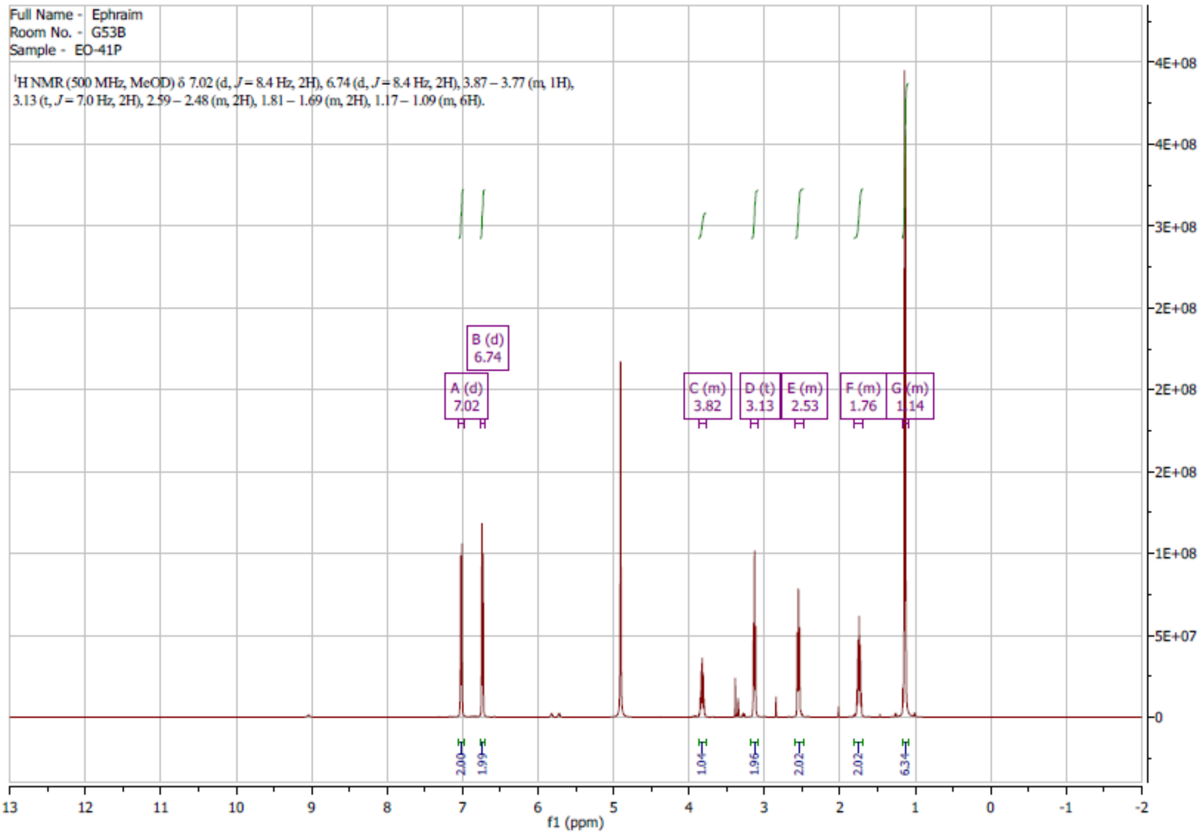

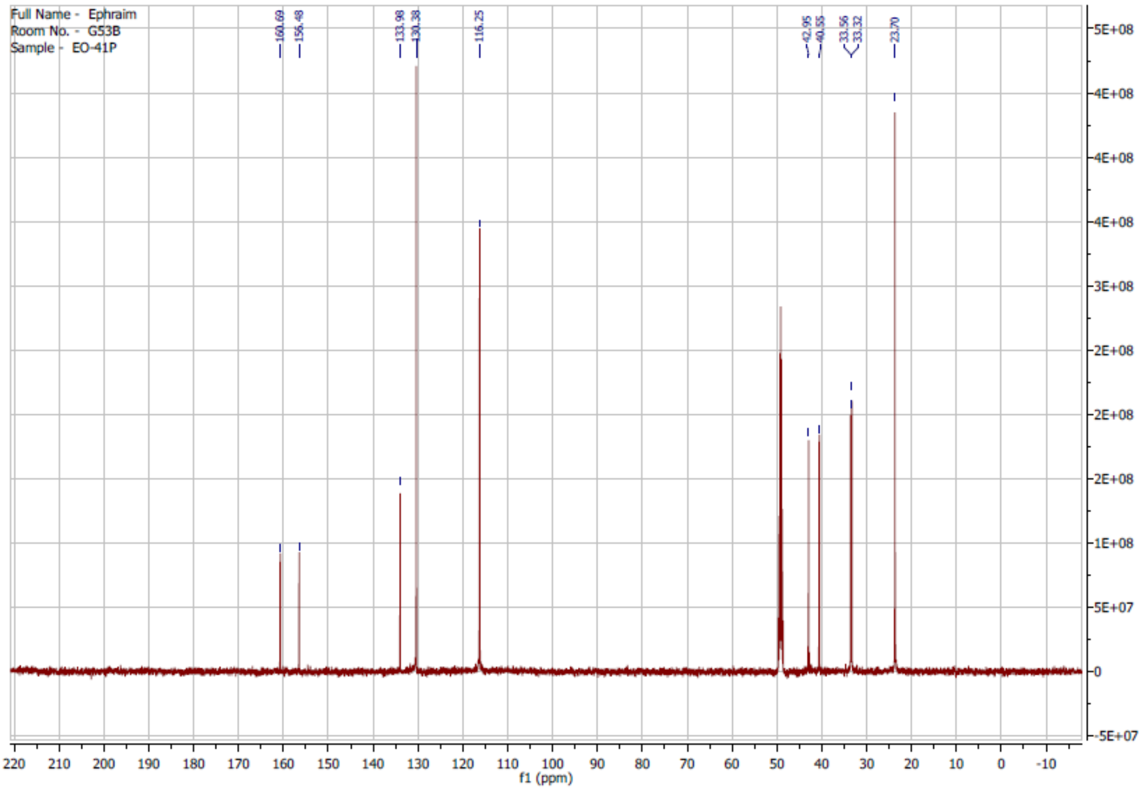

***N*-Benzyl-3-[4-(benzyloxy)phenyl]propan-1-amine S5**

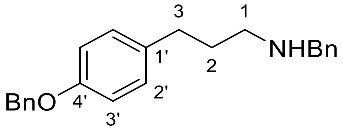

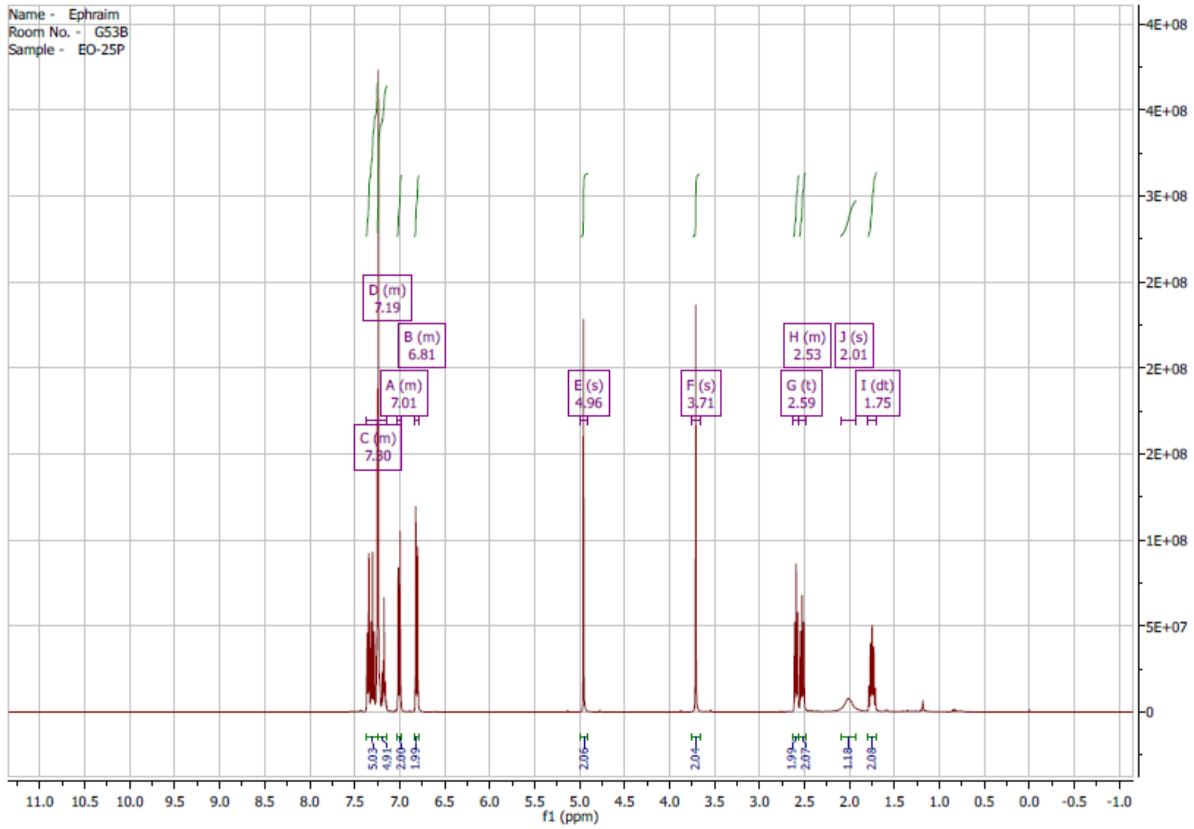

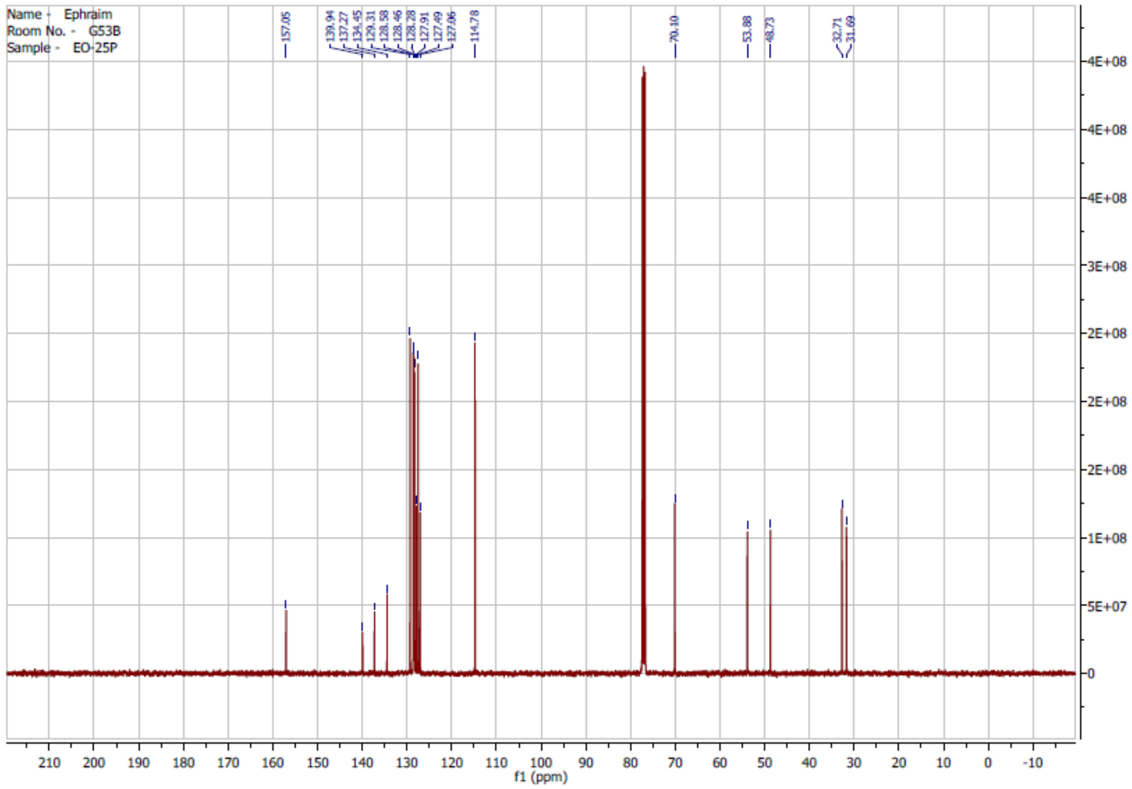

4-(3-Aminopropyl)phenol S6

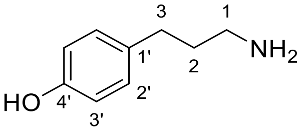

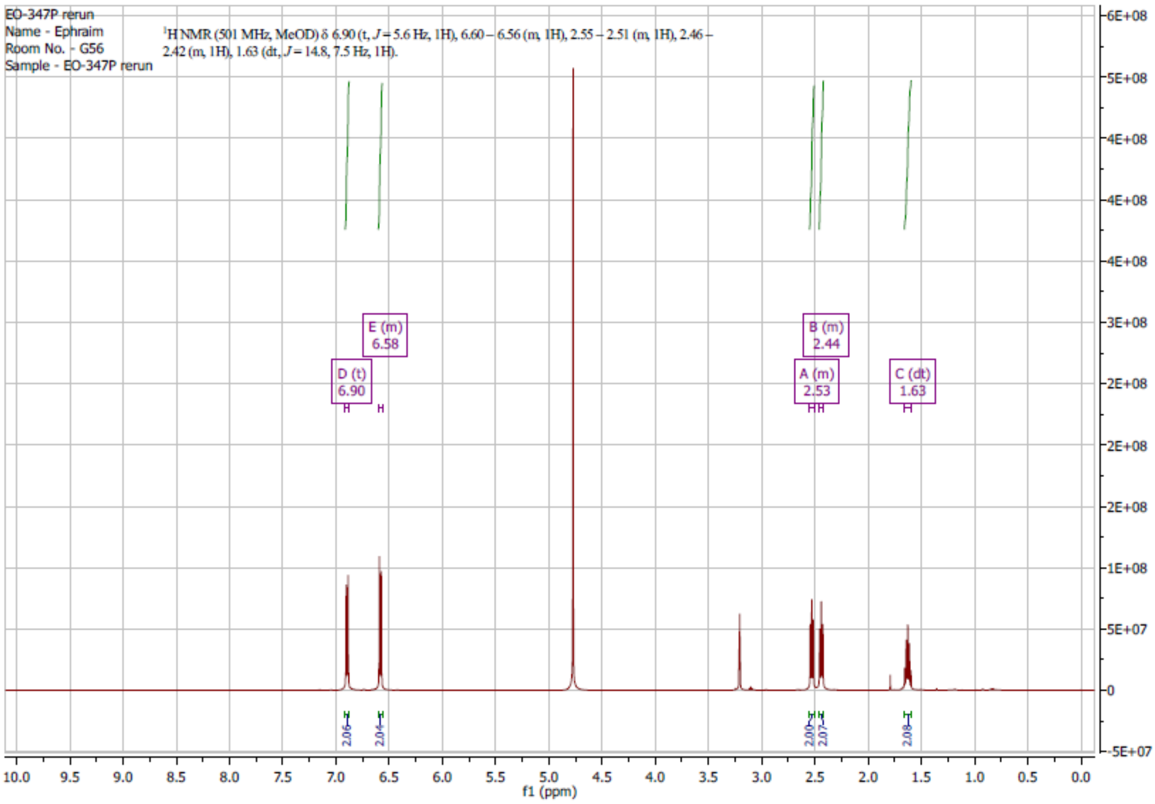

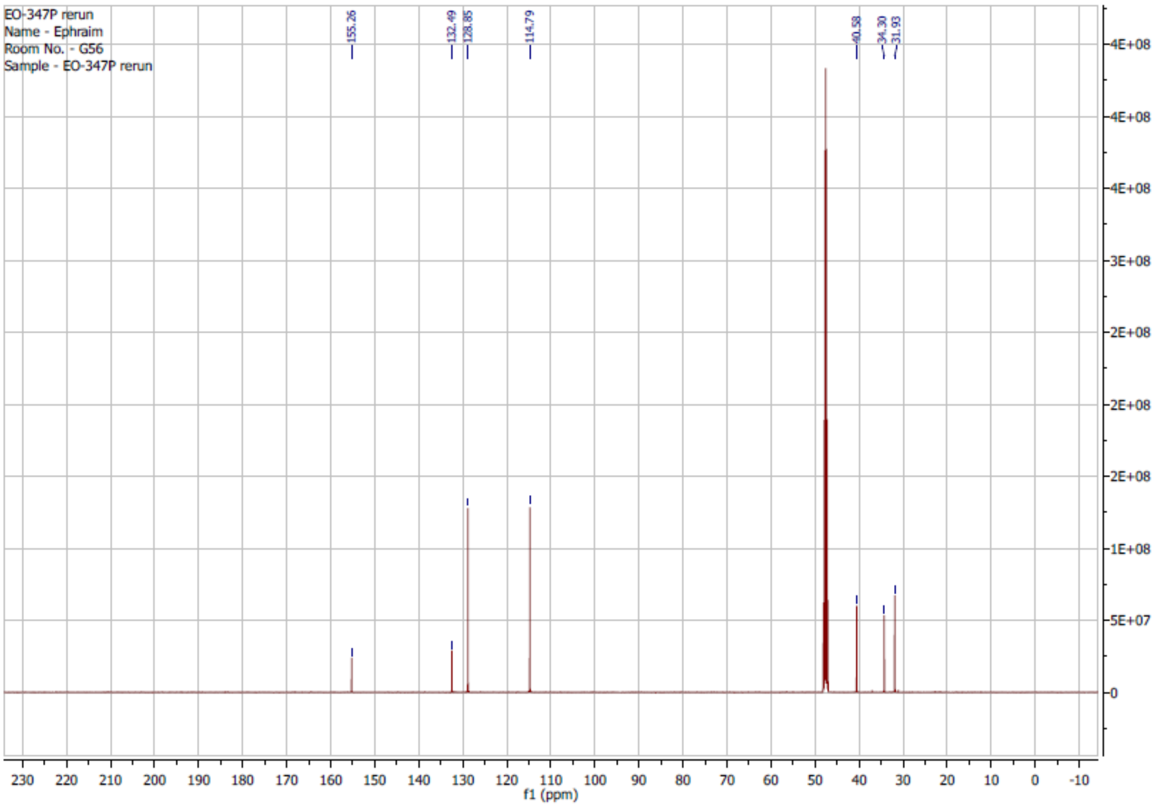

1-(3-(4-Hydroxyphenyl)propyl)-3-(4-methoxyphenyl)urea 1b

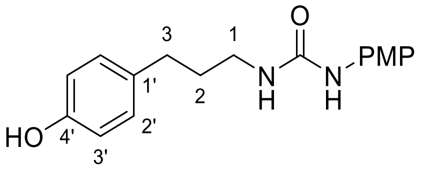

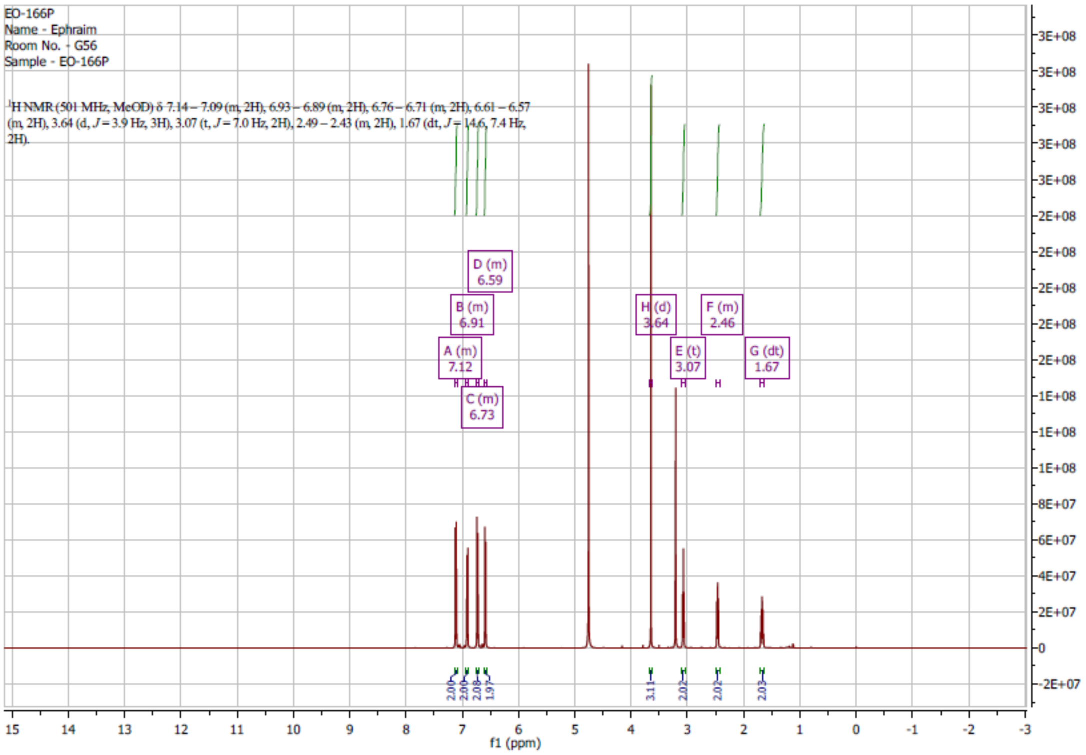

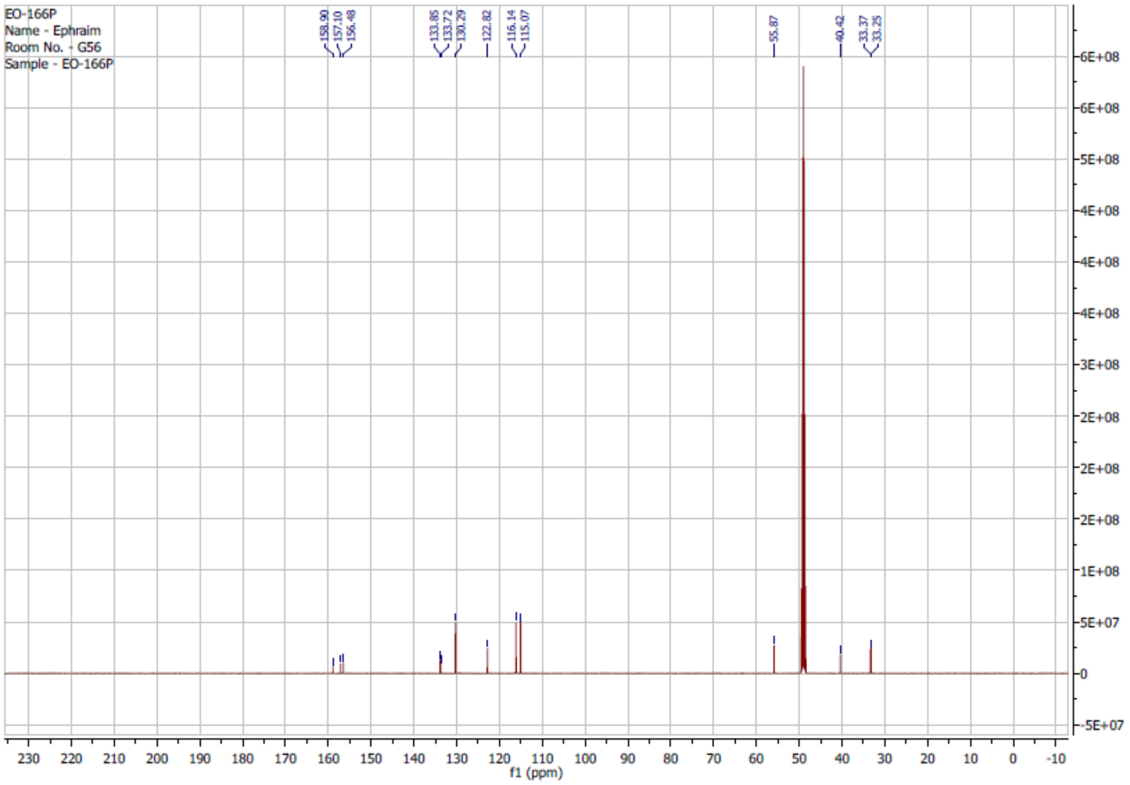

N-((3-(4-Hydroxyphenyl)propyl)carbamoyl)-4-methylbenzenesulfonamide 1c

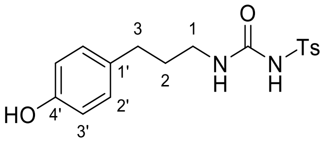

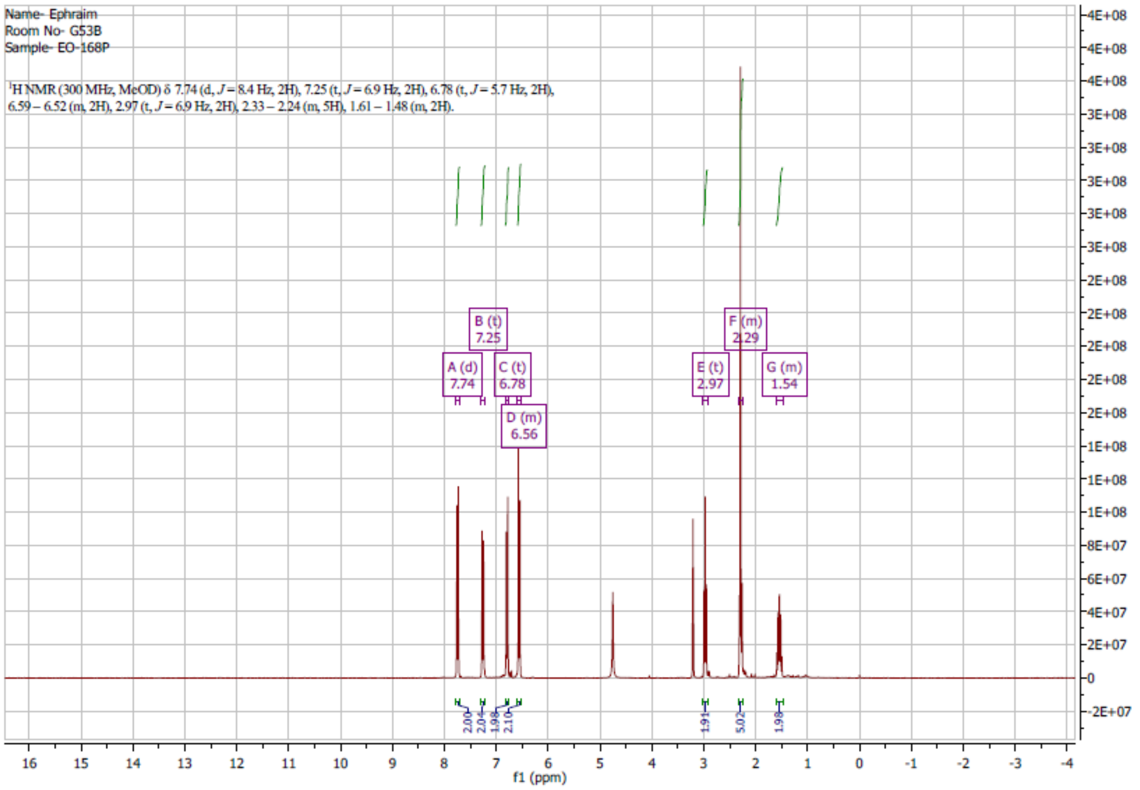

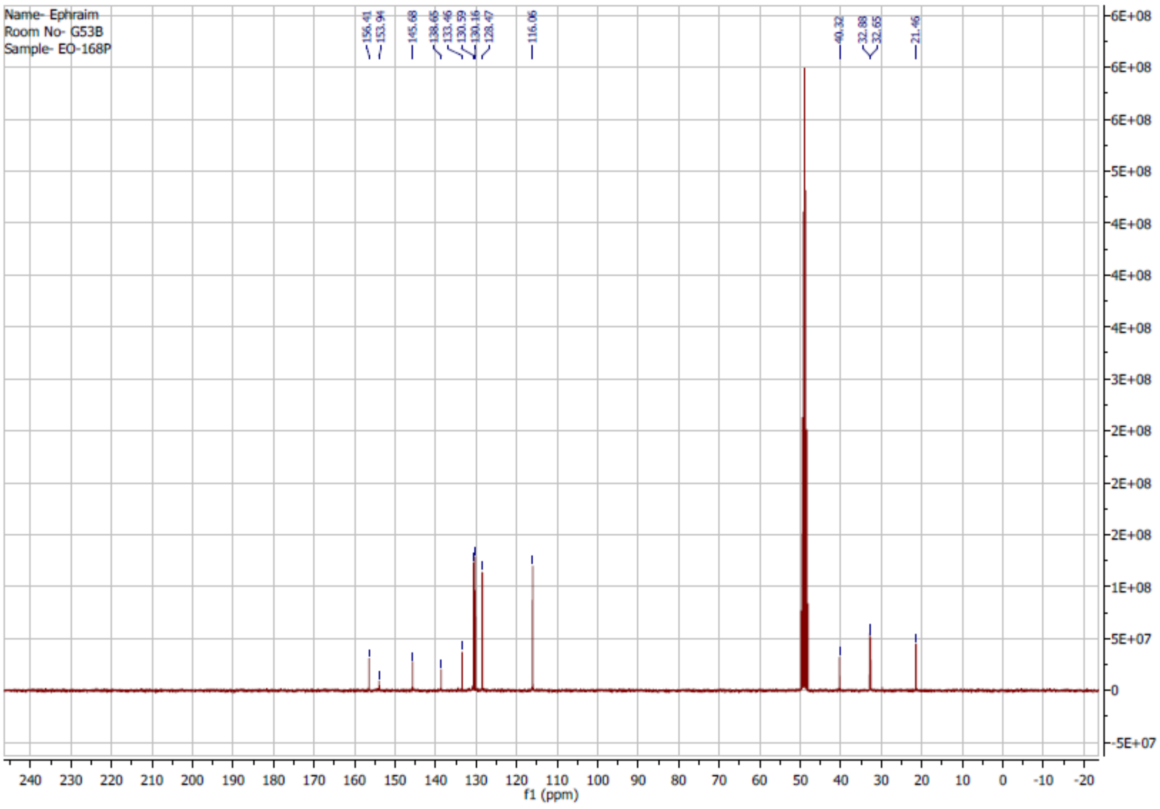

(6a*R, 10a*S**)-6-Isopropyl-2,3,6a,7-tetrahydro-1*H*,5*H*-benzo[*d*]pyrrolo[1,2-*c*]imidazole-5,8(6*H*)-dione 2a**

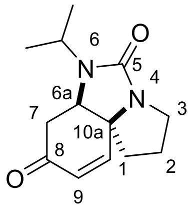

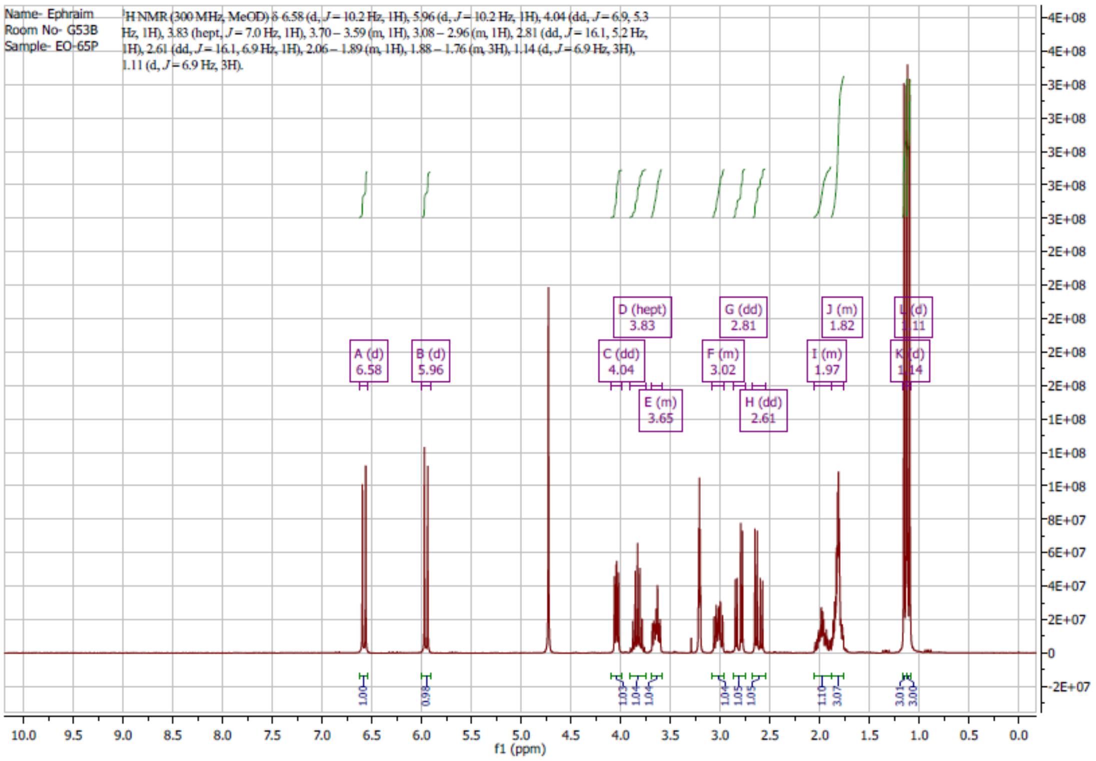

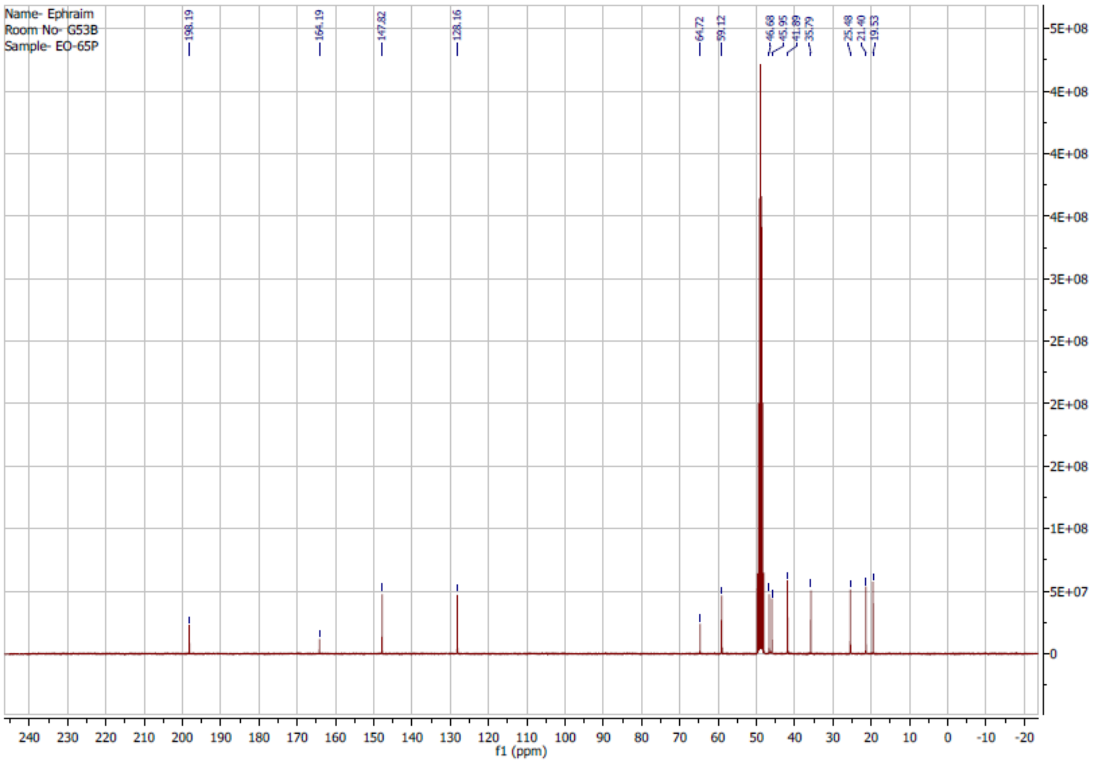

(6a*R,10a*S**)-6-(4-Methoxyphenyl)-2,3,6a,7-tetrahydro-1*H*,5*H*-benzo[*d*]pyrrolo[1,2-*c*]imidazole-5,8(6*H*)-dione 2b**

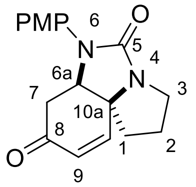

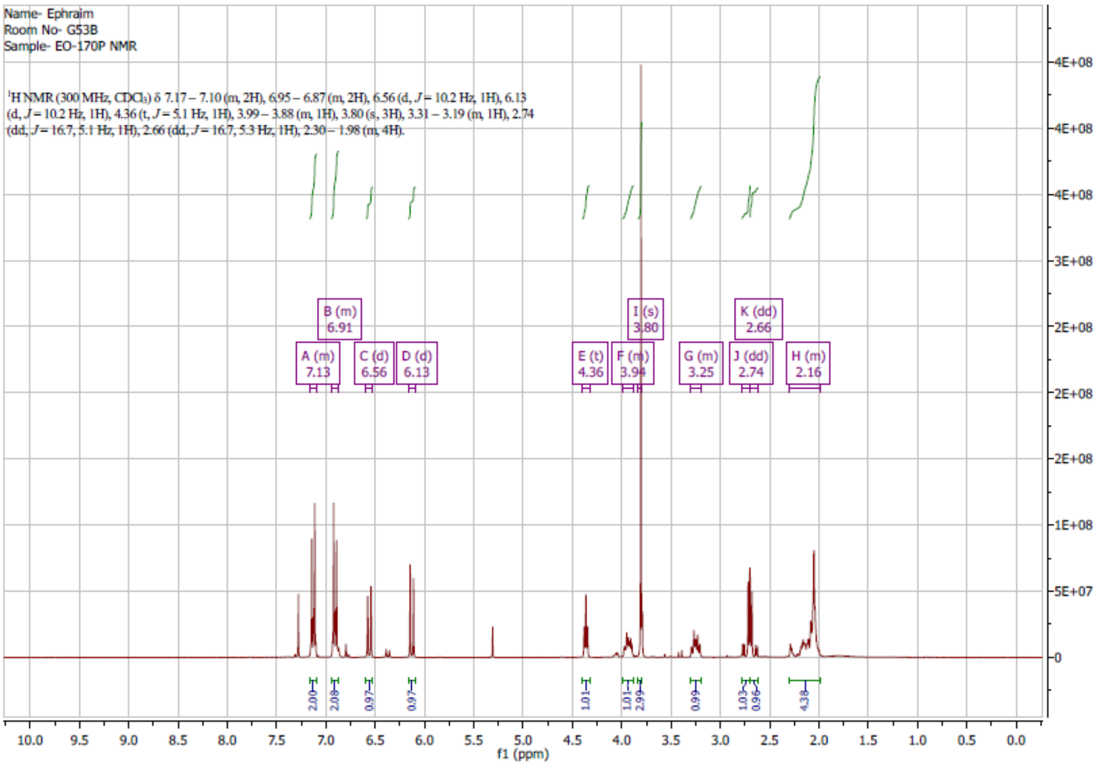

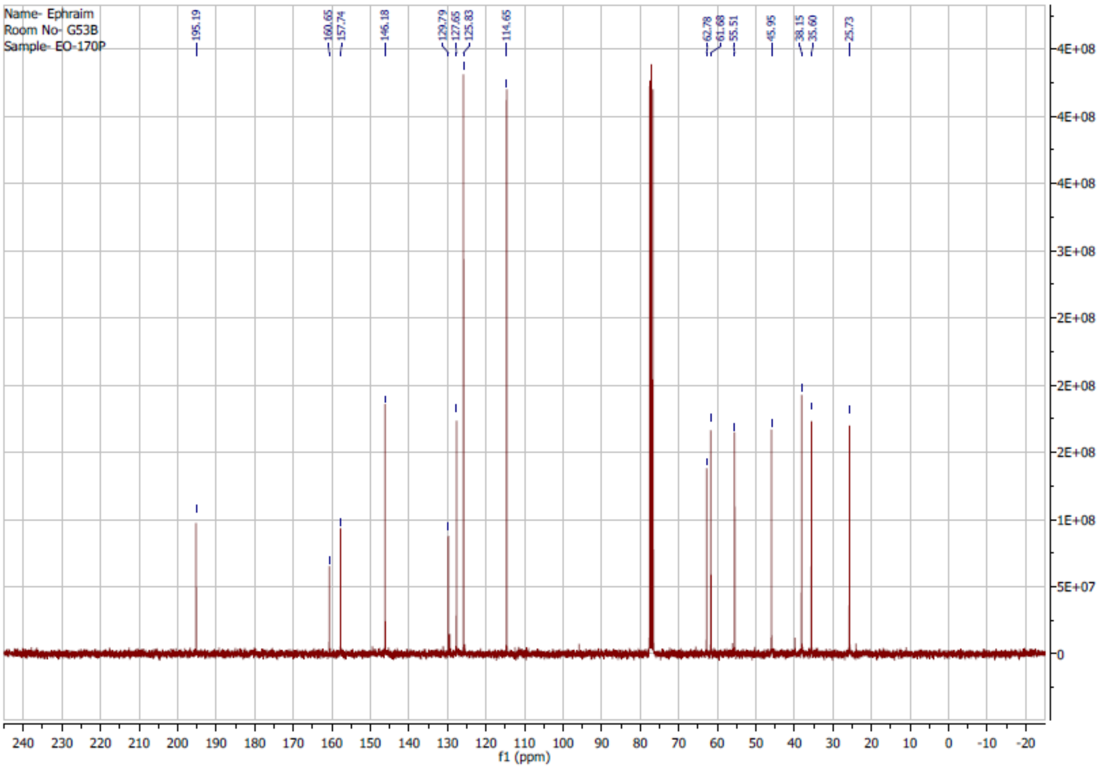

(6a*R,10a*S**)-6-(4-Toluenesulfonyl)-2,3,6a,7-tetrahydro-1*H*,5*H*-benzo[*d*]pyrrolo[1,2-*c*]imidazole-5,8(6*H*)-dione 2c**

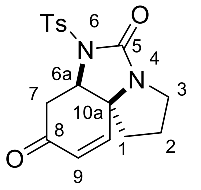

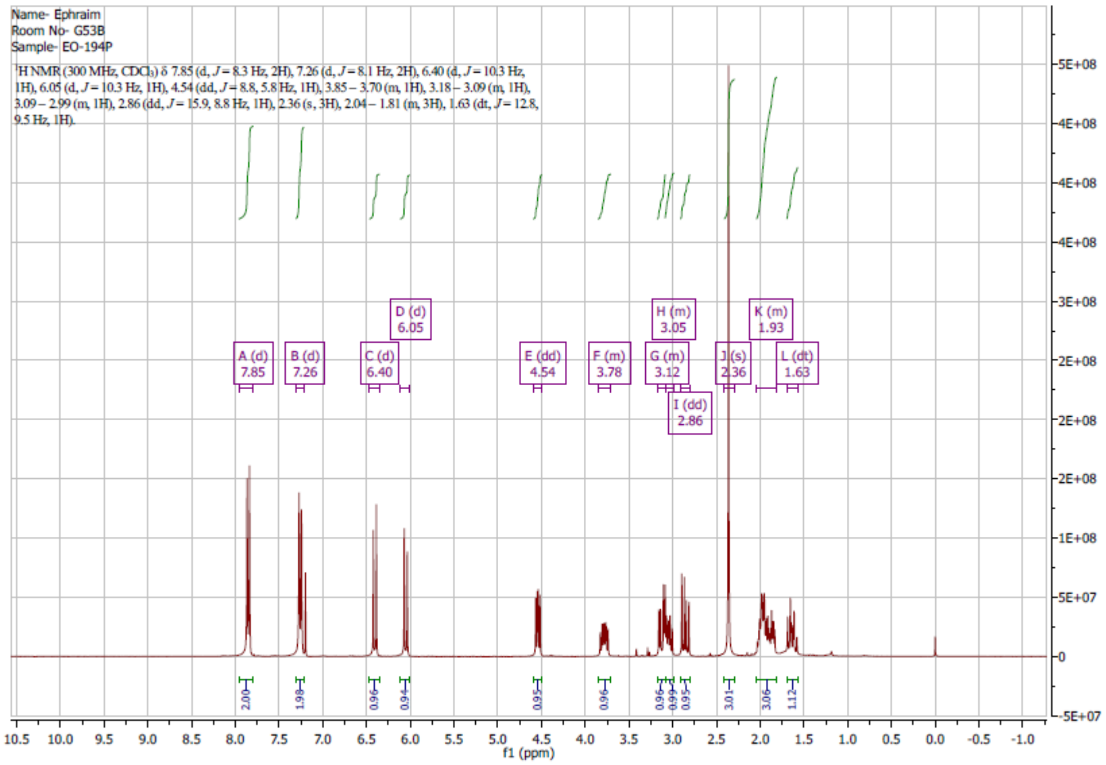

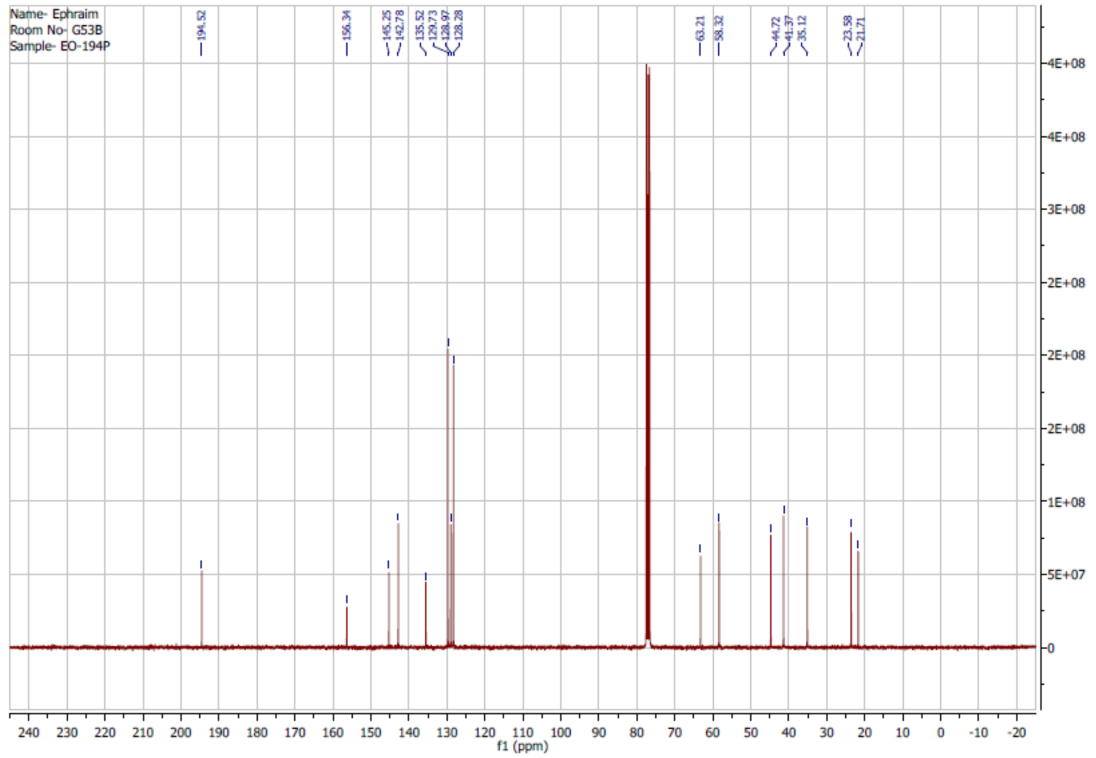

(6a*R,8*S**,10*S**)-8-Hydroxy-6-isopropyl-2,3,6,6a,7,8-hexahydro-1*H*,5*H*-benzo[*d*]pyrrolo[1,2-*c*]imidazol-5-one 3a**

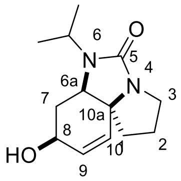

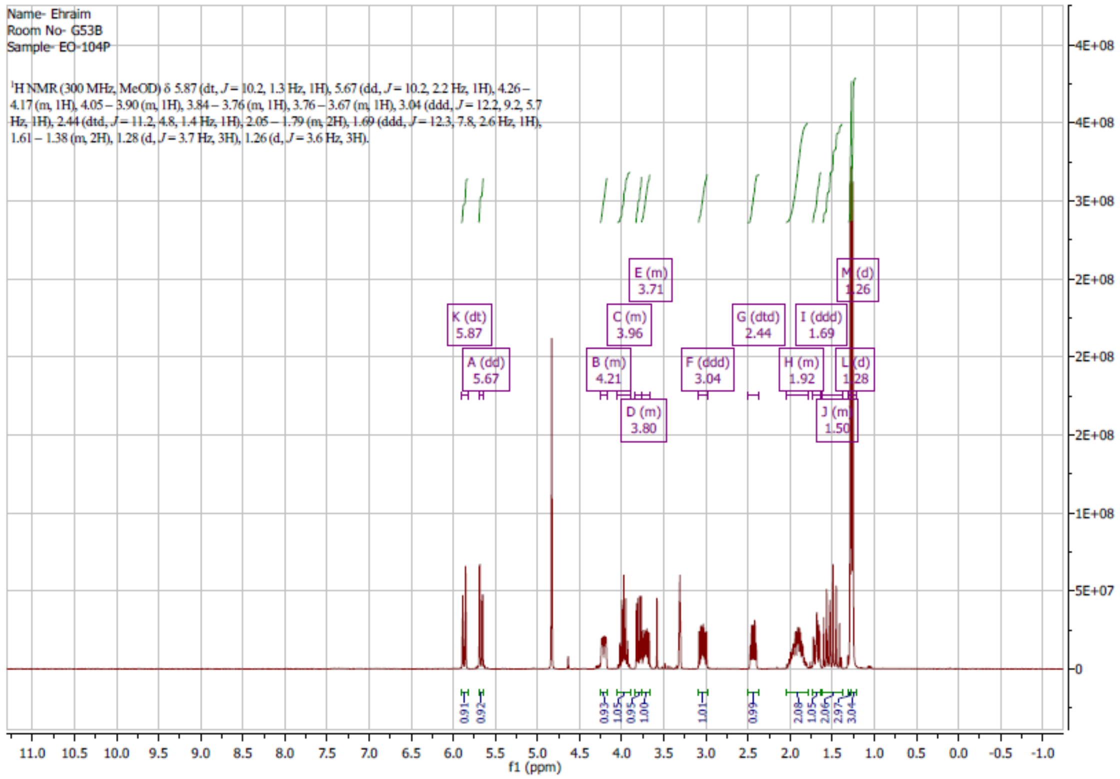

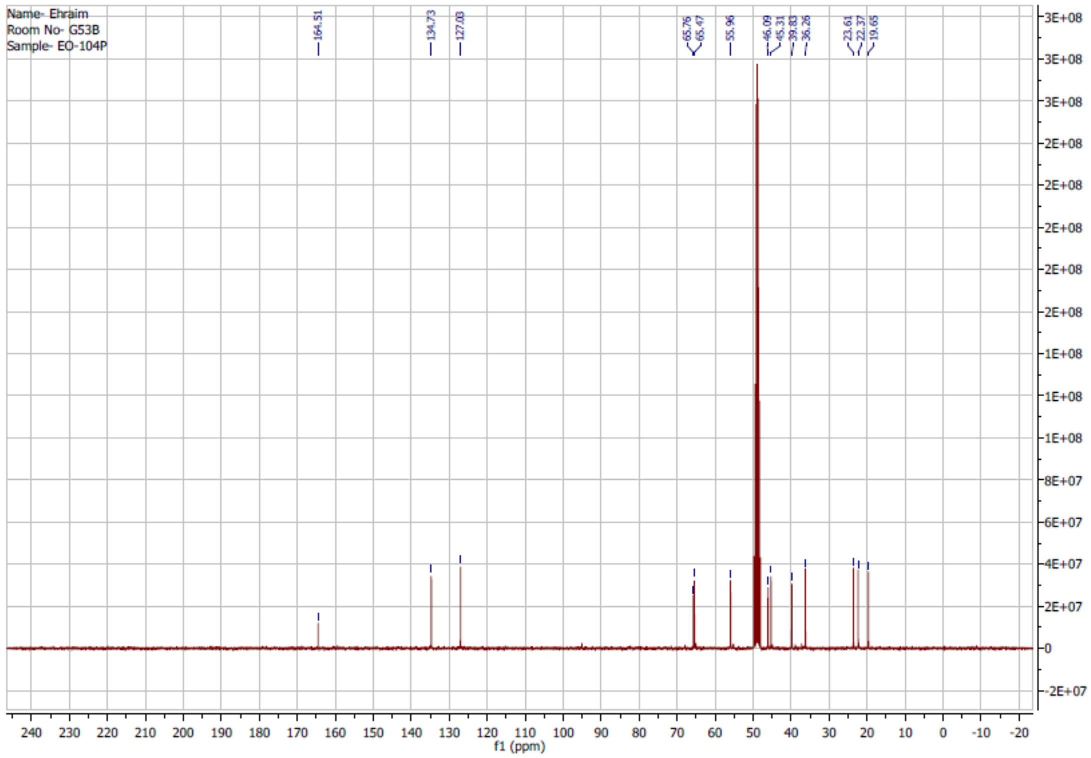

(6a*R,10a*S**)-6-Isopropylhexahydro-1*H*,5*H*-benzo[*d*]pyrrolo[1,2-*c*]imidazole-5,8(6*H*)-dione 4a**

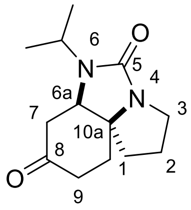

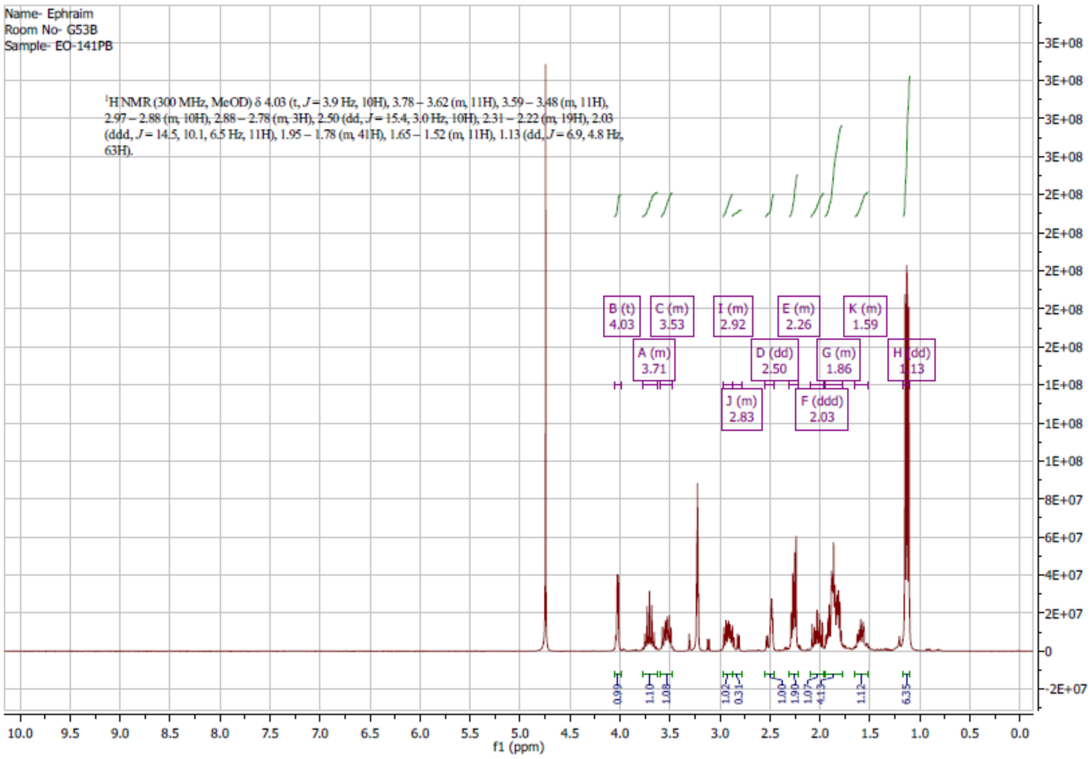

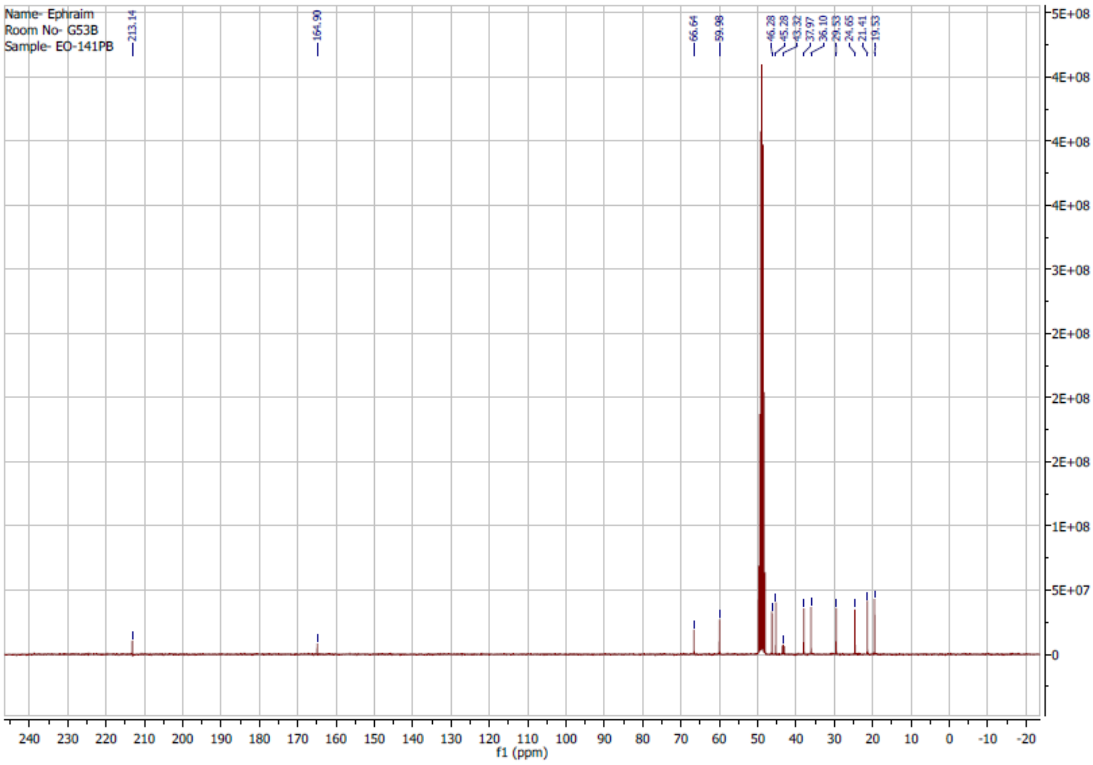

(6a*R,10a*S**)-6-(4-Methoxyphenyl)hexahydro-1*H*,5*H*-benzo[*d*]pyrrolo[1,2-*c*]imidazole-5,8(6*H*)-dione 4b**

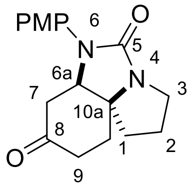

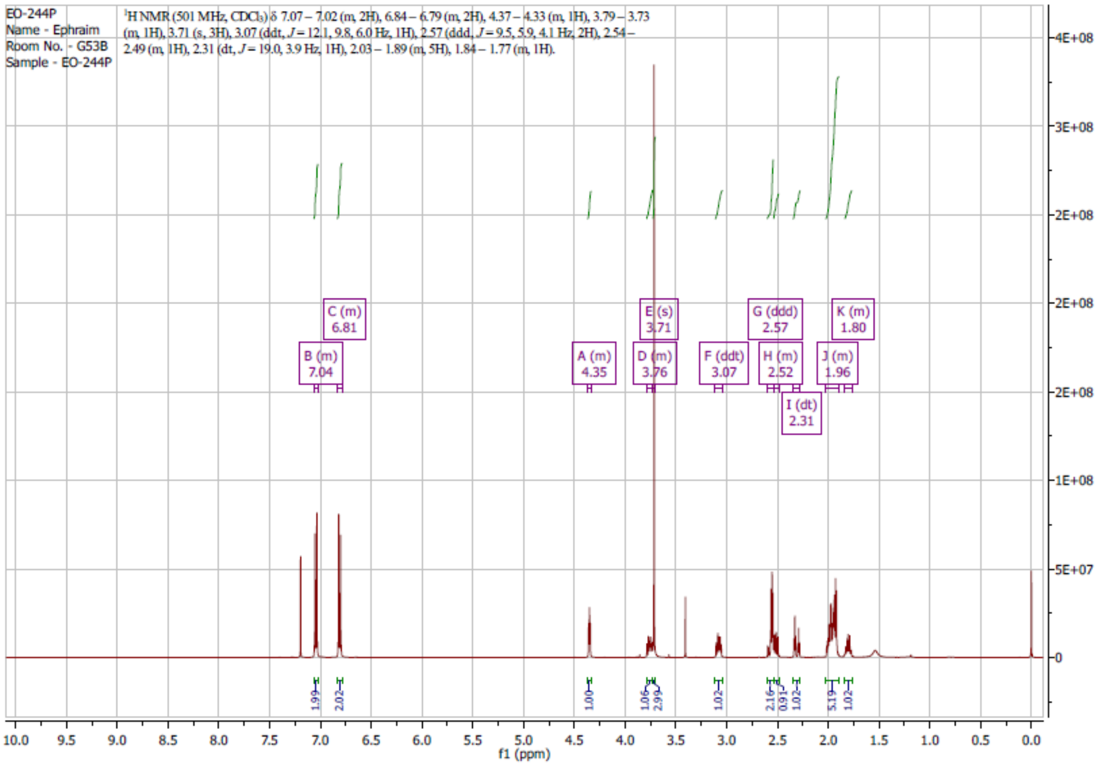

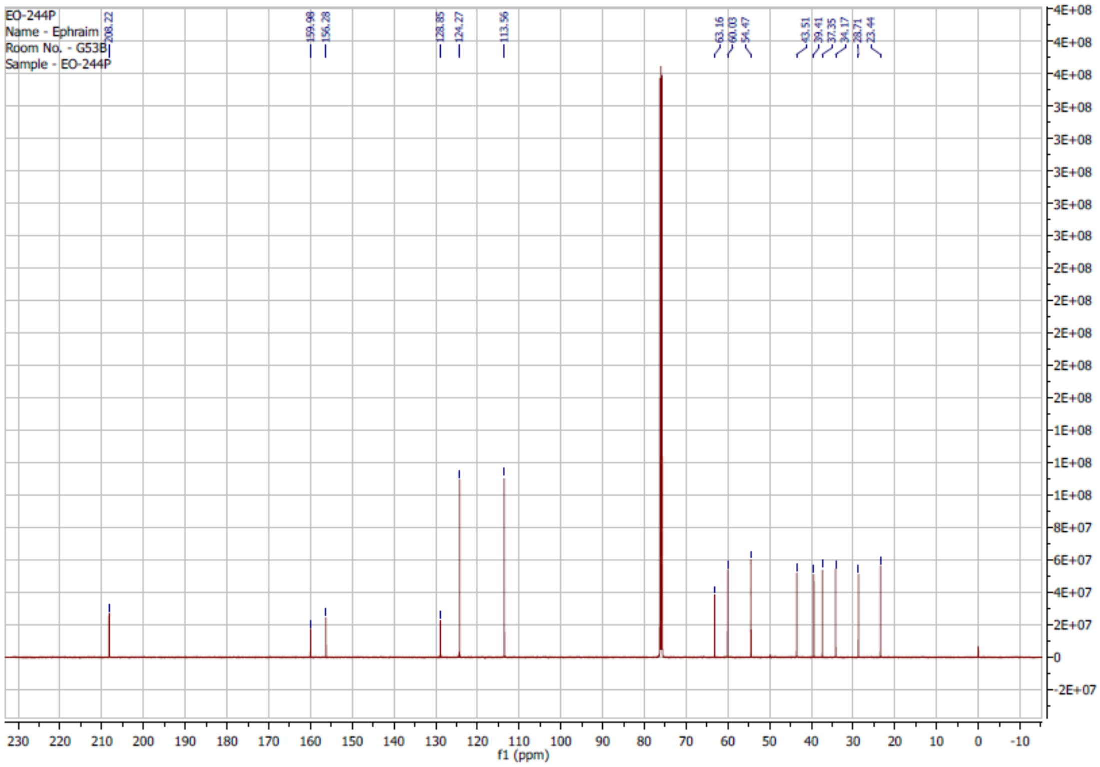

(6a*R,10a*S**)-6-(4-Toluenesulfonyl)hexahydro-1*H*,5*H*-benzo[*d*]pyrrolo[1,2-*c*]imidazole-5,8(6*H*)-dione 4c**

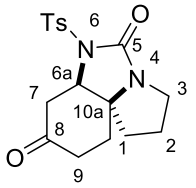

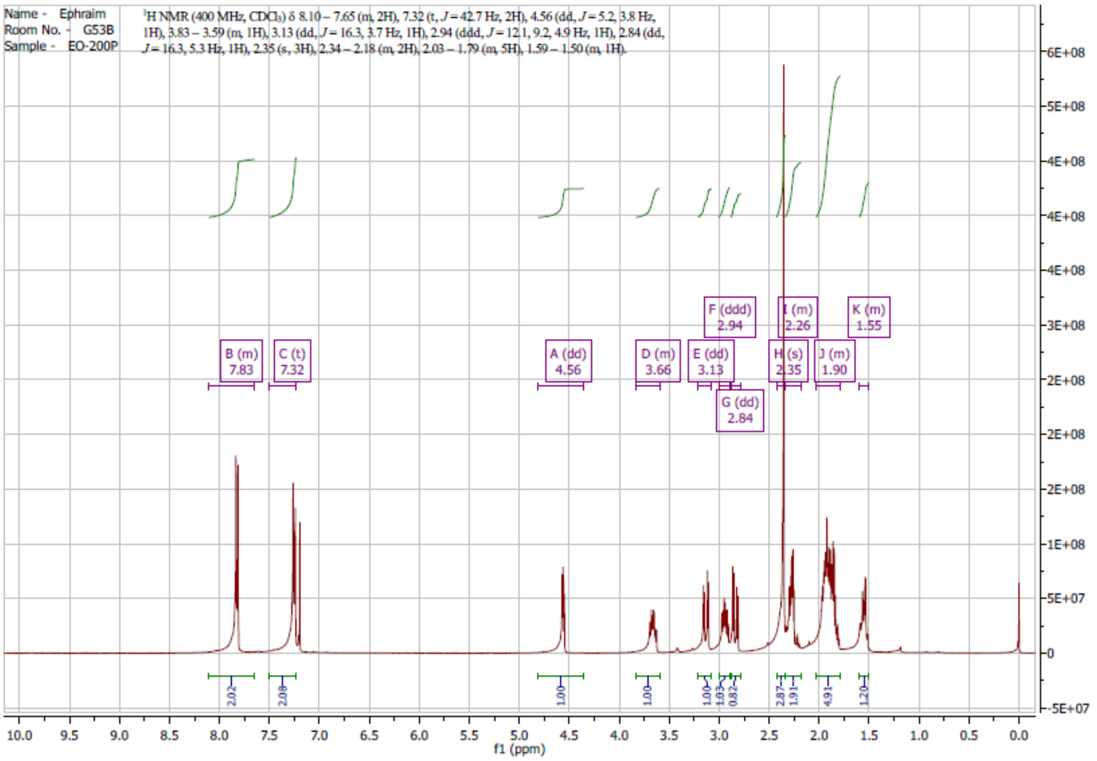

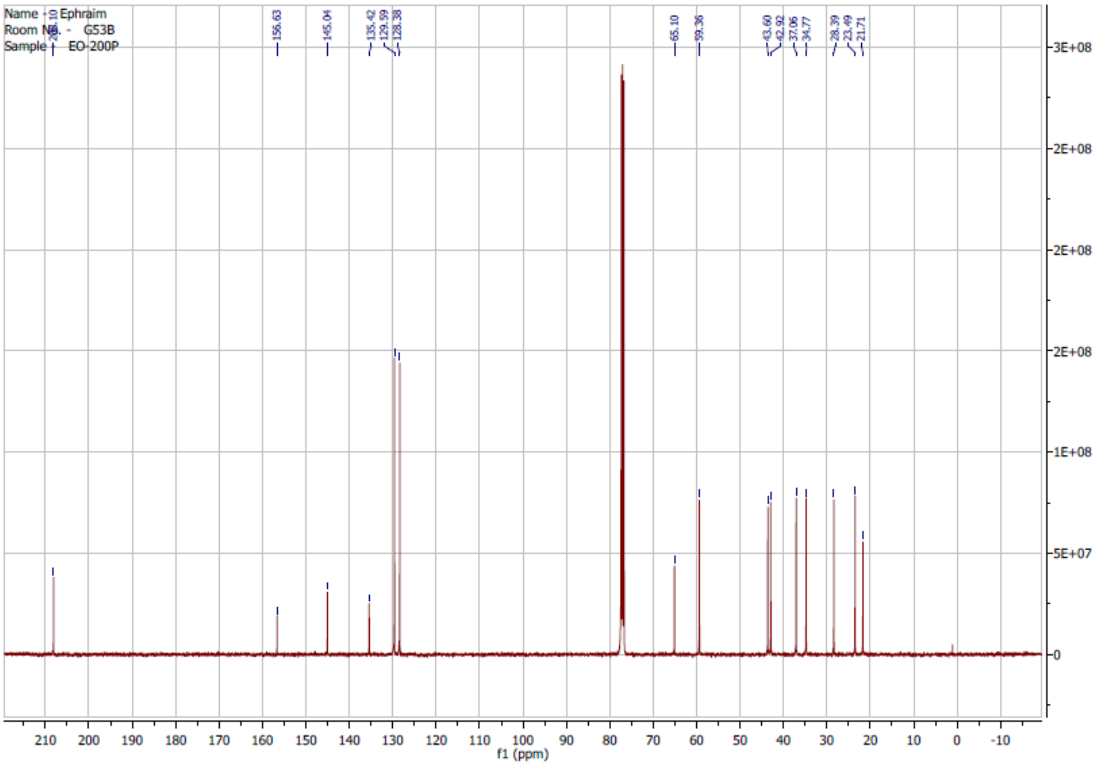

(6a*R,8*R**,10a*S**)-8-Hydoxy-6-isopropyl-8-phenyloctahydro-1*H*,5*H*-benzo[*d*]pyrrolo[1,2-*c*]imidazol-5-one 5a**

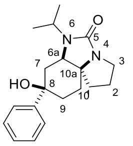

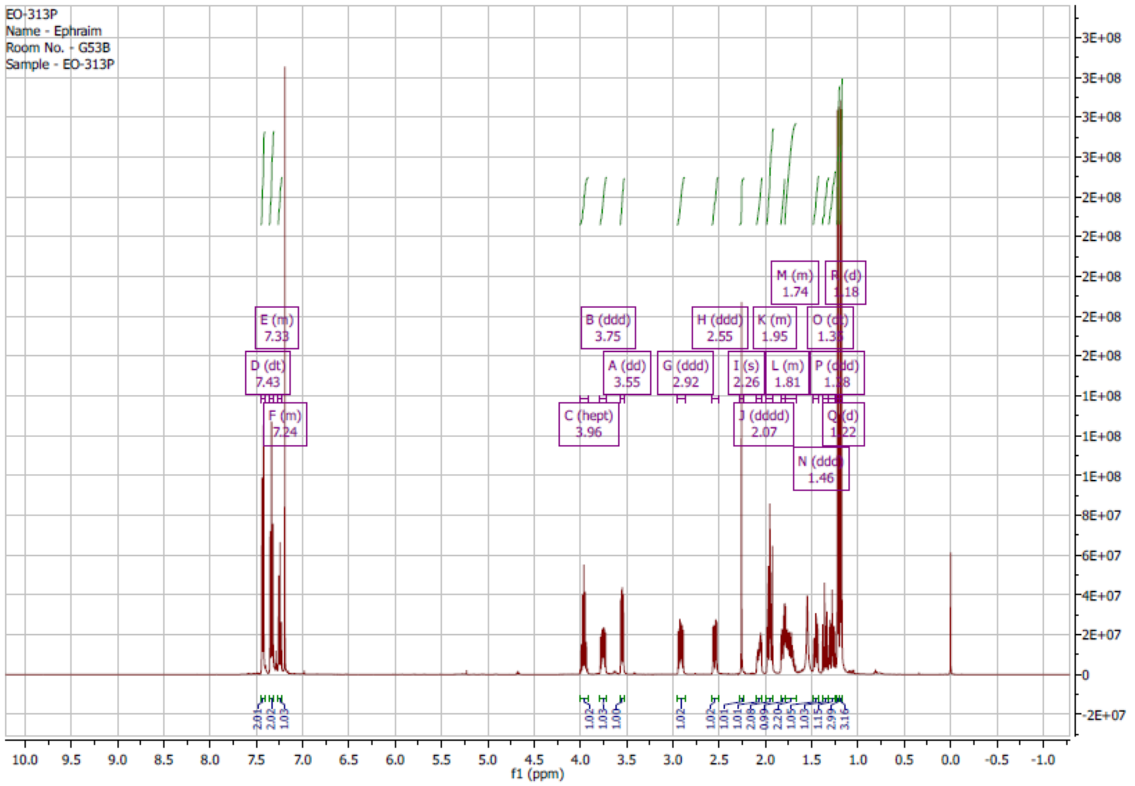

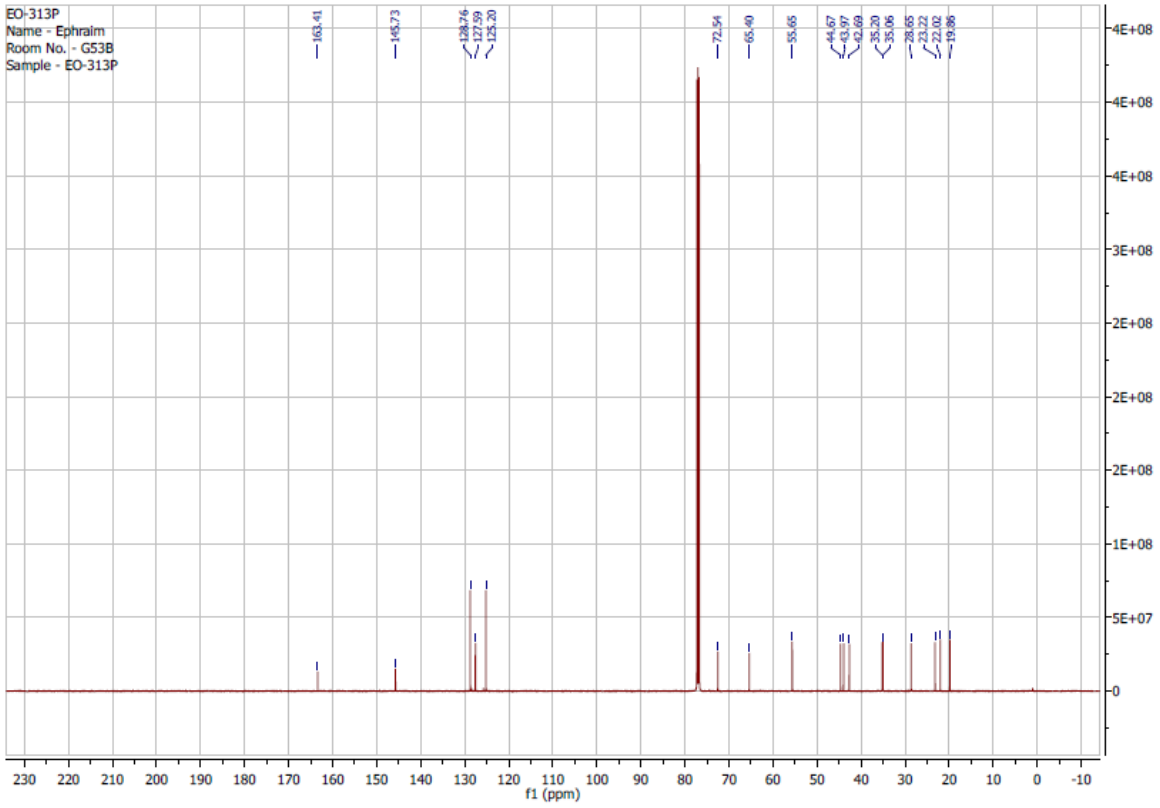

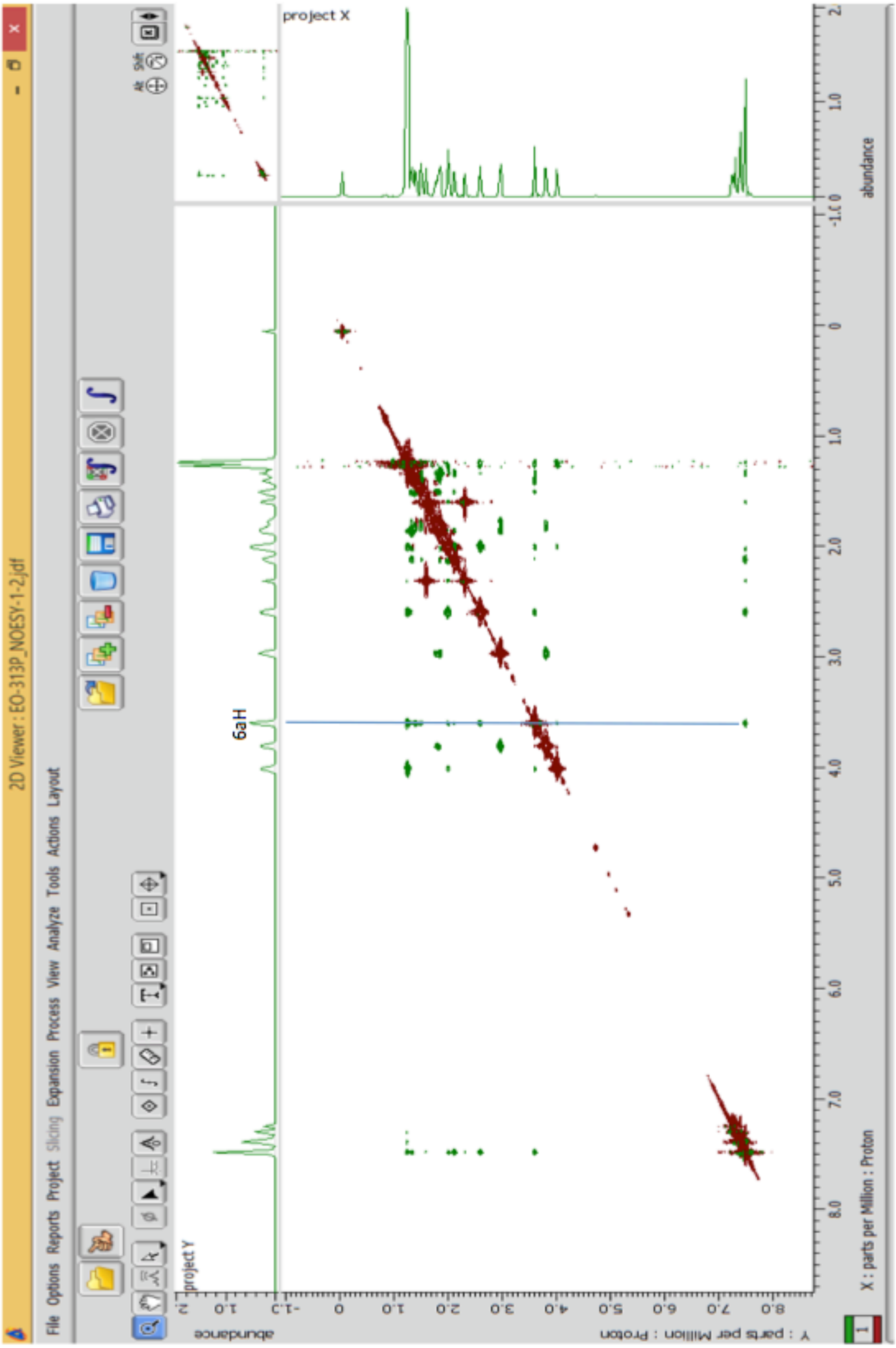
2D Viewer : EO-313P_NOESY-1-2.jdf
project X
project Y
H6a
abundance
X : parts per Million : Proton
Y : parts per Million : Proton

(6a*R,8*R**,10a*S**)-8-Hydroxy-6-isopropyloctahydro-1*H*,5*H*-benzo[*d*]pyrrolo[1,2-*c*]imidazol-5-one 6a**

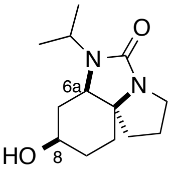

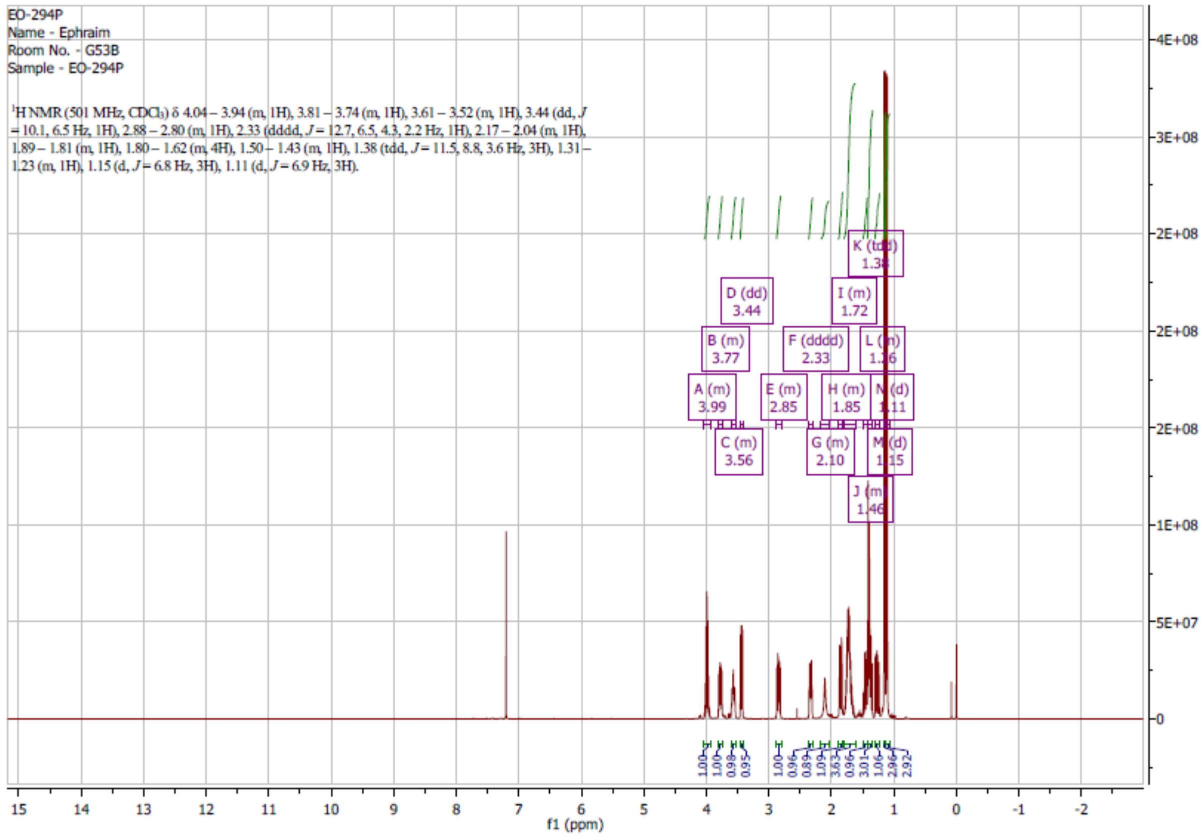

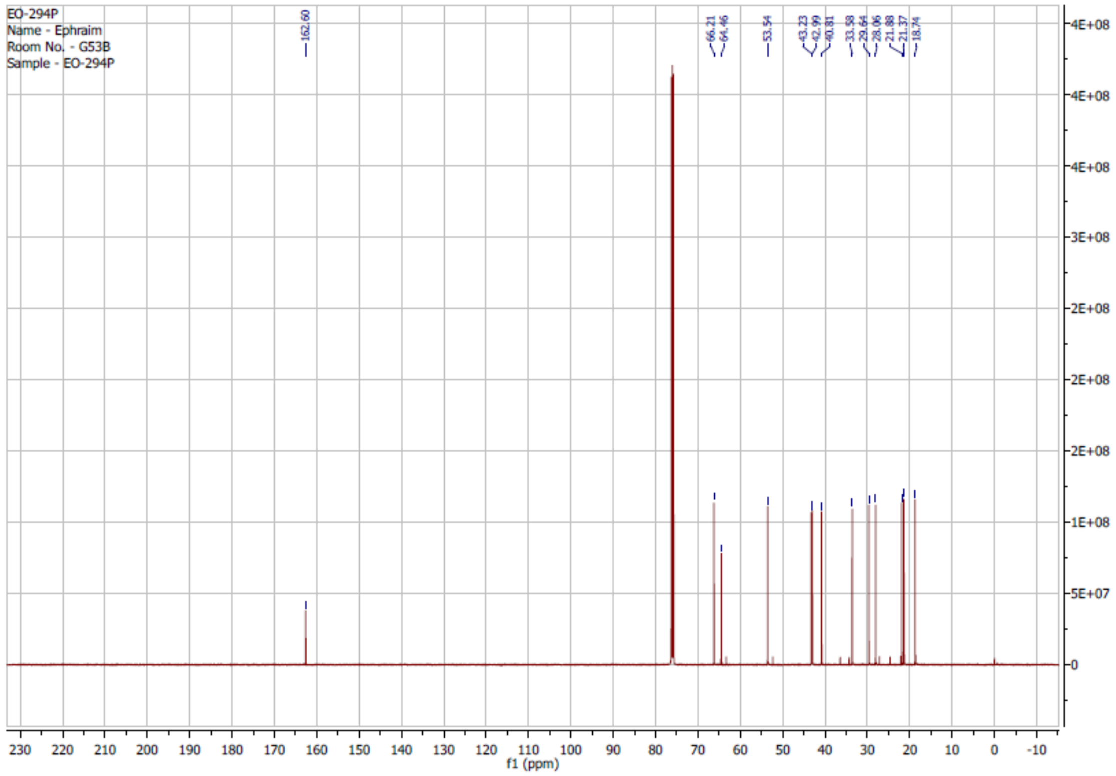

(6a*R**,8*R**,10a*S**)-8-Hydroxy-6-(4-methoxyphenyl)octahydro-1*H*,5*H*-benzo[*d*]pyrrolo[1,2-*c*]imidazol-5-one 6b

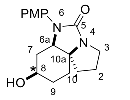

Major diastereomer, *dr* 85:15

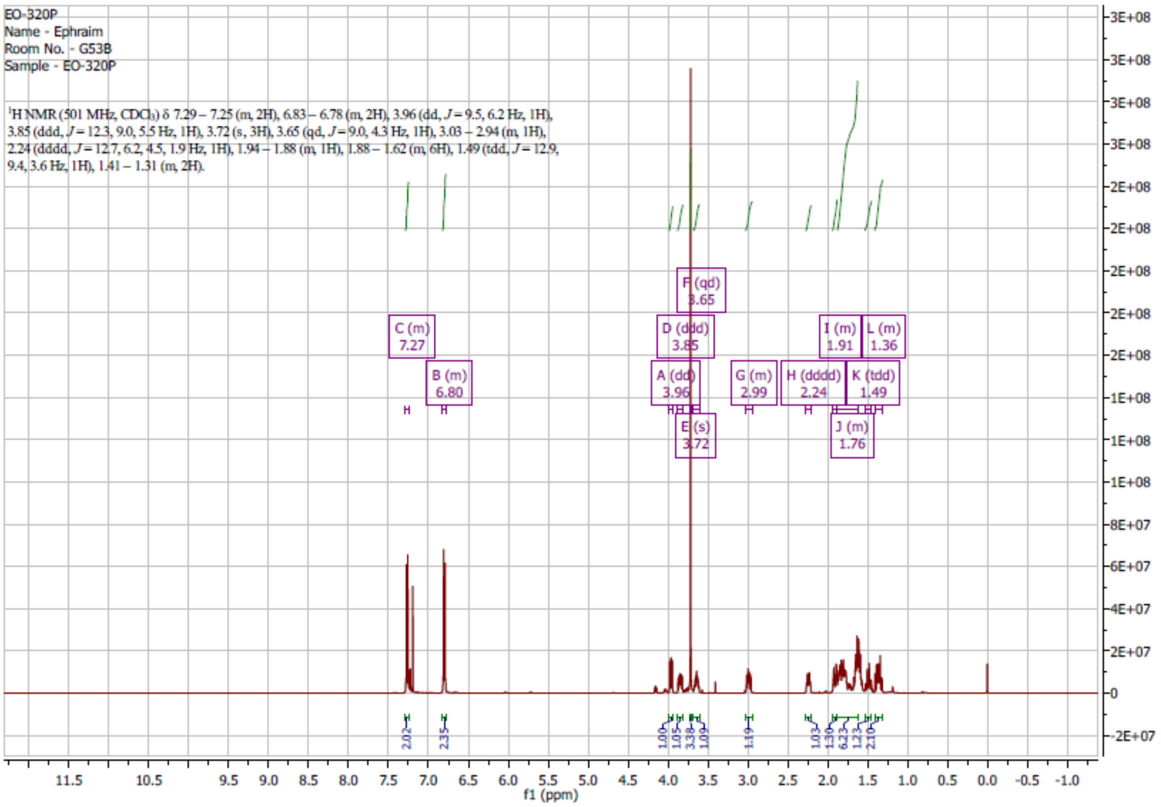

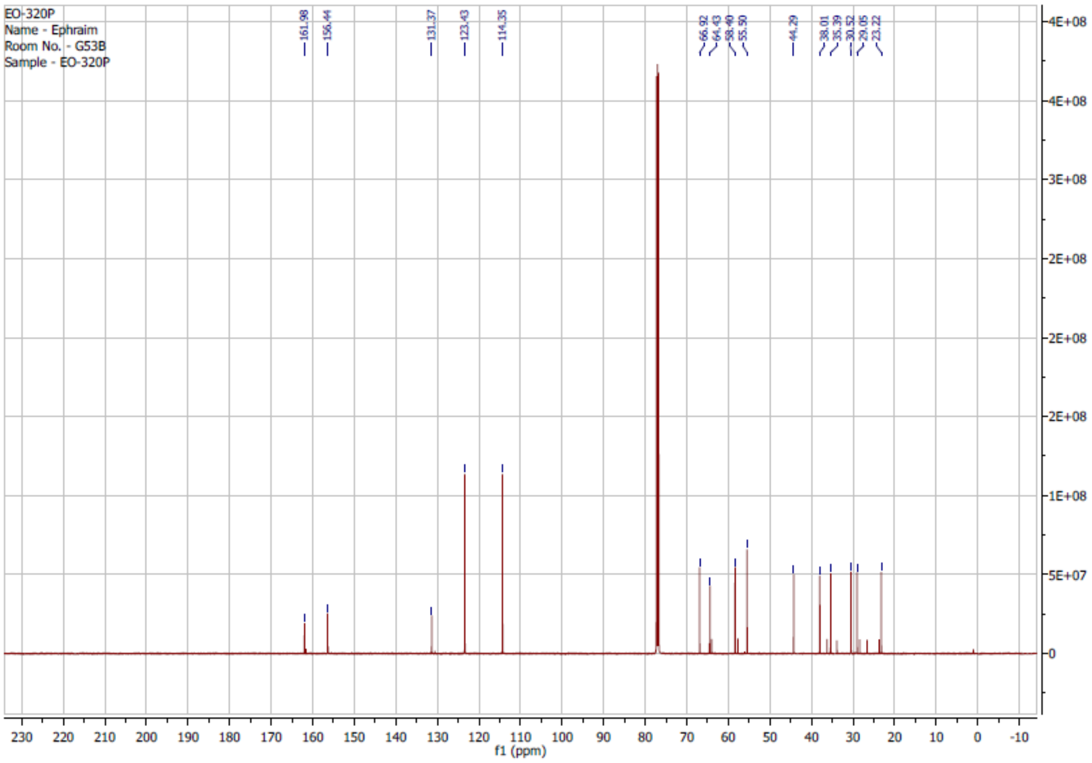

(6a*R,8*R**,10a*S**)-8-Hydroxy-6-(4-toluenesulfonyl)octahydro-1*H*,5*H*-benzo[*d*]pyrrolo[1,2-*c*]imidazol-5-one 6c**

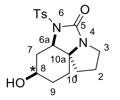

Major diastereomer, *dr* 78:22

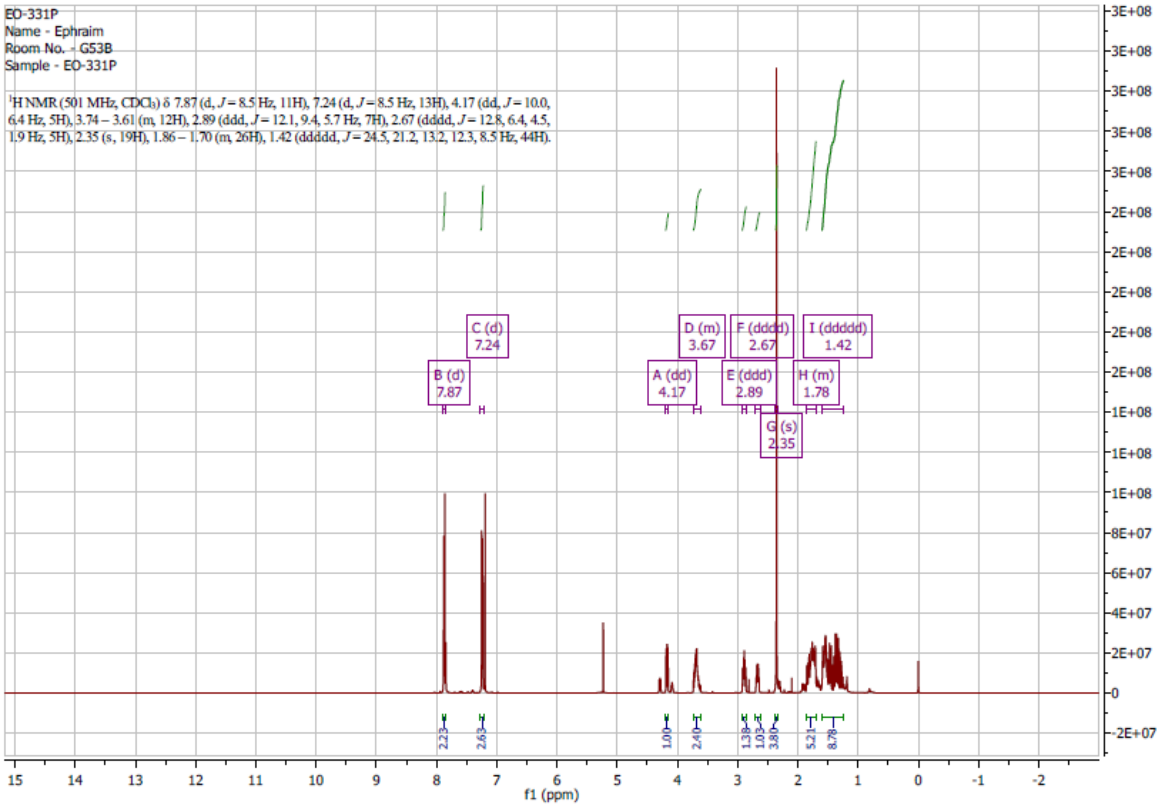

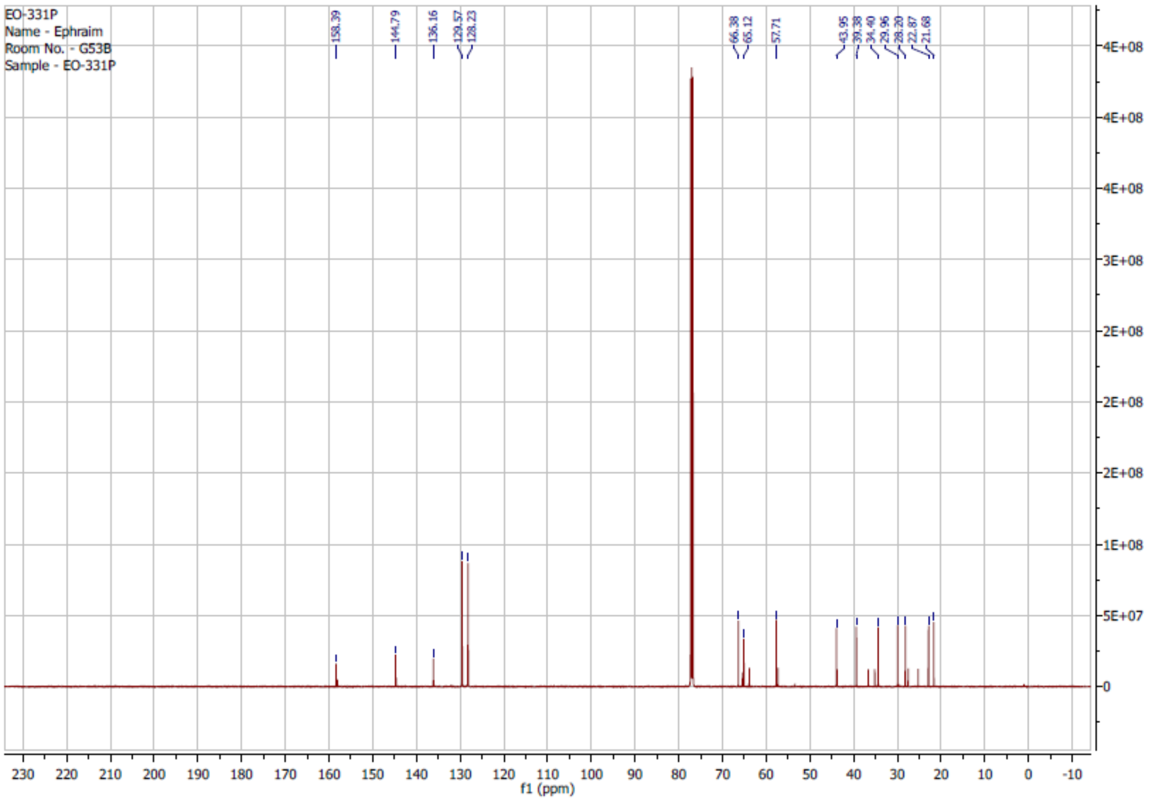

(6a*R,8*R**,10a*S**)-6-Isopropyl-8-(methylamino)octahydro-1*H*,5*H*-benzo[*d*]pyrrolo[1,2-*c*]imidazol-5-one 7a**

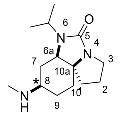

Major diastereomer, *dr* 88:12

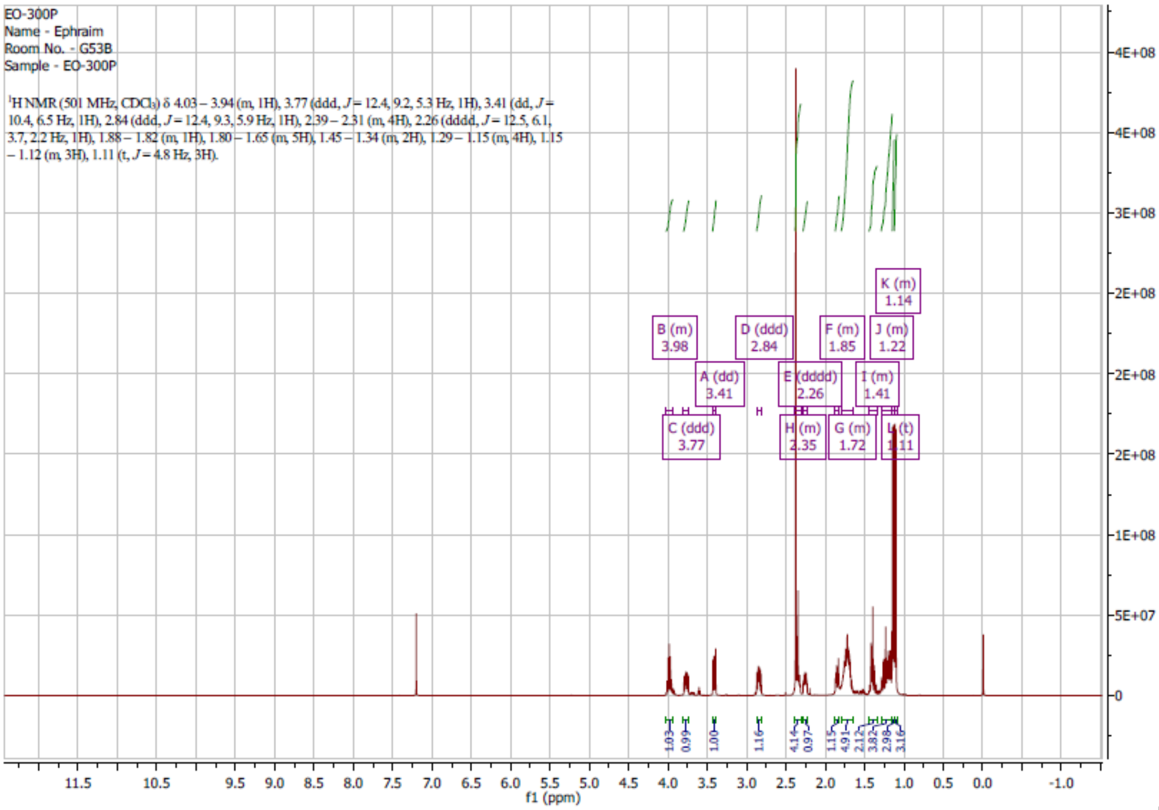

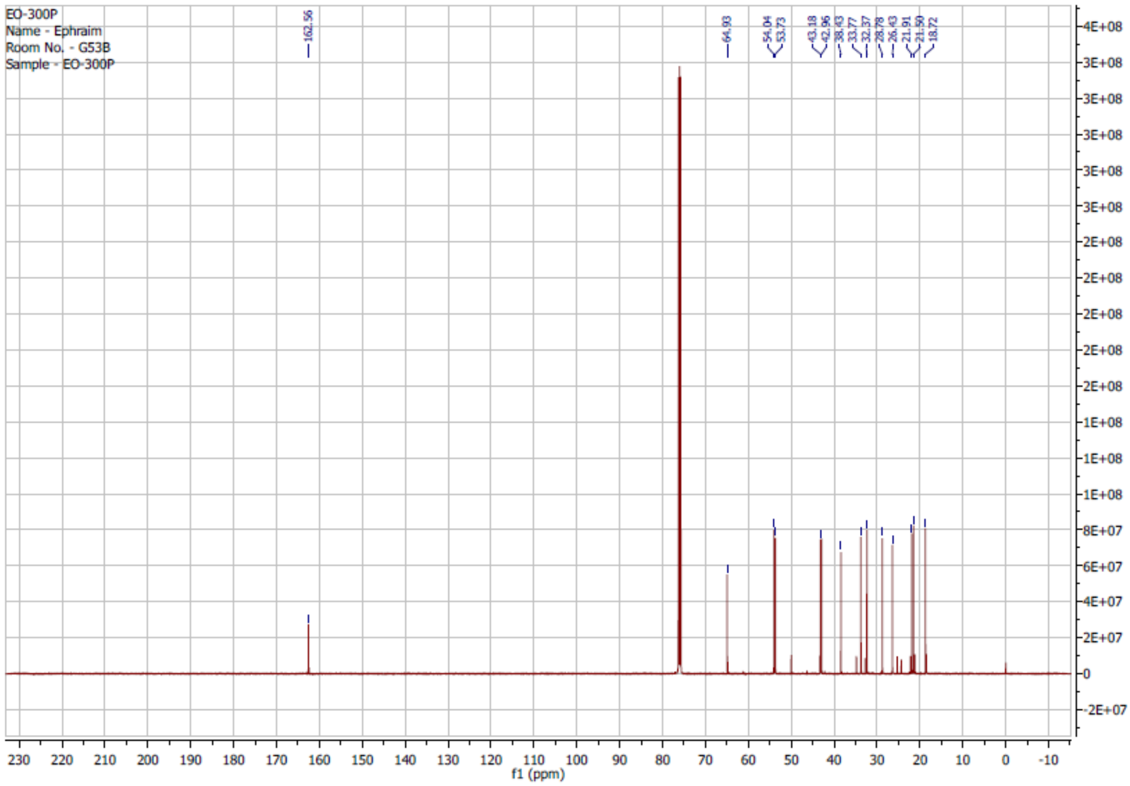

(6a*R,11b*S**)-6-Isopropyl-2,3,6a,7-tetrahydro-1*H*-pyrrolo[1',2':3,4]imidazole[4,5-*e*]isoindole-5,8(6*H*, 10*H*)-dione 8a**

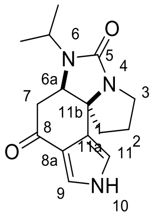

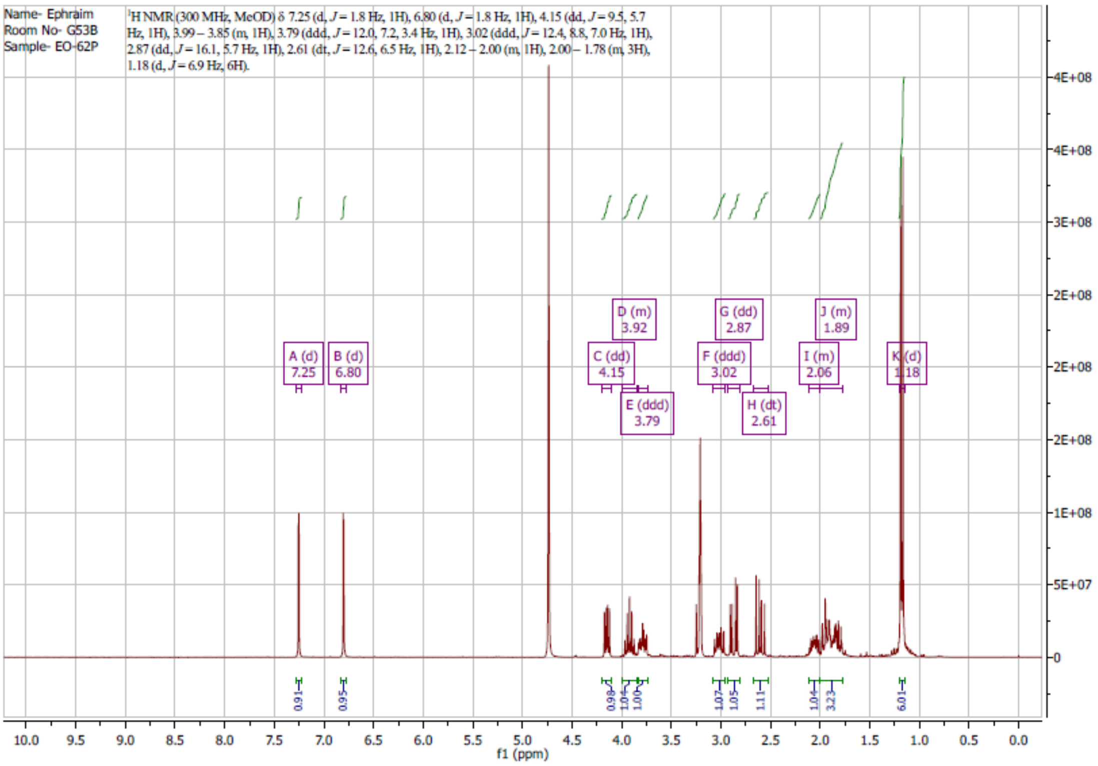

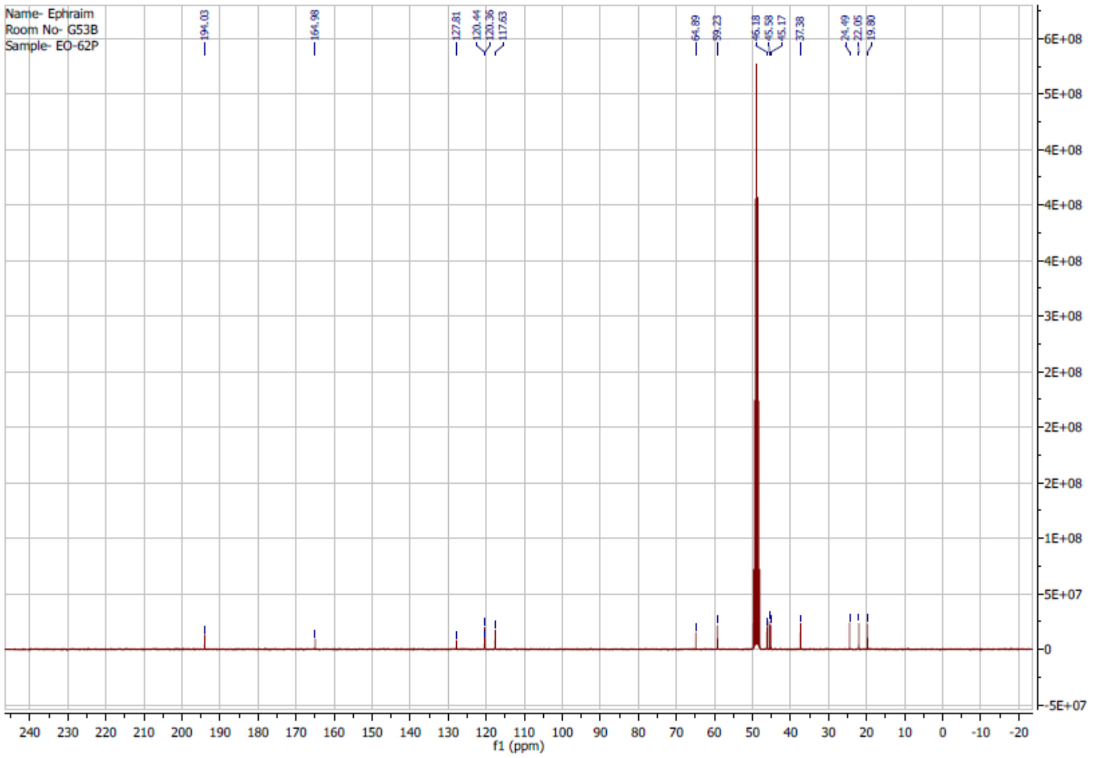

(6a*R,8a*S**,11a*R**,11b*S**)-10-Benzyl-6-isopropyloctahydro-1*H*-pyrrolo[1',2':3,4]imidazo[4,5-*e*]isoindole-5,8(6*H*,8a*H*)-dione 9a**

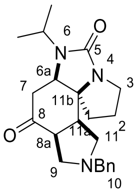

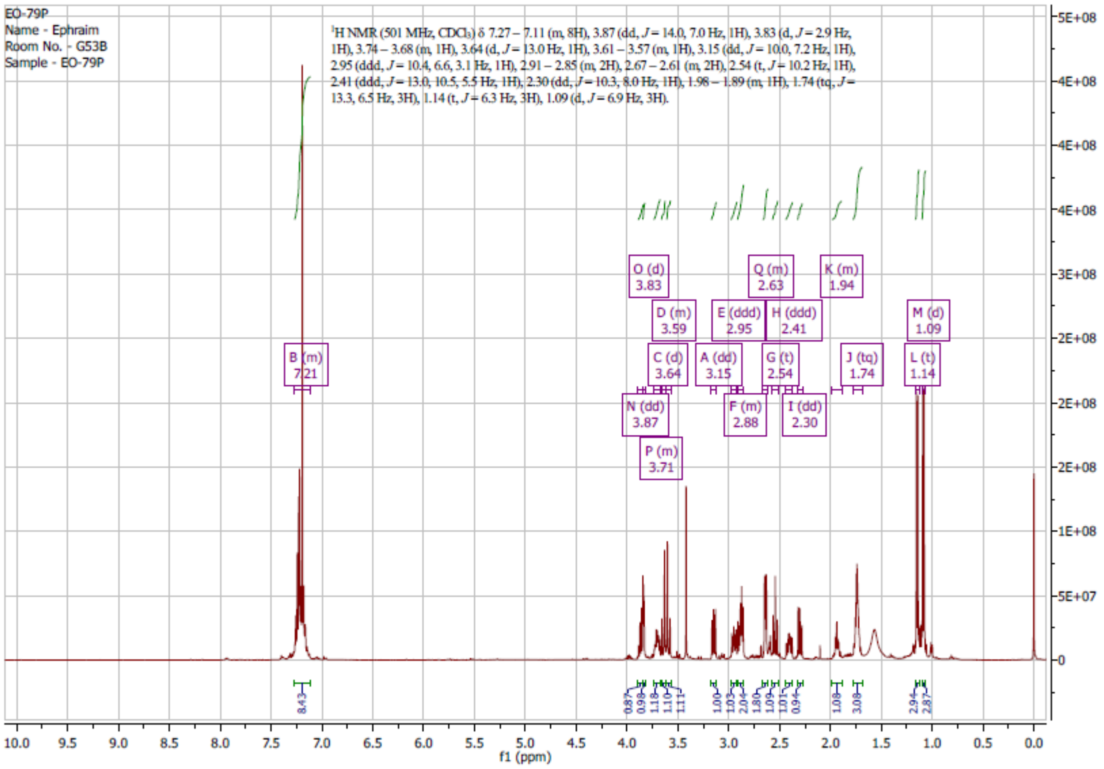

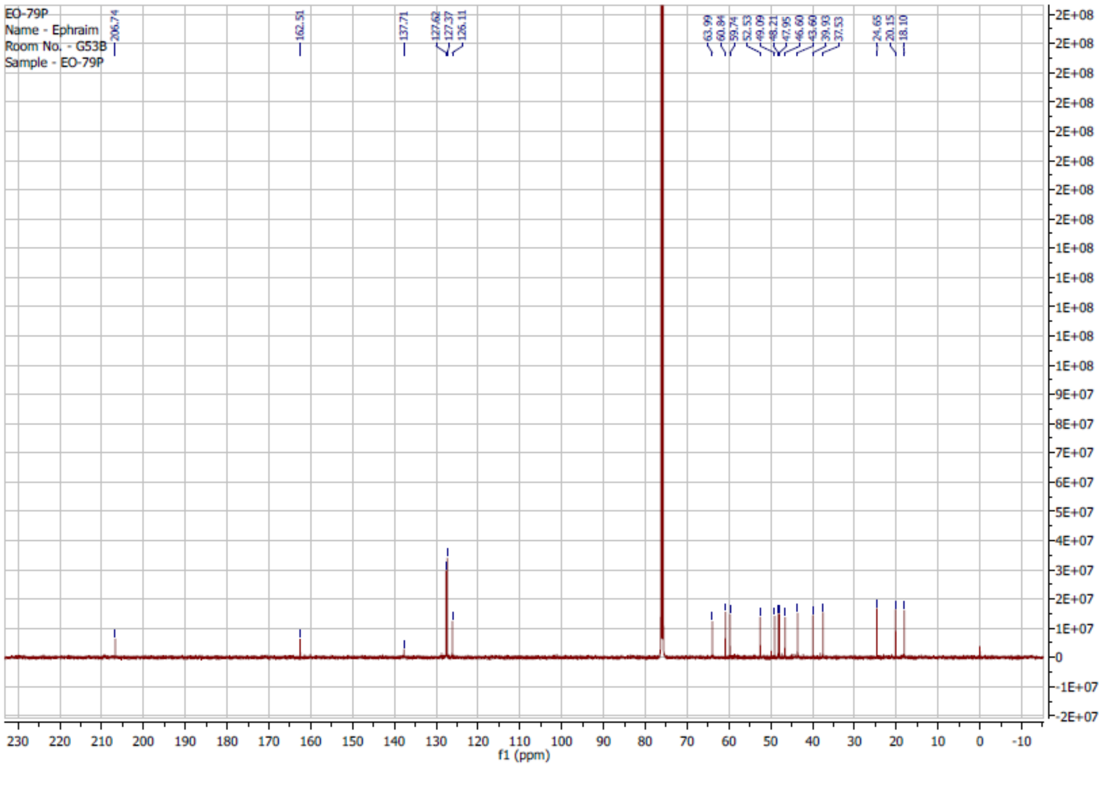

(6a*R,8a*S**,11a*R**,11b*S**)-10-Benzyl-6-(4-methoxyphenyl)octahydro-1*H*-pyrrolo[1',2':3,4]imidazo[4,5-*e*]isoindole-5,8(6*H*,8a*H*)-dione 9b**

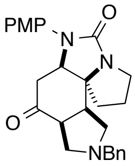

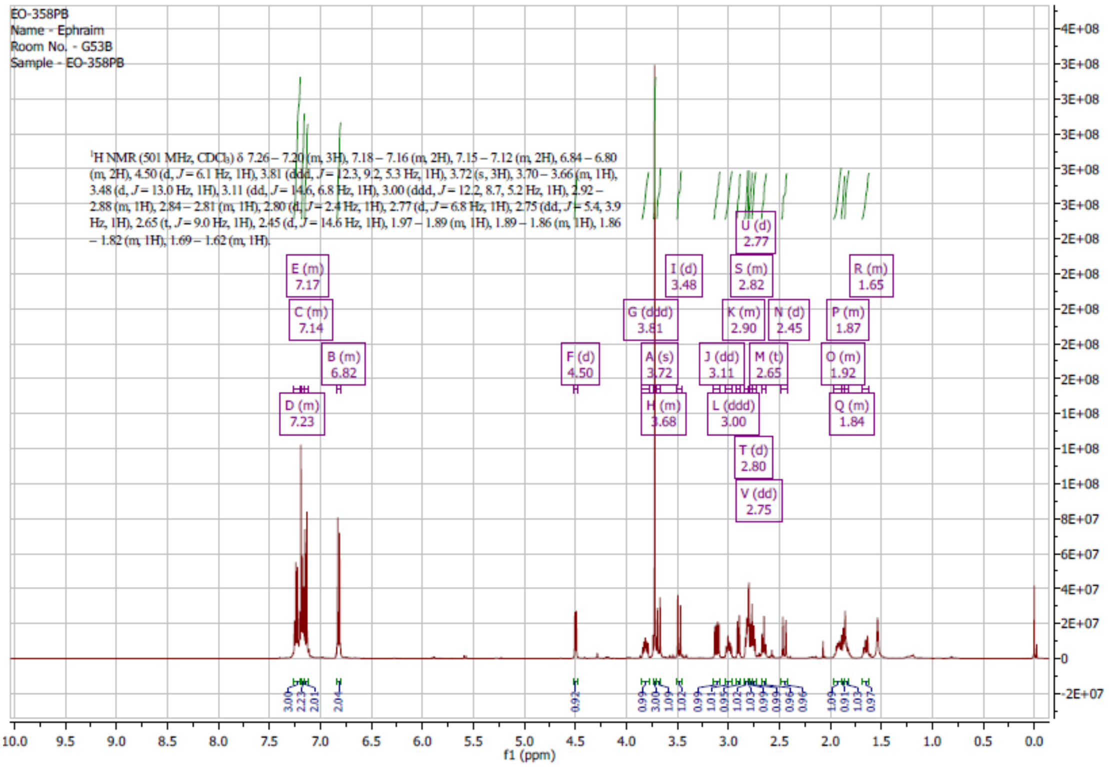

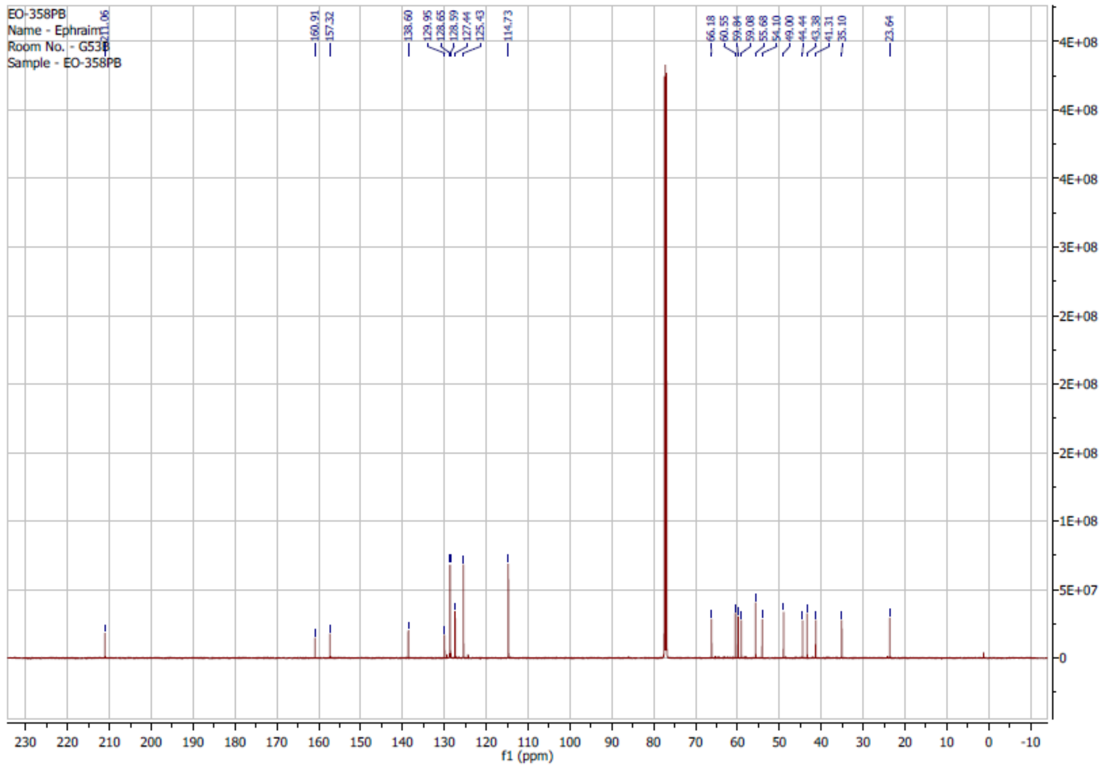

(6a*R,8a*R**,11a*S**,11b*S**)-10-Benzyl-6-(4-methoxyphenyl)octahydro-1*H*-pyrrolo[1′,2′:3,4]imidazo[4,5-*e*]isoindole-5,8(6*H*,8a*H*)-dione 9b′**

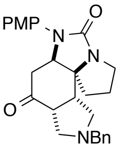

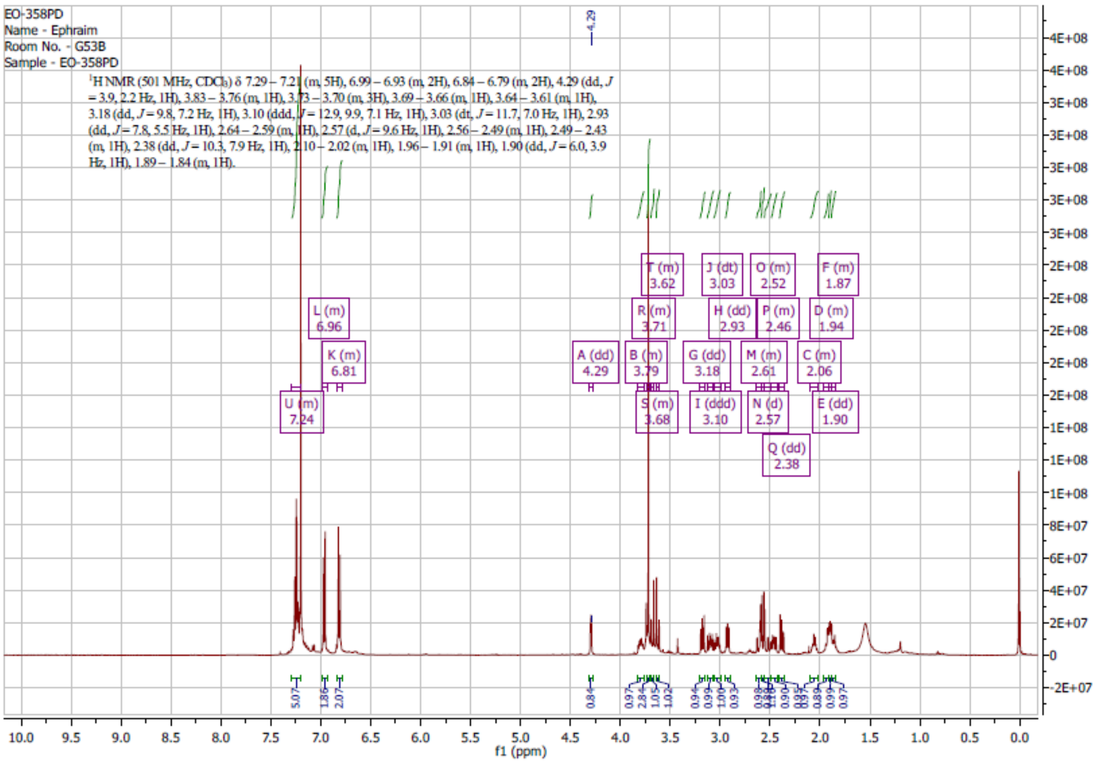

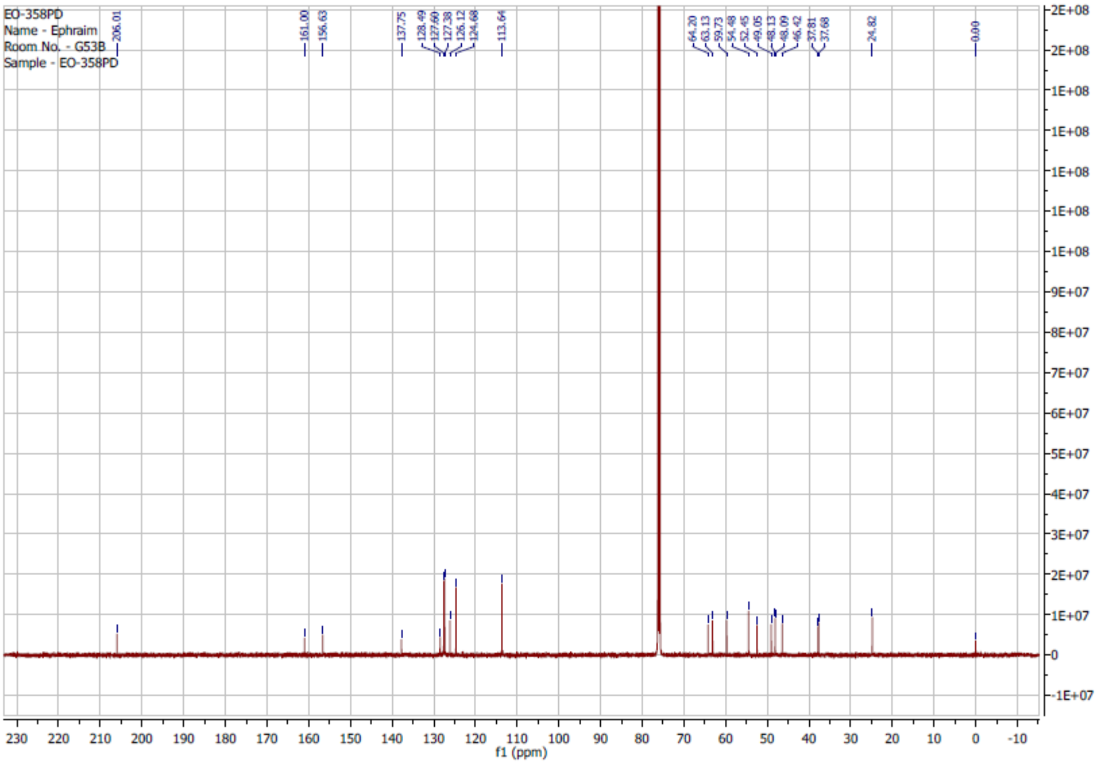

(6a*R,8a*S**,11a*R**,11b*S**)-10-Benzyl-6-tosyloctahydro-1*H*-pyrrolo[1',2':3,4]imidazo[4,5-*e*]isoindole-5,8(6*H*,8a*H*)-dione 9c**

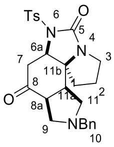

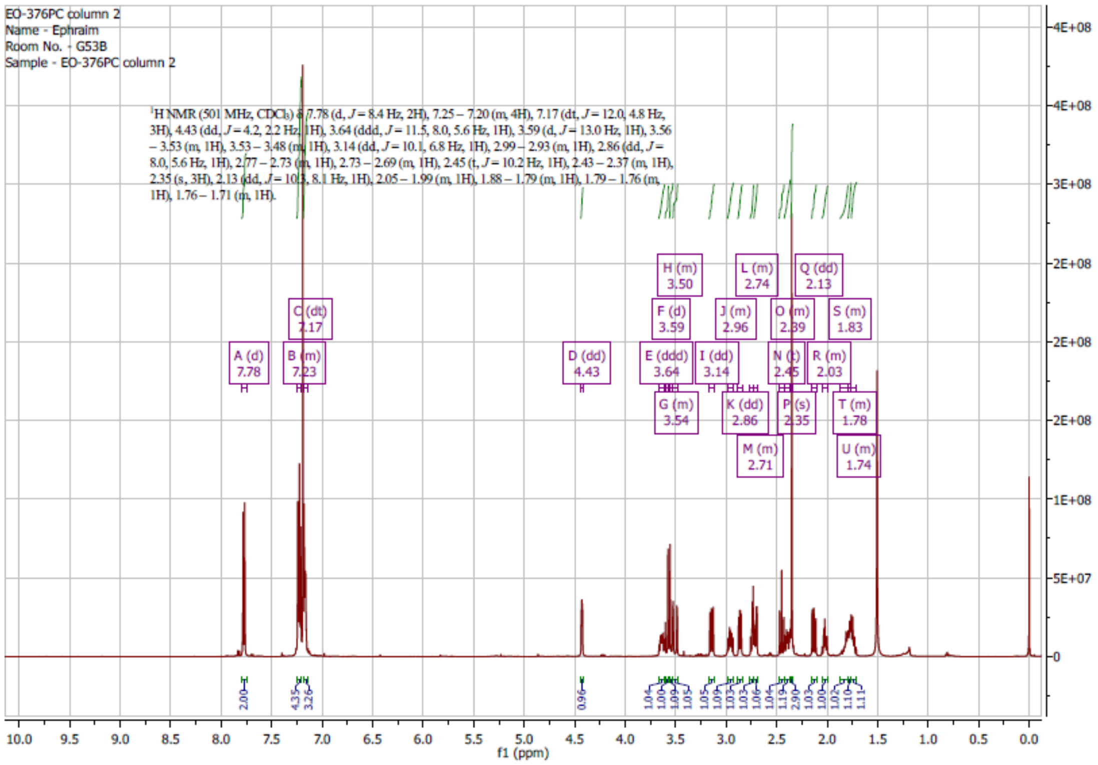

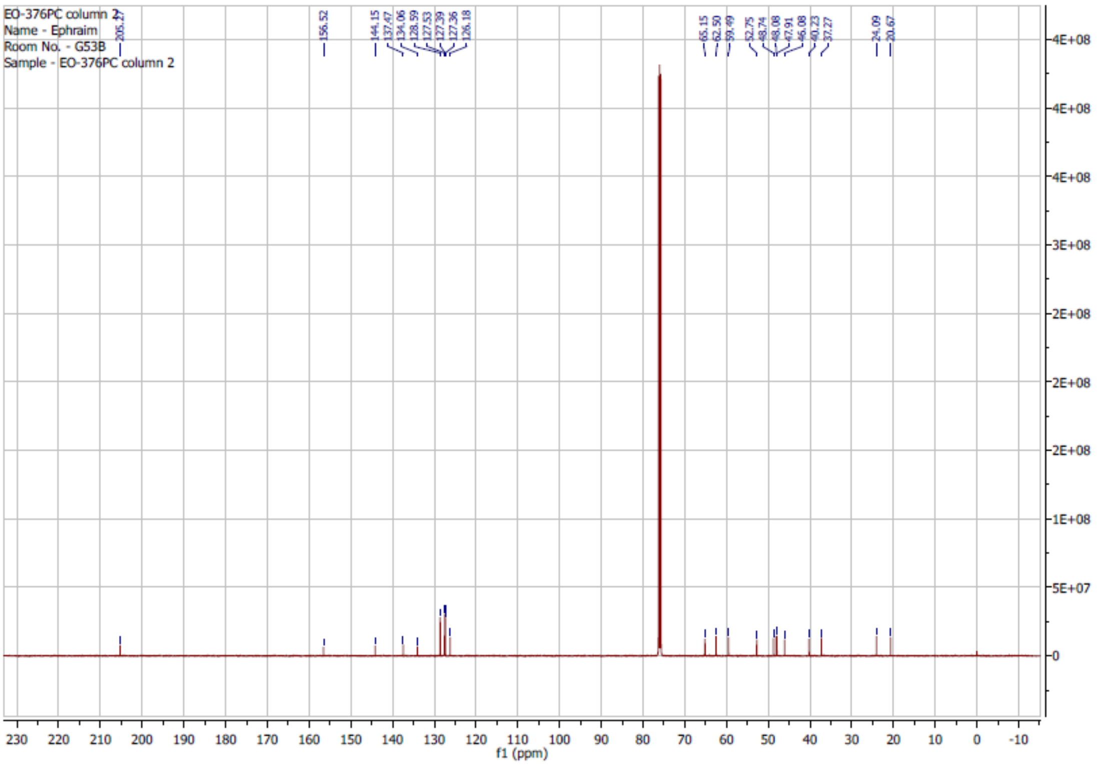

(6a*R,8a*R**,9a*S**,9b*S**)-6-Isopropylhexahydro-1*H*-cyclopropa[5,6]benzo[1,2-*d*]pyrrolo[1,2-c]imidazole-5,8(6*H*, 8a*H*)-dione 10a**

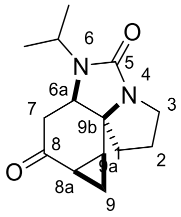

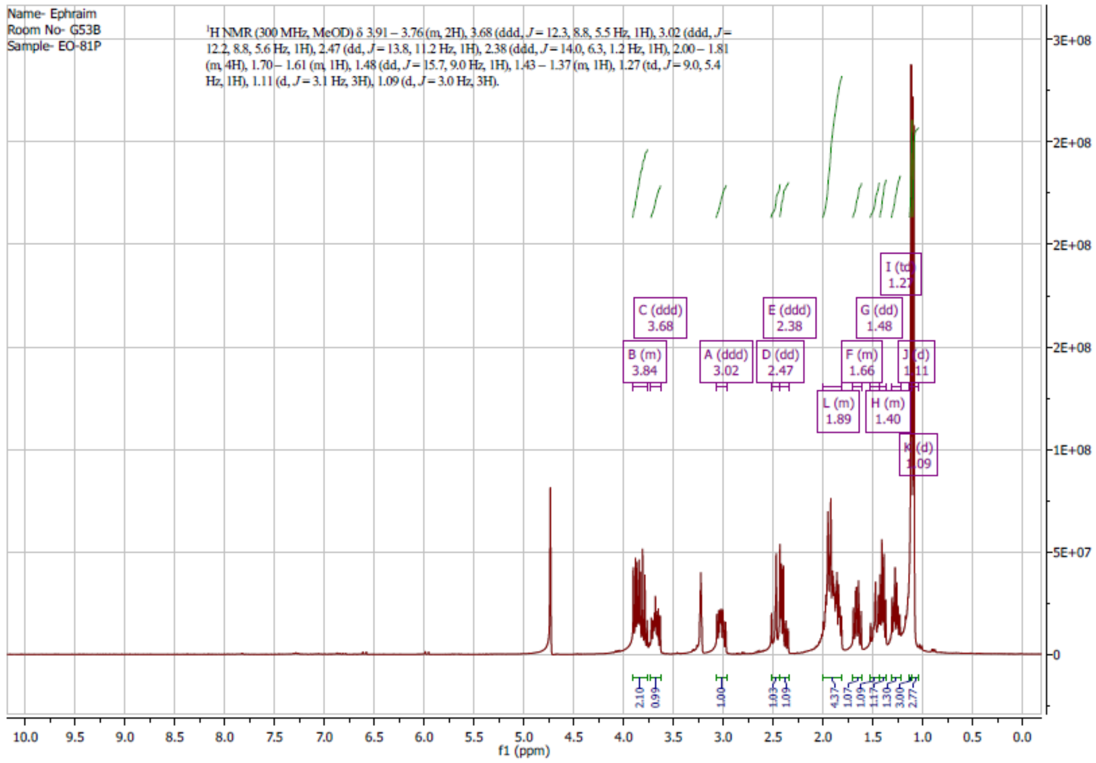

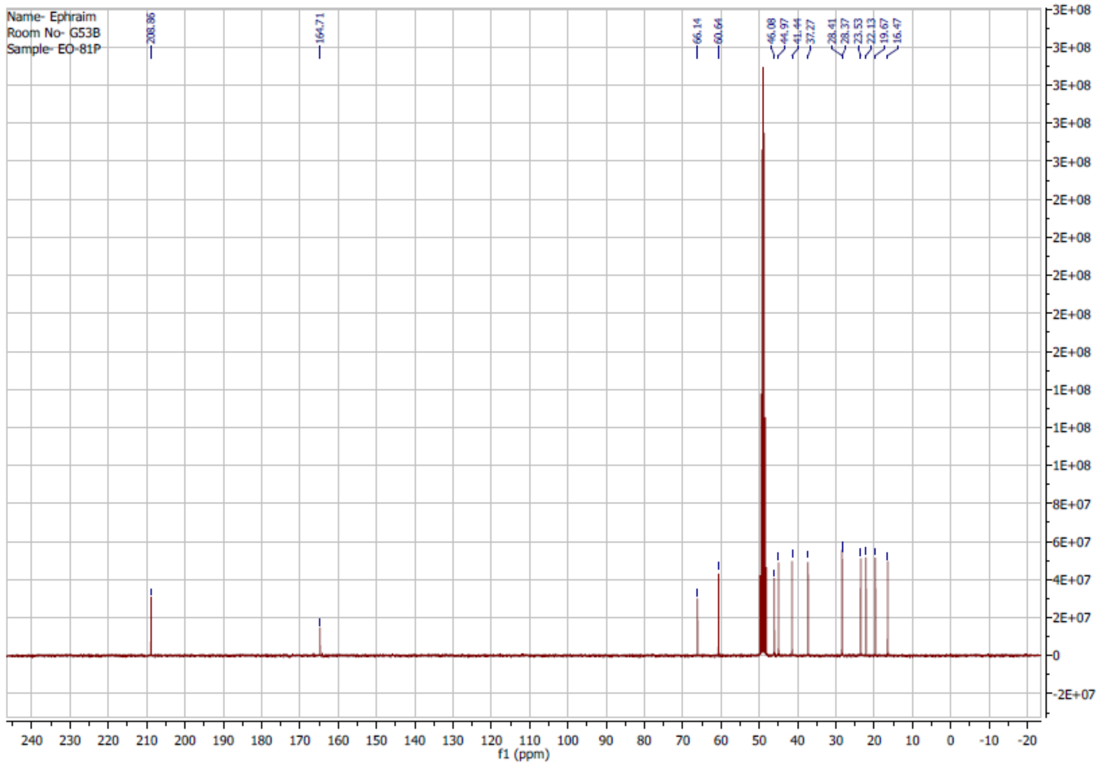

(6a*R,8a*R**,9a*S**,9b*S**)-6-(4-Methoxyphenyl)hexahydro-1*H*-cyclopropa[5,6]benzo[1,2-*d*]pyrrolo[1,2-c]imidazole-5,8(6*H*, 8a*H*)-dione 10b**

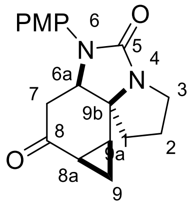

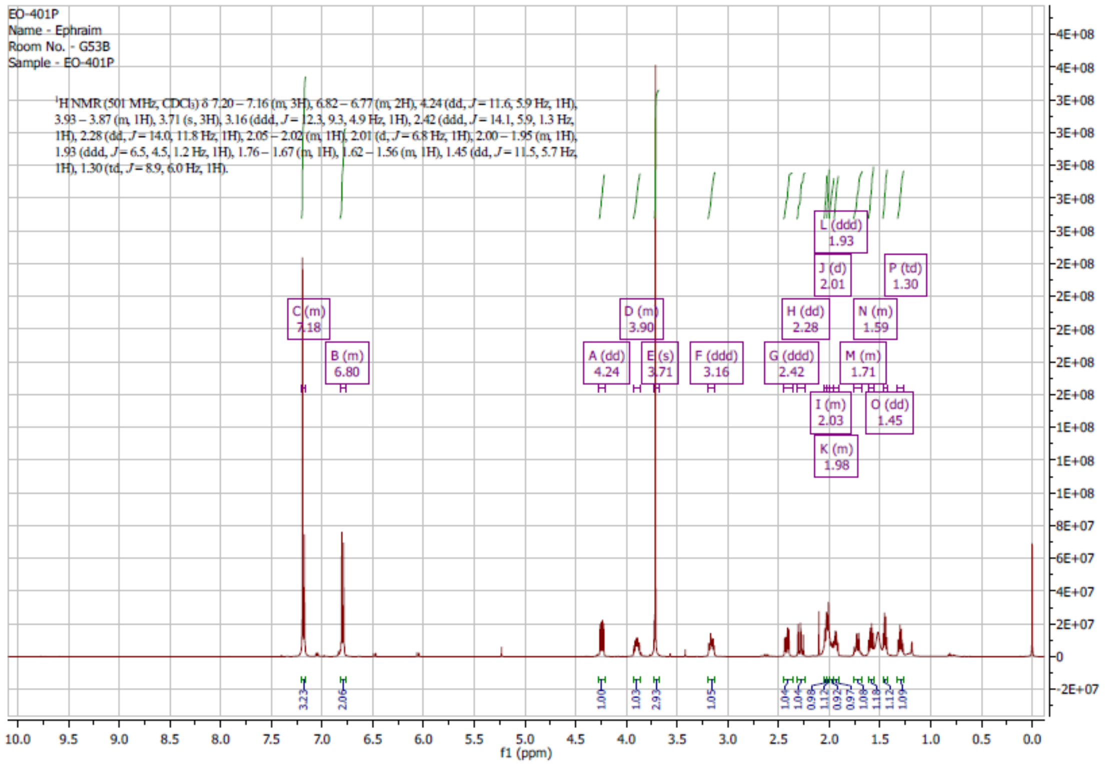

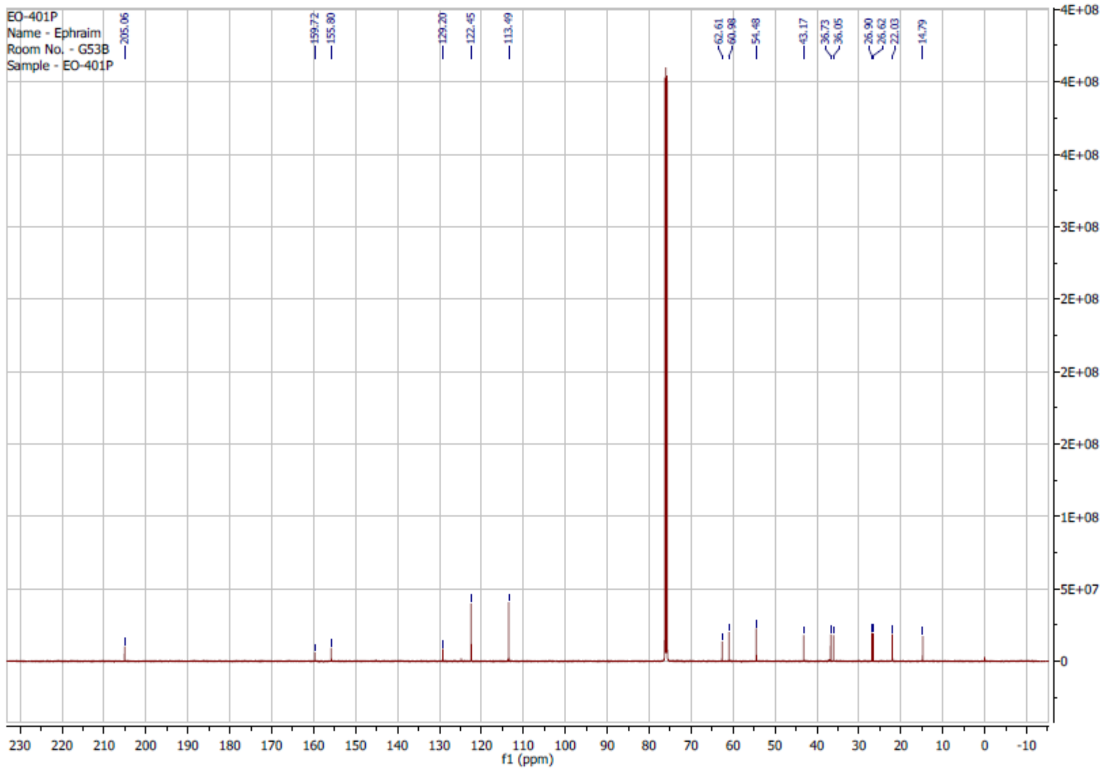

(4b*R, 10a*S**, 10b*S**, 11a*R**)-5-(4-Methoxyphenyl)-4b,9,10,10b,11,11a-hexahydro-8*H*-cyclopropa[*h*]pyrrolo[1',2':1,5]imidazo [4,5-*f*]quinolin-6(5*H*)-one 11b**

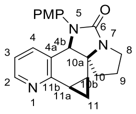

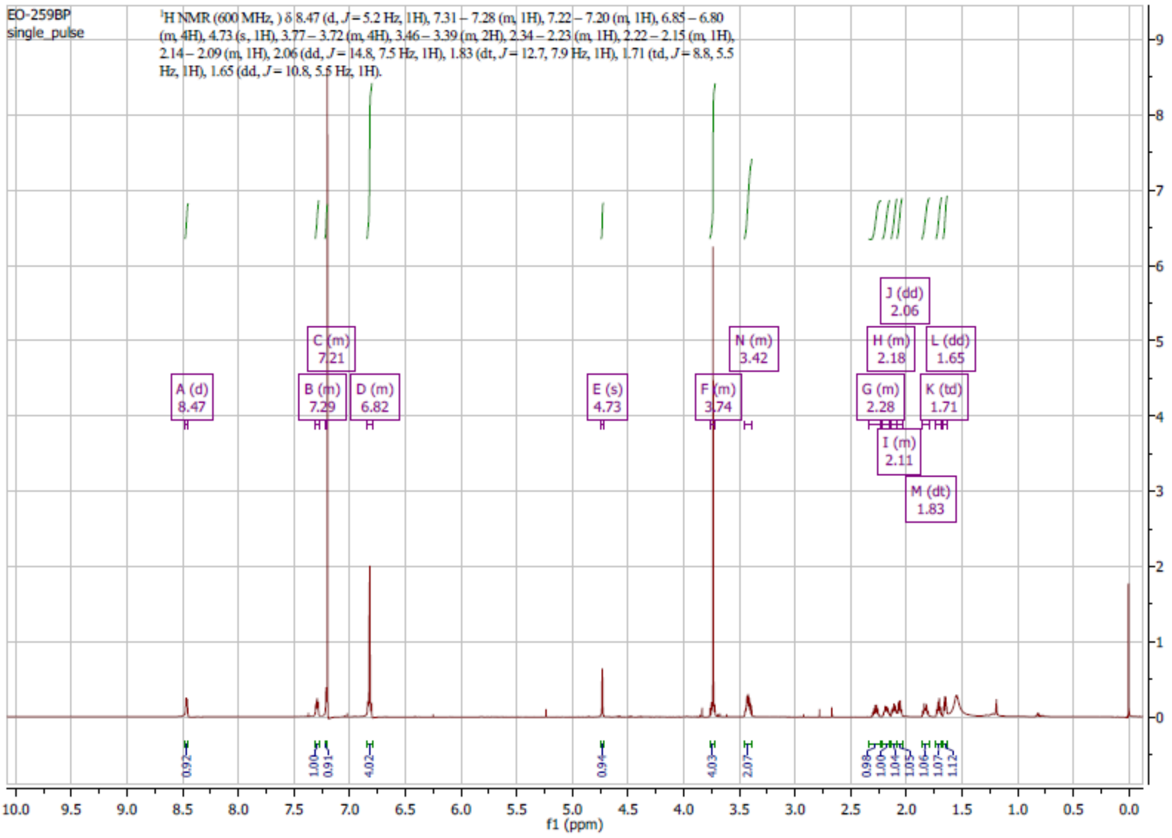

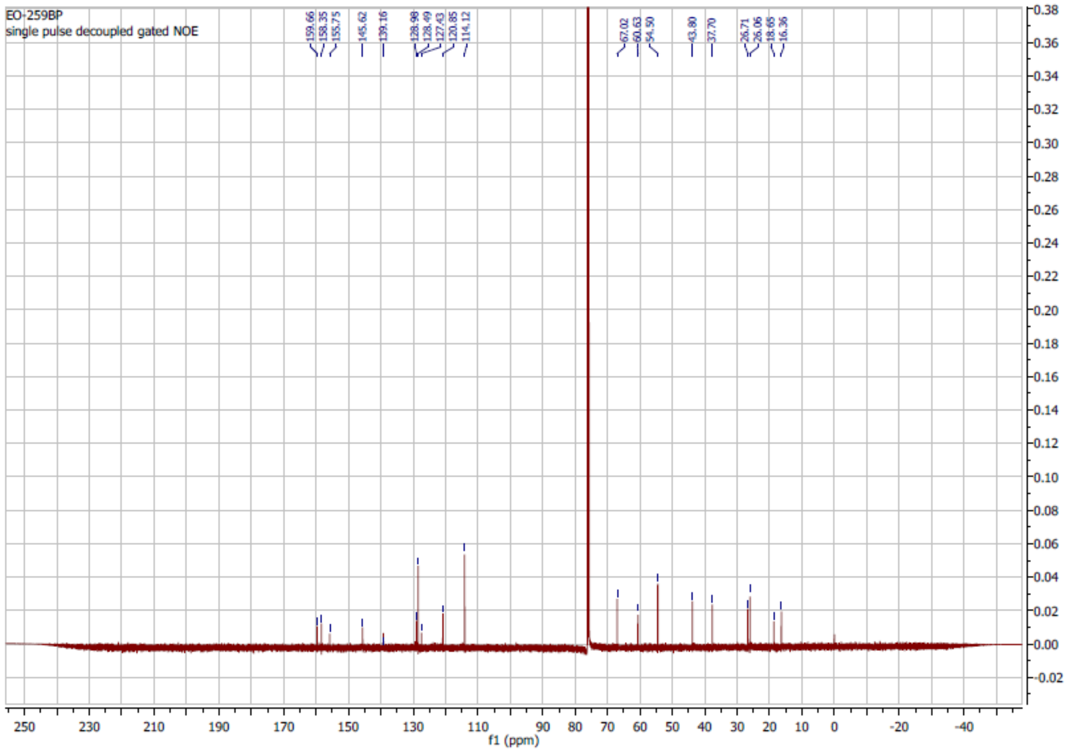

N-(5S*,6R*,8R*)-8-Hydroxy-1-methyl-1-azaspiro[4.5]decan-6-yl)-4-methylbenzenesulfonamide 12c

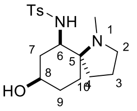

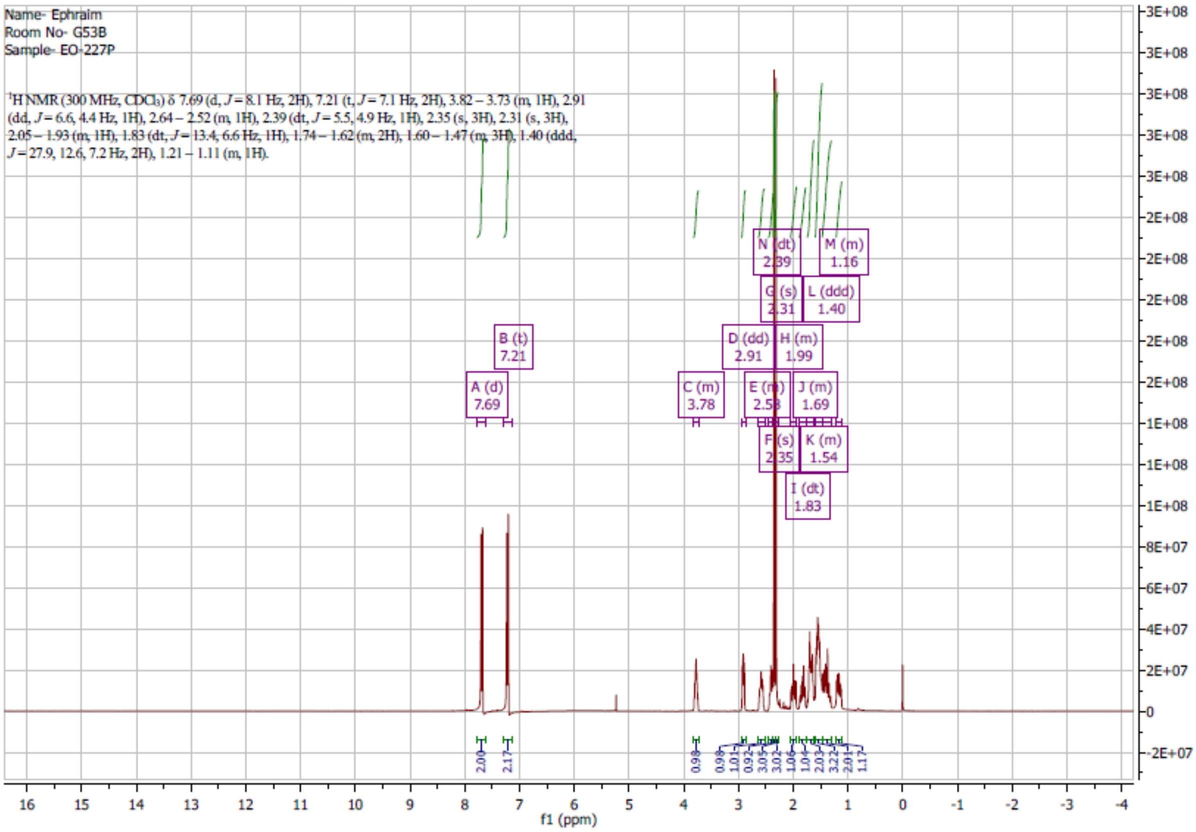

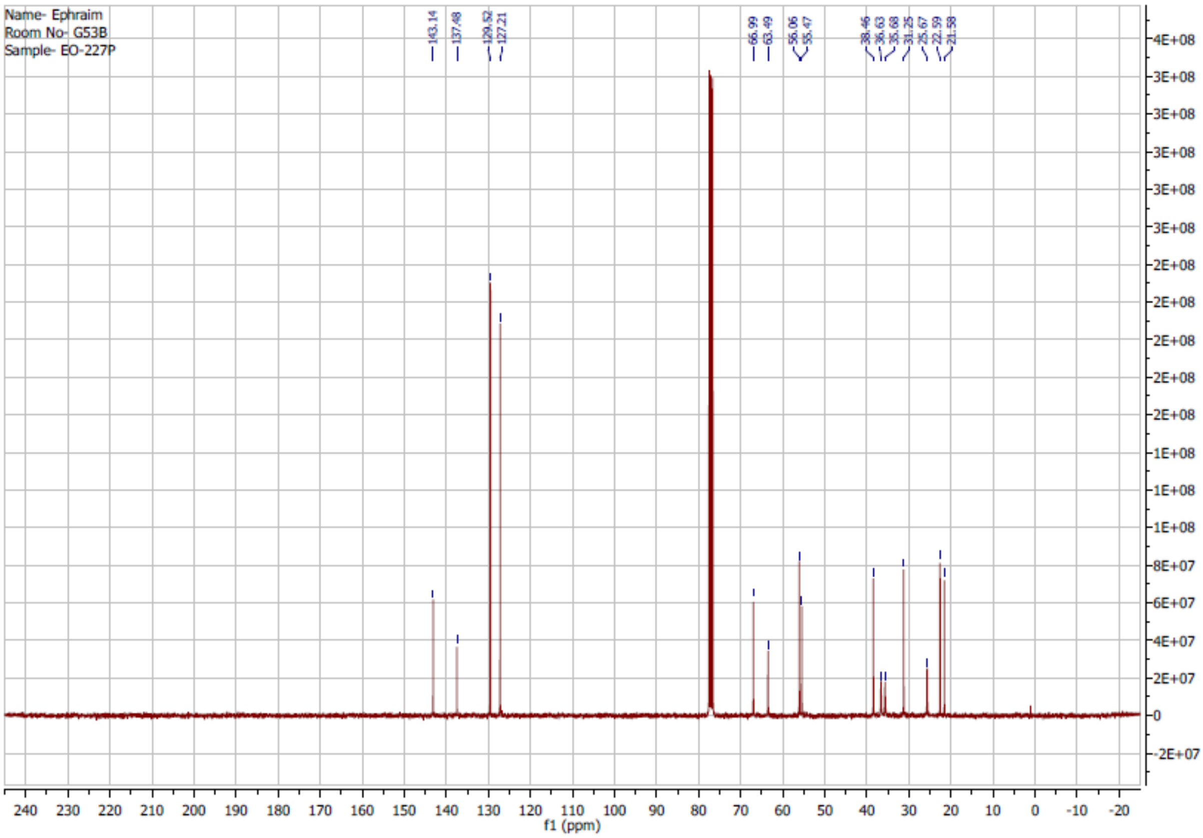

(5*S**,6*R**,8*R**)-6-(Isopropylamino)-1-methyl-1-azaspiro[4.5]decan-8-ol 13a

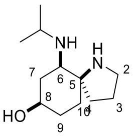

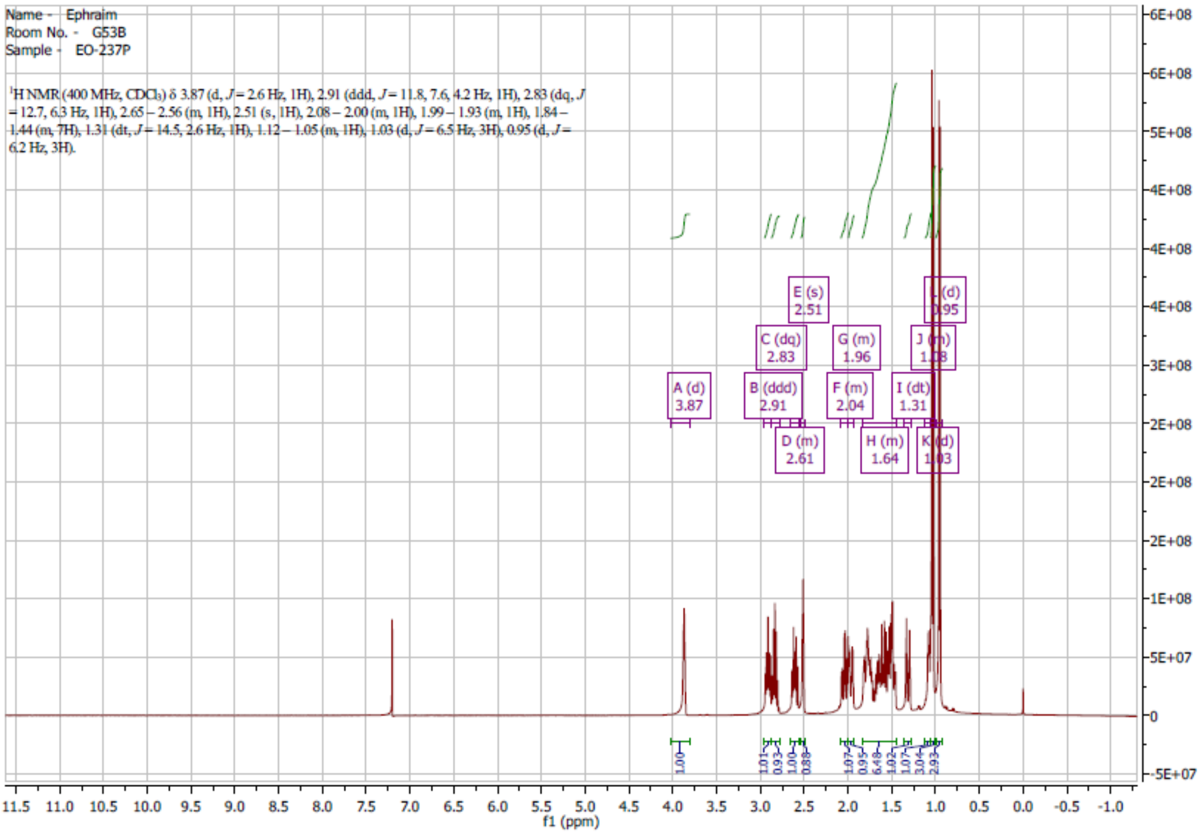

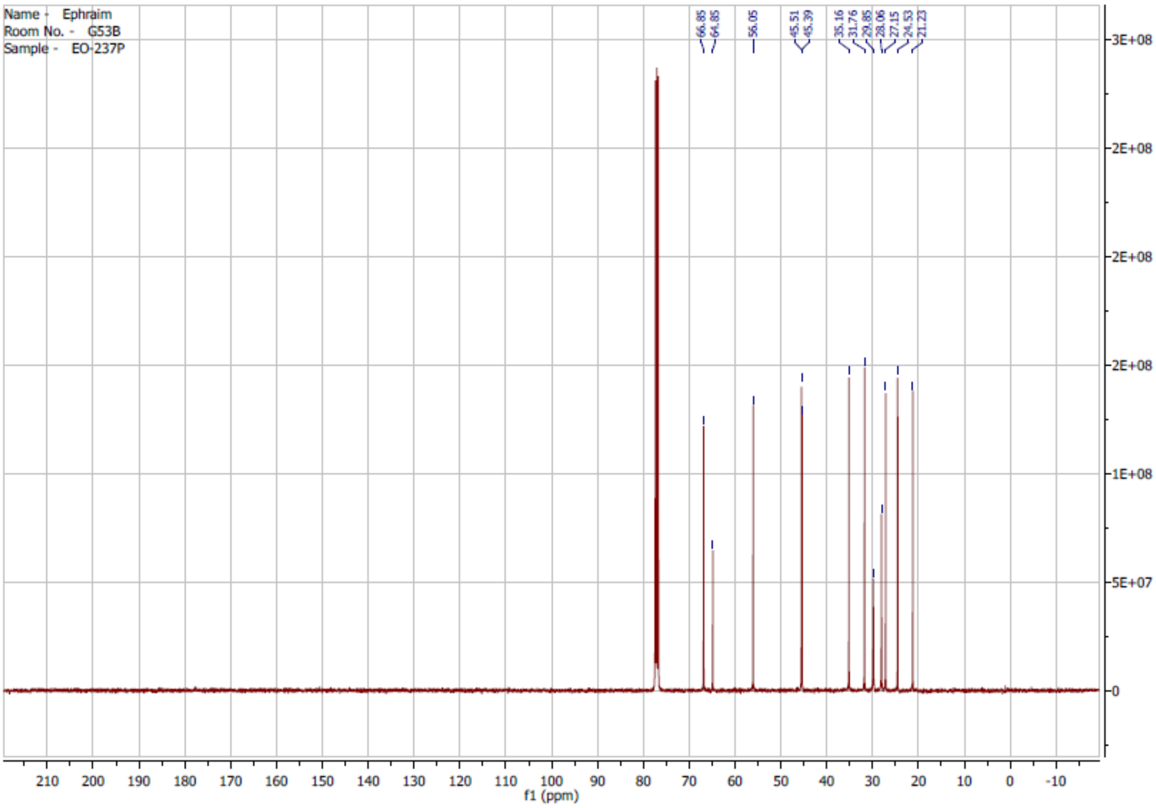

5*S,6*R**,8*R**)-6-(4-Methoxyphenyl)amino-1-azaspiro[4.5]decan-8-ol 13b**

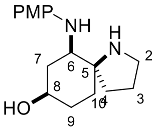

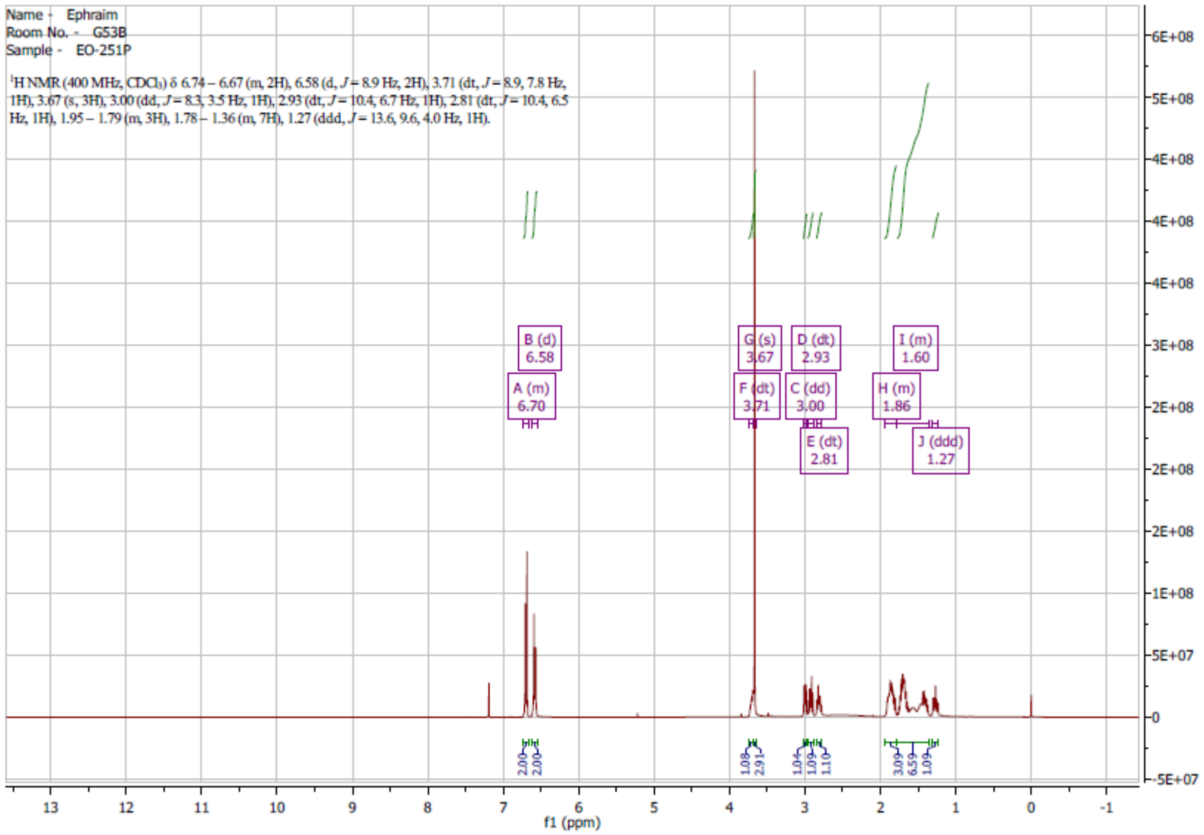

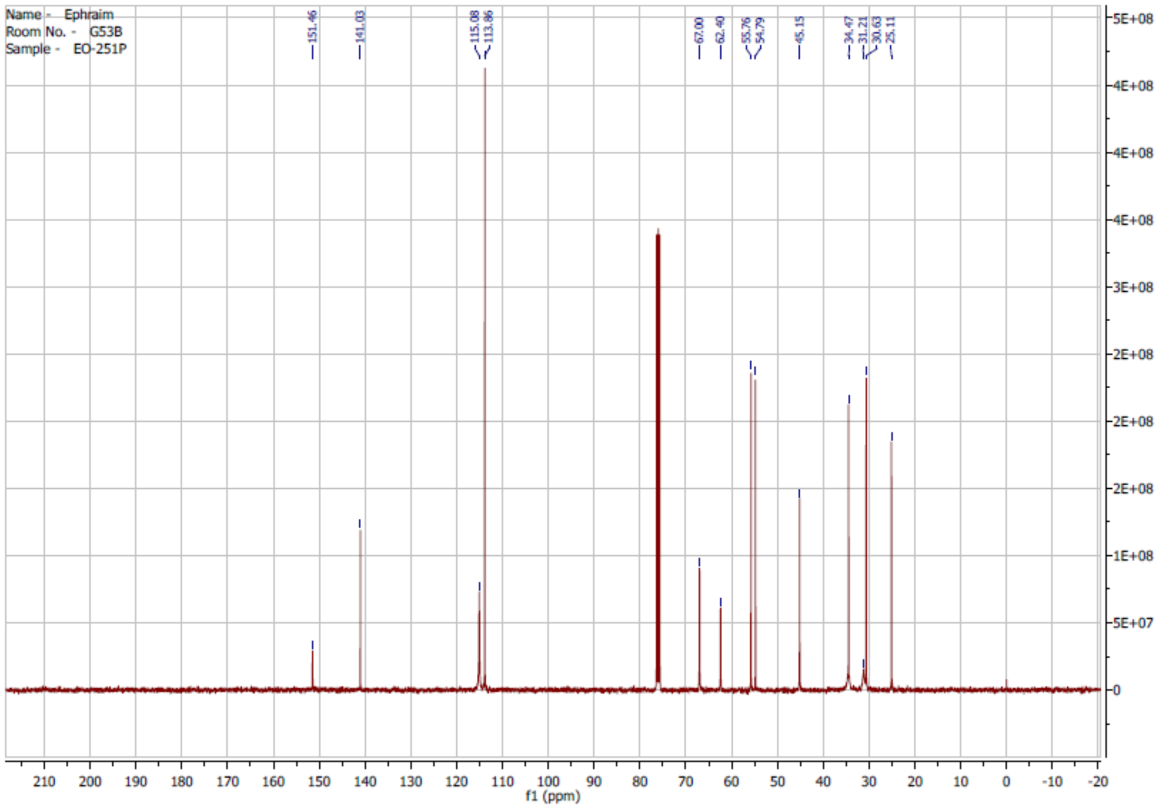

(6a*R,8*S**,10a*S**)-8-((*tert*-Butyldimethylsilyl)oxy)-6-isopropyl-2,3,6,6a,7,8-hexahydro-1*H*,5*H*-benzo[*d*]pyrrolo[1,2-*c*]imidazol-5-one S7**

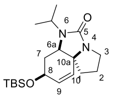

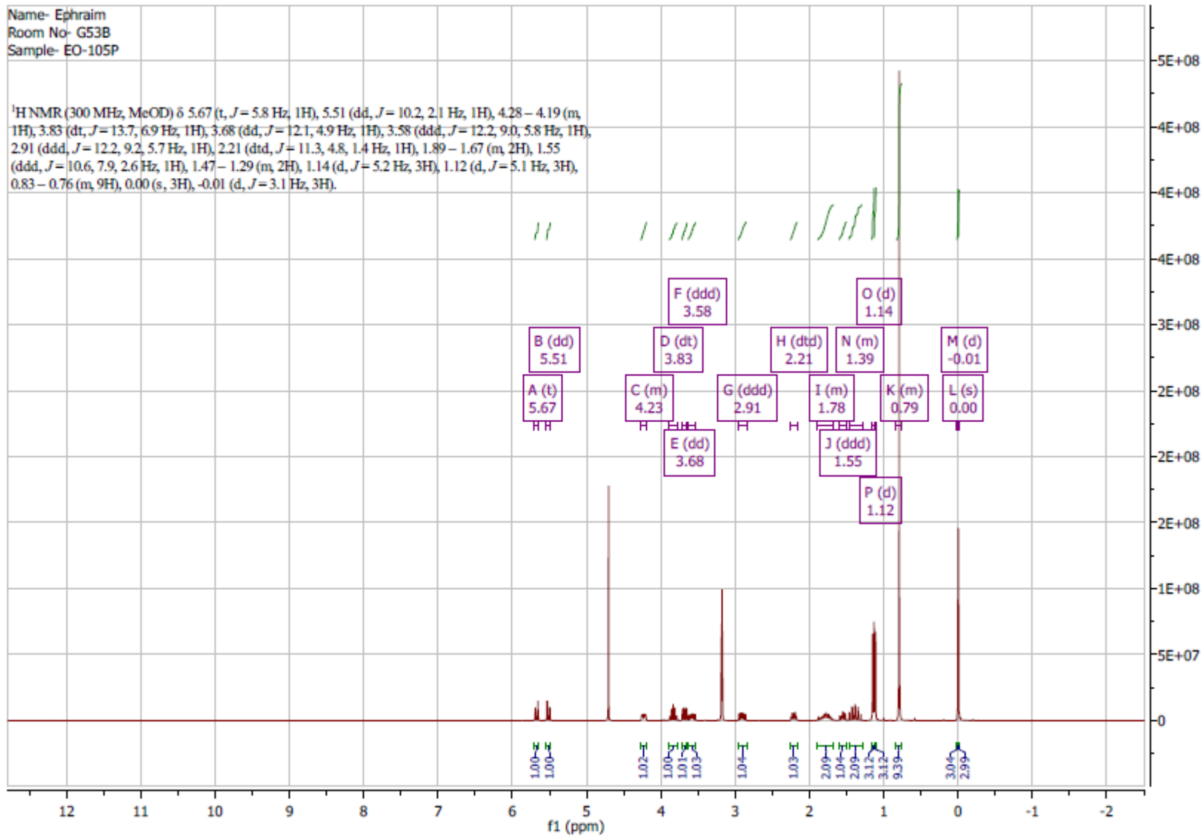

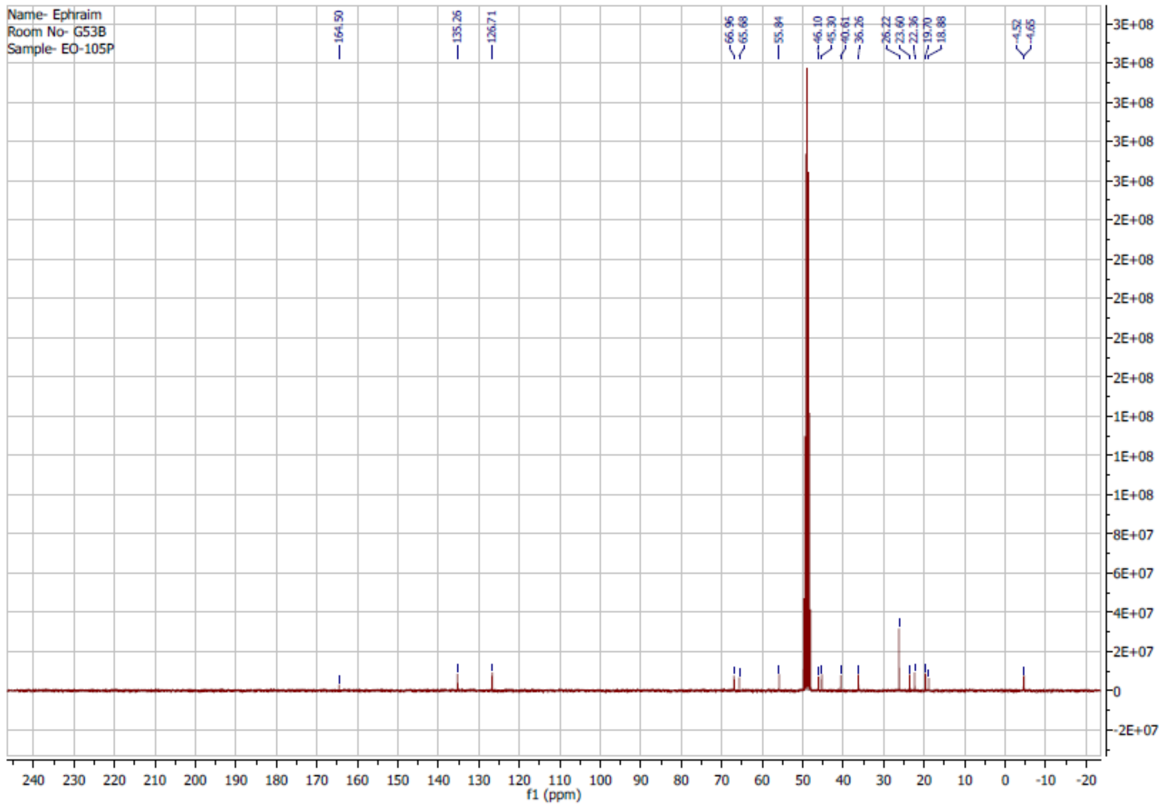

(1*R, 7a*R**)-1-(2,3-Dihydoxypropyl)-7a-(hydroxymethyl)-2-isopropylhexahydro-3*H*-pyrrolo[1,2-*c*]imidazole-3-one 14a**

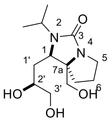

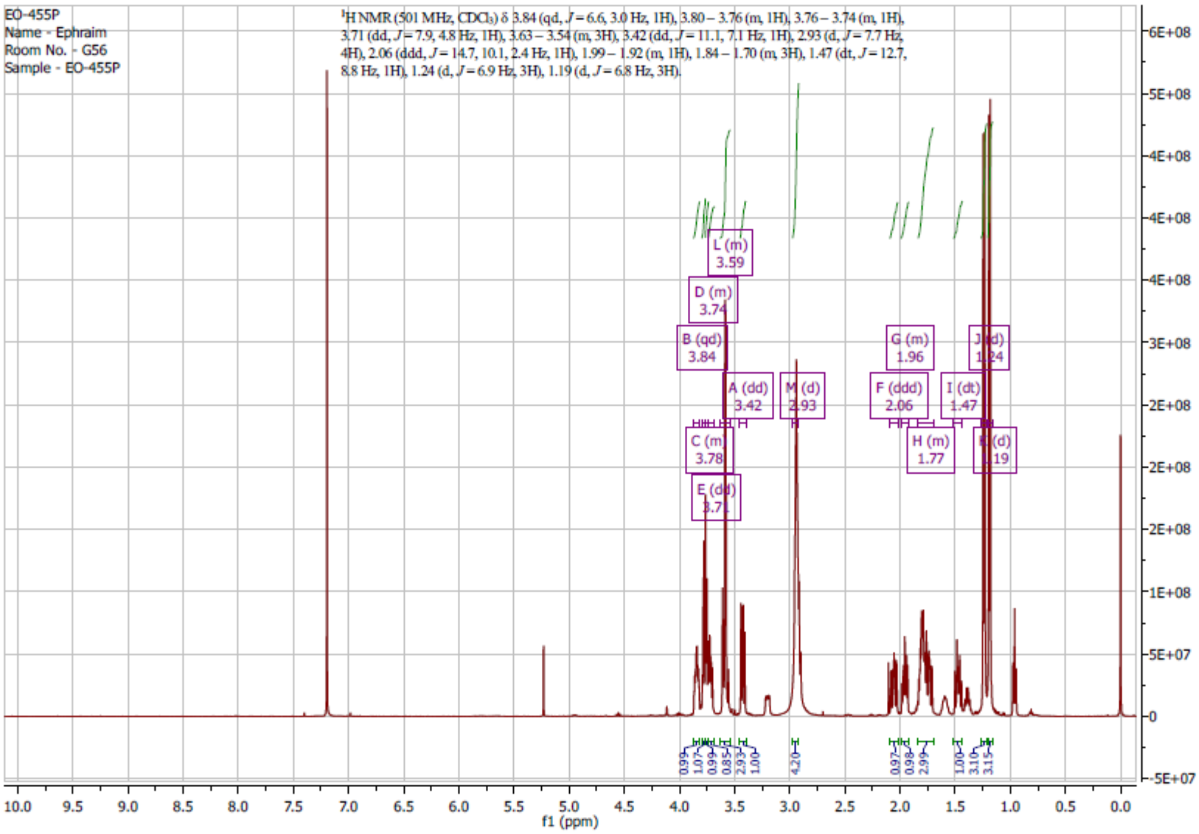

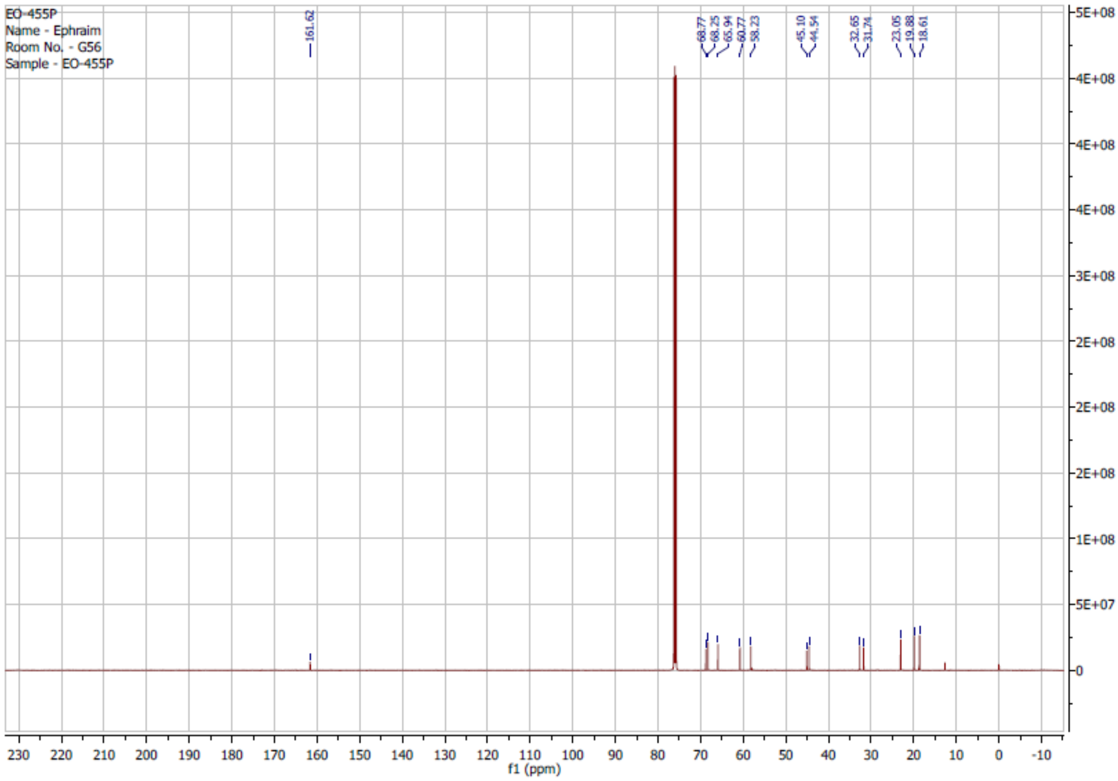

(7a*R, 9*R**, 11a*S**)-9-Hydroxy-7-(4-methoxyphenyl)octahydro-1*H*-pyrrolo[1,2-*d*]quinoxalin-5(6*H*)-one 15b**

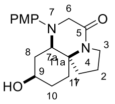

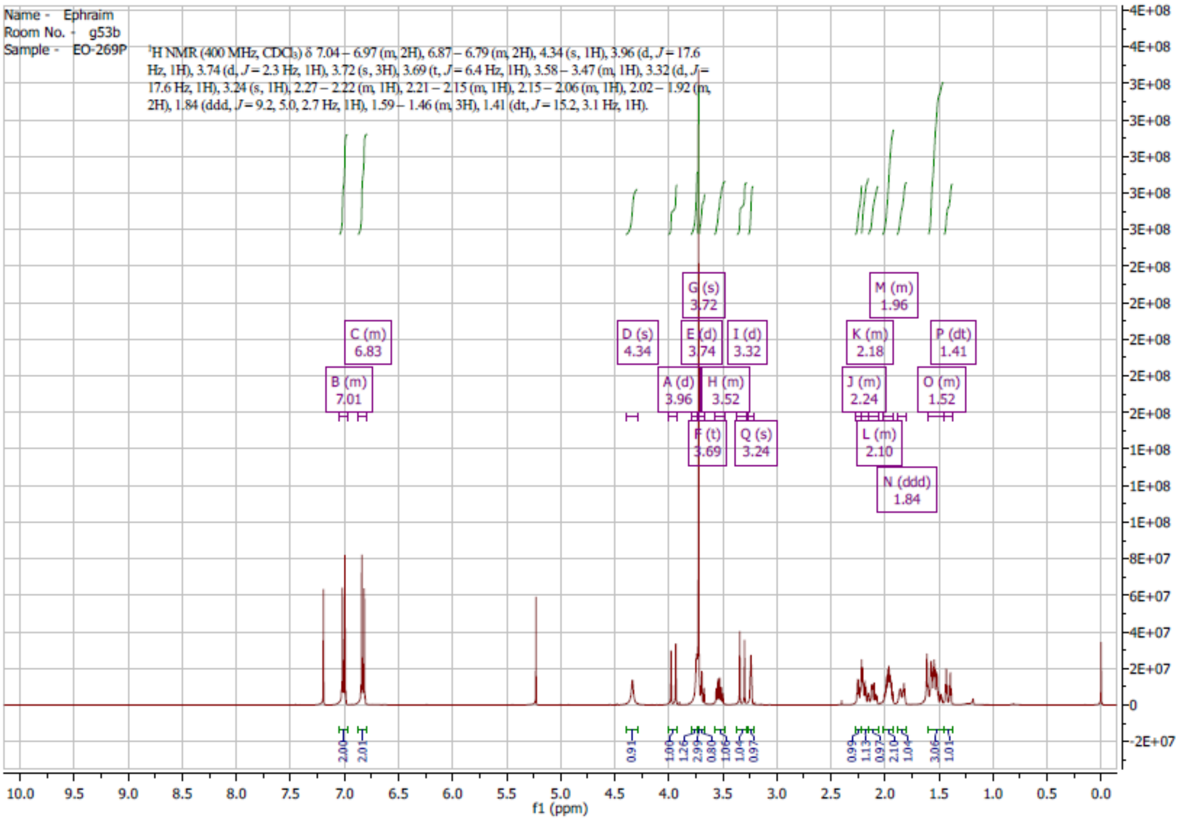

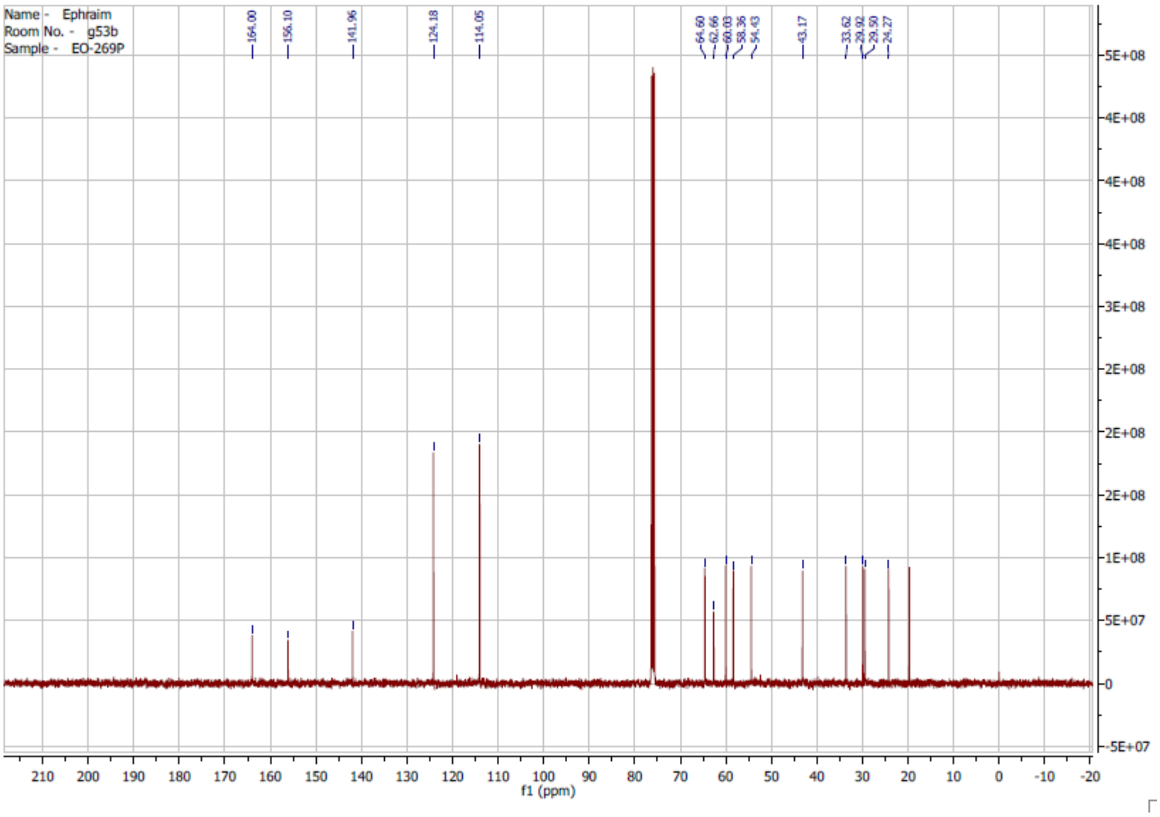

Methyl 2-((5*S, 6*R**, 8*R**)-8-Hydroxy-6-((4-methoxyphenyl)amino)-1-azaspiro[4.5]decan-1-yl)acetate S8**

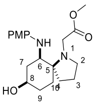

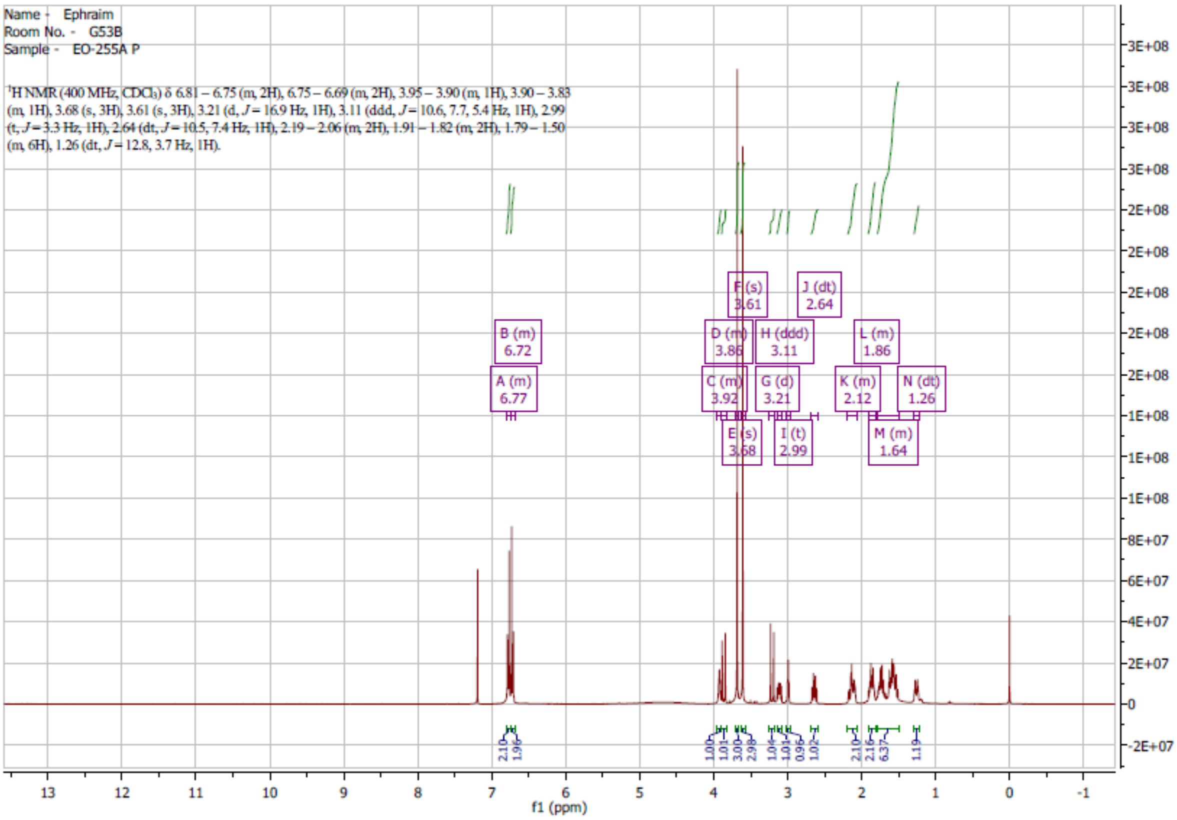

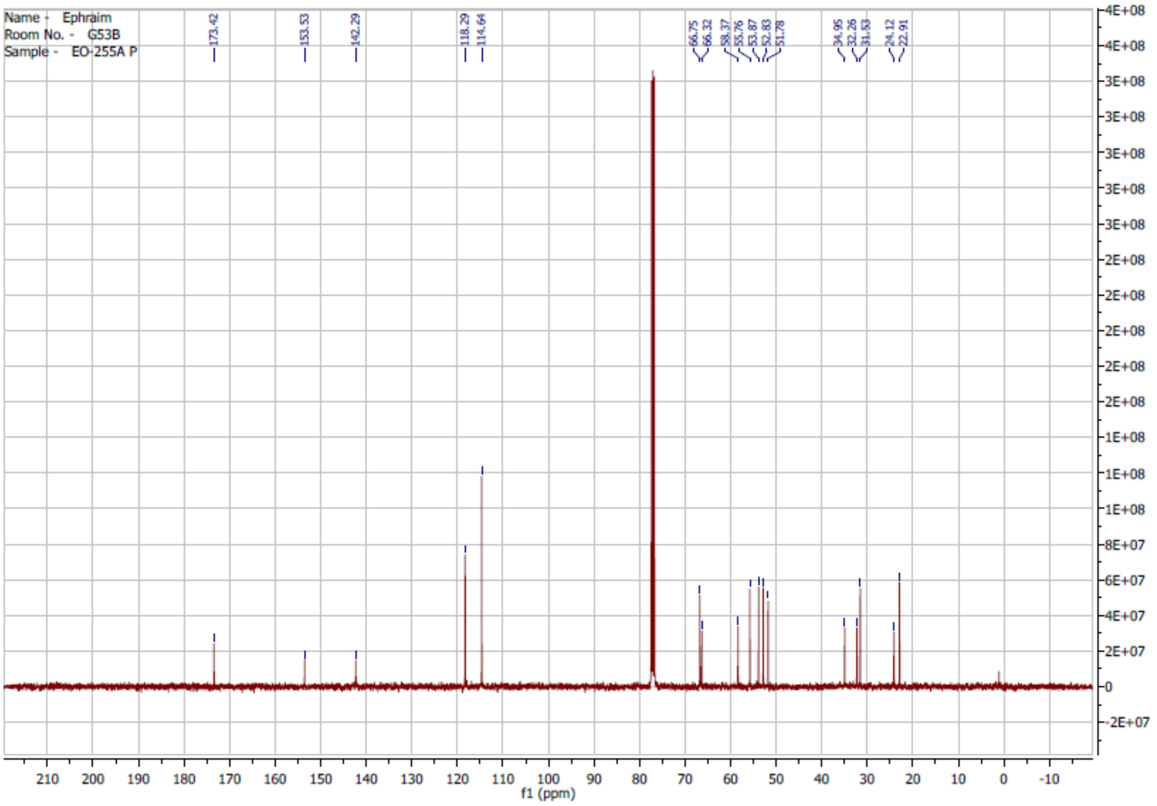

(7a*R,9*R**,11a*S**)-9-Hydroxy-7-(4-methoxyphenyl)octahydro-1H-pyrrolo[1,2-*d*]quinoxalin-6(5*H*)-one 16b**

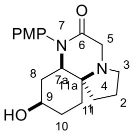

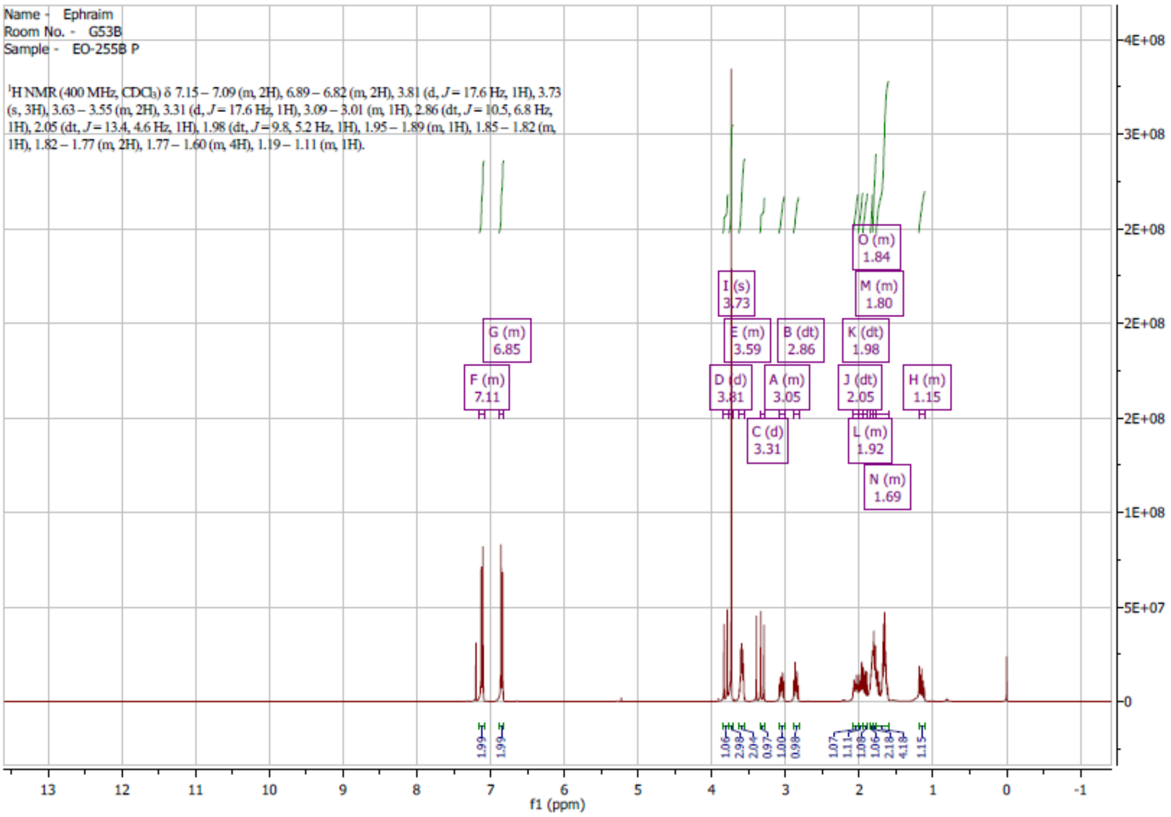

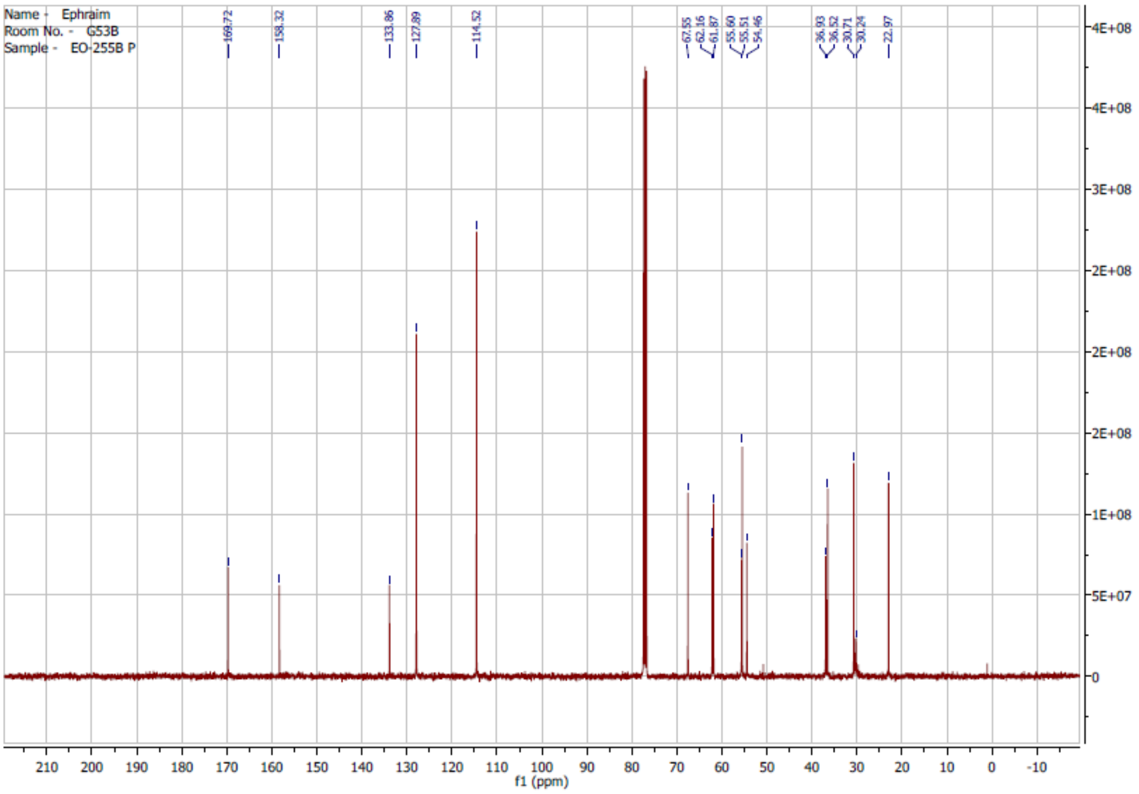

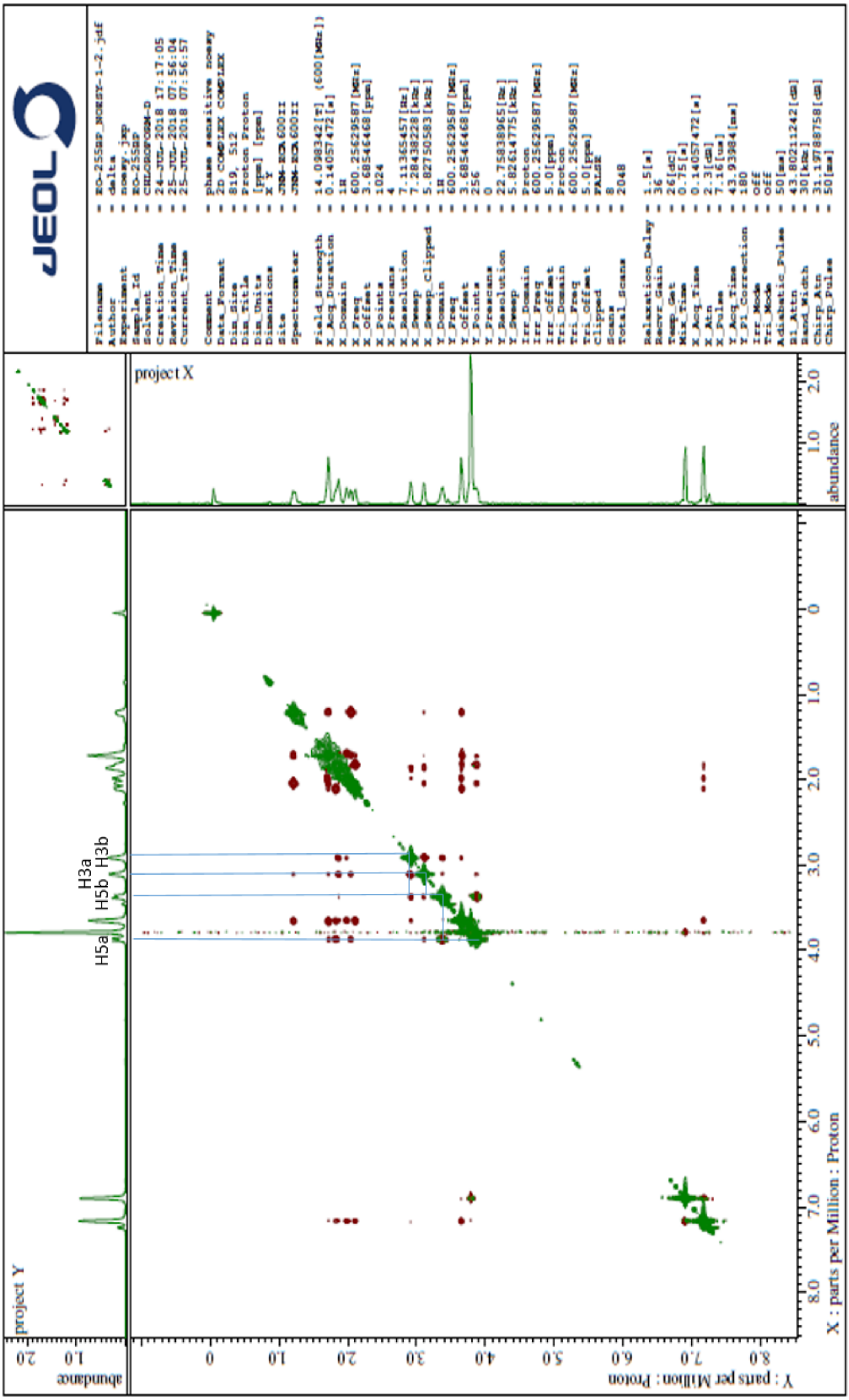

JEOL
project X
project Y
abundance
X : parts per Million : Proton
Y : parts per Million : Proton
H3a
H3b
H5b
H5a

(5a*R,11a*S**)-6-Isopropyloctahydro-9*H*-pyrrolo[1',2':1,5]imidazo[4,5-*c*]azepine-3,7-dione 17a**

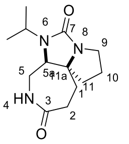

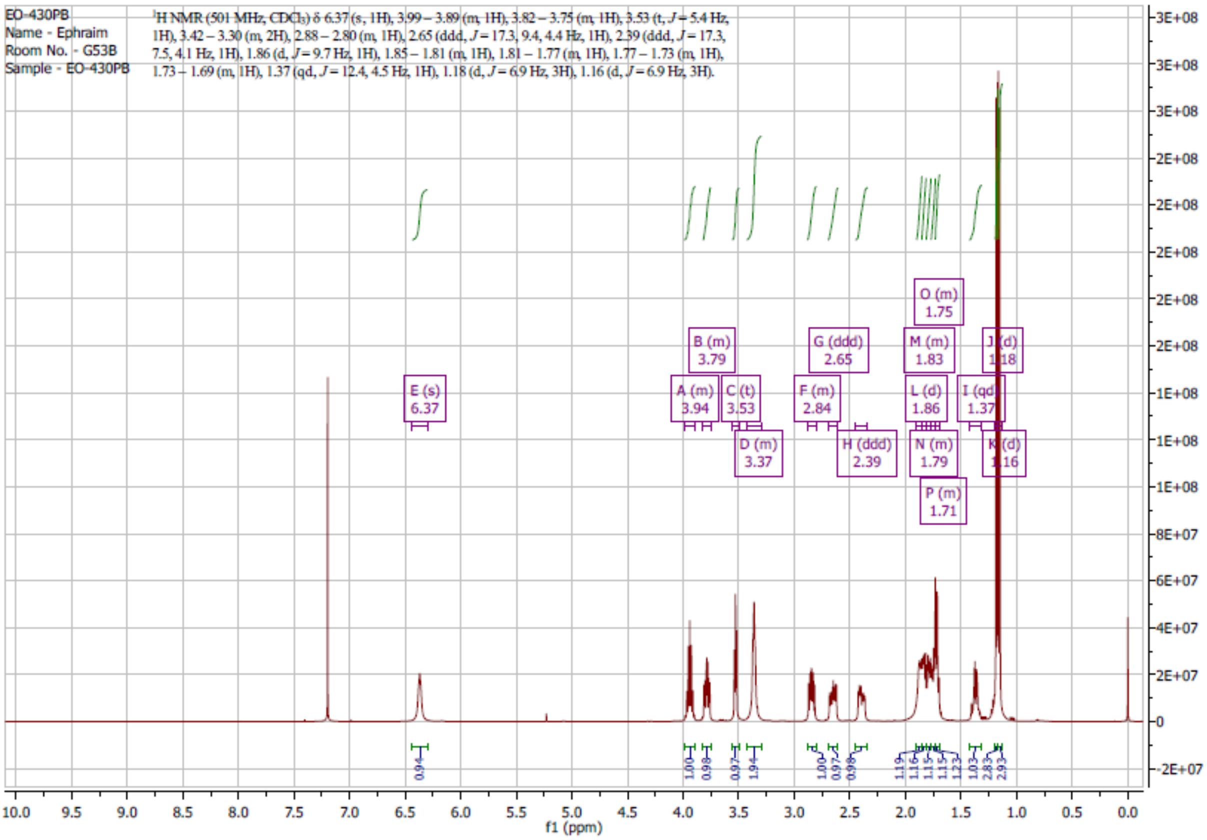

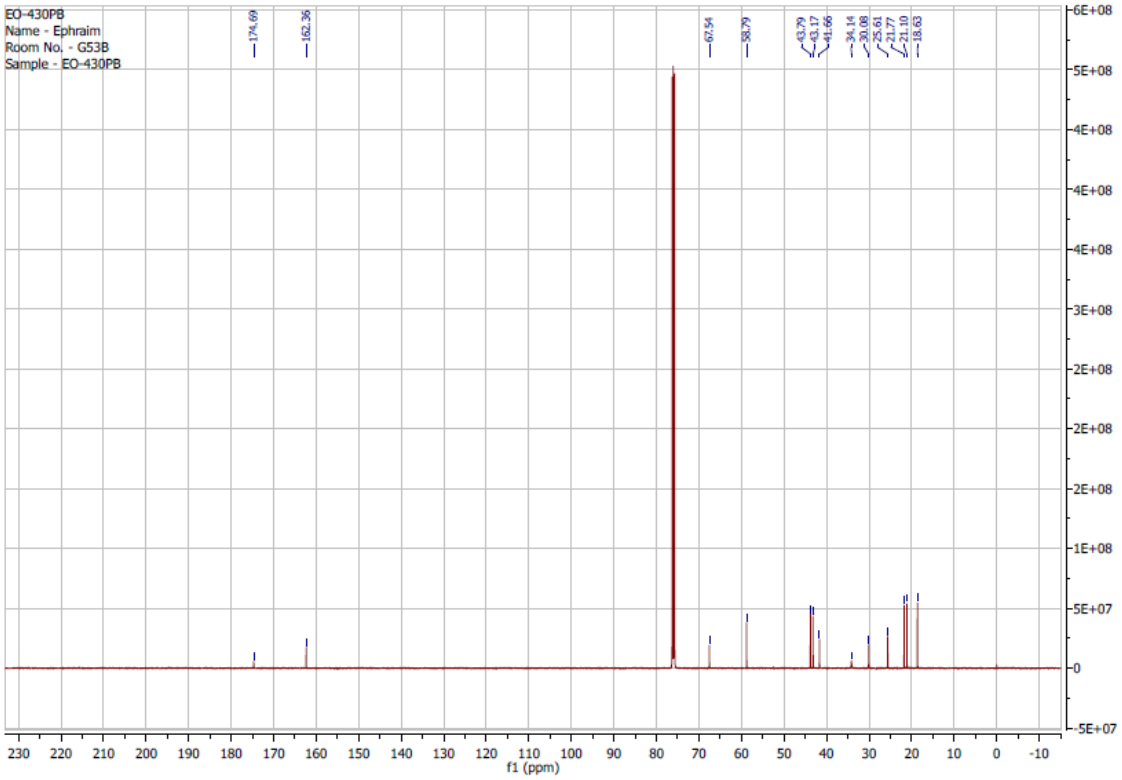

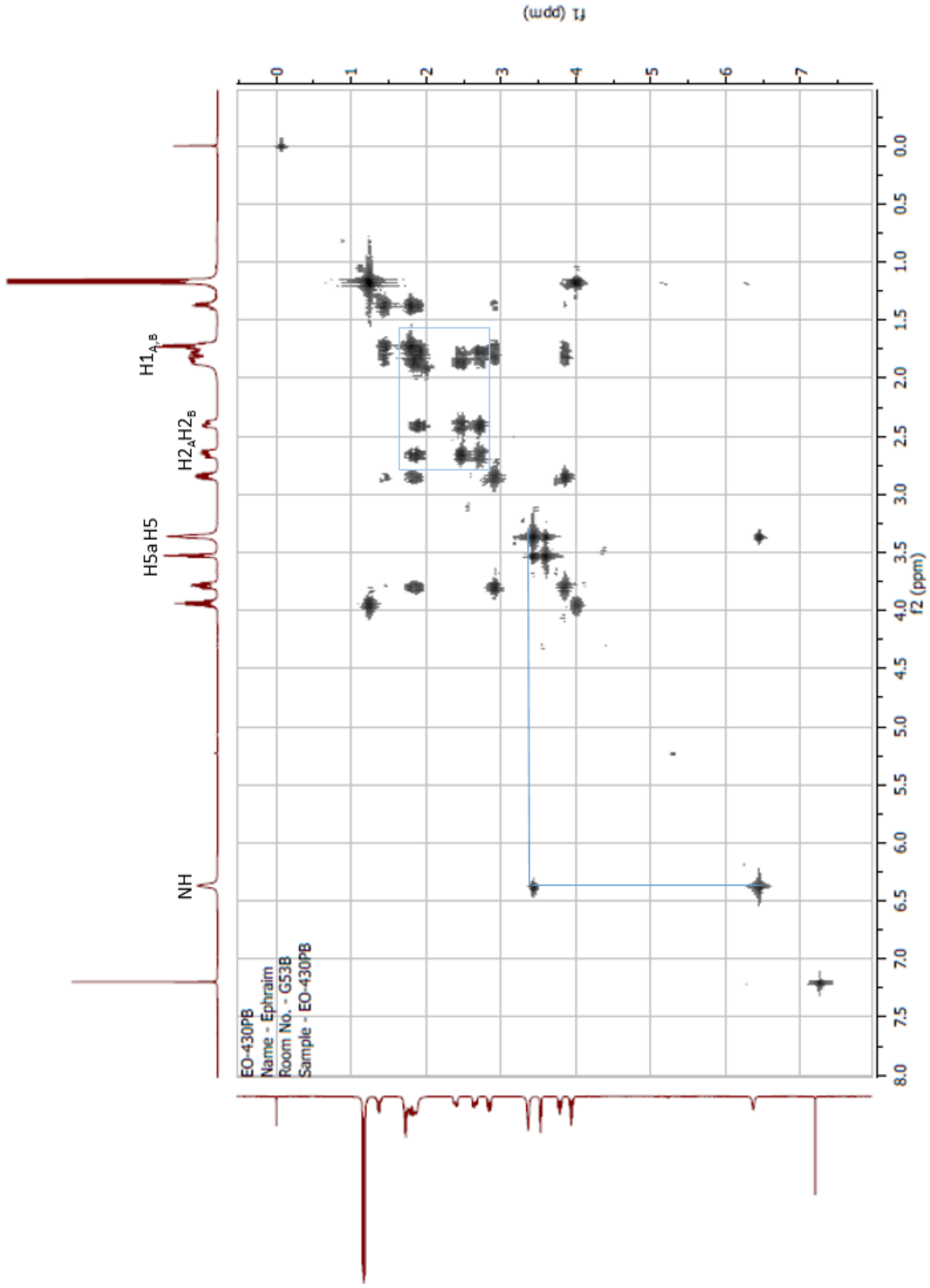
H1A,B
H2A H2B
H5a H5
NH
EO-430PB
Name - Ephraim
Room No. - G53B
Sample - EO-430PB
f1 (ppm)
f2 (ppm)

(5a*R,11a*S**)-6-(4-Methoxyphenyl)octahydro-9*H*-pyrrolo[1',2':1,5]imidazo[4,5-*c*]azepine-3,7-dione 17b**

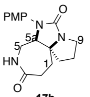

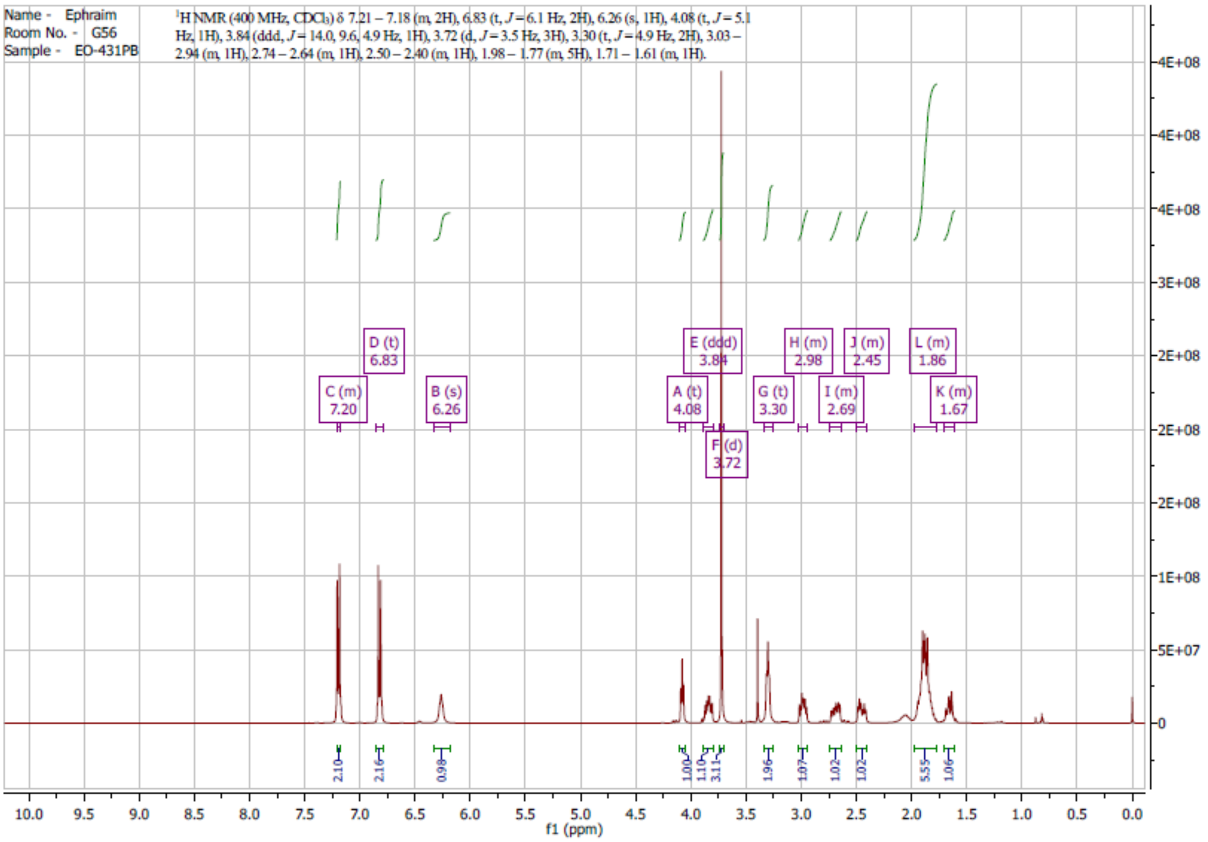

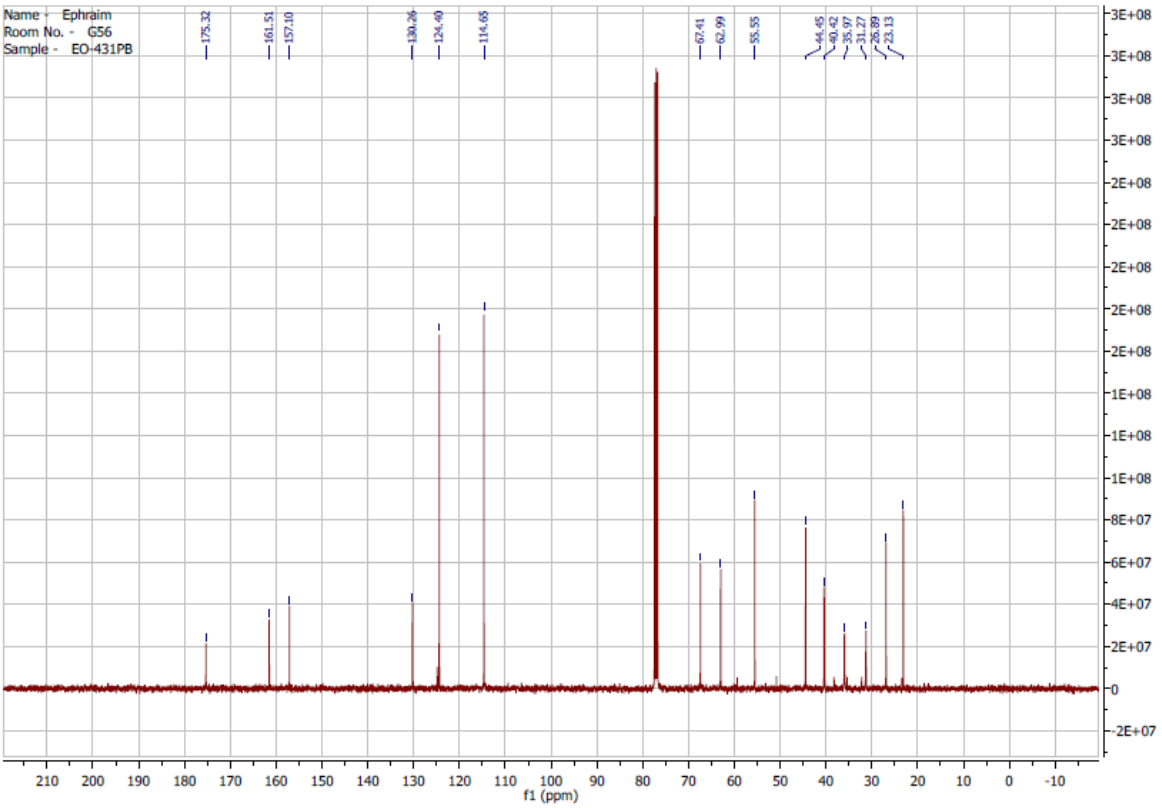

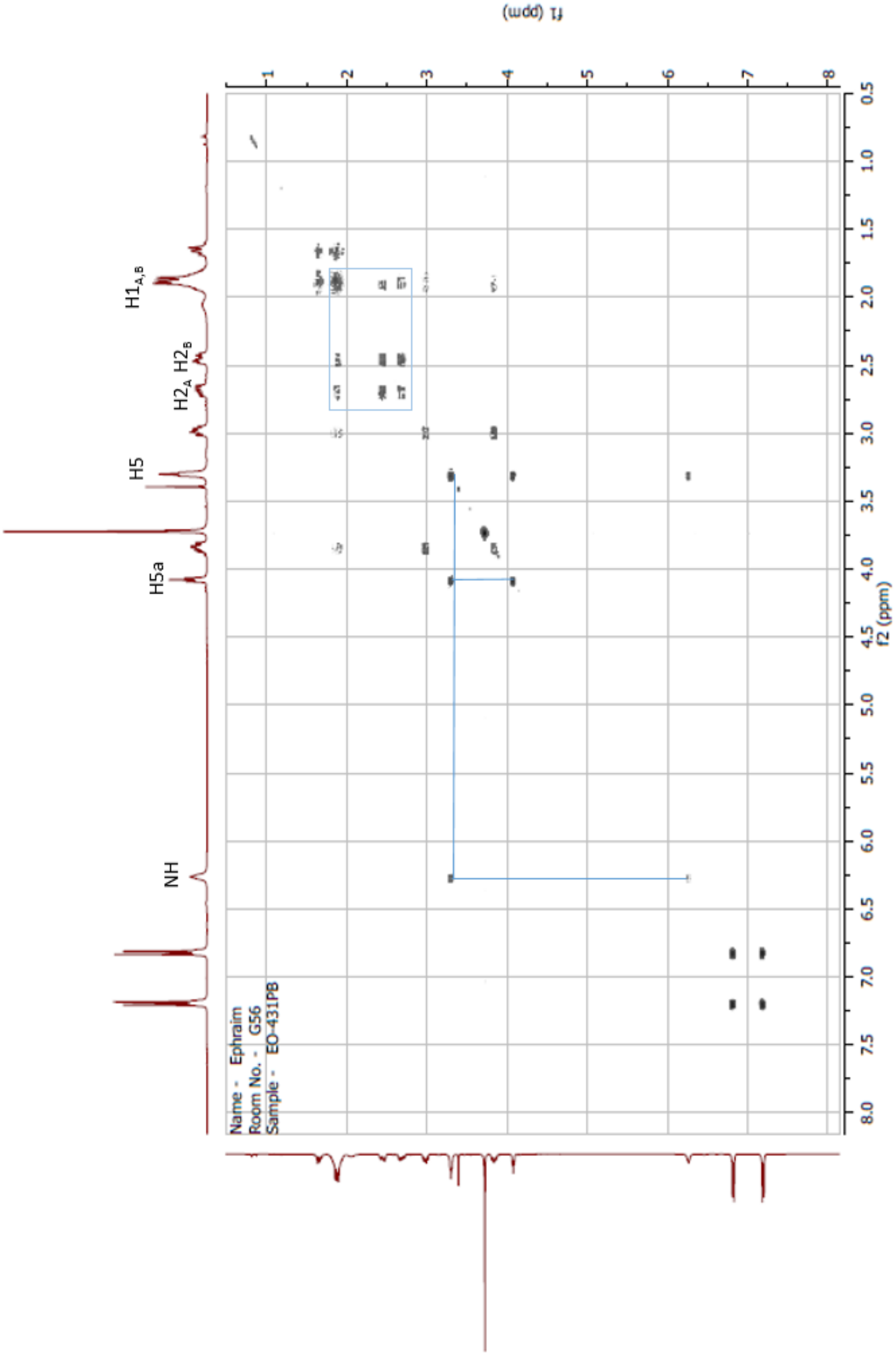
H1A,B
H2A H2B
H5
H5a
NH
f1 (ppm)
f2 (ppm)
Name - Ephraim
Room No. - G56
Sample - EO-431PB

(5a*R,11a*S**)-6-(4-Toluenesulfonyl)octahydro-9*H*-pyrrolo[1',2':1,5]imidazo[4,5-*c*]azepine-3,7-dione 17c**

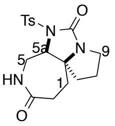

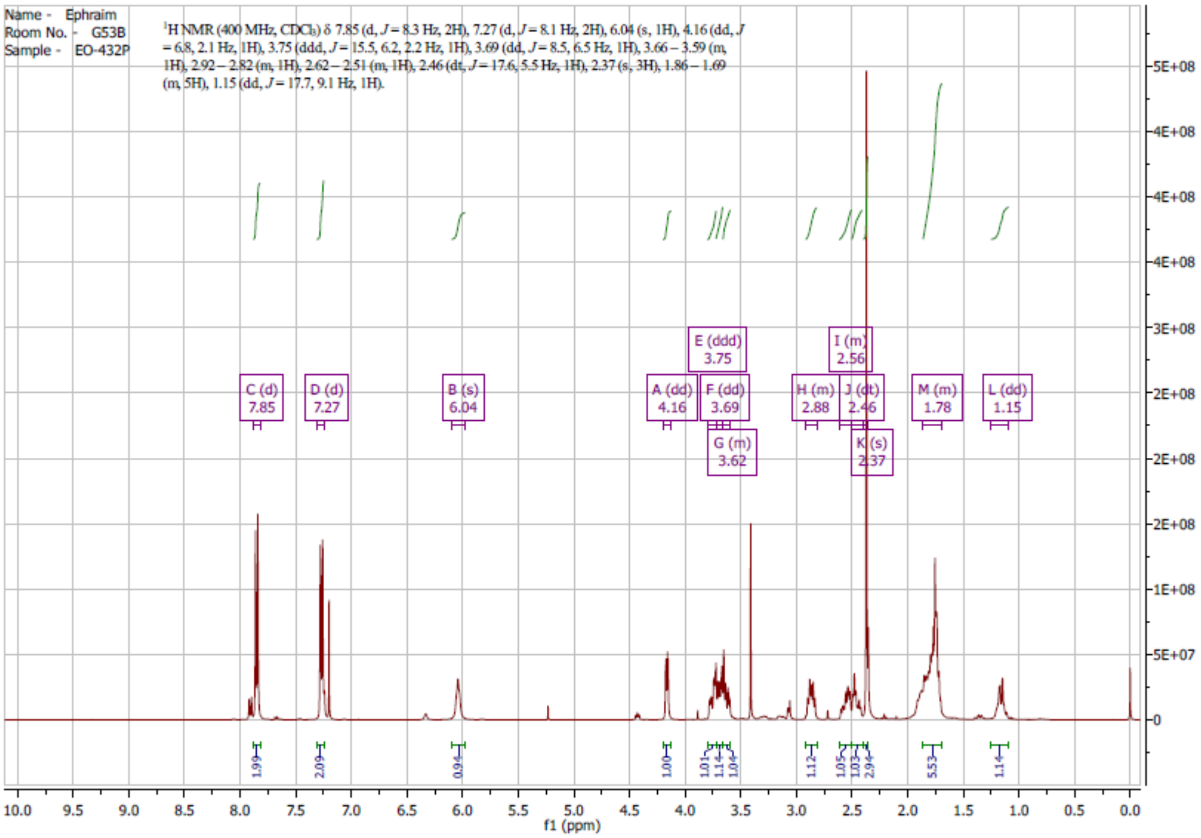

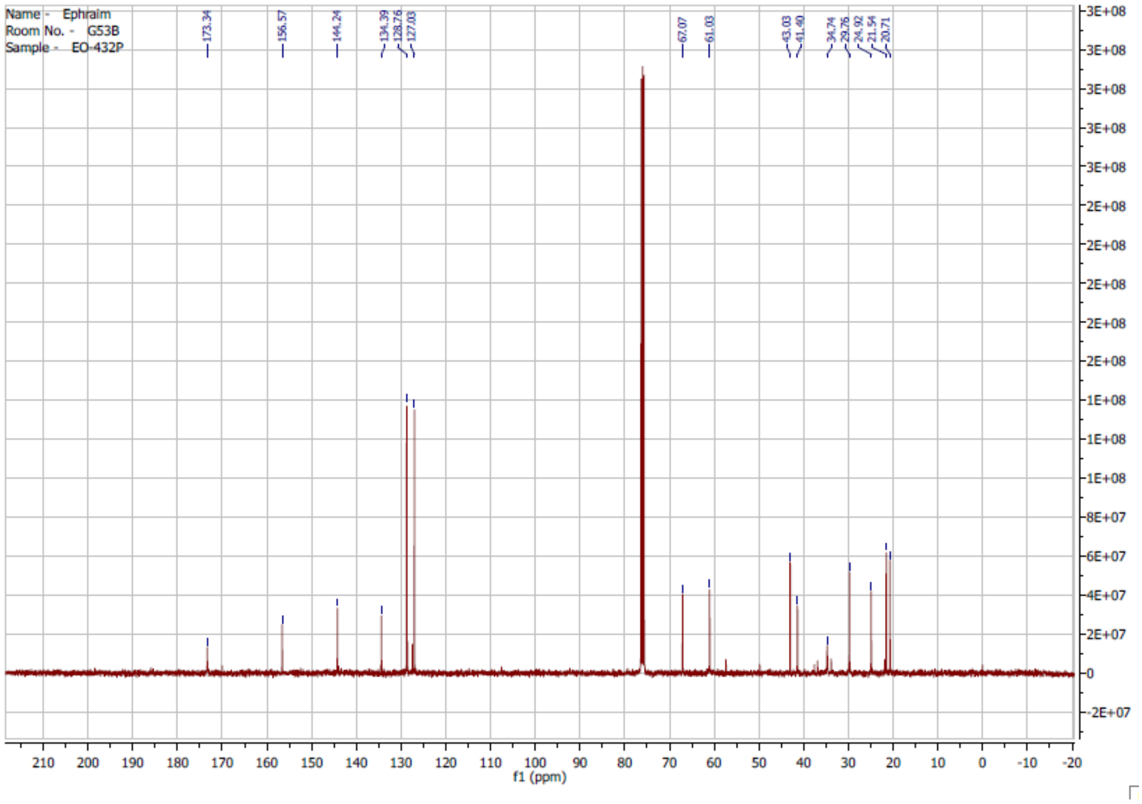

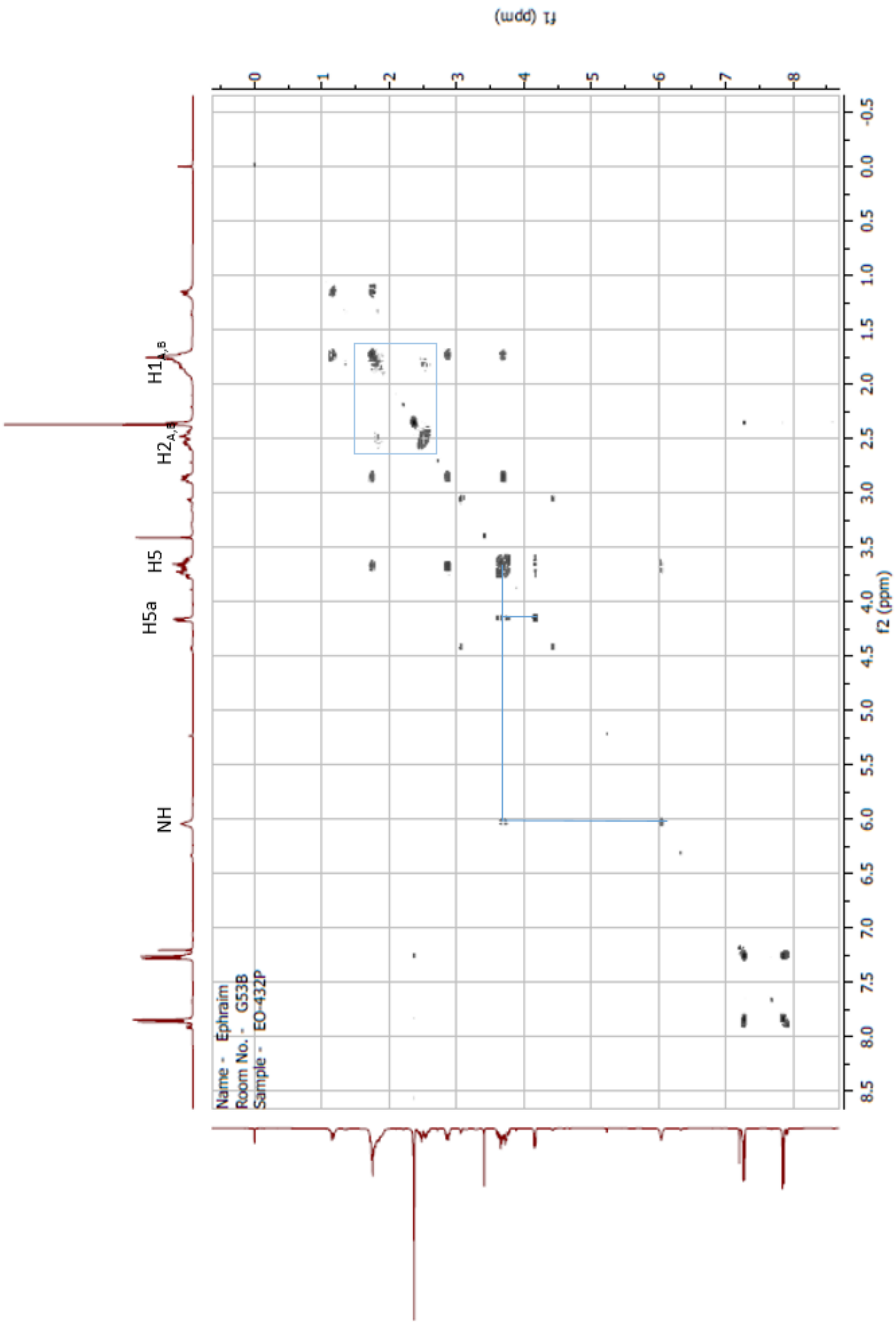
f1 (ppm)
f2 (ppm)
H1A,B
H2A,B
H5
H5a
NH
Name - Ephraim
Room No. - G53B
Sample - EO-432P

(2a*R,5a*R**,6a*S**,6b*S**)-2-Isopropyloctahydro-1*H*,7*H*-cyclopropa[*c*]pyrrolo[1',2':3,4]imidazo[4,5-*e*]azepine-1,5(5a*H*)-dione 18a**

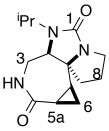

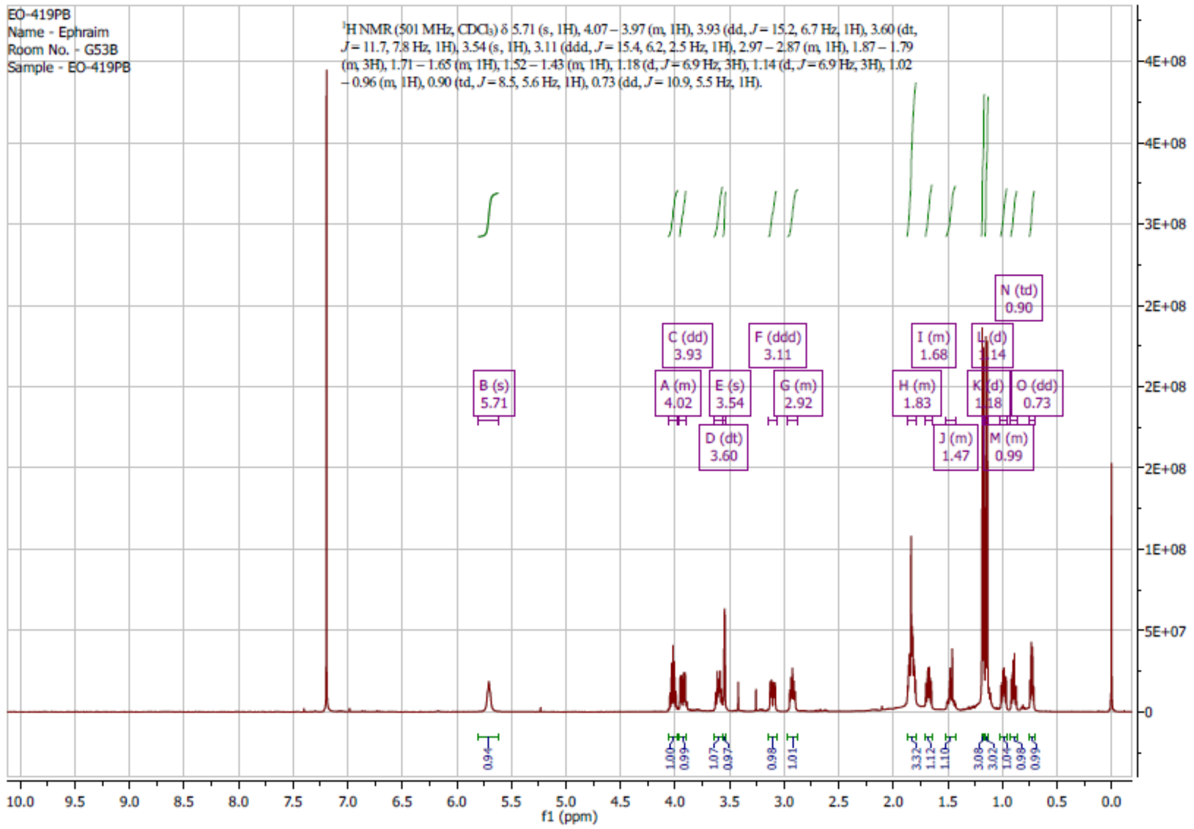

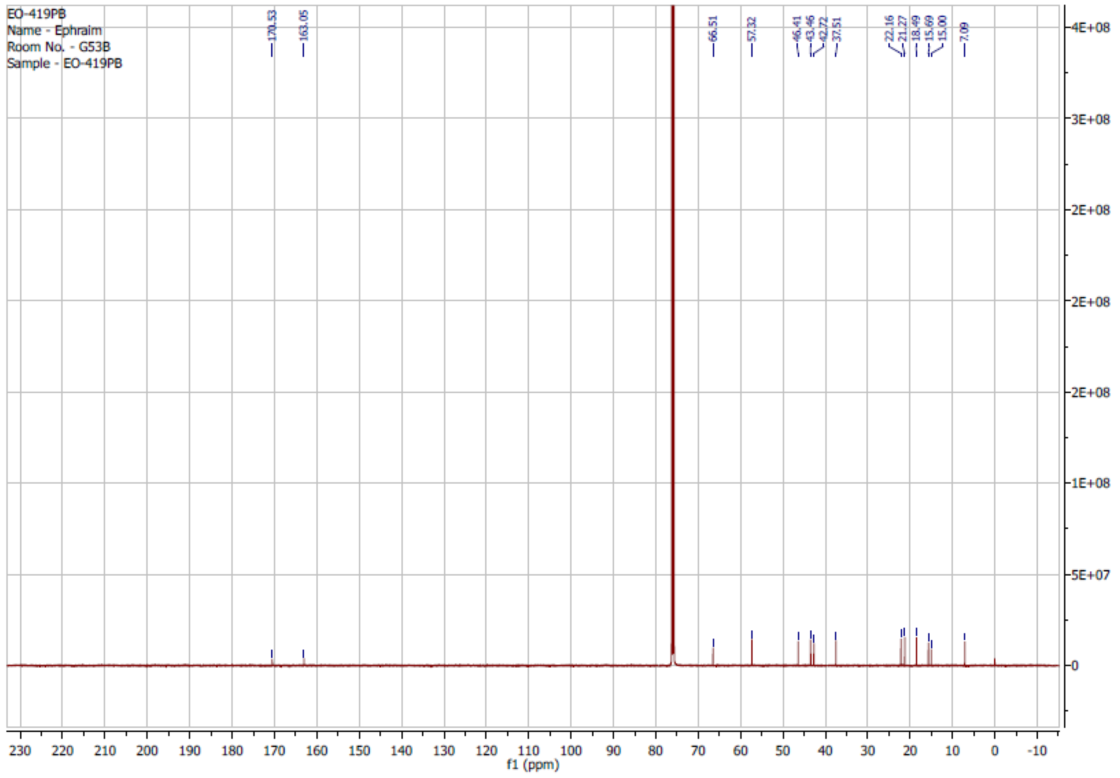

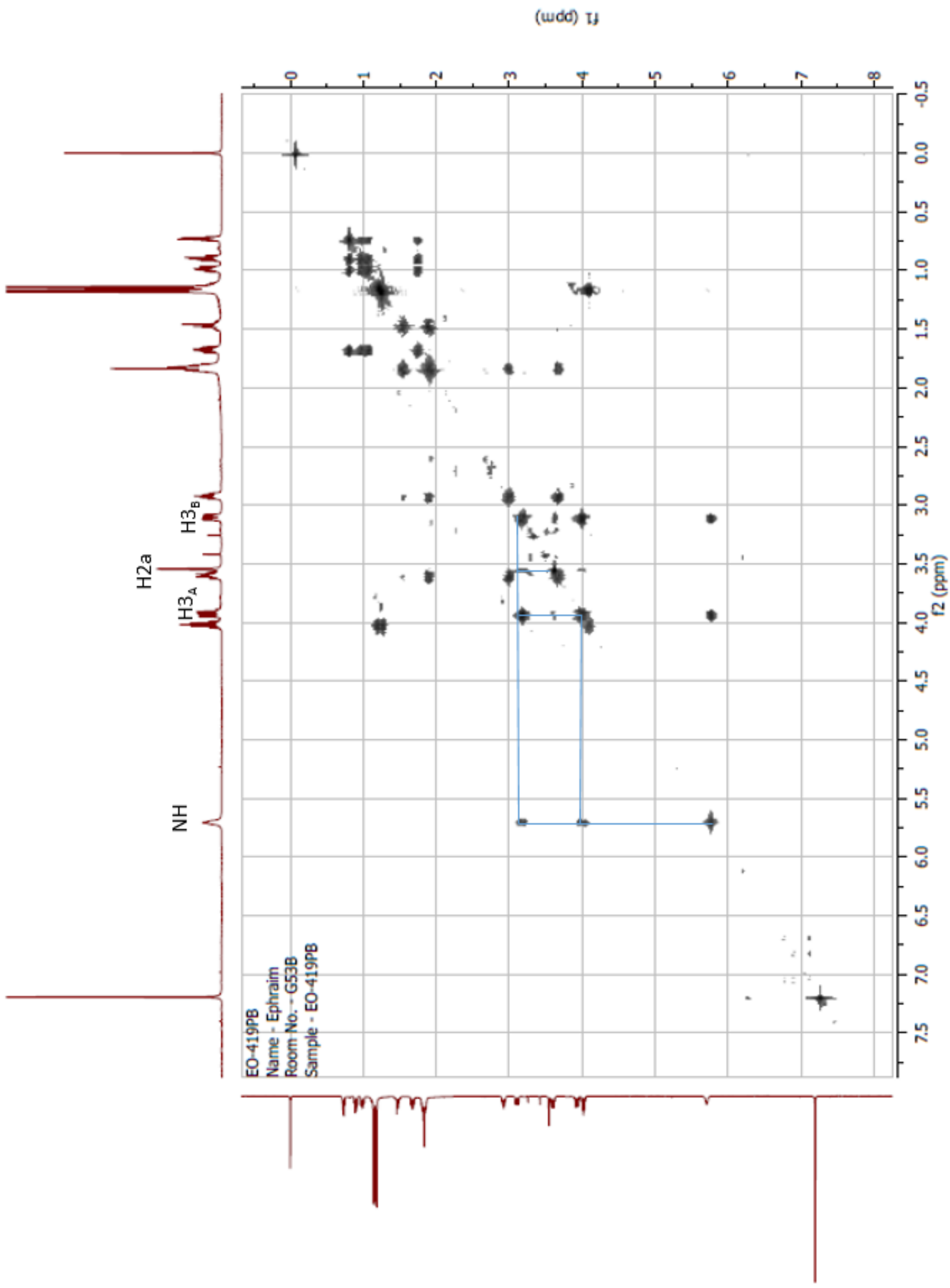
H2a
H3A
H3B
NH
f1 (ppm)
f2 (ppm)
EO-419PB
Name - Ephraim
Room No. - G53B
Sample - EO-419PB

(2a*R,5a*R**,6a*S**,6b*S**)-2-Isopropyloctahydro-1*H*,7*H*-cyclopropa[*b*]pyrrolo[1',2':3,4]imidazo[4,5-*d*]azepine-1,4(2*H*)-dione 18a'**

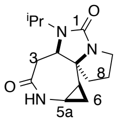

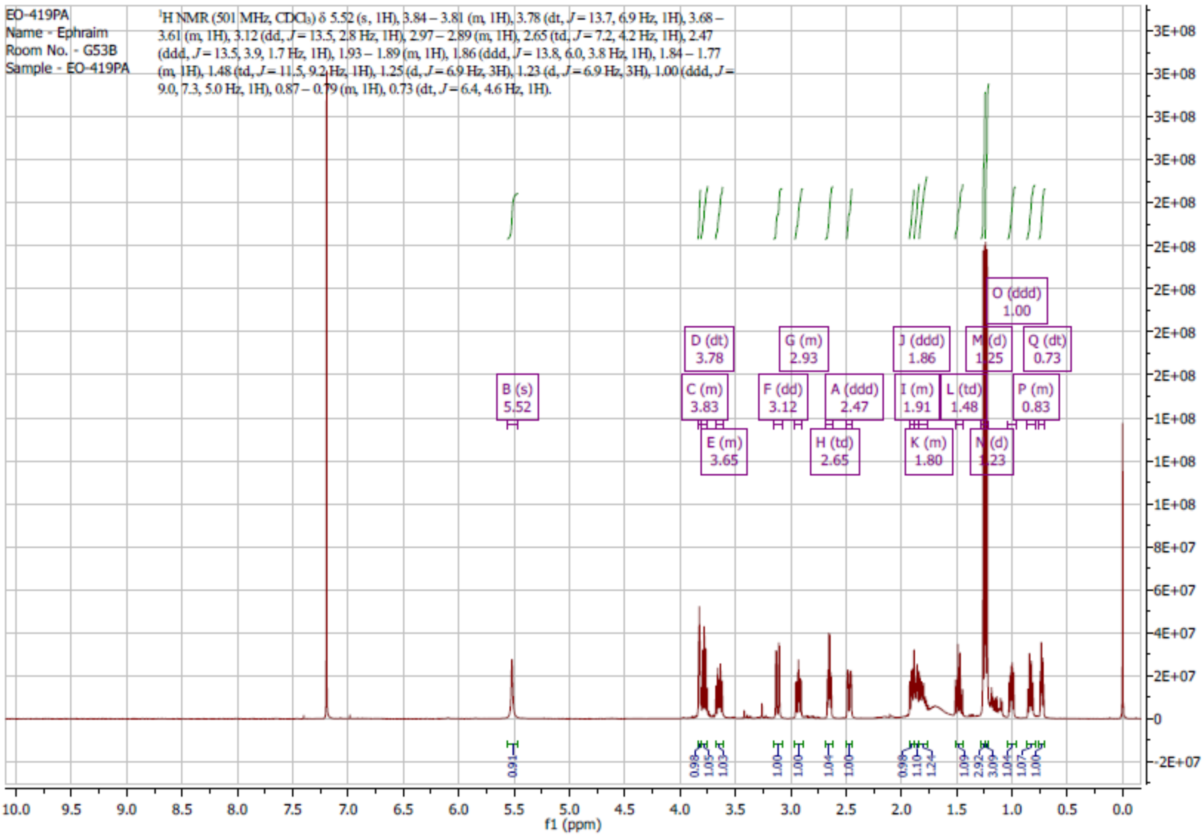

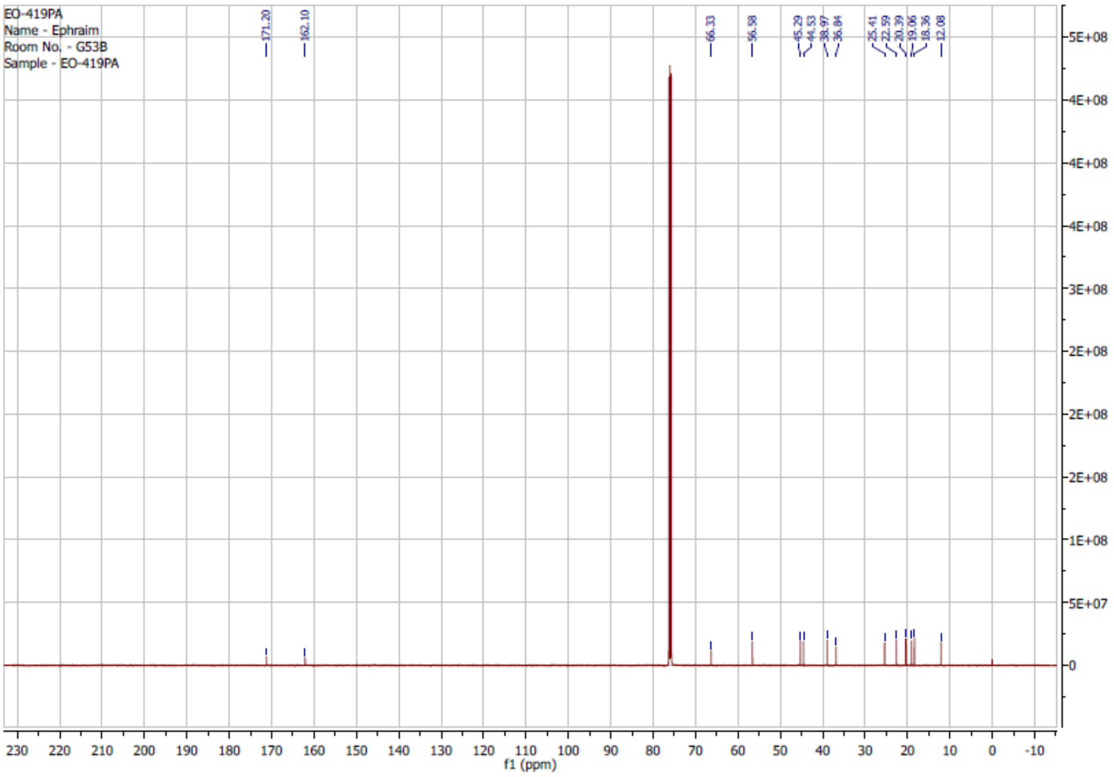

(2a*R,5a*R**,6a*S**,6b*S**)- and (2a*R**,5a*R**,6a*S**,6b*S**)-2-(4-Methoxyphenyl)octahydro-1*H*,*7H*-cyclopropa[*c*]pyrrolo[1',2':3,4]imidazo[4,5-*e*]azepine-1,5(5a*H*)-dione 18b/18b'**

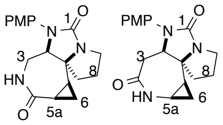

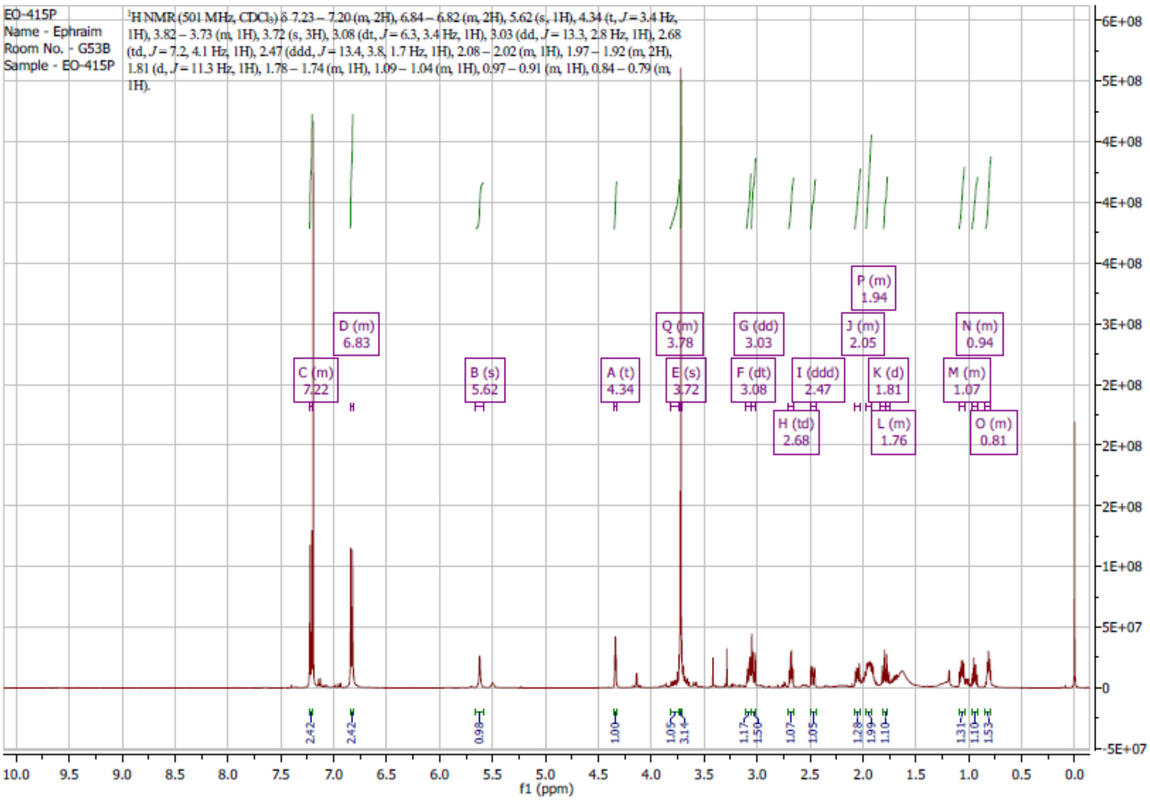

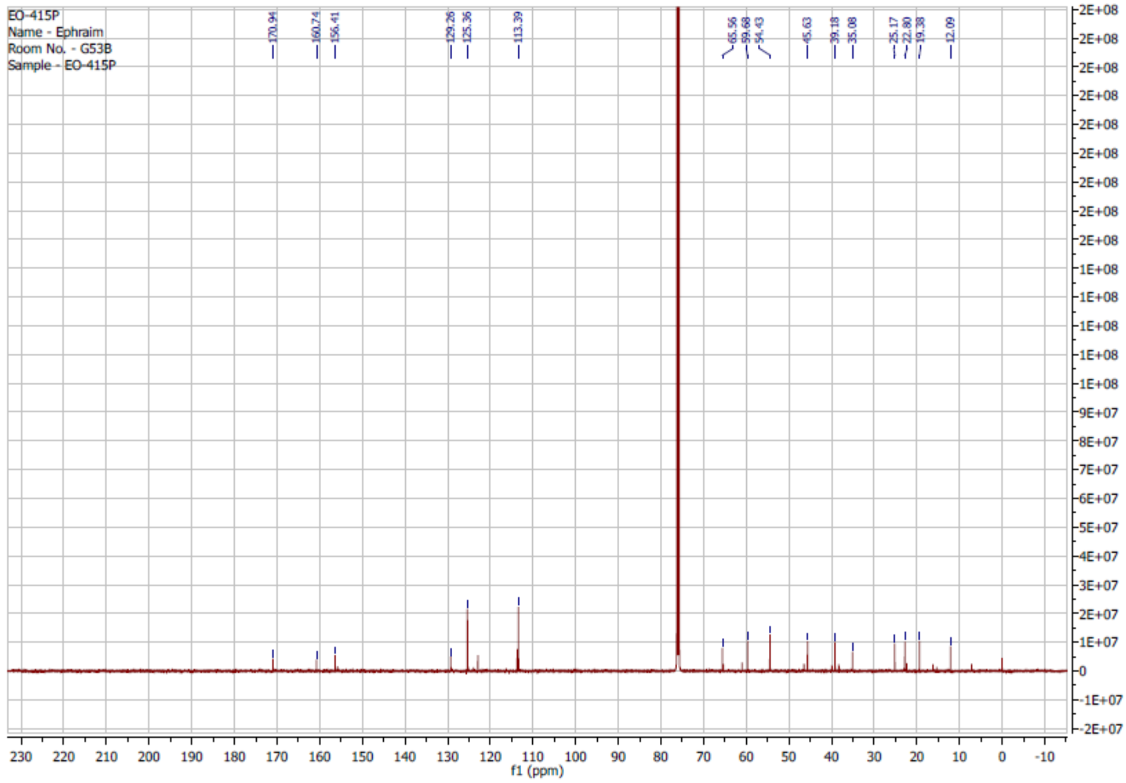

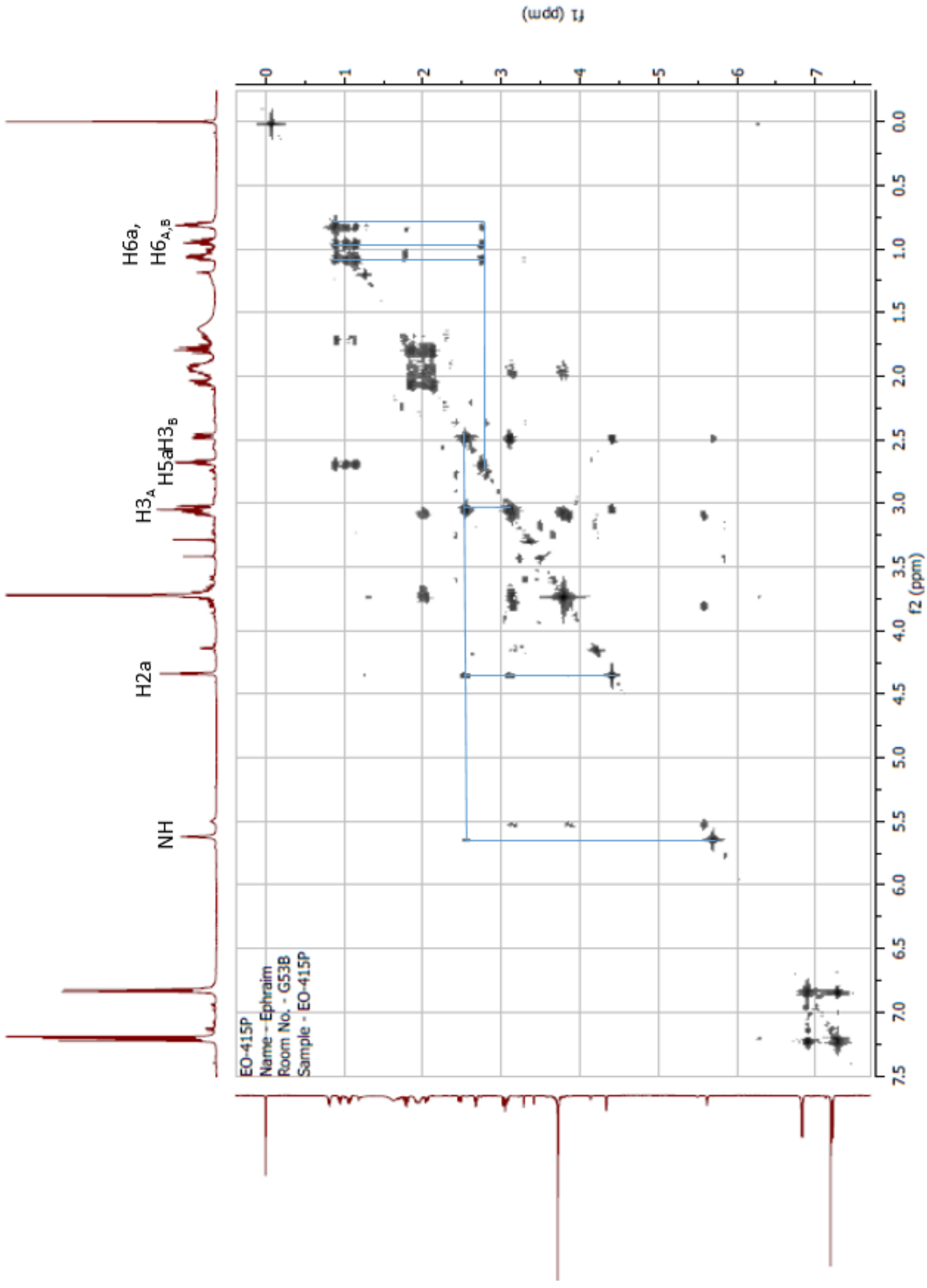
H6a,
H6A,B
H5aH3B
H3A
H2a
NH
f1 (ppm)
f2 (ppm)
EO-415P
Name - Ephraim
Room No. - G53B
Sample - EO-415P

(6a*R,8*R**,10a*S**)-8-(Oxetan-3-ylamino)-6-(4-toluenesulfonyl)octahydro-1*H*,5*H*-benzo[*d*]pyrrolo[1,2-*c*]imidazol-5-one 19**

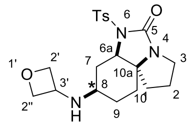

Major diastereomer, *dr* 85:15

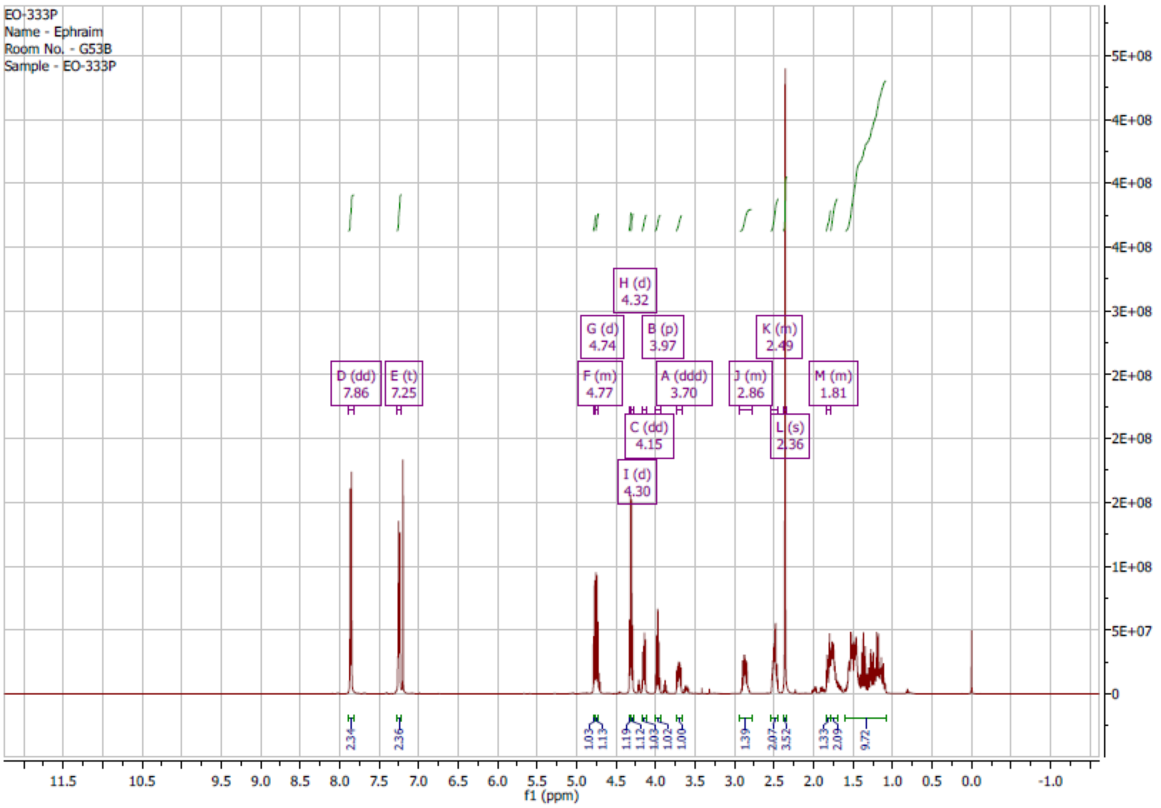

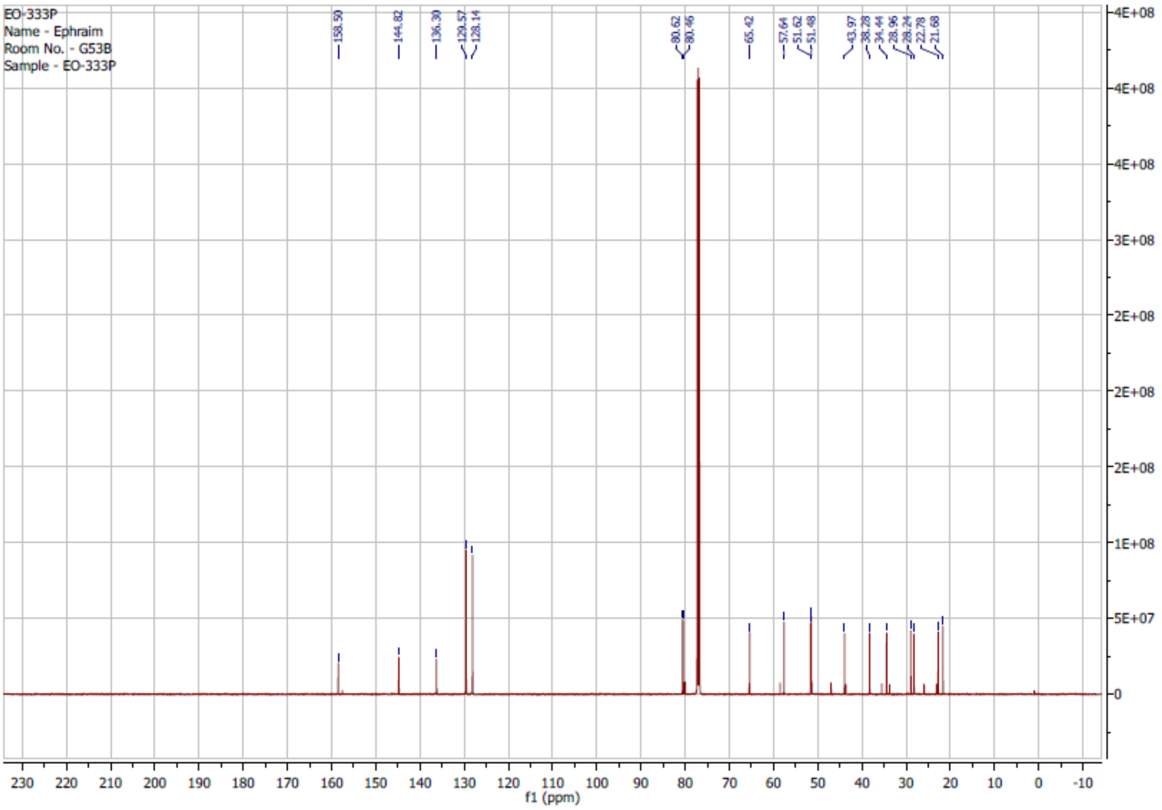

(6a*R**,8*R**,10a*S**)-6-Isopropyl-5-oxooctahydro-1*H*,5*H*-benzo[*d*]pyrrolo[1,2-*c*]imidazol-8-yl (3-fluorophenyl)carbamate 20

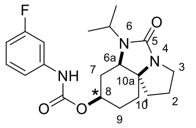

Major diastereomer, *dr* 93:7

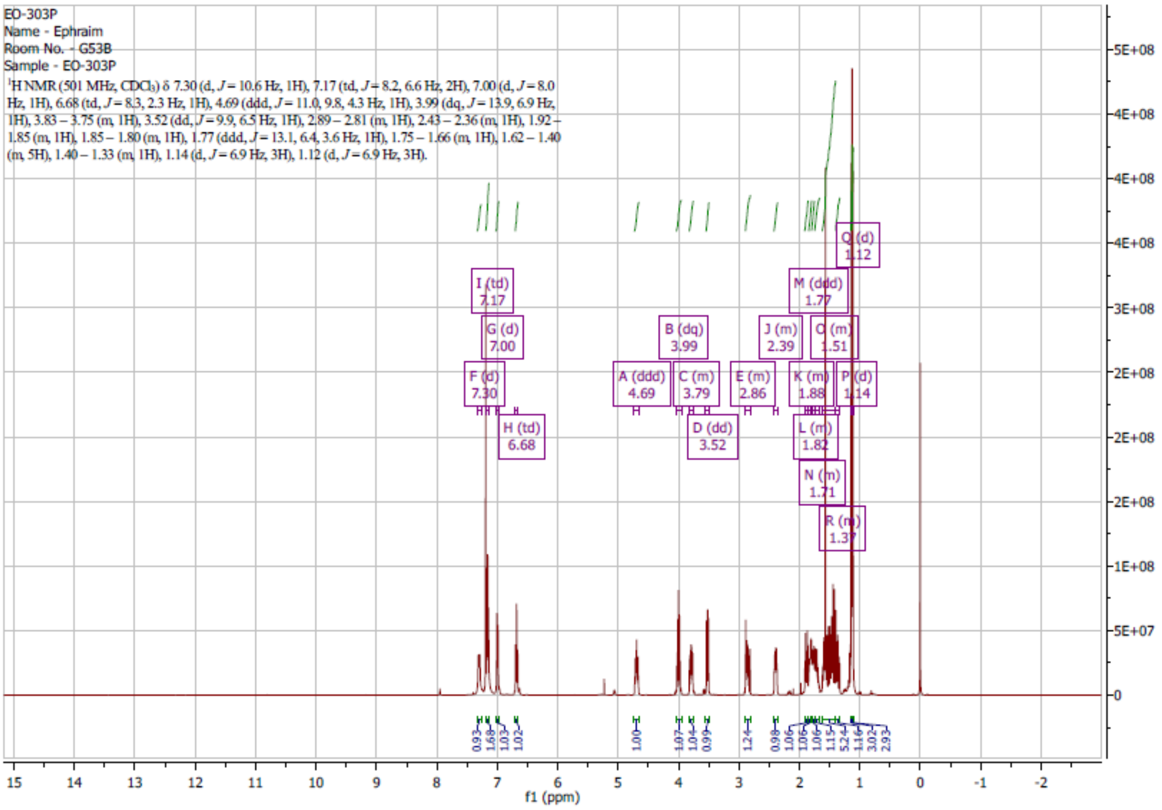

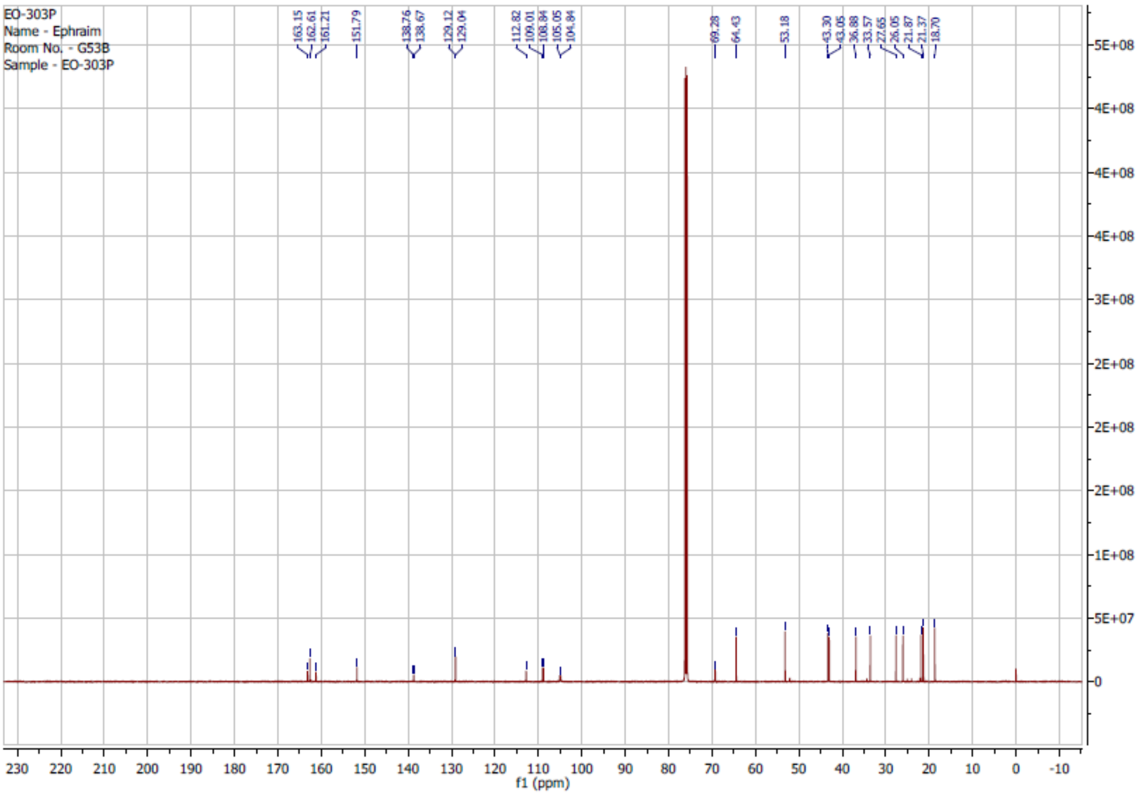

(6a*R,8*R**,10a*S**)-6-Isopropyl-8-(pyridine-2-yloxy)octahydro-1*H*,5*H*-benzo[*d*]pyrrolo[1,2-*c*]imidazol-5-one 21**

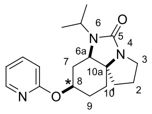

Major diastereomer, *dr* 93:7

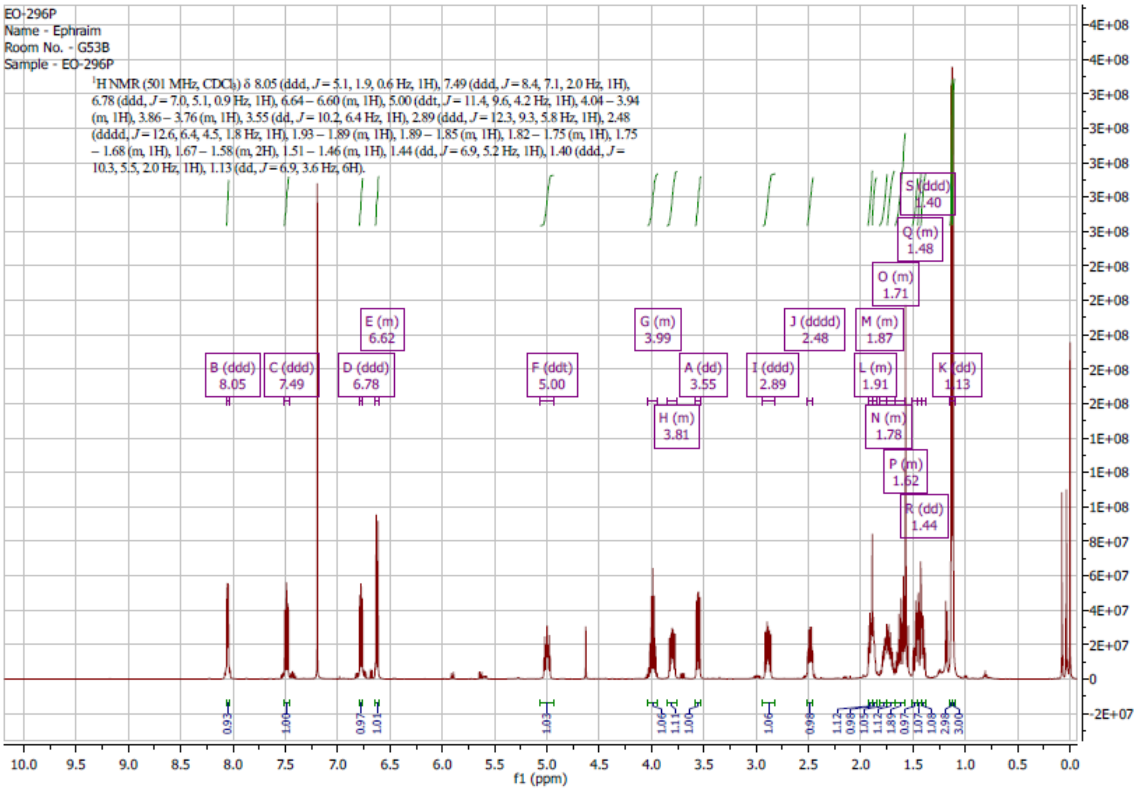

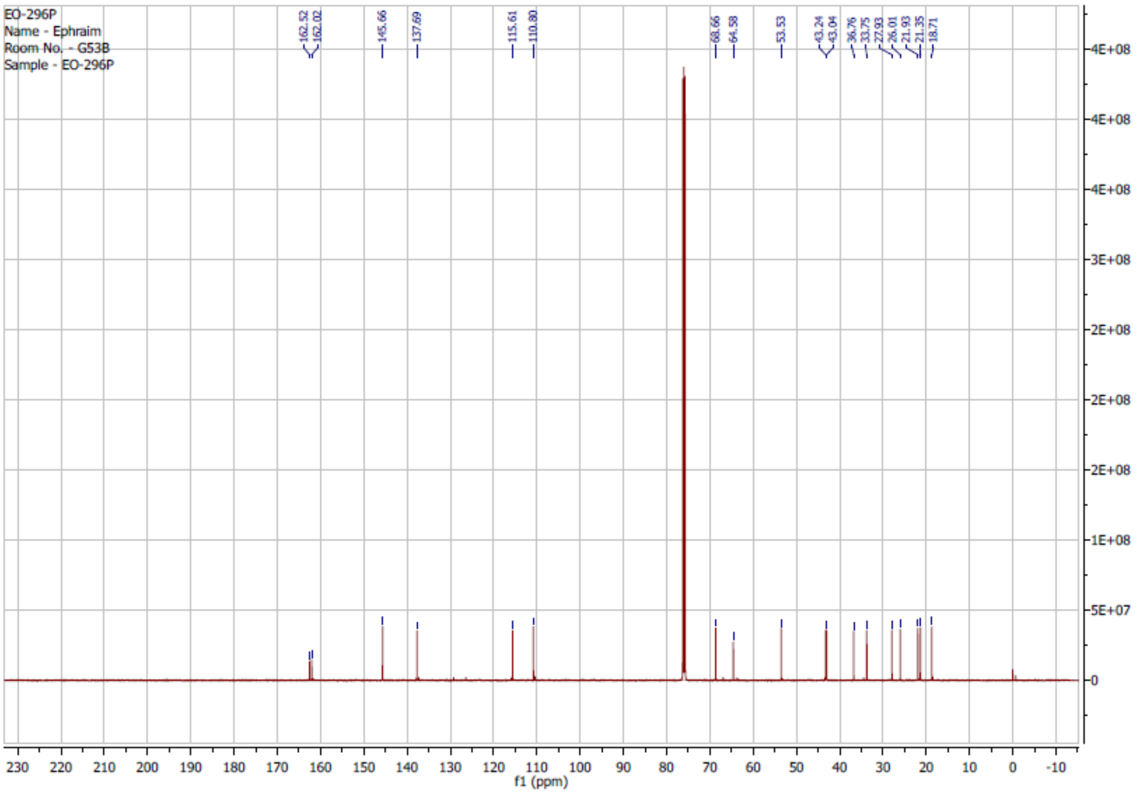

(6a*R,8*S**,8a*R**,9a*S**, 9b*S**)-8-Hydroxy-6-isopropyl-8-(thiophen-2-yl)octahydro-1*H*-cyclopropa[5,6]benzo[1,2-*d*]pyrrolo[1,2-*d*]imidazol-5(6*H*)-one 22**

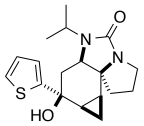

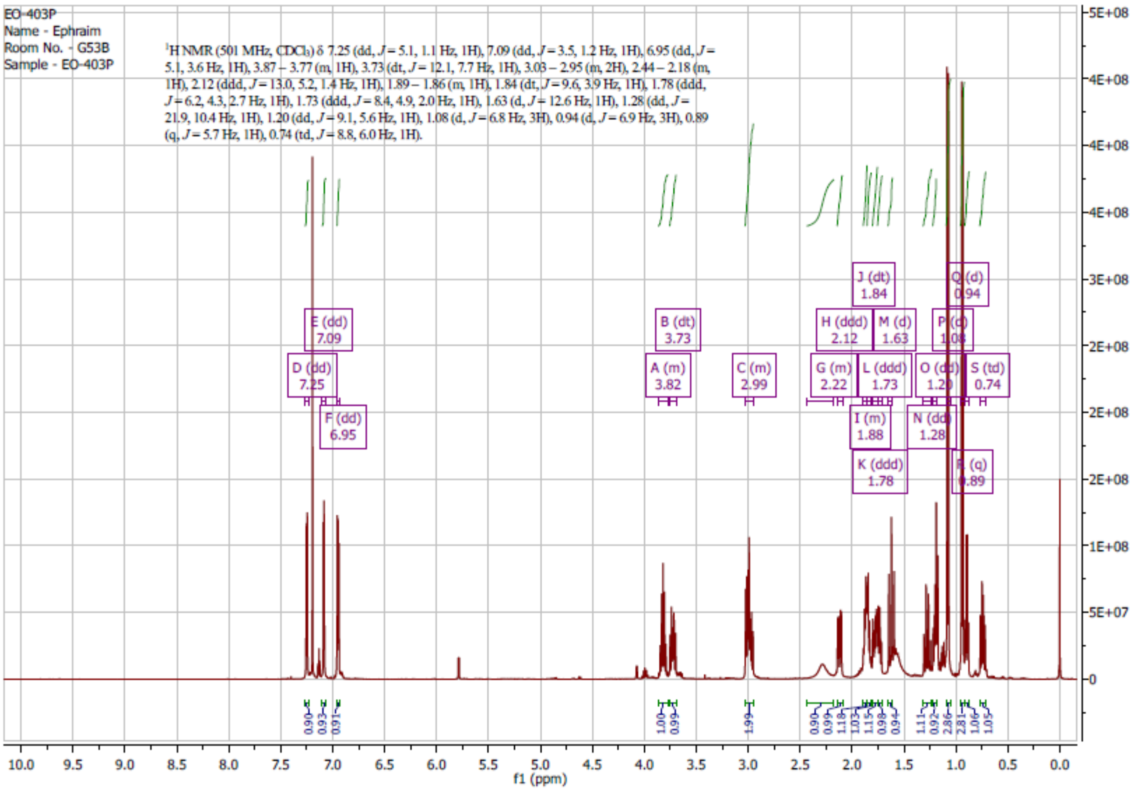

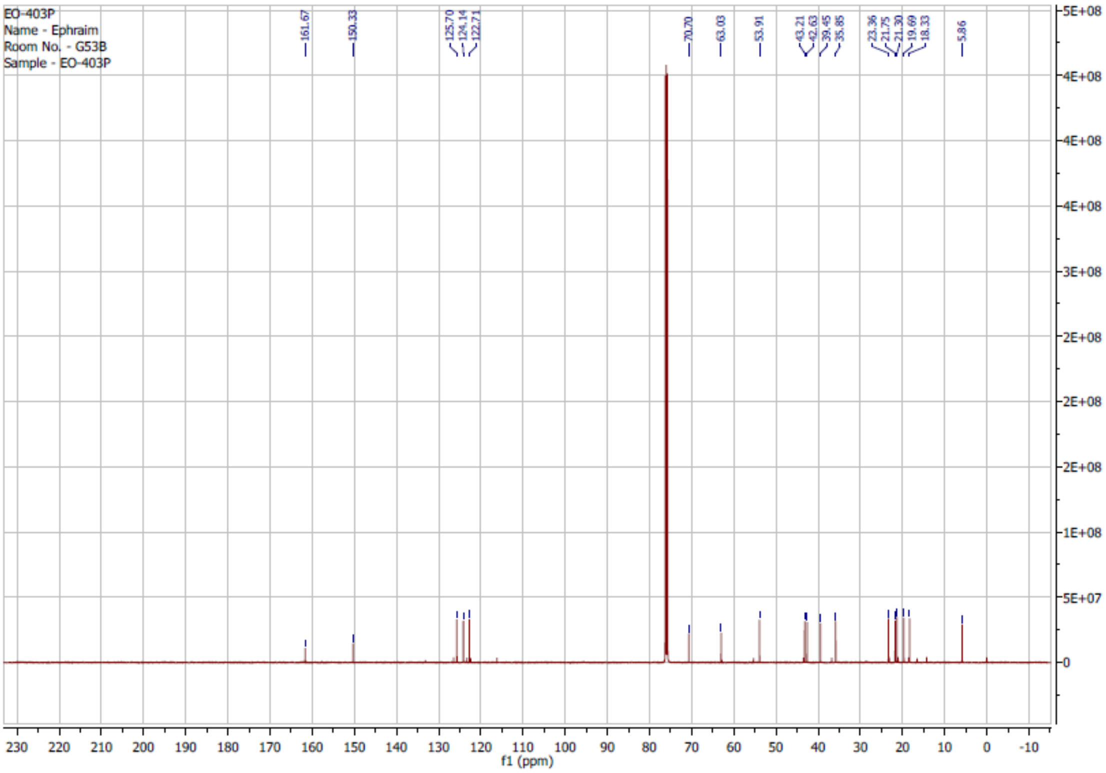

(6a*R,8*S**,8a*S**,11a*R**,11b*S**)-10-Benzyl-8-hydroxy-6-(4-methoxyphenyl)decahydro-1*H*-pyrrolo[1',2':3,4]imidazo[4,5-*e*]isoindol-5(6*H*)-one 23**

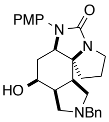

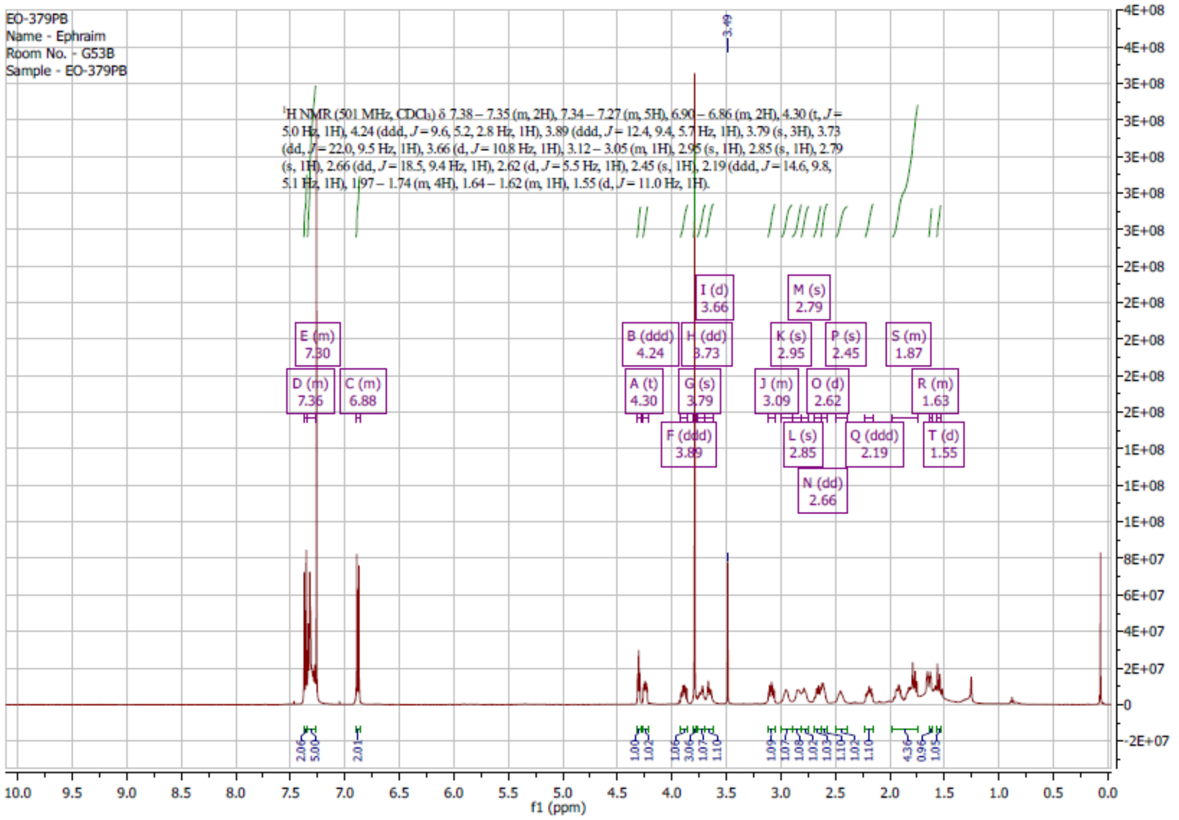

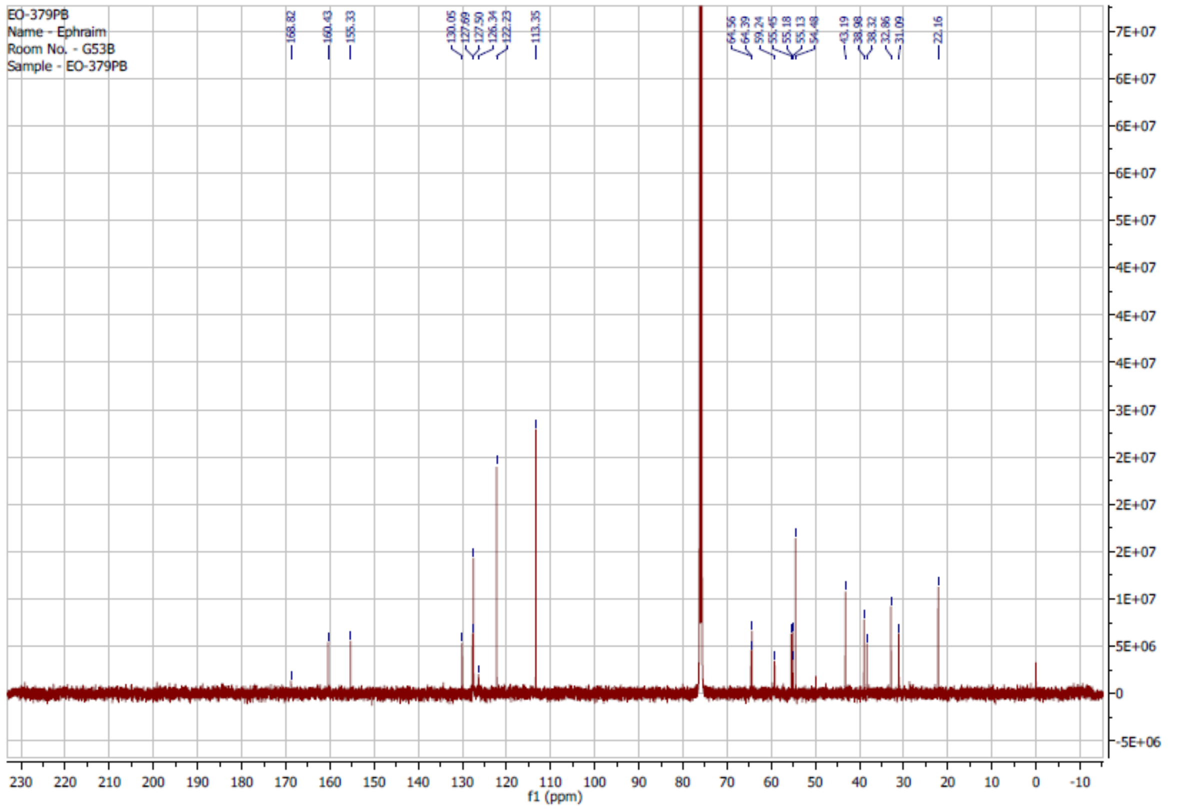

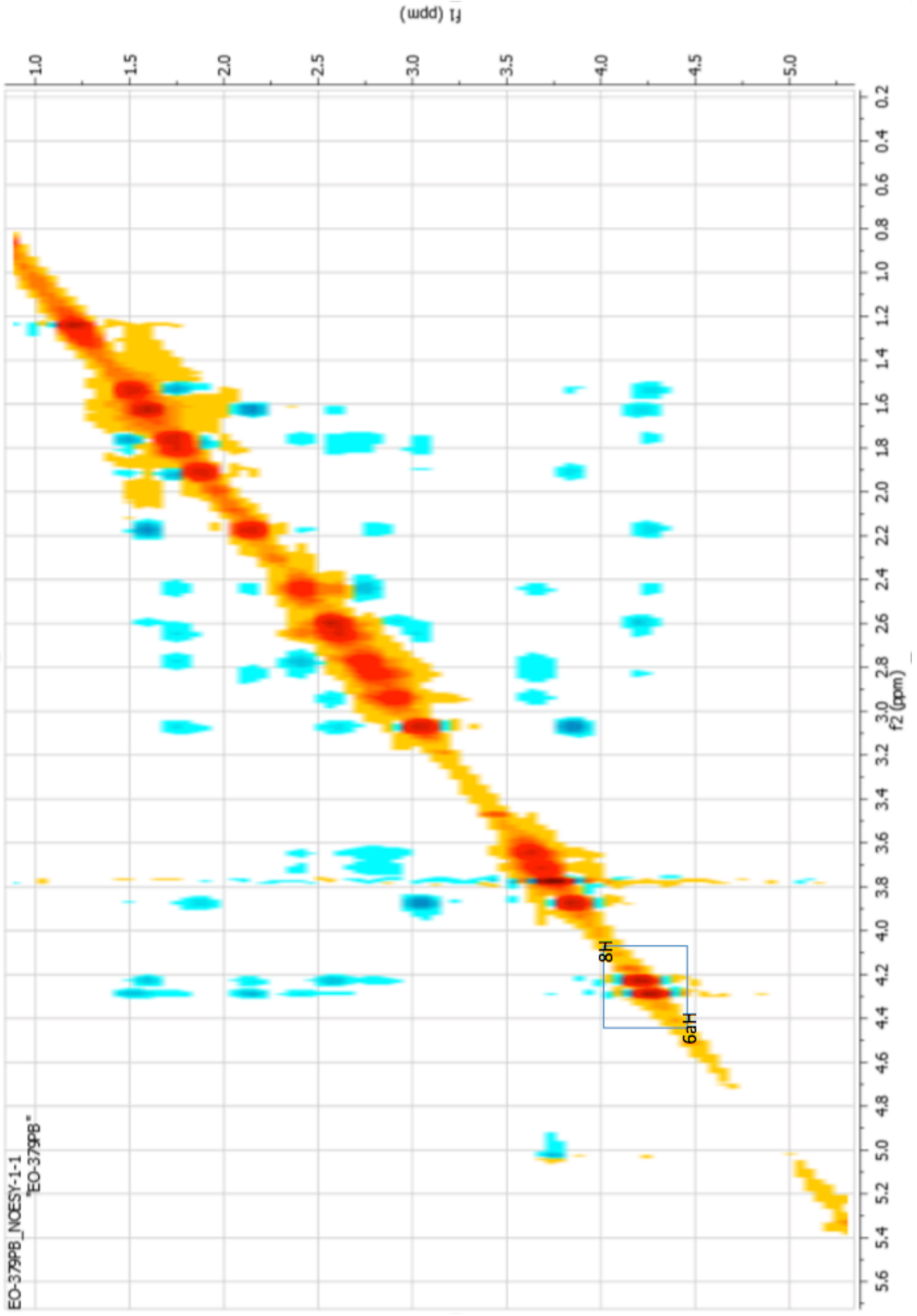
EO-379PB_NOESY-1-1
"EO-379PB"
8H
6aH
f1 (ppm)
f2 (ppm)

4-Methyl-*N*-[(5*S**,6*R**)-1-methyl-8-oxo-1-azaspiro[4.5]decan-6-yl)benzenesulfonamide S7

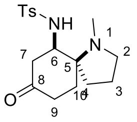

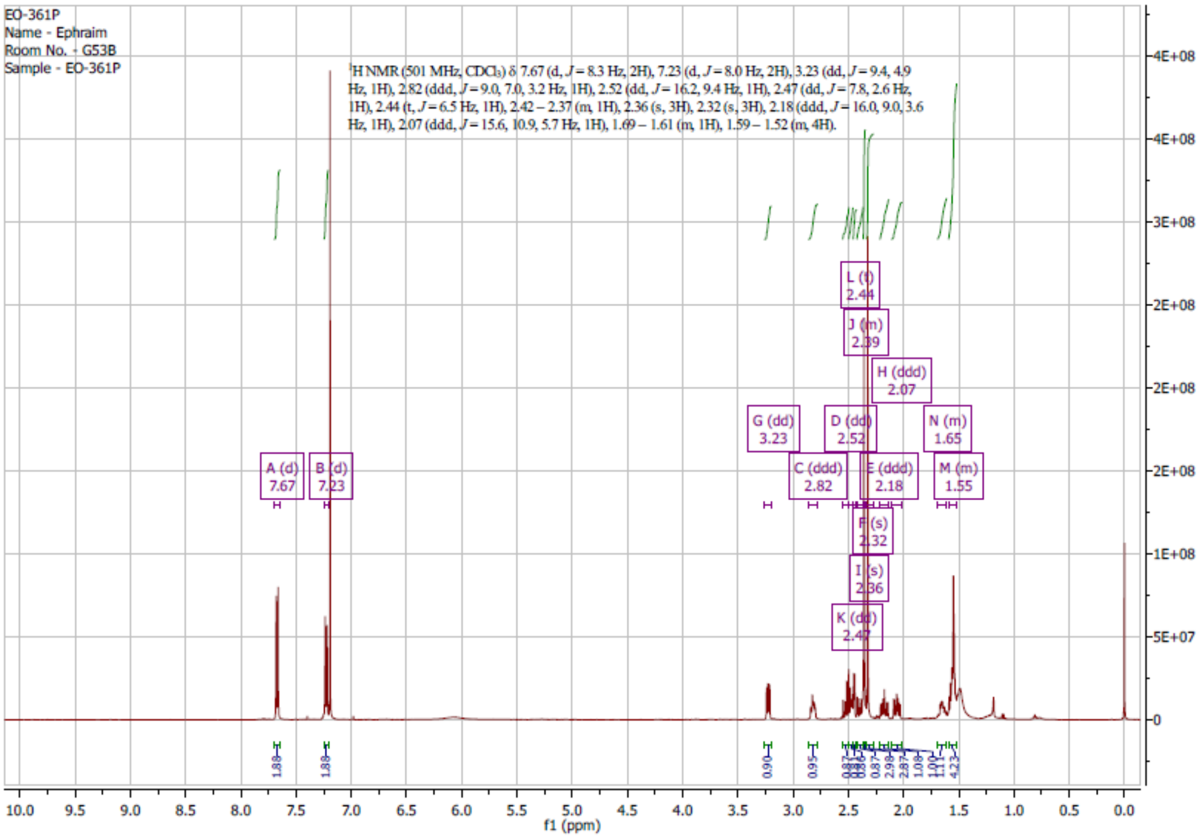

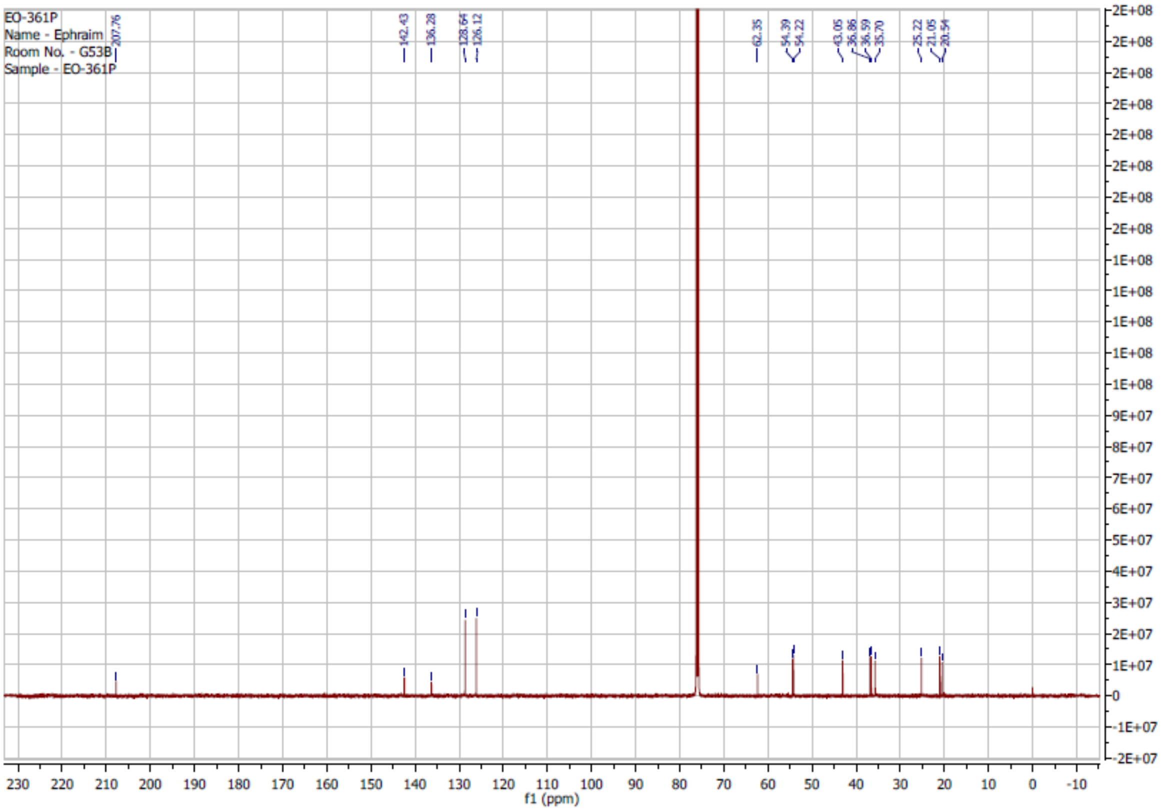

4-Methyl-*N*-[(5*S,6*R**,8*R**)-1-methyl-8-(oxoetan-3-ylamino)-1-azaspiro[4.5]decan-6-yl)benzenesulfonamide 24**

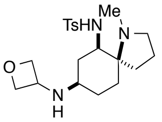

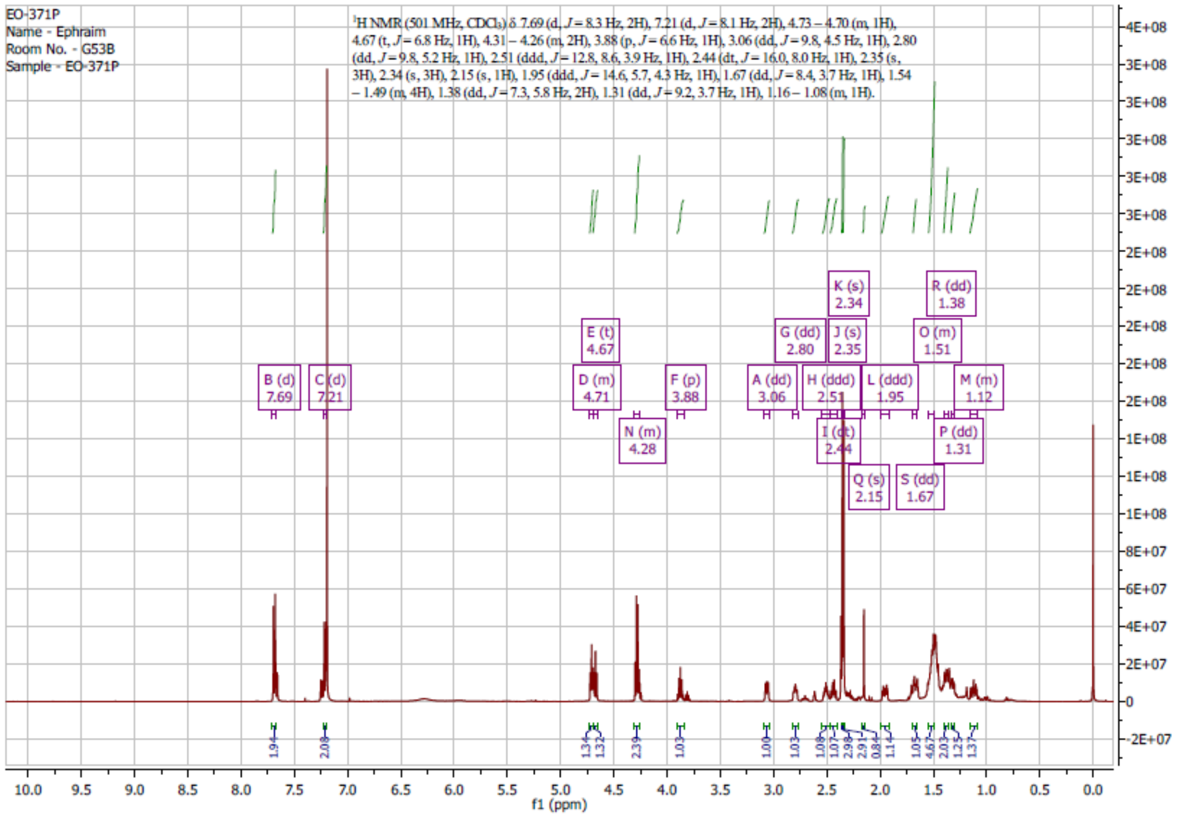

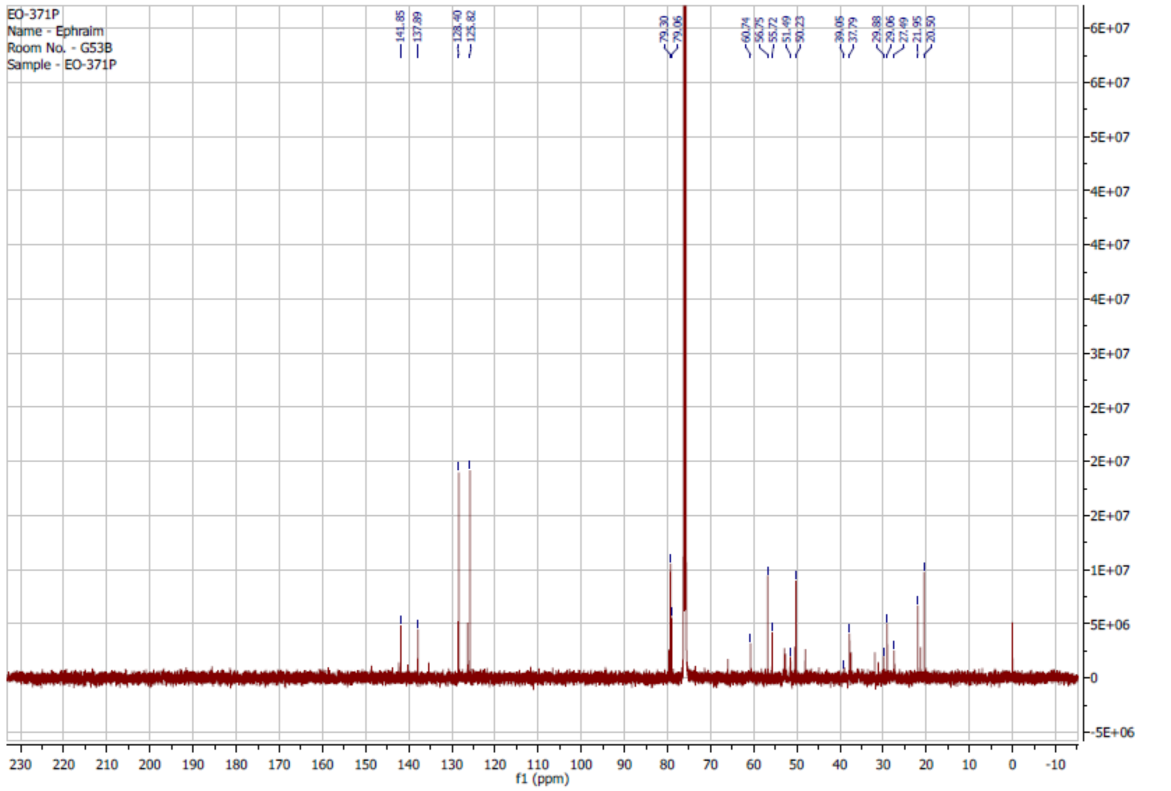

4-Methyl-*N*-[(5*S,6*R**,8*R**)-1-methyl-8-(methylamino)-1-azaspiro[4.5]decan-6-yl)benzenesulfonamide 25**

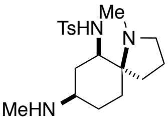

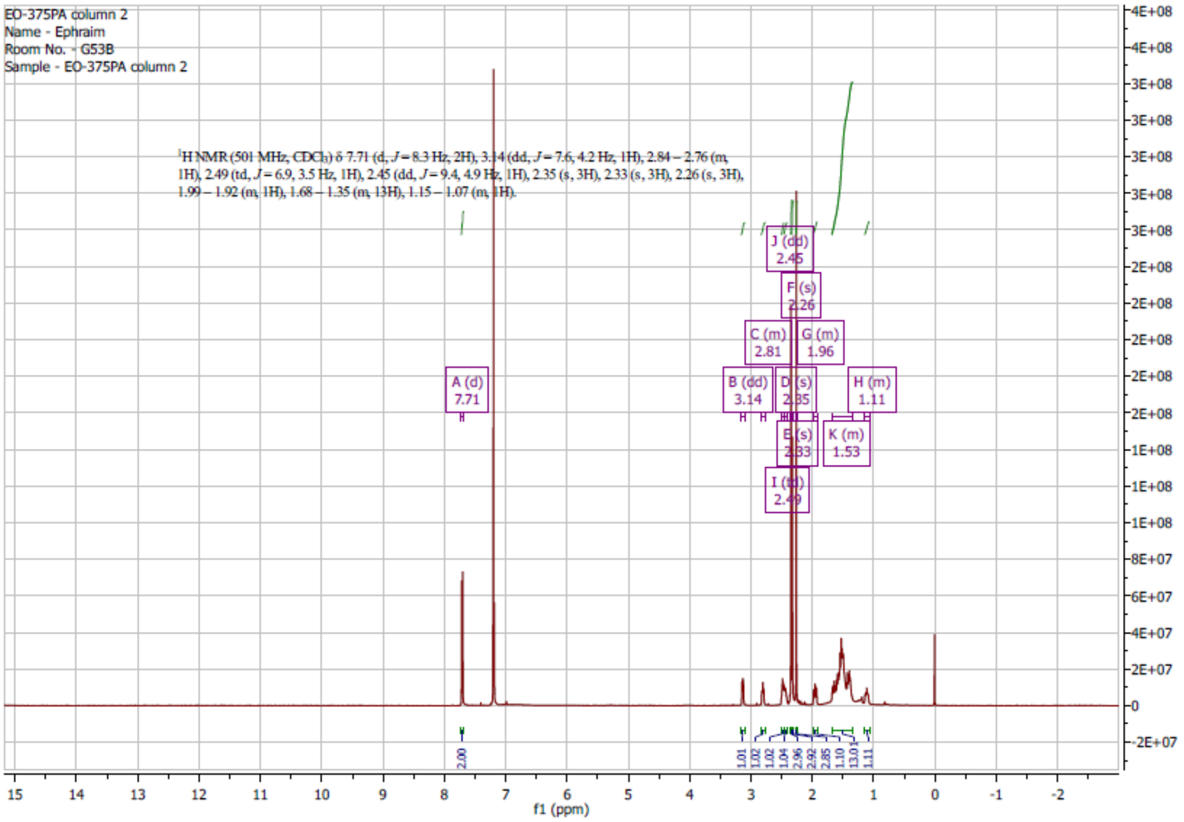

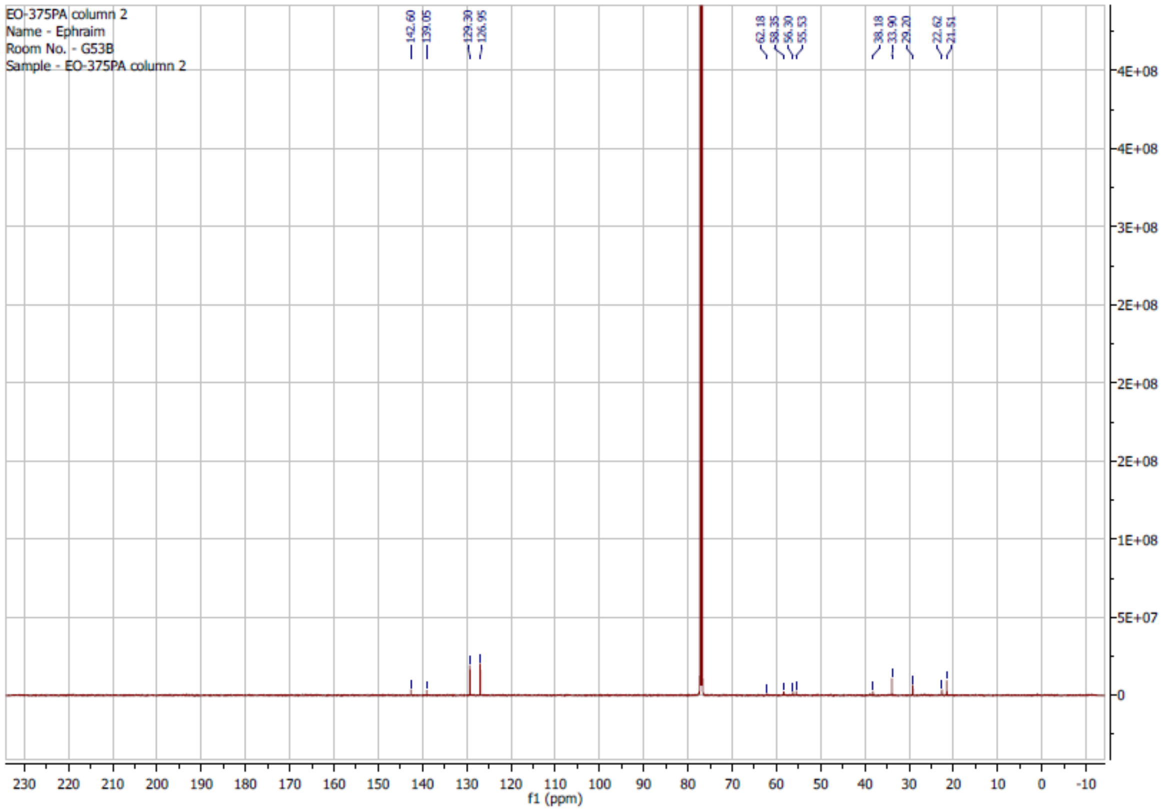

4-Methyl-*N*-[(5*S,6*R**,8*S**)-1-methyl-8-(methylamino)-1-azaspiro[4.5]decan-6-yl)benzenesulfonamide 26**

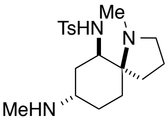

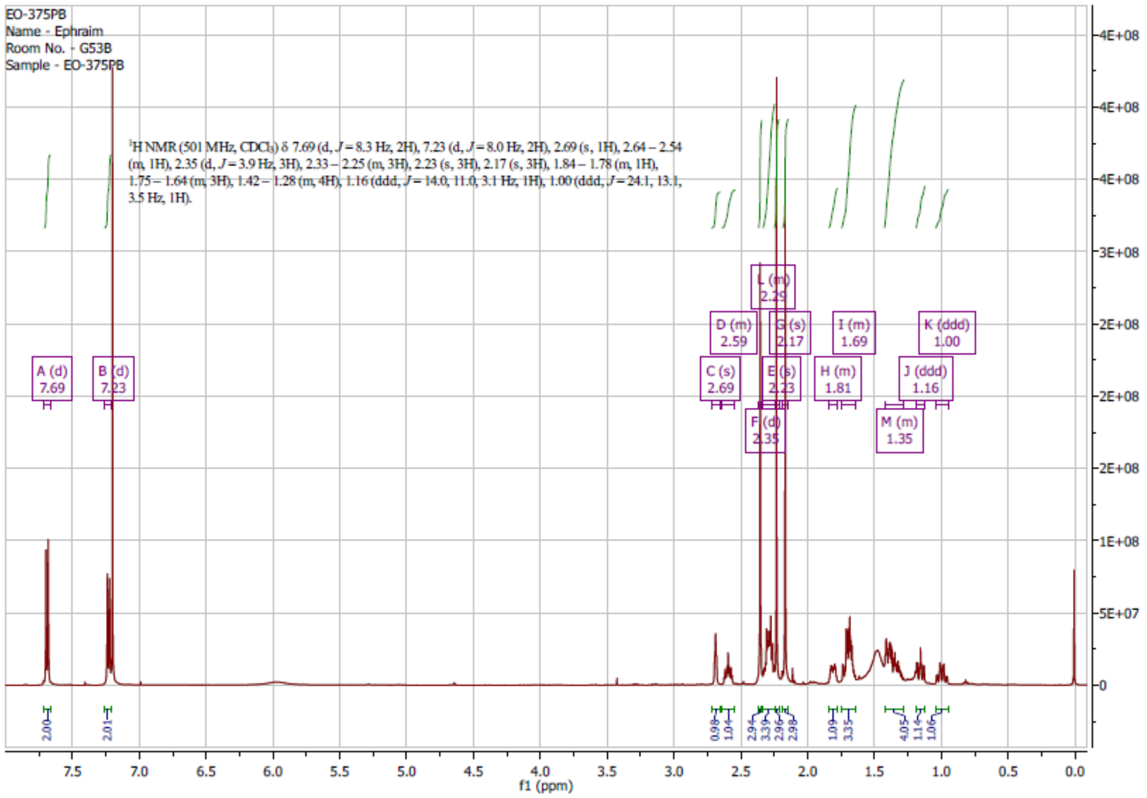

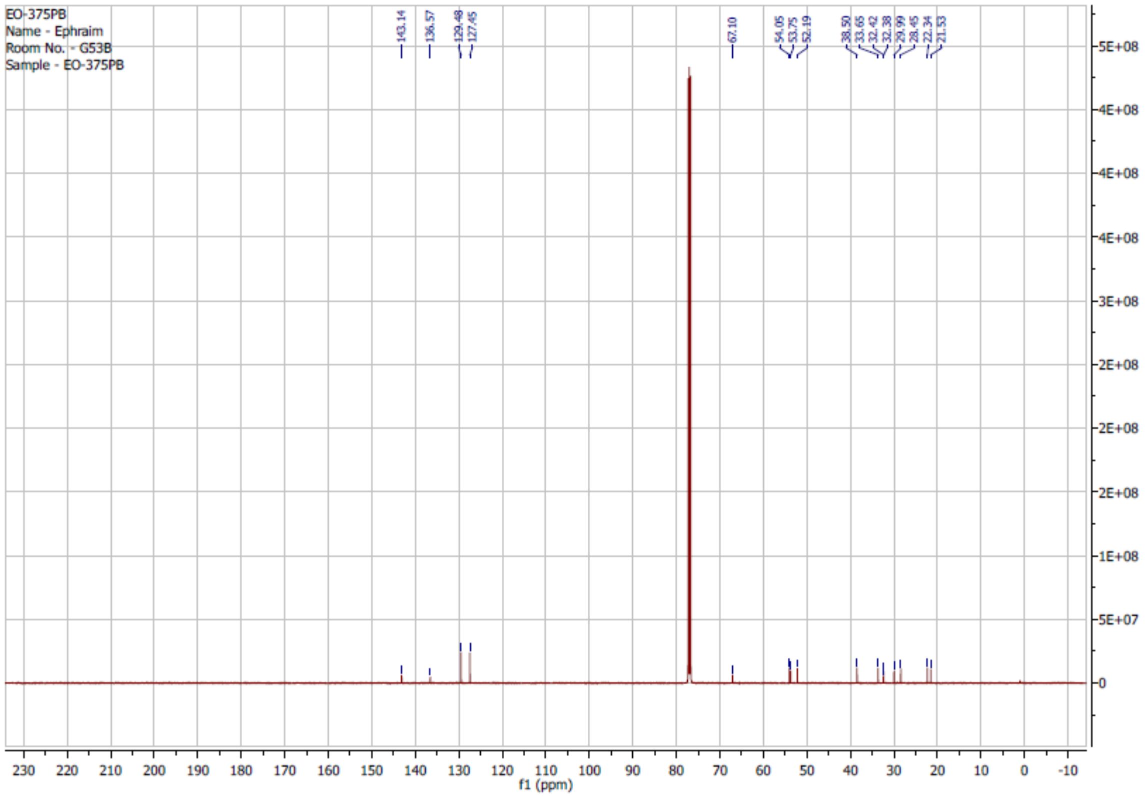

(6a*R,8*R**,10a*S**)-8-(Cyclopropylamino)-6-tosyloctahydro-1*H*,5*H*-benzo[*d*]pyrrolo[1,2-*c*]imidazol-5-one 27**

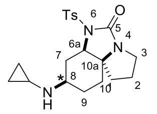

Major diastereomer, *dr* 87:13

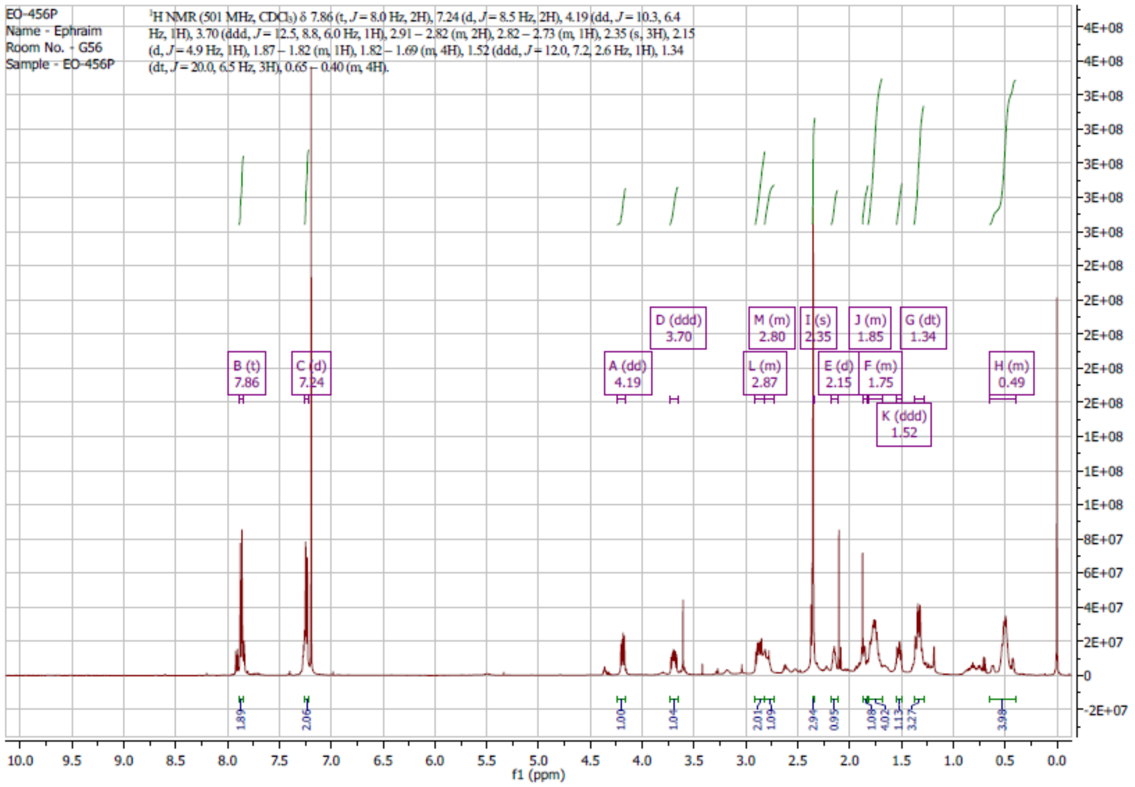

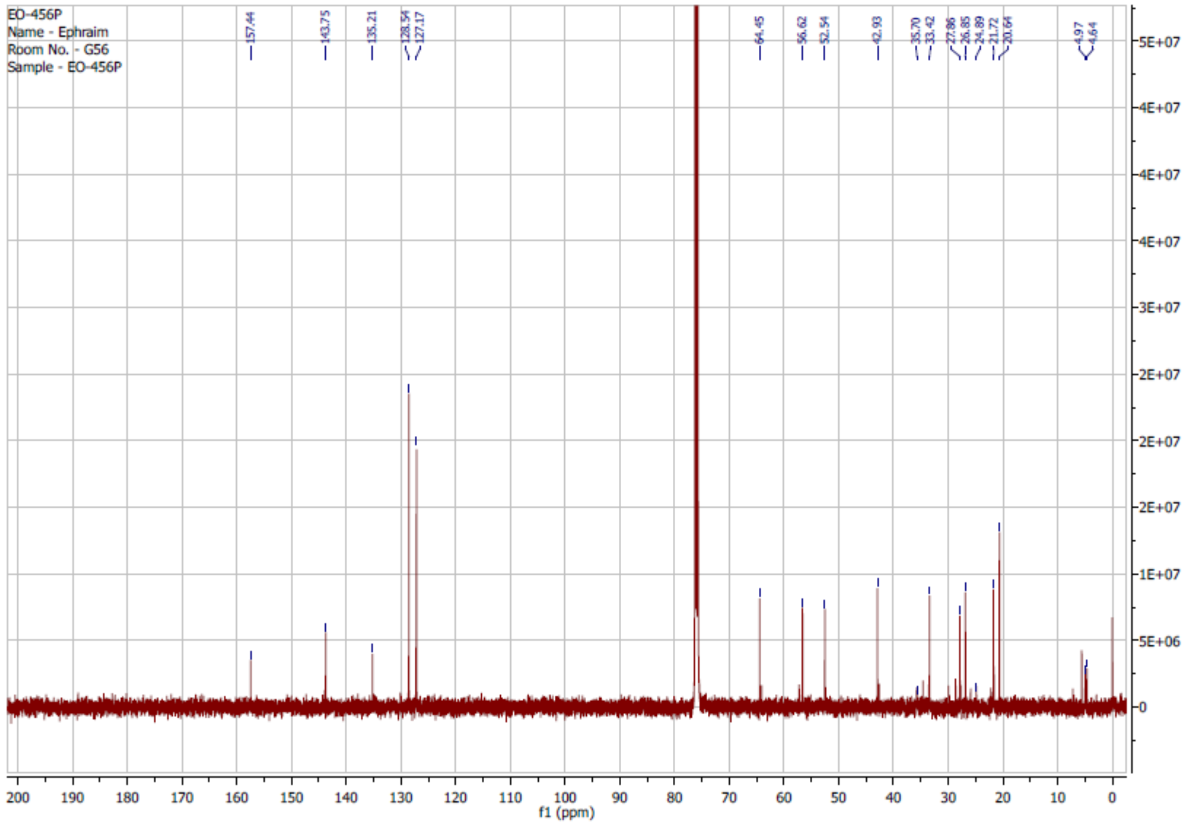

(6a*R,8*S**,8a*S**,11a*R**,11b*S**)-10-Benzyl-8-hydroxy-6-isopropyldecahydro-1*H*-pyrrolo[1’,2’:3,4]imidazo[4,5-*e*]isoindol-5(6*H*)-one 28**

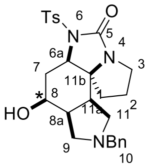

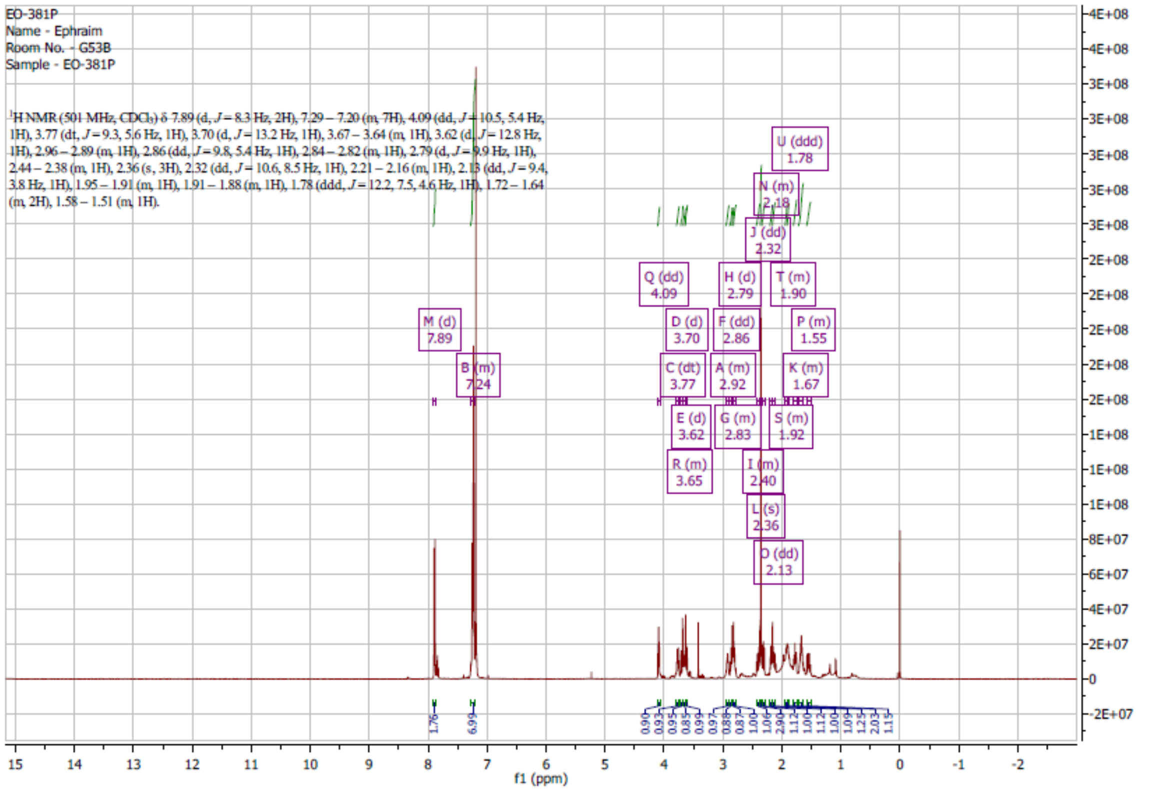

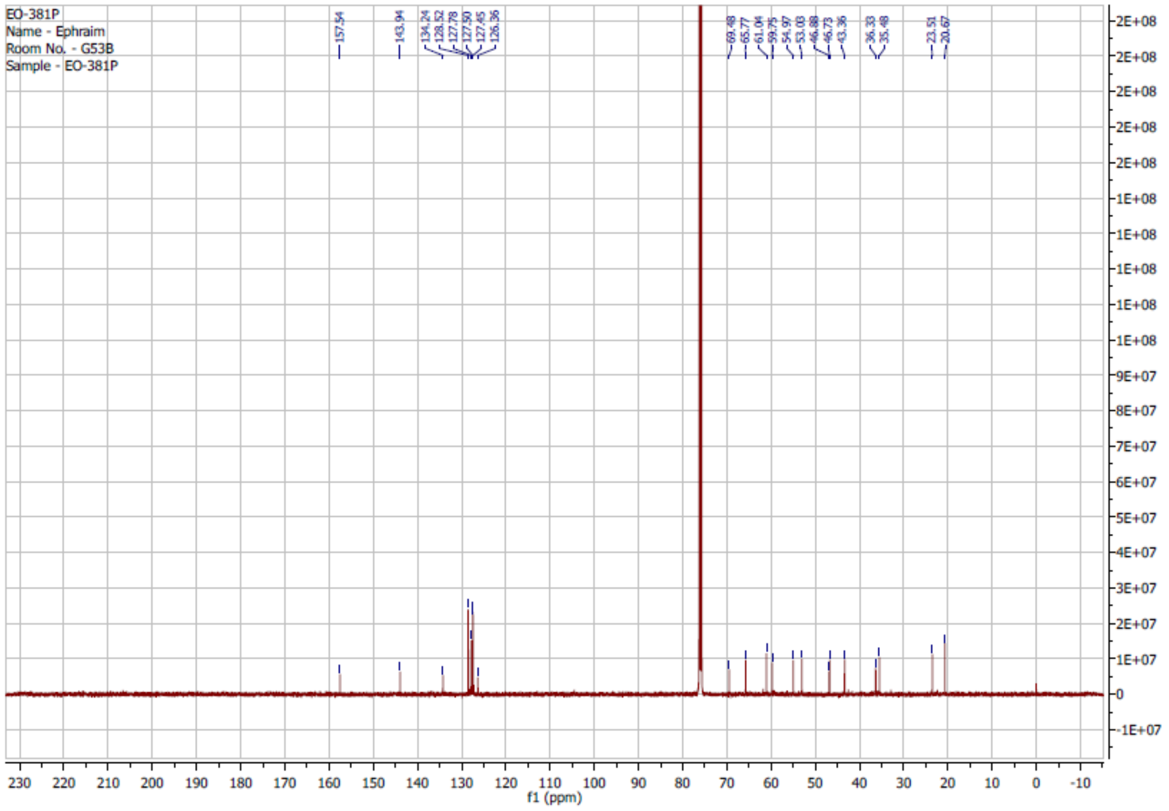

(6a*R,8*S**,8a*R**,9a*S**, 9b*S**)-6-(4-Methoxyphenyl)-8-(methylamino)octahydro-1*H*-cyclopropa[5,6]benzo[1,2-*d*]pyrrolo[1,2-*c*]imidazol-5(6*H*)-one 29**

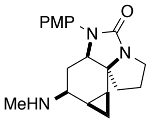

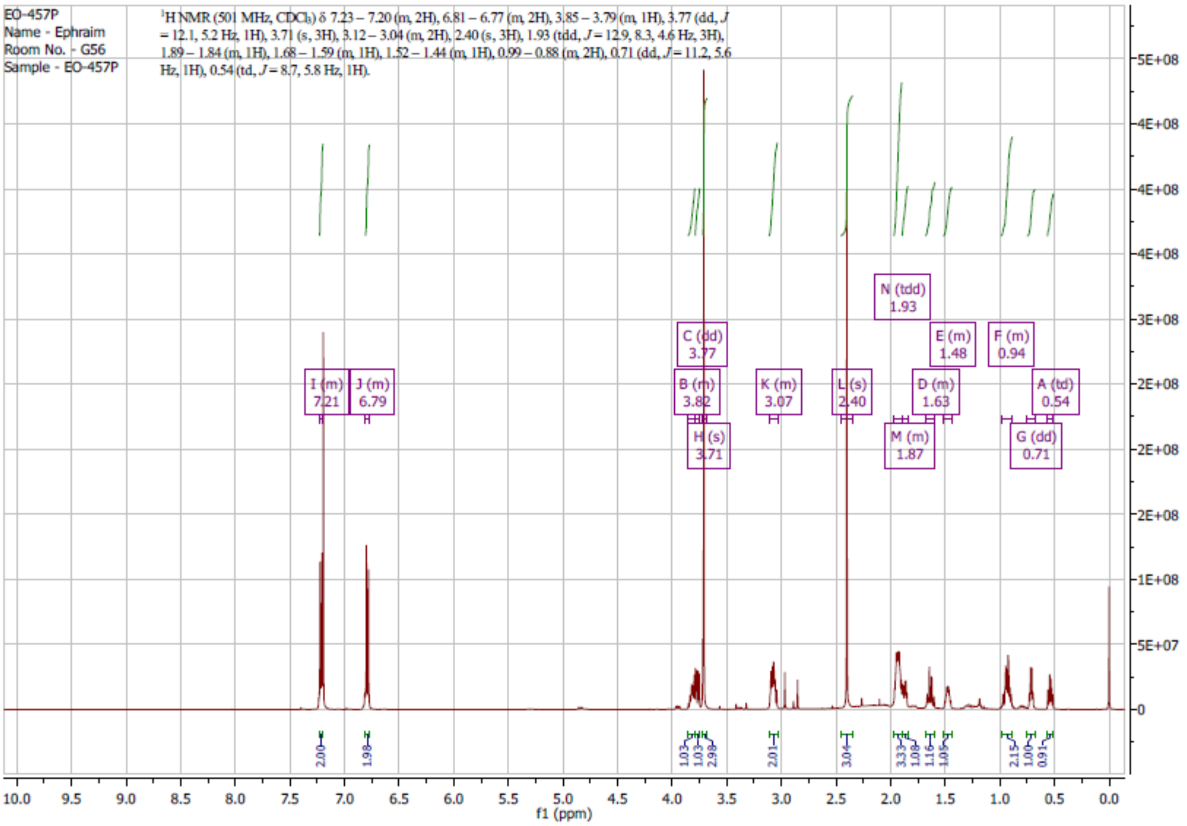

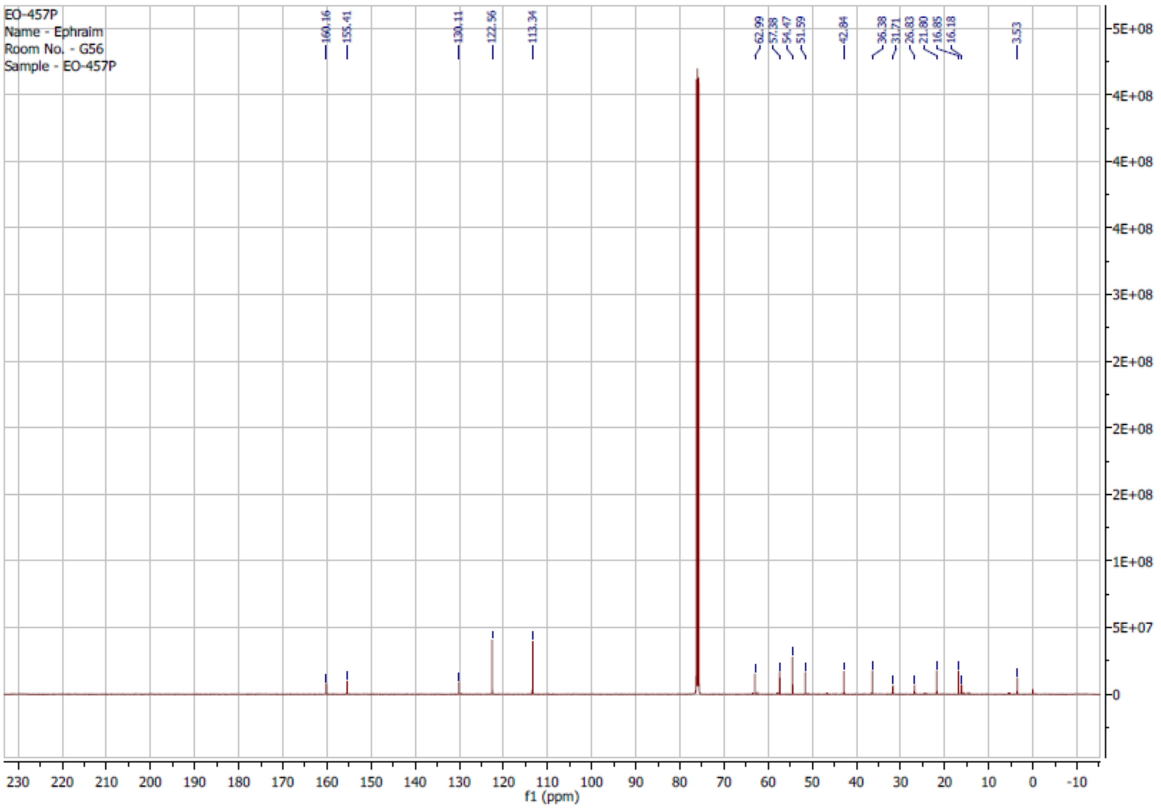

(7a*R,9*R**,11a*S**)-7-(4-Methoxyphenyl)-6-oxodecahydro-1H-pyrrolo[1,2-*d*]quinoxalin-9-yl (2-fluorophenyl)carbamate 30**

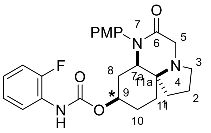

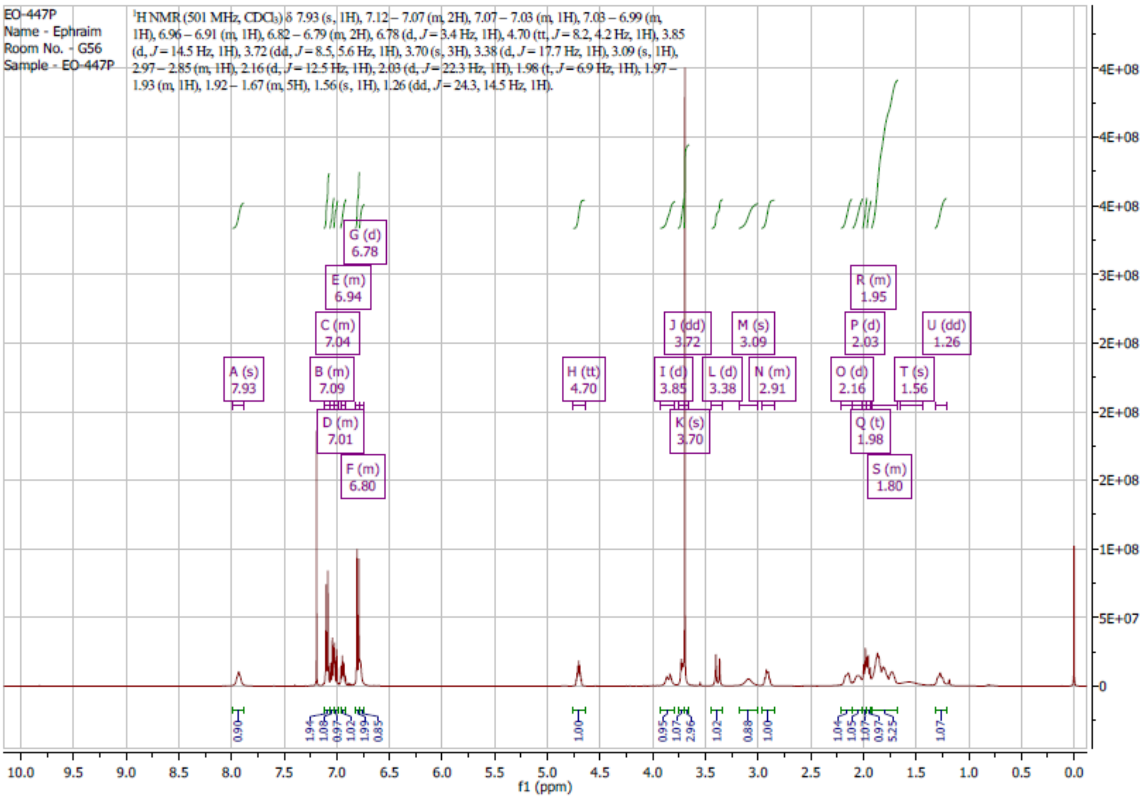

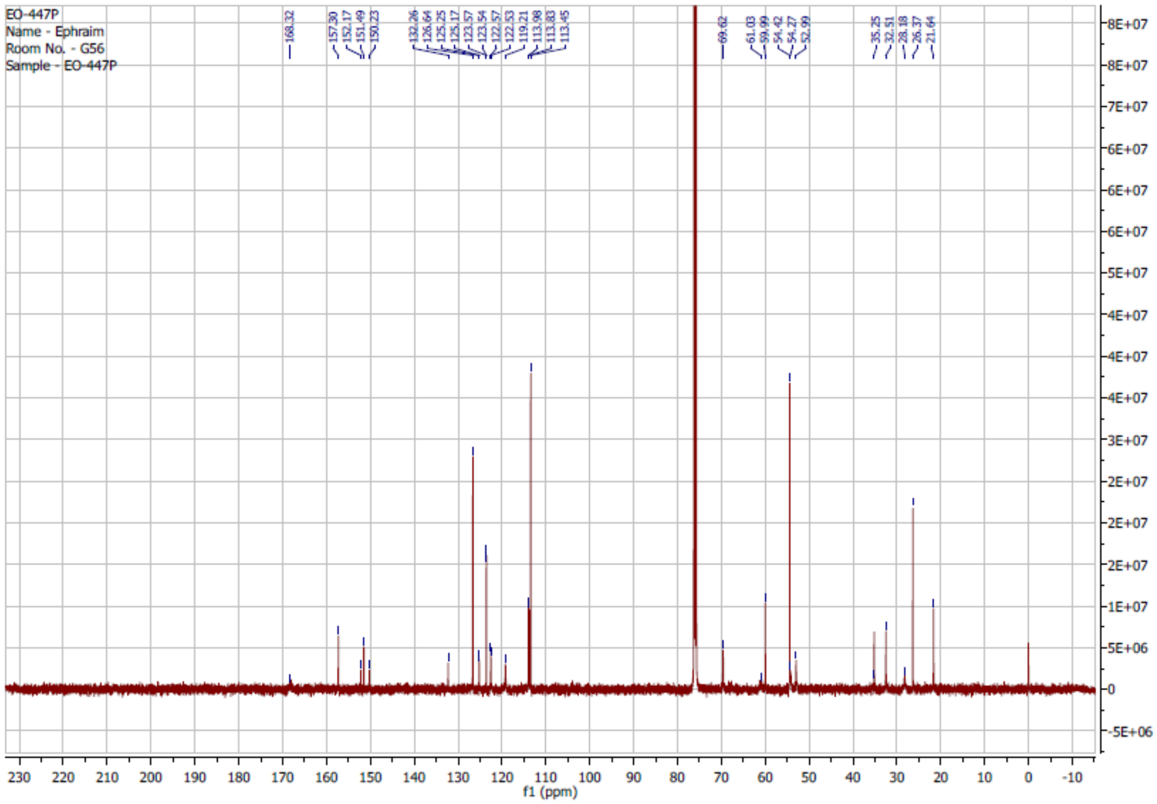

(6a*R,8*R**,10a*S**)-6-Isopropyl-5-oxooctahydro-1*H*,5*H*-benzo[*d*]pyrrolo[1,2-*c*]imidazol-8-yl (2-fluorophenyl)carbamate 31**

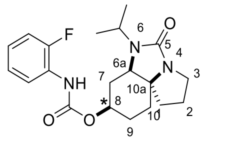

Major diastereomer, *dr* 88:12

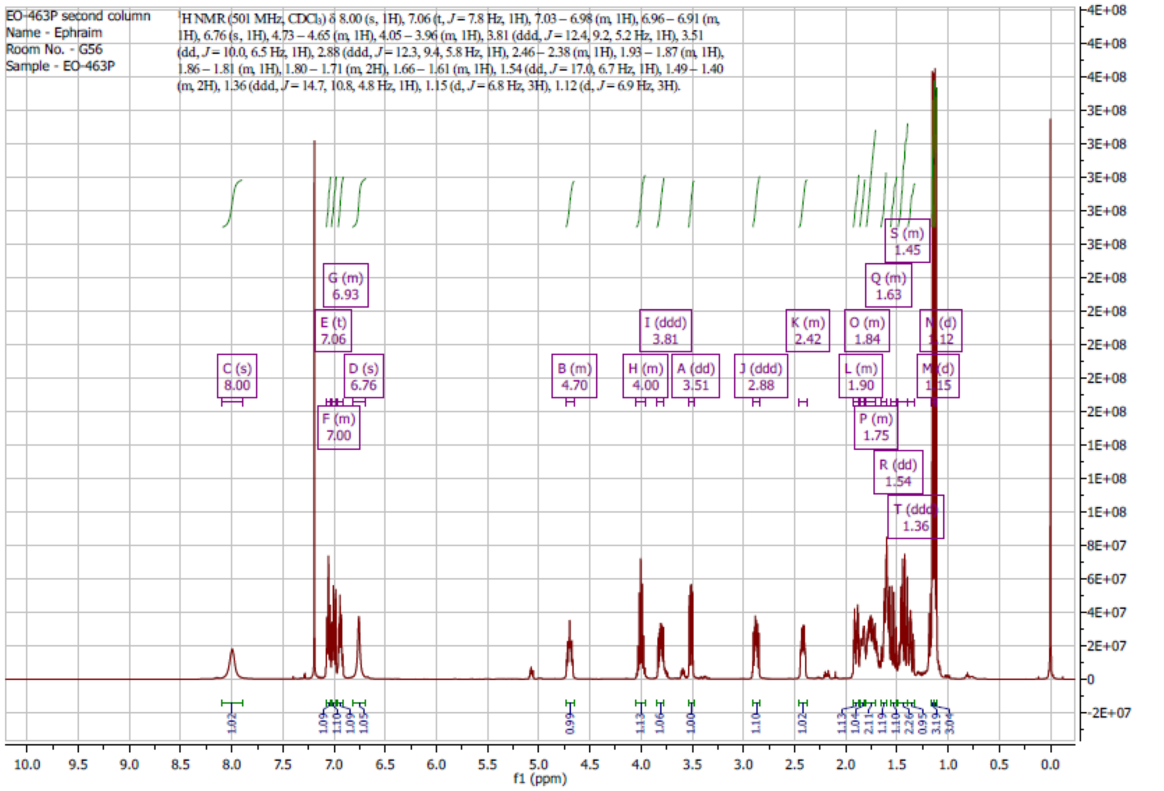

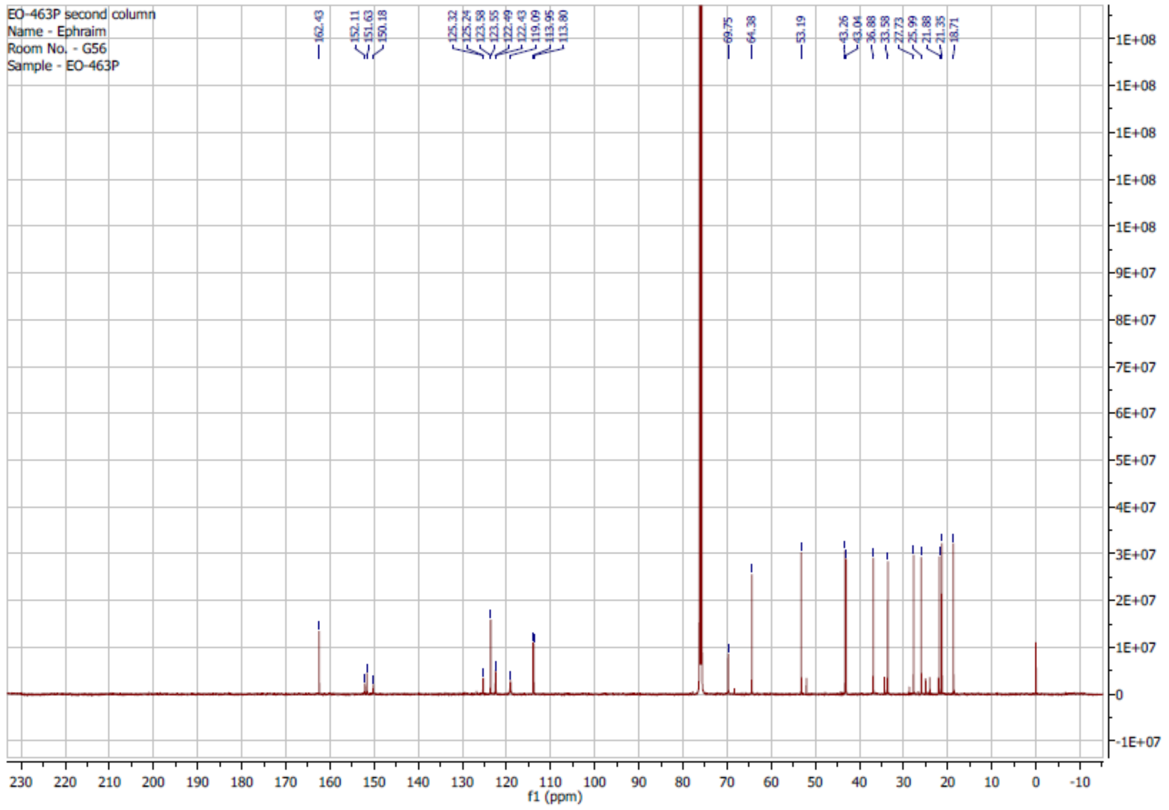

(6a*R,8*R**,10a*S**)-6-Isopropyl-5-oxooctahydro-1*H*,5*H*-benzo[*d*]pyrrolo[1,2-*c*]imidazol-8-yl)-*N*-methylpyridine-3-sulfonamide 32**

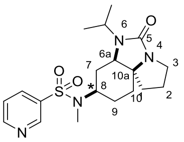

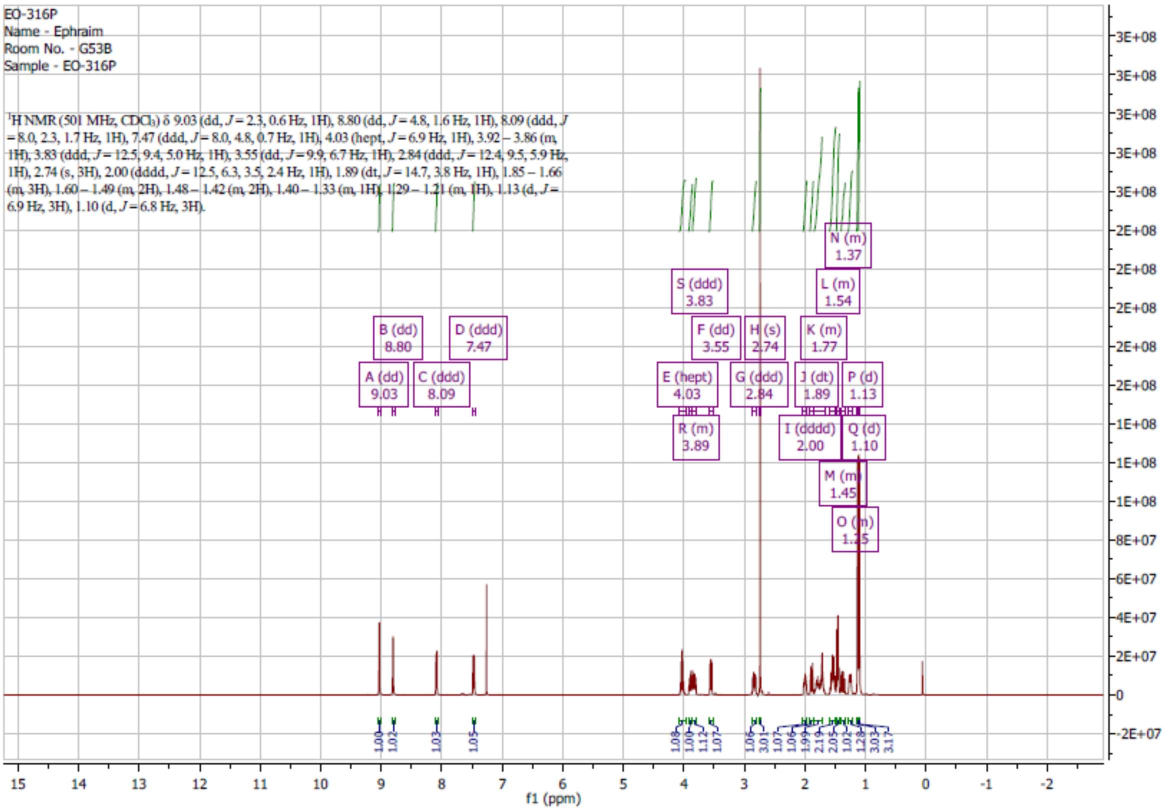

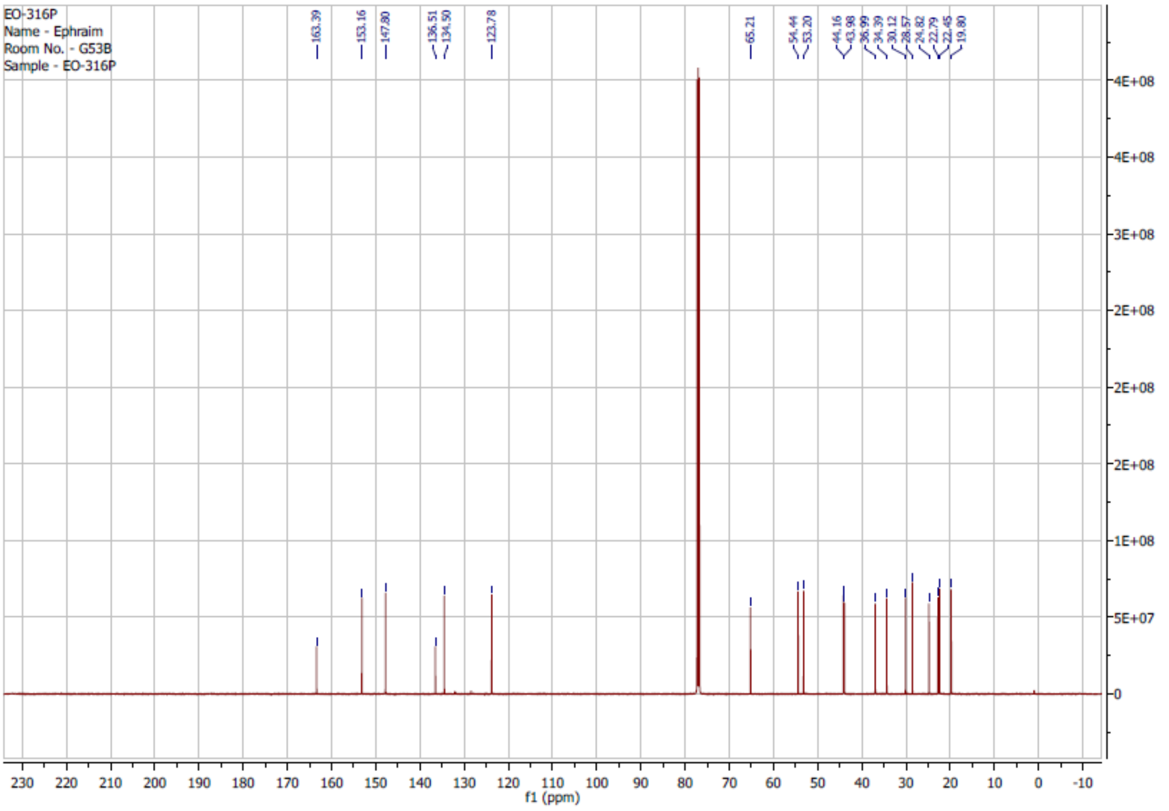

(6a*R,8*R**,10a*S**)-8-(((1-*H*-Imidazol-4-yl)methyl)amino)-6-isopropyloctahydro-1*H*,5*H*-benzo[*d*]pyrrolo[1,2-*c*]imidazol-5-one 33**

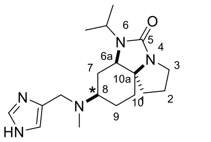

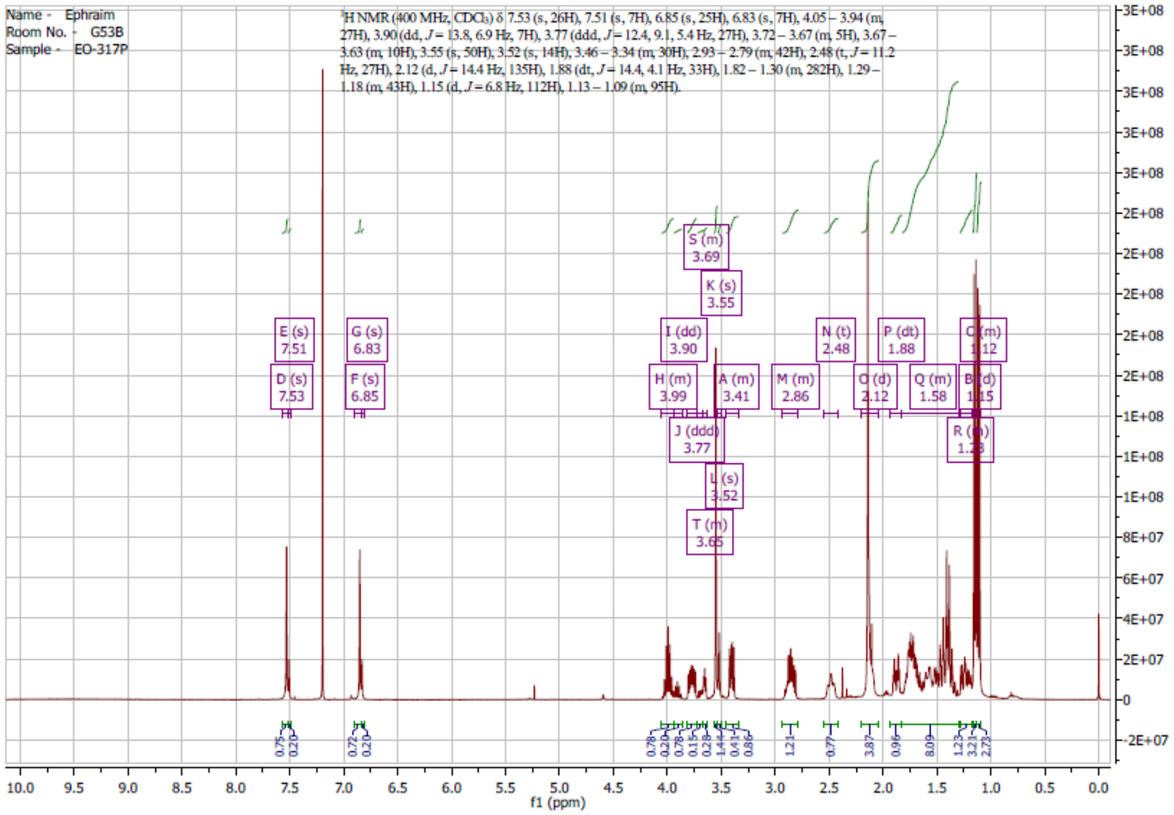

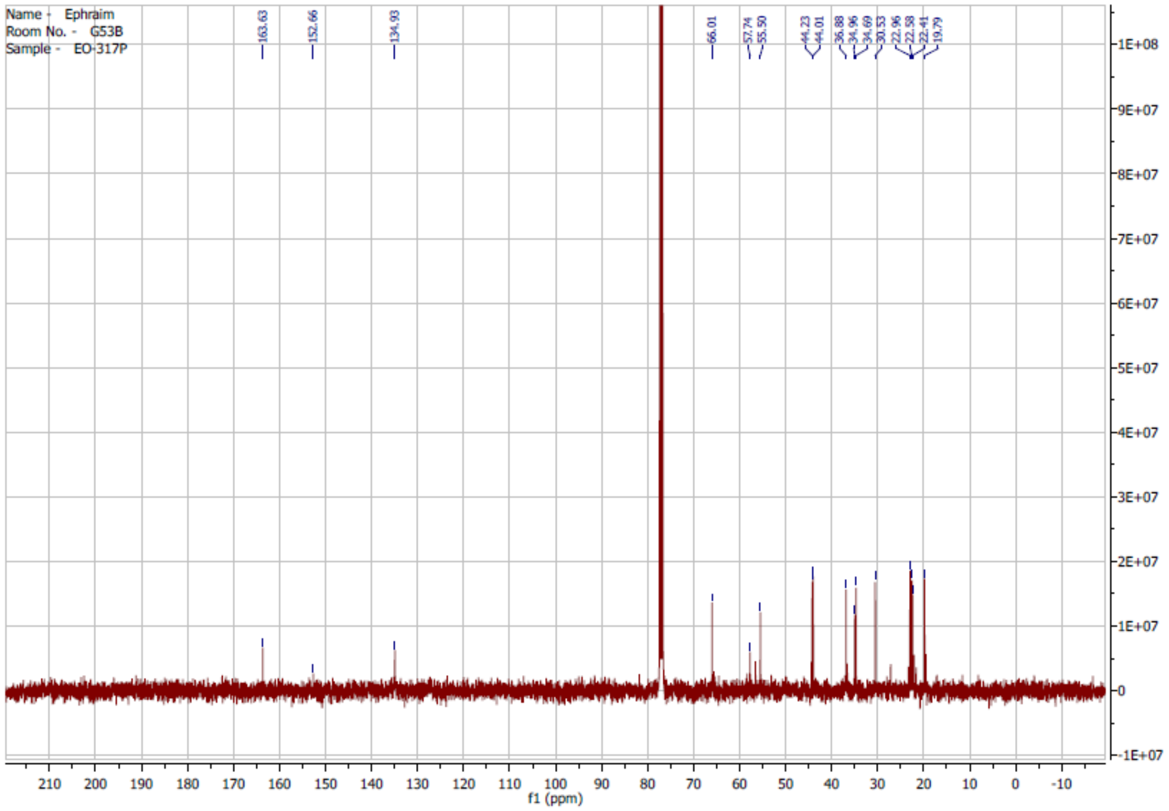

(6a*R**,8*R**,10a*S**)-6-Isopropyl-5-oxooctahydro-1*H*,5*H*-benzo[*d*]pyrrolo[1,2-*c*]imidazol-8-yl)-*N*-methylisoxazole-5-carboxamide 34

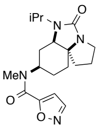

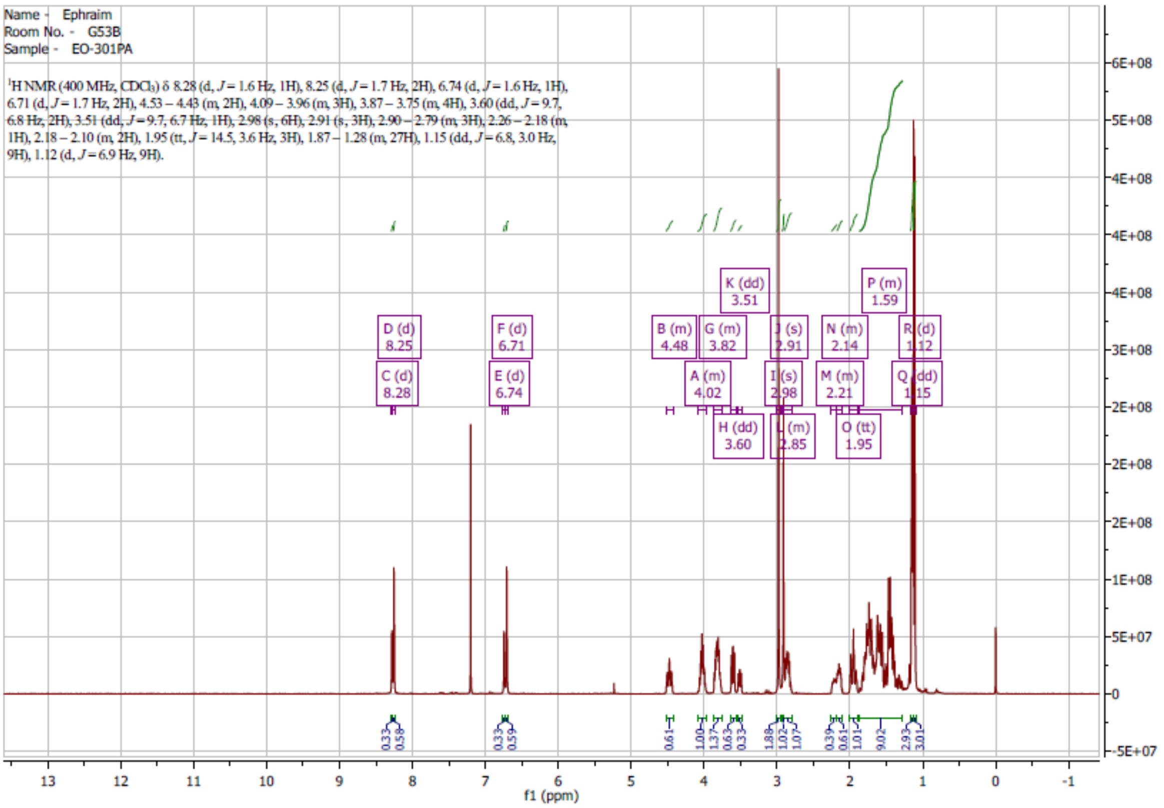

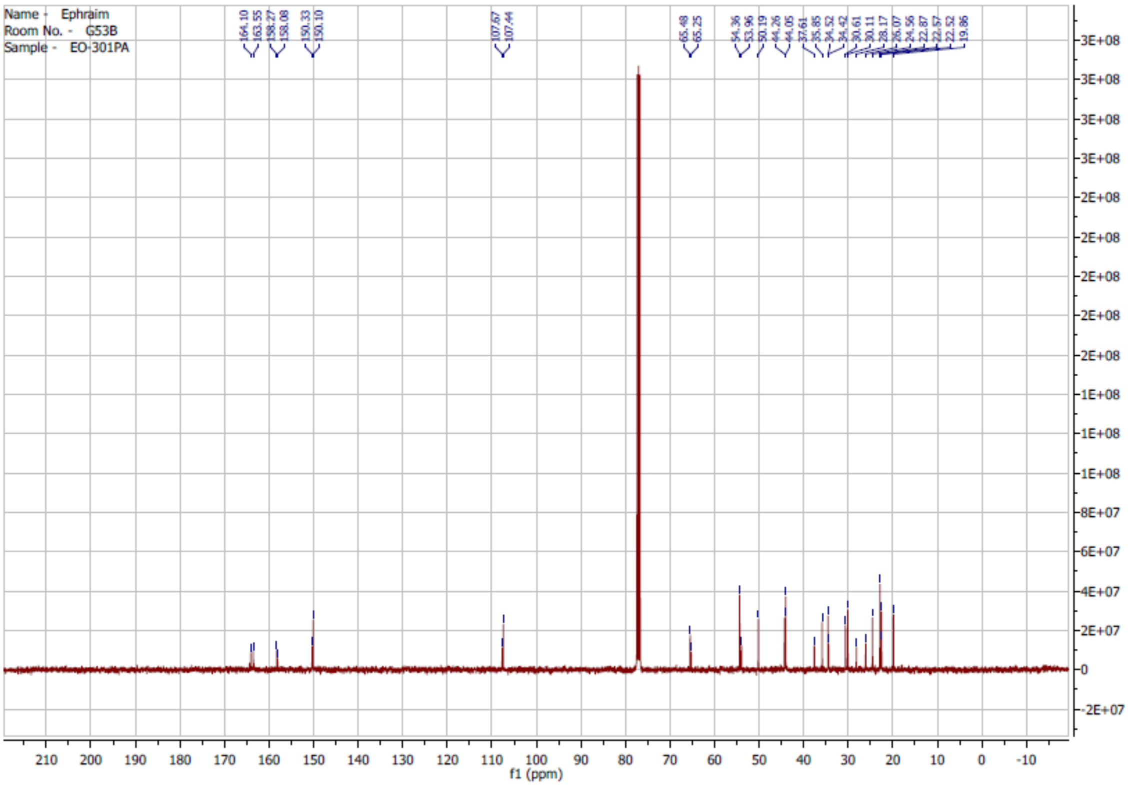

(6a*R*,8*S*,10a*S*)-6-Isopropyl-5-oxooctahydro-1*H*,5*H*-benzo[*d*]pyrrolo[1,2-*c*]imidazol-8-yl)-*N*-methylisoxazole-5-carboxamide 35

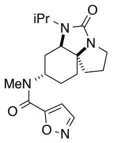

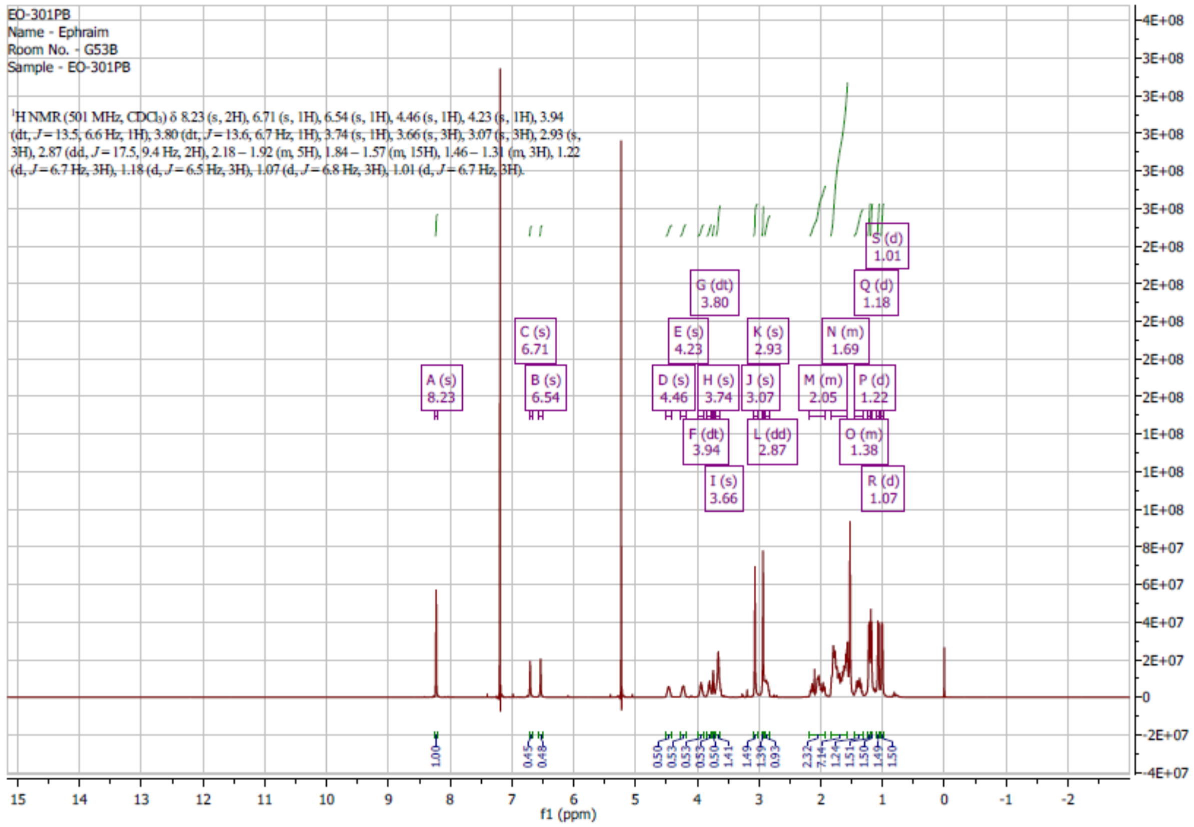

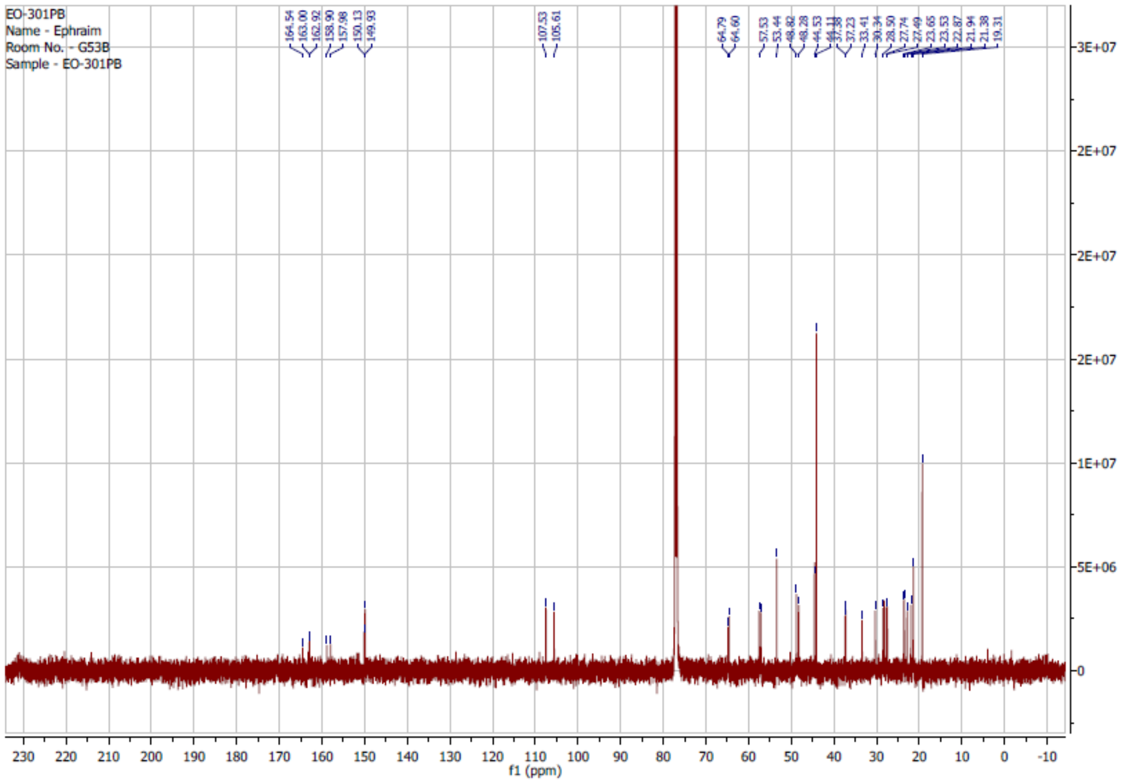

(6a*R,8*R**,10a*S**)-8-[(Furan-2-ylmethyl)amino]-6-isopropyloctahydro-1*H*,5*H*-benzo[*d*]pyrrolo[1,2-*c*]imidazol-5-one 36**

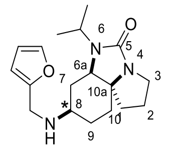

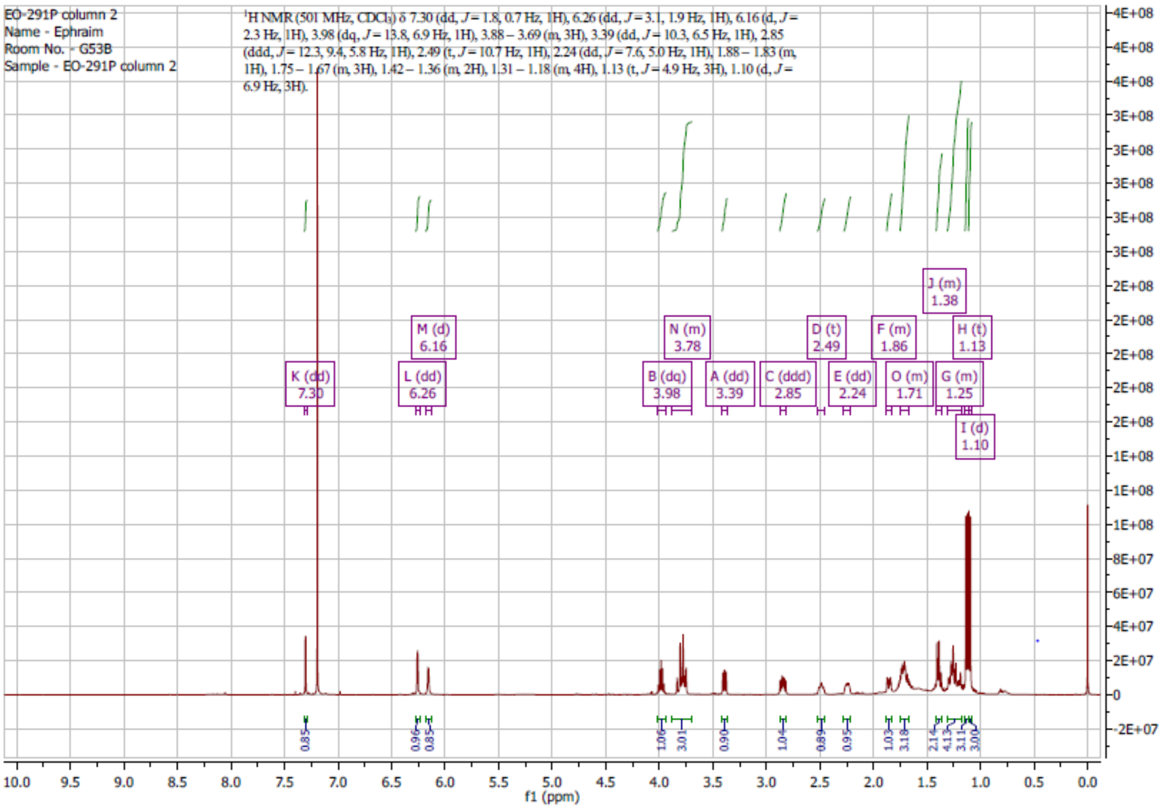

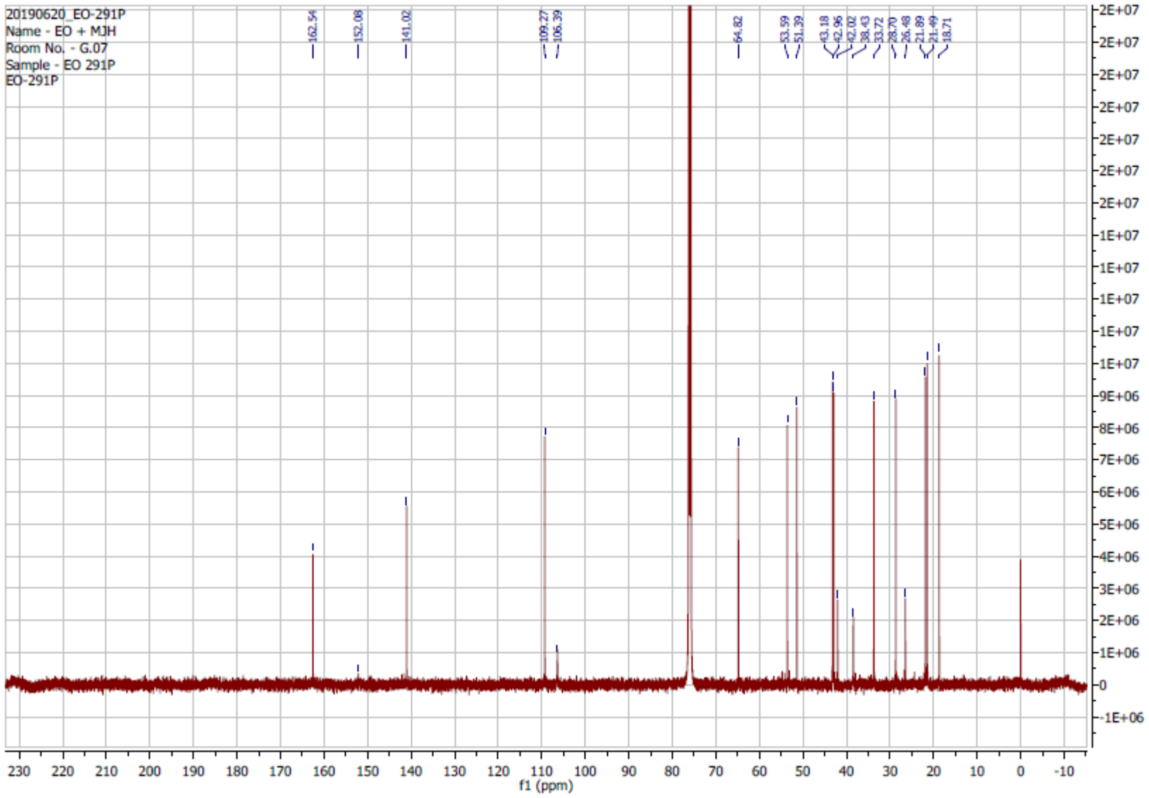

(6a*R,8*R**,10a*S**)-8-(Cyclopropylamino)-6-isopropyloctahydro-1*H*,5*H*-benzo[*d*]pyrrolo[1,2-*c*]imidazol-5-one 37**

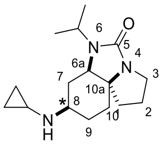

Major diastereomer, *dr* 79:21

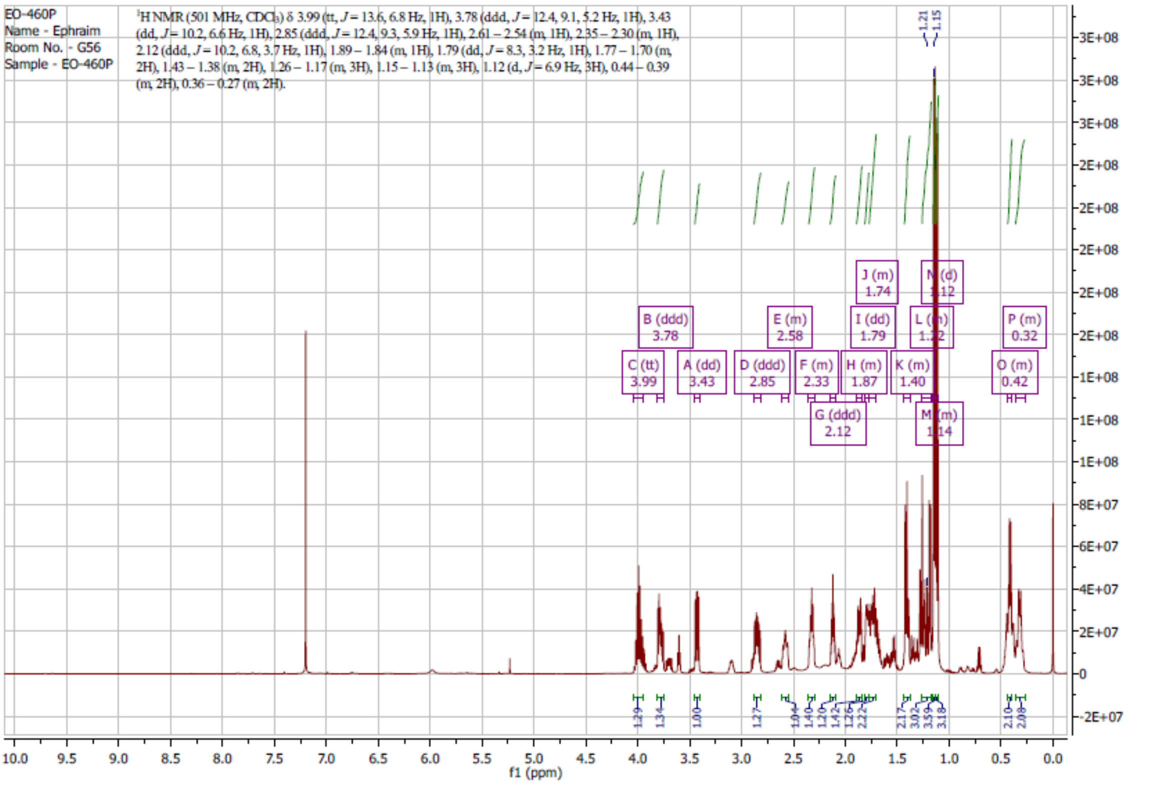

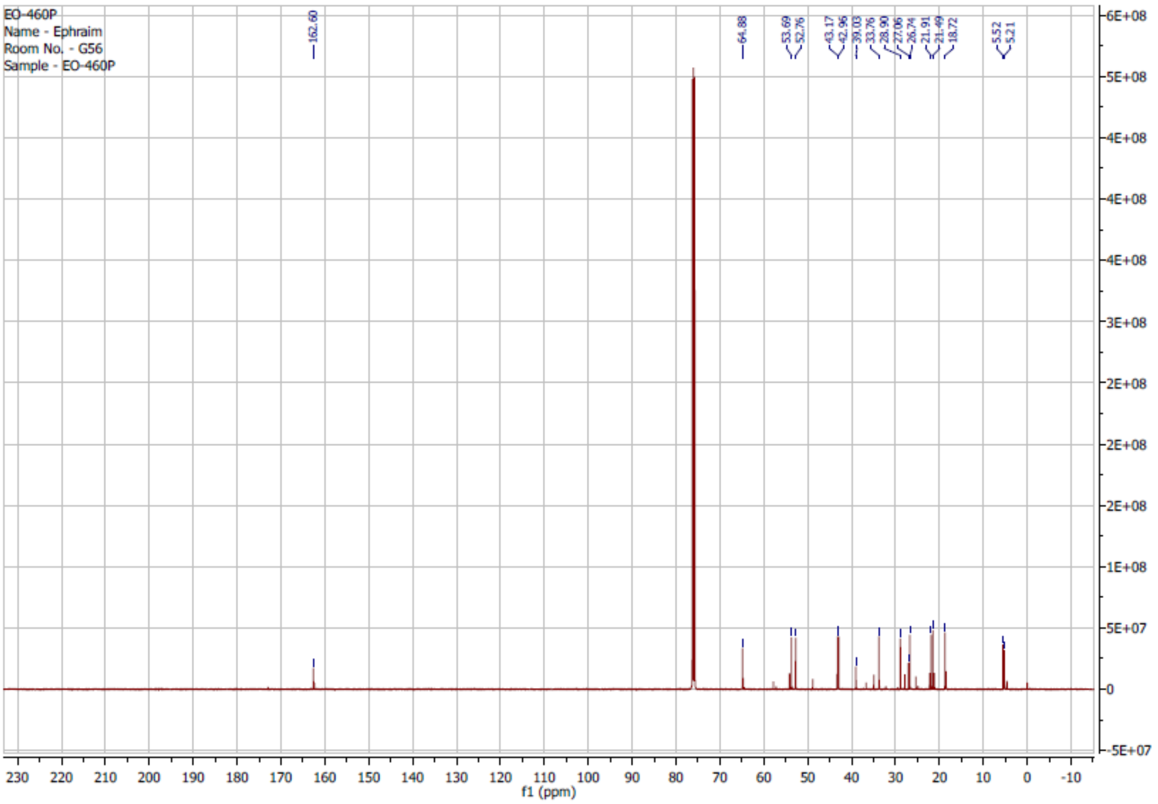

(6a*R,8*R**,10a*S**)-8-[(Furan-2-ylmethyl)amino]-6-(4-methoxyphenyl)octahydro-1*H*,5*H*-benzo[*d*]pyrrolo[1,2-*c*]imidazol-5-one 38**

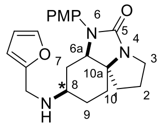

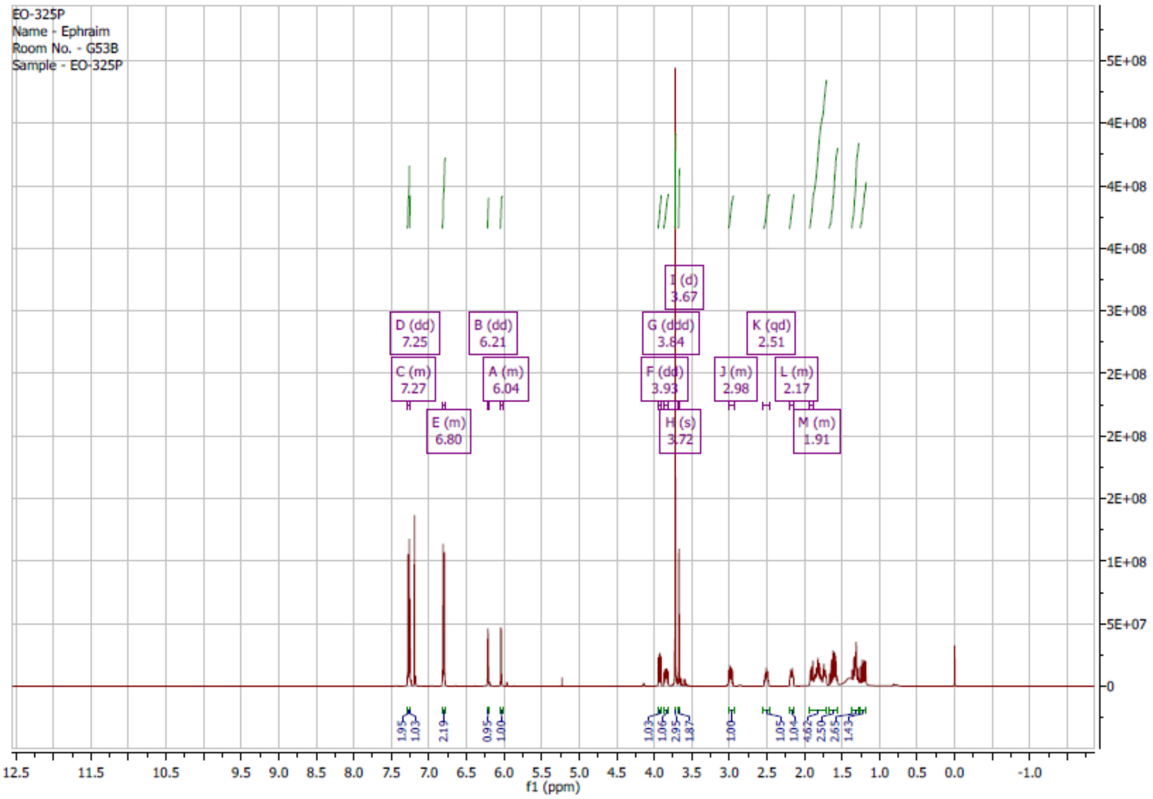

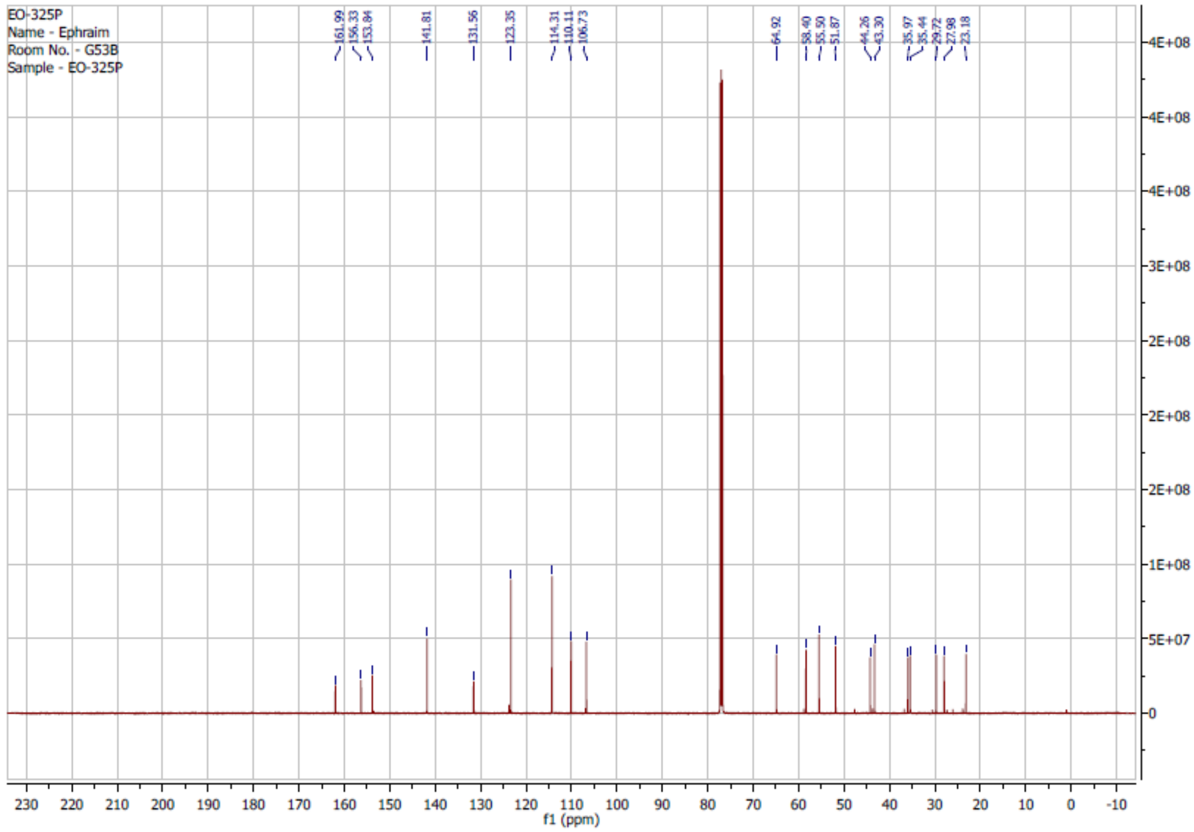

(6a*R,8*R**,10a*S**)-6-(4-Methoxyphenyl)-8-(oxetan-3-ylamino)octahydro-1*H*,5*H*-benzo[*d*]pyrrolo[1,2-*c*]imidazol-5-one 39**

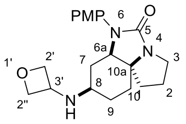

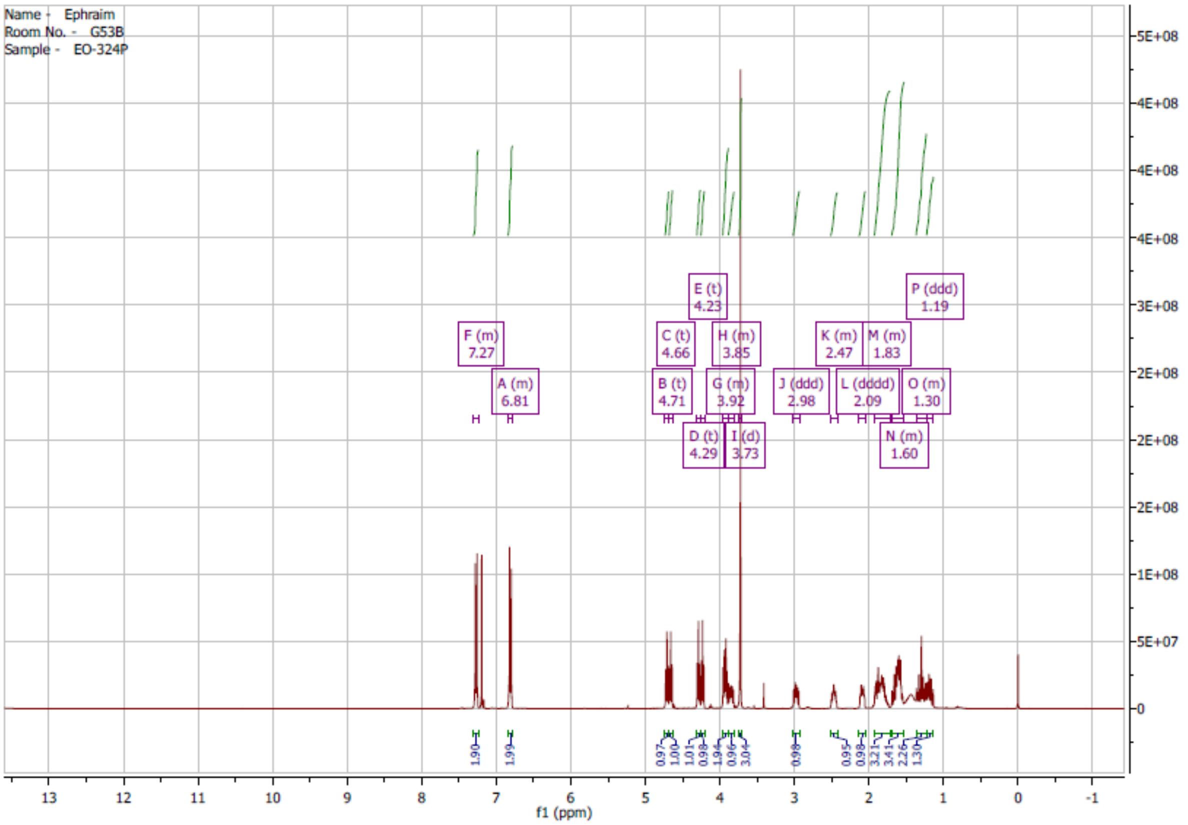

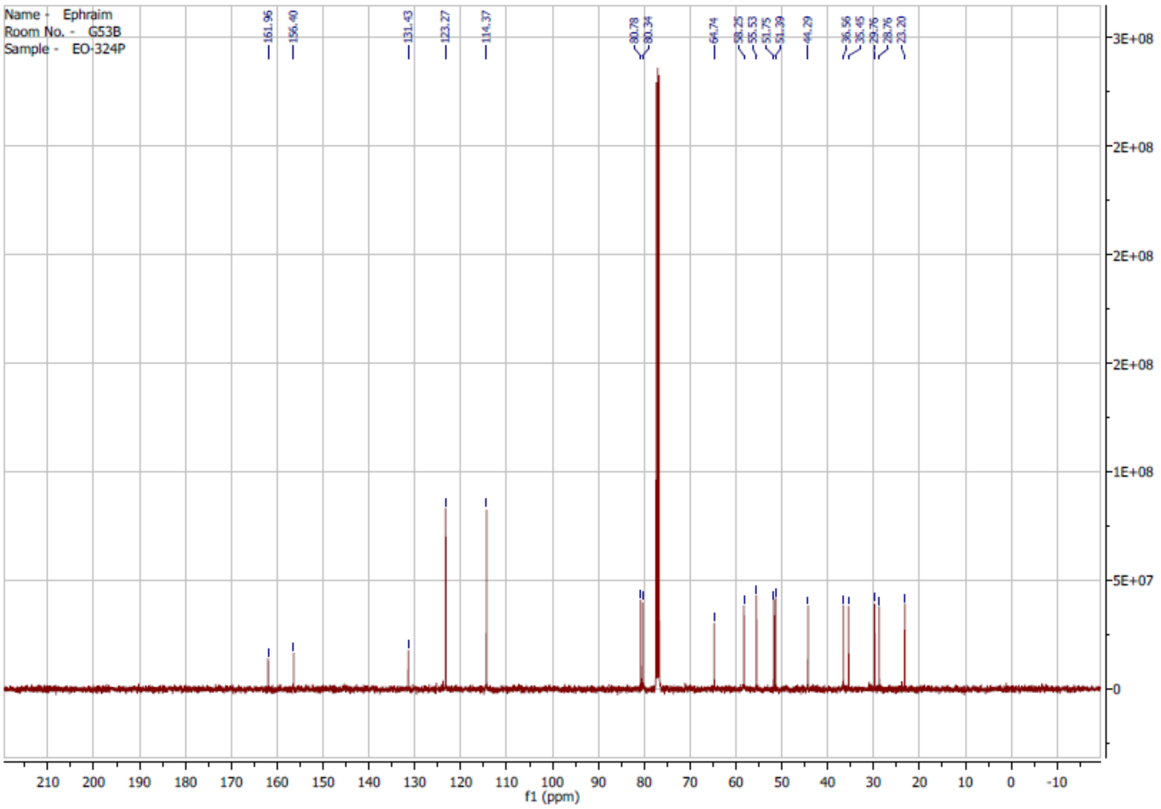

(6a*R**,8*R**,10a*S**)-8-(Azetidin-1-yl)-6-(4-methoxyphenyl)octahydro-1*H*,5*H*-benzo[*d*]pyrrolo[1,2-*c*]imidazol-5-one 40

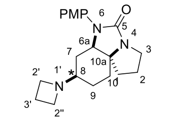

Major diastereomer, *dr* 95:5

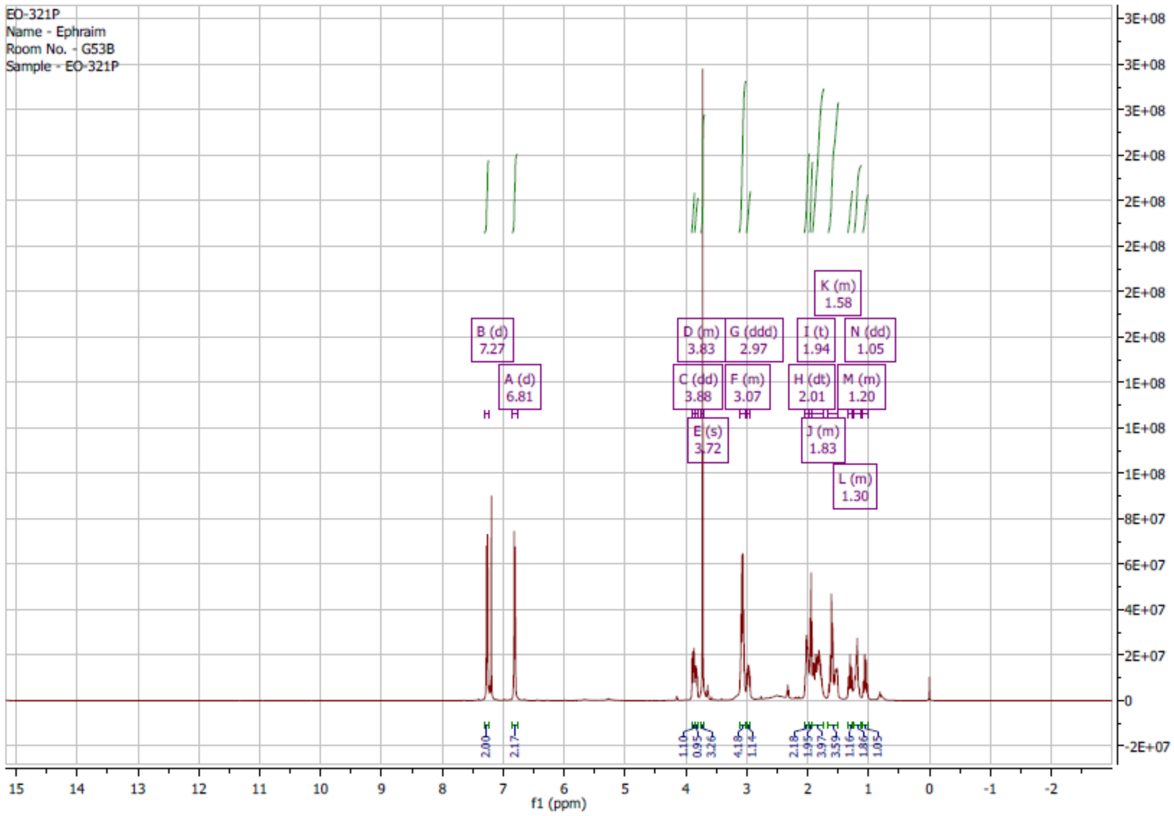

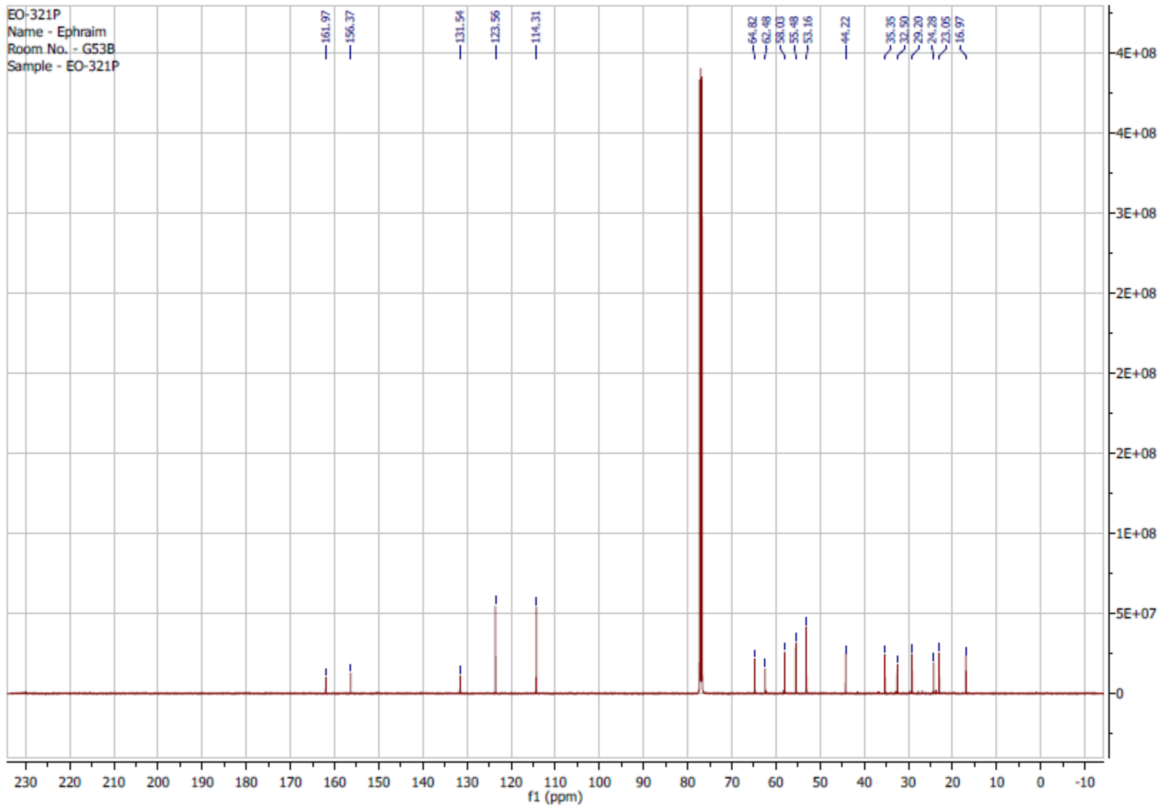

(6a*R**,10a*S**)-Hexahydro-1*H*,5*H*-benzo[*d*]pyrrolo[1,2-*c*]imidazole-5,8(6*H*)-dione S9

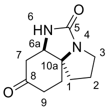

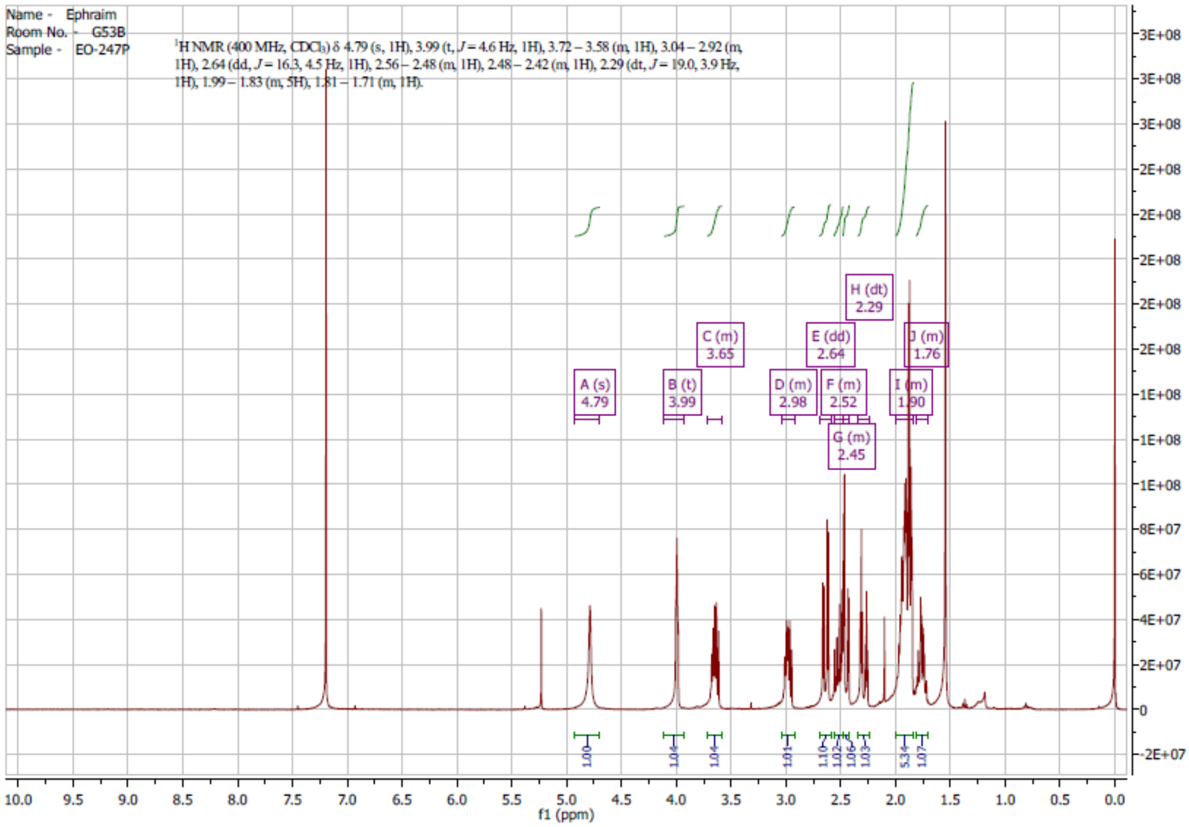

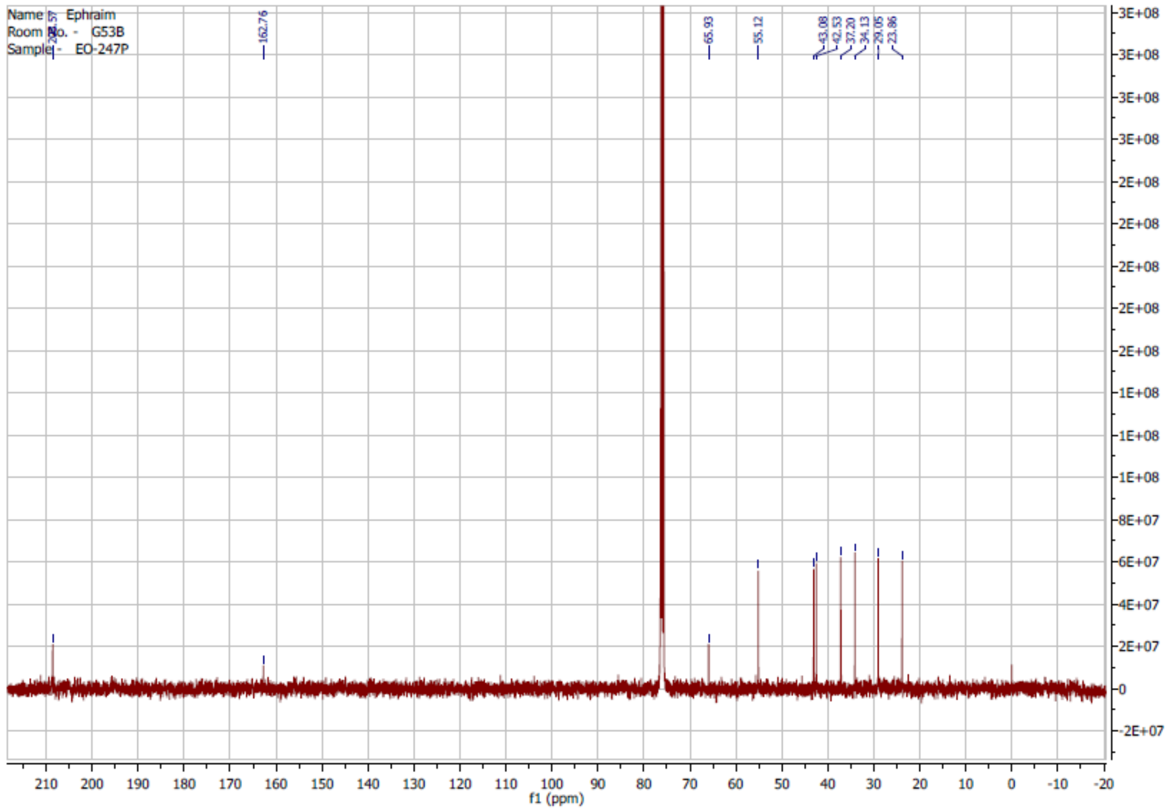

(6a*R,8*R**,10a*S**)-8-Hydroxyoctahydro-1*H*,5*H*-benzo[*d*]pyrrolo[1,2-*c*]imidazole-5-one 41**

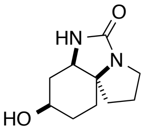

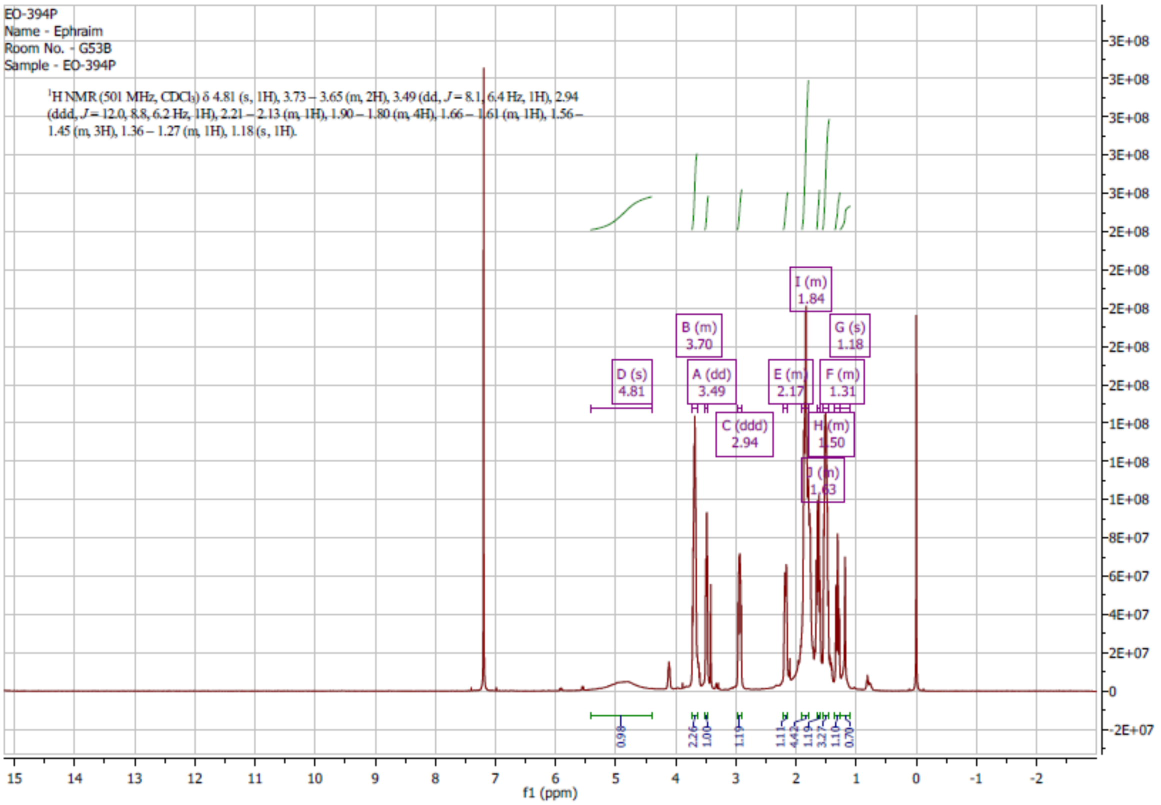

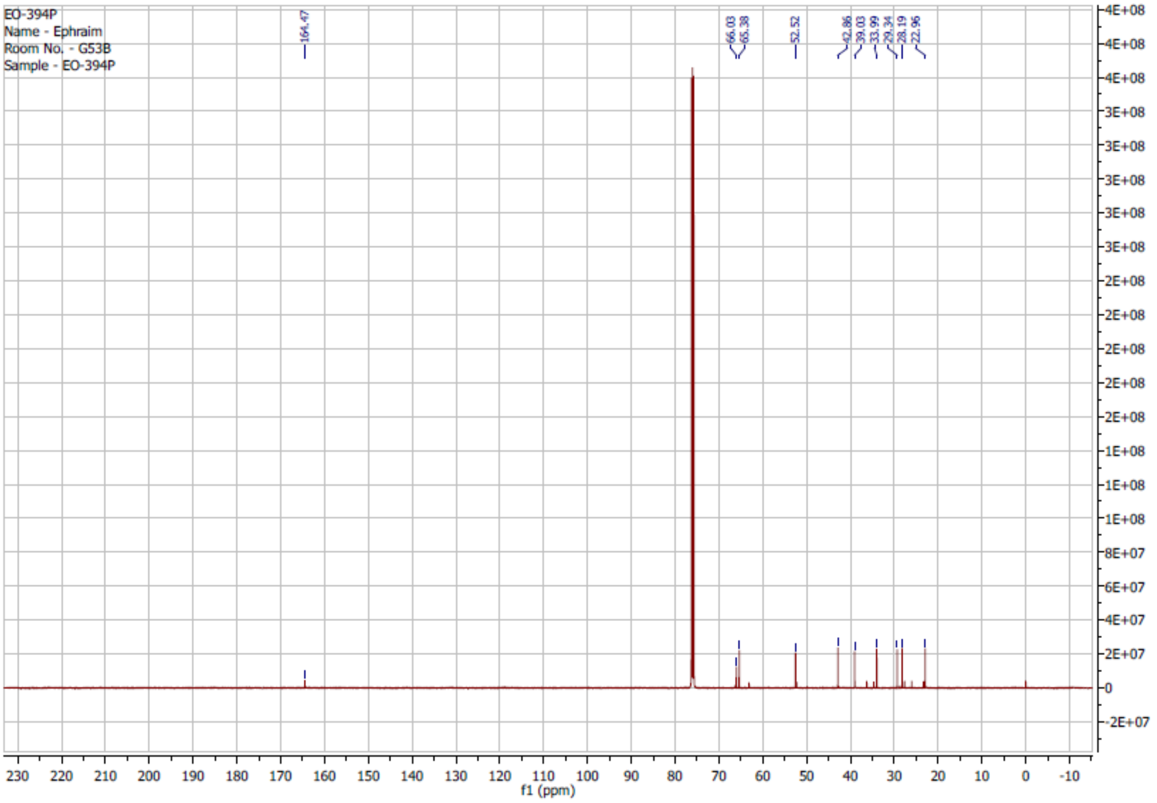

(6a*R**,8*S**,8a*R**,9a*S**, 9b*S**)-8-Hydroxy-6-isopropyloctahydro-1*H*-cyclopropa[5,6]benzo[1,2-*d*]pyrrolo[1,2-*c*]imidazol-5(6*H*)-one 42

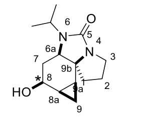

133a
Major diastereomer, *dr* 90:10

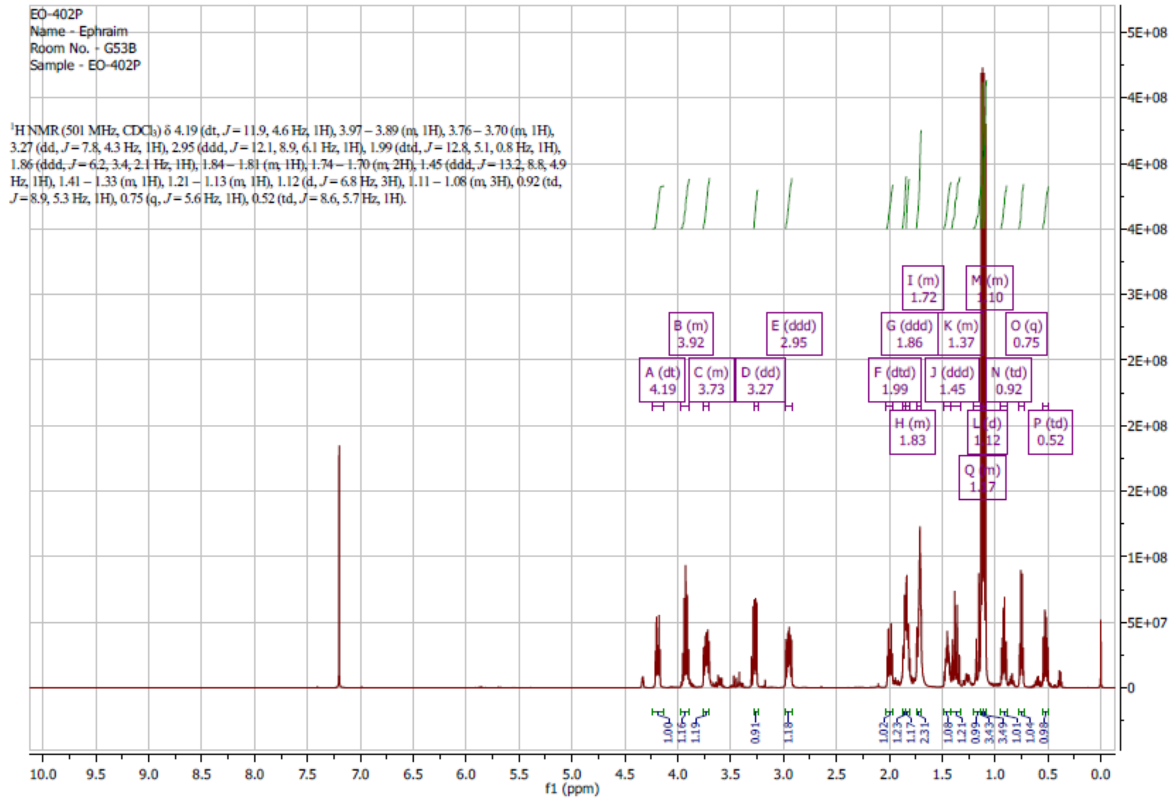

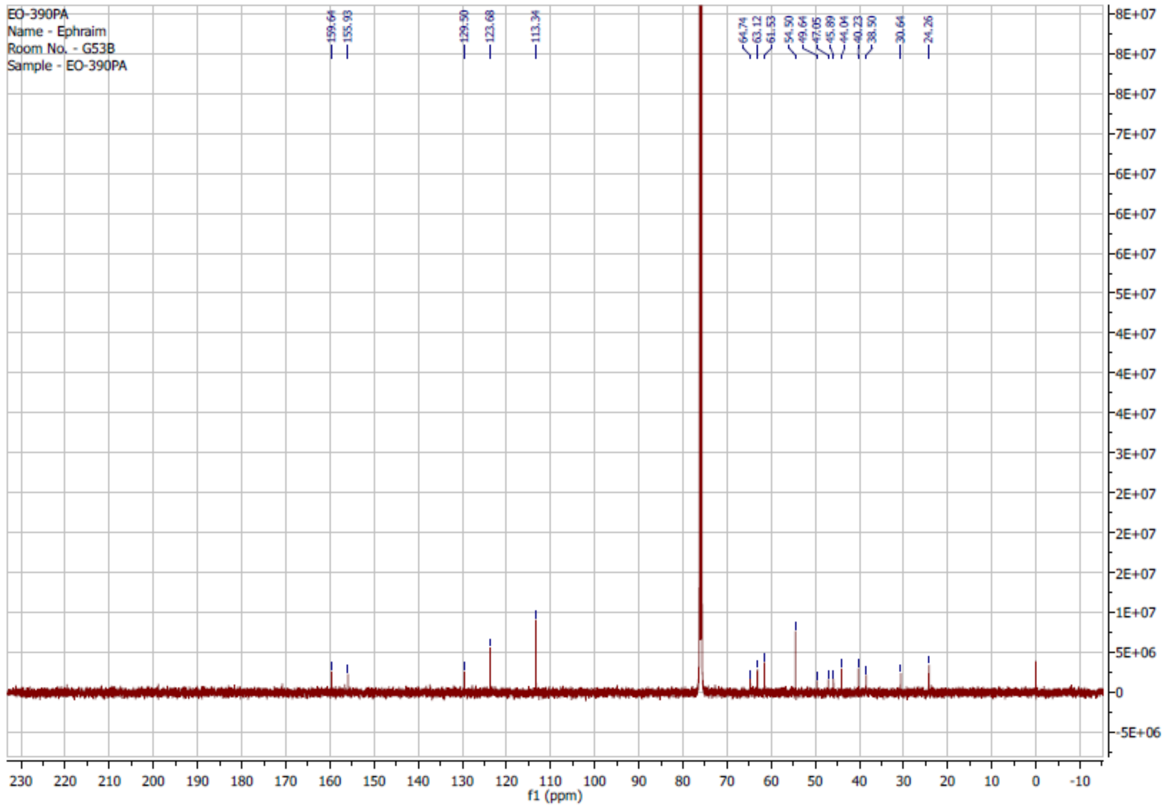

(6a*R,8*S**,8a*R**,9a*S**, 9b*S**)-6-Isopropyl-8-(methylamino)octahydro-1*H*-cyclopropa[5,6]benzo[1,2-*d*]pyrrolo[1,2-*c*]imidazol-5(6*H*)-one 43**

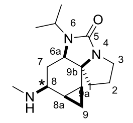

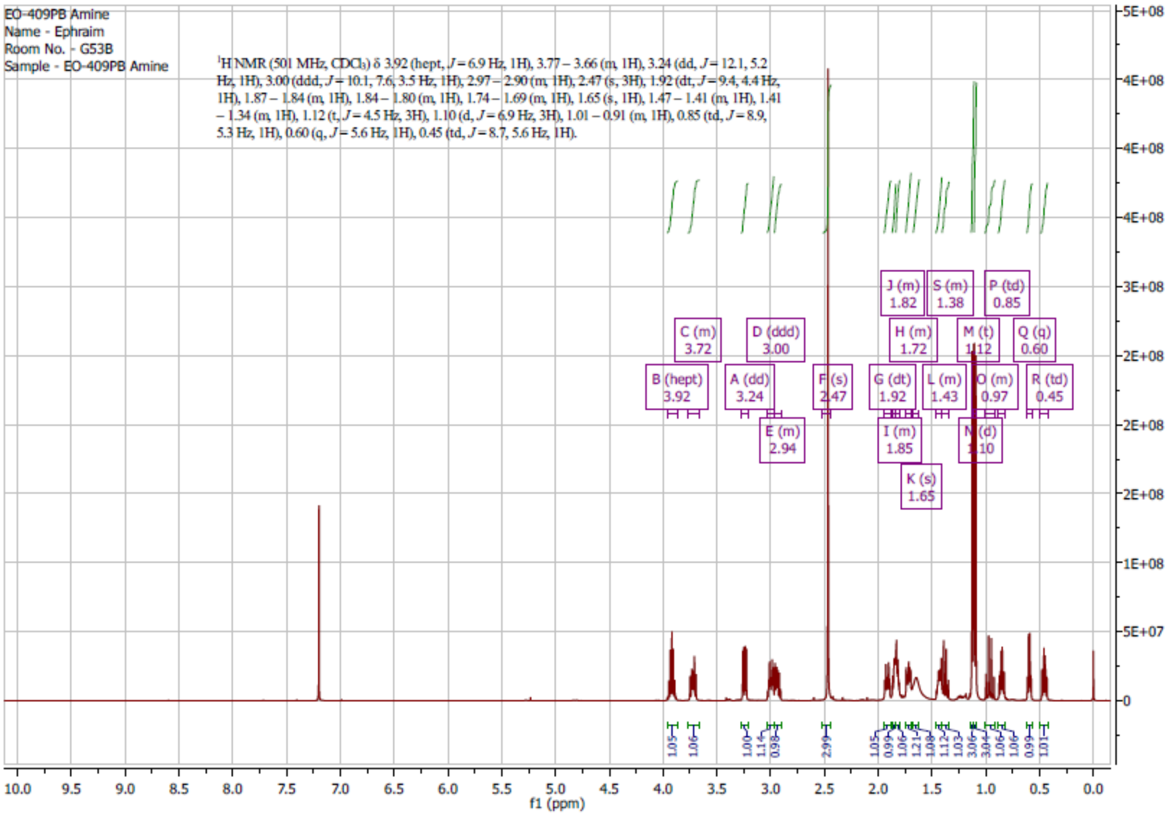

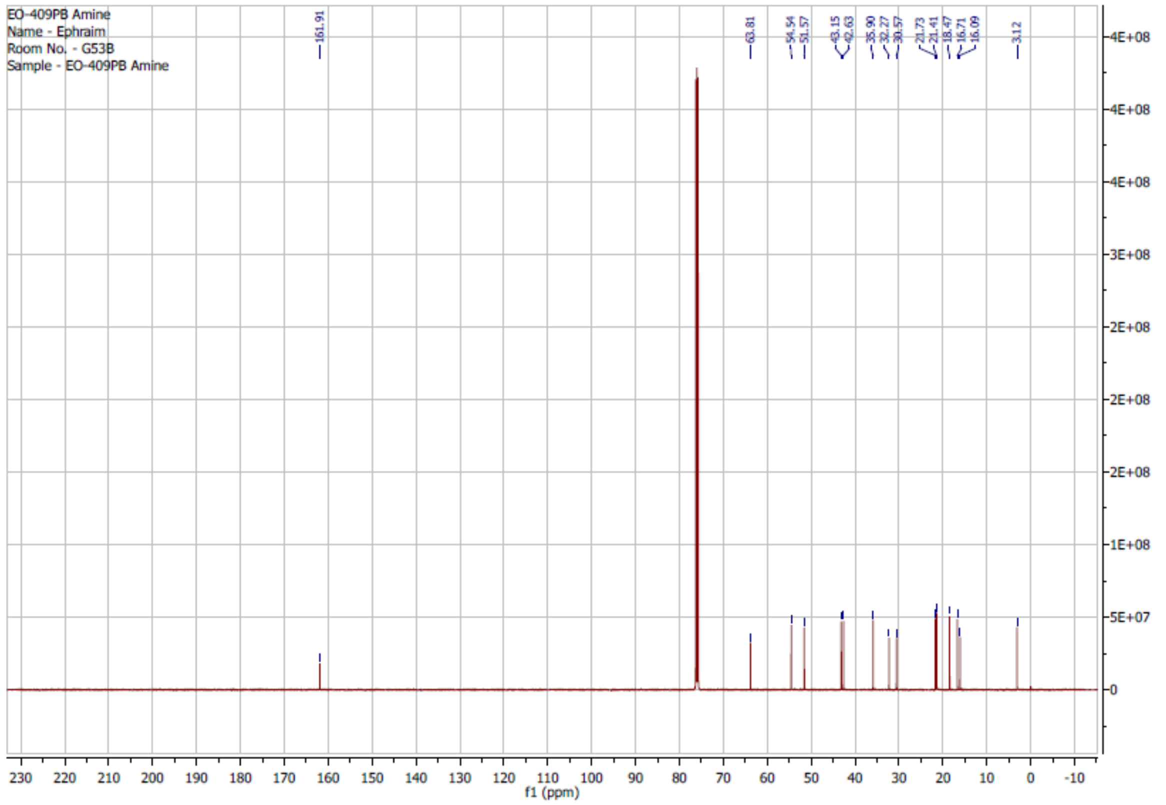

(6a*R,8*S**,8a*R**,9a*S**, 9b*S**)-8-Hydroxy-6-(4-methoxyphenyl)octahydro-1*H*-cyclopropa[5,6]benzo[1,2-*d*]pyrrolo[1,2-*c*]imidazol-5(6*H*)-ol 44**

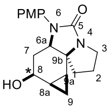

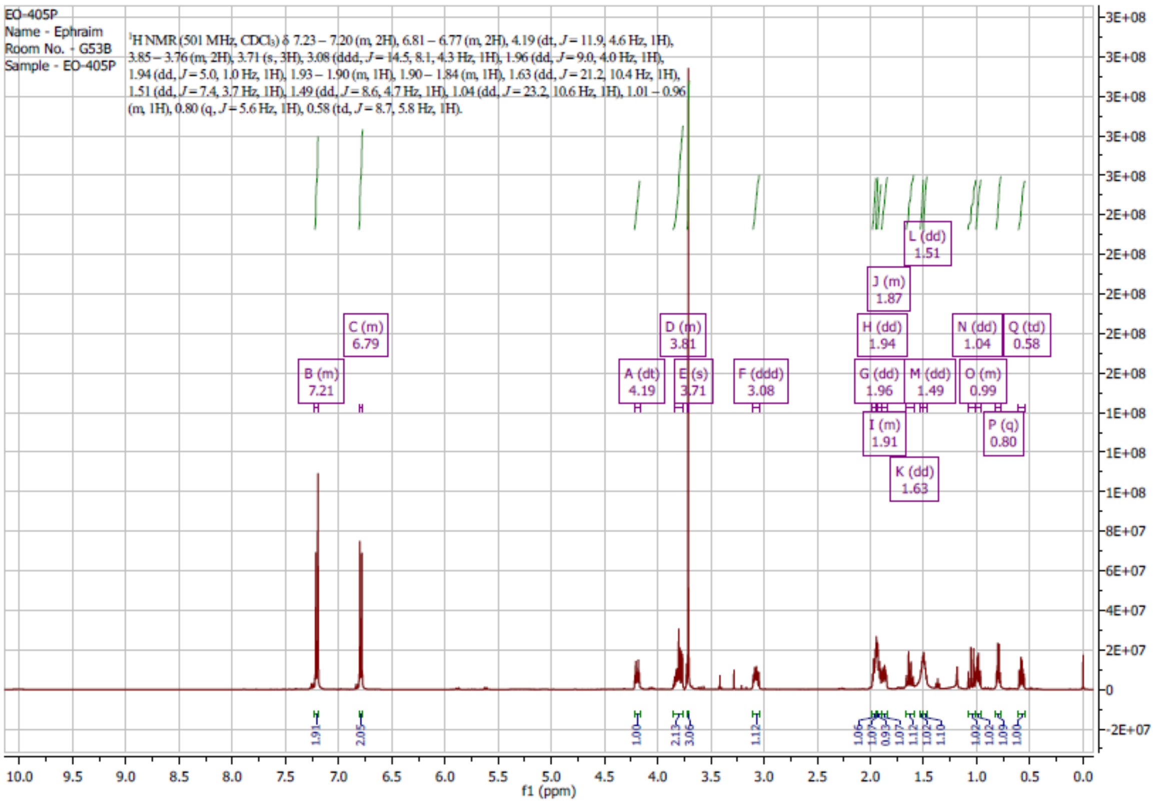

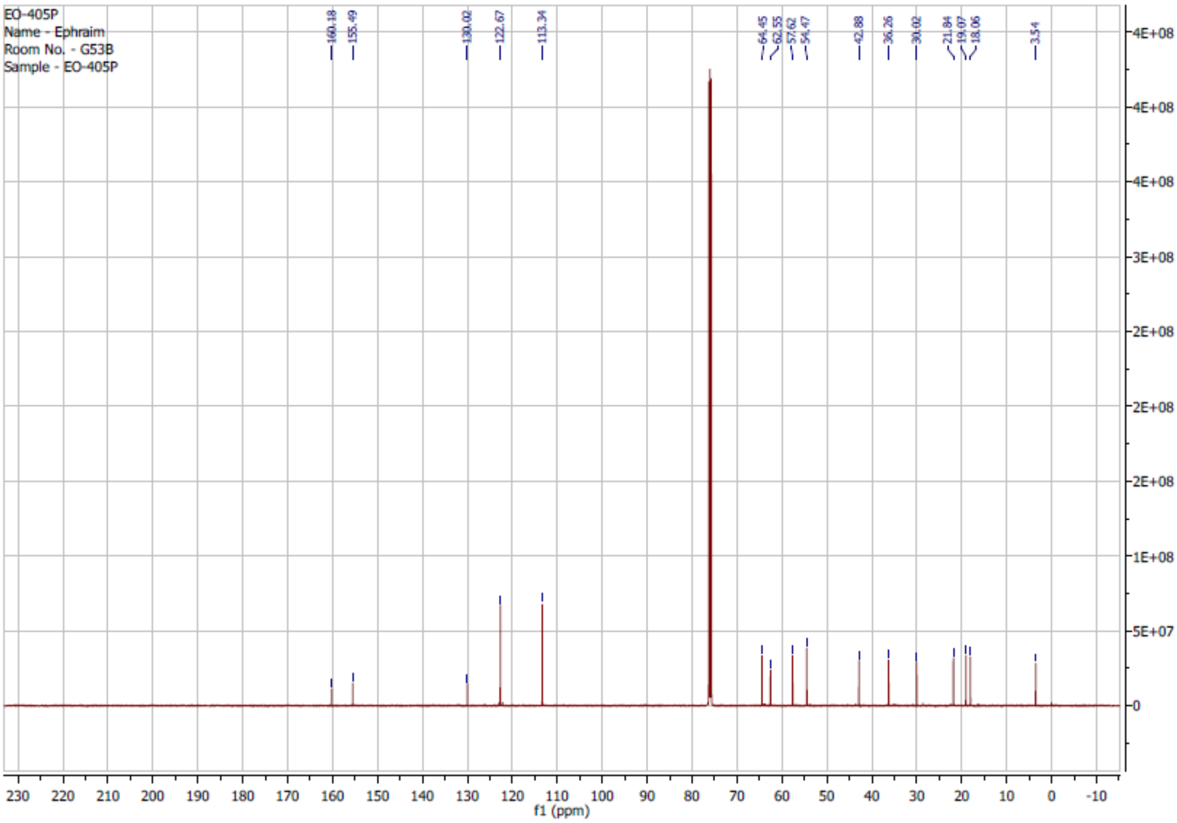

_N_-[(6a_R_*,8_S_*,8a_R_*,9a_S_*,9b_S_*)-6-(4-Methoxyphenyl)-5-oxodecahydro-1_H_-cyclopropa[5,6]benzo[1,2-_c_]imidazol-8-yl]methanesulfonamide 45

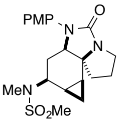

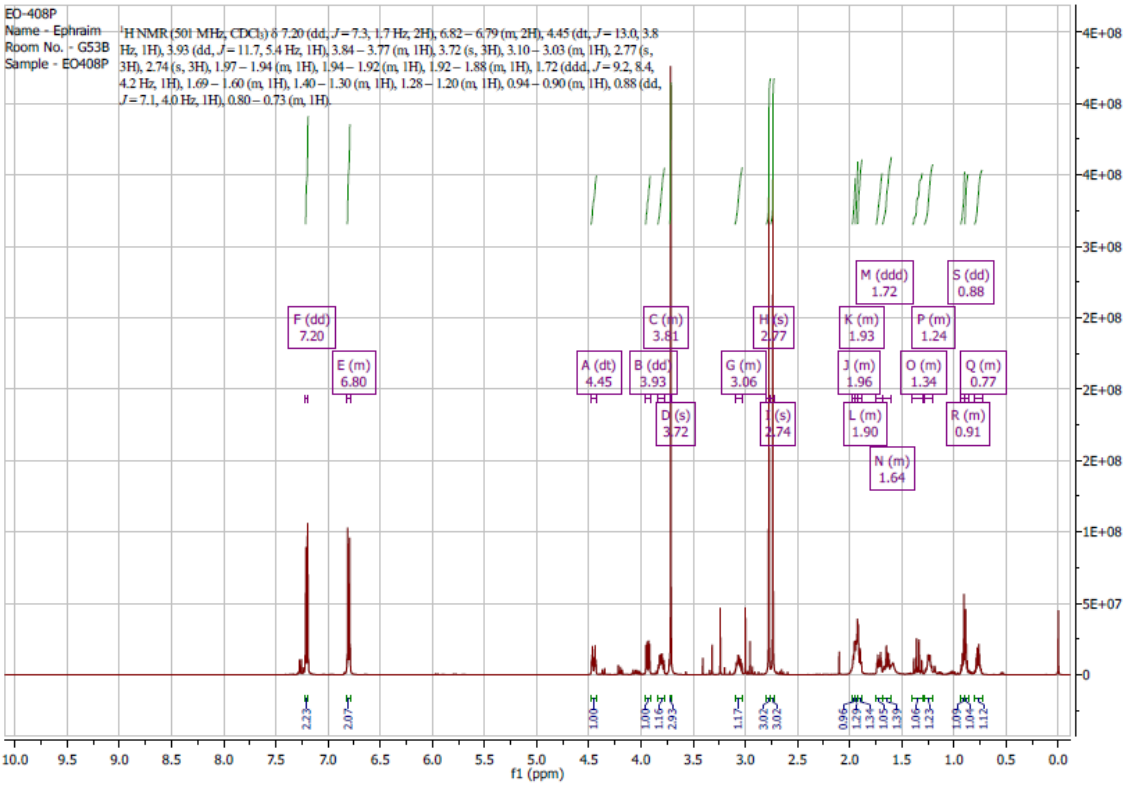

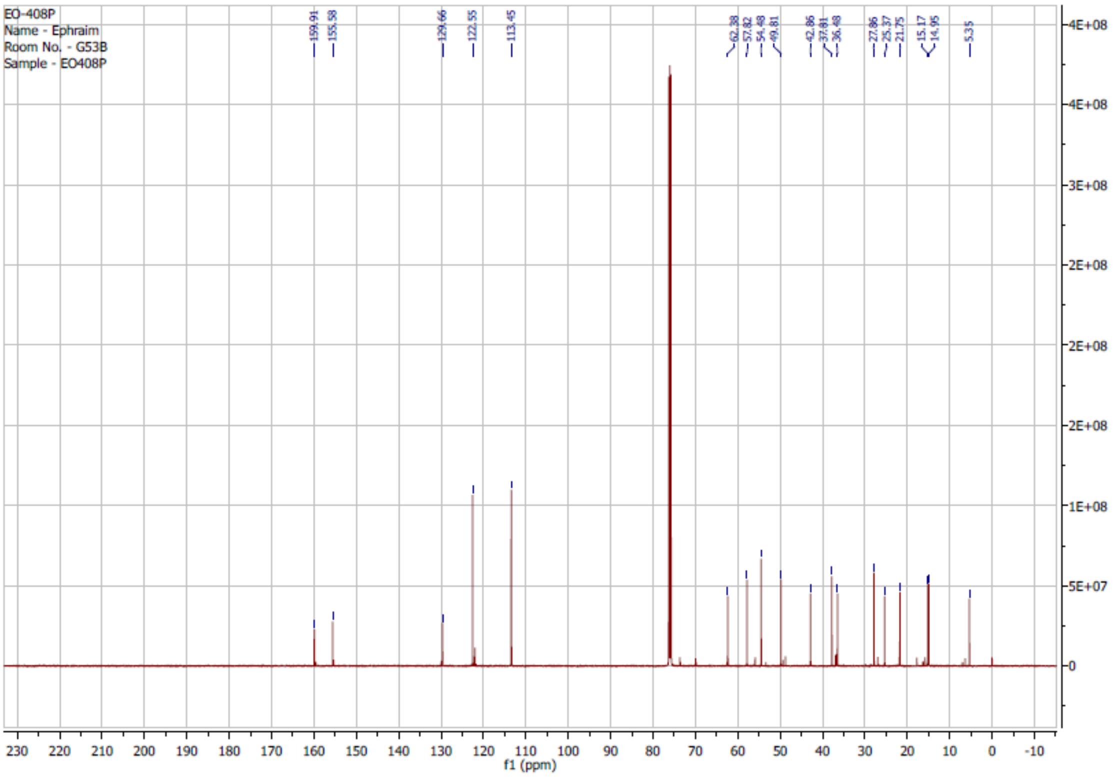

(6a*R,8*S**,8a*R**,9a*S**,9b*S**)-6-(4-Methoxyphenyl)-8-(2-oxa-6-azaspiro[3.3]heptan-6-yl)octahydro-1*H*-cyclopropa[5,6]benzo[1,2-*d*]imidazol-5(6*H*)-one 46**

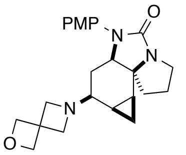

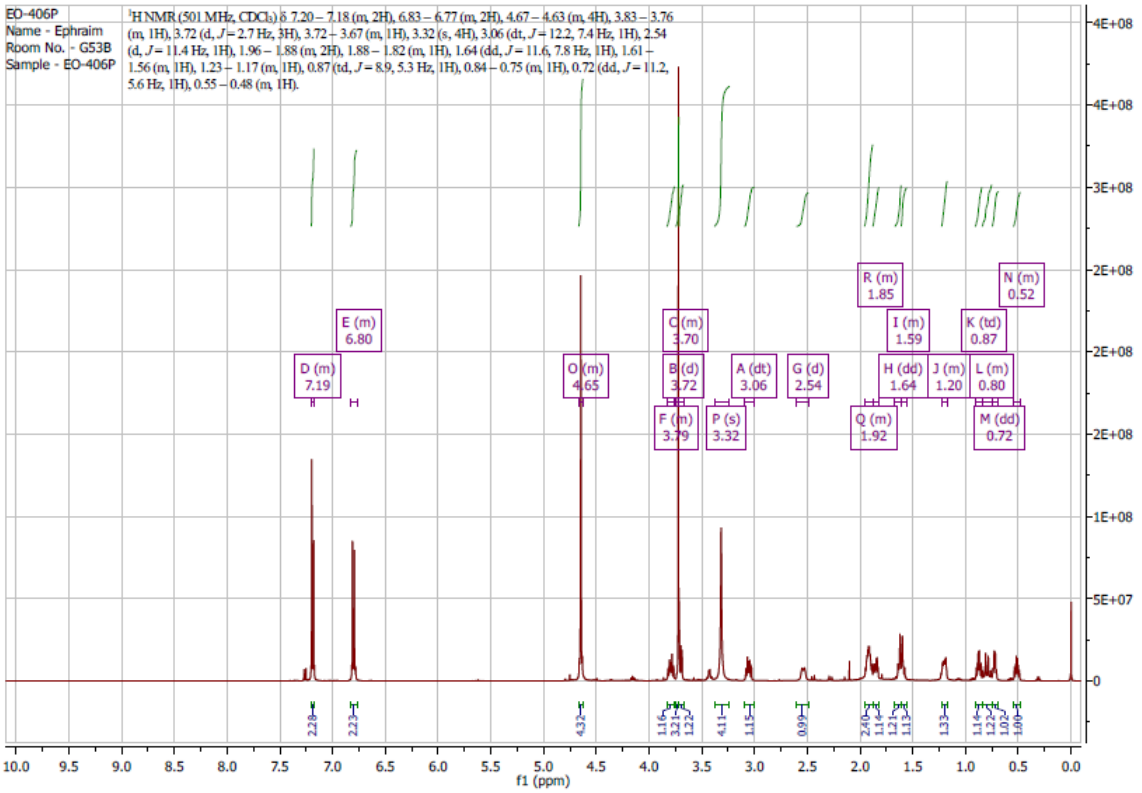

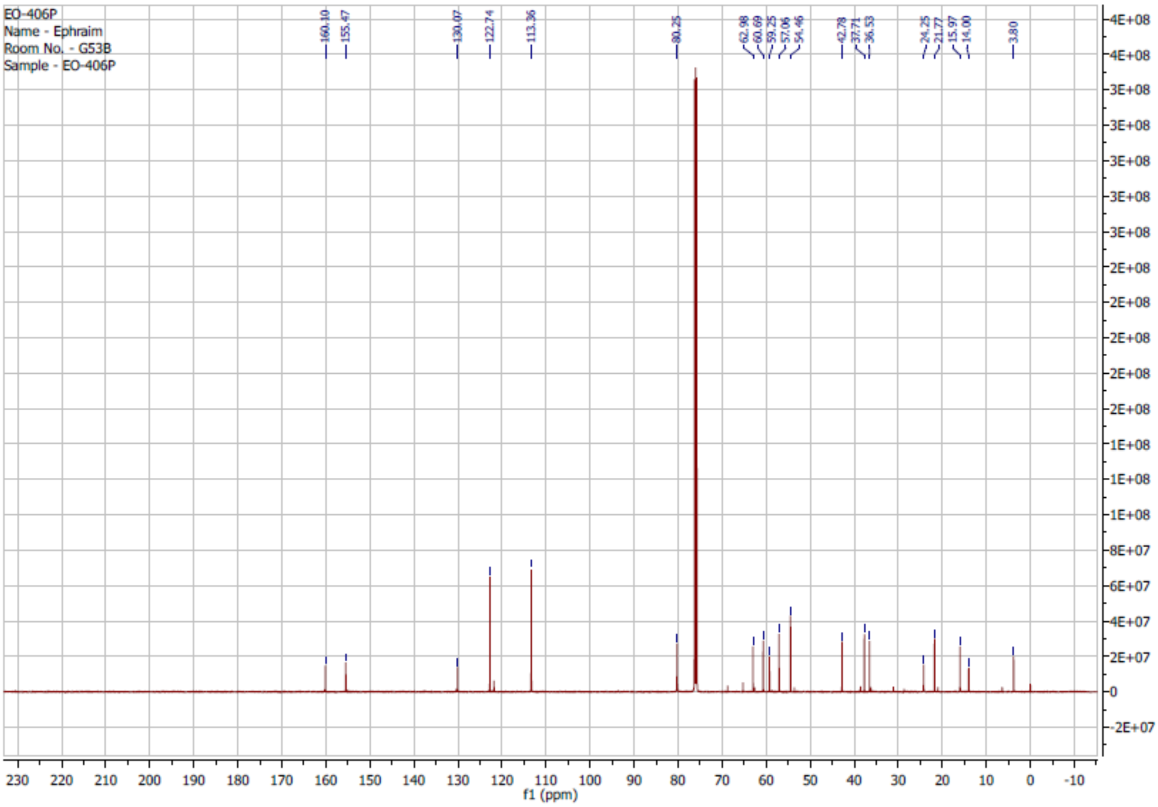

(6a*R,8a*S**,11a*R**,11b*S**)-10-Benzyl-8-hydroxy-6-isopropyldecahydro-1*H*-pyrrolo[1’,2’:3,4]imidazo[4,5-*e*]isoindol-5(6*H*)-one 47**

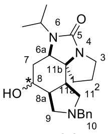

dr 3:2

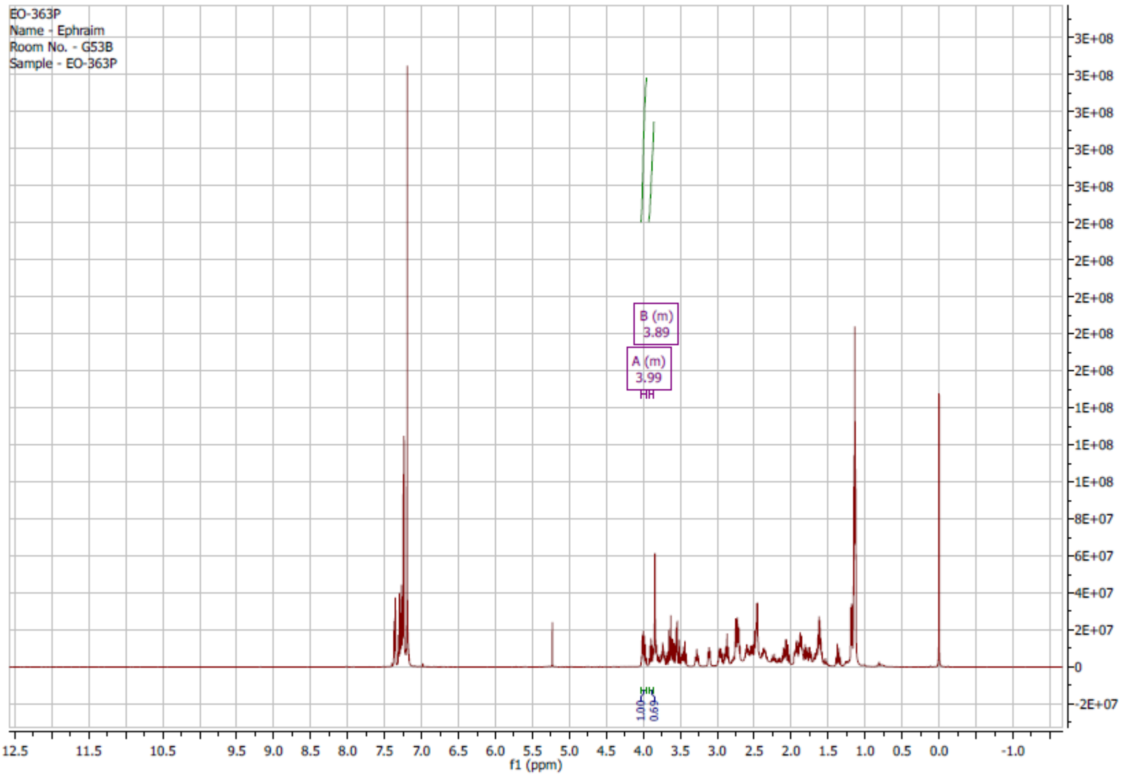

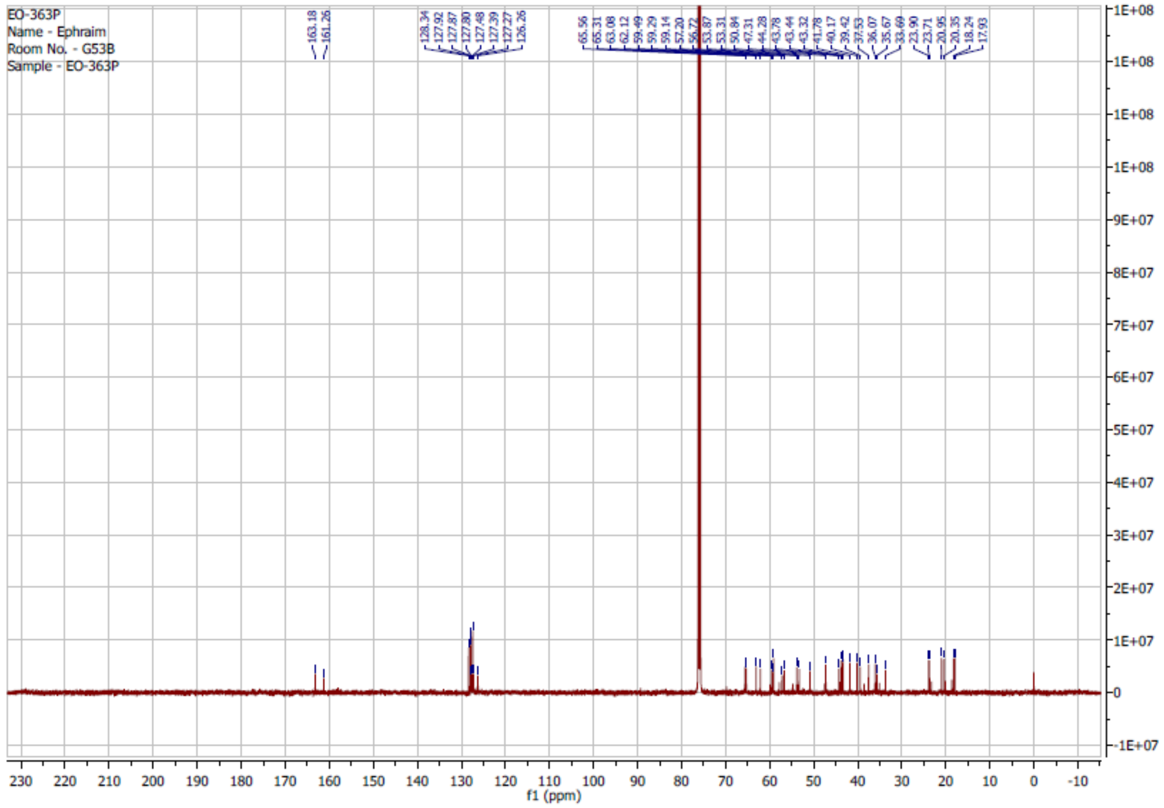

(6a*R**,8*R**,8a*R**,11a*S**,11b*S**)-10-Benzyl-8-hydroxy-6-(4-methoxyphenyl)decahydro-1*H*-pyrrolo[1',2':3,4]imidazo[4,5-*e*]isoindol-5(6*H*)-one 48

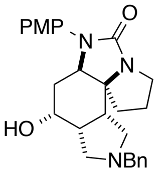

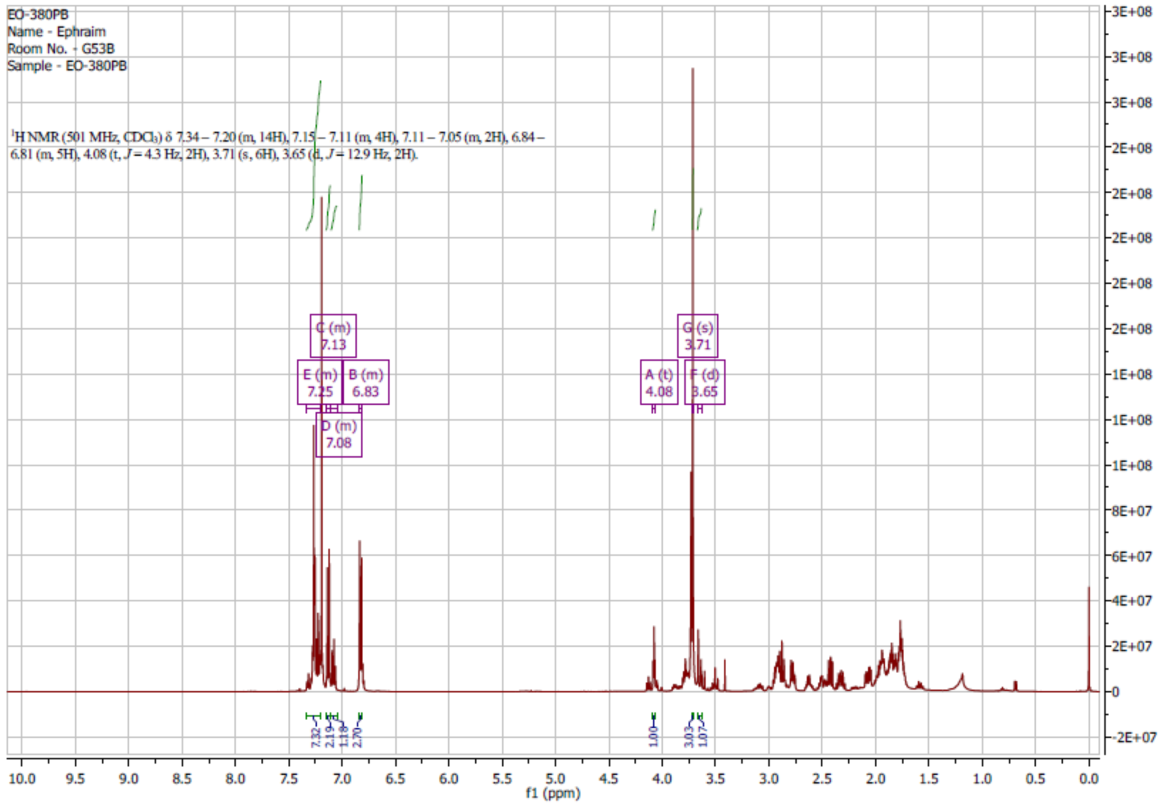

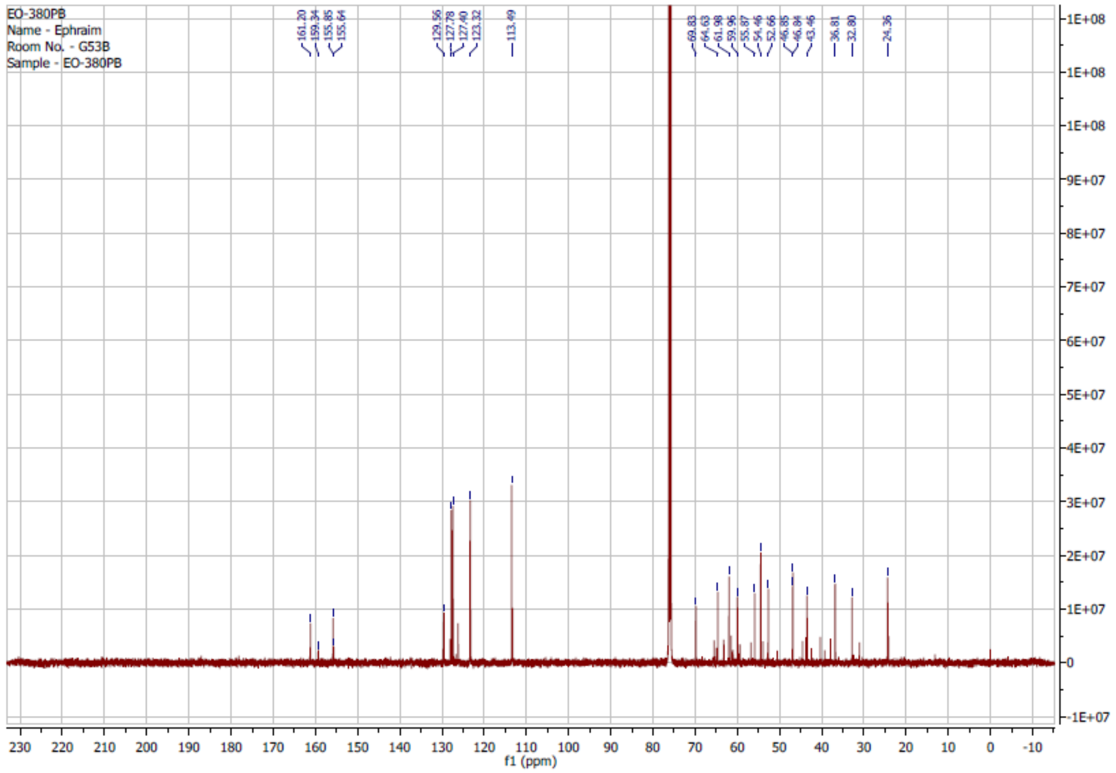

(6a*R,8*S**,8a*S**,11a*R**,11b*S**)-8-Hydroxy-6-(4-methoxyphenyl)decahydro-1*H*-pyrrolo[1’,2’:3,4]imidazo[4,5-*e*]isoindol-5(6*H*)-one 49**

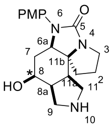

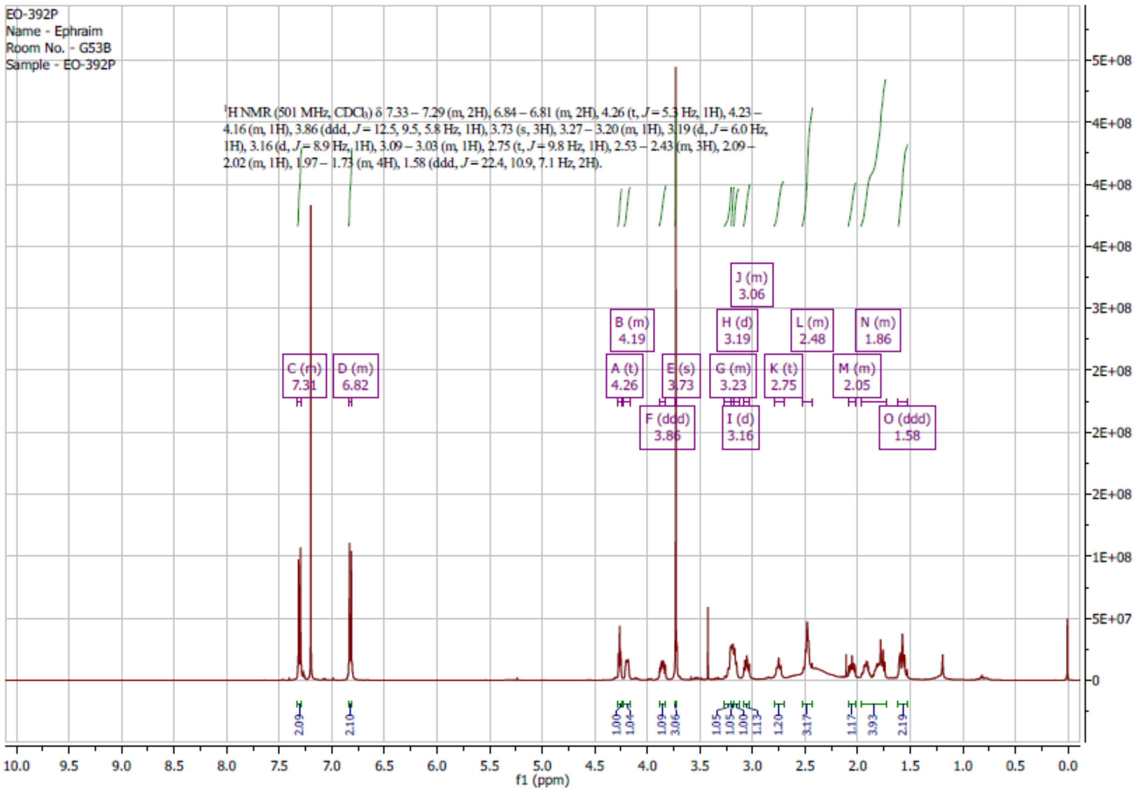

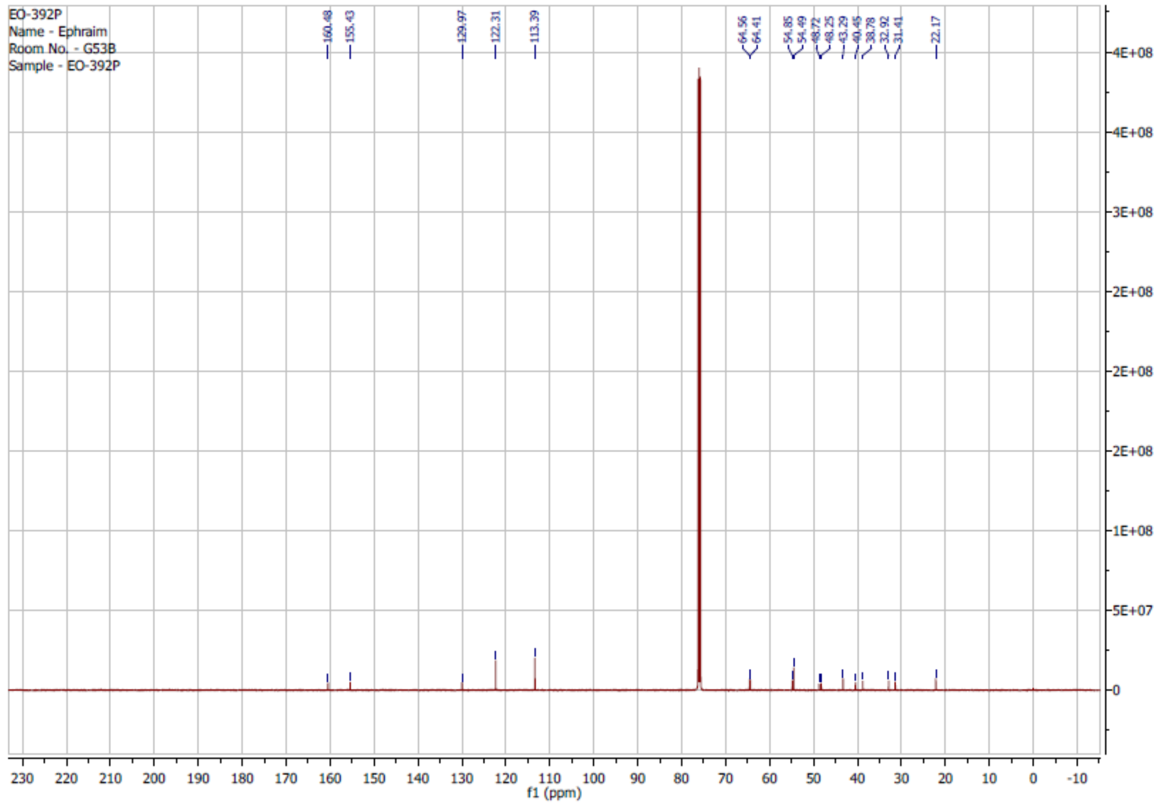

(6a*R,8*R**,8a*R**,11a*S**,11b*S**)-8-Hydroxy-6-(4-methoxyphenyl)decahydro-1*H*-pyrrolo[1′,2′:3,4]imidazo[4,5-*e*]isoindol-5(6*H*)-one 50**

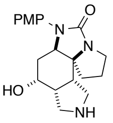

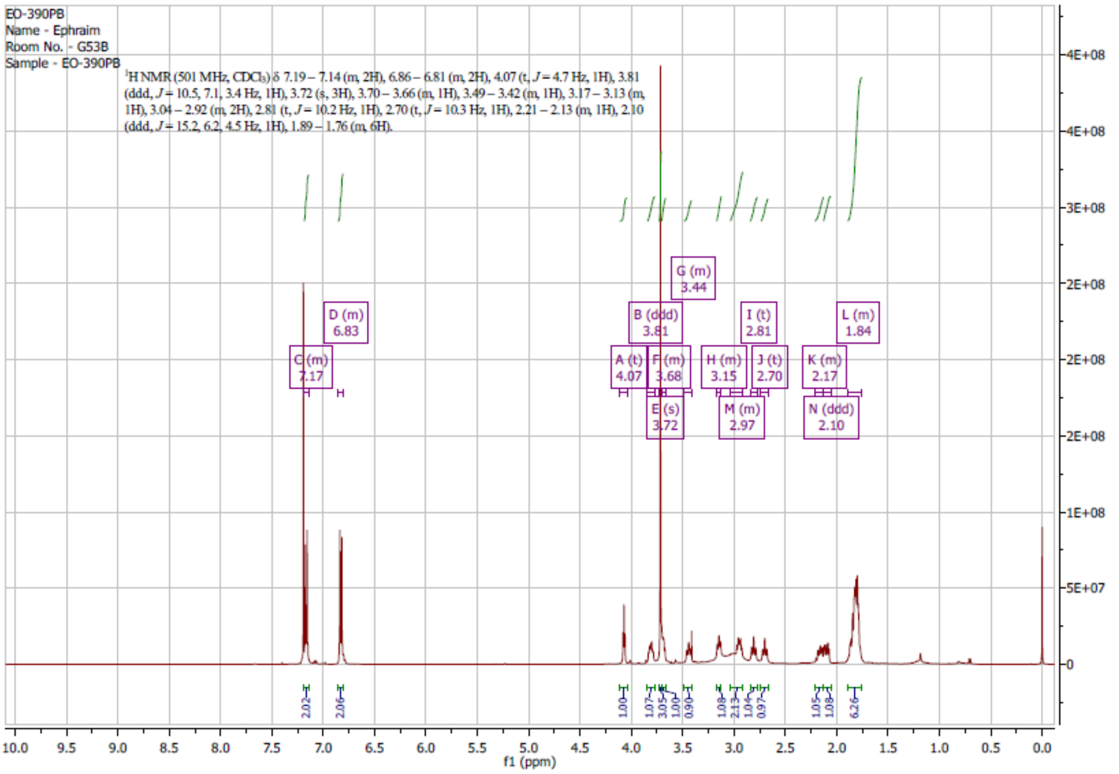

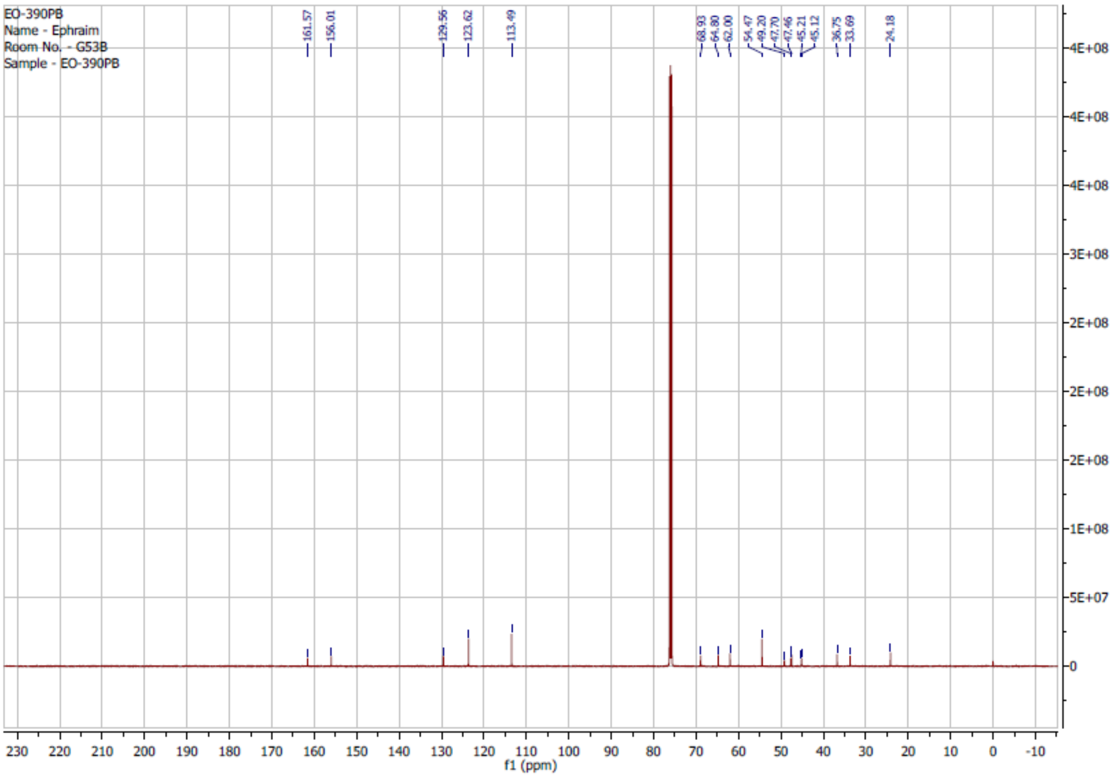

(6a*R,8*S**,8a*R**,11a*S**,11b*S**)-8-hydroxy-6-(4-methoxyphenyl)decahydro-1*H*-pyrrolo[1’,2’:3,4]imidazo[4,5-*e*]isoindol-5(6*H*)-one 51**

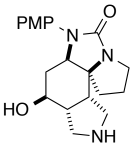

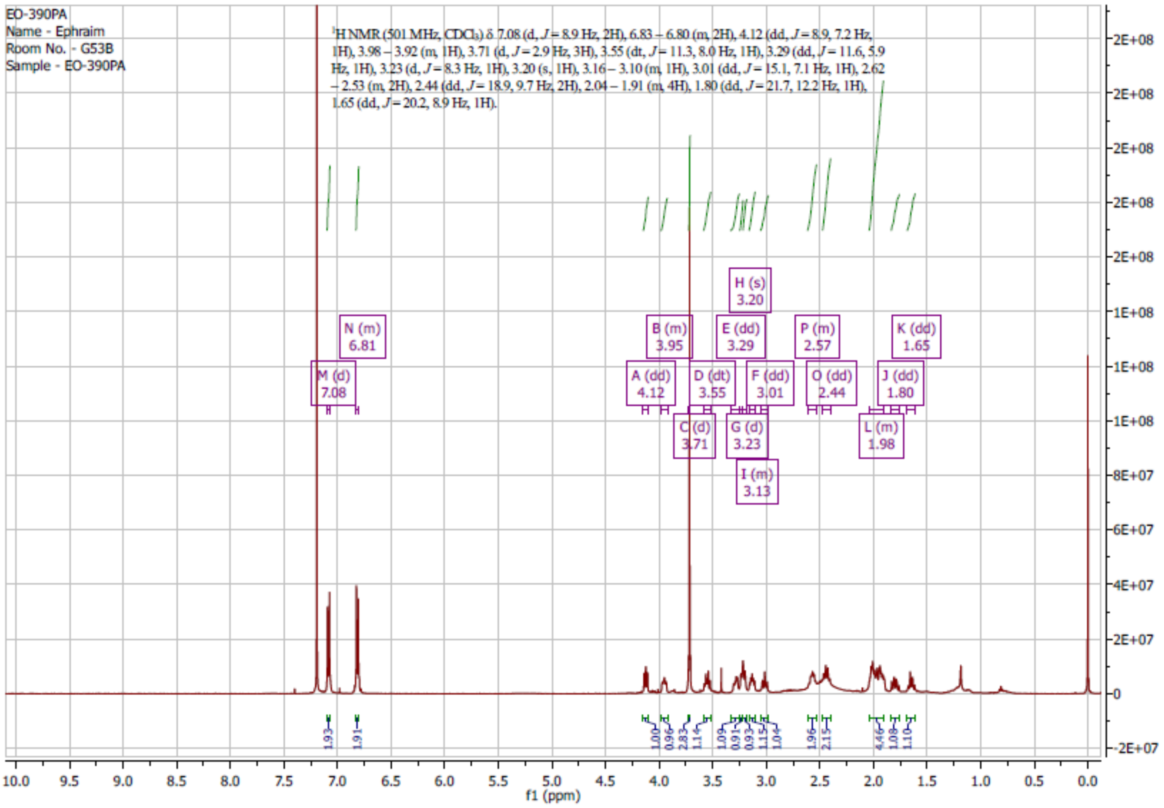

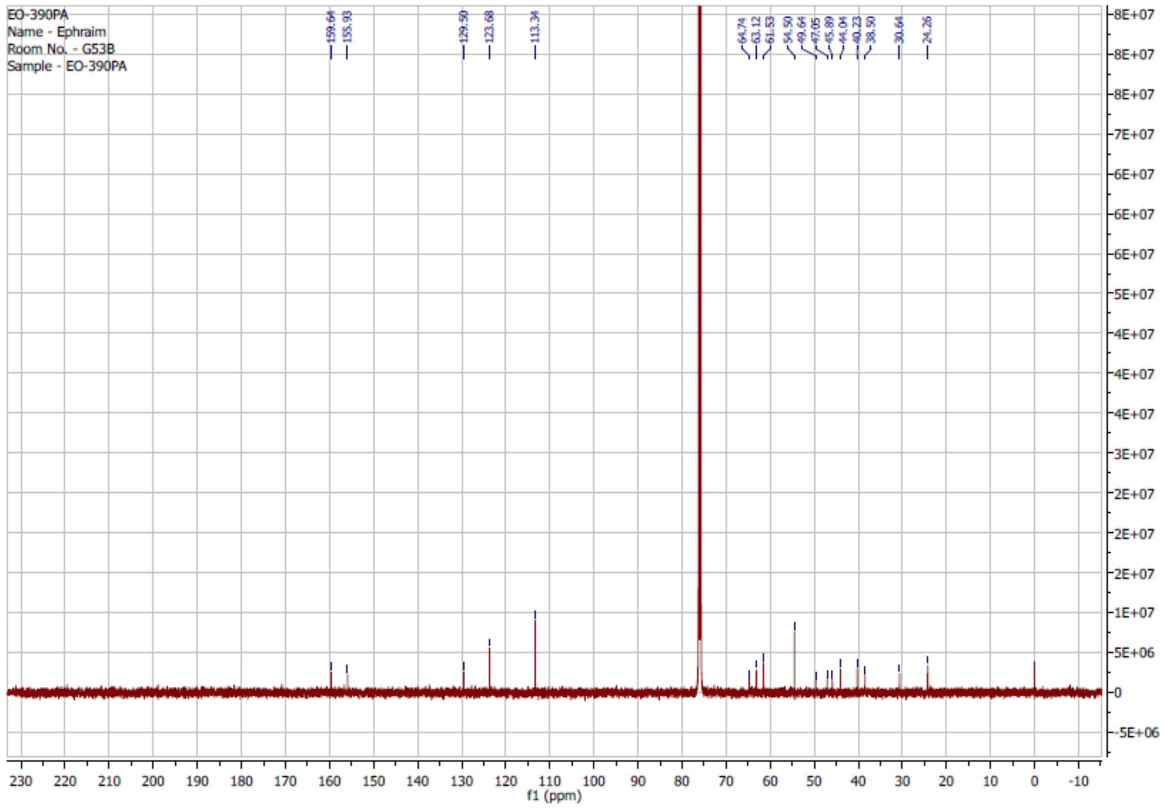

(7a*R,9*R**,11a*S**)-7-(4-Methoxyphenyl)-6-oxodecahydro-1H-pyrrolo[1,2-*d*]quinoxalin-9-yl (3-fluorophenyl)carbamate 52**

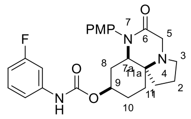

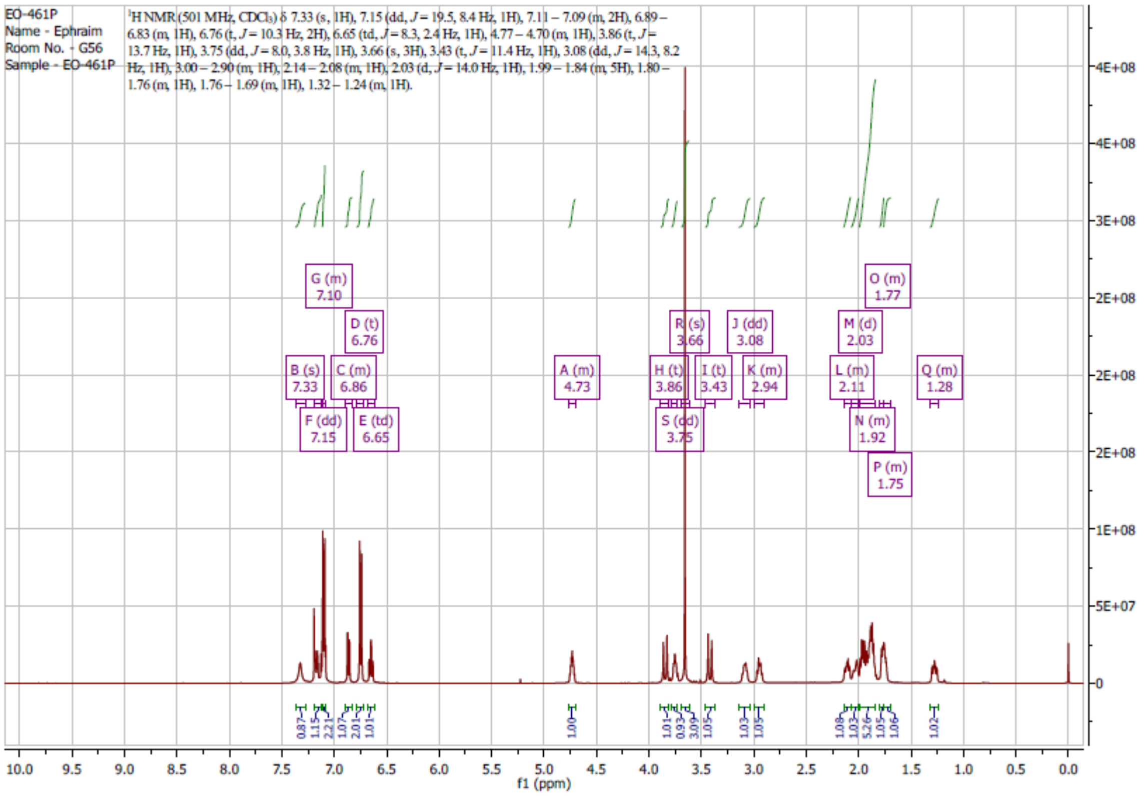

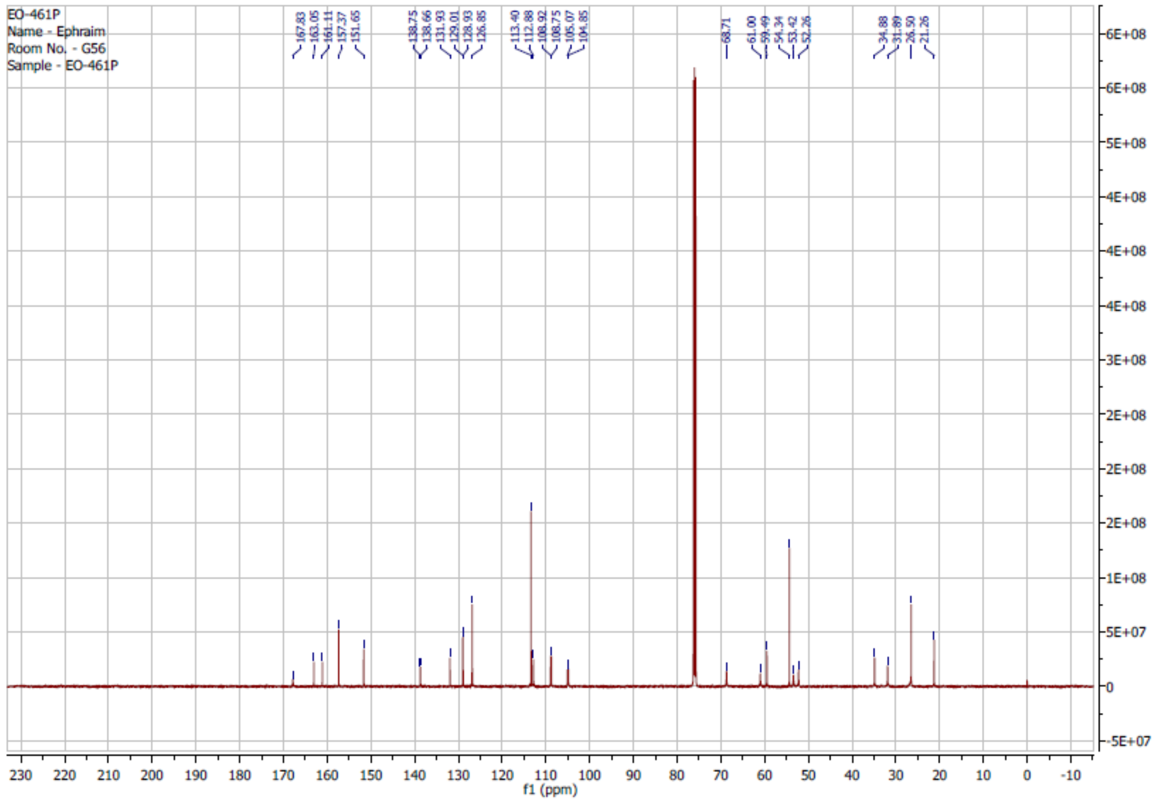

Cell painting assay

The described assay follows closely the method described by Bray et al.[6]

Initially, 5 µl U2OS medium were added to each well of a 384-well plate (PerkinElmer CellCarrier-384 Ultra). Subsequently, U2OS cell were seeded with a density of 1600 cells per well in 20 µl medium. The plate was incubated for 10 min at the ambient temperature, followed by an additional 4 h incubation (37 °C, 5% CO2). Compound treatment was performed with the Echo 520 acoustic dispenser (Labcyte) at final concentrations of 10 µM, 3 µM or 1 µM. Incubation with compound was performed for 20 h (37 °C, 5% CO2). Subsequently, mitochondria were stained with Mito Tracker Deep Red (Thermo Fisher Scientific, Cat. No. M22426). The Mito Tracker Deep Red stock solution (1 mM) was diluted to a final concentration of 100 nM in prewarmed medium. The medium was removed from the plate leaving 10 µl residual volume and 25 µl of the Mito Tracker solution were added to each well. The plate was incubated for 30 min in darkness (37 °C, 5% CO2). To fix the cells 7 µl of 18.5 % formaldehyde in PBS were added, resulting in a final formaldehyde concentration of 3.7 %. Subsequently, the plate was incubated for another 20 min in darkness (RT) and washed three times with 70 µl of PBS. (Biotek Washer Elx405). Cells were permeabilized by addition of 25 µl 0.1% Triton X-100 to each well, followed by 15 min incubation (RT) in darkness. The cells were washed three times with PBS leaving a final volume of 10 µl. To each well 25 µl of a staining solution were added, which contains 1% BSA, 5 µl/ml Phalloidin (Alexa594 conjugate, Thermo Fisher Scientific, A12381), 25 µg/ml Concanavalin A (Alexa488 conjugate, Thermo Fisher Scientific, Cat. No. C11252), 5 µg/ml Hoechst 33342 (Sigma, Cat. No. B2261-25mg), 1.5 µg/ml WGA-Alexa594 conjugate (Thermo Fisher Scientific, Cat. No. W11262) and 1.5 µM SYTO 14 solution (Thermo Fisher Scientific, Cat. No. S7576). The plate is incubated for 30 min (RT) in darkness and washed three times with 70 µl PBS. After the final washing step, the PBS was not aspirated. The plates were sealed and centrifuged for 1 min at 500 rpm.

The plates were prepared in triplicates with shifted layouts to reduce plate effects and imaged using a Micro XL High-Content Screening System (Molecular Devices) in 5 channels (DAPI: Ex350-400/ Em410-480; FITC: Ex470-500/ Em510-540; Spectrum Gold: Ex520-545/ Em560-585; TxRed: Ex535-585/ Em600-650; Cy5: Ex605-650/ Em670-715) with 9 sites per well and 20x magnification (binning 2).

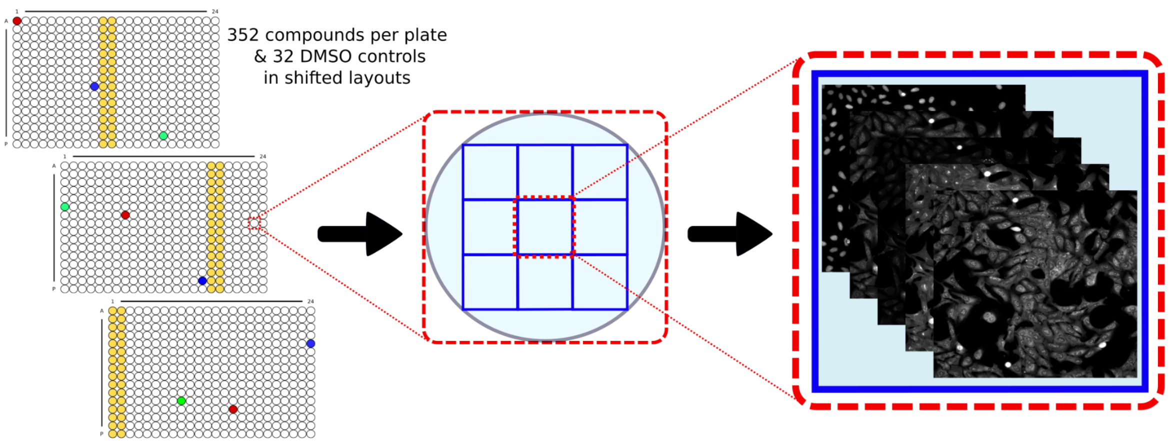

The generated images were processed with the *CellProfiler* package (https://cellprofiler.org/, version 3.0.0) on a computing cluster of the Max Planck Society to extract 1716 cell features per microscope site. The data was then further aggregated as medians per well (9 sites -> 1 well), then over the three replicates.

Further analysis was performed with custom *Python* (https://www.python.org/) scripts using the *Pandas* (https://pandas.pydata.org/) and *Dask* (https://dask.org/) data processing libraries as well as the *Scientific Python* (https://scipy.org/) package (separate publication to follow).
From the total set of 1716 features, a subset of highly reproducible and robust features was determined using the procedure described by Woehrmann et al.[7] in the following way:
Two biological repeats of one plate containing reference compounds were analysed. For every feature, its full profile over each whole plate was calculated. If the profiles from the two repeats showed a similarity >= 0.8 (see below), the feature was added to the set.

This procedure was only performed once and resulted in a set of 579 robust features out of the total of 1716 that was used for all further analyses.

Determination of reproducible Features

1716	*Determined by CellProfiler*
↓	*Keep features that have a minimum correlation of 0.80 between repeats for all cpds.*
579	*Final set of relevant features. Used for all further analyses*

The phenotypic profiles were compiled from the Z-scores of all individual cellular features, where the Z-score is a measure of how far away a data point is from a median value.

Specifically, Z-scores of test compounds were calculated relative to the Median of DMSO controls. Thus, the Z-score of a test compound defines how many MADs (Median Absolute Deviations) the measured value is away from the Median of the controls as illustrated by the following formula:

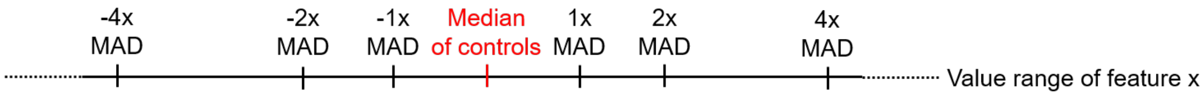

$$z\_score = \frac{value_{meas.} - Median_{Controls}}{MAD_{Controls}}$$

The phenotypic compound profile is then determined as the list of Z-scores of all features for one compound.

In addition to the phenotypic profile, an induction value was determined for each compound as the fraction of significantly changed features, in percent:

$$Induction\ [\%] = \frac{number\ of\ features\ with\ abs.\ values > 3}{total\ number\ of\ features}$$

Similarities of phenotypic profiles were calculated from the correlation distances between two profiles (https://docs.scipy.org/doc/scipy/reference/generated/scipy.spatial.distance.correlation.html; Similarity = 1 - Correlation Distance).

An example for two compounds with highly similar profiles (96% similarity):

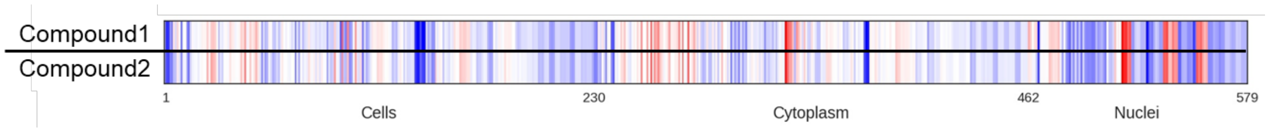

An example for two compounds with low similarity profiles (0% similarity):

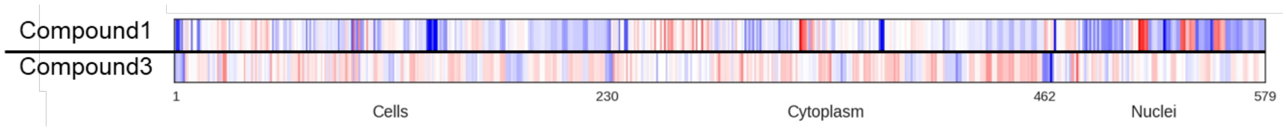

Each colored band represents one Z-score of a feature.

X-Ray Structures

X-ray structure of compound 6b

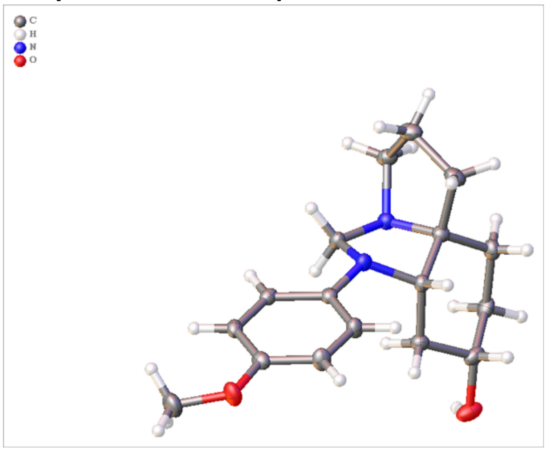

CCDC deposition number: 2174964

Empirical formula	$C_{17}H_{24}N_2O_2$
Formula weight	288.38
Temperature/K	120.0(3)
Crystal system	monoclinic
Space group	$P2_1$
a/Å	7.5005(2)
b/Å	8.1198(2)
c/Å	12.2612(4)
α/°	90
β/°	90.254(3)
γ/°	90
Volume/Å^3	746.73(4)
Z	2
ρ_{calc}g/cm^3	1.283
μ/mm^{-1}	0.669
F(000)	312.0
Crystal size/mm^3	0.11 × 0.08 × 0.04
Radiation	CuKα (λ = 1.54184)
2Θ range for data collection/°	7.21 to 146.764
Index ranges	-9 ≤ h ≤ 9, -9 ≤ k ≤ 9, -14 ≤ l ≤ 15
Reflections collected	5901
Independent reflections	2888 [R_{int} = 0.0311, R_{sigma} = 0.0416]
Data/restraints/parameters	2888/1/195
Goodness-of-fit on F^2	1.047
Final R indexes [I>=2σ (I)]	R_1 = 0.0382, wR_2 = 0.0881
Final R indexes [all data]	R_1 = 0.0434, wR_2 = 0.0908
Largest diff. peak/hole / e Å^{-3}	0.21/-0.17
Flack parameter	0.0(2)

X-ray structure of compound 8a

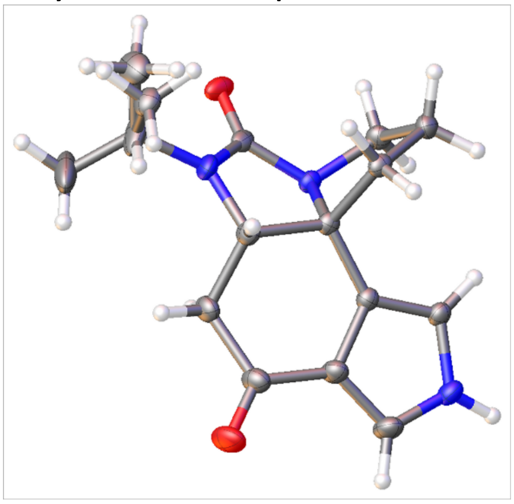

CCDC deposition number: 2175091

Empirical formula	$C_{15}H_{19}N_3O_2$
Formula weight	273.33
Temperature/K	124.99(10)
Crystal system	monoclinic
Space group	$P2_1/c$
a/Å	13.3373(6)
b/Å	7.4531(2)
c/Å	14.1571(6)
α/°	90
β/°	109.342(4)
γ/°	90
Volume/Å^3	1327.85(9)
Z	4
ρ_{calc}g/cm^3	1.367
μ/mm^{-1}	0.749
F(000)	584.0
Crystal size/mm^3	0.13 × 0.06 × 0.04
Radiation	CuKα (λ = 1.54184)
2Θ range for data collection/°	7.024 to 149.502
Index ranges	-16 ≤ h ≤ 16, -9 ≤ k ≤ 9, -17 ≤ l ≤ 17
Reflections collected	5579
Independent reflections	5579 [R_{int} = ?, R_{sigma} = 0.0274]
Data/restraints/parameters	5579/18/198
Goodness-of-fit on F^2	1.127
Final R indexes [I>=2σ (I)]	R_1 = 0.0656, wR_2 = 0.2122
Final R indexes [all data]	R_1 = 0.0810, wR_2 = 0.2180
Largest diff. peak/hole / e Å-3	0.56/-0.47

X-ray structure of compound 10a

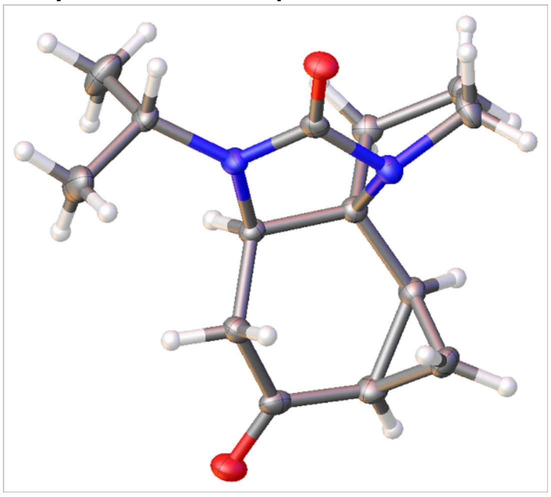

CCDC deposition number: 2175090

Empirical formula	$C_{14}H_{20}N_2O_2$
Formula weight	248.32
Temperature/K	124.97(12)
Crystal system	orthorhombic
Space group	$P2_12_12_1$
a/Å	6.3405(2)
b/Å	12.4417(4)
c/Å	15.9687(6)
α/°	90
β/°	90
γ/°	90
Volume/Å^3	1259.72(8)
Z	4
ρ_{calc}g/cm^3	1.309
μ/mm^{-1}	0.707
F(000)	536.0
Crystal size/mm^3	0.23 × 0.03 × 0.02
Radiation	CuKα (λ = 1.54184)
2Θ range for data collection/°	9.01 to 146.926
Index ranges	-7 ≤ h ≤ 7, -10 ≤ k ≤ 14, -19 ≤ l ≤ 19
Reflections collected	4744
Independent reflections	2411 [R_{int} = 0.0349, R_{sigma} = 0.0526]
Data/restraints/parameters	2411/0/165
Goodness-of-fit on F^2	1.037
Final R indexes [I>=2σ (I)]	R_1 = 0.0400, wR_2 = 0.0898
Final R indexes [all data]	R_1 = 0.0478, wR_2 = 0.0935
Largest diff. peak/hole / e Å^{-3}	0.24/-0.18
Flack parameter	0.5

X-ray structure of compound 12b

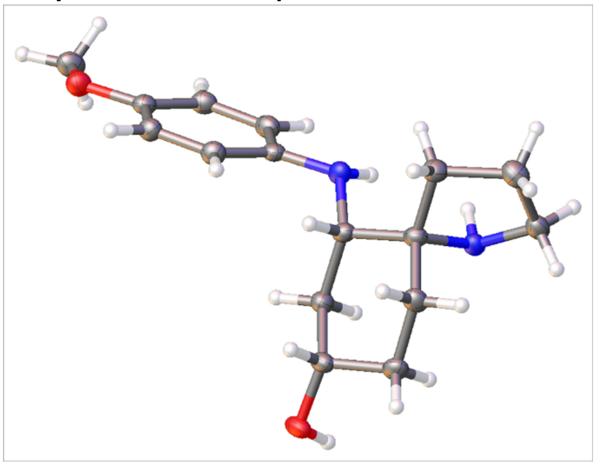

CCDC deposition number: 2175097	
Empirical formula	$C_{16}H_{24}N_2O_2$
Formula weight	276.37
Temperature/K	119.99(19)
Crystal system	monoclinic
Space group	$P2_1$
a/Å	8.2523(2)
b/Å	7.06280(10)
c/Å	12.4127(4)
α/°	90
β/°	90.803(2)
γ/°	90
Volume/Å^3	723.40(3)
Z	2
ρ_{calc}g/cm^3	1.269
μ/mm^{-1}	0.666
F(000)	300.0
Crystal size/mm^3	0.12 × 0.04 × 0.03
Radiation	CuKα (λ = 1.54184)
2Θ range for data collection/°	7.122 to 147.266
Index ranges	-10 ≤ h ≤ 10, -8 ≤ k ≤ 8, -14 ≤ l ≤ 14
Reflections collected	5854
Independent reflections	5854 [R_{int} = ?, R_{sigma} = 0.0326]
Data/restraints/parameters	5854/1/195
Goodness-of-fit on F^2	0.988
Final R indexes [I>=2σ (I)]	R_1 = 0.0375, wR_2 = 0.0919
Final R indexes [all data]	R_1 = 0.0446, wR_2 = 0.0939
Largest diff. peak/hole / e Å^{-3}	0.17/-0.17
Flack parameter	-0.13(16)

X-ray structure of compound 13c

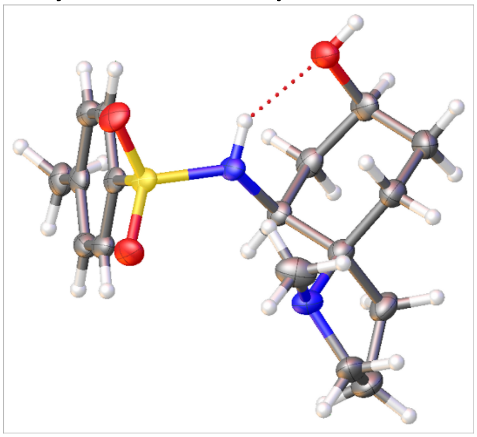

CCDC deposition number: 2175095

Empirical formula	$C_{17}H_{26}N_2O_3S$
Formula weight	338.46
Temperature/K	124.96(14)
Crystal system	monoclinic
Space group	$P2_1/c$
a/Å	16.9748(10)
b/Å	7.7610(3)
c/Å	13.4218(7)
α/°	90
β/°	104.680(5)
γ/°	90
Volume/Å^3	1710.49(15)
Z	4
ρ_{calc}g/cm^3	1.314
μ/mm^{-1}	1.818
F(000)	728.0
Crystal size/mm^3	0.22 × 0.14 × 0.02
Radiation	CuKα (λ = 1.54184)
2Θ range for data collection/°	10.776 to 147.244
Index ranges	$-21 \le h \le 20$, $-9 \le k \le 9$, $-12 \le l \le 15$
Reflections collected	12821
Independent reflections	3312 [R_{int} = 0.0556, R_{sigma} = 0.0472]
Data/restraints/parameters	3312/0/218
Goodness-of-fit on F^2	1.030
Final R indexes [I>=2σ (I)]	R_1 = 0.0447, wR_2 = 0.1035
Final R indexes [all data]	R_1 = 0.0628, wR_2 = 0.1118
Largest diff. peak/hole / e Å^{-3}	0.38/-0.36

X-ray structure of compound 17a

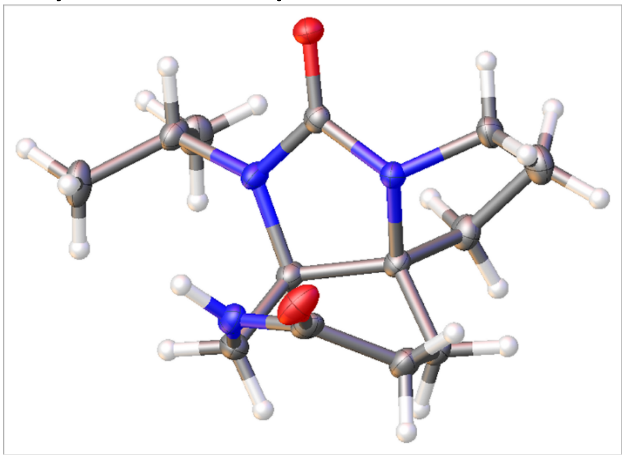

CCDC deposition number: 2175092

Empirical formula	$C_{13}H_{21}N_3O_2$
Formula weight	251.33
Temperature/K	120.01(10)
Crystal system	monoclinic
Space group	$P2_1/c$
a/Å	9.7156(4)
b/Å	11.9950(4)
c/Å	11.0289(4)
α/°	90
β/°	93.242(4)
γ/°	90
Volume/Å^3	1283.23(9)
Z	4
ρ_{calc}g/cm^3	1.301
μ/mm^{-1}	0.720
F(000)	544.0
Crystal size/mm^3	0.24 × 0.14 × 0.09
Radiation	CuKα (λ = 1.54184)
2Θ range for data collection/°	9.116 to 146.28
Index ranges	$-10 \le h \le 11, -14 \le k \le 14, -13 \le l \le 12$
Reflections collected	10753
Independent reflections	2482 [R_{int} = 0.0361, R_{sigma} = 0.0265]
Data/restraints/parameters	2482/0/169
Goodness-of-fit on F^2	1.065
Final R indexes [I>=2σ (I)]	R_1 = 0.0366, wR_2 = 0.0900
Final R indexes [all data]	R_1 = 0.0418, wR_2 = 0.0942
Largest diff. peak/hole / e Å^{-3}	0.23/-0.21

X-ray structure of compound 18a

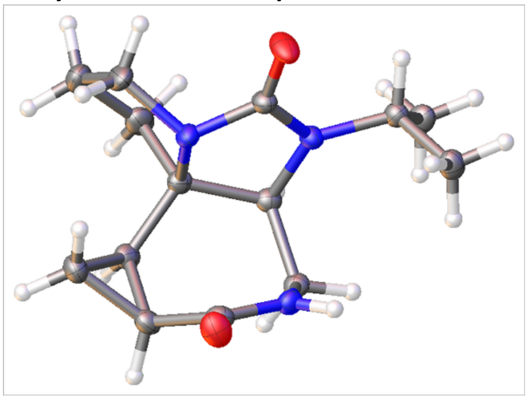

CCDC deposition number: 2175093

Empirical formula	$C_{14}H_{21}N_3O_2$
Formula weight	263.34
Temperature/K	124.98(10)
Crystal system	orthorhombic
Space group	$P2_12_12_1$
a/Å	5.99390(13)
b/Å	11.2042(2)
c/Å	20.0696(4)
α/°	90
β/°	90
γ/°	90
Volume/Å^3	1347.80(5)
Z	4
ρ_{calc}g/cm^3	1.298
μ/mm^{-1}	0.712
F(000)	568.0
Crystal size/mm^3	0.14 × 0.11 × 0.07
Radiation	CuKα (λ = 1.54184)
2Θ range for data collection/°	8.812 to 147.012
Index ranges	-6 ≤ h ≤ 7, -13 ≤ k ≤ 12, -24 ≤ l ≤ 21
Reflections collected	4717
Independent reflections	2638 [R_{int} = 0.0327, R_{sigma} = 0.0466]
Data/restraints/parameters	2638/0/178
Goodness-of-fit on F^2	1.057
Final R indexes [I>=2σ (I)]	R_1 = 0.0349, wR_2 = 0.0830
Final R indexes [all data]	R_1 = 0.0390, wR_2 = 0.0853
Largest diff. peak/hole / e Å^{-3}	0.18/-0.17
Flack parameter	0.5

X-ray structure of compound 18a'

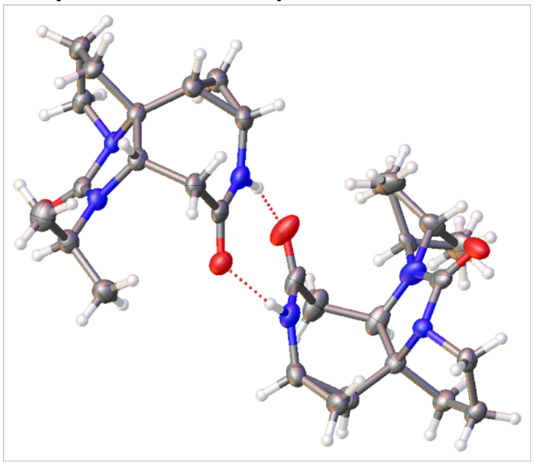

CCDC deposition number: 2224747

Empirical formula	$C_{14}H_{21}N_3O_2$
Formula weight	263.34
Temperature/K	120.00(10)
Crystal system	monoclinic
Space group	$P2_1/n$
a/Å	11.2468(3)
b/Å	14.3184(3)
c/Å	17.0737(4)
α/°	90
β/°	105.488(3)
γ/°	90
Volume/Å^3	2649.63(11)
Z	8
ρ_{calc}g/cm^3	1.320
μ/mm^{-1}	0.724
F(000)	1136.0
Crystal size/mm^3	0.43 × 0.36 × 0.28
Radiation	CuKα (λ = 1.54184)
2Θ range for data collection/°	8.186 to 146.472
Index ranges	-13 ≤ h ≤ 10, -17 ≤ k ≤ 17, -18 ≤ l ≤ 19
Reflections collected	22775
Independent reflections	5158 [R_{int} = 0.0263, R_{sigma} = 0.0178]
Data/restraints/parameters	5158/15/364
Goodness-of-fit on F^2	1.035
Final R indexes [I>=2σ (I)]	R_1 = 0.0486, wR_2 = 0.1180
Final R indexes [all data]	R_1 = 0.0530, wR_2 = 0.1218
Largest diff. peak/hole / e Å^{-3}	0.48/-0.45

X-ray structure of compound 32

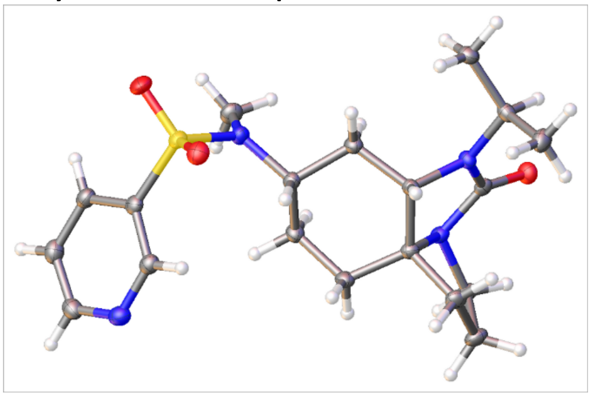

CCDC deposition number: 2175096

Empirical formula	$C_{19}H_{28}N_4O_3S$
Formula weight	392.51
Temperature/K	125.00(10)
Crystal system	monoclinic
Space group	$P2_1/n$
a/Å	12.63984(16)
b/Å	11.19439(13)
c/Å	13.94279(17)
α/°	90
β/°	104.3374(13)
γ/°	90
Volume/Å^3	1911.39(4)
Z	4
ρ_{calc}g/cm^3	1.364
μ/mm^{-1}	1.737
F(000)	840.0
Crystal size/mm^3	0.15 × 0.13 × 0.11
Radiation	CuKα (λ = 1.54184)
2Θ range for data collection/°	8.46 to 147.156
Index ranges	$-15 \le h \le 15$, $-13 \le k \le 13$, $-17 \le l \le 17$
Reflections collected	14294
Independent reflections	3795 [R_{int} = 0.0314, R_{sigma} = 0.0250]
Data/restraints/parameters	3795/0/247
Goodness-of-fit on F^2	1.043
Final R indexes [I>=2σ (I)]	R_1 = 0.0324, wR_2 = 0.0830
Final R indexes [all data]	R_1 = 0.0374, wR_2 = 0.0873
Largest diff. peak/hole / e Å^{-3}	0.31/-0.39

X-ray structure of compound 40

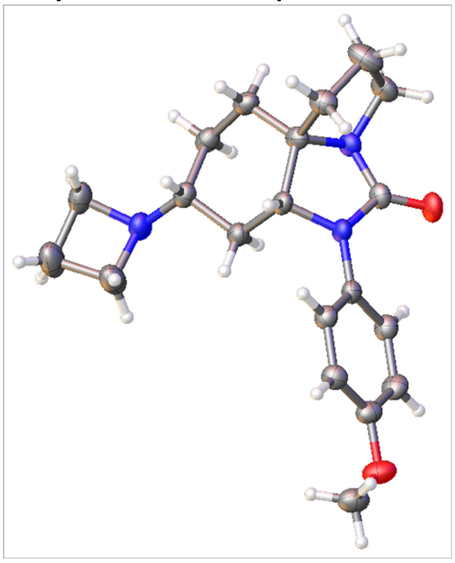

CCDC deposition number: 2175098

Empirical formula	$C_{20}H_{27}N_3O_2$
Formula weight	341.44
Temperature/K	125.01(10)
Crystal system	monoclinic
Space group	$P2_1/c$
a/Å	6.46670(10)
b/Å	10.7945(2)
c/Å	26.3038(4)
α/°	90
β/°	94.5550(10)
γ/°	90
Volume/Å^3	1830.33(5)
Z	4
ρ_{calc}g/cm^3	1.239
μ/mm^{-1}	0.643
F(000)	736.0
Crystal size/mm^3	0.17 × 0.12 × 0.09
Radiation	CuKα (λ = 1.54184)
2Θ range for data collection/°	8.86 to 147.218
Index ranges	-7 ≤ h ≤ 7, -13 ≤ k ≤ 13, -30 ≤ l ≤ 32
Reflections collected	17523
Independent reflections	3556 [R_{int} = 0.0355, R_{sigma} = 0.0231]
Data/restraints/parameters	3556/0/227
Goodness-of-fit on F^2	1.021
Final R indexes [I>=2σ (I)]	R_1 = 0.0411, wR_2 = 0.1020
Final R indexes [all data]	R_1 = 0.0494, wR_2 = 0.1084
Largest diff. peak/hole / e Å^{-3}	0.24/-0.20

X-ray structure of compound S8

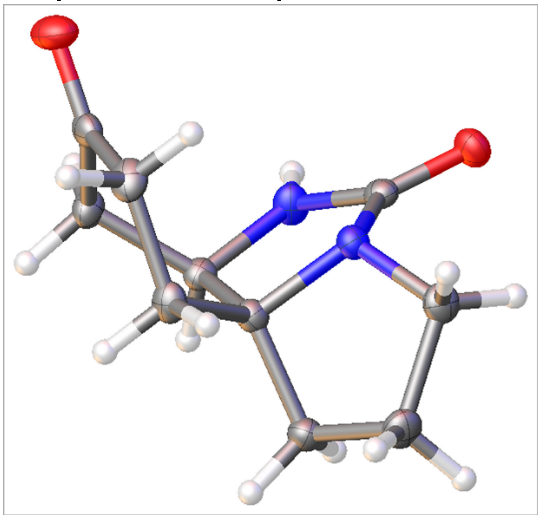

CCDC deposition number: 2174966

Empirical formula	$C_{10}H_{14}N_2O_2$
Formula weight	194.23
Temperature/K	125.01(10)
Crystal system	monoclinic
Space group	$P2_1/n$
a/Å	8.5908(3)
b/Å	10.2888(4)
c/Å	10.3886(4)
α/°	90
β/°	96.630(4)
γ/°	90
Volume/Å^3	912.10(6)
Z	4
ρ_{calc}g/cm^3	1.414
μ/mm^{-1}	0.817
F(000)	416.0
Crystal size/mm^3	0.12 × 0.08 × 0.05
Radiation	CuKα (λ = 1.54184)
2Θ range for data collection/°	12.148 to 147.18
Index ranges	-8 ≤ h ≤ 10, -12 ≤ k ≤ 12, -12 ≤ l ≤ 8
Reflections collected	3562
Independent reflections	3562 [R_{int} = ?, R_{sigma} = 0.0418]
Data/restraints/parameters	3562/0/132
Goodness-of-fit on F^2	0.945
Final R indexes [I>=2σ (I)]	R_1 = 0.0435, wR_2 = 0.1130
Final R indexes [all data]	R_1 = 0.0603, wR_2 = 0.1197
Largest diff. peak/hole / e Å^{-3}	0.32/-0.24

References

[1] H. Wang, Y. Ma, H. Tian, A. Yu, J. Chang, Y. Wu, *Tetrahedron*, **2014**, *70*, 2669–2673.
[2] J. T. Kendall, *J. Label. Compd. Radiopharm.*, **2000**, *43*, 505–514.
[3] Q. Zhou, T. A. Reekie, R. H. Abbassi, D. I. Venkata, J. S. Font, R. M. Ryan, L. M. Rendina, L. Munoz, M. Kassiou, *Aust. J. Chem.*, **2018**, *71*, 789-797.
[4] A. Aimon, M. J. Dow, A. R. Hanby, E. A. Okolo, C. M. Pask, A. Nelson, S. P. Marsden, *Chem. Commun.*, **2023**, *59*, 607-610.
[5] I. Colomer, C. J. Empson, P. Craven, Z. Owen, R. G. Doveston, I. Churcher, S. P. Marsden, A. Nelson, *Chem. Commun.*, **2016**, *52*, 7209-7212.
[6] M.-A. Bray, S. Singh, H. Han, C. T. Davis, B. Borgeson, C. Hartland, M. Kost-Alimova, S. M. Gustafsdottir, C. C. Gibson, A. E. Carpenter, *Nature Protocols*, **2016**, *11*, 1757–1774.
[7] M. H. Woehrmann, W. M. Bray, J. K. Durbin, S. C. Nisam, A. K. Michael, E. Glassey, J. M. Stuart, R. S. Lokey, *Mol. BioSyst*., **2013**, *9*, 2604–2617.
